# Restructuring Antiviral Quinazolinone Frameworks to Derive and Optimize Inhibitors of Chikungunya Virus

**DOI:** 10.1021/acsmedchemlett.5c00515

**Published:** 2025-10-24

**Authors:** Caroline M. Roach, Zachary J. Streblow, Yuting Zhang, Tyler J. Ogorek, Alejandro Ponce-Flores, Colleen B. Jonsson, Daniel N. Streblow, Jennifer E. Golden

**Affiliations:** † Division of Pharmaceutical Sciences, School of Pharmacy, 5228 University of Wisconsin-Madison, Madison, Wisconsin 53705, United States; ‡ Department of Chemistry, University of Wisconsin-Madison, Madison, Wisconsin 53706, United States; § Vaccine and Gene Therapy Institute, 6684 Oregon Health and Science University, Beaverton, Oregon 97006, United States; ∥ Regional Biocontainment Laboratory, 12326 University of Tennessee Health Science Center, Memphis, Tennessee 38163, United States; ⊥ Department of Microbiology, Immunology and Biochemistry, University of Tennessee Health Science Center, Memphis, Tennessee 38163, United States; # Division of Pathobiology and Immunology, Oregon National Primate Research Center, Beaverton, Oregon 97006, United States; ∇ Department of Pharmaceutical Sciences, College of Pharmacy, University of Tennessee Health Science Center, Memphis, Tennessee 38163, United States

**Keywords:** alphavirus, chikungunya, inhibitor, quinazolinone

## Abstract

Chikungunya virus (CHIKV) results in debilitating chronic pain in nearly half of those infected. With no FDA approved small molecule-based therapeutics available, we screened compounds to reveal quinazolinone (*S*)-**1a** with a modest 0.3 log reduction of CHIKV titer and no significant toxicity (CC_50_ > 40 μM). Five scaffold regions were surveyed to improve the titer reduction efficiency. Chemistry was established to preserve the enantiopurity of 2-piperidinyl-containing analogues, affording (*R*)-**1h** (BDGR-651) which reduced CHIKV titer in normal human dermal fibroblasts by 4.1 log at 10 μM (EC_50_ = 0.86 μM). Excellent solubility and mouse microsomal and plasma stabilities were observed, and confocal microscopy of infected Vero E6 cells treated with (*R*)-**1h** showed a dose-dependent protective effect. A narrow selectivity index prevented *in vivo* evaluation, but the study showed that antiencephalitic alphavirus quinazolinones could be reengineered to inhibit CHIKV, an arthritogenic virus, against which previous analogues showed no significant activity.

## Graphical abstract

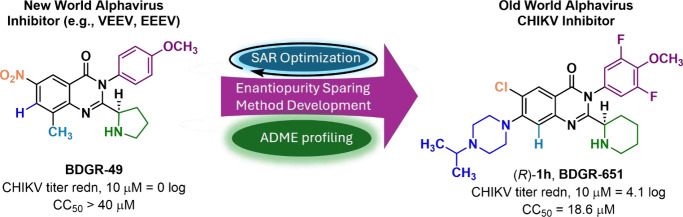

Chikungunya virus (CHIKV) is a mosquito-borne, positive-sense, single-stranded RNA alphavirus. It causes a human illness characterized by severe muscle and joint pain, fatigue, headache, nausea, and rash.^1–6^ Symptoms can resolve within weeks of the initial infection; however, at least 40% of patients suffer from debilitating arthritis pain that persists for months or years.^7,8^ CHIKV is designated as a priority pathogen due to its rapid emergence, pandemic potential, and the severity of human disease it induces.^3,5,9^ Currently, two vaccines are available. Ixchiq is a live, attenuated, single-dose vaccine that was FDA approved for those 18 years and older. It is not prescribed for those with weakened immune systems,^10^ and it carries a warning for serious adverse events in older adults (60+ yrs).^11^ Vimkunya, a recombinant single-dose vaccine, was approved in 2025 for use in people of ages 12 and older but also has limitations in pregnant women and the immune-compromised.^10^ For infected individuals, however, only supportive care is available.^12–14^ Global climate changes have enabled mosquito migration and led to the expansion of CHIKV infection beyond endemic areas of Africa and Asia.^5,15^ More than three-quarters of the world’s population is now at risk,^16^ including frequent travel spots within the Americas, Europe, and territories throughout the Caribbean Sea, as well as the Pacific and Indian oceans.^3,9^ Thus, the global distribution of CHIKV infection is likely to increase, underscoring the need for safe and effective therapeutics. Given our extensive development of inhibitors^17–20^ against encephalitic alphaviruses (e.g., VEEV, EEEV), we screened a selection of our internal compound library to identify potential CHIKV hits. While the quinazolinone BDGR-49 potently inhibited encephalitic alphaviruses,^18,19^ it showed no appreciable activity against CHIKV at 10 μM (Figure 1).

**Figure 1:**
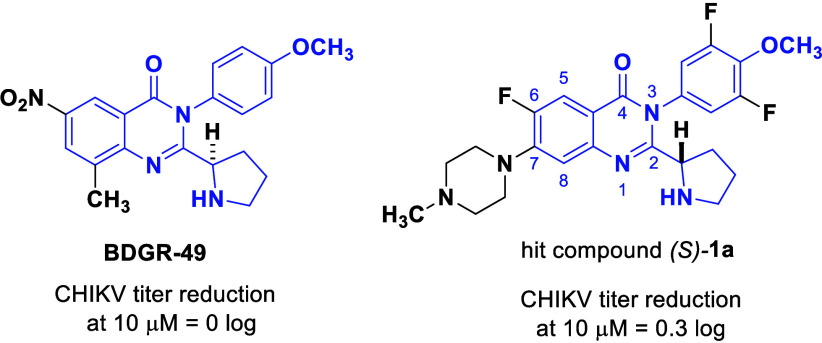
Shaded structural similarity of VEEV/EEEV inhibitor BDGR-49 and early hit CHIKV inhibitor, compound (*S*)-**1a**.

Nonetheless, we were intrigued by compounds with a differentiated substitution pattern on the quinazolinone core of BDGR-49 that showed a meager reduction of CHIKV titer at a concentration of 10 μM, exemplified by the hit compound (*S*)-**1a**. We decided to explore the distinguishing structural features of compound (*S*)-**1a** to establish a potential pharmacophore, improve potency, and investigate preliminary physiochemical and *in vitro* ADME parameters.

To assemble analogues, a variety of *N*-Boc protected, quinazolinone intermediates were generated prior to further diversification (Scheme 1). In all cases, the analogues were built from commercially available anthranilic acids **2a**–**c**. Thereafter, we often employed a one-pot, three step, copper-mediated protocol that was developed in our lab to preserve the stereochemistry of chiral amino acid derivatives used during the quinazolinone core assembly.^21^ For intermediates **4a**–**c**, an additional step using *N,O*-bis­(trimethylsilyl)­acetamide was necessary to ring-close the bis-amide precursors **3a**–**c**.^22^ For intermediates **4d**–**k**, the ring closure proceeded directly. For other N3-aryl compounds that did not form under the copper-mediated conditions (*e.g*., **4l**–**m**) or those lacking a stereocenter to be concerned with, such as **4o**–**p**, harsher conditions using triphenyl phosphite were employed. For compound **4n**, which bore an N3-methyl substituent in place of an aryl ring, *N*-methylamide **5a** was formed via coupling of the anthranilic acid,^23^ followed then by ring closure.

**Scheme 1:**
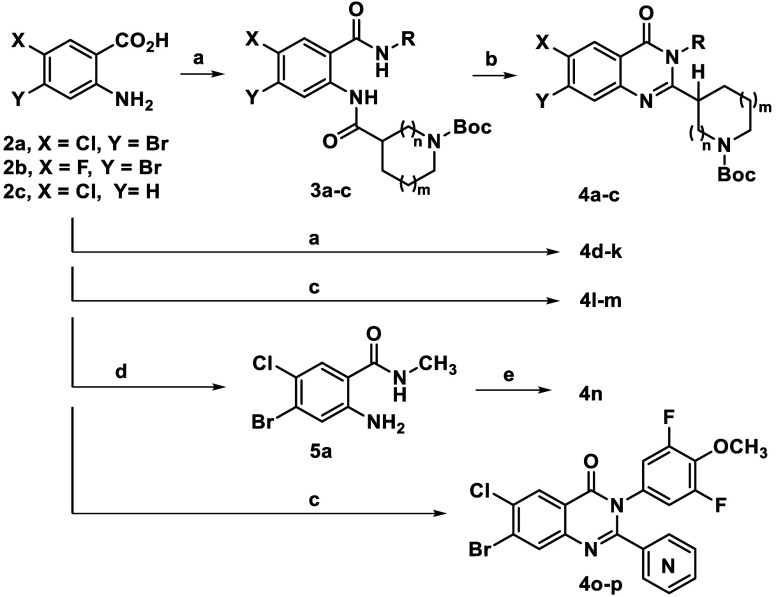
Quinazolinone Intermediate Syntheses^a^

a Reagents and conditions: (a) one pot, 3-steps: *N*-(*t*-butoxycarbonyl) amino acid, MsCl, NMI, CuCl_2_, DCE, rt, 16 h, then aniline, reflux, 24 h; then Et_3_N, TMSCl, reflux, 24 h, (19–52%, 3 steps); (b) *N,O*-bis­(trimethylsilyl)­acetamide, Et_3_N, CH_3_CN, 85 °C, 16 h, (51–79%); (c) *N*-(*t*-butoxycarbonyl) amino acid or picolinic acid or nicotinic acid, P­(OPh)_3_, pyridine, 130 °C, 1 h, then aniline, 145 °C, 8 h, (35–38%); (d) EDC·HCl, HOBT, CH_3_NH_3_Cl, DIPEA, DMF, rt, 16 h, (89%); (e) one pot, 2 steps, *N*-(*t*-butoxycarbonyl) amino acid, MsCl, NMI, CuCl_2_, DCE, rt, 16 h, then Et_3_N, TMSCl, reflux, 24 h (81–86%, 2 steps).

Quinazolinone intermediates **4a**–**g** and **4i**–**n** were subjected to Buchwald coupling conditions to afford amine substituted cores **6a**–**x** (Scheme 2, *path a*). Removal of the *N*-Boc group afforded TFA salts **7g-h, 7j, 7l-q,** and **7t-v**, which were isolated, characterized, and eventually free-based for this project to afford analogues **1g-h, 1j, 1l-q,** and **1t-v**. More recently generated intermediates were *N*-deprotected, and after implementing a basic workup, isolated as nonsalts directly to afford **1a-f, 1i, 1k, 1l-m, 1o, 1r-s,** and **1v-x**. Additional derivatives were obtained using a reductive amination^24^ or coupling procedure to install *N*-piperidinyl substituents (**1y, 1z, 1aa**). Piperidinyl analogue **1ab** was generated via *N*-deprotection of the *N*-Boc intermediate **4h** (*path b*), and pyridyl analogues **1ac** and **1ad** were obtained via Buchwald coupling of the bromide precursors **4o**–**p** (*path c*).

**Scheme 2:**
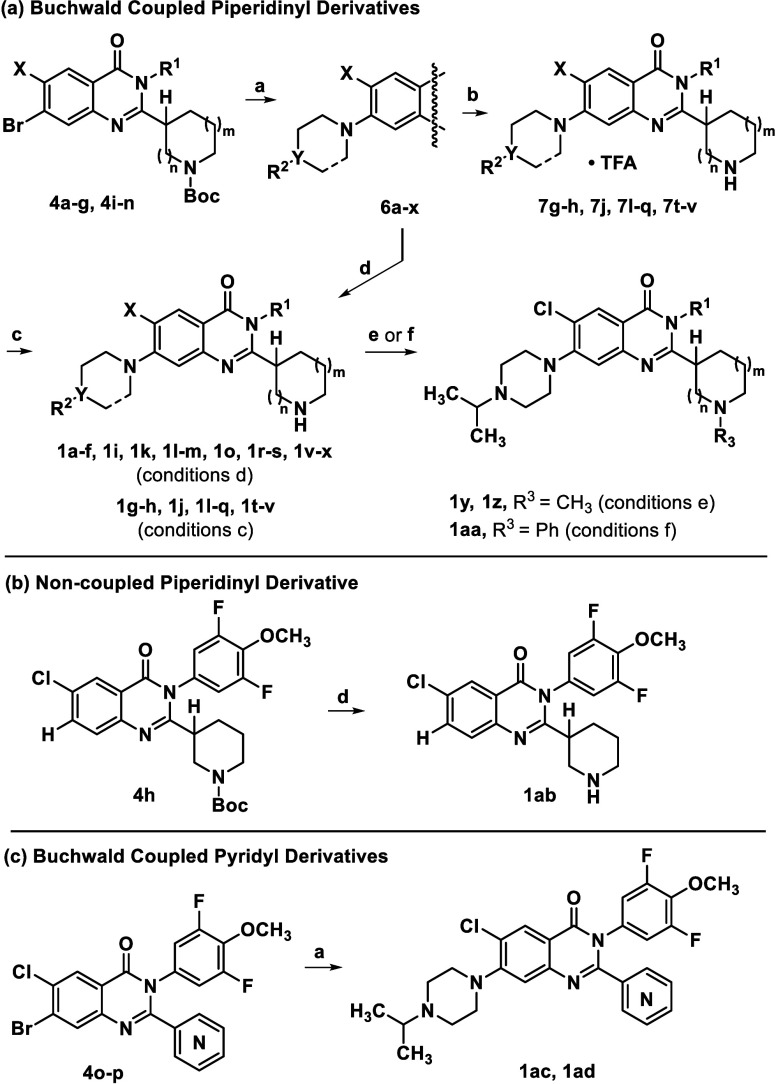
Intermediate Derivatization to Yield Analogues^a^

a Reagents and conditions: (a) amine, Pd_2_(dba)_3_, XantPhos, Cs_2_CO_3_, toluene, 100 °C, 24 h, (14–75%); (b) TFA, DCM, rt, 2 h, (53–89%); (c) K_2_CO_3_, DCM, MeOH, rt, 2 h, (32–98%); (d) TFA, DCM, rt, 2 h; then K_2_CO_3_, rt, 1 h, (42–96%); (e) 37% formaldehyde, acetic acid, NaBH_3_CN, DCM, MeOH, rt, 16 h, (49–86%); (f) bromobenzene, Pd_2_(dba)_3_, XantPhos, Cs_2_CO_3_, toluene, 100 °C, 24 h (36%).

The compounds were tested in a cell-based, antiviral titer reduction assay employing CHIKV (strain 181/25mKate) in normal human dermal fibroblast (NHDF) cells.^25,26^ Each compound was tested in triplicate at 10 μM and compared against a negative DMSO control and an unpublished positive control, SRI-42718, a CHIKV nsP4 polymerase inhibitor.^27^ Plaques were counted using a stereomicroscope at 72 h postinfection. Selected compounds were also evaluated at multiple concentrations to afford EC_50_ values. In parallel, compounds were also assessed for cytotoxicity in NHDF cells at 72 h in a dose response format at a maximum of 40 μM concentration.

Early structural modifications were made to assess stereochemical preferences and the effect of changing the *N*-substituent on the piperazine moiety. Broad structural exploration of the C6-fluorine atom was challenging due to limited commercial availability or synthetic routes leading to anthranilic acids with both a C6-substituent and neighboring coupling-friendly C7 functionality. As such, the C6 position featured either a fluorine or chlorine atom in all reported analogues. No significant preference for one enantiomer over another was observed in the log titer reduction for either the *R*- or *S*-isomer of hit **1a** (Table 1, entries 1–2); however, improved titer reduction was associated with the *R*-isomer of several analogues in the overall study, depending on the substituents distributed across the scaffold.

**Table 1:**
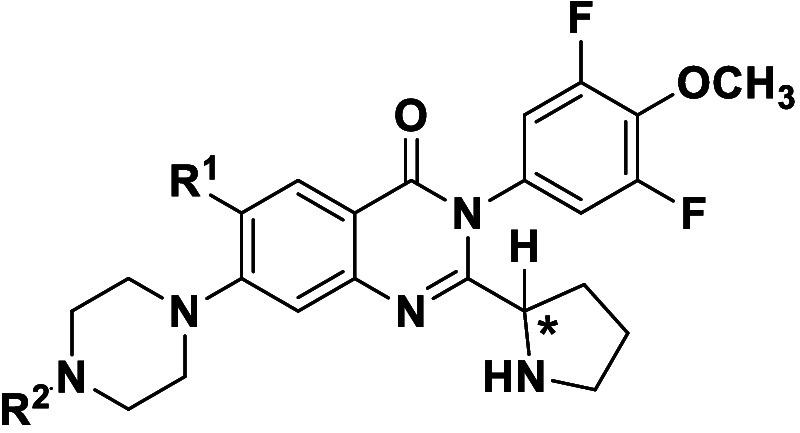
Exploratory SAR of Hit (*S*)-**1a**: Core Halide, Piperazinyl Substitution and Pyrrolidine Stereochemistry

entry	cmpd	R^1^	R^2^	titer redn, log^a^	fold reduction^b^	CC_50_ μM^c^
1	(*S*)-**1a**	F	CH_3_	0.3	2.0	>40
2	(*R*)-**1a**	F	CH_3_	0.1	1.3	>40
3	(*S*)-**1b**	F	*i*-Pr	0.64	4.6	>40
4	(*R*)-**1b**	F	*i*-Pr	1.3	20	>40
5	(*S*)-**1c**	Cl	CH_3_	1.0	10	>40
6	(*R*)-**1c**	Cl	CH_3_	0.5	3.2	>40
7	(*S*)-**1d**	Cl	*i*-Pr	1.3	20	>40
8	(*R*)-**1d**	Cl	*i*-Pr	2.3	200	19.0
9	(*S*)-**1e**	Cl	H	1.6	40	22.8
10	(*R*)-**1e**	Cl	H	2.1	126	24.9
11	SRI-42718^d^	-	-	3.7	5012	>40

a CHIKV 181/25mKate, 10 μM compound conc., 72 h, NHDF cells; averaged data, *n* ≥ 3 runs.

b Fold improvement in viral plaque reduction vs DMSO control, calculated as antilog (base 10) of the antiviral log reduction.

c NHDF cells, 72 h.

d Positive control.^27^

A 20-fold improvement in viral titer reduction compared to the control was observed by exchanging the piperazinyl *N*-methyl group for the more sterically demanding *N*-isopropyl group (Table 1, entry 4) while also revealing a preference for the (*R)*-isomer. Replacement of the C6 fluorine atom with a chlorine atom in the hit structure showed a stronger preference for the (*S*)-enantiomer (entry 5) and a more pronounced reduction in viral plaques compared to that in the fluorine-containing hit. As such, the effect of integrating the piperazinyl *N*-isopropyl substituent in tandem with a C6 chlorine atom was surveyed, resulting in the discovery of (*R*)-**1d**, which exhibited a 200-fold reduction of CHIKV titer but with some cytotoxicity noted (entry 8). The absence of a piperazinyl alkyl substituent (R^2^ = H) showed similar outcomes for selectivity and enantiomeric preferences (entries 9–10).

While enantiomeric preferences were still unclear, the early results suggested that the piperazinyl *N*-isopropyl substituent, in combination with a C6 chlorine atom, was preferred over the fluorine atom in terms of virus titer reduction. Synthetically, C6-fluorine containing intermediates would often stall at the bis-amide stage and failed to undergo efficient ring closure, thereby preventing a robust synthesis of C6-fluorine containing analogues. With this in mind, we forged ahead with the SAR studies preserving the C6 chlorine atom and *N*-isopropyl group while further investigating the role of the C2 pyrrolidine ring and its associated stereochemistry (Table 2).

**Table 2:**
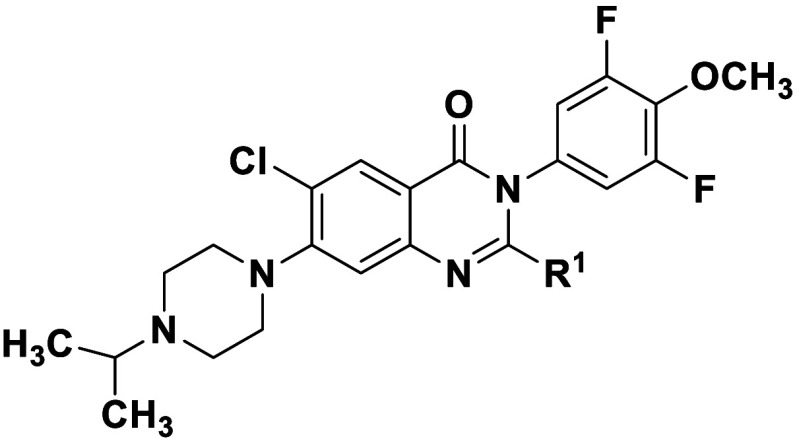

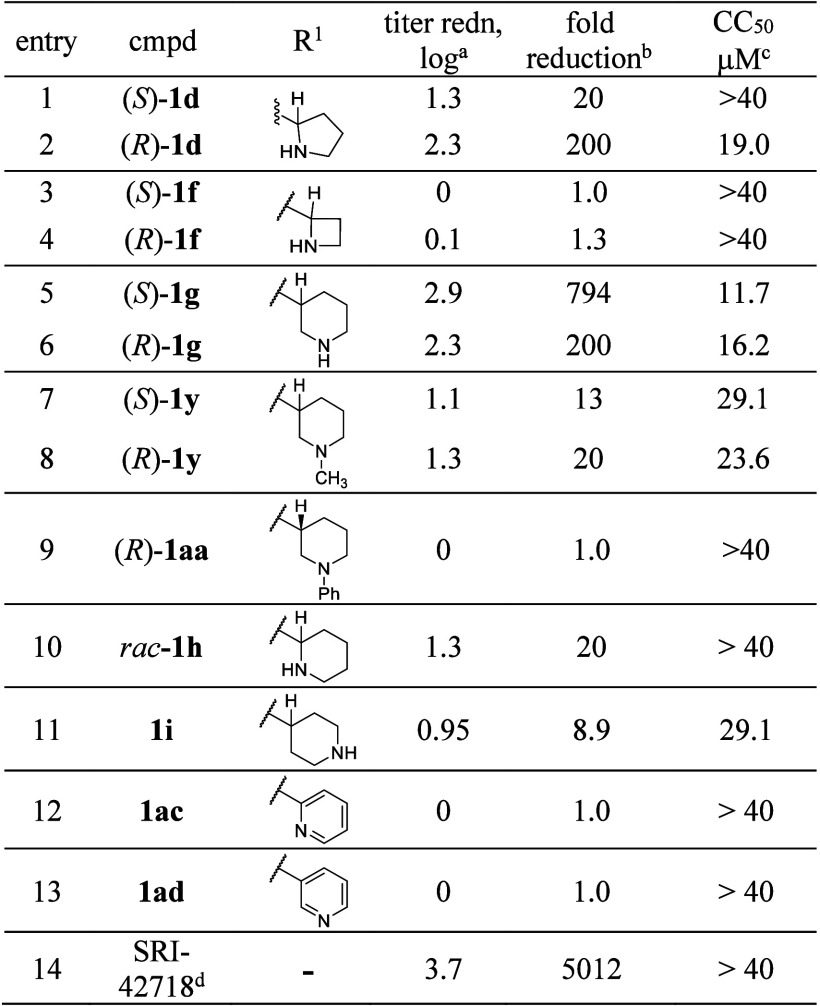
Effects of Pyrrolidine Modifications on CHIKV Titer Reduction and Cytotoxicity

a CHIKV 181/25mKate, 10 μM compound conc., 72 h, NHDF cells; averaged data, *n* ≥ 3.

b Fold improvement in viral plaque reduction vs DMSO control, calculated as antilog (base 10) of the antiviral log reduction.

c NHDF cells, 72 h.

d Positive control.^27^

The azetidine analogues (Table 2, entries 3–4) suffered from significant erosion of enantiopurity during synthesis (41–64% ee), but nonetheless, neither offered antiviral advantages over the pyrrolidines, so a variety of six-membered ring azacycles were evaluated. While the 3-piperidine analogue (*S*)-**1g** offered a ∼800-fold reduction in CHIKV titer, the increased cytotoxicity associated with this compound obscured a clear understanding of its significance. Alternatively, (*R*)-**1g** afforded a profile like that of pyrrolidine (*R*)-**1d**. Piperidinyl *N*-methyl or *N*-phenyl variants were not advantageous over other modifications (entries 7–9). The 2-piperidine analogues could not be initially synthesized effectively without epimerizing the stereocenter (<10% ee in most examples). To avoid generating a scalemic mixture, racemic **1h** was intentionally generated, which showed a 20-fold reduction in viral titer but notably without observed cytotoxicity at 40 μM (entry 10). The 4-piperidinyl **1i** and the pyridyl derivatives, **1ac-ad**, showed inferior antiviral effects.

We pursued the piperidinyl-substituted analogues given the possibility of improved antiviral activity and the idiosyncratic cytotoxicity observed with various substitution patterns (Table 3). As discussed later, we also developed a route to 2-piperidyl analogues without sacrificing the enantiopurity of intermediates with the intent of integrating learned SAR optimizations.

**Table 3:**
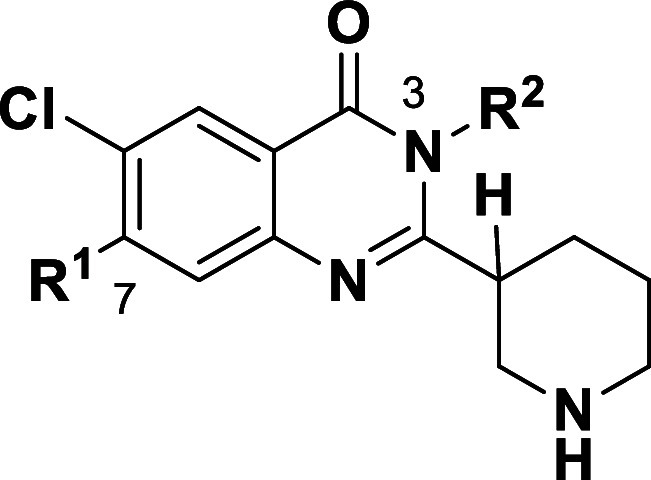

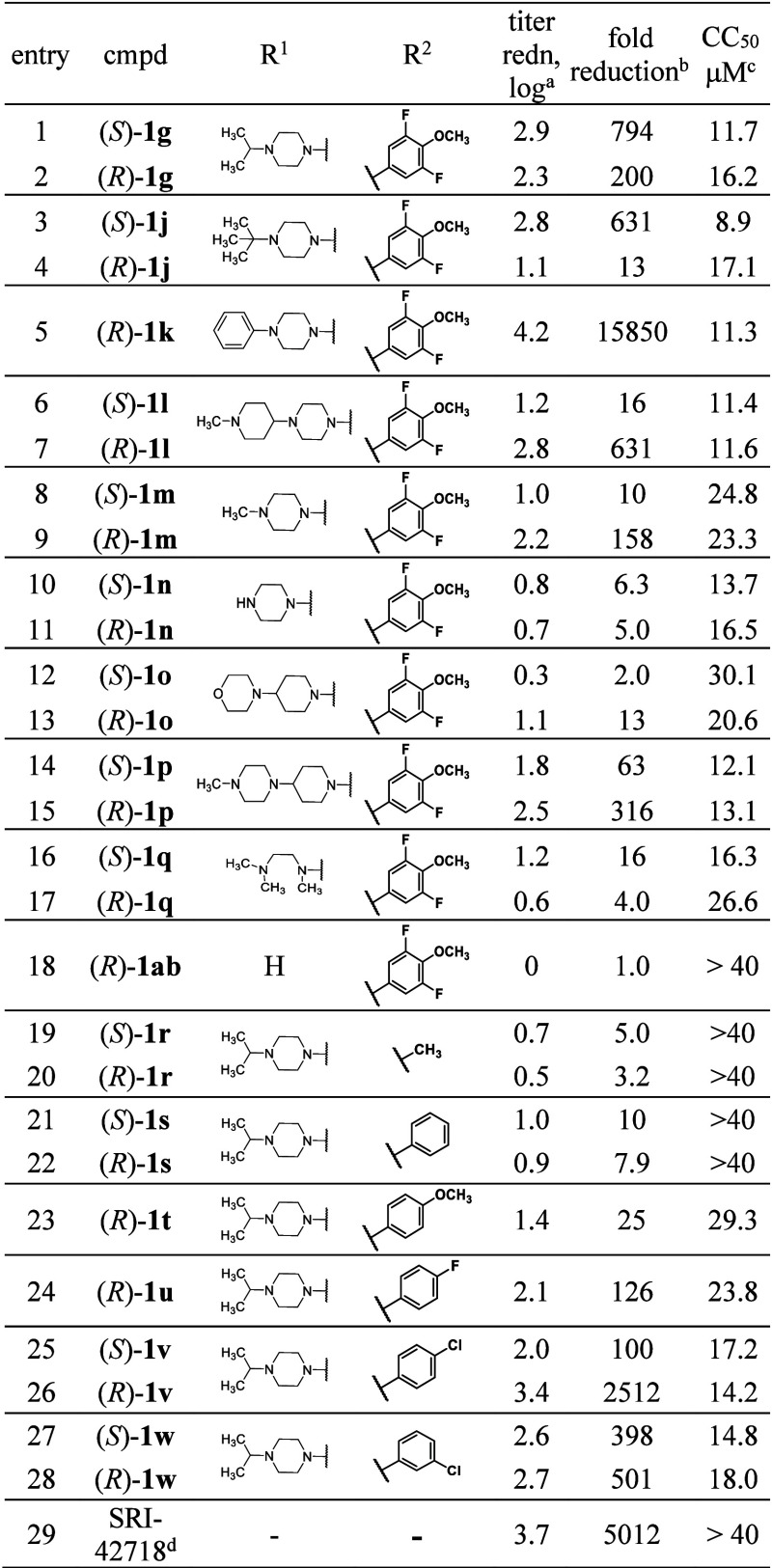
Survey of C7 Amine and N3 Substituent Modifications

a CHIKV 181/25mKate, 10 μM compound conc., 72 h, NHDF cells; averaged data, *n* ≥ 3 runs.

b Fold improvement in viral plaque reduction vs DMSO control, calculated as antilog (base 10) of the antiviral log reduction.

c NHDF cells, 72 h.

d Positive control.^27^

We selected the 3-piperidinyl analogue (*R*)-**1g** as a template to scout alterations of the C7 *N*-isopropyl piperazine (Table 3, entries 3–18). The *N-t*-butyl piperazine (*S*)-**1j** and *N*-phenyl piperazine (*R*)-**1k** showed a nearly 3- and over 4-log reduction of viral plaques, respectively, but with confounding cytotoxicity overlapping in the same concentration window, which prevented a clear interpretation of antiviral activity. Further elongation of the *N*-substituent to include an *N*-piperidine led to a similar cytotoxicity outcome. However, the smaller *N*-methyl piperazine (*R*)-**1m** showed a better selectivity profile. Replacement of the alkyl substituent with a hydrogen atom (entries 10–11) led to inferior antiviral activity while also showing cytotoxicity. Elongated piperidine derivatives (entries 12–15) and acyclic analogues (entries 16–17) also did not improve the compound profiles. Replacement of the *N*-isopropyl moiety with a hydrogen atom (entry 18) abolished all antiviral activity.

While preserving the C7 *N*-isopropyl piperazine, we also varied the N3-substituent (Table 3, entries 19–28). Switching from the difluoro­methoxyphenyl moiety to a methyl group or a simplified phenyl ring attenuated antiviral activity (entries 19–22). Halogenated phenyl rings fared better, resulting in 2- to nearly 3.5 log reductions in viral titer but still with cytotoxic effects. These structural permutations were conducted in parallel to explorations of a modified synthesis of 2-piperidinyl quinazolinone derivatives, which were racemized in our earlier protocols (see Table 2, entry 10). We were intrigued by this structural feature as it avoided cytotoxicity and was hypothesized to hold promise when paired with other structural features that reduced viral plaques. In fact, prototype *rac*-**1x** (bearing the C7 *N*-isopropyl piperazine and N3 4-chlorophenyl substituent) was determined to reduce viral titer by nearly 4 log while demonstrating favorable physiochemical and preliminary ADME characteristics, including excellent plasma and microsomal stability in mice (Table 4).

**Table 4:**
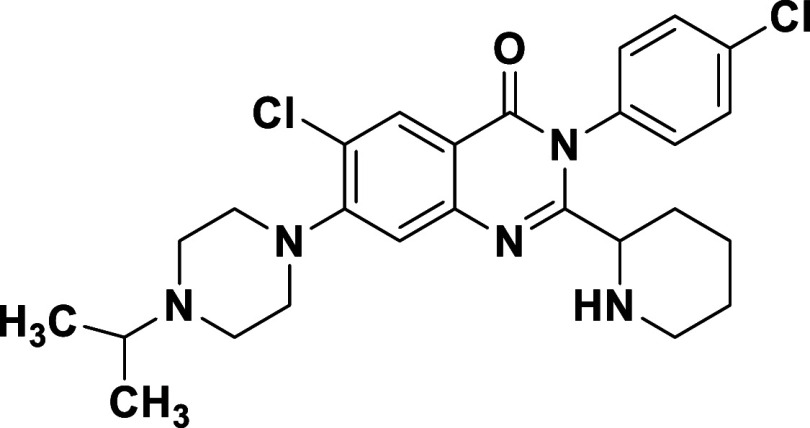
Activity and Primary ADME Profile of *rac*-**1x**

parameter	outcome
CHIKV titer reduction^a^	3.8 log (6310-fold reduction)^b^
CC_50_	20.9 μM^c^
log D_7.4_	2.3^d^
aqueous kinetic solubility	34.6 μM^d^
microsomal stability, mouse	*T*_1/2_ > 145 min^e^
plasma stability, mouse	*T*_1/2_ > 289.1 min^e^
plasma protein binding, mouse	96.7%^e^ ^,^ ^f^

a CHIKV 181/25mKate, 10 μM compound conc., 72 h, NHDF cells; averaged data, *n* > 3.

b Fold improvement in viral plaque reduction vs DMSO control, calculated as antilog (base 10) of the antiviral log reduction.

c NHDF cells, 72 h.

d PBS buffer, pH 7.4.

e CD-1 mouse.

f 2 μM protein concentration.

We employed triphenyl phosphite to assemble 2-piperidinyl containing quinazolinones, but the harsh conditions led to significant epimerization, so we opted to prepare and assess racemates of these analogues initially. While we had previously developed an excellent protocol to address epimerization for most quinazolinones, we noted that the one-pot, copper-mediated method (Scheme 1, *step a*) failed to afford the desired product when applied to these 2-piperidinyl analogues. Stepwise analysis of the copper-mediated protocol revealed that the intermediary benzoxazinone **S3** did not form, likely due to the presence of the bulky *N*-Boc-2-piperidinyl substituent (see SI, Scheme S3).

Alternatively, we decided to build the bis-amide intermediate in a stepwise fashion and evaluate condensation methods to form the quinazolinone ring while preserving the stereochemistry component appended to it (Scheme 3). After extensive screening of various strategies and conditions (SI, Tables S1–S2), we determined that HATU-facilitated formation of benzamides **5b**–**c** was preferential before integrating the more sterically demanding *N*-Boc-2-piperidinyl substituent (Scheme 3). The second amidation to form **3d**–**e** was optimally executed using POCl_3_ ^28^ due to the comparatively short reaction time over other options, ease of purification, and retention of starting material and intermediate enantiopurity. Subsequent quinazolinone ring closure was successfully completed to afford **4l**–**m** using *N,O*-bis­(trimethyl­silyl)­acetamide, thereby providing the best overall results in terms of yield and enantiopurity preservation (Scheme 3).

**Scheme 3:**
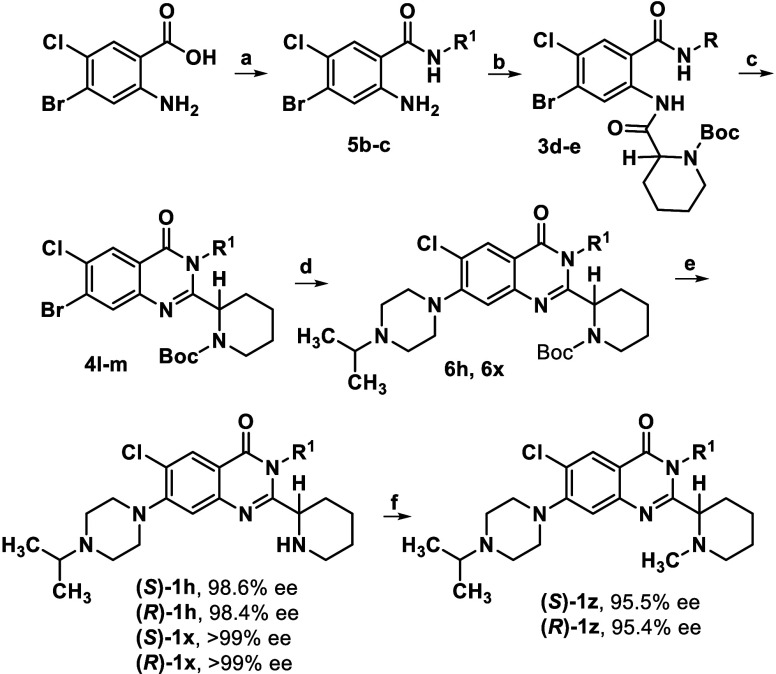
Synthetic Route to Enantioenriched 2-(Piperidin-2-yl)­quinazolinones^a^

a Reagents and conditions: (a) aniline, HATU, Et_3_N, DMF, rt, 16 h (61–90%); (b) carboxylic acid, POCl_3_, Et_3_N, DMAP, DCE, rt, 24 h (23–48%); (c) *N,O*-bis­(trimethylsilyl)­acetamide, Et_3_N, MeCN, 85 °C, 16 h (56–88%); (d) *N*-isopropylpiperazine, Pd_2_(dba)_3_, XantPhos, Cs_2_CO_3_, toluene, 100 °C, 24 h, (18–27%); (d) TFA, DCM, rt, 2 h; then K_2_CO_3_, rt, 1 h, (49–81%); (f) 37% formaldehyde, acetic acid, NaBH_3_CN, DCM, MeOH, rt, 16 h, (65%).

Buchwald coupling, followed by *N*-Boc deprotection, afforded each individual enantiomer of compounds **1h** and **1x**. The *N*-methylation of the piperidinyl ring nitrogen atom via reductive amination gave the enantiomers of **1z**. This approach, applied to the synthesis of selected analogues that incorporated key functionality at N3 and C6–7, afforded each enantiomer with >95% ee (Scheme 3). Assessment of the individual isomers of **1h**, **1x**, and **1z** (Table 5) revealed (*R*)-**1h**, known internally as BDGR-651, with the best combination of viral plaque reduction (4.1 log) and cytotoxicity (CC_50_ ∼ 19 μM). The titer reduction assay, performed in dose response format, revealed an EC_50_ = 0.86 μM in NHDF cells (Figure S1).

**Table 5:**
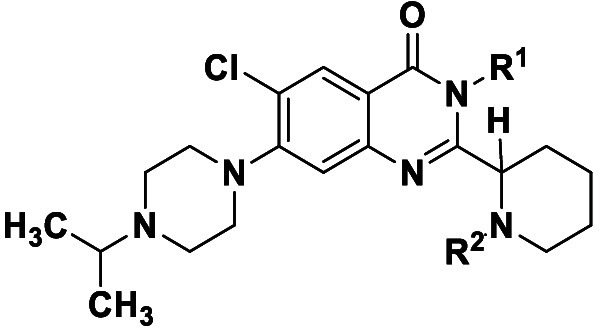

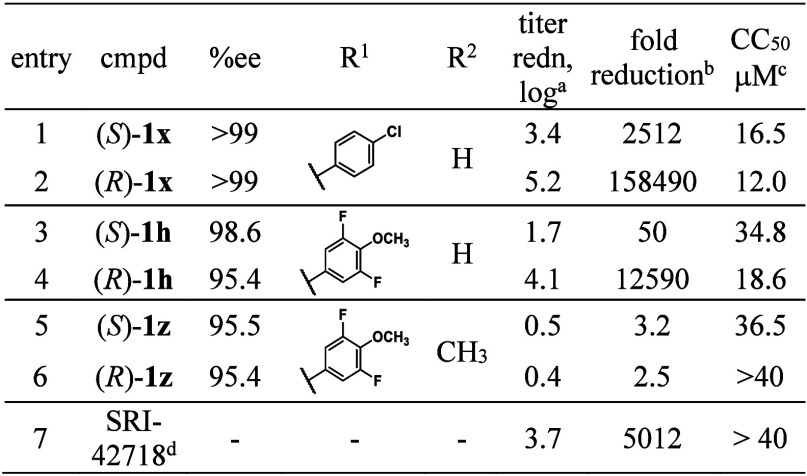
Antiviral and Cytotoxicity Data for Enantiomers of C2-Substituted 2-Piperidinyl Quinazolinone Analogues

a CHIKV 181/25mKate, 10 μM, 72 h, NHDF cells; data averaged, *n* ≥ 3 runs.

b fold improvement in viral plaque reduction vs DMSO control, calculated as antilog (base 10) of the antiviral log reduction.

c NHDF cells, 72 h exposure.

d Positive control.^27^

The effect of (*R*)-**1h** on CHIKV infection was observed microscopically using high-content confocal imaging of the viral capsid within infected Vero E6 cells (Figure 2A). Uninfected, untreated cells stained brightly with nuclear staining (mock control) and were abundant. Cells infected with CHIKV at an MOI of 1 without (*R*)-**1h** showed a reduced number of stained cells. The addition of (*R*)-**1h** in increasing concentrations to infected cells at 1, 2, 8, 16, and 20 μM resulted in a dose-dependent protective effect in which the highest concentration of (*R*)-**1h** reduced CHIKV induced cell death by the greatest degree. Quantitative assessment of the capsid protein in 20 images per well in triplicate for all concentrations revealed an EC_50_ = 6.0 μM (Figure 2B). At the highest compound concentrations in Vero E6 cells, cytotoxic effects were observed for (*R*)-**1h**, resulting in a decrease in live cell count. The CC_50_ value in this cell line was determined to be >20 μM.

**Figure 2:**
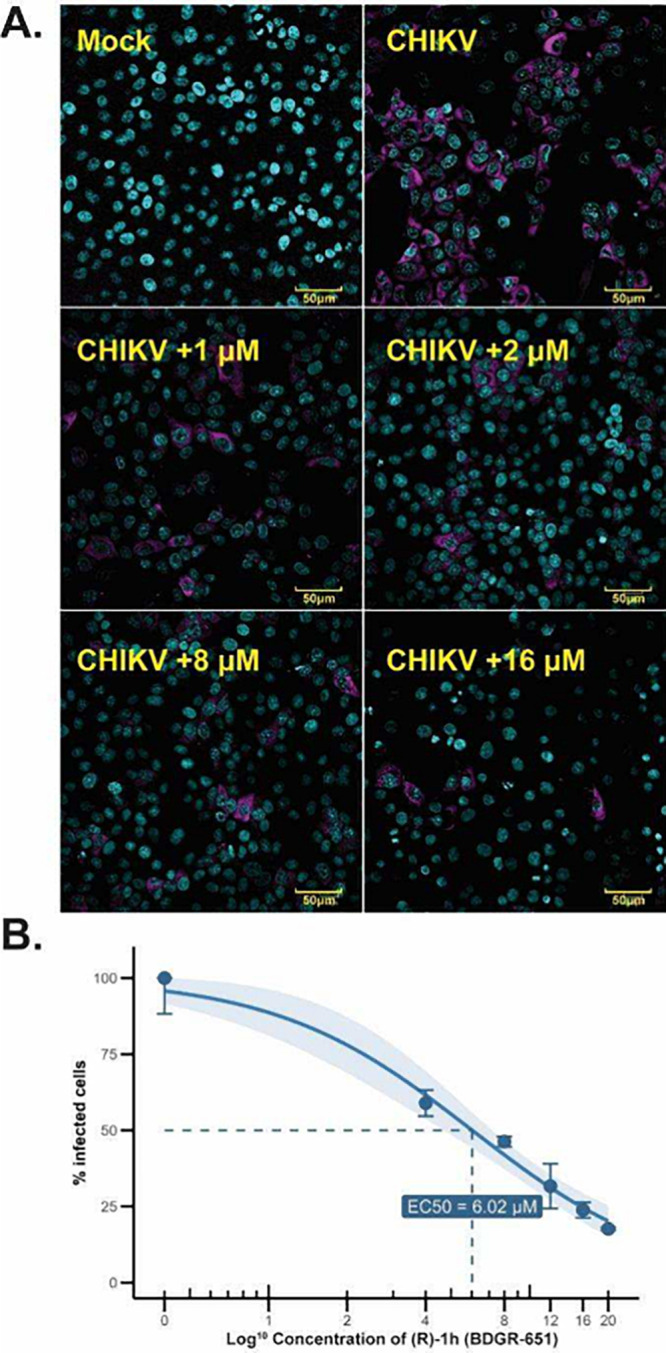
A. Selected microscopic images from high-content confocal screening. Noninfected versus CHIKV infected Vero E6 cells as a function of (*R*)-**1h** treatment. DAPI stained nuclei (blue), CHIKV capsid protein visualized with Alexa Fluor 488-conjugated antibody (pseudocolored magenta). B. Dose response curve of imaging data (3 replicates of each concentration/20 images per well).

While the selectivity index of (*R*)-**1h** limited its advancement into pharmacokinetic models, quinazolinone (*R*)-**1h** (BDGR-651) was profiled for initial physiochemical and ADME properties to characterize and benchmark these attributes as a function of structural features prior to optimizing the series further. Compared to the early prototype, *rac*-**1x** (Table 4), a 5-fold boost in aqueous solubility and a significant free fraction of unbound compound in mouse plasma was observed for (*R*)-**1h** (Table 6). Gratifyingly, excellent mouse plasma and microsomal stability was preserved. On these grounds alone, (*R*)-**1h** displayed a reasonable profile which suggests that further tuning of potency and cytotoxicity may bode well for the series in additional rounds of optimization. This will be the subject of future work involving this CHIKV-based quinazolinone series.

**Table 6:** Solubility and Early ADME Data for (*R*)-**1h**, (BDGR-651)

parameter	outcome
kinetic solubility (PBS buffer)	183 μM^a^
microsomal stability, mouse	*T*_1/2_ > 145 min^b^
plasma stability, mouse	*T*_1/2_ > 240 min^b^
plasma protein binding, mouse	76.8%^c^

a PBS buffer, pH 7.4.

b CD-1 mouse.

c 2 μM protein concentration.

Despite the extensive quinazolinone compound library built internally to combat New World encephalitic alphaviruses such as VEEV and EEEV, no significant activity was observed for selected compounds that were tested against the Old World arthritogenic alphavirus CHIKV. Nonetheless, assessment of a broader sampling of quinazolinones from our collection that were negative controls against the encephalitic alphaviruses revealed modest anti-CHIKV activity in a titer reduction assay. As a result, an optimization effort involving five regions of the quinazolinone core improved the titer reduction capability from a meager 0.3 log of hit compound (*S*)-**1a** to an analogue, (*R*)-**1h**, that reduced CHIKV plaques by 10,000-fold compared to the DMSO control while also exhibiting favorable physiochemical and tier one ADME characteristics.

Key lessons from the SAR effort included a preference for a C5 substituent combined with a fluorine or chlorine atom at C6. The presence of a substituted aryl ring on the quinazolinone core N3 position was preferred over a simple phenyl ring or methyl group, and 2-piperidines at C2 were favored over four-, five-, and other isomeric six-membered ring amines. A strong stereochemical preference regarding the C2 moiety was not apparent, although combinatorial structural changes across the scaffold appear to influence enantiomeric preferences. Compound (*R*)-**1h** featured the presence of a quinazolinone C2 2-piperidinyl moiety, the installation of which required the development of an alternative synthetic strategy to successfully form the quinazolinone while also preventing racemization of the stereochemistry of the partnered amino acid derivative.

Some degree of cytotoxicity was observed for most analogues in this series due to structural differences – contrary to that observed for the VEEV and EEEV inhibitors, and generally, the magnitude of the cytotoxicity may correlate with antiviral effects. However, some analogues showed a broader selectivity index that suggested that a more extensive SAR effort, especially focused on the N3 aryl substituent and C7 and C8 substitution, may offer opportunities for improvement. These future studies will be important to pursue resistant mutations to reveal potential involvement of viral proteins, studies that were hampered in the current work by the low selectivity index with (*R*)-**1h**, as well as assessing *in vivo* pharmacokinetic profiles and efficacy, which are premature at this time. Compared to quinazolinones like BDGR-49 that potently inhibit VEE and EEE alphaviruses, CHIKV quinazolinones contain pivotal structural differences (e.g., C6 nitro group to halide change) that are inconsistent with quinazolinone SAR that is known to be required for encephalitic alphavirus inhibition. As such, we suspect that the mechanism of action of the CHIKV quinazolinones may be different. This will be assessed and reported elsewhere. Nonetheless, this work highlights the capacity of the quinazolinone core to be structurally tuned for activity against phylogenetically related yet unique viruses such as VEEV and CHIKV which make targeting them effectively with a single compound particularly challenging. The development of chemical scaffolds that can be optimized for re-emerging viruses such as CHIKV is critical, as the effects of global warming remain significant and variably unmitigated. Further, new therapies will be needed to address the potential increased number of infections and vulnerable populations.

## Supplementary Material



## Notes

Assay and experimental details, synthetic procedures, and NMR spectra for all new final compounds reported in this work (PDF)

## Notes

Phosphorus oxychloride (POCl_3_) is corrosive, water reactive, and causes skin, eye, and lung irritation. It is toxic by inhalation or if swallowed. Use is restricted to a chemical fume hood and should be handled with suitable protective eyewear, clothing, and gloves.

## ABBREVIATIONS

ADME: absorption, distribution, metabolism, excretion
CHIKV: chikungunya virus
EEEV: eastern equine encephalitis virus
NHDF: normal human dermal fibroblasts
SAR: structure–activity relationships
VEEV: Venezuelan equine encephalitis virus
