# Supplementary material for: Restructuring Antiviral Quinazolinone Frameworks to Derive and Optimize Inhibitors of Chikungunya Virus
Source: ACS Med Chem Lett. 2025 Oct 24;16(11):2320–7. doi: 10.1021/acsmedchemlett.5c00515 (PMC12621019; doi:10.1021/acsmedchemlett.5c00515)

Restructuring Antiviral Quinazolinone Frameworks to Derive and Optimize Inhibitors of Chikungunya Virus

Caroline M. Roach[†,‡], Zachary J. Streblow[Ŧ,‡], Yuting Zhang[¥], Tyler J. Ogorek[ɣ], Alejandro Ponce-Flores[ϴ], Colleen B. Jonsson[¥,ϴ,ℇ], Daniel N. Streblow[Ŧ,¶], Jennifer E. Golden[†,ɣ,*]

[†] Division of Pharmaceutical Sciences, School of Pharmacy, University of Wisconsin-Madison, Madison, Wisconsin 53705, United States
[ɣ] Department of Chemistry, University of Wisconsin-Madison, Madison, Wisconsin 53706, United States
[Ŧ] Vaccine and Gene Therapy Institute, Oregon Health and Science University, Beaverton, Oregon, United States
[¥] Regional Biocontainment Laboratory, University of Tennessee Health Science Center, Memphis, Tennessee, United States
[ϴ] Department of Microbiology, Immunology and Biochemistry, University of Tennessee Health Science Center, Memphis, Tennessee, United States
[¶] Division of Pathobiology and Immunology, Oregon National Primate Research Center, Beaverton, Oregon, United States
[ℇ] Department of Pharmaceutical Sciences, College of Pharmacy, University of Tennessee Health Science Center, Memphis, Tennessee, United States
*corresponding author, jennifer.golden@wisc.edu

Contents

General Chemistry Considerations **S2**

Scheme S1. Quinazolinone Intermediate Syntheses **S3**

Scheme S2. Intermediate Derivatization to Yield Analogs **S21**

Scheme S3. Stepwise Formation of Quinazolinone Intermediates **S81**

Scheme S4. Synthetic Route to 2-Piperidin-2-yl Quinazolinone Intermediates **S82**

Table S1. Methods Screened to Yield 2-Piperidin-2-yl Quinazolinones **S83**

Scheme S5. Coupling Method Using $POCl_3$ **S84**

Table S2. Conditions for Optimization of $POCl_3$ Coupling Step **S85**

Scheme S6. Synthetic Route to Enantioenriched 2-(Piperidin-2-yl) Quinazolinones **S86**

LCMS and Chiral HPLC Chromatograms for (*R*)-**1h** **S99**

Assay Protocols **S102**

- CHIKV Antiviral Assay (3 point, 10-fold dilution series) S102
- Cytotoxicity Assay (10 point, 2-fold dilution series) S102
- Log D Determination S102
- Kinetic Solubility Determination S103
- Liver Microsomal Stability S103
- Mouse Plasma Stability S103
- Plasma Protein Binding S103
- High-Content Microscopy Anti-CHIKV Screen S103

Figure S1. CHIKV Titer Reduction Assay Dose Response Curve for (*R*)-**1h** **S105**

References **S105**

^{1}H, ^{13}C, ^{19}F NMR Spectra for Selected Intermediates and New Final Compounds **S107**

General Chemistry Considerations

All final compounds were determined to be of > 95% purity by UV-LCMS at 254 nm. Commercial reagents from commercial suppliers were used as received unless otherwise indicated. Caution: Phosphorus oxychloride ($POCl_3$) is corrosive, water reactive, and causes skin, eye, and lung irritation. It is toxic by inhalation or if swallowed. Use is restricted to a chemical fume hood and should be handled with suitable protective eyewear, clothing, and gloves. Solvents were used as received from commercial suppliers or from the UW-Madison School of Pharmacy Medicinal Chemistry Center dry solvent system. Reactions run in anhydrous conditions were conducted in flasks that had been oven dried at 205 °C and cooled under a dry nitrogen atmosphere. Reactions run under microwave irradiation were carried out in sealed tubes using a Biotage Initiator+ Fourth Generation Microwave Synthesizer. Flash column chromatography was conducted using a Teledyne Isco CombiFlash Rf-150 or a Teledyne Isco CombiFlash NextGen 100. Analytical TLC experiments were performed on aluminum-backed Silica Gel plates (TLC Silica gel 60 F254) from EMD Millipore and analyzed with 254 nm UV light using diluted samples. LC-MS data were obtained using an Agilent 1290 Infinity II HPLC with an Agilent 6120 Quadrupole Mass Spectrometer or via using a Shimadzu SIL-40 XR UHPLC with a Shimadzu LCMS-2020 Quadrupole Mass Spectrometer. The Agilent analytical chromatography method utilized the following parameters: Poroshell 120 EC-C18, 1.9 µm column, UV detection wavelength = 254 nm, Flow rate = 1.0 mL/min, Gradient = 5-100% LC-MS grade methanol over 4 min; the organic mobile phase and aqueous mobile phase contained 0.1% LC-MS grade formic acid. The Agilent mass spectrometer utilized the following parameters: an Agilent multimode source that simultaneously acquires ESI+/APCI+. The Shimadzu analytical chromatography method utilized the following parameters: Shim-pack Velox SP-C18, 2.7 µm column, UV detection wavelength = 254 nm, Flow rate = 1.0 mL/min, Gradient = 20-100% LC-MS grade methanol over 3.2 min; the organic mobile phase and aqueous mobile phase contained 0.1% LC-MS grade formic acid. The Shimadzu mass spectrometer utilized the following parameters: a scanning multimode source that simultaneously acquires ESI+ and ESI-. High resolution mass spectra (HRMS) were performed by an Analytical Instrument Center at the School of Pharmacy on an Electron Spray Injection (ESI) mass spectrometer. NMR spectra were obtained on either a Bruker 400 MHz or Varian 500 MHz spectrometer with compounds dissolved in chloroform-d, methanol-d_4, acetone-d_6, dimethyl sulfoxide-d_6, or *N,N*-dimethylformamide-d_7. Chemical Shifts are given in parts per million (ppm), coupling constants (J) are given in Hz, and splitting is abbreviated as follows: s (singlet), d (doublet), t (triplet), q (quartet), p (pentet), dd (doublet of doublets), dt = doublet of triplet, td = triplet of doublet, tt = triplet of triplet, ddd (doublet of doublet of doublets), m (multiplet). Enantiopurity determinations were performed on an Agilent 1200-series HPLC, using either Regis Pack, 25 cm x 4.6 mm, 5 micron column, (*R, R*)-Whelk O-1, 5 micron column, Reflect I-Amylose A, 5 micron column, or Reflect I-Cellulose B, 5 micron column.

Scheme S1. Quinazolinone Intermediate Syntheses

(Reproduced from Scheme 1 in main manuscript)

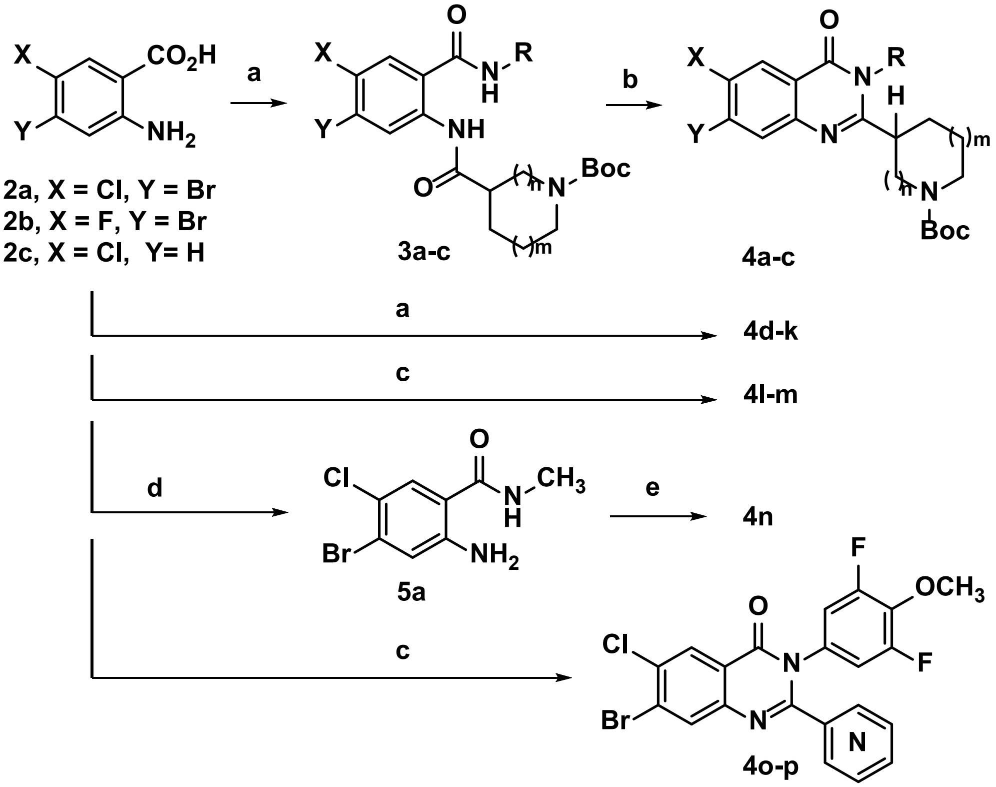

Protocol A (refer to Scheme S1)[1]

Copper chloride and Boc-amino acid derivative were added to an oven dried flask that was cooled under N_2, then anhydrous dichloroethane was added to the flask. The mixture was cooled to 0 °C using an ice-water bath and allowed to stir for 10 min until *N*-methylimidazole was added dropwise over 2 min. Methanesulfonyl chloride was added dropwise over 2 min, the ice bath was removed, and the reaction was allowed to reach rt over 2 h. The anthranilic acid was added, the reaction mixture was purged with N_2, and after stirring at rt for 16 h, the aniline was added. The resulting mixture was heated to 85 °C while stirring for 24 h. The reaction mixture was removed from heat and allowed to cool to rt. Triethylamine was added over 2 min, and then trimethylsilyl chloride was added over 2 min. The reaction mixture heated for 24 h at 85 °C. After cooling to rt, the mixture was diluted with dichloromethane (200 mL) and washed sequentially with 1 N HCl (150 mL), sat. sodium bicarbonate solution (150 mL) and sat. brine solution (150 mL). The organic layer was dried over anhydrous sodium sulfate, filtered, and the solvent was evaporated under reduced pressure. The residue was purified by flash chromatography (0% ethyl acetate / hexane ~ 50% ethyl acetate / hexane) to afford quinazolinone or diamide.

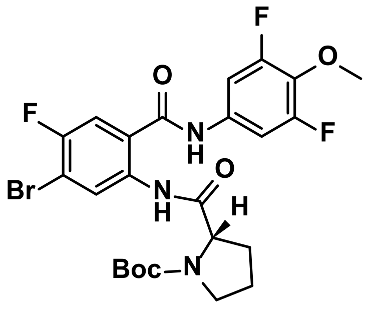

(*S*)-3a

***tert*-butyl (*S*)-2-((5-bromo-2-((3,5-difluoro-4-methoxyphenyl)carbamoyl)-4-fluorophenyl)carbamoyl)pyrrolidine-1-carboxylate**

(*S*)-3a (0.25 g, 43.6%) was prepared from $CuCl_2$ (0.034 g, 0.25 mmol), Boc-*L*-proline (0.32 g, 1.47 mmol), dichloroethane (10 mL), *N*-methylimidazole (0.27 mL, 3.40 mmol), methanesulfonyl chloride (0.11 mL, 1.47 mmol), 2-amino-4-bromo-5-fluorobenzoic acid (0.23 g, 1.00 mmol), 3,5-difluoro-4-methoxyaniline (0.32 g, 2.00 mmol), triethylamine (1.21 mL, 8.70 mmol), and trimethylsilyl chloride (0.86 mL, 6.80 mmol) in the same manner described for **protocol A**. Brown solid. *N*-methylimidazole. impurity observed in NMR, ^{1}H NMR (400 MHz, $CDCl_3$) δ 11.62 (s, 1H), 9.40 (s, 1H), 8.66 (d, *J* = 6.6 Hz, 1H), 7.66 (d, *J* = 9.2 Hz, 1H), 7.40 – 7.27 (m, 2H), 6.84 (impurity), 6.82 (impurity), 4.22 (dd, *J* = 8.5, 2.7 Hz, 1H), 3.99 (s, 3H), 3.69 (ddd, *J* = 11.3, 7.6, 4.2 Hz, 1H), 3.50 (q, *J* = 8.1 Hz, 1H), 2.29 – 1.88 (m, 4H), 1.58 (d, *J* = 51.6 Hz, 9H). ^{13}C NMR (101 MHz, $CDCl_3$) δ 171.2, 165.9, 155.5, 154.4, 136.7, 132.8, 125.3, 118.7, 114.9, 114.8, 114.5, 105.7, 105.5, 105.4, 105.2, 81.1, 62.2, 61.9, 47.4, 39.7, 30.8, 28.7, 24.4.

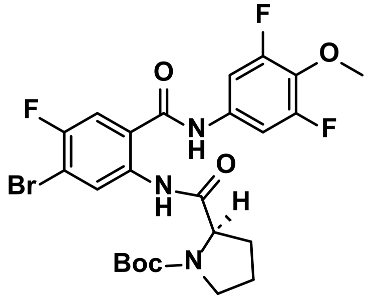

(*R*)-3a

***tert*-butyl (*R*)-2-((5-bromo-2-((3,5-difluoro-4-methoxyphenyl)carbamoyl)-4-fluorophenyl)carbamoyl)pyrrolidine-1-carboxylate**

(*R*)-3a (0.30 g, 51.5%) was prepared from $CuCl_2$ (0.034 g, 0.25 mmol), Boc-*D*-proline (0.32 g, 1.47 mmol), dichloroethane (10 mL), *N*-methylimidazole (0.27 mL, 3.40 mmol), methanesulfonyl chloride (0.11 mL, 1.47 mmol), 2-amino-4-bromo-5-fluorobenzoic acid (0.23 g, 1.00 mmol), 3,5-difluoro-4-methoxyaniline (0.32 g, 2.00 mmol), triethylamine (1.21 mL, 8.70 mmol), and trimethylsilyl chloride (0.86 mL, 6.80 mmol) in the same manner described for **protocol A**. Light brown solid. *N*-methylimidazole impurity observed in NMR ^{1}H NMR (400 MHz, $CDCl_3$) δ 11.61 (s, 1H), 9.37 (s, 1H), 8.66 (d, *J* = 6.5 Hz, 1H), 7.65 (d, *J* = 9.2 Hz, 1H), 7.38 – 7.27 (m, 2H), 6.84 (impurity), 6.82 (impurity), 4.23 (dd, *J* = 8.4, 2.7 Hz, 1H), 3.99 (s, 3H), 3.69 (ddd, *J* = 11.9, 7.6,

4.3 Hz, 1H), 3.61 – 3.44 (m, 1H), 2.27 – 1.87 (m, 4H), 1.58 (d, J = 56.9 Hz, 9H). ^{13}C NMR (101 MHz, $CDCl_3$) δ 171.2, 165.9, 157.5, 157.4, 156.9, 155.5, 155.0, 154.4, 153.2, 136.6, 132.8, 131.8, 125.3, 118.8, 114.8, 114.5, 105.7, 105.5, 105.4, 105.2, 81.1, 62.2, 61.9, 47.4, 39.7, 30.8, 28.7, 24.4.

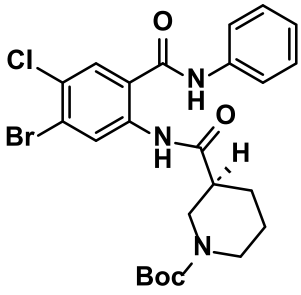

(*S*)-3b

***tert*-butyl (*S*)-3-((5-bromo-4-chloro-2-(phenylcarbamoyl)phenyl)carbamoyl)piperidine-1-carboxylate**

(*S*)-3b (0.33 g crude) was prepared from $CuCl_2$ (0.034 g, 0.25 mmol), *S*-Boc-nipecotic acid (0.337 g, 1.47 mmol), dichloroethane (10 mL), *N*-methylimidazole (0.27 mL, 3.40 mmol), methanesulfonyl chloride (0.11 mL, 1.47 mmol), 2-amino-4-bromo-5-chlorobenzoic acid (0.25 g, 1.00 mmol), aniline (0.18 mL, 2.00 mmol), triethylamine (1.21 mL, 8.70 mmol), and trimethylsilyl chloride (0.86 mL, 6.80 mmol) in the same manner described for **protocol A**. Brown solid. Carried forward without further purification or analysis.

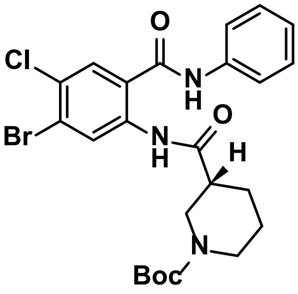

(*R*)-3b

***tert*-butyl (*R*)-3-((5-bromo-4-chloro-2-(phenylcarbamoyl)phenyl)carbamoyl)piperidine-1-carboxylate**

(*R*)-3b (0.22 g crude) was prepared from $CuCl_2$ (0.034 g, 0.25 mmol), *R*-Boc-nipecotic acid (0.34 g, 1.47 mmol), dichloroethane (10 mL), *N*-methylimidazole (0.27 mL, 3.40 mmol), methanesulfonyl chloride (0.11 mL, 1.47 mmol), 2-amino-4-bromo-5-chlorobenzoic acid (0.25 g, 1.00 mmol), aniline (0.18 mL, 2.00 mmol), triethylamine (1.21 mL, 8.70 mmol), and trimethylsilyl chloride (0.86 mL, 6.80 mmol) in the same manner described for **protocol A**. Brown solid. Carried forward without further purification or analysis.

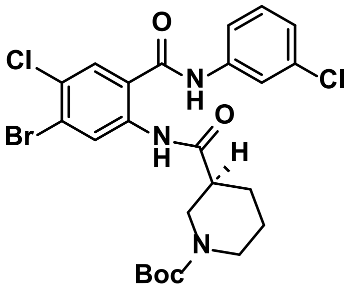

(*S*)-3c

tert-butyl (*S*)-3-((5-bromo-4-chloro-2-((3-chlorophenyl)carbamoyl)phenyl)carbamoyl)piperidine-1-carboxylate

(*S*)-3c (0.35 g crude) was prepared from $CuCl_2$ (0.034 g, 0.25 mmol), *S*-Boc-nipecotic acid (0.34 g, 1.47 mmol), dichloroethane (10 mL), *N*-methylimidazole (0.27 mL, 3.40 mmol), methanesulfonyl chloride (0.11 mL, 1.47 mmol), 2-amino-4-bromo-5-chlorobenzoic acid (0.25 g, 1.00 mmol), 3-chloroaniline (0.21 mL, 2.00 mmol), triethylamine (1.21 mL, 8.70 mmol), and trimethylsilyl chloride (0.86 mL, 6.80 mmol) in the same manner described for **protocol A**. Brown solid. Carried forward without further purification or analysis.

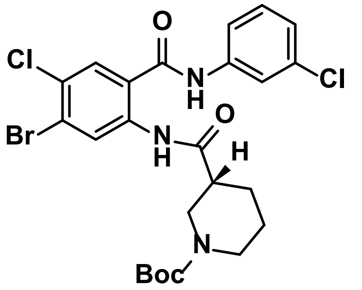

(*R*)-3c

tert-butyl (*R*)-3-((5-bromo-4-chloro-2-((3-chlorophenyl)carbamoyl)phenyl)carbamoyl)piperidine-1-carboxylate

(*R*)-3c (0.26 g crude) was prepared from $CuCl_2$ (0.034 g, 0.25 mmol), *R*-Boc-nipecotic acid (0.34 g, 1.47 mmol), dichloroethane (10 mL), *N*-methylimidazole (0.27 mL, 3.40 mmol), methanesulfonyl chloride (0.11 mL, 1.47 mmol), 2-amino-4-bromo-5-chlorobenzoic acid (0.25 g, 1.00 mmol), 3-chloroaniline (0.21 mL, 2.00 mmol), triethylamine (1.21 mL, 8.70 mmol), and trimethylsilyl chloride (0.86 mL, 6.80 mmol) in the same manner described for **protocol A**. Brown solid. Carried forward without further purification or analysis.

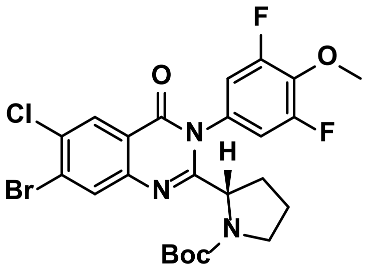

(*S*)-4d

***tert*-butyl (*S*)-2-(7-bromo-6-chloro-3-(3,5-difluoro-4-methoxyphenyl)-4-oxo-3,4-dihydroquinazolin-2-yl)pyrrolidine-1-carboxylate**

(*S*)-4d (0.33 g, 19.2%) was prepared from $CuCl_2$ (0.10 g, 0.75 mmol), Boc-*L*-proline (0.95 g, 4.41 mmol), dichloromethane (20 mL), *N*-methylimidazole (0.81 mL, 10.19 mmol), methanesulfonyl chloride (0.34 mL, 4.41 mmol), 2-amino-4-bromo-5-chlorobenzoic acid (0.75 g, 3.00 mmol), 3,5-difluoro-4-methoxyaniline (0.96 g, 6.00 mmol), triethylamine (3.64 mL, 26.10 mmol), and trimethylsilyl chloride (2.59 mL, 20.40 mmol) in the same manner described for **protocol A**. Brown solid. *N*-methylimidazole impurity observed in NMR. ^{1}H NMR (400 MHz, $CDCl_3$) δ 8.24 (d, *J* = 12.0 Hz, 1H), 7.98 (d, *J* = 21.7 Hz, 1H), 7.06 (ddt, *J* = 125.6, 10.4, 2.3 Hz, 1H), 6.82 (ddt, *J* = 12.5, 10.3, 2.2 Hz, 1H), 6.20 (impurity), 6.17 (impurity), 4.46 (ddd, *J* = 15.0, 7.9, 3.9 Hz, 1H), 4.10 (dt, *J* = 11.2, 1.3 Hz, 3H), 3.71 (dddd, *J* = 38.1, 10.2, 7.5, 5.3 Hz, 2H), 3.49 (ddt, *J* = 29.3, 10.0, 6.6 Hz, 1H), 2.17 – 1.78 (m, 3H), 1.35 (d, *J* = 51.9 Hz, 9H). ^{13}C NMR (101 MHz, $CDCl_3$) δ 161.0, 160.9, 159.4, 159.1, 158.1, 158.0, 157.4, 157.3, 157.1, 157.1, 157.0, 156.9, 155.6, 155.6, 154.9, 154.8, 154.7, 154.6, 154.6, 154.5, 154.4, 153.6, 146.6, 146.3, 142.6, 138.1, 137.9, 133.5, 133.0, 132.9, 132.7, 130.3, 130.2, 130.1, 129.7, 129.6, 127.9, 127.8, 120.9, 120.5, 114.3, 114.3, 114.1, 114.1, 114.0, 114.0, 113.8, 113.8, 113.6, 113.6, 113.5, 113.5, 113.4, 113.4, 113.3, 113.2, 99.0, 98.9, 98.8, 98.7, 80.1, 79.9, 62.4, 62.0, 62.0, 58.4, 58.3, 47.3, 47.3, 33.0, 31.8, 28.7, 28.6, 24.1, 23.0.

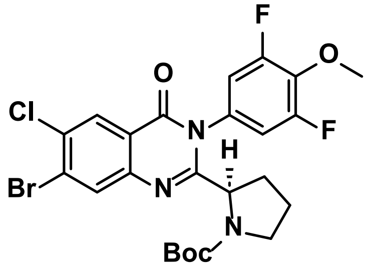

(*R*)-4d

***tert*-butyl (*R*)-2-(7-bromo-6-chloro-3-(3,5-difluoro-4-methoxyphenyl)-4-oxo-3,4-dihydroquinazolin-2-yl)pyrrolidine-1-carboxylate**

(*R*)-4d (0.35 g, 20.4%) was prepared from $CuCl_2$ (0.10 g, 0.75 mmol), Boc-*D*-proline (0.95 g, 4.41 mmol), dichloromethane (20 mL), *N*-methylimidazole (0.81 mL, 10.19 mmol), methanesulfonyl chloride (0.34 mL, 4.41 mmol), 2-amino-4-bromo-5-chlorobenzoic acid (0.75 g, 3.00 mmol), 3,5-difluoro-4-methoxyaniline (0.96 g, 6.00 mmol), triethylamine (3.64 mL, 26.10 mmol), and

trimethylsilyl chloride (2.59 mL, 20.40 mmol) in the same manner described for **protocol A**. Brown solid. *N*-methylimidazole impurity observed in NMR. ^{1}H NMR (400 MHz, $CDCl_3$) δ 8.24 (d, *J* = 12.1 Hz, 1H), 7.98 (d, *J* = 21.9 Hz, 1H), 7.06 (ddt, *J* = 125.7, 10.4, 2.2 Hz, 1H), 6.83 (ddt, *J* = 12.5, 10.3, 2.2 Hz, 1H), 6.19 (impurity), 6.17 (impurity), 4.46 (ddd, *J* = 15.1, 7.9, 3.9 Hz, 1H), 4.10 (dt, *J* = 11.2, 1.3 Hz, 3H), 3.71 (dddd, *J* = 38.0, 10.1, 7.5, 5.3 Hz, 1H), 3.49 (ddt, *J* = 29.2, 10.1, 6.6 Hz, 1H), 2.18 – 1.79 (m, 3H), 1.35 (d, *J* = 52.0 Hz, 9H). ^{13}C NMR (101 MHz, $CDCl_3$) δ 161.0, 160.9, 159.4, 159.1, 157.4, 157.3, 157.2, 157.1, 157.1, 157.0, 156.9, 154.9, 154.8, 154.7, 154.6, 154.6, 154.5, 154.4, 153.6, 146.6, 146.3, 138.2, 138.1, 137.9, 133.5, 133.0, 132.9, 132.7, 130.3, 130.2, 130.1, 129.8, 129.7, 129.6, 127.9, 127.8, 120.9, 120.5, 114.3, 114.3, 114.1, 114.1, 114.0, 114.0, 113.8, 113.8, 113.6, 113.6, 113.5, 113.4, 113.4, 113.4, 113.3, 113.2, 99.0, 98.7, 80.1, 79.9, 62.0, 62.0, 58.4, 58.3, 47.3, 47.3, 33.0, 31.8, 28.7, 28.6, 24.1, 23.0.

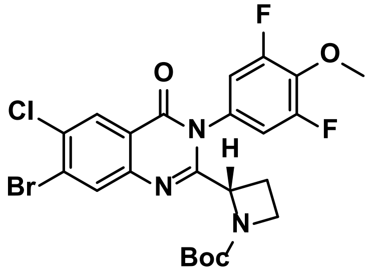

(*S*)-4e

***tert*-butyl (*S*)-2-(7-bromo-6-chloro-3-(3,5-difluoro-4-methoxyphenyl)-4-oxo-3,4-dihydroquinazolin-2-yl)azetidine-1-carboxylate**

(*S*)-4e (0.18 g, 32.7%) was prepared from $CuCl_2$ (0.034 g, 0.25 mmol), 1-Boc-*L*-azetidine-2-carboxylic acid (0.30 g, 1.47 mmol), dichloroethane (10 mL), *N*-methylimidazole (0.27 mL, 3.40 mmol), methanesulfonyl chloride (0.11 mL, 1.47 mmol), 2-amino-4-bromo-5-chlorobenzoic acid (0.25 g, 1.00 mmol), 3,5-difluoro-4-methoxyaniline (0.32 g, 2.00 mmol), triethylamine (1.21 mL, 8.70 mmol), and trimethylsilyl chloride (0.86 mL, 6.80 mmol) in the same manner described for **protocol A**. Brown solid. ^{1}H NMR (400 MHz, $CDCl_3$) δ 8.29 (d, *J* = 2.6 Hz, 1H), 8.10 (s, 1H), 7.12 – 6.89 (m, 1H), 6.73 (dt, *J* = 10.3, 2.2 Hz, 1H), 4.76 (dd, *J* = 8.5, 5.1 Hz, 1H), 4.22 – 4.09 (m, 4H), 3.87 (td, *J* = 8.4, 5.5 Hz, 1H), 2.41 – 2.28 (m, 1H), 2.27 – 2.14 (m, 1H), 1.51 – 1.29 (m, 9H). ^{13}C NMR (101 MHz, $CDCl_3$) δ 160.7, 157.3, 157.2, 157.0, 157.0, 156.4, 154.8, 154.7, 154.6, 154.5, 146.4, 138.2, 138.1, 138.0, 133.6, 133.0, 129.5, 129.4, 129.3, 127.9, 120.8, 113.2, 113.2, 113.0, 112.9, 80.2, 62.0, 28.5, 23.3.

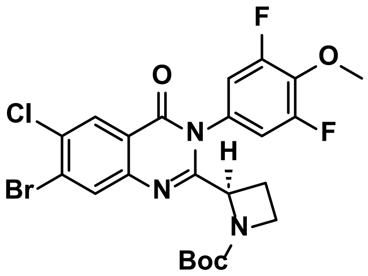

(*R*)-4e

***tert*-butyl (*R*)-2-(7-bromo-6-chloro-3-(3,5-difluoro-4-methoxyphenyl)-4-oxo-3,4-dihydroquinazolin-2-yl)azetidine-1-carboxylate**

(*R*)-4e (0.14 g, 24.3%) was prepared from $CuCl_2$ (0.034 g, 0.25 mmol), 1-Boc-*D*-azetidine-2-carboxylic acid (0.30 g, 1.47 mmol), dichloroethane (10 mL), *N*-methylimidazole (0.27 mL, 3.40 mmol), methanesulfonyl chloride (0.11 mL, 1.47 mmol), 2-amino-4-bromo-5-chlorobenzoic acid (0.25 g, 1.00 mmol), 3,5-difluoro-4-methoxyaniline (0.32 g, 2.00 mmol), triethylamine (1.21 mL, 8.70 mmol), and trimethylsilyl chloride (0.86 mL, 6.80 mmol) in the same manner described for **protocol A**. Brown solid. *N*-methylimidazole impurity observed in NMR ^{1}H NMR (400 MHz, $CDCl_3$) δ 8.28 (s, 1H), 8.08 (s, 1H), 7.11 – 6.88 (m, 1H), 6.71 (dt, *J* = 10.3, 2.2 Hz, 1H), 6.20 (impurity), 6.17 (impurity), 4.75 (dd, *J* = 8.5, 5.1 Hz, 1H), 4.10 (d, *J* = 1.5 Hz, 4H), 3.93 (s, 1H), 2.37 – 2.25 (m, 1H), 2.19 (tt, *J* = 10.4, 5.3 Hz, 1H), 1.44 – 1.27 (m, 9H). ^{13}C NMR (101 MHz, $CDCl_3$) δ 169.8, 160.7, 157.3, 157.2, 157.0, 156.5, 154.8, 154.7, 146.4, 138.3, 138.1, 138.0, 133.7, 133.0, 129.6, 129.4, 129.3, 128.0, 120.9, 113.2, 113.2, 113.0, 112.9, 104.1, 103.9, 99.0, 98.7, 81.8, 80.3, 62.4, 62.0, 28.5, 23.3.

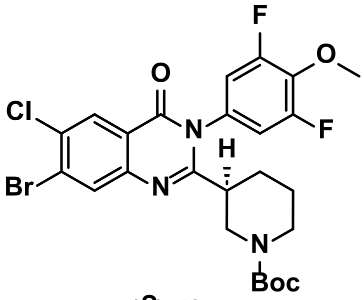

(*S*)-4f

***tert*-butyl (*S*)-3-(7-bromo-6-chloro-3-(3,5-difluoro-4-methoxyphenyl)-4-oxo-3,4-dihydroquinazolin-2-yl)piperidine-1-carboxylate**

(*S*)-4f (2.27 g, 38.8%) was prepared from $CuCl_2$ (0.34 g, 2.50 mmol), Boc-*S*-nipecotic acid (3.37 g, 14.70 mmol), dichloroethane (100 mL), *N*-methylimidazole (2.71 mL, 34.00 mmol), methanesulfonyl chloride (1.14 mL, 14.70 mmol), 2-amino-4-bromo-5-chlorobenzoic acid (2.51 g, 10.00 mmol), 3,5-difluoro-4-methoxyaniline (3.18 g, 20.00 mmol), triethylamine (12.13 mL, 87.00 mmol), and trimethylsilyl chloride (8.63 mL, 68.00 mmol) in the same manner described for **protocol A**. Brown solid. ^{1}H NMR (400 MHz, $CDCl_3$) δ 8.24 (s, 1H), 7.99 (s, 1H), 6.91 (s, 1H), 6.86 – 6.79 (m, 1H), 4.10 (s, 3H), 3.85 (s, 1H), 3.07 (t, *J* = 11.9 Hz, 1H), 2.74 (s, 1H), 2.40 (ddt, *J* = 10.3, 8.2, 4.0 Hz, 1H), 1.93 – 1.80 (m, 2H), 1.70 (dt, *J* = 13.6, 3.2 Hz, 1H), 1.41 (s, 9H), 1.30 (s,

1H). ^{19}F NMR (376 MHz, $CDCl_3$) δ -125.42 (d, *J* = 439.3 Hz). ^{13}C NMR (101 MHz, $CDCl_3$) δ 160.9, 158.0, 157.2, 155.5, 154.5, 146.2, 142.6, 138.2, 137.9, 133.4, 132.7, 130.0, 127.8, 120.7, 113.1, 99.0, 80.0, 62.4, 62.0, 41.0, 29.8, 28.4, 24.3.

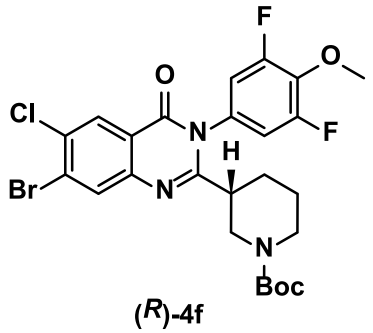

(*R*)-4f

***tert*-butyl (*R*)-3-(7-bromo-6-chloro-3-(3,5-difluoro-4-methoxyphenyl)-4-oxo-3,4-dihydroquinazolin-2-yl)piperidine-1-carboxylate**

(*R*)-4f (1.90 g, 42.1%) was prepared from $CuCl_2$ (0.27 g, 1.99 mmol), Boc-*R*-nipecotic acid (2.65 g, 11.57 mmol), dichloroethane (80 mL), *N*-methylimidazole (2.1 mL, 26.35 mmol), methanesulfonyl chloride (0.9 mL, 11.63 mmol), 2-amino-4-bromo-5-chlorobenzoic acid (1.94 g, 7.73 mmol), 3,5-difluoro-4-methoxyaniline (2.49 g, 15.63 mmol), triethylamine (9.5 mL, 68.16 mmol), and trimethylsilyl chloride (6.7 mL, 52.79 mmol) in the same manner described for **protocol A**. Brown solid. *N*-methylimidazole impurity observed in NMR. ^{1}H NMR (400 MHz, $CDCl_3$) δ 8.24 (s, 1H), 8.00 (s, 1H), 6.90 (s, 1H), 6.86 – 6.79 (m, 1H), 6.20 (impurity), 6.17 (impurity), 4.17 – 4.00 (m, 5H), 3.07 (t, *J* = 11.9 Hz, 1H), 2.81 – 2.65 (m, 1H), 2.40 (tt, *J* = 10.0, 4.1 Hz, 1H), 1.94 – 1.83 (m, 2H), 1.75 – 1.63 (m, 1H), 1.41 (s, 9H), 1.35 – 1.26 (m, 1H). ^{13}C NMR (101 MHz, $CDCl_3$) δ 160.9, 158.4, 157.2, 154.7, 154.5, 146.2, 138.2, 138.0, 137.9, 133.4, 132.7, 130.3, 130.2, 130.1, 130.0, 127.8, 120.7, 113.3, 113.1, 80.0, 62.0, 41.0, 29.8, 28.4, 24.4.

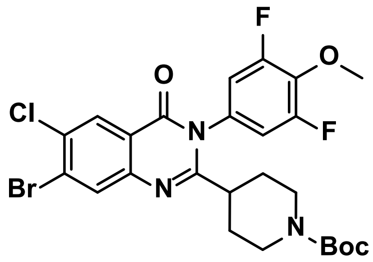

4g

***tert*-butyl 4-(7-bromo-6-chloro-3-(3,5-difluoro-4-methoxyphenyl)-4-oxo-3,4-dihydroquinazolin-2-yl)piperidine-1-carboxylate**

4g (0.16 g, 27.6%) was prepared from $CuCl_2$ (0.034 g, 0.25 mmol), 1-Boc-piperidine-4-carboxylic acid (0.34 g, 1.47 mmol), dichloroethane (10 mL), *N*-methylimidazole (0.27 mL, 3.40 mmol), methanesulfonyl chloride (0.11 mL, 1.47 mmol), 2-amino-4-bromo-5-chlorobenzoic acid (0.25 g, 1.00 mmol), 3,5-difluoro-4-methoxyaniline (0.32 g, 2.00 mmol), triethylamine (1.21 mL, 8.70 mmol), and trimethylsilyl chloride (0.86 mL, 6.80 mmol) in the same manner described for

protocol A. Brown solid. ^{1}H NMR (400 MHz, $CDCl_3$) δ 8.15 (s, 1H), 7.96 (s, 1H), 6.89 – 6.81 (m, 2H), 4.17 – 4.07 (m, 5H), 2.53 (s, 2H), 2.43 (tt, *J* = 11.3, 3.6 Hz, 1H), 1.96 – 1.79 (m, 2H), 1.74 – 1.65 (m, 2H), 1.44 (s, 9H). ^{19}F NMR (376 MHz, $CDCl_3$) δ -124.51 – -125.77 (m). ^{13}C NMR (101 MHz, $CDCl_3$) δ 160.9, 159.8, 157.1, 154.6, 146.2, 138.1, 137.8, 133.2, 132.6, 130.3, 130.2, 130.0, 127.6, 120.5, 113.2, 80.0, 62.0, 62.0, 40.8, 30.3, 28.5.

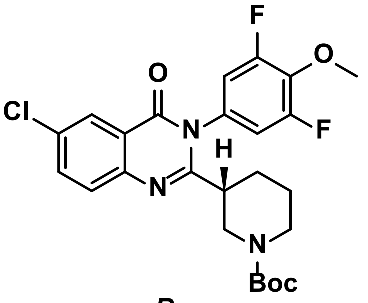

(*R*)-4h

***tert*-butyl (*R*)-3-(6-chloro-3-(3,5-difluoro-4-methoxyphenyl)-4-oxo-3,4-dihydroquinazolin-2-yl)piperidine-1-carboxylate**

(*R*)-4h (0.21 g, 24.2%) was prepared from $CuCl_2$ (0.059 g, 0.44 mmol), Boc-*R*-nipecotic acid (0.59 g, 2.58 mmol), dichloroethane (18 mL), *N*-methylimidazole (0.48 mL, 5.97 mmol), methanesulfonyl chloride (0.20 mL, 2.58 mmol), 2-amino-5-chlorobenzoic acid (0.30 g, 1.75 mmol), 3,5-difluoro-4-methoxyaniline (0.56 g, 3.51 mmol), triethylamine (2.13 mL, 15.26 mmol), and trimethylsilyl chloride (1.51 mL, 11.93 mmol) in the same manner described for **protocol A**. Brown solid. *N*-methylimidazole impurity observed in NMR. ^{1}H NMR (400 MHz, $CDCl_3$) δ 8.10 (d, *J* = 2.4 Hz, 1H), 7.65 (dd, *J* = 8.7, 2.4 Hz, 1H), 7.58 (d, *J* = 8.7 Hz, 1H), 6.90 (s, 1H), 6.86 – 6.80 (m, 1H), 6.16 (impurity), 6.13 (impurity), 4.16 – 3.94 (m, 5H), 3.08 (t, *J* = 11.8 Hz, 1H), 2.78 – 2.63 (m, 1H), 2.39 (dtd, *J* = 10.7, 7.7, 3.8 Hz, 1H), 1.95 – 1.82 (m, 2H), 1.68 (dp, *J* = 13.2, 3.2 Hz, 1H), 1.39 (s, 9H). ^{13}C NMR (101 MHz, $CDCl_3$) δ 161.2, 158.0, 157.9, 157.0, 155.5, 155.4, 154.6, 154.5, 145.7, 142.9, 142.7, 142.6, 138.0, 137.8, 137.7, 135.2, 132.8, 130.6, 130.5, 130.4, 129.1, 126.2, 121.6, 113.3, 113.1, 98.8, 98.8, 98.7, 98.6, 79.9, 62.3, 62.0, 61.9, 61.9, 40.8, 29.7, 28.3, 24.3.

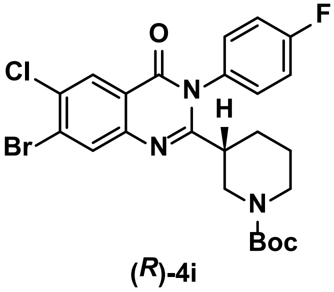

(*R*)-4i

***tert*-butyl (*R*)-3-(7-bromo-6-chloro-3-(4-fluorophenyl)-4-oxo-3,4-dihydroquinazolin-2-yl)piperidine-1-carboxylate**

(*R*)-4i (0.65 g, 40.0%) was prepared from $CuCl_2$ (0.10 g, 0.75 mmol), Boc-*R*-nipecotic acid (1.01 g, 4.41 mmol), dichloroethane (20 mL), *N*-methylimidazole (0.81 mL, 10.19 mmol),

methanesulfonyl chloride (0.34 mL, 4.41 mmol), 2-amino-4-bromo-5-chlorobenzoic acid (0.75 g, 3.00 mmol), 4-fluoroaniline (0.57 mL, 6.00 mmol), triethylamine (3.64 mL, 26.10 mmol), and trimethylsilyl chloride (2.59 mL, 20.40 mmol) in the same manner described for **protocol A**. Light brown solid. ^{1}H NMR (400 MHz, $CDCl_3$) δ 8.25 (s, 1H), 8.00 (s, 1H), 7.32 – 7.17 (m, 4H), 4.19 – 3.86 (m, 2H), 3.08 (t, *J* = 11.9 Hz, 1H), 2.72 (t, *J* = 10.5 Hz, 1H), 2.37 (tt, *J* = 10.5, 4.0 Hz, 1H), 1.93 – 1.77 (m, 2H), 1.66 (dt, *J* = 13.7, 3.3 Hz, 1H), 1.40 (s, 9H), 1.29 – 1.13 (m, 1H). ^{13}C NMR (101 MHz, $CDCl_3$) δ 164.3, 161.8, 161.1, 159.0, 154.5, 146.4, 133.1, 132.6, 132.4, 130.1, 130.0, 129.7, 127.9, 120.9, 117.6, 117.3, 79.8, 40.9, 29.8, 28.5, 24.4.

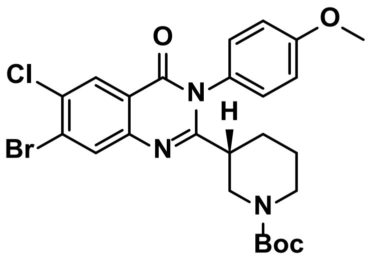

(*R*)-4j

***tert*-butyl (*R*)-3-(7-bromo-6-chloro-3-(4-methoxyphenyl)-4-oxo-3,4-dihydroquinazolin-2-yl)piperidine-1-carboxylate**

(*R*)-4j (0.80 g, 48.4%) was prepared from $CuCl_2$ (0.10 g, 0.75 mmol), Boc-*R*-nipecotic acid (1.01 g, 4.41 mmol), dichloroethane (20 mL), *N*-methylimidazole (0.81 mL, 10.19 mmol), methanesulfonyl chloride (0.34 mL, 4.41 mmol), 2-amino-4-bromo-5-chlorobenzoic acid (0.75 g, 3.00 mmol), 4-methoxyaniline (0.74 g, 6.00 mmol), triethylamine (3.64 mL, 26.10 mmol), and trimethylsilyl chloride (2.59 mL, 20.40 mmol) in the same manner described for **protocol A**. Brown solid. *N*-methylimidazole impurity observed in NMR. ^{1}H NMR (400 MHz, $CDCl_3$) δ 8.26 (s, 1H), 7.99 (s, 1H), 7.17 (s, 1H), 7.11 (dd, *J* = 8.7, 2.6 Hz, 1H), 7.08 – 7.01 (m, 2H), 6.74 (impurity), 6.72 (impurity), 6.64 (impurity), 6.62 (impurity), 4.19 – 3.92 (m, 1H), 3.87 (s, 3H), 3.08 (dd, *J* = 13.2, 10.6 Hz, 1H), 2.71 (t, *J* = 12.8 Hz, 1H), 2.44 (tt, *J* = 10.7, 3.8 Hz, 1H), 1.93 – 1.70 (m, 2H), 1.65 (dt, *J* = 13.5, 3.3 Hz, 1H), 1.40 (s, 9H), 1.24 (dt, *J* = 13.3, 9.2 Hz, 1H). ^{13}C NMR (101 MHz, $CDCl_3$) δ 161.4, 160.3, 159.6, 154.5, 146.5, 132.9, 132.5, 129.5, 129.1, 128.9, 127.9, 121.0, 116.5, 115.5, 114.9, 79.7, 55.7, 40.9, 29.9, 28.5, 24.4.

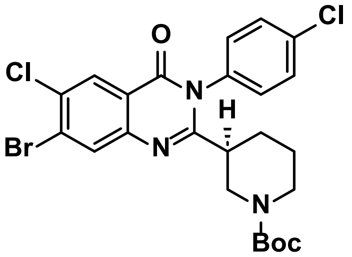

(*S*)-4k

***tert*-butyl (*S*)-3-(7-bromo-6-chloro-3-(4-chlorophenyl)-4-oxo-3,4-dihydroquinazolin-2-yl)piperidine-1-carboxylate**

***(S)*-4k** (0.24 g, 43.3%) was prepared from $CuCl_2$ (0.034 g, 0.25 mmol), *S*-Boc-nipecotic acid (0.34 g, 1.47 mmol), dichloroethane (10 mL), *N*-methylimidazole (0.27 mL, 3.40 mmol), methanesulfonyl chloride (0.11 mL, 1.47 mmol), 2-amino-4-bromo-5-chlorobenzoic acid (0.25 g, 1.00 mmol), 4-chloroaniline (0.26 g, 2.00 mmol), triethylamine (1.21 mL, 8.70 mmol), and trimethylsilyl chloride (0.86 mL, 6.80 mmol) in the same manner described for **protocol A**. Brown solid. ^{1}H NMR (400 MHz, $CDCl_3$) δ 8.27 (s, 1H), 8.01 (s, 1H), 7.60 – 7.51 (m, 2H), 7.24 (s, 1H), 7.16 (dd, *J* = 8.4, 2.6 Hz, 1H), 4.05 (d, *J* = 40.7 Hz, 1H), 3.08 (t, *J* = 11.9 Hz, 1H), 2.73 (t, *J* = 11.3 Hz, 1H), 2.37 (tt, *J* = 10.4, 4.0 Hz, 1H), 1.95 – 1.77 (m, 2H), 1.67 (dt, *J* = 13.5, 3.2 Hz, 1H), 1.40 (s, 9H), 1.30 – 1.15 (m, 1H). ^{13}C NMR (101 MHz, $CDCl_3$) δ 161.0, 158.7, 154.5, 146.4, 136.0, 135.0, 133.2, 132.6, 130.7, 130.4, 129.8, 129.6, 127.9, 120.9, 79.9, 41.0, 29.8, 28.5, 24.4.

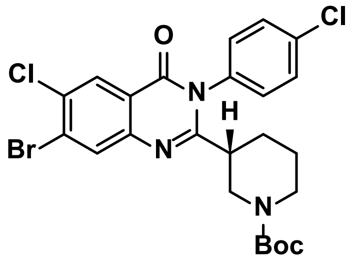

(*R*)-4k

***tert*-butyl (*R*)-3-(7-bromo-6-chloro-3-(4-chlorophenyl)-4-oxo-3,4-dihydroquinazolin-2-yl)piperidine-1-carboxylate**

***(R)*-4k** (2.27 g, 41.0%) was prepared from $CuCl_2$ (0.34 g, 2.50 mmol), Boc-*R*-nipecotic acid (3.37 g, 14.70 mmol), dichloroethane (100 mL), *N*-methylimidazole (2.70 mL, 34.00 mmol), methanesulfonyl chloride (1.10 mL, 14.70 mmol), 2-amino-4-bromo-5-chlorobenzoic acid (2.51 g, 10.00 mmol), 4-chloroaniline (2.55 g, 20.00 mmol), triethylamine (12.10 mL, 87.00 mmol), and trimethylsilyl chloride (8.60 mL, 68.00 mmol) in the same manner described for **protocol A**. Brown solid. *N*-methylimidazole impurity observed in NMR. ^{1}H NMR (400 MHz, $CDCl_3$) δ 8.24 (s, 1H), 8.00 (s, 1H), 7.58 – 7.49 (m, 2H), 7.24 (s, 1H), 7.16 (dd, *J* = 8.4, 2.6 Hz, 1H), 6.59 (impurity), 6.57 (impurity), 4.21 – 3.90 (m, 2H), 3.08 (t, *J* = 11.8 Hz, 1H), 2.73 (t, *J* = 10.7 Hz, 1H), 2.36 (tt, *J* = 10.5, 4.0 Hz, 1H), 1.93 – 1.76 (m, 2H), 1.70 – 1.62 (m, 1H), 1.40 (s, 9H), 1.31 – 1.14 (m, 1H). ^{13}C NMR (101 MHz, $CDCl_3$) δ 161.0, 158.7, 154.5, 146.3, 135.9, 135.0, 133.2, 132.6, 130.6, 130.4, 129.8, 129.6, 129.2, 127.8, 120.8, 116.3, 79.9, 40.9, 29.8, 28.5, 24.3.

Protocol B (refer to Scheme S1)[2]:

To oven-dried, inert gas-cooled glassware was added diamide and acetonitrile followed by triethylamine. The vessel was purged with nitrogen, and the reaction mixture was stirred at rt for 20 min. *N,O*-bis(trimethylsilyl)acetamide was added to the reaction mixture over 1 min, and the mixture was heated to 85°C with stirring for 20 h. After cooling to rt, the reaction mixture was concentrated *in vacuo*. The residue was diluted with ethyl acetate (120 mL) and washed two times with water (100 mL) and once with saturated brine solution (100 mL). The organic layer was dried

over anhydrous sodium sulfate, filtered, and the solvent was evaporated under reduced pressure. The residue was purified by flash chromatography (0% 3:1 dichloromethane : ethyl acetate / hexanes ~ 15% 3:1 dichloromethane : ethyl acetate / hexanes) to afford quinazolinone product.

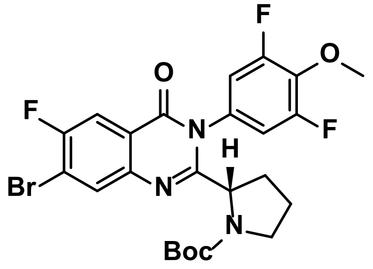

(*S*)-4a

***tert*-butyl (*S*)-2-(7-bromo-3-(3,5-difluoro-4-methoxyphenyl)-6-fluoro-4-oxo-3,4-dihydroquinazolin-2-yl)pyrrolidine-1-carboxylate**

(*S*)-4a (0.15 g, 66.0%) was prepared from **(*S*)-3a** (0.23 g, 0.41 mmol), acetonitrile (16.3 mL), triethylamine (1.70 mL, 12.21 mmol), and *N,O*-bis(trimethylsilyl)acetamide (1.49 mL, 6.11 mmol) in the same manner described for **protocol B**. Light brown solid. ^{1}H NMR (400 MHz, $CDCl_3$) δ 7.94 (dd, *J* = 22.5, 6.2 Hz, 1H), 7.87 (dd, *J* = 12.6, 8.1 Hz, 1H), 7.06 (ddt, *J* = 124.4, 10.4, 2.2 Hz, 1H), 6.83 (ddt, *J* = 12.6, 10.4, 2.2 Hz, 1H), 4.47 (ddd, *J* = 16.3, 7.9, 3.8 Hz, 1H), 4.09 (dt, *J* = 11.3, 1.2 Hz, 3H), 3.70 (dddd, *J* = 37.7, 10.2, 7.5, 5.3 Hz, 1H), 3.48 (ddt, *J* = 28.3, 10.0, 6.7 Hz, 1H), 2.18 – 1.76 (m, 4H), 1.35 (d, *J* = 53.4 Hz, 9H). ^{13}C NMR (101 MHz, $CDCl_3$) δ 161.3, 161.2, 161.2, 161.1, 158.8, 158.6, 158.4, 158.3, 158.1, 158.1, 157.4, 157.1, 157.1, 157.0, 156.9, 156.3, 156.1, 154.9, 154.8, 154.7, 154.6, 154.6, 154.4, 153.6, 144.8, 144.8, 144.5, 144.5, 138.0, 137.8, 137.7, 133.2, 133.0, 130.4, 130.2, 130.1, 129.9, 129.8, 129.6, 121.3, 121.2, 120.8, 120.7, 118.4, 118.2, 117.8, 117.6, 114.3, 114.3, 114.1, 114.0, 114.0, 114.0, 113.8, 113.7, 113.6, 113.6, 113.5, 113.4, 113.4, 113.4, 113.3, 113.2, 112.9, 112.8, 112.7, 112.6, 80.1, 79.9, 62.0, 58.3, 58.2, 47.3, 47.3, 33.0, 31.8, 28.7, 28.6, 24.1, 22.9.

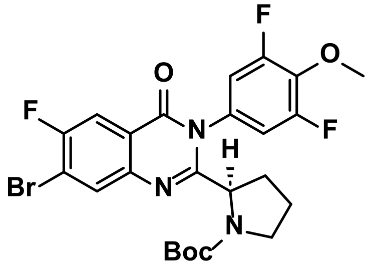

(*R*)-4a

***tert*-butyl (*R*)-2-(7-bromo-3-(3,5-difluoro-4-methoxyphenyl)-6-fluoro-4-oxo-3,4-dihydroquinazolin-2-yl)pyrrolidine-1-carboxylate**

(*R*)-4a (0.18 g, 64.8%) was prepared from **(*R*)-3a** (0.28 g, 0.49 mmol), acetonitrile (19.4 mL), triethylamine (2.03 mL, 14.57 mmol), and *N,O*-bis(trimethylsilyl)acetamide (1.78 mL, 7.29 mmol) in the same manner described for **protocol B**. Light brown solid. ^{1}H NMR (400 MHz, $CDCl_3$) δ

7.94 (dd, J = 22.5, 6.2 Hz, 1H), 7.87 (dd, J = 12.7, 8.1 Hz, 1H), 7.06 (ddt, J = 124.2, 10.4, 2.2 Hz, 1H), 6.83 (ddt, J = 12.7, 10.4, 2.2 Hz, 1H), 4.47 (ddd, J = 16.3, 7.9, 3.8 Hz, 1H), 4.09 (dt, J = 11.2, 1.2 Hz, 3H), 3.70 (dddd, J = 37.5, 10.1, 7.5, 5.3 Hz, 1H), 3.48 (ddt, J = 28.1, 10.0, 6.7 Hz, 1H), 2.18 – 1.76 (m, 3H), 1.35 (d, J = 53.5 Hz, 9H). ^{13}C NMR (101 MHz, $CDCl_3$) δ 161.3, 161.2, 161.2, 161.1, 158.8, 158.6, 158.4, 158.3, 158.1, 158.1, 157.4, 157.3, 157.2, 157.1, 157.1, 157.0, 156.9, 156.3, 156.1, 154.9, 154.8, 154.7, 154.6, 154.6, 154.5, 154.4, 153.6, 144.8, 144.8, 144.5, 144.5, 138.0, 137.8, 133.2, 133.0, 130.4, 130.2, 130.1, 129.9, 129.8, 129.6, 121.2, 121.2, 120.8, 120.7, 118.4, 118.2, 117.8, 117.6, 114.3, 114.3, 114.1, 114.0, 114.0, 114.0, 113.8, 113.8, 113.6, 113.6, 113.5, 113.4, 113.4, 113.4, 113.3, 113.2, 112.9, 112.8, 112.6, 112.6, 80.1, 79.9, 62.0, 58.3, 58.2, 47.3, 47.3, 33.0, 31.8, 28.7, 28.6, 24.1, 22.9.

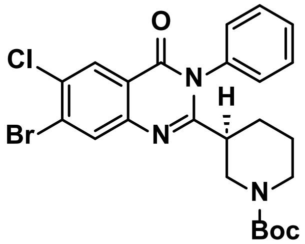

(*S*)-4b

***tert*-butyl (*S*)-3-(7-bromo-6-chloro-4-oxo-3-phenyl-3,4-dihydroquinazolin-2-yl)piperidine-1-carboxylate**

(*S*)-4b (0.23 g, 76.8%) was prepared from **(*S*)-3b** (0.31 g, 0.58 mmol), acetonitrile (23 mL), triethylamine (2.41 mL, 17.32 mmol), and *N,O*-bis(trimethylsilyl)acetamide (2.12 mL, 8.66 mmol) in the same manner described for **protocol B**. Light brown solid. ^{1}H NMR (400 MHz, $CDCl_3$) δ 8.27 (s, 1H), 8.01 (s, 1H), 7.62 – 7.50 (m, 3H), 7.31 – 7.27 (m, 1H), 7.21 (dt, J = 6.9, 2.3 Hz, 1H), 4.19 – 3.91 (m, 2H), 3.09 (dd, J = 13.2, 10.6 Hz, 1H), 2.71 (t, J = 13.4 Hz, 1H), 2.37 (tt, J = 10.7, 3.9 Hz, 1H), 1.93 – 1.75 (m, 2H), 1.64 (dq, J = 13.2, 3.2 Hz, 1H), 1.39 (s, 9H), 1.27 – 1.10 (m, 1H). ^{13}C NMR (101 MHz, $CDCl_3$) δ 161.1, 159.1, 154.5, 146.5, 136.6, 133.0, 132.5, 130.3, 129.8, 129.6, 128.2, 127.9, 121.1, 79.8, 40.9, 29.9, 28.5, 24.4.

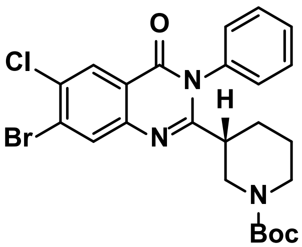

(*R*)-4b

***tert*-butyl (*R*)-3-(7-bromo-6-chloro-4-oxo-3-phenyl-3,4-dihydroquinazolin-2-yl)piperidine-1-carboxylate**

(*R*)-4b (0.16 g, 79.4%) was prepared from **(*R*)-3b** (0.20 g, 0.38 mmol), acetonitrile (15 mL), triethylamine (1.58 mL, 11.34 mmol), and *N,O*-bis(trimethylsilyl)acetamide (1.39 mL, 5.67 mmol) in the same manner described for **protocol B**. Light brown solid. ^{1}H NMR (400 MHz, $CDCl_3$) δ

8.29 (s, 1H), 8.02 (s, 1H), 7.62 – 7.51 (m, 3H), 7.28 (d, J = 7.6 Hz, 1H), 7.21 (dt, J = 7.0, 2.3 Hz, 1H), 4.21 – 3.90 (m, 2H), 3.09 (dd, J = 13.2, 10.6 Hz, 1H), 2.71 (t, J = 12.9 Hz, 1H), 2.38 (tt, J = 10.7, 3.8 Hz, 1H), 1.95 – 1.74 (m, 2H), 1.64 (dh, J = 11.1, 2.8 Hz, 1H), 1.39 (s, 9H), 1.22 – 1.10 (m, 1H). ^{13}C NMR (101 MHz, $CDCl_3$) δ 161.1, 159.2, 154.5, 146.5, 136.6, 133.0, 132.6, 130.4, 129.8, 129.6, 128.2, 127.9, 121.1, 79.8, 41.0, 29.9, 28.5, 24.4.

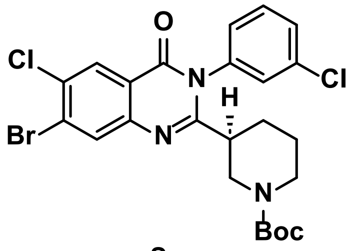

(*S*)-4c

***tert*-butyl (*S*)-3-(7-bromo-6-chloro-3-(3-chlorophenyl)-4-oxo-3,4-dihydroquinazolin-2-yl)piperidine-1-carboxylate**

(*S*)-4c (0.28 g, 51.3%) was prepared from **(*S*)-3c** (0.35 g, 0.62 mmol), acetonitrile (25 mL), triethylamine (2.58 mL, 18.49 mmol), and *N,O*-bis(trimethylsilyl)acetamide (2.26 mL, 9.24 mmol) in the same manner described for **protocol B**. Light brown solid. ^{1}H NMR (400 MHz, $CDCl_3$) δ 8.24 (s, 1H), 7.99 (s, 1H), 7.55 – 7.46 (m, 2H), 7.33 – 7.09 (m, 2H), 4.20 – 3.91 (m, 2H), 3.18 – 3.03 (m, 1H), 2.80 – 2.64 (m, 1H), 2.41 – 2.30 (m, 1H), 1.93 – 1.77 (m, 2H), 1.67 (ddt, J = 16.5, 13.8, 3.3 Hz, 1H), 1.40 (d, J = 7.2 Hz, 9H), 1.29 – 1.16 (m, 1H). ^{13}C NMR (101 MHz, $CDCl_3$) δ 160.9, 158.6, 158.5, 154.6, 146.3, 137.7, 133.2, 132.6, 132.6, 131.1, 130.2, 130.2, 129.8, 128.6, 127.8, 126.5, 120.8, 80.0, 79.8, 40.9, 30.1, 29.7, 28.5, 28.4, 24.3.

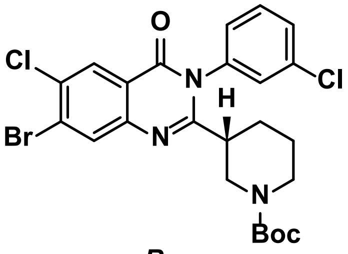

(*R*)-4c

***tert*-butyl (*R*)-3-(7-bromo-6-chloro-3-(3-chlorophenyl)-4-oxo-3,4-dihydroquinazolin-2-yl)piperidine-1-carboxylate**

(*R*)-4c (0.20 g, 77.3%) was prepared from **(*R*)-3c** (0.26 g, 0.46 mmol), acetonitrile (18.5 mL), triethylamine (1.90 mL, 13.65 mmol), and *N,O*-bis(trimethylsilyl)acetamide (1.67 mL, 6.83 mmol) in the same manner described for **protocol B**. Light brown solid. ^{1}H NMR (400 MHz, $CDCl_3$) δ 8.24 (s, 1H), 7.99 (s, 1H), 7.54 – 7.47 (m, 2H), 7.33 – 7.04 (m, 2H), 4.17 – 3.93 (m, 2H), 3.19 – 3.02 (m, 1H), 2.80 – 2.65 (m, 1H), 2.40 – 2.30 (m, 1H), 1.95 – 1.73 (m, 2H), 1.67 (ddt, J = 16.4, 9.9, 3.2 Hz, 1H), 1.40 (d, J = 7.2 Hz, 9H), 1.30 – 1.10 (m, 1H). ^{13}C NMR (101 MHz, $CDCl_3$) δ 160.9, 158.6, 158.5, 154.6, 146.3, 137.6, 136.0, 133.2, 132.6, 131.2, 130.2, 130.2, 129.8, 128.6, 127.8, 126.5, 120.8, 80.0, 79.8, 40.9, 30.1, 29.7, 28.5, 28.4, 24.3.

Protocol C (refer to Scheme S1):

Anthranilic acid, Boc-amino acid derivative or pyridyl acid, and triphenyl phosphite were added to pyridine and heated at 130 °C for 1 h. After allowing the reaction mixture to cool to rt, aniline was added to the reaction, and the resulting mixture was heated to 145 °C for 8 h. After cooling to rt, the reaction mixture was diluted with water (50 mL) and concentrated to evaporate off excess pyridine. The resulting residue was diluted with ethyl acetate (120 mL) and washed sequentially with sat. sodium bicarbonate solution (100 mL), water (100 mL), and sat. brine solution (100 mL). The organic layer was dried over anhydrous sodium sulfate, filtered, and the solvent was evaporated under reduced pressure. The residue was purified by flash chromatography (0% 4:1 ethyl acetate : dichloromethane / hexane ~ 25% 4:1 ethyl acetate : dichloromethane / hexane) to afford quinazolinone product.

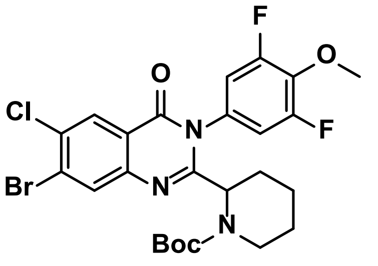

***rac*-4l**

***tert*-butyl 2-(7-bromo-6-chloro-3-(3,5-difluoro-4-methoxyphenyl)-4-oxo-3,4-dihydroquinazolin-2-yl)piperidine-1-carboxylate**

***rac*-4l** (0.95 g crude) was prepared from 2-amino-4-bromo-5-chlorobenzoic acid (1.13 g, 4.55 mmol), Boc-2-piperidine carboxylic acid (1.25 g, 5.46 mmol), triphenyl phosphite (2.63 mL, 10.01 mmol), pyridine (25 mL), and 3,5-difluoro-4-methoxyaniline (1.45 g, 9.10 mmol) in the same manner described for **protocol C**. Brown solid. Carried forward without further purification or analysis.

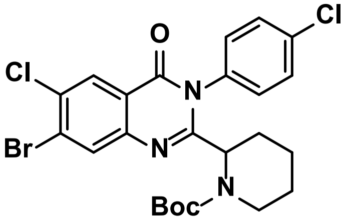

***rac*-4m**

***tert*-butyl 2-(7-bromo-6-chloro-3-(4-chlorophenyl)-4-oxo-3,4-dihydroquinazolin-2-yl)piperidine-1-carboxylate**

***rac*-4m** (0.95 g crude) was prepared from 2-amino-4-bromo-5-chlorobenzoic acid (1.13 g, 4.55 mmol), Boc-2-piperidine carboxylic acid (1.25 g, 5.46 mmol), triphenyl phosphite (2.63 mL, 10.01 mmol), pyridine (25 mL), and 4-chloroaniline (1.16 g, 9.10 mmol) in the same manner described for **protocol C**. Brown solid. Carried forward without further purification or analysis.

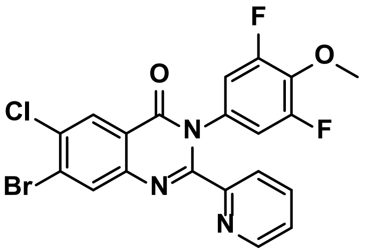

4o

7-bromo-6-chloro-3-(3,5-difluoro-4-methoxyphenyl)-2-(pyridin-2-yl)quinazolin-4(3*H*)-one

4o (0.44 g, 35.6%) was prepared from 2-amino-4-bromo-5-chlorobenzoic acid (0.65 g, 2.60 mmol), picolinic acid (0.38 g, 3.11 mmol), triphenyl phosphite (1.71 mL, 6.49 mmol), pyridine (17 mL), and 3,5-difluoro-4-methoxyaniline (0.50 g, 3.11 mmol) in the same manner described for **protocol C**. White solid. ^{1}H NMR (400 MHz, DMSO) δ 8.37 (dt, *J* = 4.7, 1.3 Hz, 1H), 8.29 (d, *J* = 8.1 Hz, 2H), 7.90 (td, *J* = 7.7, 1.7 Hz, 1H), 7.77 (dt, *J* = 7.8, 1.1 Hz, 1H), 7.37 (ddd, *J* = 7.7, 4.8, 1.2 Hz, 1H), 7.30 – 7.20 (m, 2H), 3.90 (s, 3H). ^{13}C NMR (101 MHz, DMSO) δ 159.9, 155.2, 155.2, 154.2, 152.8, 152.7, 152.1, 148.2, 146.2, 137.0, 135.8, 135.7, 135.6, 132.7, 132.3, 131.9, 131.8, 131.7, 129.6, 128.8, 127.3, 125.0, 124.5, 121.8, 121.5, 114.6, 114.5, 114.4, 114.3, 61.8.

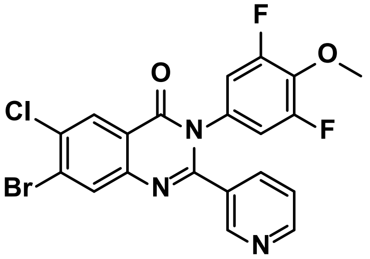

4p

7-bromo-6-chloro-3-(3,5-difluoro-4-methoxyphenyl)-2-(pyridin-3-yl)quinazolin-4(3*H*)-one

4p (0.44 g, 35.6%) was prepared from 2-amino-4-bromo-5-chlorobenzoic acid (0.75 g, 2.99 mmol), nicotinic acid (0.44 g, 3.59 mmol), triphenyl phosphite (1.97 mL, 7.49 mmol), pyridine (20 mL), and 3,5-difluoro-4-methoxyaniline (0.57 g, 3.53 mmol) in the same manner described for **protocol C**. White solid. ^{1}H NMR (400 MHz, DMSO) δ 8.63 (dd, *J* = 2.2, 0.9 Hz, 1H), 8.53 (dd, *J* = 4.9, 1.7 Hz, 1H), 8.30 (d, *J* = 3.8 Hz, 2H), 7.79 (dt, *J* = 8.0, 2.0 Hz, 1H), 7.42 – 7.33 (m, 3H), 3.91 (d, *J* = 1.2 Hz, 3H). ^{13}C NMR (101 MHz, DMSO) δ 159.9, 155.5, 155.4, 154.2, 153.0, 152.9, 150.3, 149.0, 146.3, 136.2, 136.1, 132.6, 132.2, 131.6, 131.5, 131.4, 130.8, 130.0, 128.9, 127.2, 125.4, 122.8, 121.2, 120.5, 114.9, 114.8, 114.7, 114.6, 61.8.

Protocol D (refer to Scheme S1)[3]:

Anthranilic acid, EDC hydrochloride, 1-hydroxybenzotriazole, and methylammonium chloride were added to a flask with dimethylformamide and allowed to stir at rt for 30 min. *N,N*-

diisopropylethylamine was added slowly to the reaction mixture over 2 min, and the reaction mixture was stirred at rt for 20 h. The reaction mixture was quenched with water (25 mL). The resulting mixture was extracted three times with ethyl acetate (3 x 60 mL). The organic layers were then combined and washed sequentially with water (100 mL), sat. sodium bicarbonate solution (100 mL), and sat. brine solution (100 mL). The organic layer was dried over anhydrous sodium sulfate, filtered, and the solvent was evaporated under reduced pressure to afford anthranilic amide product.

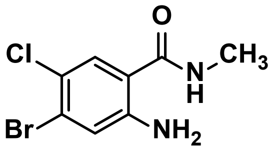

5a

2-amino-4-bromo-5-chloro-*N*-methylbenzamide

5a (0.59 g, 89.2%) was prepared from 2-amino-4-bromo-5-chlorobenzoic acid (0.63 g, 2.50 mmol), methylammonium chloride (0.71 g, 10.50 mmol), EDC hydrochloride (0.58 g, 3.00 mmol), HOBT (0.44 g, 3.25 mmol), dimethylformamide (5.6 mL), and DIPEA (1.83 mL, 10.50 mmol) in the same manner described for **protocol D**. Light brown solid. ^{1}H NMR (400 MHz, DMSO) δ 8.37 (d, J = 4.9 Hz, 1H), 7.67 (s, 1H), 7.10 (s, 1H), 6.70 (s, 2H), 2.71 (d, J = 4.5 Hz, 3H). ^{13}C NMR (101 MHz, DMSO) δ 167.4, 149.3, 129.1, 124.4, 120.2, 117.3, 115.2, 26.0.

Protocol E (refer to Scheme S1):

Copper chloride and Boc-amino acid derivative were added to an oven dried flask that was cooled under N_2, then anhydrous dichloroethane was added to the flask. The mixture was cooled to 0 °C using an ice-water bath and allowed to stir for 10 min until *N*-methylimidazole was added dropwise over 2 min. Methanesulfonyl chloride was added dropwise over 2 min, the ice bath was removed, and the reaction was allowed to reach rt over 2 h. The anthranilic amide was added, the reaction mixture was purged with N_2, and after stirring at rt for 16 h, triethylamine was added over 2 min, and then trimethylsilyl chloride was added over 2 min. The reaction mixture was heated at 85 °C for 24 h. After cooling to rt, the mixture was diluted with dichloromethane (120 mL) and washed sequentially with 1 N HCl (100 mL), sat. sodium bicarbonate solution (100 mL), and sat. brine solution (100 mL). The organic layer was dried over anhydrous sodium sulfate, filtered, and the solvent was evaporated under reduced pressure. The residue was purified by flash chromatography (0% ethyl acetate / hexane ~ 50% ethyl acetate / hexane) to afford quinazolinone product.

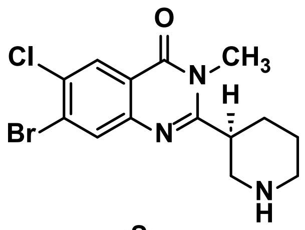

(*S*)-4n

(*S*)-7-bromo-6-chloro-3-methyl-2-(piperidin-3-yl)quinazolin-4(3*H*)-one

(*S*)-4n (0.39 g, 86.2%) was prepared from $CuCl_2$ (0.068 g, 0.50 mmol), *S*-Boc-nipecotic acid (0.34 g, 1.47 mmol), dichloroethane (10 mL), *N*-methylimidazole (0.27 mL, 3.40 mmol), methanesulfonyl chloride (0.11 mL, 1.47 mmol), **5a** (0.26 g, 1.00 mmol), triethylamine (1.21 mL, 8.70 mmol), and trimethylsilyl chloride (0.86 mL, 6.80 mmol) in the same manner described for **protocol E**. Brown solid. ^{1}H NMR (400 MHz, $CDCl_3$) δ 8.29 (s, 1H), 7.94 (s, 1H), 4.39 – 4.08 (m, 2H), 3.67 (s, 3H), 3.06 (s, 1H), 2.90 (tt, *J* = 10.8, 3.3 Hz, 1H), 2.81 (t, *J* = 10.7 Hz, 1H), 2.13 – 2.04 (m, 1H), 1.98 – 1.78 (m, 2H), 1.65 – 1.54 (m, 1H), 1.47 (s, 9H). ^{13}C NMR (101 MHz, $CDCl_3$) δ 161.3, 158.7, 146.2, 132.8, 132.5, 129.2, 127.7, 120.5, 80.1, 41.0, 30.4, 29.5, 28.6, 25.0.

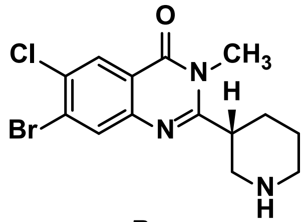

(*R*)-4n

(*R*)-7-bromo-6-chloro-3-methyl-2-(piperidin-3-yl)quinazolin-4(3*H*)-one

(*R*)-4n (0.37 g, 80.7%) was prepared from $CuCl_2$ (0.068 g, 0.50 mmol), *R*-Boc-nipecotic acid (0.34 g, 1.47 mmol), dichloroethane (10 mL), *N*-methylimidazole (0.27 mL, 3.40 mmol), methanesulfonyl chloride (0.11 mL, 1.47 mmol), **5a** (0.26 g, 1.00 mmol), triethylamine (1.21 mL, 8.70 mmol), and trimethylsilyl chloride (0.86 mL, 6.80 mmol) in the same manner described for **protocol E**. Brown solid. ^{1}H NMR (400 MHz, $CDCl_3$) δ 8.29 (s, 1H), 7.94 (s, 1H), 4.38 – 4.08 (m, 2H), 3.67 (s, 3H), 3.14 – 2.99 (m, 1H), 2.90 (ddt, *J* = 10.7, 6.8, 3.3 Hz, 1H), 2.80 (t, *J* = 9.4 Hz, 1H), 2.12 – 2.05 (m, 1H), 1.98 – 1.78 (m, 2H), 1.66 – 1.54 (m, 1H), 1.47 (s, 9H). ^{13}C NMR (101 MHz, $CDCl_3$) δ 161.3, 158.7, 146.2, 132.7, 132.5, 129.1, 127.7, 120.5, 80.1, 41.0, 30.4, 29.5, 28.6, 25.0.

Scheme S2. Intermediate Derivatization to Yield Analogs

(Reproduced from Scheme 2 in main manuscript)

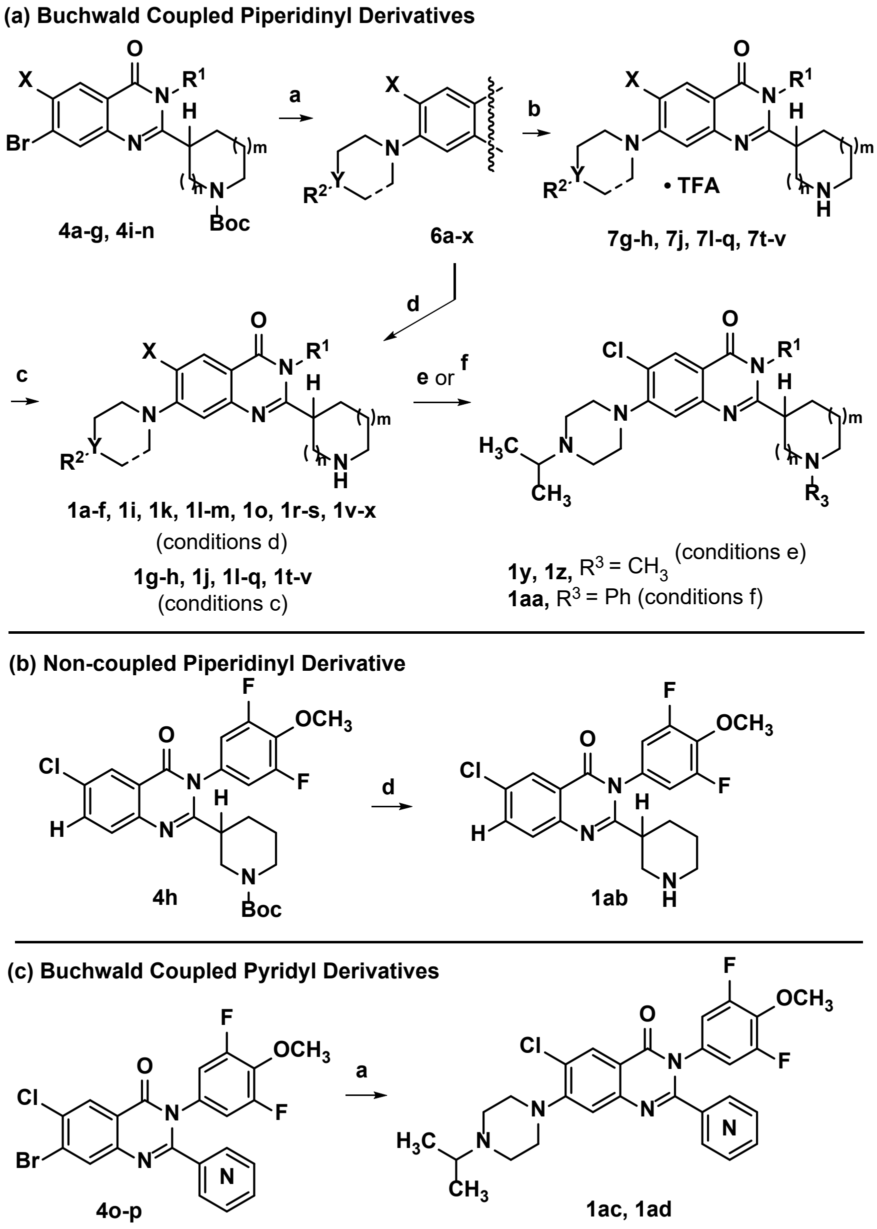

Protocols A1 and A2 (refer to Scheme S2):

A1 To an oven-dried, inert gas-cooled flask was added the quinazolinone, amine, $Pd_2(dba)_3$, Xantphos, cesium carbonate, and anhydrous toluene. The reaction mixture was sparged with N_2 for 5 min, heated to 100 °C, and allowed to stir for 24 h. After cooling to rt, the mixture was diluted with ethyl acetate (120 mL) and washed three times with sat. brine solution (3x 80 mL). The organic layer was dried over anhydrous sodium sulfate, filtered, and the solvent was evaporated

under reduced pressure. The residue was purified by flash chromatography (0% methanol / dichloromethane ~ 10% methanol / dichloromethane) to afford the aminated product.

A2 To an oven-dried, inert gas-cooled flask was added $Pd_2(dba)_3$, Xantphos, cesium carbonate, and anhydrous toluene. The reaction mixture was sparged with N_2 for 5 min and allowed to stir for 15 min. In a separate flask, the quinazolinone was dissolved in anhydrous toluene, then added to the reaction mixture, sparged with N_2 for 5 min, and allowed to stir for 15 min. In a separate flask, the piperazine was dissolved in toluene, then added to the reaction mixture and sparged with N_2 for 5 min. The reaction mixture was heated to 100 °C and allowed to stir for 24 h. After cooling to rt, the mixture was diluted with ethyl acetate (120 mL) and washed three times with sat. brine solution (3x 80 mL). The organic layer was dried over anhydrous sodium sulfate, filtered, and the solvent was evaporated under reduced pressure. The residue was purified by flash chromatography (0% methanol / dichloromethane ~ 10% methanol / dichloromethane) to afford the aminated product.

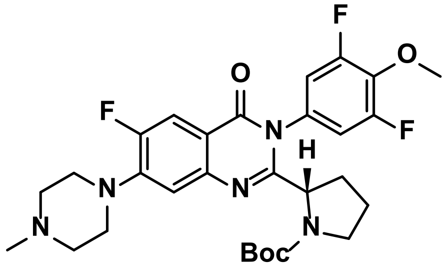

(*S*)-6a

***tert*-butyl (*S*)-2-(3-(3,5-difluoro-4-methoxyphenyl)-6-fluoro-7-(4-methylpiperazin-1-yl)-4-oxo-3,4-dihydroquinazolin-2-yl)pyrrolidine-1-carboxylate**

(*S*)-6a (0.17 g, 74.5%) was prepared from $Pd_2(dba)_3$ (0.018 g, 0.019 mmol), Xantphos (0.022 g, 0.039 mmol), cesium carbonate (0.18 g, 0.54 mmol), anhydrous toluene (4.0 mL), **(*S*)-4a** (0.22 g, 0.39 mmol), and 1-methylpiperazine (47.3 μL, 0.40 mmol), in the same manner described for **protocol A2**. Light orange solid. ^{1}H NMR (400 MHz, $CDCl_3$) δ 7.75 (dd, *J* = 12.9, 7.7 Hz, 1H), 7.05 (dd, *J* = 10.3, 7.8 Hz, 1H), 7.04 (ddt, *J* = 116.6, 10.4, 2.2 Hz, 1H), 6.80 (tt, *J* = 9.9, 2.2 Hz, 1H), 4.45 (ddd, *J* = 23.0, 7.8, 4.0 Hz, 1H), 4.07 (d, *J* = 10.2 Hz, 3H), 3.70 (dddd, *J* = 41.7, 10.0, 7.2, 5.4 Hz, 1H), 3.48 (ddt, *J* = 31.9, 10.2, 6.5 Hz, 1H), 3.30 (td, *J* = 10.8, 5.6 Hz, 5H), 2.61 (t, *J* = 4.8 Hz, 4H), 2.37 (s, 3H), 2.16 – 1.75 (m, 3H), 1.35 (d, *J* = 56.2 Hz, 9H). ^{13}C NMR (101 MHz, $CDCl_3$) δ 161.3, 157.1, 156.9, 155.6, 154.4, 153.8, 153.1, 147.0, 146.9, 146.8, 146.7, 145.7, 145.5, 137.8, 130.5, 115.8, 115.7, 115.7, 114.4, 114.2, 114.0, 113.8, 113.6, 113.5, 113.5, 112.8, 112.7, 112.6, 112.5, 80.0, 79.7, 62.0, 58.4, 58.2, 55.0, 50.0, 50.0, 47.4, 47.3, 46.2, 33.0, 31.8, 28.6, 28.6, 24.1, 23.0.

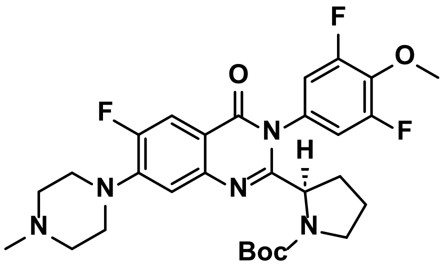

(*R*)-6a

tert-butyl (*R*)-2-(3-(3,5-difluoro-4-methoxyphenyl)-6-fluoro-7-(4-methylpiperazin-1-yl)-4-oxo-3,4-dihydroquinazolin-2-yl)pyrrolidine-1-carboxylate

(*R*)-6a (0.12 g, 54.1%) was prepared from $Pd_2(dba)_3$ (0.019 g, 0.021 mmol), Xantphos (0.025 g, 0.042 mmol), cesium carbonate (0.19 g, 0.59 mmol), anhydrous toluene (4.5 mL), **(*R*)-4a** (0.24 g, 0.42 mmol), and 1-methylpiperazine (51.7 μL, 0.47 mmol), in the same manner described for **protocol A2**. Orange solid. ^{1}H NMR (400 MHz, $CDCl_3$) δ 7.75 (dd, *J* = 12.9, 7.6 Hz, 1H), 7.05 (dd, *J* = 10.3, 7.8 Hz, 1H), 7.04 (ddt, *J* = 116.5, 10.4, 2.2 Hz, 1H), 6.80 (tt, *J* = 9.8, 2.2 Hz, 1H), 4.45 (ddd, *J* = 23.0, 7.8, 4.0 Hz, 1H), 4.07 (d, *J* = 10.2 Hz, 3H), 3.80 – 3.60 (m, 1H), 3.48 (ddt, *J* = 32.0, 10.2, 6.6 Hz, 1H), 3.30 (dq, *J* = 9.7, 5.6 Hz, 4H), 2.61 (t, *J* = 4.9 Hz, 4H), 2.37 (s, 3H), 2.18 – 1.75 (m, 4H), 1.35 (d, *J* = 56.2 Hz, 9H). ^{13}C NMR (101 MHz, $CDCl_3$) δ 161.4, 161.3, 157.1, 156.9, 155.6, 154.6, 154.4, 153.8, 153.1, 147.0, 146.9, 145.7, 145.5, 137.8, 130.5, 115.8, 115.7, 115.7, 114.4, 114.2, 114.0, 113.6, 113.5, 113.5, 112.8, 112.7, 112.6, 112.5, 80.0, 79.7, 62.0, 58.4, 58.2, 55.0, 50.0, 50.0, 47.4, 47.3, 46.2, 33.0, 31.8, 28.6, 28.6, 24.1, 23.0.

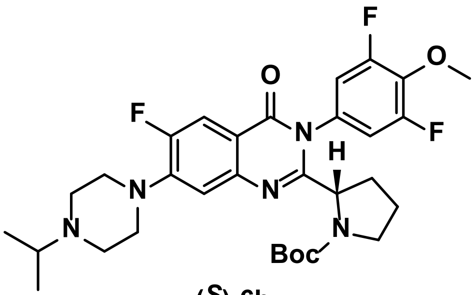

(*S*)-6b

tert-butyl (*S*)-2-(3-(3,5-difluoro-4-methoxyphenyl)-6-fluoro-7-(4-isopropylpiperazin-1-yl)-4-oxo-3,4-dihydroquinazolin-2-yl)pyrrolidine-1-carboxylate

(*S*)-6b (0.12 g, 54.1%) was prepared from $Pd_2(dba)_3$ (0.017 g, 0.018 mmol), Xantphos (0.021 g, 0.036 mmol), cesium carbonate (0.17 g, 0.51 mmol), anhydrous toluene (3.6 mL), **(*S*)-4a** (0.20 g, 0.36 mmol), and 1-isopropylpiperazine (57.1 μL, 0.40 mmol), in the same manner described for **protocol A2**. Orange solid. ^{1}H NMR (400 MHz, $CDCl_3$) δ 7.74 (dd, *J* = 13.0, 7.4 Hz, 1H), 7.04 (t, *J* = 8.6 Hz, 1H), 7.03 (ddt, *J* = 116.2, 10.4, 2.2 Hz, 1H), 6.80 (tt, *J* = 9.0, 2.2 Hz, 1H), 4.45 (ddd, *J* = 23.8, 7.8, 4.1 Hz, 1H), 4.07 (d, *J* = 10.2 Hz, 3H), 3.70 (ddt, *J* = 40.8, 9.4, 6.7 Hz, 1H), 3.48 (ddt, *J* = 32.2, 10.2, 6.5 Hz, 1H), 3.29 (dq, *J* = 7.2, 3.8 Hz, 4H), 2.80 – 2.67 (m, 5H), 2.15 – 1.73 (m, 3H), 1.34 (d, *J* = 58.2 Hz, 9H), 1.09 (d, *J* = 6.5 Hz, 6H). ^{13}C NMR (101 MHz, $CDCl_3$) δ 161.4, 161.3, 161.3, 157.1, 157.0, 156.8, 155.6, 154.6, 154.4, 153.8, 153.1, 147.1, 147.0, 146.9, 146.8,

145.7, 145.5, 137.7, 130.5, 115.7, 115.6, 115.5, 114.2, 113.9, 113.8, 113.6, 113.5, 113.4, 112.8, 112.7, 112.6, 112.5, 80.0, 79.7, 62.0, 58.4, 58.2, 54.7, 50.4, 48.6, 47.4, 47.3, 32.9, 31.8, 28.6, 24.1, 23.1, 18.6.

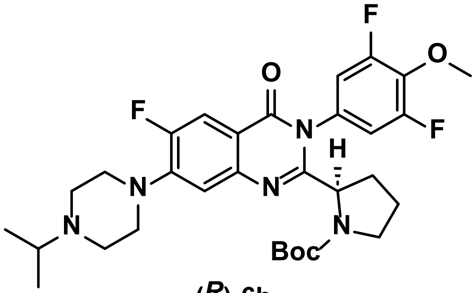

(*R*)-6b

***tert*-butyl (*R*)-2-(3-(3,5-difluoro-4-methoxyphenyl)-6-fluoro-7-(4-isopropylpiperazin-1-yl)-4-oxo-3,4-dihydroquinazolin-2-yl)pyrrolidine-1-carboxylate**

(*R*)-6b (0.14 g, 55.4%) was prepared from $Pd_2(dba)_3$ (0.019 g, 0.020 mmol), Xantphos (0.024 g, 0.041 mmol), cesium carbonate (0.19 g, 0.57 mmol), anhydrous toluene (4.0 mL), **(*R*)-4a** (0.23 g, 0.41 mmol), and 1-isopropylpiperazine (64.2 μL, 0.45 mmol), in the same manner described for **protocol A2**. Light orange solid. ^{1}H NMR (400 MHz, $CDCl_3$) δ 7.74 (dd, *J* = 12.9, 7.5 Hz, 1H), 7.03 (dd, *J* = 9.3, 7.8 Hz, 1H), 7.03 (ddt, *J* = 116.1, 10.4, 2.2 Hz, 1H), 6.80 (tt, *J* = 9.1, 2.2 Hz, 1H), 4.45 (ddd, *J* = 23.8, 7.8, 4.0 Hz, 1H), 4.07 (d, *J* = 10.2 Hz, 3H), 3.70 (dddd, *J* = 40.4, 9.8, 7.2, 5.5 Hz, 1H), 3.48 (ddt, *J* = 32.0, 10.3, 6.5 Hz, 1H), 3.35 – 3.21 (m, 4H), 2.82 – 2.66 (m, 5H), 2.20 – 1.73 (m, 4H), 1.34 (d, *J* = 58.2 Hz, 9H), 1.09 (d, *J* = 6.5 Hz, 6H). ^{13}C NMR (101 MHz, $CDCl_3$) δ 161.4, 161.3, 157.1, 157.0, 156.8, 155.5, 154.6, 154.4, 153.8, 153.1, 147.1, 147.0, 146.8, 146.7, 145.7, 145.5, 137.7, 130.6, 130.5, 115.7, 115.6, 115.5, 114.2, 114.2, 114.0, 113.9, 113.8, 113.6, 113.6, 113.5, 113.4, 112.8, 112.7, 112.5, 112.4, 79.9, 79.7, 62.0, 58.4, 58.2, 54.7, 50.3, 50.3, 48.6, 47.3, 47.3, 32.9, 31.8, 28.6, 24.1, 23.0, 18.6.

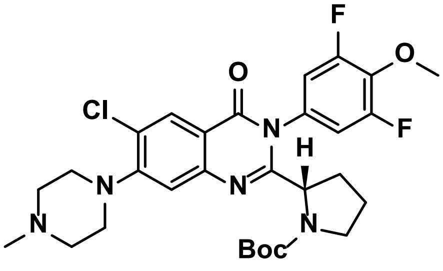

(*S*)-6c

***tert*-butyl (*S*)-2-(6-chloro-3-(3,5-difluoro-4-methoxyphenyl)-7-(4-methylpiperazin-1-yl)-4-oxo-3,4-dihydroquinazolin-2-yl)pyrrolidine-1-carboxylate**

(*S*)-6c (0.58 g, 45.0%) was prepared from **(*S*)-4d** (1.20 g, 2.10 mmol), 1-methylpiperazine (0.23 mL, 2.10 mmol), XPhos Pd G3 (0.14 g, 0.16 mmol), cesium carbonate (2.05 g, 6.30 mmol), and anhydrous toluene (21 mL) in the same manner described for **protocol A1**. Orange solid. ^{1}H NMR (400 MHz, $CDCl_3$) δ 8.20 – 8.08 (m, 1H), 7.24 – 7.14 (m, 1H), 6.94 – 6.70 (m, 2H), 6.37 (dd, *J* =

21.6, 9.2 Hz, 1H), 4.47 (dddd, J = 28.9, 17.8, 7.1, 3.1 Hz, 1H), 4.06 (d, J = 11.2 Hz, 2H), 3.99 (d, J = 7.4 Hz, 1H), 3.91 (d, J = 4.7 Hz, 2H), 3.81 (d, J = 3.9 Hz, 1H), 3.66 – 3.54 (m, 1H), 3.54 – 3.40 (m, 2H), 3.35 (d, J = 12.5 Hz, 1H), 3.18 (s, 3H), 2.74 (s, 1H), 2.69 (s, 1H), 2.20 – 1.85 (m, 1H), 1.45 – 1.35 (m, 8H), 1.25 (s, 4H). ^{19}F NMR (376 MHz, $CDCl_3$) δ -125.52 – - 126.25 (m), -126.28 – -126.83 (m). ^{13}C NMR (101 MHz, $CDCl_3$) δ 160.9, 157.0, 153.6, 147.7, 141.1, 137.8, 132.6, 129.6, 127.6, 125.5, 123.7, 120.6, 117.9, 115.9, 108.7, 80.6, 62.3, 57.0, 54.1, 49.4, 47.3, 46.6, 33.4, 31.8, 31.0, 28.6, 24.0.

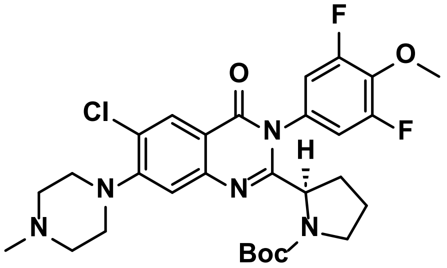

(*R*)-6c

***tert*-butyl (*R*)-2-(6-chloro-3-(3,5-difluoro-4-methoxyphenyl)-7-(4-methylpiperazin-1-yl)-4-oxo-3,4-dihydroquinazolin-2-yl)pyrrolidine-1-carboxylate**

(*R*)-6c (0.075 g, 30.8%) was prepared from $Pd_2(dba)_3$ (0.019 g, 0.021 mmol), Xantphos (0.024 g, 0.042 mmol), cesium carbonate (0.19 g, 0.58 mmol), anhydrous toluene (4.2 mL), **(*R*)-4d** (0.24 g, 0.42 mmol), and 1-methylpiperazine (50.7 μL, 0.46 mmol), in the same manner described for **protocol A2**. Light orange solid. ^{1}H NMR (400 MHz, $CDCl_3$) δ 8.14 (d, J = 8.8 Hz, 1H), 7.21 – 6.86 (m, 2H), 6.80 (tt, J = 9.3, 2.2 Hz, 1H), 4.44 (ddd, J = 20.6, 7.9, 4.1 Hz, 1H), 4.07 (d, J = 10.3 Hz, 3H), 3.80 – 3.61 (m, 1H), 3.48 (ddt, J = 32.3, 10.3, 6.5 Hz, 1H), 3.31 – 3.15 (m, 4H), 2.63 (t, J = 4.8 Hz, 4H), 2.38 (s, 3H), 2.17 – 1.75 (m, 4H), 1.34 (d, J = 55.6 Hz, 9H). ^{13}C NMR (101 MHz, $CDCl_3$) δ 161.1, 161.0, 158.1, 157.9, 155.4, 155.0, 154.4, 153.8, 147.7, 147.5, 137.8, 130.7, 130.3, 128.8, 128.7, 127.7, 127.6, 117.6, 116.0, 115.3, 114.1, 114.1, 113.9, 113.9, 113.8, 113.8, 113.6, 113.6, 113.4, 113.4, 80.0, 79.7, 62.0, 58.5, 58.3, 55.1, 51.1, 51.1, 47.4, 47.3, 46.2, 33.0, 31.8, 28.6, 28.6, 24.1, 23.1.

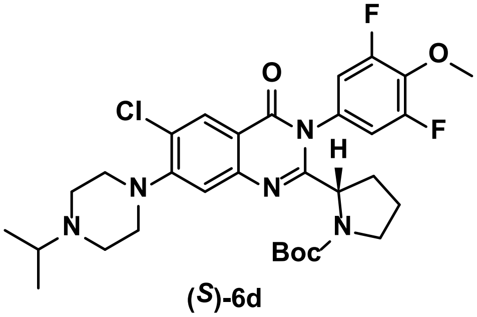

(*S*)-6d

***tert*-butyl (*S*)-2-(6-chloro-3-(3,5-difluoro-4-methoxyphenyl)-7-(4-isopropylpiperazin-1-yl)-4-oxo-3,4-dihydroquinazolin-2-yl)pyrrolidine-1-carboxylate**

(*S*)-6d (0.073 g, 20.4%) was prepared from **(*S*)-4d** (0.33 g, 0.58 mmol), 1-isopropylpiperazine (86.7 μL, 0.61mmol), $Pd_2(dba)_3$ (0.026 g, 0.029 mmol), Xantphos (0.033 g, 0.058 mmol), cesium carbonate (0.26 g, 0.81 mmol), and anhydrous toluene (5.8 mL) in the same manner described for **protocol A1**. Orange solid. ^{1}H NMR (400 MHz, $CDCl_3$) δ 8.16 (d, *J* = 7.8 Hz, 1H), 7.22 – 6.86 (m, 2H), 6.81 (ddt, *J* = 10.4, 8.2, 2.2 Hz, 1H), 4.45 (ddd, *J* = 22.1, 7.8, 4.1 Hz, 1H), 4.08 (d, *J* = 10.3 Hz, 3H), 3.82 – 3.60 (m, 1H), 3.49 (ddt, *J* = 33.5, 10.3, 6.5 Hz, 1H), 3.24 (h, *J* = 6.1 Hz, 5H), 2.77 (dq, *J* = 10.0, 5.9 Hz, 5H), 2.17 – 1.77 (m, 3H), 1.35 (d, *J* = 57.5 Hz, 9H), 1.12 (dd, *J* = 6.6, 2.4 Hz, 6H). ^{13}C NMR (101 MHz, $CDCl_3$) δ 161.2, 161.1, 158.0, 157.8, 155.5, 155.1, 154.5, 153.8, 147.7, 147.5, 137.7, 130.8, 130.3, 128.8, 128.7, 127.9, 127.7, 127.5, 117.6, 117.5, 116.0, 115.2, 114.4, 114.2, 114.0, 113.8, 113.8, 113.6, 113.4, 80.0, 79.8, 62.0, 58.5, 58.3, 54.8, 54.7, 51.4, 48.7, 47.4, 47.3, 33.0, 31.8, 28.7, 28.6, 24.1, 23.1, 18.7, 18.7.

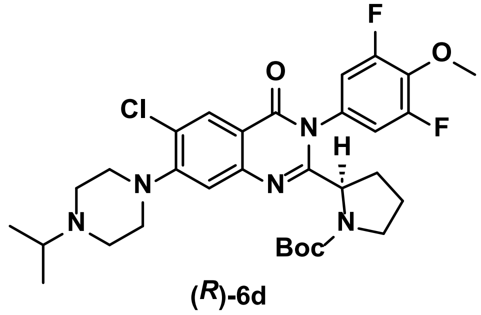

(*R*)-6d

***tert*-butyl (*R*)-2-(6-chloro-3-(3,5-difluoro-4-methoxyphenyl)-7-(4-isopropylpiperazin-1-yl)-4-oxo-3,4-dihydroquinazolin-2-yl)pyrrolidine-1-carboxylate**

(*R*)-6d (0.049 g, 23.6%) was prepared from $Pd_2(dba)_3$ (0.015 g, 0.017 mmol), Xantphos (0.019 g, 0.033 mmol), cesium carbonate (0.15 g, 0.47 mmol), anhydrous toluene (3.3 mL), **(*R*)-4d** (0.19 g, 0.33 mmol), and 1-isopropylpiperazine (52.7 μL, 0.37 mmol), in the same manner described for **protocol A2**. Orange solid. ^{1}H NMR (400 MHz, $CDCl_3$) δ 8.14 (d, *J* = 8.0 Hz, 1H), 7.21 – 6.85 (m, 2H), 6.85 – 6.76 (m, 1H), 4.44 (ddd, *J* = 21.8, 7.9, 4.1 Hz, 1H), 4.07 (d, *J* = 10.2 Hz, 3H), 3.71 (ddt, *J* = 38.8, 9.3, 6.6 Hz, 1H), 3.48 (ddt, *J* = 32.4, 10.2, 6.5 Hz, 1H), 3.23 (tq, *J* = 11.5, 5.5 Hz, 5H), 2.82 – 2.69 (m, 5H), 2.16 – 1.76 (m, 3H), 1.34 (d, *J* = 57.6 Hz, 9H), 1.11 (d, *J* = 6.4 Hz, 6H). ^{13}C NMR (101 MHz, $CDCl_3$) δ 161.2, 161.0, 158.0, 157.8, 155.4, 155.1, 154.6, 154.5, 153.8, 147.6, 147.5, 137.8, 130.7, 130.3, 128.8, 128.7, 127.7, 127.5, 117.5, 117.4, 115.9, 115.2, 114.4, 114.3, 114.2, 114.1, 113.9, 113.8, 113.8, 113.6, 113.6, 113.4, 113.4, 80.0, 79.7, 62.0, 58.5, 58.3, 54.7, 51.3, 48.7, 47.3, 47.3, 33.0, 31.8, 28.6, 28.6, 24.1, 23.1, 18.7, 18.7.

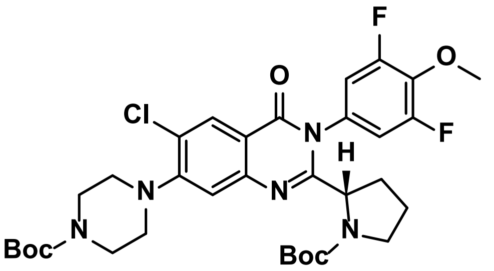

(*S*)-6e

tert-butyl (*S*)-4-(2-(1-(_tert_-butoxycarbonyl)pyrrolidin-2-yl)-6-chloro-3-(3,5-difluoro-4-methoxyphenyl)-4-oxo-3,4-dihydroquinazolin-7-yl)piperazine-1-carboxylate

(S)-6e (0.10 g, 15.0%) was prepared from **(*S*)-4d** (0.57 g, 1.00 mmol), 1-Boc-piperazine (0.090 g, 1.05 mmol), $Pd_2(dba)_3$ (0.045 g, 0.050 mmol), Xantphos (0.057 g, 0.10 mmol), cesium carbonate (0.46 g, 1.40 mmol), and anhydrous toluene (10 mL) in the same manner described for **protocol A1**. Orange solid. ^{1}H NMR (400 MHz, $CDCl_3$) δ 8.16 (d, *J* = 10.0 Hz, 1H), 7.23 – 7.11 (m, 2H), 6.89 (dt, *J* = 10.3, 2.2 Hz, 1H), 6.80 (tt, *J* = 9.7, 2.2 Hz, 1H), 4.45 (ddd, *J* = 20.9, 7.9, 4.0 Hz, 1H), 4.08 (d, *J* = 10.5 Hz, 3H), 3.75 (ddd, *J* = 10.4, 7.1, 5.5 Hz, 1H), 3.63 (t, *J* = 5.1 Hz, 5H), 3.48 (ddt, *J* = 30.3, 10.2, 6.6 Hz, 1H), 3.13 (dhept, *J* = 11.4, 4.8 Hz, 4H), 2.07 – 1.95 (m, 1H), 1.93 – 1.75 (m, 1H), 1.48 (d, *J* = 2.6 Hz, 9H), 1.41 (s, 4H), 1.27 (s, 5H). ^{19}F NMR (376 MHz, $CDCl_3$) δ -125.27 (dd, *J* = 148.9, 9.1 Hz), -126.21 (dd, *J* = 425.5, 9.0 Hz). ^{13}C NMR (101 MHz, $CDCl_3$) δ 161.1, 158.1, 157.1, 155.2, 154.6, 153.7, 147.6, 138.0, 137.7, 130.6, 128.8, 127.9, 120.3, 117.9, 116.3, 115.7, 113.7, 80.1, 62.0, 60.5, 58.4, 51.1, 47.3, 37.4, 32.9, 31.8, 28.6, 26.1.

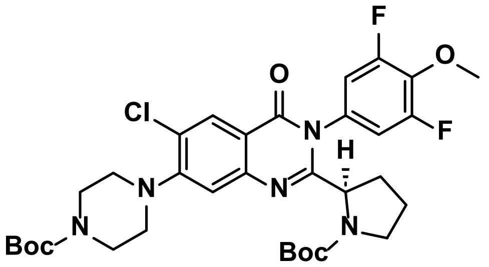

(*R*)-6e

tert-butyl (*R*)-4-(2-(1-(_tert_-butoxycarbonyl)pyrrolidin-2-yl)-6-chloro-3-(3,5-difluoro-4-methoxyphenyl)-4-oxo-3,4-dihydroquinazolin-7-yl)piperazine-1-carboxylate

(*R*)-6e (0.083 g, 23.4%) was prepared from **(*R*)-4d** (0.35 g, 0.61 mmol), 1-Boc-piperazine (0.055 g, 0.64 mmol), $Pd_2(dba)_3$ (0.028 g, 0.031 mmol), Xantphos (0.035 g, 0.061 mmol), cesium carbonate (0.28 g, 0.86 mmol), and anhydrous toluene (6.1 mL) in the same manner described for **protocol A1**. Light brown solid. ^{1}H NMR (400 MHz, $CDCl_3$) δ 8.15 (d, *J* = 8.9 Hz, 1H), 7.22 – 6.87 (m, 2H), 6.84 – 6.75 (m, 1H), 4.45 (ddd, *J* = 21.1, 7.8, 4.0 Hz, 1H), 4.08 (d, *J* = 10.4 Hz, 3H), 3.81 – 3.62 (m, 1H), 3.49 (ddt, *J* = 31.9, 10.1, 6.5 Hz, 1H), 3.25 – 3.02 (m, 10H), 2.29 – 1.75 (m, 3H), 1.34 (d, *J* = 56.0 Hz, 9H). ^{13}C NMR (101 MHz, $CDCl_3$) δ 161.2, 161.0, 158.1, 157.9, 157.1, 155.8, 155.4, 154.6, 154.4, 153.8, 147.7, 147.5, 137.8, 130.7, 130.4, 130.3, 130.2, 128.8, 128.7, 127.9, 127.7, 117.6, 116.0, 115.3, 114.4, 114.4, 114.2, 114.2, 114.1, 114.1, 113.9, 113.9, 113.8,

113.8, 113.6, 113.6, 113.5, 113.4, 113.4, 80.0, 79.7, 62.0, 58.5, 58.3, 52.5, 52.4, 47.4, 47.3, 46.1, 33.0, 31.8, 28.7, 28.6, 24.1, 23.1.

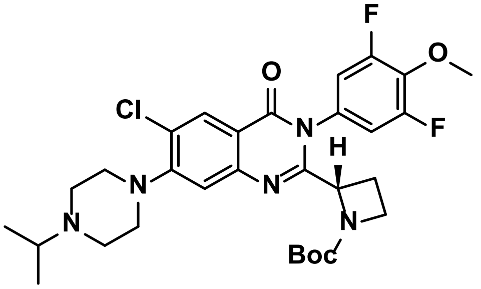

(*S*)-6f

tert-butyl (*S*)-2-(6-chloro-3-(3,5-difluoro-4-methoxyphenyl)-7-(4-isopropylpiperazin-1-yl)-4-oxo-3,4-dihydroquinazolin-2-yl)azetidine-1-carboxylate

(*S*)-6f (0.037 g, 19.0%) was prepared from $Pd_2(dba)_3$ (0.015 g, 0.016 mmol), Xantphos (0.019 g, 0.032 mmol), cesium carbonate (0.15 g, 0.45 mmol), anhydrous toluene (3.5 mL), **(*S*)-4e** (0.18 g, 0.32 mmol), and 1-isopropylpiperazine (51.2 μL, 0.36 mmol), in the same manner described for **protocol A2**. Orange solid. ^{1}H NMR (400 MHz, $CDCl_3$) δ 8.17 (s, 1H), 7.26 (s, 1H), 7.05 – 6.85 (m, 1H), 6.69 (dt, *J* = 10.2, 2.2 Hz, 1H), 4.73 (dd, *J* = 8.4, 5.3 Hz, 1H), 4.22 – 4.02 (m, 4H), 3.83 (td, *J* = 8.2, 5.6 Hz, 1H), 3.26 (d, *J* = 6.2 Hz, 4H), 2.83 – 2.71 (m, 5H), 2.38 – 2.10 (m, 2H), 1.34 (s, 9H), 1.12 (d, *J* = 6.5 Hz, 6H). ^{13}C NMR (101 MHz, $CDCl_3$) δ 160.9, 157.3, 157.2, 157.0, 155.3, 155.2, 154.8, 154.7, 154.5, 154.5, 147.5, 138.0, 137.9, 137.7, 130.1, 130.0, 129.9, 128.8, 128.0, 117.8, 113.3, 113.3, 113.1, 113.1, 80.1, 62.0, 54.8, 51.3, 48.7, 28.4, 23.3, 18.7, 18.6.

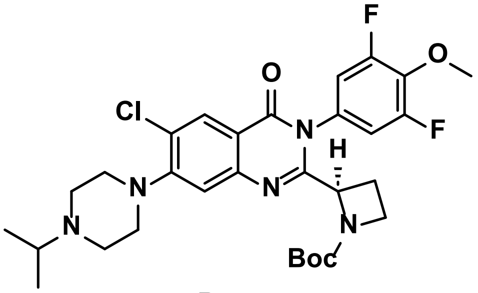

(*R*)-6f

tert-butyl (*R*)-2-(6-chloro-3-(3,5-difluoro-4-methoxyphenyl)-7-(4-isopropylpiperazin-1-yl)-4-oxo-3,4-dihydroquinazolin-2-yl)azetidine-1-carboxylate

(*R*)-6f (0.021 g, 14.4%) was prepared from $Pd_2(dba)_3$ (0.011 g, 0.012 mmol), Xantphos (0.014 g, 0.024 mmol), cesium carbonate (0.11 g, 0.34 mmol), anhydrous toluene (2.5 mL), **(*R*)-4e** (0.14 g, 0.24 mmol), and 1-isopropylpiperazine (38.4 μL, 0.27 mmol), in the same manner described for **protocol A2**. Orange solid. ^{1}H NMR (400 MHz, $CDCl_3$) δ 8.19 (s, 1H), 7.27 (s, 1H), 7.07 – 6.87 (m, 1H), 6.69 (dt, *J* = 10.3, 2.2 Hz, 1H), 4.74 (dd, *J* = 8.5, 5.3 Hz, 1H), 4.22 – 4.04 (m, 4H), 3.84 (td, *J* = 8.2, 5.6 Hz, 1H), 3.26 (s, 4H), 2.84 – 2.69 (m, 5H), 2.36 – 2.12 (m, 2H), 1.47 – 1.27 (m, 9H), 1.12 (d, *J* = 6.5 Hz, 6H). ^{13}C NMR (101 MHz, $CDCl_3$) δ 160.9, 157.3, 157.2, 157.0, 157.0,

155.4, 155.3, 154.8, 154.7, 154.5, 154.5, 147.5, 138.0, 137.9, 137.8, 130.1, 130.0, 129.9, 128.9, 128.0, 127.2, 127.1, 117.8, 113.3, 113.1, 80.2, 62.0, 60.4, 54.8, 51.3, 48.7, 28.4, 23.3, 18.7, 18.7.

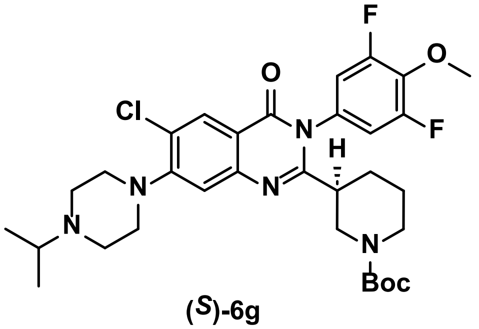

(*S*)-6g

***tert*-butyl (*S*)-3-(6-chloro-3-(3,5-difluoro-4-methoxyphenyl)-7-(4-isopropylpiperazin-1-yl)-4-oxo-3,4-dihydroquinazolin-2-yl)piperidine-1-carboxylate**

(*S*)-6g (0.065 g, 15%) was prepared from **(*S*)-4f** (0.41 g, 0.70 mmol), 1-isopropylpiperazine (0.11 mL, 0.77 mmol), $Pd_2(dba)_3$ (0.032 g, 0.035 mmol), Xantphos (0.041 g, 0.070 mmol), cesium carbonate (0.32 g, 0.98 mmol), and anhydrous toluene (7.0 mL) in the same manner described for **protocol A2**. Orange solid. ^1H NMR (400 MHz, $CDCl_3$) δ 8.05 (s, 1H), 7.12 (s, 1H), 6.85 (d, *J* = 8.5 Hz, 1H), 6.82 – 6.75 (m, 1H), 4.12 – 4.05 (m, 1H), 4.03 (s, 3H), 3.99 (s, 1H), 3.19 (t, *J* = 4.5 Hz, 4H), 3.04 (t, *J* = 12.0 Hz, 1H), 2.77 – 2.66 (m, 6H), 2.33 (dtd, *J* = 10.6, 7.4, 3.7 Hz, 1H), 1.91 – 1.80 (m, 2H), 1.64 (dt, *J* = 13.6, 3.1 Hz, 1H), 1.35 (s, 9H), 1.29 – 1.13 (m, 1H), 1.06 (d, *J* = 6.5 Hz, 6H). ^{19}F NMR (376 MHz, $CDCl_3$) δ -124.69 – -127.29 (m). ^{13}C NMR (101 MHz, $CDCl_3$) δ 160.8, 157.2, 155.1, 154.5, 147.1, 143.2, 137.7, 130.8, 130.4, 128.9, 127.6, 125.4, 117.0, 115.4, 113.3, 79.7, 61.8, 61.8, 54.5, 51.2, 48.5, 43.4, 40.8, 29.6, 28.3, 24.3, 18.6.

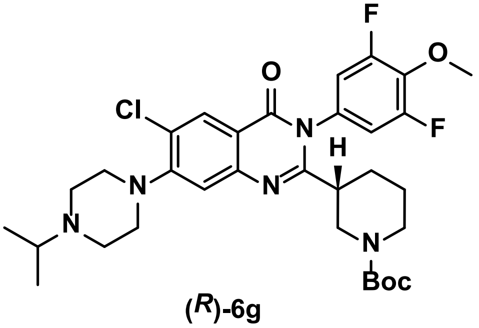

(*R*)-6g

***tert*-butyl (*R*)-3-(6-chloro-3-(3,5-difluoro-4-methoxyphenyl)-7-(4-isopropylpiperazin-1-yl)-4-oxo-3,4-dihydroquinazolin-2-yl)piperidine-1-carboxylate**

(*R*)-6g (0.13 g, 29.1%) was prepared from **(*R*)-4f** (0.40 g, 0.68 mmol), 1-isopropylpiperazine (0.1 mL, 0.718 mmol), $Pd_2(dba)_3$ (0.031 g, 0.034 mmol), Xantphos (0.040 g, 0.068 mmol), cesium carbonate (0.31 g, 0.96 mmol), and anhydrous toluene (6.8 mL) in the same manner described for **protocol A1**. Orange solid. ^1H NMR (400 MHz, $CDCl_3$) δ 8.15 (s, 1H), 7.17 (s, 1H), 6.87 (s, 1H), 6.83 – 6.78 (m, 1H), 4.08 (d, *J* = 1.2 Hz, 3H), 3.25 (t, *J* = 4.6 Hz, 4H), 3.08 (t, *J* = 11.9 Hz, 1H), 2.80 – 2.67 (m, 6H), 2.38 (dtd, *J* = 10.7, 7.5, 3.8 Hz, 1H), 1.90 (t, *J* = 5.5 Hz, 2H), 1.68 (dt, *J* =

13.6, 3.1 Hz, 1H), 1.40 (s, 9H), 1.32 – 1.22 (m, 1H), 1.11 (d, J = 6.5 Hz, 6H). ^{13}C NMR (101 MHz, $CDCl_3$) δ 161.0, 157.4, 157.2, 155.4, 154.7, 154.5, 147.3, 137.9, 137.8, 137.6, 130.9, 130.8, 130.7, 128.8, 127.8, 117.2, 115.5, 113.4, 113.2, 79.9, 62.0, 54.7, 51.4, 48.7, 41.0, 29.8, 28.4, 24.4, 18.7.

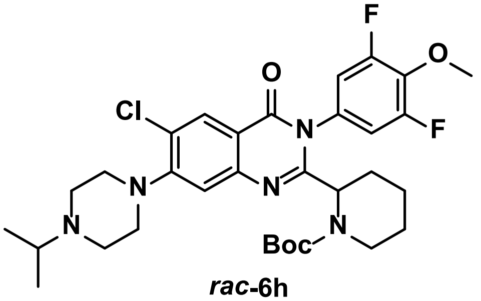

***rac*-6h**

***tert*-butyl 2-(6-chloro-3-(3,5-difluoro-4-methoxyphenyl)-7-(4-isopropylpiperazin-1-yl)-4-oxo-3,4-dihydroquinazolin-2-yl)piperidine-1-carboxylate**

***rac*-6h** (0.039 g, 12.1%) was prepared from ***rac*-4l** (0.30 g, 0.51 mmol), 1-isopropylpiperazine (77 μL, 0.54 mmol), $Pd_2(dba)_3$ (0.023 g, 0.030 mmol), Xantphos (0.030 g, 0.050 mmol), cesium carbonate (0.23 g, 0.71 mmol), and anhydrous toluene (5.0 mL) in the same manner described for **protocol A1**. Orange solid. ^{1}H NMR (400 MHz, $CDCl_3$) δ 8.16 (s, 1H), 7.17 (s, 1H), 7.11 – 7.03 (m, 1H), 6.86 (d, J = 10.3 Hz, 1H), 4.92 (dd, J = 6.4, 2.6 Hz, 1H), 4.07 (d, J = 1.2 Hz, 3H), 3.92 – 3.78 (m, 1H), 3.65 (dt, J = 14.0, 10.8 Hz, 1H), 3.26 (t, J = 4.7 Hz, 4H), 2.83 – 2.73 (m, 5H), 1.84 (d, J = 13.1 Hz, 1H), 1.72 (d, J = 12.9 Hz, 2H), 1.62 – 1.46 (m, 2H), 1.39 (s, 9H), 1.12 (d, J = 6.5 Hz, 6H). ^{13}C NMR (101 MHz, $CDCl_3$) δ 161.3, 157.1, 157.0, 156.8, 156.7, 156.0, 155.2, 154.6, 154.5, 154.3, 154.2, 146.9, 137.7, 137.5, 137.4, 130.5, 130.3, 130.2, 129.6, 128.8, 127.8, 117.6, 115.6, 114.0, 113.9, 113.8, 113.7, 80.3, 61.9, 54.7, 52.4, 51.4, 48.7, 42.5, 28.6, 27.1, 24.8, 19.0, 18.7.

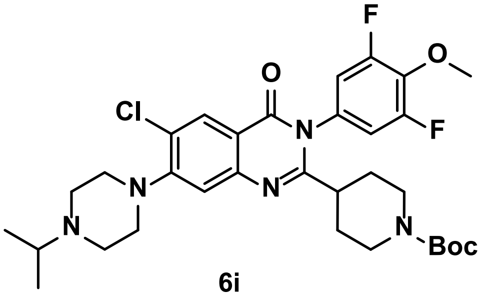

6i

***tert*-butyl 4-(6-chloro-3-(3,5-difluoro-4-methoxyphenyl)-7-(4-isopropylpiperazin-1-yl)-4-oxo-3,4-dihydroquinazolin-2-yl)piperidine-1-carboxylate**

6i (0.031 g, 18.5%) was prepared from $Pd_2(dba)_3$ (0.013 g, 0.014 mmol), Xantphos (0.016 g, 0.027 mmol), cesium carbonate (0.13 g, 0.38 mmol), anhydrous toluene (2.7 mL), **4g** (0.16 g, 0.27 mmol), and 1-isopropylpiperazine (50.0 μL, 0.35 mmol), in the same manner described for **protocol A2**. Brown solid. ^{1}H NMR (400 MHz, $CDCl_3$) δ 8.16 (s, 1H), 7.19 (s, 1H), 6.86 – 6.81

(m, 2H), 4.12 – 4.09 (m, 3H), 3.34 (s, 1H), 3.29 (s, 4H), 2.85 – 2.77 (m, 2H), 2.42 (ddt, *J* = 11.3, 7.2, 3.6 Hz, 1H), 1.91 (qd, *J* = 12.7, 4.3 Hz, 2H), 1.70 (d, *J* = 13.2 Hz, 3H), 1.45 (s, 9H), 1.30 – 1.24 (m, 4H), 1.15 (d, *J* = 6.5 Hz, 6H), 0.90 – 0.80 (m, 1H). ^{13}C NMR (101 MHz, $CDCl_3$) δ 161.2, 158.9, 154.6, 147.4, 130.9, 128.8, 127.8, 117.3, 115.5, 113.5, 113.3, 79.8, 62.1, 56.1, 51.0, 48.6, 40.8, 30.4, 29.8, 28.6, 18.5.

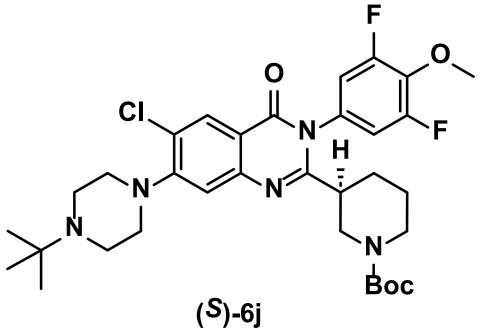

(*S*)-6j

***tert*-butyl (*S*)-3-(7-(4-(*tert*-butyl)piperazin-1-yl)-6-chloro-3-(3,5-difluoro-4-methoxyphenyl)-4-oxo-3,4-dihydroquinazolin-2-yl)piperidine-1-carboxylate**

(*S*)-6j (0.072 g, 30%) was prepared from **(*S*)-4f** (0.21 g, 0.37 mmol), 1-*tert*-butylpiperazine (0.062 g, 0.30 mmol), $Pd_2(dba)_3$ (0.017 g, 0.02 mmol), Xantphos (0.021 g, 0.04 mmol), cesium carbonate (0.17 g, 0.52 mmol), and anhydrous toluene (4.0 mL) in the same manner described for **protocol A1**. Red solid. ^{1}H NMR (400 MHz, $CDCl_3$) δ 8.14 (s, 1H), 7.16 (s, 1H), 6.87 (s, 1H), 6.84 – 6.76 (m, 1H), 4.16 – 4.09 (m, 1H), 4.08 (d, *J* = 1.4 Hz, 3H), 3.24 (t, *J* = 4.4 Hz, 4H), 3.08 (t, *J* = 12.0 Hz, 1H), 2.79 (t, *J* = 4.7 Hz, 4H), 2.73 (s, 1H), 2.37 (dtd, *J* = 10.7, 7.3, 3.7 Hz, 1H), 1.89 (q, *J* = 6.0 Hz, 2H), 1.74 – 1.63 (m, 1H), 1.40 (s, 10H), 1.13 (s, 10H). ^{19}F NMR (376 MHz, $CDCl_3$) δ -125.85 (d, *J* = 438.1 Hz). ^{13}C NMR (101 MHz, $CDCl_3$) δ 161.0, 157.3, 157.2, 155.3, 154.5, 147.3, 137.8, 130.8, 128.7, 127.8, 117.1, 115.5, 113.4, 79.9, 62.0, 62.0, 61.9, 54.0, 51.7, 45.8, 40.9, 29.8, 28.4, 26.0, 24.4.

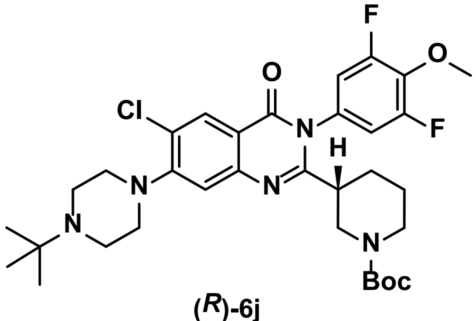

(*R*)-6j

***tert*-butyl (*R*)-3-(7-(4-(*tert*-butyl)piperazin-1-yl)-6-chloro-3-(3,5-difluoro-4-methoxyphenyl)-4-oxo-3,4-dihydroquinazolin-2-yl)piperidine-1-carboxylate**

(*R*)-6j (0.065 g, 18.1%) was prepared from **(*R*)-4f** (0.33 g, 0.56 mmol), 1-*tert*-butylpiperazine (0.084 g, 0.59 mmol), $Pd_2(dba)_3$ (0.026 g, 0.028 mmol), Xantphos (0.033 g, 0.056 mmol), cesium carbonate (0.26 g, 0.79 mmol), and anhydrous toluene (5.6 mL) in the same manner described for **protocol A1**. Light orange solid. ^{1}H NMR (400 MHz, $CDCl_3$) δ 8.16 (s, 1H), 7.17 (s, 1H), 6.94 –

6.83 (m, 1H), 6.83 – 6.76 (m, 1H), 4.12 – 4.06 (m, 5H), 3.25 (t, *J* = 4.7 Hz, 4H), 3.08 (t, *J* = 12.0 Hz, 1H), 2.85 – 2.68 (m, 5H), 2.38 (dtd, *J* = 10.7, 7.5, 3.8 Hz, 1H), 1.94 – 1.84 (m, 2H), 1.69 (dq, *J* = 13.6, 3.9 Hz, 1H), 1.40 (s, 9H), 1.33 – 1.21 (m, 1H), 1.14 (s, 9H). ^{13}C NMR (101 MHz, $CDCl_3$) δ 161.0, 157.4, 157.2, 155.3, 154.7, 154.5, 147.3, 137.9, 137.8, 137.7, 131.0, 130.9, 130.7, 128.8, 127.8, 117.1, 115.5, 113.4, 113.2, 79.9, 62.0, 54.1, 51.7, 45.8, 41.0, 29.8, 28.4, 26.0, 24.4.

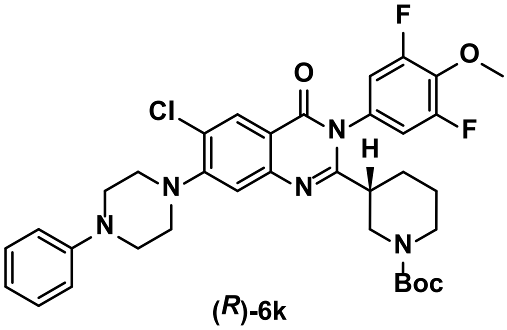

(*R*)-6k

***tert*-butyl (*R*)-3-(6-chloro-3-(3,5-difluoro-4-methoxyphenyl)-4-oxo-7-(4-phenylpiperazin-1-yl)-3,4-dihydroquinazolin-2-yl)piperidine-1-carboxylate**

(R)-6k (0.077 g, 33.8%) was prepared from **(*R*)-4f** (0.20 g, 0.34 mmol), 1-phenylpiperazine (56.5 μL, 0.36 mmol), $Pd_2(dba)_3$ (0.016 g, 0.017 mmol), Xantphos (0.020 g, 0.034 mmol), cesium carbonate (0.16 g, 0.48 mmol), and anhydrous toluene (3.5 mL) in the same manner described for **protocol A1**. Yellow solid. ^{1}H NMR (400 MHz, $CDCl_3$) δ 8.20 (d, *J* = 1.0 Hz, 1H), 7.34 – 7.27 (m, 2H), 7.26 (s, 1H), 7.03 – 6.98 (m, 2H), 6.91 (dd, *J* = 8.1, 6.5 Hz, 2H), 6.86 – 6.80 (m, 2H), 4.19 – 4.07 (m, 4H), 3.40 (q, *J* = 5.6 Hz, 8H), 3.10 (t, *J* = 12.0 Hz, 1H), 2.74 (t, *J* = 13.0 Hz, 1H), 2.40 (dtt, *J* = 11.1, 7.8, 3.9 Hz, 1H), 1.95 – 1.84 (m, 2H), 1.76 – 1.66 (m, 2H), 1.42 (s, 9H). ^{13}C NMR (101 MHz, $CDCl_3$) δ 161.0, 157.6, 157.2, 155.1, 154.5, 151.2, 147.3, 138.0, 137.8, 137.7, 130.8, 130.7, 130.6, 129.3, 128.8, 128.0, 120.3, 117.4, 116.5, 115.9, 113.4, 113.3, 80.0, 62.0, 51.2, 49.4, 41.0, 29.8, 28.4.

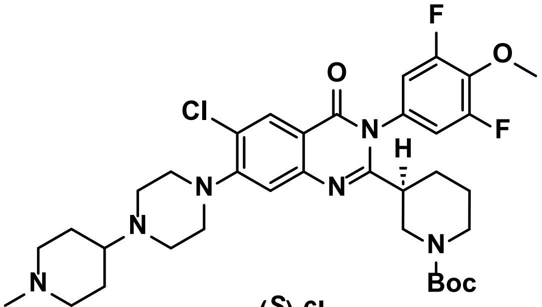

(*S*)-6l

***tert*-butyl (*S*)-3-(6-chloro-3-(3,5-difluoro-4-methoxyphenyl)-7-(4-(1-methylpiperidin-4-yl)piperazin-1-yl)-4-oxo-3,4-dihydroquinazolin-2-yl)piperidine-1-carboxylate**

(*S*)-6l (0.12 g, 34.0%) was prepared from **(*S*)-4f** (0.30 g, 0.51 mmol), 1(1-methylpiperidin-4-yl) piperazine (0.099 g, 0.54 mmol), $Pd_2(dba)_3$ (0.024 g, 0.026 mmol), Xantphos (0.030 g, 0.051 mmol), cesium carbonate (0.23 g, 0.72 mmol), and anhydrous toluene (5 mL) in the same manner described for **protocol A1**. Pale yellow solid. ^{1}H NMR (400 MHz, $CDCl_3$) δ 8.12 (s, 1H), 7.15 (s,

1H), 6.86 (s, 1H), 6.82 – 6.76 (m, 1H), 4.06 (d, *J* = 1.1 Hz, 5H), 3.21 (d, *J* = 5.0 Hz, 4H), 3.10 – 3.00 (m, 1H), 2.95 (dt, *J* = 12.3, 3.3 Hz, 2H), 2.76 (t, *J* = 4.7 Hz, 5H), 2.40 – 2.30 (m, 2H), 2.28 (s, 3H), 2.06 – 1.97 (m, 2H), 1.91 – 1.79 (m, 4H), 1.67 (qd, *J* = 9.9, 3.8 Hz, 3H), 1.38 (s, 9H), 1.32 – 1.17 (m, 1H). ^{13}C NMR (101 MHz, $CDCl_3$) δ 161.0, 157.3, 157.1, 155.2, 154.6, 154.5, 147.2, 137.9, 137.7, 137.6, 130.9, 130.7, 130.6, 128.7, 127.8, 117.2, 115.5, 113.3, 113.1, 79.9, 61.9, 61.4, 55.3, 51.4, 49.0, 46.0, 40.9, 29.7, 28.4, 28.0, 24.4.

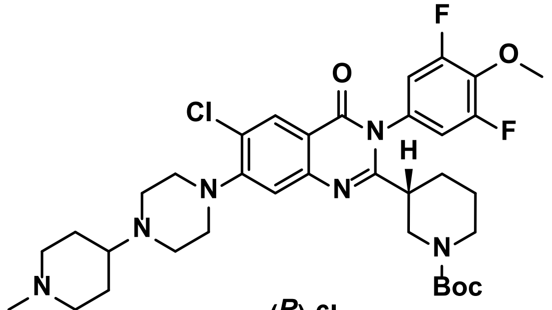

(*R*)-6l

***tert*-butyl (*R*)-3-(6-chloro-3-(3,5-difluoro-4-methoxyphenyl)-7-(4-(1-methylpiperidin-4-yl)piperazin-1-yl)-4-oxo-3,4-dihydroquinazolin-2-yl)piperidine-1-carboxylate**

(*R*)-6l (0.068 g, 20.1%) was prepared from **(*R*)-4f** (0.29 g, 0.49 mmol), 1(1-methylpiperidin-4-yl)piperazine (0.094 g, 0.51 mmol), $Pd_2(dba)_3$ (0.022 g, 0.025 mmol), Xantphos (0.028 g, 0.049 mmol), cesium carbonate (0.22 g, 0.69 mmol), and anhydrous toluene (5.0 mL) in the same manner described for **protocol A1**. Pale orange solid. ^{1}H NMR (400 MHz, $CDCl_3$) δ 8.11 (s, 1H), 7.14 (s, 1H), 6.90 – 6.82 (m, 1H), 6.81 – 6.75 (m, 1H), 4.15 – 3.95 (m, 4H), 3.25 – 3.16 (m, 4H), 3.04 (t, *J* = 11.8 Hz, 1H), 2.93 (d, *J* = 11.2 Hz, 2H), 2.79 – 2.64 (m, 5H), 2.40 – 2.22 (m, 5H), 2.00 (t, *J* = 11.5 Hz, 2H), 1.91 – 1.78 (m, 4H), 1.65 (qd, *J* = 12.4, 3.6 Hz, 3H), 1.37 (s, 9H), 1.30 – 1.18 (m, 2H). ^{13}C NMR (101 MHz, $CDCl_3$) δ 160.9, 157.3, 157.1, 155.2, 154.6, 154.4, 147.2, 137.8, 137.7, 137.5, 130.8, 130.7, 130.6, 128.6, 127.7, 117.1, 115.5, 113.3, 113.1, 79.8, 61.9, 61.4, 55.3, 51.3, 49.0, 46.0, 40.9, 29.7, 28.3, 28.0, 24.3.

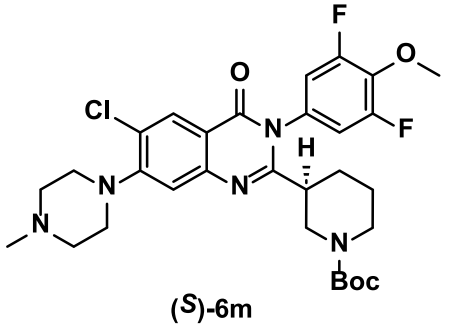

(*S*)-6m

***tert*-butyl (*S*)-3-(6-chloro-3-(3,5-difluoro-4-methoxyphenyl)-7-(4-methylpiperazin-1-yl)-4-oxo-3,4-dihydroquinazolin-2-yl)piperidine-1-carboxylate**

(*S*)-6m (0.091 g, 43.0%) was prepared from $Pd_2(dba)_3$ (0.016 g, 0.018 mmol), Xantphos (0.020 g, 0.035 mmol), cesium carbonate (0.16 g, 0.49 mmol), anhydrous toluene (3.5 mL), **(*S*)-4f** (0.21 g, 0.35 mmol), and 1-methylpiperazine (42.7 μL, 0.39 mmol), in the same manner described for

protocol A2. Light orange solid. ^{1}H NMR (400 MHz, $CDCl_3$) δ 8.18 (s, 1H), 7.20 (s, 1H), 6.89 (s, 1H), 6.84 – 6.78 (m, 1H), 4.09 (d, *J* = 1.2 Hz, 3H), 3.25 (s, 4H), 3.08 (t, *J* = 11.9 Hz, 1H), 2.79 – 2.60 (m, 5H), 2.39 (s, 4H), 1.95 – 1.83 (m, 3H), 1.73 – 1.65 (m, 1H), 1.41 (s, 9H), 1.34 – 1.22 (m, 2H). ^{13}C NMR (101 MHz, $CDCl_3$) δ 161.1, 157.5, 155.3, 154.5, 147.3, 137.8, 130.8, 128.8, 128.0, 117.4, 115.7, 113.4, 113.3, 80.0, 62.0, 55.1, 51.1, 46.2, 41.0, 29.9, 28.5.

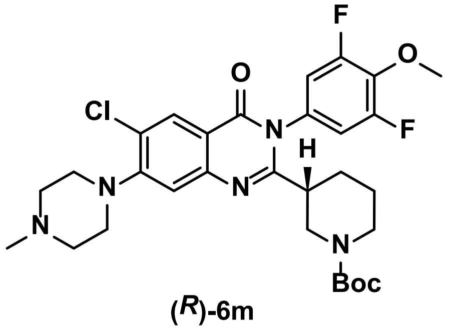

(*R*)-6m

***tert*-butyl (*R*)-3-(6-chloro-3-(3,5-difluoro-4-methoxyphenyl)-7-(4-methylpiperazin-1-yl)-4-oxo-3,4-dihydroquinazolin-2-yl)piperidine-1-carboxylate**

(*R*)-6m (0.11 g, 21.0%) was prepared from **(*R*)-4f** (0.50 g, 0.86 mmol), 1-methylpiperazine (99.6 μL, 0.90 mmol), $Pd_2(dba)_3$ (0.039 g, 0.043 mmol), Xantphos (0.050 g, 0.085 mmol), cesium carbonate (0.39 g, 1.20 mmol), and anhydrous toluene (8.6 mL) in the same manner described for **protocol A1**. Pale orange solid. ^{1}H NMR (400 MHz, $CDCl_3$) δ 8.17 – 8.14 (m, 1H), 7.19 (s, 1H), 6.88 (s, 1H), 6.84 – 6.77 (m, 1H), 4.16 – 4.01 (m, 5H), 3.24 (s, 4H), 3.08 (t, *J* = 12.0 Hz, 1H), 2.78 – 2.59 (m, 5H), 2.43 – 2.33 (m, 4H), 1.89 (q, *J* = 10.6 Hz, 2H), 1.68 (dt, *J* = 13.7, 3.1 Hz, 1H), 1.40 (s, 9H), 1.34 – 1.22 (m, 1H). ^{13}C NMR (101 MHz, $CDCl_3$) δ 161.0, 157.5, 157.2, 155.3, 154.7, 154.5, 147.3, 137.9, 137.8, 137.7, 130.9, 130.8, 130.7, 128.8, 127.9, 117.3, 115.6, 113.4, 113.2, 79.9, 62.0, 55.1, 51.1, 46.2, 41.0, 29.8, 28.4, 24.4.

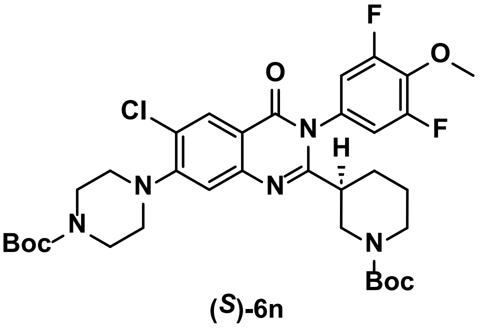

(*S*)-6n

***tert*-butyl (*S*)-4-(2-(1-(*tert*-butoxycarbonyl)piperidin-3-yl)-6-chloro-3-(3,5-difluoro-4-methoxyphenyl)-4-oxo-3,4-dihydroquinazolin-7-yl)piperazine-1-carboxylate**

(*S*)-6n (0.057 g, 22%) was prepared from **(*S*)-4f** (0.22 g, 0.38 mmol), *N*-Boc-piperazine (0.074 g, 0.42 mmol), $Pd_2(dba)_3$ (0.017 g, 0.020 mmol), Xantphos (0.022 g, 0.040 mmol), cesium carbonate (0.12 g, 0.53 mmol), and anhydrous toluene (4.0 mL) in the same manner described for **protocol A1**. Orange solid. ^{1}H NMR (400 MHz, $CDCl_3$) δ 8.16 (s, 1H), 7.17 (s, 1H), 6.88 (s, 1H), 6.85 – 6.77 (m, 1H), 4.13 (s, 1H), 4.08 (s, 3H), 3.99 (s, 1H), 3.67 – 3.60 (m, 3H), 3.13 (t, *J* = 4.9 Hz, 4H),

3.06 (s, 1H), 2.72 (t, J = 12.9 Hz, 1H), 2.37 (ddt, J = 10.4, 6.9, 3.8 Hz, 1H), 1.88 (s, 2H), 1.68 (dt, J = 14.1, 3.2 Hz, 1H), 1.48 (s, 9H), 1.39 (s, 9H), 1.35 – 0.77 (m, 1H). ^{19}F NMR (376 MHz, $CDCl_3$) δ -125.76 (d, J = 436.8 Hz). ^{13}C NMR (101 MHz, $CDCl_3$) δ 160.9, 157.6, 157.1, 155.1, 154.9, 154.6, 154.5, 147.3, 137.9, 137.7, 130.8, 130.7, 128.8, 128.4, 128.0, 117.6, 116.0, 113.4, 113.2, 80.1, 62.0, 51.1, 43.4, 40.9, 29.8, 28.4.

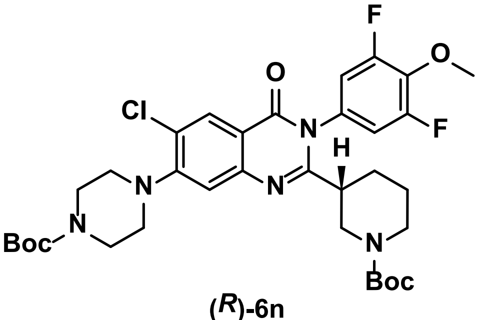

(*R*)-6n

***tert*-butyl (*R*)-4-(2-(1-(*tert*-butoxycarbonyl)piperidin-3-yl)-6-chloro-3-(3,5-difluoro-4-methoxyphenyl)-4-oxo-3,4-dihydroquinazolin-7-yl)piperazine-1-carboxylate**

(*R*)-6n (0.13 g, 36.9%) was prepared from **(*R*)-4f** (0.30 g, 0.51 mmol), *N*-Boc-piperazine (0.10 g, 0.54 mmol), $Pd_2(dba)_3$ (0.024 g, 0.026 mmol), Xantphos (0.030 g, 0.051 mmol), cesium carbonate (0.23 g, 0.72 mmol), and anhydrous toluene (5.0 mL) in the same manner described for **protocol A1**. Orange solid. ^{1}H NMR (400 MHz, $CDCl_3$) δ 8.14 (s, 1H), 7.17 (s, 1H), 6.90 – 6.85 (m, 1H), 6.81 (dd, J = 8.5, 2.5 Hz, 1H), 4.16 – 4.01 (m, 4H), 3.62 (dd, J = 6.3, 3.7 Hz, 4H), 3.17 – 2.99 (m, 5H), 2.80 – 2.64 (m, 2H), 2.42 – 2.31 (m, 1H), 1.94 – 1.83 (m, 3H), 1.67 (ddd, J = 13.9, 8.3, 5.1 Hz, 1H), 1.43 (d, J = 36.8 Hz, 18H). ^{13}C NMR (101 MHz, $CDCl_3$) δ 160.9, 157.5, 157.1, 155.0, 154.8, 154.6, 154.5, 147.2, 137.9, 137.8, 137.6, 130.7, 130.6, 130.5, 128.7, 127.9, 117.6, 116.0, 113.3, 113.2, 80.1, 79.9, 61.9, 51.1, 40.9, 29.8, 28.5, 28.4, 24.3.

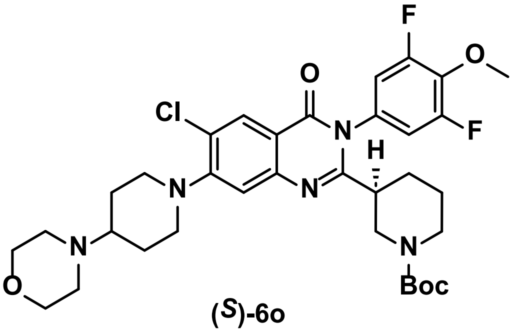

(*S*)-6o

***tert*-butyl (*S*)-3-(6-chloro-3-(3,5-difluoro-4-methoxyphenyl)-7-(4-morpholinopiperidin-1-yl)-4-oxo-3,4-dihydroquinazolin-2-yl)piperidine-1-carboxylate**

(*S*)-6o (0.064 g, 27.1%) was prepared from $Pd_2(dba)_3$ (0.016 g, 0.018 mmol), Xantphos (0.020 g, 0.035 mmol), cesium carbonate (0.16 g, 0.49 mmol), anhydrous toluene (3.5 mL), **(*S*)-4f** (0.21 g, 0.35 mmol), and 4-piperidin-4-yl morpholine (0.066 g, 0.39 mmol), in the same manner described for **protocol A2**. Pale yellow solid. ^{1}H NMR (400 MHz, $CDCl_3$) δ 8.17 (s, 1H), 7.19 (s, 1H), 6.89 (s, 1H), 6.84 – 6.78 (m, 1H), 4.18 – 3.99 (m, 4H), 3.76 (t, J = 4.6 Hz, 4H), 3.67 (d, J = 11.8 Hz,

2H), 3.08 (t, J = 11.9 Hz, 1H), 2.75 (q, J = 14.1 Hz, 3H), 2.62 (t, J = 4.7 Hz, 4H), 2.40 (ddt, J = 12.4, 8.8, 4.1 Hz, 2H), 1.99 (d, J = 12.4 Hz, 2H), 1.95 – 1.86 (m, 2H), 1.79 (qd, J = 11.9, 3.7 Hz, 2H), 1.73 – 1.54 (m, 2H), 1.41 (s, 9H), 1.34 – 1.24 (m, 1H). ^{13}C NMR (101 MHz, $CDCl_3$) δ 161.1, 160.1, 157.4, 155.7, 154.6, 147.3, 138.5, 130.9, 128.7, 128.2, 117.4, 115.5, 113.4, 113.2, 80.0, 67.5, 62.1, 61.9, 51.1, 50.0, 29.9, 28.5, 28.5.

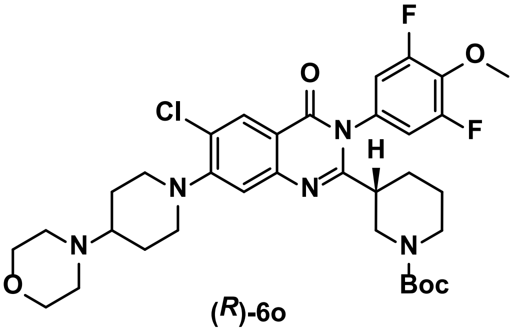

(*R*)-6o

***tert*-butyl (*R*)-3-(6-chloro-3-(3,5-difluoro-4-methoxyphenyl)-7-(4-morpholinopiperidin-1-yl)-4-oxo-3,4-dihydroquinazolin-2-yl)piperidine-1-carboxylate**

(*R*)-6o (0.16 g, 34.8%) was prepared from **(*R*)-4f** (0.40 g, 0.68 mmol), 4-piperidin-4-yl morpholine (0.12 g, 0.72 mmol), $Pd_2(dba)_3$ (0.031 g, 0.034 mmol), Xantphos (0.040 g, 0.068 mmol), cesium carbonate (0.31 g, 0.96 mmol), and anhydrous toluene (6.8 mL) in the same manner described for **protocol A1**. Orange solid. ^{1}H NMR (400 MHz, $CDCl_3$) δ 8.15 (s, 1H), 7.18 (s, 1H), 6.88 (s, 1H), 6.84 – 6.76 (m, 1H), 4.22 – 3.93 (m, 4H), 3.74 (t, J = 4.6 Hz, 4H), 3.65 (d, J = 11.8 Hz, 2H), 3.06 (t, J = 11.9 Hz, 1H), 2.80 – 2.66 (m, 3H), 2.64 – 2.58 (m, 4H), 2.44 – 2.32 (m, 2H), 2.03 – 1.93 (m, 2H), 1.93 – 1.84 (m, 2H), 1.78 (qd, J = 11.9, 3.7 Hz, 2H), 1.71 – 1.64 (m, 1H), 1.40 (s, 9H), 1.36 – 1.17 (m, 1H). ^{13}C NMR (101 MHz, $CDCl_3$) δ 161.0, 157.4, 157.2, 155.7, 154.7, 154.5, 147.2, 137.9, 137.8, 137.6, 130.9, 130.8, 130.7, 128.7, 128.1, 117.3, 115.5, 113.4, 113.2, 79.9, 67.4, 62.0, 61.8, 51.0, 51.0, 50.0, 40.9, 29.8, 28.5, 28.4, 24.4.

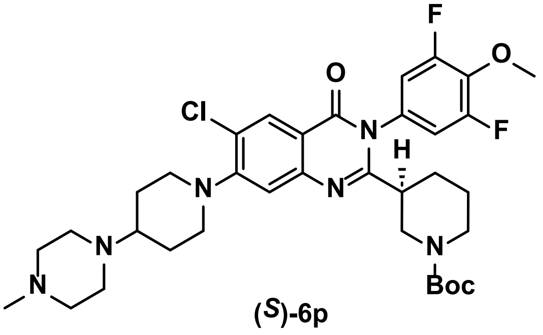

(*S*)-6p

***tert*-butyl (*S*)-3-(6-chloro-3-(3,5-difluoro-4-methoxyphenyl)-7-(4-(4-methylpiperazin-1-yl)piperidin-1-yl)-4-oxo-3,4-dihydroquinazolin-2-yl)piperidine-1-carboxylate**

(*S*)-6p (0.25 g, 52.0%) was prepared from **(*S*)-4f** (0.41 g, 0.70 mmol), 1-methyl-4-(piperidin-4-yl)piperazine (0.14 g, 0.74 mmol), $Pd_2(dba)_3$ (0.032 g, 0.035 mmol), Xantphos (0.041 g, 0.070 mmol), cesium carbonate (0.32 g, 0.098 mmol), and anhydrous toluene (7.0 mL) in the same

manner described for **protocol A1**. Red solid. ^{1}H NMR (400 MHz, $CDCl_3$) δ 8.08 (s, 1H), 7.14 (s, 1H), 6.84 (d, *J* = 9.4 Hz, 1H), 6.81 – 6.74 (m, 1H), 4.08 (s, 1H), 4.03 (s, 3H), 3.99 (s, 1H), 3.60 (d, *J* = 11.5 Hz, 2H), 3.02 (t, *J* = 11.9 Hz, 1H), 2.71 (ddd, *J* = 12.5, 9.8, 2.6 Hz, 3H), 2.62 (s, 4H), 2.45 (s, 4H), 2.35 (ddt, *J* = 18.7, 10.3, 3.4 Hz, 2H), 2.25 (s, 3H), 1.93 (dd, *J* = 12.9, 3.5 Hz, 2H), 1.89 – 1.82 (m, 2H), 1.75 (tt, *J* = 12.0, 6.0 Hz, 2H), 1.72 – 1.59 (m, 1H), 1.35 (s, 9H), 1.28 – 1.18 (m, 1H). ^{19}F NMR (376 MHz, $CDCl_3$) δ -125.87 (d, *J* = 426.1 Hz). ^{13}C NMR (101 MHz, $CDCl_3$) δ 160.8, 157.2, 157.0, 155.6, 154.5, 154.4, 147.1, 137.8, 137.5, 130.7, 128.5, 128.0, 117.2, 115.3, 113.1, 79.8, 61.9, 61.8, 61.4, 55.5, 51.1, 51.0, 49.1, 46.0, 40.8, 29.7, 28.3, 24.3.

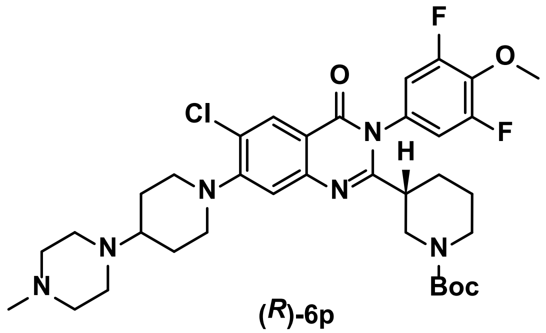

(*R*)-6p

***tert*-butyl (*R*)-3-(6-chloro-3-(3,5-difluoro-4-methoxyphenyl)-7-(4-(4-methylpiperazin-1-yl)piperidin-1-yl)-4-oxo-3,4-dihydroquinazolin-2-yl)piperidine-1-carboxylate**

(R)-6p (0.12 g, 21.0%) was prepared from **(*R*)-4f** (0.50 g, 0.86 mmol), 1-methyl-4-(piperidin-4-yl)piperazine (0.17 g, 0.90 mmol), $Pd_2(dba)_3$ (0.039 g, 0.043 mmol), Xantphos (0.050 g, 0.085 mmol), cesium carbonate (0.39 g, 1.20 mmol), and anhydrous toluene (8.6 mL) in the same manner described for **protocol A2**. Pale orange solid. ^{1}H NMR (400 MHz, $CDCl_3$) δ 8.15 (s, 1H), 7.17 (s, 1H), 6.87 (s, 1H), 6.83 – 6.77 (m, 1H), 4.18 – 3.96 (m, 4H), 3.23 (t, *J* = 4.6 Hz, 4H), 3.07 (t, *J* = 11.9 Hz, 1H), 2.96 (d, *J* = 11.0 Hz, 2H), 2.82 – 2.66 (m, 5H), 2.37 (ddt, *J* = 14.9, 11.4, 3.8 Hz, 1H), 2.30 (s, 3H), 2.01 (t, *J* = 11.5 Hz, 2H), 1.92 – 1.82 (m, 4H), 1.68 (qd, *J* = 11.6, 3.7 Hz, 3H), 1.40 (s, 9H), 1.34 – 1.21 (m, 2H). ^{13}C NMR (101 MHz, $CDCl_3$) δ 161.0, 157.4, 157.2, 155.3, 154.7, 154.5, 147.3, 137.9, 137.8, 137.7, 130.9, 130.8, 130.7, 128.8, 127.9, 117.2, 115.6, 113.4, 113.2, 79.9, 62.0, 61.6, 55.4, 51.5, 49.1, 46.1, 41.0, 29.8, 28.4, 28.2, 24.4.

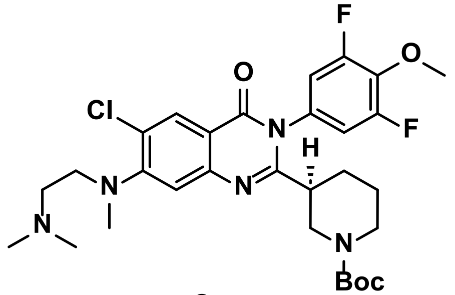

(*S*)-6q

***tert*-butyl (*S*)-3-(6-chloro-3-(3,5-difluoro-4-methoxyphenyl)-7-((2-(dimethylamino)ethyl)(methyl)amino)-4-oxo-3,4-dihydroquinazolin-2-yl)piperidine-1-carboxylate**

(*S*)-6q (0.14 g, 45%) was prepared from **(*S*)-4f** (0.30 g, 0.51 mmol), *N,N,N'*-trimethyl ethylenediamine (70.0 μL, 0.54 mmol), $Pd_2(dba)_3$ (0.024 g, 0.026 mmol), Xantphos (0.030 g, 0.051 mmol), cesium carbonate (0.23 g, 0.72 mmol), and anhydrous toluene (5 mL) in the same manner described for **protocol A1**. Orange solid. ^{1}H NMR (400 MHz, $CDCl_3$) δ 8.08 (s, 1H), 7.15 (s, 1H), 6.87 (s, 1H), 6.84 – 6.77 (m, 1H), 4.32 – 3.76 (m, 3H), 3.36 – 3.28 (m, 2H), 3.05 (t, *J* = 12.0 Hz, 1H), 2.94 (s, 3H), 2.70 (t, *J* = 12.6 Hz, 1H), 2.59 (dd, *J* = 8.2, 6.5 Hz, 2H), 2.36 (td, *J* = 9.5, 7.5, 3.7 Hz, 1H), 2.24 (s, 6H), 1.94 – 1.74 (m, 2H), 1.66 (dt, *J* = 13.5, 3.2 Hz, 1H), 1.37 (s, 10H), 1.32 – 1.12 (m, 2H). ^{19}F NMR (376 MHz, $CDCl_3$) δ -125.92 (d, *J* = 417.9 Hz). ^{13}C NMR (101 MHz, $CDCl_3$) δ 160.9, 157.3, 157.1, 155.6, 154.4, 147.0, 137.8, 137.6, 137.5, 130.8, 130.7, 128.8, 126.7, 116.8, 114.6, 113.4, 113.2, 79.8, 61.9, 57.0, 53.4, 46.2, 45.9, 40.8, 40.6, 29.7, 28.3, 24.3.

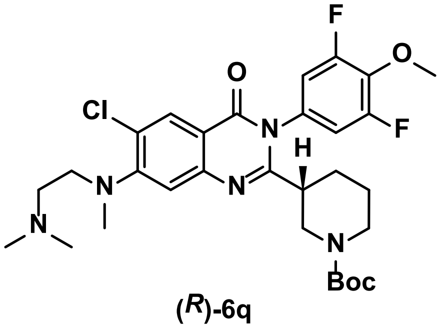

(*R*)-6q

***tert*-butyl (*R*)-3-(6-chloro-3-(3,5-difluoro-4-methoxyphenyl)-7-((2-(dimethylamino)ethyl)(methyl)amino)-4-oxo-3,4-dihydroquinazolin-2-yl)piperidine-1-carboxylate**

(*R*)-6q (0.19 g, 60.5%) was prepared from **(*R*)-4f** (0.30 g, 0.51 mmol), *N,N,N'*-trimethyl ethylenediamine (70.0 μL, 0.54 mmol), $Pd_2(dba)_3$ (0.024 g, 0.026 mmol), Xantphos (0.030 g, 0.051 mmol), cesium carbonate (0.23 g, 0.72 mmol), and anhydrous toluene (5 mL) in the same manner described for **protocol A1**. Orange solid. ^{1}H NMR (400 MHz, $CDCl_3$) δ 8.14 – 8.10 (m, 1H), 7.19 – 7.15 (m, 1H), 6.92 – 6.84 (m, 1H), 6.84 – 6.77 (m, 1H), 4.16 – 3.99 (m, 4H), 3.34 (t, *J* = 6.8 Hz, 2H), 3.06 (t, *J* = 11.9 Hz, 1H), 2.98 – 2.92 (m, 3H), 2.71 (t, *J* = 12.4 Hz, 1H), 2.61 (dt, *J* = 8.2, 4.2 Hz, 2H), 2.42 – 2.31 (m, 1H), 2.28 – 2.23 (m, 6H), 1.93 – 1.83 (m, 2H), 1.72 – 1.62 (m, 1H), 1.42 – 1.35 (m, 9H), 1.32 – 1.20 (m, 2H). ^{13}C NMR (101 MHz, $CDCl_3$) δ 160.9, 157.3, 157.1, 155.7, 154.5, 147.1, 137.8, 137.7, 137.6, 131.0, 130.8, 130.7, 128.9, 126.8, 116.9, 114.7, 113.4, 113.2, 79.9, 62.0, 57.1, 53.5, 45.9, 40.9, 40.6, 29.8, 28.4, 24.4.

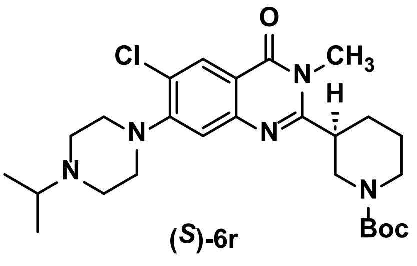

(*S*)-6r

***tert*-butyl (*S*)-3-(6-chloro-7-(4-isopropylpiperazin-1-yl)-3-methyl-4-oxo-3,4-dihydroquinazolin-2-yl)piperidine-1-carboxylate**

(***S***)**-6r** (0.085 g, 38.3%) was prepared from $Pd_2(dba)_3$ (0.020 g, 0.022 mmol), Xantphos (0.025 g, 0.044 mmol), cesium carbonate (0.20 g, 0.61 mmol), anhydrous toluene (4.5 mL), (***S***)**-4n** (0.20 g, 0.44 mmol), and 1-isopropylpiperazine (69.3 μL, 0.48 mmol), in the same manner described for **protocol A2**. Orange solid. ^{1}H NMR (400 MHz, $CDCl_3$) δ 8.16 (s, 1H), 7.10 (s, 1H), 4.42 – 4.04 (m, 2H), 3.64 (s, 3H), 3.19 (t, *J* = 4.6 Hz, 4H), 3.05 (s, 1H), 2.87 (tt, *J* = 10.9, 3.3 Hz, 1H), 2.83 – 2.62 (m, 6H), 2.07 (dt, *J* = 13.7, 3.2 Hz, 1H), 1.91 (tdd, *J* = 13.2, 11.2, 3.8 Hz, 1H), 1.80 (dt, *J* = 13.7, 3.2 Hz, 1H), 1.71 – 1.48 (m, 1H), 1.45 (s, 9H), 1.08 (d, *J* = 6.5 Hz, 6H). ^{13}C NMR (101 MHz, $CDCl_3$) δ 161.3, 157.7, 154.7, 147.1, 128.5, 127.4, 117.0, 115.6, 80.0, 54.6, 51.4, 48.7, 40.8, 30.1, 29.4, 28.5, 25.0, 18.7.

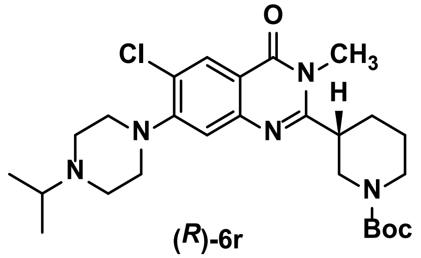

(*R*)-6r

***tert*-butyl (*R*)-3-(6-chloro-7-(4-isopropylpiperazin-1-yl)-3-methyl-4-oxo-3,4-dihydroquinazolin-2-yl)piperidine-1-carboxylate**

(***R***)**-6r** (0.088 g, 40.0%) was prepared from $Pd_2(dba)_3$ (0.020 g, 0.022 mmol), Xantphos (0.025 g, 0.044 mmol), cesium carbonate (0.20 g, 0.61 mmol), anhydrous toluene (4.5 mL), (***R***)**-4n** (0.20 g, 0.44 mmol), and 1-isopropylpiperazine (69.3 μL, 0.48 mmol), in the same manner described for **protocol A2**. Pale yellow solid. ^{1}H NMR (400 MHz, $CDCl_3$) δ 8.16 (s, 1H), 7.10 (s, 1H), 4.38 – 4.04 (m, 2H), 3.63 (s, 3H), 3.19 (t, *J* = 4.7 Hz, 4H), 3.05 (s, 1H), 2.87 (tt, *J* = 11.0, 3.4 Hz, 1H), 2.83 – 2.62 (m, 6H), 2.06 (dt, *J* = 13.5, 3.2 Hz, 1H), 1.91 (tdd, *J* = 13.3, 11.2, 3.8 Hz, 1H), 1.79 (dt, *J* = 13.6, 3.1 Hz, 1H), 1.57 (t, *J* = 12.0 Hz, 1H), 1.44 (s, 9H), 1.08 (d, *J* = 6.6 Hz, 6H). ^{13}C NMR (101 MHz, $CDCl_3$) δ 161.3, 157.7, 154.6, 147.1, 128.4, 127.4, 117.0, 115.6, 79.9, 54.6, 51.4, 48.7, 40.8, 30.1, 29.4, 28.5, 25.0, 18.7.

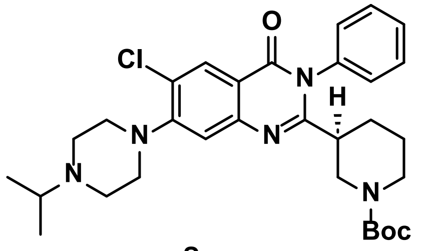

(*S*)-6s

***tert*-butyl (*S*)-3-(6-chloro-7-(4-isopropylpiperazin-1-yl)-4-oxo-3-phenyl-3,4-dihydroquinazolin-2-yl)piperidine-1-carboxylate**

(***S***)**-6s** (0.11 g, 42.4%) was prepared from $Pd_2(dba)_3$ (0.020 g, 0.022 mmol), Xantphos (0.026 g, 0.044 mmol), cesium carbonate (0.20 g, 0.62 mmol), anhydrous toluene (4.5 mL), (***S***)**-4b** (0.23 g, 0.44 mmol), and 1-isopropylpiperazine (70.2 μL, 0.49 mmol), in the same manner described for

protocol A2. Light orange solid. ^{1}H NMR (400 MHz, $CDCl_3$) δ 8.18 (s, 1H), 7.60 – 7.47 (m, 3H), 7.28 – 7.23 (m, 1H), 7.22 – 7.17 (m, 2H), 4.22 – 3.89 (m, 2H), 3.25 (t, *J* = 4.6 Hz, 4H), 3.11 (dd, *J* = 13.2, 10.8 Hz, 1H), 2.81 – 2.65 (m, 6H), 2.36 (tt, *J* = 10.8, 3.9 Hz, 1H), 1.94 – 1.77 (m, 2H), 1.62 (dt, *J* = 13.4, 3.0 Hz, 1H), 1.38 (s, 9H), 1.11 (d, *J* = 6.5 Hz, 6H). ^{13}C NMR (101 MHz, $CDCl_3$) δ 161.2, 158.2, 155.1, 154.5, 147.5, 137.0, 130.2, 129.5, 128.7, 128.3, 127.6, 117.1, 116.0, 79.6, 54.7, 51.5, 48.7, 40.9, 29.9, 28.5, 24.4, 18.7.

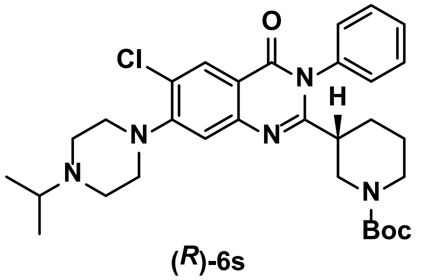

(*R*)-6s

***tert*-butyl (*R*)-3-(6-chloro-7-(4-isopropylpiperazin-1-yl)-4-oxo-3-phenyl-3,4-dihydroquinazolin-2-yl)piperidine-1-carboxylate**

(R)-6s (0.056 g, 33.2%) was prepared from $Pd_2(dba)_3$ (0.014 g, 0.015 mmol), Xantphos (0.017 g, 0.030 mmol), cesium carbonate (0.14 g, 0.42 mmol), anhydrous toluene (3.0 mL), **(*R*)-4b** (0.16 g, 0.30 mmol), and 1-isopropylpiperazine (47.3 μL, 0.48 mmol), in the same manner described for **protocol A2**. Light orange solid. ^{1}H NMR (400 MHz, $CDCl_3$) δ 8.19 (s, 1H), 7.59 – 7.47 (m, 3H), 7.26 (d, *J* = 9.7 Hz, 1H), 7.22 – 7.17 (m, 2H), 4.21 – 3.89 (m, 2H), 3.25 (t, *J* = 4.7 Hz, 4H), 3.11 (dd, *J* = 13.2, 10.7 Hz, 1H), 2.81 – 2.64 (m, 6H), 2.36 (tt, *J* = 10.8, 3.9 Hz, 1H), 1.93 – 1.76 (m, 2H), 1.62 (dt, *J* = 13.4, 3.1 Hz, 1H), 1.39 (s, 9H), 1.11 (d, *J* = 6.5 Hz, 6H). ^{13}C NMR (101 MHz, $CDCl_3$) δ 161.3, 158.2, 155.1, 154.5, 147.6, 137.0, 130.2, 129.5, 128.8, 128.3, 127.6, 117.1, 116.0, 79.7, 54.7, 51.5, 48.7, 40.9, 29.9, 28.5, 24.4, 18.7.

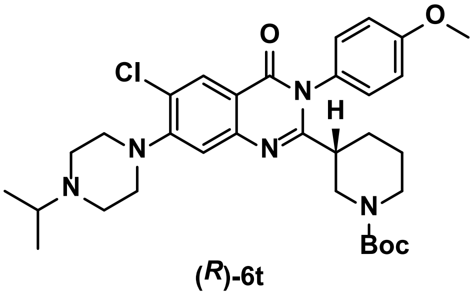

(*R*)-6t

***tert*-butyl (*R*)-3-(6-chloro-7-(4-isopropylpiperazin-1-yl)-3-(4-methoxyphenyl)-4-oxo-3,4-dihydroquinazolin-2-yl)piperidine-1-carboxylate**

(*R*)-6t (0.18 g, 40.4%) was prepared from **(*R*)-4j** (0.40 g, 0.73 mmol), 1-isopropylpiperazine (0.11 mL, 0.77 mmol), $Pd_2(dba)_3$ (0.033 g, 0.036 mmol), Xantphos (0.042 g, 0.073 mmol), cesium carbonate (0.33 g, 1.02 mmol), and anhydrous toluene (7.3 mL) in the same manner described for **protocol A1**. Brown-orange solid. ^{1}H NMR (400 MHz, $CDCl_3$) δ 8.18 (s, 1H), 7.20 – 7.12 (m, 2H), 7.10 (dd, *J* = 8.8, 2.5 Hz, 1H), 7.07 – 6.98 (m, 2H), 4.20 – 3.95 (m, 2H), 3.86 (s, 3H), 3.25 (t, *J* = 4.7 Hz, 4H), 3.10 (dd, *J* = 13.2, 10.8 Hz, 1H), 2.80 – 2.64 (m, 6H), 2.42 (tt, *J* = 10.8, 3.8 Hz,

1H), 1.94 – 1.74 (m, 2H), 1.63 (dt, *J* = 13.3, 2.9 Hz, 1H), 1.40 (s, 9H), 1.30 – 1.16 (m, 1H), 1.11 (d, *J* = 6.5 Hz, 6H). ^{13}C NMR (101 MHz, $CDCl_3$) δ 161.5, 160.1, 158.6, 155.0, 154.5, 147.5, 129.4, 129.3, 128.8, 127.5, 117.1, 116.0, 115.3, 79.6, 55.6, 54.7, 51.4, 48.7, 40.8, 29.9, 28.5, 24.5, 18.7.

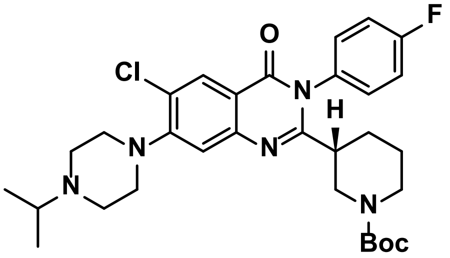

(*R*)-6u

***tert*-butyl (*R*)-3-(6-chloro-3-(4-fluorophenyl)-7-(4-isopropylpiperazin-1-yl)-4-oxo-3,4-dihydroquinazolin-2-yl)piperidine-1-carboxylate**

(*R*)-6u (0.14 g, 33.1%) was prepared from **(*R*)-4i** (0.40 g, 0.75 mmol), 1-isopropylpiperazine (0.11 mL, 0.78 mmol), $Pd_2(dba)_3$ (0.034 g, 0.037 mmol), Xantphos (0.043 g, 0.075 mmol), cesium carbonate (0.24 g, 1.04 mmol), and anhydrous toluene (7.5 mL) in the same manner described for **protocol A1**. Light brown solid. ^{1}H NMR (400 MHz, $CDCl_3$) δ 8.17 (s, 1H), 7.27 – 7.14 (m, 5H), 4.25 – 3.86 (m, 2H), 3.25 (t, *J* = 4.7 Hz, 4H), 3.10 (t, *J* = 12.0 Hz, 1H), 2.82 – 2.63 (m, 6H), 2.40 – 2.30 (m, 1H), 1.93 – 1.76 (m, 2H), 1.64 (dt, *J* = 14.0, 3.2 Hz, 1H), 1.39 (s, 9H), 1.30 – 1.14 (m, 1H), 1.11 (d, *J* = 6.5 Hz, 6H). ^{13}C NMR (101 MHz, $CDCl_3$) δ 164.1, 161.6, 161.3, 158.0, 155.2, 154.5, 147.5, 132.8, 130.2, 130.1, 128.8, 127.7, 117.4, 117.2, 115.8, 79.7, 54.7, 51.4, 48.7, 40.9, 29.8, 28.5, 24.5, 18.7.

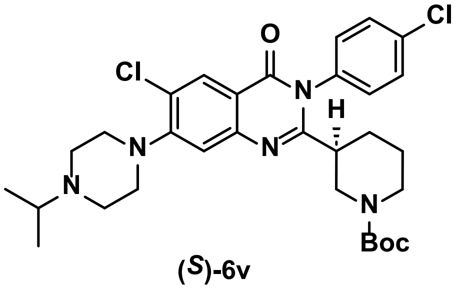

(*S*)-6v

***tert*-butyl (*S*)-3-(6-chloro-3-(4-chlorophenyl)-7-(4-isopropylpiperazin-1-yl)-4-oxo-3,4-dihydroquinazolin-2-yl)piperidine-1-carboxylate**

(*S*)-6v (0.080 g, 32.8%) was prepared from $Pd_2(dba)_3$ (0.020 g, 0.022 mmol), Xantphos (0.025 g, 0.043 mmol), cesium carbonate (0.20 g, 0.61 mmol), anhydrous toluene (4.5 mL), **(*S*)-4k** (0.23 g, 0.41 mmol), and 1-isopropylpiperazine (68.4 μL, 0.48 mmol), in the same manner described for **protocol A2**. Light orange solid. ^{1}H NMR (400 MHz, $CDCl_3$) δ 8.17 (s, 1H), 7.52 (ddd, *J* = 14.0, 8.4, 2.4 Hz, 2H), 7.26 – 7.16 (m, 2H), 7.14 (dd, *J* = 8.4, 2.6 Hz, 1H), 4.28 – 3.88 (m, 2H), 3.25 (t, *J* = 4.6 Hz, 4H), 3.09 (t, *J* = 12.0 Hz, 1H), 2.81 – 2.66 (m, 6H), 2.40 – 2.29 (m, 1H), 1.91 – 1.80 (m, 2H), 1.64 (dt, *J* = 13.6, 3.2 Hz, 1H), 1.39 (s, 9H), 1.30 – 1.13 (m, 1H), 1.11 (d, *J* = 6.5 Hz, 6H). ^{13}C NMR (101 MHz, $CDCl_3$) δ 161.16, 157.70, 155.23, 154.50, 147.44, 135.61, 135.46,

130.49, 129.75, 128.76, 127.74, 117.16, 115.73, 79.77, 77.48, 76.84, 54.66, 51.44, 48.72, 40.90, 29.80, 28.48, 24.41, 18.73.

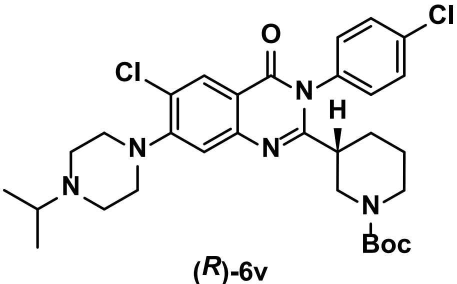

(*R*)-6v

***tert*-butyl (*R*)-3-(6-chloro-3-(4-chlorophenyl)-7-(4-isopropylpiperazin-1-yl)-4-oxo-3,4-dihydroquinazolin-2-yl)piperidine-1-carboxylate**

(*R*)-6v (0.079 g, 14.6%) was prepared from **(*R*)-4k** (0.50 g, 0.90 mmol), 1-isopropylpiperazine (0.14 mL, 0.95 mmol), $Pd_2(dba)_3$ (0.041 g, 0.045 mmol), Xantphos (0.052 g, 0.090 mmol), cesium carbonate (0.41 g, 1.27 mmol), and anhydrous toluene (9.0 mL) in the same manner described for **protocol A1**. Orange solid. ^{1}H NMR (400 MHz, $CDCl_3$) δ 8.18 (s, 1H), 7.52 (ddd, *J* = 13.5, 8.2, 2.4 Hz, 2H), 7.21 (d, *J* = 11.9 Hz, 2H), 7.15 (dd, *J* = 8.4, 2.5 Hz, 1H), 4.22 – 3.88 (m, 2H), 3.25 (t, *J* = 4.7 Hz, 4H), 3.15 – 3.04 (m, 1H), 2.82 – 2.66 (m, 6H), 2.35 (ddt, *J* = 10.5, 8.1, 4.0 Hz, 1H), 1.93 – 1.78 (m, 2H), 1.69 – 1.60 (m, 1H), 1.40 (s, 9H), 1.31 – 1.16 (m, 1H), 1.12 (d, *J* = 6.5 Hz, 6H). ^{13}C NMR (101 MHz, $CDCl_3$) δ 161.2, 157.7, 155.2, 154.5, 147.5, 135.6, 135.5, 130.5, 130.3, 129.8, 128.8, 127.8, 117.2, 115.8, 79.8, 54.7, 51.4, 48.7, 40.9, 29.8, 28.5, 24.5, 18.7.

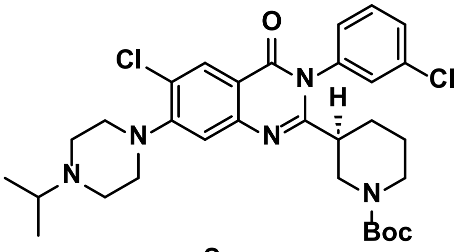

(*S*)-6w

***tert*-butyl (*S*)-3-(6-chloro-3-(3-chlorophenyl)-7-(4-isopropylpiperazin-1-yl)-4-oxo-3,4-dihydroquinazolin-2-yl)piperidine-1-carboxylate**

(*S*)-6w (0.11 g, 34.7%) was prepared from $Pd_2(dba)_3$ (0.023 g, 0.026 mmol), Xantphos (0.030 g, 0.051 mmol), cesium carbonate (0.23 g, 0.72 mmol), anhydrous toluene (5.1 mL), **(*S*)-4c** (0.28 g, 0.51 mmol), and 1-isopropylpiperazine (81.0 μL, 0.56 mmol), in the same manner described for **protocol A2**. Light orange solid. ^{1}H NMR (400 MHz, $CDCl_3$) δ 8.17 (s, 1H), 7.53 – 7.45 (m, 2H), 7.30 – 7.08 (m, 3H), 4.23 – 3.91 (m, 2H), 3.30 – 3.03 (m, 5H), 2.80 – 2.65 (m, 6H), 2.33 (tq, *J* = 10.9, 3.5 Hz, 1H), 1.92 – 1.77 (m, 2H), 1.70 – 1.59 (m, 1H), 1.39 (d, *J* = 7.2 Hz, 9H), 1.29 – 1.16 (m, 1H), 1.11 (d, *J* = 6.5 Hz, 6H). ^{13}C NMR (101 MHz, $CDCl_3$) δ 161.0, 157.6, 157.5, 155.3, 154.6, 147.4, 138.1, 130.0, 129.9, 128.7, 127.8, 126.7, 117.2, 115.7, 79.9, 79.7, 54.7, 51.4, 48.7, 40.9, 30.1, 29.7, 28.5, 28.4, 24.4, 18.7.

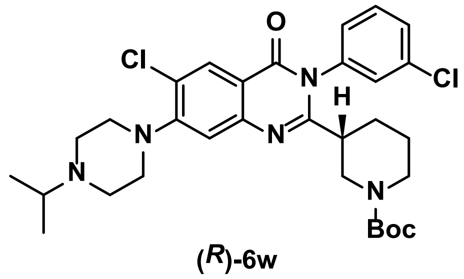

(*R*)-6w

***tert*-butyl (*R*)-3-(6-chloro-3-(3-chlorophenyl)-7-(4-isopropylpiperazin-1-yl)-4-oxo-3,4-dihydroquinazolin-2-yl)piperidine-1-carboxylate**

(*R*)-6w (0.078 g, 36.8%) was prepared from $Pd_2(dba)_3$ (0.016 g, 0.018 mmol), Xantphos (0.020 g, 0.035 mmol), cesium carbonate (0.16 g, 0.49 mmol), anhydrous toluene (3.5 mL), **(*R*)-4c** (0.19 g, 0.35 mmol), and 1-isopropylpiperazine (55.5 μL, 0.39 mmol), in the same manner described for **protocol A2**. Orange solid. ^{1}H NMR (400 MHz, $CDCl_3$) δ 8.18 (s, 1H), 7.53 – 7.45 (m, 2H), 7.31 – 7.09 (m, 3H), 4.21 – 3.93 (m, 1H), 3.31 – 3.04 (m, 5H), 2.81 – 2.67 (m, 6H), 2.34 (tq, *J* = 11.1, 3.4 Hz, 1H), 1.95 – 1.78 (m, 2H), 1.74 – 1.59 (m, 1H), 1.40 (d, *J* = 7.3 Hz, 9H), 1.31 – 1.17 (m, 1H), 1.12 (d, *J* = 6.5 Hz, 6H). ^{13}C NMR (101 MHz, $CDCl_3$) δ 161.1, 157.7, 157.6, 155.3, 147.4, 138.1, 130.0, 128.8, 127.8, 126.7, 117.2, 115.7, 79.9, 79.7, 77.5, 76.8, 54.7, 51.5, 48.7, 41.0, 30.1, 29.8, 28.5, 28.5, 24.4, 18.7.

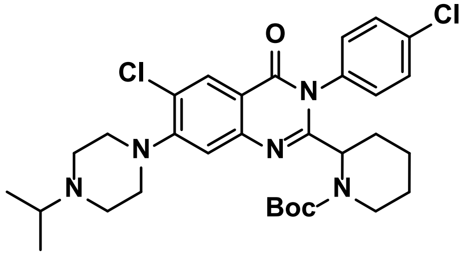

***rac*-6x**

***tert*-butyl 2-(6-chloro-3-(4-chlorophenyl)-7-(4-isopropylpiperazin-1-yl)-4-oxo-3,4-dihydroquinazolin-2-yl)piperidine-1-carboxylate**

***rac*-6x** (0.069 g, 21%) was prepared from ***rac*-4m** (0.30 g, 0.54 mmol), 1-isopropylpiperazine (81.0 μL, 0.57 mmol), $Pd_2(dba)_3$ (0.025 g, 0.028 mmol), Xantphos (0.031 g, 0.054 mmol), cesium carbonate (0.25 g, 0.75 mmol), and anhydrous toluene (5.5 mL) in the same manner described for **protocol A1**. Orange solid. ^{1}H NMR (400 MHz, $CDCl_3$) δ 8.14 (s, 1H), 7.49 – 7.42 (m, 2H), 7.42 – 7.35 (m, 1H), 7.23 (s, 1H), 7.17 (s, 1H), 3.85 (d, *J* = 13.0 Hz, 1H), 3.68 (td, *J* = 12.6, 3.4 Hz, 1H), 3.25 (t, *J* = 4.7 Hz, 3H), 2.76 (dt, *J* = 6.5, 4.0 Hz, 4H), 1.75 – 1.66 (m, 2H), 1.52 – 1.41 (m, 3H), 1.38 (s, 9H), 1.14 – 1.01 (m, 6H). ^{13}C NMR (101 MHz, $CDCl_3$) δ 161.1, 156.0, 155.1, 146.9, 136.7, 134.8, 130.1, 129.5, 128.7, 127.6, 123.5, 121.9, 119.7, 117.5, 115.7, 80.1, 54.6, 52.8, 52.3, 51.3, 51.0, 48.6, 42.4, 28.5, 26.9, 24.7, 18.8, 18.6.

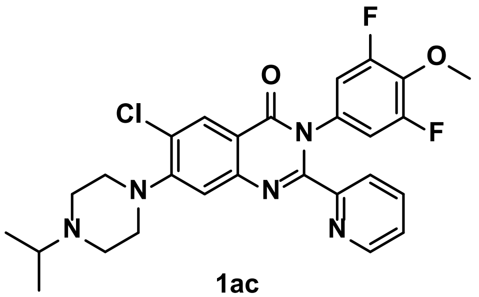

1ac

6-chloro-3-(3,5-difluoro-4-methoxyphenyl)-7-(4-isopropylpiperazin-1-yl)-2-(pyridin-2-yl)quinazolin-4(3*H*)-one

1ac (0.12 g, 54.6%) was prepared from **4o** (0.20 g, 0.42 mmol), 1-isopropylpiperazine (62.8 μL, 0.44 mmol), $Pd_2(dba)_3$ (0.019 g, 0.021 mmol), Xantphos (0.024 g, 0.042 mmol), cesium carbonate (0.19 g, 0.59 mmol), and anhydrous toluene (4.0 mL) in the same manner described for **protocol A1**. Light orange solid. ^{1}H NMR (400 MHz, $CDCl_3$) δ 8.40 (ddd, *J* = 4.9, 1.8, 0.9 Hz, 1H), 8.26 (d, *J* = 0.6 Hz, 1H), 7.71 (td, *J* = 7.8, 1.7 Hz, 1H), 7.61 (d, *J* = 7.8 Hz, 1H), 7.34 (s, 1H), 7.21 (ddd, *J* = 7.2, 4.7, 1.1 Hz, 1H), 6.76 (d, *J* = 8.2 Hz, 2H), 3.95 (s, 3H), 3.25 (t, *J* = 4.8 Hz, 4H), 2.83 – 2.66 (m, 5H), 1.10 (d, *J* = 6.5 Hz, 6H). ^{13}C NMR (101 MHz, $CDCl_3$) δ 160.7, 156.3, 156.2, 155.5, 153.8, 153.8, 153.2, 152.8, 149.0, 147.1, 136.9, 136.8, 136.7, 136.6, 131.7, 131.5, 131.4, 129.0, 128.7, 124.3, 124.3, 117.9, 115.9, 114.1, 114.1, 114.0, 113.9, 61.9, 54.7, 51.3, 48.7, 18.7. HRMS (ESI-QTOF) m/z: $[M + H]^+$ Calcd for $C_{27}H_{27}ClF_2N_5O_2^+$: 526.1816; Observed: 526.1815.

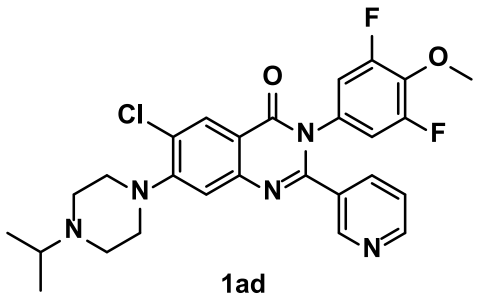

1ad

6-chloro-3-(3,5-difluoro-4-methoxyphenyl)-7-(4-isopropylpiperazin-1-yl)-2-(pyridin-3-yl)quinazolin-4(3*H*)-one

1ad (0.059 g, 42.7%) was prepared from **4p** (0.13 g, 0.26 mmol), 1-isopropylpiperazine (39.2 μL, 0.27 mmol), $Pd_2(dba)_3$ (0.012 g, 0.013 mmol), Xantphos (0.015 g, 0.026 mmol), cesium carbonate (0.12 g, 0.37 mmol), and anhydrous toluene (3 mL) in the same manner described for **protocol A1**. Pale yellow solid. ^{1}H NMR (400 MHz, $CDCl_3$) δ 8.61 (d, *J* = 2.3 Hz, 1H), 8.55 (dd, *J* = 4.9, 1.6 Hz, 1H), 8.21 (s, 1H), 7.64 (dt, *J* = 8.0, 2.0 Hz, 1H), 7.29 (s, 1H), 7.22 (dd, *J* = 8.0, 4.9 Hz, 1H), 6.77 – 6.69 (m, 2H), 3.97 (d, *J* = 1.3 Hz, 3H), 3.26 (t, *J* = 4.8 Hz, 4H), 2.75 (q, *J* = 5.9 Hz, 5H), 1.10 (d, *J* = 6.5 Hz, 6H). ^{13}C NMR (101 MHz, $CDCl_3$) δ 160.5, 156.6, 156.5, 155.6, 154.1, 154.0, 152.5, 150.7, 149.4, 147.1, 137.4, 137.3, 137.2, 136.0, 131.1, 131.0, 130.9, 130.7, 129.0, 128.7, 123.0, 117.7, 115.4, 114.3, 114.2, 114.1, 114.0, 61.9, 54.7, 51.2, 48.6, 18.6. HRMS (ESI-QTOF) m/z: $[M + H]^+$ Calcd for $C_{27}H_{27}ClF_2N_5O_2^+$: 526.1816; Observed: 526.1809.

Protocol B (refer to Scheme S2):

Boc-protected quinazolinone was dissolved in ACS grade dichloromethane and cooled to 0 °C using an ice-water bath and allowed to stir for 10 min. To this cooled solution, trifluoroacetic acid was added over 1 min, the ice bath was removed and the reaction was allowed to reach rt over 2 h. The resulting mixture was added dropwise into a flask of rapidly stirring diethyl ether (200 mL). The resulting precipitate was isolated via vacuum filtration in a fritted funnel and rinsed with diethyl ether (3 x 25 mL). The collected TFA salt product was further dried *in vacuo*.

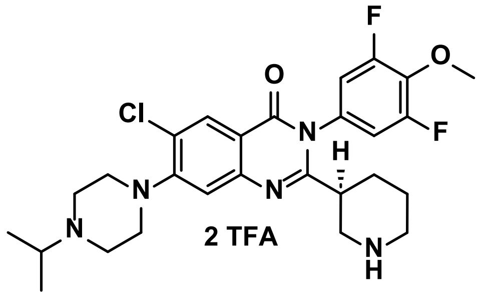

(*S*)-7g

(*S*)-6-chloro-3-(3,5-difluoro-4-methoxyphenyl)-7-(4-isopropylpiperazin-1-yl)-2-(piperidin-3-yl)quinazolin-4(3*H*)-one

(*S*)-7g (0.17 g, 88.0% yield) was prepared from **(*S*)-6g** (0.17 g, 0.26 mmol), dichloromethane (2.6 mL), and trifluoroacetic acid (1.1 mL, 7.84 mmol) in the same manner described for **protocol B**. White solid. 2 equivalents TFA by ^{19}F NMR. ^{1}H NMR (400 MHz, MeOD) δ 8.14 (s, 1H), 7.57 (s, 1H), 7.26 (ddt, *J* = 21.0, 10.8, 2.2 Hz, 2H), 4.08 (d, *J* = 1.1 Hz, 3H), 3.87 – 3.77 (m, 2H), 3.72 – 3.65 (m, 2H), 3.62 (ddd, *J* = 15.6, 13.1, 7.0 Hz, 2H), 3.40 (ddt, *J* = 12.0, 8.6, 4.5 Hz, 3H), 3.31 – 3.13 (m, 4H), 3.06 (tt, *J* = 7.4, 3.8 Hz, 1H), 2.05 – 1.96 (m, 1H), 1.96 – 1.81 (m, 2H), 1.64 (tdd, *J* = 14.7, 7.6, 3.7 Hz, 1H), 1.46 (d, *J* = 6.6 Hz, 6H). ^{19}F NMR (376 MHz, MeOD) δ -76.87, -127.60 (d, *J* = 8.9 Hz), -128.08 (d, *J* = 9.1 Hz). ^{13}C NMR (101 MHz, MeOD) δ 162.2, 157.9, 155.5, 153.9, 147.4, 138.7, 131.2, 128.8, 128.4, 118.6, 117.5, 115.9, 114.8, 114.1, 61.9, 59.2, 49.0, 48.7, 47.9, 47.7, 46.6, 44.3, 36.6, 26.9, 20.4, 16.5.

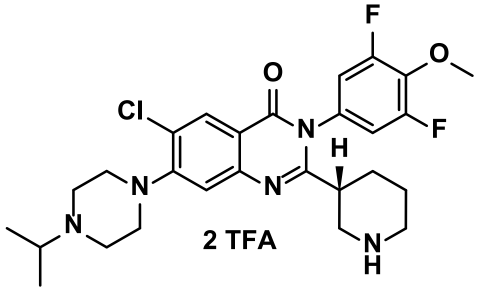

(*R*)-7g

(*R*)-6-chloro-3-(3,5-difluoro-4-methoxyphenyl)-7-(4-isopropylpiperazin-1-yl)-2-(piperidin-3-yl)quinazolin-4(3*H*)-one

(*R*)-7g (0.11 g, 70.0%) was prepared from **(*R*)-6g** (0.13 g, 0.20 mmol), dichloromethane (2.0 mL), and trifluoroacetic acid (0.46 mL, 5.96 mmol) in the same manner described for **protocol B**. Orange solid. 1.78 equivalents of TFA by ^{19}F NMR, assumed 2 equivalents. ^{1}H NMR (400 MHz, MeOD) δ 8.15 (s, 1H), 7.57 (s, 1H), 7.26 (ddt, *J* = 20.9, 10.8, 2.2 Hz, 2H), 4.08 (d, *J* = 1.1 Hz, 3H), 3.82 (d, *J* = 12.9 Hz, 2H), 3.72 – 3.56 (m, 4H), 3.40 (td, *J* = 10.2, 5.4 Hz, 3H), 3.26 (d, *J* = 12.1 Hz, 1H), 3.21 (dt, *J* = 10.1, 4.7 Hz, 2H), 3.05 (tt, *J* = 7.5, 3.9 Hz, 1H), 2.04 – 1.82 (m, 3H), 1.70 – 1.56 (m, 1H), 1.46 (d, *J* = 6.7 Hz, 6H). ^{19}F NMR (376 MHz, MeOD) δ -76.89, -127.64 (d, *J* = 8.7 Hz), -128.10 (d, *J* = 9.2 Hz). ^{13}C NMR (101 MHz, MeOD) δ 162.3, 158.6, 158.5, 158.4, 158.4, 156.1, 156.1, 155.9, 155.9, 154.6, 148.1, 139.3, 139.2, 139.1, 131.9, 131.7, 131.6, 129.4, 129.1, 119.3, 118.2, 115.4, 115.4, 115.2, 115.2, 115.0, 115.0, 114.8, 114.7, 62.5, 59.9, 49.7, 49.4, 47.2, 45.0, 37.3, 27.5, 21.1, 17.1.

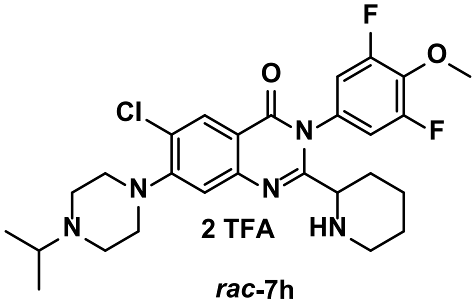

***rac*-7h**

6-chloro-3-(3,5-difluoro-4-methoxyphenyl)-7-(4-isopropylpiperazin-1-yl)-2-(piperidin-2-yl)quinazolin-4(3*H*)-one

***rac*-7h** (0.027 g, 65.8%) was prepared from ***rac*-6h** (0.033 g, 0.054 mmol), dichloromethane (0.5 mL), and trifluoroacetic acid (0.12 mL, 1.62 mmol) in the same manner described for **protocol B**. Light brown solid. ^{1}H NMR (400 MHz, MeOD) δ 8.21 (s, 1H), 7.48 (s, 1H), 7.30 (dt, *J* = 10.9, 2.2 Hz, 1H), 7.21 – 7.12 (m, 2H), 6.82 – 6.74 (m, 1H), 4.11 (t, *J* = 1.2 Hz, 3H), 4.08 – 4.00 (m, 1H), 3.83 (d, *J* = 12.8 Hz, 2H), 3.72 – 3.61 (m, 3H), 3.53 – 3.46 (m, 1H), 3.45 – 3.36 (m, 2H), 3.33 – 3.21 (m, 1H), 3.15 (td, *J* = 12.6, 3.9 Hz, 1H), 2.15 (dd, *J* = 14.4, 3.3 Hz, 1H), 1.92 – 1.68 (m, 4H), 1.46 (d, *J* = 6.6 Hz, 6H), 1.40 (td, *J* = 7.4, 3.8 Hz, 1H). ^{19}F NMR (376 MHz, MeOD) δ -76.95, -126.98 (d, *J* = 9.4 Hz), -127.73 (d, *J* = 9.1 Hz). ^{13}C NMR (101 MHz, MeOD) δ 161.8, 158.6, 158.6, 158.4, 158.3, 156.2, 156.1, 155.9, 155.9, 154.8, 154.7, 147.8, 139.7, 139.6, 139.5, 130.4, 130.3, 130.2, 130.0, 129.8, 129.7, 120.5, 119.1, 118.4, 116.6, 116.2, 115.8, 115.7, 115.5, 115.5, 115.0, 115.0, 114.8, 114.8, 62.5, 59.9, 57.9, 49.3, 45.5, 30.1, 22.7, 17.1.

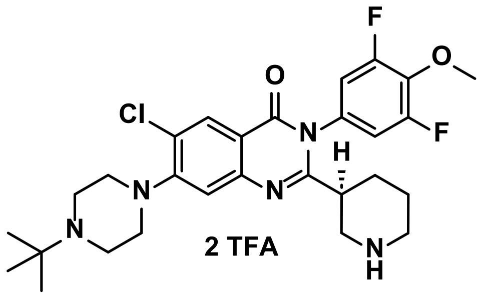

(*S*)-7j

(*S*)-7-(4-(*tert*-butyl)piperazin-1-yl)-6-chloro-3-(3,5-difluoro-4-methoxyphenyl)-2-(piperidin-3-yl)quinazolin-4(3*H*)-one

(*S*)-7j (0.045 g, 53%) was prepared from **(*S*)-6j** (0.072 g, 0.11 mmol), dichloromethane (1.1 mL), and trifluoroacetic acid (0.25 mL, 3.34 mmol) in the same manner described for **protocol B**. White solid. 2 equivalents TFA by ^{19}F NMR. ^{1}H NMR (400 MHz, MeOD) δ 8.16 (s, 1H), 7.56 (s, 1H), 7.26 (ddt, *J* = 20.9, 10.9, 2.2 Hz, 2H), 4.08 (d, *J* = 1.1 Hz, 3H), 3.89 – 3.80 (m, 2H), 3.80 (s, 1H), 3.77 (s, 1H), 3.59 (dd, *J* = 12.8, 7.3 Hz, 1H), 3.38 (td, *J* = 11.8, 10.9, 2.5 Hz, 3H), 3.29 (t, *J* = 12.6 Hz, 2H), 3.21 (dt, *J* = 8.4, 4.5 Hz, 2H), 3.05 (tt, *J* = 7.6, 3.9 Hz, 1H), 1.98 (s, 1H), 1.98 – 1.81 (m, 2H), 1.67 – 1.58 (m, 1H), 1.52 (s, 9H). ^{19}F NMR (376 MHz, MeOD) δ -76.88, -127.67 (d, *J* = 9.2 Hz), -128.11 (d, *J* = 9.0 Hz). ^{13}C NMR (101 MHz, MeOD) δ 161.7, 158.0, 157.7, 155.2, 153.9, 147.4, 138.4, 131.0, 129.8, 128.4, 119.8, 118.5, 117.6, 115.6, 114.1, 64.6, 61.9, 61.9, 47.9, 47.2, 46.6, 44.4, 36.7, 26.9, 24.0, 20.5.

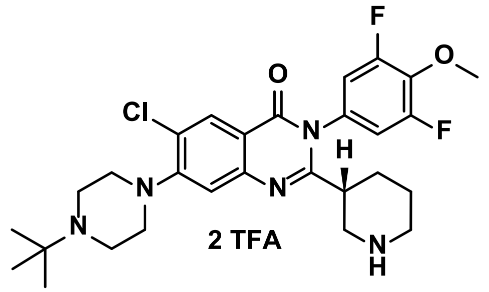

(*R*)-7j

(*R*)-7-(4-(*tert*-butyl)piperazin-1-yl)-6-chloro-3-(3,5-difluoro-4-methoxyphenyl)-2-(piperidin-3-yl)quinazolin-4(3*H*)-one

(*R*)-7j (0.044 g, 55.6%) was prepared from **(*R*)-6j** (0.066 g, 0.10 mmol), dichloromethane (1.0 mL), and trifluoroacetic acid (0.23 mL, 3.05 mmol) in the same manner described for **protocol B**. Light orange solid. 2 equivalents of TFA by ^{19}F NMR. ^{1}H NMR (400 MHz, MeOD) δ 8.15 (s, 1H), 7.56 (s, 1H), 7.26 (ddt, *J* = 20.6, 10.8, 2.2 Hz, 2H), 4.08 (d, *J* = 1.1 Hz, 3H), 3.89 – 3.74 (m, 4H), 3.59 (dd, *J* = 12.8, 7.3 Hz, 1H), 3.38 (td, *J* = 12.5, 3.0 Hz, 3H), 3.29 (t, *J* = 12.3 Hz, 2H), 3.22 – 3.17 (m, 2H), 3.05 (tt, *J* = 7.6, 3.9 Hz, 1H), 2.03 – 1.80 (m, 4H), 1.70 – 1.59 (m, 1H), 1.52 (s, 9H). ^{19}F NMR (376 MHz, MeOD) δ -76.86, -127.64 (d, *J* = 9.2 Hz), -128.10 (d, *J* = 8.9 Hz). ^{13}C NMR (101 MHz, MeOD) δ 162.8, 162.5, 162.3, 158.6, 158.5, 158.4, 158.3, 156.1, 156.1, 155.9, 155.9,

154.5, 148.1, 139.3, 139.2, 139.0, 131.9, 131.7, 131.6, 129.4, 129.0, 119.2, 118.2, 115.4, 115.4, 115.2, 115.2, 115.0, 115.0, 114.8, 114.8, 65.2, 62.5, 49.5, 47.8, 47.2, 45.0, 37.3, 27.5, 24.6, 21.1.

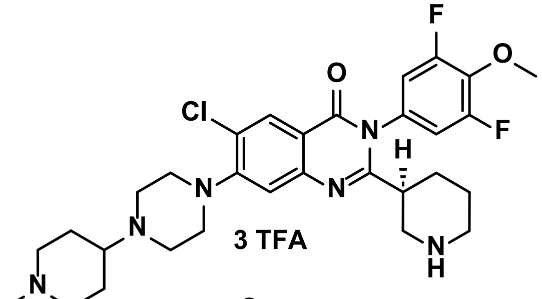

(*S*)-7l

(*S*)-6-chloro-3-(3,5-difluoro-4-methoxyphenyl)-7-(4-(1-methylpiperidin-4-yl)piperazin-1-yl)-2-(piperidin-3-yl)quinazolin-4(3*H*)-one

(*S*)-7l (0.14 g, 89.0%) was prepared from **(*S*)-6l** (0.12 g, 0.17 mmol), dichloromethane (2.0 mL), and trifluoroacetic acid (0.40 mL, 5.23 mmol) in the same manner described for **protocol B**. Light yellow solid. 3 TFA equivalents by ^{19}F NMR. ^{1}H NMR (400 MHz, MeOD) δ 8.14 (s, 1H), 7.56 (s, 1H), 7.26 (ddt, *J* = 21.3, 10.8, 2.2 Hz, 2H), 4.08 (s, 3H), 3.82 (s, 1H), 3.77 – 3.62 (m, 3H), 3.60 (dd, *J* = 12.9, 7.2 Hz, 1H), 3.39 (dd, *J* = 12.8, 3.9 Hz, 1H), 3.22 (dt, *J* = 11.0, 7.7 Hz, 4H), 3.16 (s, 1H), 3.05 (dq, *J* = 7.3, 3.7 Hz, 1H), 2.93 (s, 3H), 2.54 (d, *J* = 13.3 Hz, 2H), 2.24 (d, *J* = 13.5 Hz, 1H), 2.18 (d, *J* = 13.0 Hz, 1H), 2.05 – 1.81 (m, 3H), 1.92 (s, 1H), 1.63 (tdd, *J* = 11.9, 7.7, 4.4 Hz, 2H). ^{19}F NMR (376 MHz, MeOD) δ -76.86, -127.62 (d, *J* = 9.1 Hz), -128.09 (d, *J* = 8.9 Hz). ^{13}C NMR (101 MHz, MeOD) δ 161.7, 157.9, 157.7, 155.2, 153.8, 147.4, 138.4, 131.0, 128.4, 118.6, 117.6, 116.0, 114.8, 114.1, 66.2, 61.9, 61.8, 60.7, 53.0, 50.1, 47.9, 47.7, 46.6, 44.3, 42.9, 36.6, 26.8, 25.0, 20.4, 14.8.

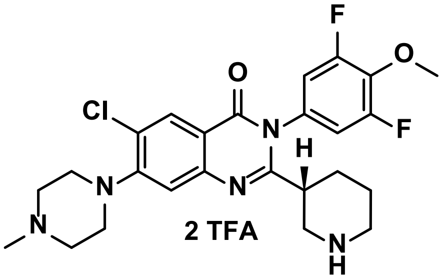

(*R*)-7m

(*R*)-6-chloro-3-(3,5-difluoro-4-methoxyphenyl)-7-(4-methylpiperazin-1-yl)-2-(piperidin-3-yl)quinazolin-4(3*H*)-one

(*R*)-7m (0.073 g, 70.9%) was prepared from **(*R*)-6m** (0.085 g, 0.14 mmol), dichloromethane (1.4 mL), and trifluoroacetic acid (0.32 mL, 4.22 mmol) in the same manner described for **protocol B**. Pale orange solid. 1.72 equivalents of TFA by ^{19}F NMR, assumed 2 equivalents. ^{1}H NMR (400 MHz, MeOD) δ 8.15 (s, 1H), 7.57 (s, 1H), 7.26 (ddt, *J* = 21.1, 10.9, 2.2 Hz, 2H), 4.08 (d, *J* = 1.1 Hz, 3H), 3.73 (dd, *J* = 30.2, 12.4 Hz, 4H), 3.59 (dd, *J* = 12.8, 7.2 Hz, 1H), 3.39 (dd, *J* = 12.8, 4.0 Hz, 3H), 3.33 – 3.14 (m, 4H), 3.02 (s, 4H), 2.03 – 1.83 (m, 2H), 1.69 – 1.57 (m, 1H). ^{19}F NMR

(376 MHz, MeOD) δ -76.91, -127.65 (d, J = 9.2 Hz), -128.11 (d, J = 9.0 Hz). ^{13}C NMR (101 MHz, MeOD) δ 162.3, 158.6, 158.5, 158.4, 158.3, 156.1, 156.1, 155.9, 155.9, 154.6, 148.0, 139.3, 139.2, 139.1, 131.8, 131.7, 131.6, 129.4, 129.1, 119.4, 118.2, 115.4, 115.4, 115.2, 115.2, 115.0, 115.0, 114.8, 114.7, 62.5, 54.8, 49.4, 47.2, 45.0, 43.6, 37.2, 27.5, 21.0.

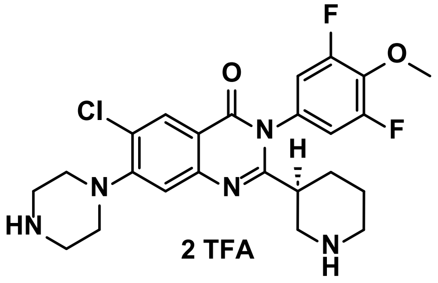

(*S*)-7n

(*S*)-6-chloro-3-(3,5-difluoro-4-methoxyphenyl)-7-(piperazin-1-yl)-2-(piperidin-3-yl)quinazolin-4(3*H*)-one

(S)-7n (0.038 g, 67.0%) was prepared from **(*S*)-6n** (0.052 g, 0.076 mmol), dichloromethane (0.7 mL), and trifluoroacetic acid (0.17 mL, 2.28 mmol) in the same manner described for **protocol B**. White solid. 2 equivalents of TFA by ^{19}F NMR. ^{1}H NMR (400 MHz, MeOD) δ 8.15 (s, 1H), 7.57 (s, 1H), 7.26 (ddt, J = 20.6, 10.8, 2.2 Hz, 2H), 4.08 (s, 3H), 3.59 (dd, J = 12.8, 7.3 Hz, 1H), 3.48 (s, 8H), 3.39 (dd, J = 12.9, 3.9 Hz, 1H), 3.21 (dt, J = 9.1, 4.6 Hz, 2H), 3.05 (tt, J = 7.3, 3.8 Hz, 1H), 2.04 – 1.81 (m, 3H), 1.64 (dt, J = 15.6, 5.1 Hz, 1H). ^{19}F NMR (376 MHz, MeOD) δ -76.89, -127.66 (d, J = 9.1 Hz), -128.11 (d, J = 8.9 Hz). ^{13}C NMR (101 MHz, MeOD) δ 162.8, 158.0, 157.7, 155.2, 154.5, 147.4, 138.4, 131.0, 128.5, 121.8, 118.7, 117.5, 116.0, 114.8, 61.9, 61.8, 47.9, 47.7, 46.6, 44.4, 44.3, 36.6, 26.9, 20.4.

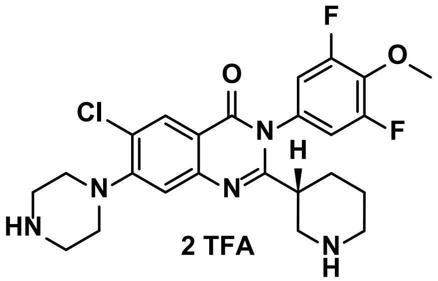

(*R*)-7n

(*R*)-6-chloro-3-(3,5-difluoro-4-methoxyphenyl)-7-(piperazin-1-yl)-2-(piperidin-3-yl)quinazolin-4(3*H*)-one

(*R*)-7n (0.109 g, 80.3%) was prepared from **(*R*)-6n** (0.13 g, 0.19 mmol), dichloromethane (2.0 mL), and trifluoroacetic acid (0.43 mL, 5.68 mmol) in the same manner described for **protocol B**. Orange solid. 2 equivalents of TFA by ^{19}F NMR. ^{1}H NMR (400 MHz, MeOD) δ 8.09 (s, 1H), 7.56 (s, 1H), 7.25 (ddt, J = 20.5, 10.8, 2.2 Hz, 2H), 4.08 (s, 3H), 3.60 (dd, J = 12.8, 7.4 Hz, 1H), 3.48 (s, 8H), 3.41 (dd, J = 12.9, 3.9 Hz, 1H), 3.27 – 3.16 (m, 2H), 3.06 (tt, J = 7.6, 3.8 Hz, 1H), 2.05 – 1.82 (m, 3H), 1.70 – 1.59 (m, 1H). ^{13}C NMR (101 MHz, MeOD) δ 163.2, 162.8, 162.3, 158.5,

158.4, 158.3, 158.2, 156.0, 156.0, 155.8, 155.8, 155.1, 148.0, 139.3, 139.1, 139.0, 131.8, 131.7, 131.5, 129.3, 129.0, 119.6, 119.3, 118.0, 116.7, 115.4, 115.4, 115.2, 115.1, 115.0, 115.0, 114.8, 114.8, 62.5, 49.2, 47.2, 44.9, 44.8, 37.3, 27.6, 21.1.

.

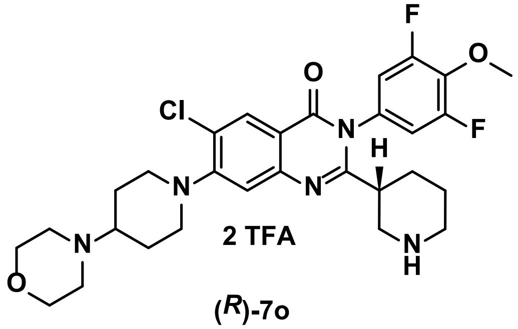

(*R*)-7o

(*R*)-6-chloro-3-(3,5-difluoro-4-methoxyphenyl)-7-(4-morpholinopiperidin-1-yl)-2-(piperidin-3-yl)quinazolin-4(3*H*)-one

(*R*)-7o (0.15 g, 75.8%) was prepared from **(*R*)-6o** (0.16 g, 0.24 mmol), dichloromethane (2.4 mL), and trifluoroacetic acid (0.55 mL, 7.15 mmol) in the same manner described for **protocol B**. Orange solid. 1.83 equivalents of TFA by ^{19}F NMR, assumed 2 equivalents. ^{1}H NMR (400 MHz, MeOD) δ 8.15 – 8.09 (m, 1H), 7.50 (d, *J* = 1.4 Hz, 1H), 7.26 (ddq, *J* = 21.1, 11.1, 2.0 Hz, 2H), 4.19 – 4.05 (m, 5H), 3.92 – 3.73 (m, 4H), 3.59 (dd, *J* = 12.8, 7.5 Hz, 3H), 3.53 – 3.36 (m, 2H), 3.30 – 3.15 (m, 4H), 3.05 (tt, *J* = 7.5, 3.9 Hz, 1H), 2.96 – 2.84 (m, 2H), 2.40 – 2.31 (m, 2H), 2.09 – 1.83 (m, 5H), 1.69 – 1.57 (m, 1H). ^{19}F NMR (376 MHz, MeOD) δ -76.86, -76.92, -127.63 – -127.77 (m), -128.14 (t, *J* = 9.8 Hz). ^{13}C NMR (101 MHz, MeOD) δ 163.0, 162.7, 162.4, 158.6, 158.5, 158.4, 158.4, 158.3, 156.1, 156.1, 156.0, 155.9, 155.9, 148.1, 139.3, 139.2, 139.0, 131.9, 131.8, 131.7, 129.3, 129.2, 119.5, 119.0, 117.3, 116.6, 115.4, 115.4, 115.2, 115.2, 115.0, 115.0, 114.8, 114.8, 65.2, 64.9, 62.6, 50.9, 50.5, 47.2, 45.0, 37.3, 27.8, 27.5, 21.1.

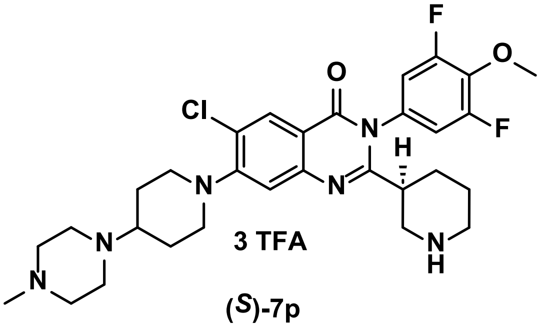

(*S*)-7p

(*S*)-6-chloro-3-(3,5-difluoro-4-methoxyphenyl)-7-(4-(4-methylpiperazin-1-yl)piperidin-1-yl)-2-(piperidin-3-yl)quinazolin-4(3*H*)-one

(*S*)-7p (0.24 g, 73.2% yield) was prepared from **(*S*)-6p** (0.25 g, 0.36 mmol), dichloromethane (3.5 mL), and trifluoroacetic acid (0.83 mL, 10.80 mmol) in the same manner described for **protocol B**. White solid. 3 equivalents TFA by ^{19}F NMR. ^{1}H NMR (400 MHz, MeOD) δ 8.07 (s, 1H), 7.47

(s, 1H), 7.24 (ddt, J = 22.2, 10.8, 2.2 Hz, 2H), 4.08 (s, 3H), 3.75 (dt, J = 12.3, 3.3 Hz, 2H), 3.65 (s, 8H), 3.59 (dd, J = 12.7, 7.4 Hz, 1H), 3.42 (qd, J = 12.6, 11.1, 2.9 Hz, 2H), 3.21 (dt, J = 8.2, 4.5 Hz, 2H), 3.04 (tt, J = 7.5, 3.9 Hz, 1H), 2.98 (s, 3H), 2.94 – 2.83 (m, 2H), 2.33 – 2.24 (m, 2H), 2.08 – 1.93 (m, 5H), 1.87 (dt, J = 9.5, 7.7 Hz, 1H), 1.68 – 1.57 (m, 1H). ^{19}F NMR (376 MHz, MeOD) δ -76.93, -127.64 (d, J = 8.9 Hz), -128.10 (d, J = 8.9 Hz). ^{13}C NMR (101 MHz, MeOD) δ 162.4, 157.6, 155.4, 147.4, 138.6, 138.5, 138.4, 131.3, 131.0, 128.6, 118.3, 116.5, 115.8, 114.2, 63.7, 61.9, 51.6, 50.4, 50.4, 47.9, 47.7, 46.7, 46.6, 44.3 (2C), 42.8, 36.6, 27.5, 26.9, 20.5.

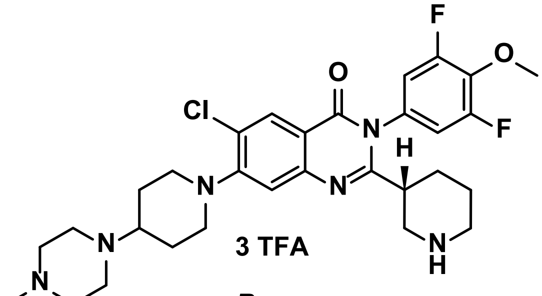

(*R*)-7p

(*R*)-6-chloro-3-(3,5-difluoro-4-methoxyphenyl)-7-(4-(4-methylpiperazin-1-yl)piperidin-1-yl)-2-(piperidin-3-yl)quinazolin-4(3*H*)-one

(*R*)-7p (0.11 g, 84.6%) was prepared from **(*R*)-6p** (0.094 g, 0.14 mmol), dichloromethane (1.4 mL), and trifluoroacetic acid (0.32 mL, 4.12 mmol) in the same manner described for **protocol B**. Pale orange solid. 3.2 equivalents of TFA by ^{19}F NMR, assumed 3 equivalents. ^{1}H NMR (400 MHz, MeOD) δ 8.14 (s, 1H), 7.54 (s, 1H), 7.23 (ddt, J = 21.3, 10.8, 2.2 Hz, 2H), 4.05 (s, 3H), 3.75 – 3.52 (m, 10H), 3.46 (q, J = 7.0 Hz, 1H), 3.36 (dd, J = 12.8, 3.9 Hz, 1H), 3.17 (qd, J = 12.5, 8.0 Hz, 4H), 3.02 (tt, J = 7.3, 3.9 Hz, 1H), 2.90 (s, 3H), 2.52 (d, J = 13.2 Hz, 2H), 2.25 – 2.10 (m, 2H), 2.02 – 1.80 (m, 3H), 1.66 – 1.55 (m, 1H), 1.15 (t, J = 7.0 Hz, 1H). ^{19}F NMR (376 MHz, MeOD) δ -77.06, -127.68 (d, J = 9.0 Hz), -128.12 (d, J = 9.0 Hz). ^{13}C NMR (101 MHz, MeOD) δ 162.7, 162.3, 158.6, 158.4, 158.4, 156.1, 156.0, 155.9, 154.5, 148.1, 139.3, 139.2, 139.1, 131.9, 131.7, 131.6, 129.5, 129.1, 119.3, 118.3, 116.4, 115.4, 115.4, 115.2, 115.2, 115.0, 115.0, 114.8, 114.7, 66.9, 62.5, 61.4, 53.7, 50.8, 49.3, 47.2, 45.0, 37.2, 27.5, 25.6, 21.0, 15.4.

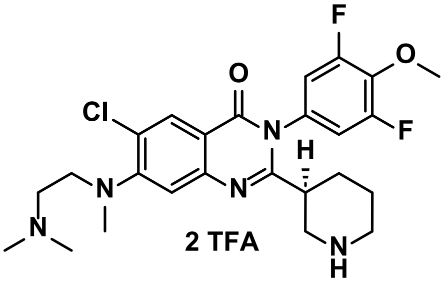

(*S*)-7q

(*S*)-6-chloro-3-(3,5-difluoro-4-methoxyphenyl)-7-((2-(dimethylamino)ethyl)(methyl)amino)-2-(piperidin-3-yl)quinazolin-4(3*H*)-one

(*S*)-7q (0.13 g, 78.1% yield) was prepared from **(*S*)-6q** (0.14 g, 0.23 mmol), dichloromethane (2.5 mL), and trifluoroacetic acid (0.53 mL, 6.93 mmol) in the same manner described for **protocol B**. Off-white solid. 2 equivalents TFA by ^{19}F NMR. ^{1}H NMR (400 MHz, MeOD) δ 8.10 (s, 1H), 7.58 (s, 1H), 7.25 (ddt, *J* = 20.1, 10.8, 2.2 Hz, 2H), 4.08 (s, 3H), 3.65 – 3.44 (m, 5H), 3.41 (dd, *J* = 12.8, 3.9 Hz, 1H), 3.21 (t, *J* = 4.5 Hz, 2H), 3.07 (ddd, *J* = 11.4, 7.9, 4.2 Hz, 1H), 2.98 (s, 9H), 1.98 (qp, *J* = 8.2, 4.5 Hz, 2H), 1.88 (s, 1H), 1.65 (qd, *J* = 12.6, 10.0, 5.3 Hz, 1H). ^{19}F NMR (376 MHz, MeOD) δ -76.81, -127.53 (d, *J* = 8.9 Hz), -128.06 (d, *J* = 8.9 Hz). ^{13}C NMR (101 MHz, MeOD) δ 161.7, 157.6, 155.2, 147.3, 138.6, 138.3, 131.0, 128.8, 128.2, 118.7, 116.7, 115.8, 114.2, 112.9, 61.9, 54.9, 50.6, 47.9, 46.6, 44.3, 43.5, 40.7, 36.6, 26.9, 20.5.

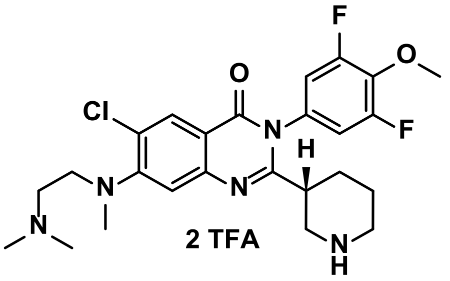

(*R*)-7q

(*R*)-6-chloro-3-(3,5-difluoro-4-methoxyphenyl)-7-((2-(dimethylamino)ethyl)(methyl)amino)-2-(piperidin-3-yl)quinazolin-4(3*H*)-one

(*R*)-7q (0.19 g, 80.2%) was prepared from **(*R*)-6q** (0.19 g, 0.31 mmol), dichloromethane (3.0 mL), and trifluoroacetic acid (0.71 mL, 9.30 mmol) in the same manner described for **protocol B**. Orange solid. 2 equivalents of TFA by ^{19}F NMR. ^{1}H NMR (400 MHz, MeOD) δ 8.12 (s, 1H), 7.59 (s, 1H), 7.25 (ddt, *J* = 19.8, 11.0, 2.2 Hz, 2H), 4.08 (d, *J* = 1.1 Hz, 3H), 3.63 – 3.50 (m, 5H), 3.40 (dd, *J* = 12.8, 3.9 Hz, 1H), 3.23 – 3.17 (m, 2H), 3.05 (dp, *J* = 7.5, 3.9 Hz, 1H), 2.98 (d, *J* = 1.3 Hz, 9H), 2.03 – 1.82 (m, 3H), 1.69 – 1.57 (m, 1H). ^{19}F NMR (376 MHz, MeOD) δ -76.80, -127.62 (d, *J* = 8.9 Hz), -128.10 (d, *J* = 8.9 Hz). ^{13}C NMR (101 MHz, MeOD) δ 162.9, 162.6, 162.3, 158.6, 158.5, 158.4, 158.3, 158.3, 156.3, 156.1, 156.0, 155.9, 155.8, 147.9, 139.3, 139.2, 139.0, 131.9, 131.8, 131.6, 129.5, 128.9, 119.6, 119.5, 117.4, 116.6, 115.4, 115.4, 115.2, 115.2, 115.0, 115.0, 114.8, 114.8, 62.5, 55.6, 51.3, 47.2, 45.0, 44.1, 41.3, 37.3, 27.6, 21.1.

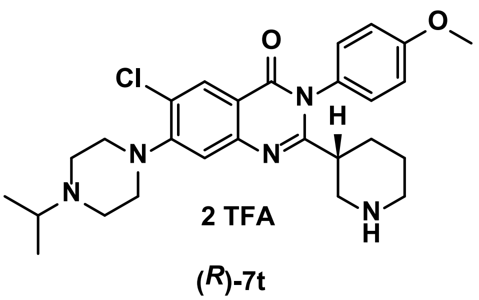

(*R*)-7t

(*R*)-6-chloro-7-(4-isopropylpiperazin-1-yl)-3-(4-methoxyphenyl)-2-(piperidin-3-yl)quinazolin-4(3*H*)-one

(*R*)-7t (0.12 g, 59.9%) was prepared from **(*R*)-6t** (0.18 g, 0.30 mmol), dichloromethane (3.0 mL), and trifluoroacetic acid (0.68 mL, 8.90 mmol) in the same manner described for **protocol B**. Brown-orange solid. 2.14 equivalents of TFA by ^{19}F NMR, assumed 2 equivalents. TFA standardization was done in 0.085 M 4-fluorobenzoic acid in DMSO-d_6. ^{1}H NMR (400 MHz, MeOD) δ 8.13 (s, 1H), 7.55 (s, 1H), 7.36 – 7.25 (m, 2H), 7.14 – 7.08 (m, 2H), 3.86 (s, 3H), 3.84 – 3.76 (m, 2H), 3.70 – 3.54 (m, 4H), 3.37 (qd, *J* = 10.7, 3.3 Hz, 3H), 3.25 (d, *J* = 12.5 Hz, 1H), 3.16 (p, *J* = 7.2 Hz, 2H), 3.04 (tt, *J* = 7.4, 4.0 Hz, 1H), 2.01 – 1.77 (m, 3H), 1.56 (tdd, *J* = 10.9, 7.2, 3.7 Hz, 1H), 1.44 (d, *J* = 6.6 Hz, 6H). ^{13}C NMR (101 MHz, MeOD) δ 163.0, 162.8, 162.7, 162.3, 162.0, 159.5, 154.4, 148.2, 131.2, 130.6, 129.8, 129.4, 128.9, 119.2, 118.4, 116.4, 116.0, 59.9, 56.1, 49.7, 47.3, 45.0, 37.3, 27.5, 21.1, 17.2.

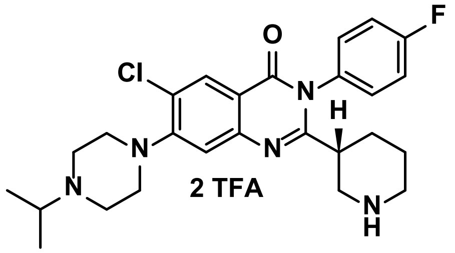

(*R*)-7u
(*R*)-6-chloro-3-(4-fluorophenyl)-7-(4-isopropylpiperazin-1-yl)-2-(piperidin-3-yl)quinazolin-4(3*H*)-one

(*R*)-7u (0.12 g, 72.8%) was prepared from **(*R*)-6u** (0.14 g, 0.24 mmol), dichloromethane (2.5 mL), and trifluoroacetic acid (0.54 mL, 7.12 mmol) in the same manner described for **protocol B**. Light brown solid. 2.3 equivalents of TFA by ^{19}F NMR, assumed 2 equivalents. ^{1}H NMR (400 MHz, MeOD) δ 8.14 (s, 1H), 7.56 (s, 1H), 7.51 – 7.41 (m, 2H), 7.36 – 7.29 (m, 2H), 3.80 (ddt, *J* = 13.8, 5.1, 2.5 Hz, 2H), 3.70 – 3.54 (m, 5H), 3.37 (td, *J* = 10.5, 6.2 Hz, 3H), 3.24 (d, *J* = 12.7 Hz, 1H), 3.21 – 3.09 (m, 2H), 2.98 (tt, *J* = 7.3, 4.0 Hz, 1H), 1.87 (ddddd, *J* = 28.6, 21.0, 13.4, 7.2, 3.5 Hz, 2H), 1.61 – 1.50 (m, 1H), 1.44 (d, *J* = 6.7 Hz, 6H). ^{19}F NMR (376 MHz, MeOD) δ -77.00, -113.13. ^{13}C NMR (101 MHz, MeOD) δ 165.8, 163.3, 162.6, 162.5, 162.1, 158.9, 154.5, 148.2, 133.6, 133.6, 132.4, 132.3, 131.9, 131.8, 129.4, 129.0, 119.3, 118.3, 118.3, 118.0, 117.9, 117.7, 59.9, 49.7, 47.2, 45.0, 37.3, 27.4, 21.1, 17.2.

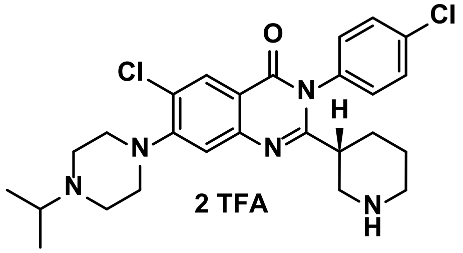

(*R*)-7v
(*R*)-6-chloro-3-(4-chlorophenyl)-7-(4-isopropylpiperazin-1-yl)-2-(piperidin-3-yl)quinazolin-4(3*H*)-one

(*R*)-7v (0.070 g, 72.5%) was prepared from **(*R*)-6v** (0.079 g, 0.13 mmol), dichloromethane (1.3 mL), and trifluoroacetic acid (0.31 mL, 3.97 mmol) in the same manner described for **protocol B**. Orange solid. 2 equivalents of TFA by ^{19}F NMR. TFA standardization was done in 0.085 M 4-fluorobenzoic acid in DMSO-d_6. ^{1}H NMR (400 MHz, MeOD) δ 8.16 (s, 1H), 7.65 – 7.60 (m, 2H), 7.58 (s, 1H), 7.45 (dddd, *J* = 19.2, 7.6, 2.6, 1.4 Hz, 2H), 3.83 (d, *J* = 12.8 Hz, 2H), 3.73 – 3.55 (m, 5H), 3.45 – 3.34 (m, 3H), 3.29 – 3.13 (m, 3H), 2.99 (tt, *J* = 7.2, 3.9 Hz, 1H), 2.01 – 1.79 (m, 2H), 1.58 (ddq, *J* = 12.4, 8.5, 5.0 Hz, 1H), 1.46 (d, *J* = 6.6 Hz, 6H). ^{13}C NMR (101 MHz, MeOD) δ 162.4, 158.7, 154.6, 148.2, 136.9, 136.3, 131.9, 131.5, 131.4, 131.1, 129.4, 129.1, 119.3, 118.3, 59.9, 49.7, 47.2, 45.0, 37.3, 27.4, 21.1, 17.2.

Protocol C (refer to Scheme S2):

TFA salt was dissolved in a mixture of ACS grade dichloromethane and methanol. Saturated potassium carbonate solution was added to the reaction mixture and stirred vigorously for 2 h. The solution was diluted with dichloromethane (80 mL) and washed 5 times with sat. potassium carbonate solution (5 x 60 mL). The organic extract was dried over anhydrous sodium sulfate, filtered, and the solvent was evaporated under reduced pressure to afford the free base product.

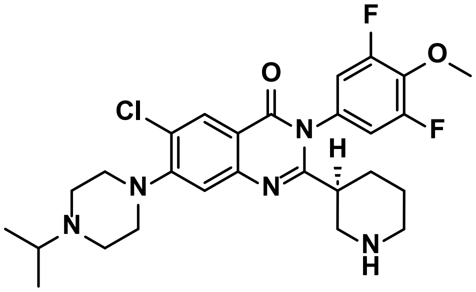

(*S*)-1g

(*S*)-6-chloro-3-(3,5-difluoro-4-methoxyphenyl)-7-(4-isopropylpiperazin-1-yl)-2-(piperidin-3-yl)quinazolin-4(3*H*)-one

(*S*)-1g (0.025 g, 98.1%) was prepared from **(*S*)-7g** (0.030 g, 0.040 mmol), methanol (2.5 mL), and saturated potassium carbonate solution (4 mL) in the same manner described for **protocol C**. Yellow solid. Less than 1% TFA contamination by ^{19}F NMR. ^{1}H NMR (400 MHz, $CDCl_3$) δ 8.15 (s, 1H), 7.19 (s, 1H), 6.85 – 6.78 (m, 2H), 4.09 (d, *J* = 1.2 Hz, 3H), 3.25 (t, *J* = 4.8 Hz, 4H), 3.09 (dd, *J* = 12.3, 4.0 Hz, 1H), 3.05 – 2.93 (m, 2H), 2.75 (t, *J* = 4.9 Hz, 4H), 2.67 (td, *J* = 12.1, 2.9 Hz, 1H), 2.46 (tt, *J* = 9.5, 4.3 Hz, 1H), 1.92 – 1.83 (m, 2H), 1.83 – 1.65 (m, 3H), 1.25 (d, *J* = 1.4 Hz, 1H), 1.11 (d, *J* = 6.5 Hz, 6H). ^{13}C NMR (101 MHz, $CDCl_3$) δ 161.2, 158.5, 157.2, 155.4, 154.7, 147.5, 137.9, 137.7, 137.6, 131.1, 131.0, 130.9, 128.8, 127.7, 117.2, 115.5, 113.6, 113.5, 113.5, 113.4, 113.4, 113.3, 113.3, 113.2, 62.1, 62.0, 62.0, 54.7, 51.5, 50.8, 48.7, 46.2, 42.1, 30.1, 25.8,

18.7. HRMS (ESI-QTOF) m/z: $[M + H]^+$ Calcd for $C_{27}H_{33}ClF_2N_5O_2^+$: 532.2285; Observed: 532.2285. Enantiopurity determined to be 97.4%.

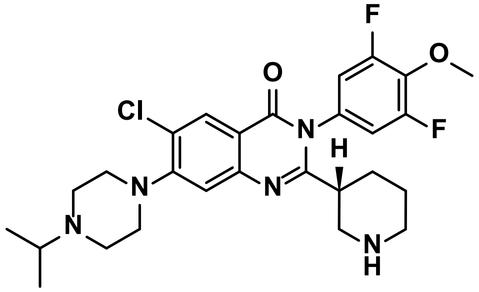

(*R*)-1g

(*R*)-6-chloro-3-(3,5-difluoro-4-methoxyphenyl)-7-(4-isopropylpiperazin-1-yl)-2-(piperidin-3-yl)quinazolin-4(3*H*)-one

(*R*)-1g (0.021 g, 98.0%) was prepared from **(*R*)-7g** (0.030 g, 0.040 mmol), methanol (2.5 mL), and saturated potassium carbonate solution (4 mL) in the same manner described for **protocol C**. Yellow solid. Less than 1% TFA contamination by ^{19}F NMR. ^{1}H NMR (400 MHz, $CDCl_3$) δ 8.15 (s, 1H), 7.20 (s, 1H), 6.85 – 6.78 (m, 2H), 4.09 (t, *J* = 1.2 Hz, 3H), 3.25 (t, *J* = 4.8 Hz, 4H), 3.09 (dd, *J* = 12.2, 4.0 Hz, 1H), 3.06 – 3.00 (m, 1H), 2.99 – 2.93 (m, 1H), 2.75 (t, *J* = 4.9 Hz, 4H), 2.67 (td, *J* = 12.1, 2.9 Hz, 1H), 2.46 (tt, *J* = 9.5, 4.3 Hz, 1H), 1.92 – 1.75 (m, 3H), 1.69 (dt, *J* = 13.4, 3.3 Hz, 1H), 1.25 (s, 1H), 1.11 (d, *J* = 6.5 Hz, 6H). ^{13}C NMR (101 MHz, $CDCl_3$) δ 161.2, 158.5, 157.2, 155.4, 154.7, 147.5, 137.9, 137.7, 137.6, 131.1, 131.0, 130.9, 128.8, 127.8, 117.2, 115.5, 113.6, 113.5, 113.5, 113.5, 113.4, 113.3, 113.3, 113.2, 62.1, 62.0, 62.0, 54.7, 51.5, 50.8, 48.7, 46.2, 42.1, 30.1, 25.8, 18.7. HRMS (ESI-QTOF) m/z: $[M + H]^+$ Calcd for $C_{27}H_{33}ClF_2N_5O_2^+$: 532.2285; Observed: 532.2277. Enantiopurity determined to be 94.7%.

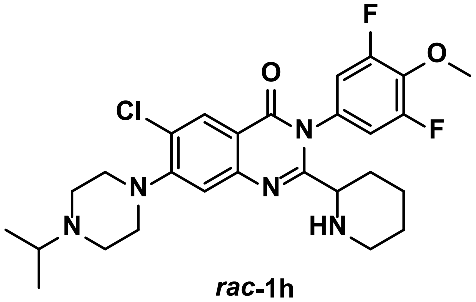

***rac*-1h**

6-chloro-3-(3,5-difluoro-4-methoxyphenyl)-7-(4-isopropylpiperazin-1-yl)-2-(piperidin-2-yl)quinazolin-4(3*H*)-one

***rac*-1h** (0.007 g, 98.8%) was prepared from ***rac*-7h** (0.010 g, 0.013 mmol), methanol (1.0 mL), and saturated potassium carbonate solution (3 mL) in the same manner described for **protocol C**. Pale yellow solid. Less than 1% TFA contamination by ^{19}F NMR. ^{1}H NMR (400 MHz, $CDCl_3$) δ 8.17 (s, 1H), 7.25 (s, 1H), 6.98 (dt, *J* = 10.6, 2.3 Hz, 1H), 6.84 (dt, *J* = 10.4, 2.3 Hz, 1H), 4.11 (d, *J* = 1.2 Hz, 3H), 3.37 – 3.32 (m, 1H), 3.26 (s, 4H), 3.16 (d, *J* = 13.7 Hz, 1H), 2.76 (s, 5H), 2.55 – 2.47 (m, 1H), 1.85 (d, *J* = 13.5 Hz, 2H), 1.79 – 1.71 (m, 1H), 1.65 – 1.51 (m, 2H), 1.13 (d, *J* = 6.5

Hz, 6H), 0.86 (dt, J = 13.0, 6.1 Hz, 2H). HRMS (ESI-QTOF) m/z: $[M + H]^+$ Calcd for $C_{27}H_{33}ClF_2N_5O_2^+$: 532.2285; Observed: 532.2279.

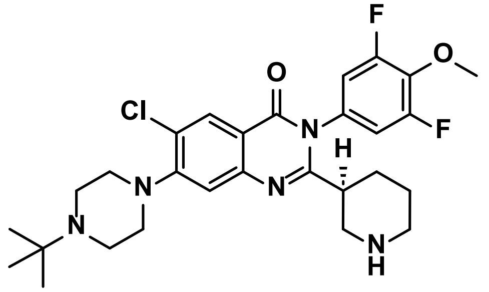

(*S*)-1j

(*S*)-7-(4-(*tert*-butyl)piperazin-1-yl)-6-chloro-3-(3,5-difluoro-4-methoxyphenyl)-2-(piperidin-3-yl)quinazolin-4(3*H*)-one

(*S*)-1j (0.004 g, 36.6%) was prepared from **(*S*)-7j** (0.017 g, 0.022 mmol), dichloromethane (0.65 mL), methanol (0.15 mL), and saturated potassium carbonate solution (2 mL) in the same manner described for **protocol C**. Pale yellow solid. Less than 1% TFA contamination. ^{1}H NMR (400 MHz, $CDCl_3$) δ 8.16 (s, 1H), 7.19 (s, 1H), 6.85 – 6.78 (m, 2H), 4.10 (d, J = 1.2 Hz, 3H), 3.25 (t, J = 4.7 Hz, 4H), 3.10 (dd, J = 12.2, 4.0 Hz, 1H), 3.06 – 3.01 (m, 1H), 3.00 – 2.94 (m, 1H), 2.80 (t, J = 4.8 Hz, 4H), 2.68 (td, J = 12.1, 2.9 Hz, 1H), 2.47 (tt, J = 9.6, 4.4 Hz, 1H), 1.88 (td, J = 10.2, 3.8 Hz, 2H), 1.69 (dt, J = 13.6, 3.2 Hz, 1H), 1.62 (s, 2H), 1.14 (s, 9H). ^{13}C NMR (101 MHz, $CDCl_3$) δ 161.2, 158.5, 157.2, 155.4, 154.7, 147.5, 137.8, 131.1, 128.8, 127.7, 117.2, 115.4, 113.6, 113.5, 113.4, 113.3, 62.0, 54.0, 51.8, 50.9, 46.3, 45.9, 42.2, 30.1, 26.1, 25.8. HRMS (ESI-QTOF) m/z: $[M + H]^+$ Calcd for $C_{28}H_{35}ClF_2N_5O_2^+$: 546.2442; Observed: 546.2446. Enantiopurity determined to be 96.3%.

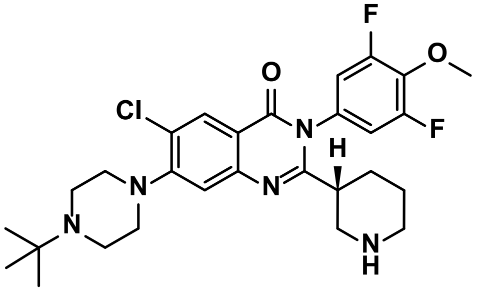

(*R*)-1j

(*R*)-7-(4-(*tert*-butyl)piperazin-1-yl)-6-chloro-3-(3,5-difluoro-4-methoxyphenyl)-2-(piperidin-3-yl)quinazolin-4(3*H*)-one

(*R*)-1j (0.014 g, 77.4%) was prepared from **(*R*)-7j** (0.026 g, 0.034 mmol), dichloromethane (0.75 mL), methanol (0.25 mL), and saturated potassium carbonate solution (2 mL) in the same manner described for **protocol C**. Pale yellow solid. Less than 1% TFA contamination. ^{1}H NMR (400 MHz, $CDCl_3$) δ 8.15 (s, 1H), 7.19 (s, 1H), 6.85 – 6.78 (m, 2H), 4.10 (d, J = 1.2 Hz, 3H), 3.25 (t, J = 4.7 Hz, 4H), 3.09 (dd, J = 12.2, 4.0 Hz, 1H), 3.05 – 3.00 (m, 1H), 3.00 – 2.93 (m, 1H), 2.80 (t, J = 4.7 Hz, 4H), 2.67 (td, J = 12.1, 2.9 Hz, 1H), 2.46 (tt, J = 9.6, 4.3 Hz, 1H), 1.88 (td, J = 10.2, 4.0

Hz, 2H), 1.74 – 1.64 (m, 2H), 1.31 (tq, *J* = 7.3, 3.8 Hz, 1H), 1.13 (s, 9H). ^{13}C NMR (101 MHz, $CDCl_3$) δ 161.2, 158.5, 157.2, 155.3, 154.7, 147.5, 137.7, 131.0, 128.8, 127.7, 117.2, 115.4, 113.6, 113.5, 113.3, 113.3, 62.0, 54.0, 51.8, 50.8, 46.3, 45.9, 42.1, 30.1, 26.1, 25.8. HRMS (ESI-QTOF) m/z: $[M + H]^+$ Calcd for $C_{28}H_{35}ClF_2N_5O_2^+$: 546.2442; Observed: 546.2438. Enantiopurity determined to be 90.5%.

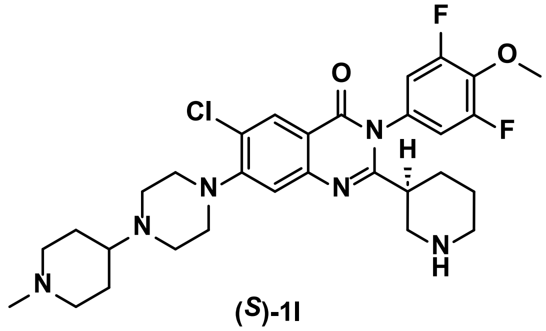

(*S*)-1l

(*S*)-6-chloro-3-(3,5-difluoro-4-methoxyphenyl)-7-(4-(1-methylpiperidin-4-yl)piperazin-1-yl)-2-(piperidin-3-yl)quinazolin-4(3*H*)-one

(*S*)-1l (0.033 g, 86.8%) was prepared from **(*S*)-7l** (0.060 g, 0.065 mmol), methanol (1 mL), and saturated potassium carbonate solution (3 mL) in the same manner described for **protocol C**. Light yellow solid. Less than 1% TFA contamination. ^{1}H NMR (400 MHz, $CDCl_3$) δ 8.16 (s, 1H), 7.20 (s, 1H), 6.86 – 6.77 (m, 2H), 4.10 (d, *J* = 1.2 Hz, 3H), 3.25 (t, *J* = 4.5 Hz, 4H), 3.10 (dd, *J* = 12.1, 4.0 Hz, 1H), 3.05 – 3.00 (m, 1H), 2.96 (t, *J* = 12.0 Hz, 4H), 2.79 (t, *J* = 4.8 Hz, 4H), 2.67 (td, *J* = 12.1, 2.9 Hz, 1H), 2.47 (tt, *J* = 9.3, 4.4 Hz, 1H), 2.37 – 2.24 (m, 4H), 2.02 – 1.93 (m, 2H), 1.92 – 1.80 (m, 4H), 1.74 – 1.60 (m, 2H), 1.36 – 1.20 (m, 2H). ^{13}C NMR (101 MHz, $CDCl_3$) δ 161.2, 158.5, 157.3, 155.3, 154.7, 147.5, 137.8, 131.0, 128.8, 127.8, 117.3, 115.5, 113.6, 113.5, 113.4, 113.3, 62.0, 61.8, 55.6, 51.5, 50.9, 49.1, 46.3, 46.3, 42.2, 30.1, 28.4, 25.8. HRMS (ESI-QTOF) m/z: $[M + H]^+$ Calcd for $C_{30}H_{38}ClF_2N_6O_2^+$: 587.2707; Observed: 587.2692. Enantiopurity determined to be 96.8%.

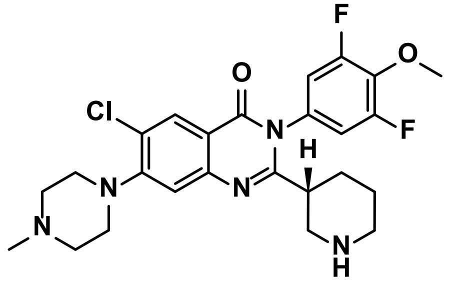

(*R*)-1m

(*R*)-6-chloro-3-(3,5-difluoro-4-methoxyphenyl)-7-(4-methylpiperazin-1-yl)-2-(piperidin-3-yl)quinazolin-4(3*H*)-one

(*R*)-1m (0.024 g, 95.7%) was prepared from **(*R*)-7m** (0.030 g, 0.041 mmol), methanol (2.5 mL), and saturated potassium carbonate solution (4 mL) in the same manner described for **protocol C**. Light brown solid. Less than 1% TFA contamination by ^{19}F NMR. ^{1}H NMR (400 MHz, $CDCl_3$) δ

8.15 (s, 1H), 7.21 (s, 1H), 6.86 – 6.77 (m, 2H), 4.09 (d, *J* = 1.2 Hz, 3H), 3.25 (s, 4H), 3.09 (dd, *J* = 12.3, 4.0 Hz, 1H), 3.05 – 2.99 (m, 1H), 2.99 – 2.92 (m, 1H), 2.70 – 2.60 (m, 5H), 2.46 (tt, *J* = 9.4, 4.3 Hz, 1H), 2.38 (s, 3H), 1.88 (q, *J* = 4.2 Hz, 2H), 1.68 (dt, *J* = 13.5, 3.2 Hz, 1H), 1.31 (ddd, *J* = 21.5, 11.3, 5.1 Hz, 2H). ^{13}C NMR (101 MHz, $CDCl_3$) δ 161.2, 158.5, 157.2, 157.2, 157.1, 155.3, 154.7, 154.7, 154.6, 147.4, 137.9, 137.7, 137.6, 131.1, 131.0, 130.9, 128.8, 127.8, 117.4, 115.6, 113.6, 113.5, 113.5, 113.4, 113.4, 113.3, 113.3, 113.2, 62.0, 55.1, 51.1, 50.8, 46.2, 46.2, 42.1, 30.1, 25.8. HRMS (ESI-QTOF) m/z: $[M + H]^+$ Calcd for $C_{25}H_{29}ClF_2N_5O_2^+$: 504.1972; Observed: 504.1971. Enantiopurity determined to be 85.4%.

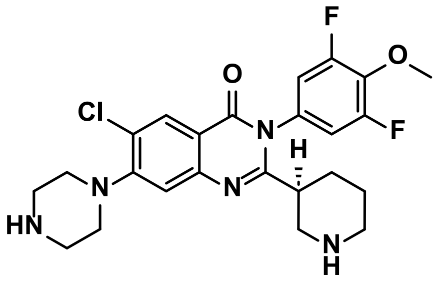

(*S*)-1n

(*S*)-6-chloro-3-(3,5-difluoro-4-methoxyphenyl)-7-(piperazin-1-yl)-2-(piperidin-3-yl)quinazolin-4(3*H*)-one

(*S*)-1n (0.006 g, 58.6%) was prepared from **(S)-7n** (0.015 g, 0.021 mmol), dichloromethane (0.6 mL), methanol (0.2 mL), and saturated potassium carbonate solution (1.5 mL) in the same manner described for **protocol C**. Pale yellow solid. Less than 1% TFA contamination. ^{1}H NMR (400 MHz, $CDCl_3$) δ 8.17 (s, 1H), 7.21 (s, 1H), 6.86 – 6.79 (m, 2H), 4.10 (d, *J* = 1.2 Hz, 3H), 3.19 (t, *J* = 4.6 Hz, 4H), 3.10 (t, *J* = 4.7 Hz, 5H), 3.06 – 2.94 (m, 2H), 2.69 (td, *J* = 12.1, 2.9 Hz, 1H), 2.49 (tt, *J* = 9.2, 4.3 Hz, 1H), 1.93 – 1.81 (m, 3H), 1.75 – 1.64 (m, 1H), 1.32 (q, *J* = 11.2 Hz, 2H). ^{13}C NMR (101 MHz, $CDCl_3$) δ 161.2, 158.5, 157.2, 155.7, 154.7, 147.4, 137.8, 131.0, 128.8, 128.0, 117.4, 115.6, 113.6, 113.5, 113.3, 113.3, 62.0, 52.6, 50.7, 46.2, 41.9, 30.0, 25.6. HRMS (ESI-QTOF) m/z: $[M + H]^+$ Calcd for $C_{24}H_{26}ClF_2N_5O_2^+$: 490.1816; Observed: 490.1809. Enantiopurity determined to be >99%.

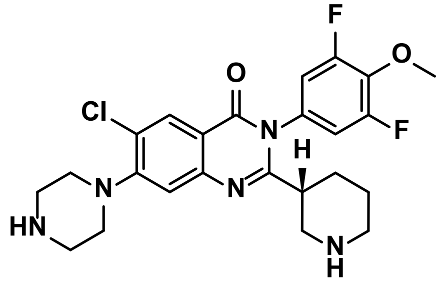

(*R*)-1n

(*R*)-6-chloro-3-(3,5-difluoro-4-methoxyphenyl)-7-(piperazin-1-yl)-2-(piperidin-3-yl)quinazolin-4(3*H*)-one

(***R***)**-1n** (0.026 g, 90.0%) was prepared from (***R***)**-7n** (0.042 g, 0.058 mmol), dichloromethane (0.75 mL), methanol (0.25 mL), and saturated potassium carbonate solution (2 mL) in the same manner described for **protocol C**. Light orange solid. Less than 1% TFA contamination. ^{1}H NMR (400 MHz, $CDCl_3$) δ 8.16 (s, 1H), 7.21 (s, 1H), 6.86 – 6.78 (m, 2H), 4.09 (d, *J* = 1.2 Hz, 3H), 3.18 (dd, *J* = 6.5, 3.1 Hz, 4H), 3.13 – 3.08 (m, 5H), 3.06 – 2.94 (m, 2H), 2.69 (td, *J* = 12.1, 3.0 Hz, 1H), 2.50 (tt, *J* = 9.2, 4.3 Hz, 1H), 2.30 – 2.07 (m, 2H), 1.92 – 1.81 (m, 2H), 1.70 (dq, *J* = 13.6, 3.5 Hz, 1H), 1.40 – 1.22 (m, 1H). ^{13}C NMR (101 MHz, $CDCl_3$) δ 161.1, 158.4, 157.2, 157.2, 157.1, 155.6, 154.8, 154.7, 154.6, 147.4, 137.9, 137.8, 137.6, 131.0, 130.9, 130.8, 128.8, 127.9, 117.4, 115.6, 113.6, 113.5, 113.5, 113.5, 113.4, 113.3, 113.3, 113.2, 62.0, 52.5, 50.6, 46.1, 41.8, 29.9, 25.5. HRMS (ESI-QTOF) m/z: $[M + H]^+$ Calcd for $C_{24}H_{26}ClF_2N_5O_2^+$: 490.1816; Observed: 490.1817. Enantiopurity determined to be >99%.

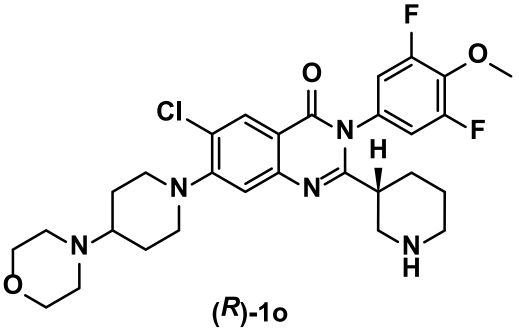

(*R*)-1o

(*R*)-6-chloro-3-(3,5-difluoro-4-methoxyphenyl)-7-(4-morpholinopiperidin-1-yl)-2-(piperidin-3-yl)quinazolin-4(3*H*)-one

(***R***)**-1o** (0.029 g, 96.4%) was prepared from (***R***)**-7o** (0.042 g, 0.052 mmol), dichloromethane (0.75 mL), methanol (0.5 mL), and saturated potassium carbonate solution (2 mL) in the same manner described for **protocol C**. Light yellow solid. Less than 1% TFA contamination. ^{1}H NMR (400 MHz, $CDCl_3$) δ 8.15 (s, 1H), 7.20 (s, 1H), 6.82 (ddt, *J* = 10.0, 4.1, 2.3 Hz, 2H), 4.09 (d, *J* = 1.1 Hz, 3H), 3.78 – 3.71 (m, 4H), 3.66 (d, *J* = 11.9 Hz, 2H), 3.09 (dd, *J* = 12.4, 3.9 Hz, 1H), 3.05 – 2.92 (m, 2H), 2.76 (td, *J* = 11.9, 2.2 Hz, 2H), 2.67 (dd, *J* = 12.1, 2.9 Hz, 1H), 2.64 – 2.57 (m, 4H), 2.42 (dddt, *J* = 26.2, 11.2, 7.6, 4.0 Hz, 2H), 2.02 – 1.93 (m, 2H), 1.92 – 1.64 (m, 6H), 1.37 – 1.21 (m, 1H). ^{13}C NMR (101 MHz, $CDCl_3$) δ 161.2, 158.5, 157.2, 155.6, 154.7, 147.4, 137.7, 131.0, 128.6, 128.0, 117.4, 115.4, 113.6, 113.5, 113.5, 113.5, 113.4, 113.3, 113.3, 113.2, 67.5, 62.0, 61.8, 51.0, 50.8, 50.0, 46.2, 42.0, 30.0, 28.5, 25.7. HRMS (ESI-QTOF) m/z: $[M + H]^+$ Calcd for $C_{29}H_{34}ClF_2N_5O_3^+$: 574.2391; Observed: 574.2384. Enantiopurity determined to be 93.6%.

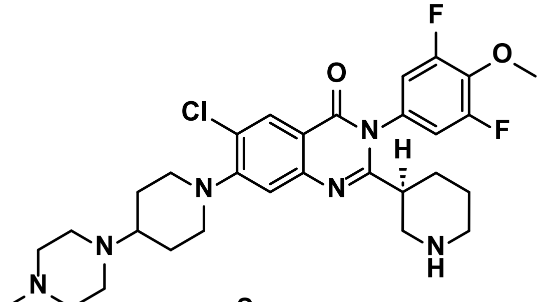

(S)-1p

(*S*)-6-chloro-3-(3,5-difluoro-4-methoxyphenyl)-7-(4-(4-methylpiperazin-1-yl)piperidin-1-yl)-2-(piperidin-3-yl)quinazolin-4(3*H*)-one

(*S*)-1p (0.015 g, 74.7%) was prepared from **(*S*)-7p** (0.031 g, 0.033 mmol), dichloromethane (0.75 mL), methanol (0.25 mL), and saturated potassium carbonate solution (2 mL) in the same manner described for **protocol C**. Yellow solid. Less than 1% TFA contamination. ^{1}H NMR (400 MHz, $CDCl_3$) δ 8.14 (s, 1H), 7.19 (s, 1H), 6.86 – 6.77 (m, 2H), 4.09 (t, *J* = 1.2 Hz, 3H), 3.69 – 3.62 (m, 3H), 3.08 (dd, *J* = 12.2, 3.9 Hz, 1H), 3.03 – 2.92 (m, 2H), 2.80 – 2.60 (m, 8H), 2.45 (qd, *J* = 10.4, 5.6 Hz, 2H), 2.30 (s, 3H), 2.01 – 1.93 (m, 3H), 1.91 – 1.73 (m, 4H), 1.68 (dt, *J* = 13.4, 3.2 Hz, 1H), 1.36 – 1.21 (m, 2H). ^{13}C NMR (101 MHz, $CDCl_3$) δ 161.2, 158.4, 157.2, 155.7, 154.7, 147.4, 137.7, 131.0, 128.6, 128.0, 117.4, 115.3, 113.6, 113.5, 113.4, 113.3, 62.0, 61.6, 55.7, 51.2, 50.9, 49.3, 46.3, 46.2, 42.1, 30.1, 28.6, 25.8. HRMS (ESI-QTOF) m/z: $[M + H]^+$ Calcd for $C_{30}H_{38}ClF_2N_6O_2^+$: 587.2707; Observed: 587.2710. Enantiopurity determined to be >99%.

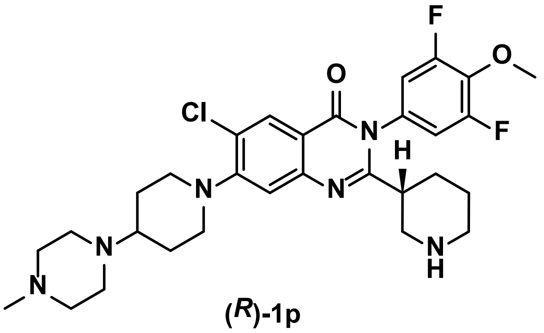

(R)-1p

(*R*)-6-chloro-3-(3,5-difluoro-4-methoxyphenyl)-7-(4-(4-methylpiperazin-1-yl)piperidin-1-yl)-2-(piperidin-3-yl)quinazolin-4(3*H*)-one

(*R*)-1p (0.017 g, 93.3%) was prepared from **(*R*)-7p** (0.029 g, 0.031 mmol), dichloromethane (0.75 mL), methanol (0.25 mL), and saturated potassium carbonate solution (1 mL) in the same manner described for **protocol C**. Light yellow solid. Less than 1% TFA contamination. ^{1}H NMR (400 MHz, $CDCl_3$) δ 8.15 (s, 1H), 7.19 (s, 1H), 6.85 – 6.77 (m, 2H), 4.09 (t, *J* = 1.1 Hz, 3H), 3.24 (t, *J* = 4.7 Hz, 4H), 3.08 (dd, *J* = 12.3, 3.9 Hz, 1H), 3.04 – 3.00 (m, 1H), 2.94 (ddd, *J* = 14.5, 8.1, 2.6 Hz, 3H), 2.78 (t, *J* = 4.8 Hz, 4H), 2.66 (td, *J* = 12.1, 2.9 Hz, 1H), 2.46 (tt, *J* = 9.4, 4.4 Hz, 1H), 2.27 (s, 3H), 1.96 (td, *J* = 11.8, 2.3 Hz, 2H), 1.85 (dtd, *J* = 13.3, 9.9, 3.8 Hz, 5H), 1.73 – 1.56 (m, 3H), 1.37 – 1.21 (m, 2H). ^{13}C NMR (101 MHz, $CDCl_3$) δ 161.2, 158.5, 157.2, 155.3, 154.7, 147.4, 137.7, 131.0, 128.7, 127.7, 117.3, 115.5, 113.6, 113.4, 113.4, 113.3, 62.0, 61.8, 55.6, 51.5, 50.8,

49.1, 46.3, 42.1, 30.1, 28.3, 25.8. HRMS (ESI-QTOF) m/z: $[M + H]^+$ Calcd for $C_{30}H_{38}ClF_2N_6O_2^+$: 587.2707; Observed: 587.2703. Enantiopurity determined to be >99%.

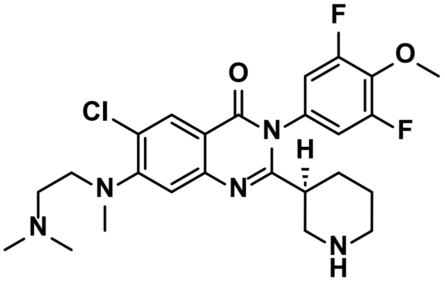

(*S*)-1q

(*S*)-6-chloro-3-(3,5-difluoro-4-methoxyphenyl)-7-((2-(dimethylamino)ethyl)(methyl)amino)-2-(piperidin-3-yl)quinazolin-4(3*H*)-one

(*S*)-1q (0.014 g, 67.4%) was prepared from **(*S*)-7q** (0.031 g, 0.042 mmol), dichloromethane (0.75 mL), methanol (0.25 mL), and saturated potassium carbonate solution (2 mL) in the same manner described for **protocol C**. Light orange solid. Less than 1% TFA contamination. ^{1}H NMR (400 MHz, $CDCl_3$) δ 8.14 (s, 1H), 7.20 (s, 1H), 6.86 – 6.79 (m, 2H), 4.09 (d, *J* = 1.2 Hz, 3H), 3.38 – 3.32 (m, 2H), 3.10 (dd, *J* = 12.3, 3.9 Hz, 1H), 3.05 – 3.00 (m, 1H), 2.99 – 2.93 (m, 4H), 2.68 (dd, *J* = 12.1, 2.9 Hz, 1H), 2.64 – 2.58 (m, 2H), 2.47 (tt, *J* = 9.5, 4.3 Hz, 1H), 2.27 (s, 6H), 1.87 (dq, *J* = 10.2, 3.9 Hz, 2H), 1.69 (dq, *J* = 13.5, 3.4 Hz, 1H), 1.35 – 1.24 (m, 2H). ^{13}C NMR (101 MHz, $CDCl_3$) δ 161.1, 158.5, 157.2, 157.2, 157.1, 155.8, 154.8, 154.7, 154.6, 147.2, 137.9, 137.7, 137.6, 131.2, 131.1, 131.0, 129.0, 126.8, 117.0, 114.7, 113.6, 113.6, 113.5, 113.5, 113.4, 113.4, 113.3, 113.3, 62.0, 57.2, 53.7, 50.8, 46.3, 46.0, 42.1, 40.7, 30.1, 25.8. HRMS (ESI-QTOF) m/z: $[M + H]^+$ Calcd for $C_{25}H_{31}ClF_2N_5O_2^+$: 506.2129; Observed: 506.2128. Enantiopurity determined to be >99%.

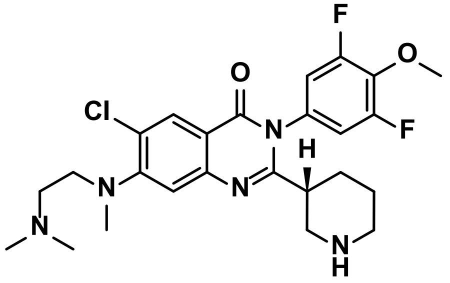

(*R*)-1q

(*R*)-6-chloro-3-(3,5-difluoro-4-methoxyphenyl)-7-((2-(dimethylamino)ethyl)(methyl)amino)-2-(piperidin-3-yl)quinazolin-4(3*H*)-one

(*R*)-1q (0.024 g, 85.9%) was prepared from **(*R*)-7q** (0.041 g, 0.056 mmol), dichloromethane (0.75 mL), methanol (0.25 mL), and saturated potassium carbonate solution (2 mL) in the same manner described for **protocol C**. Orange solid. Less than 1% TFA contamination. ^{1}H NMR (400 MHz, $CDCl_3$) δ 8.13 (s, 1H), 7.20 (s, 1H), 6.86 – 6.77 (m, 2H), 4.09 (s, 3H), 3.40 – 3.30 (m, 2H), 3.10 (dd, *J* = 12.3, 4.0 Hz, 1H), 3.07 – 3.01 (m, 1H), 3.01 – 2.93 (m, 4H), 2.68 (td, *J* = 12.3, 3.0 Hz,

1H), 2.61 (t, 2H), 2.48 (tt, J = 8.9, 4.1 Hz, 1H), 2.27 (s, 6H), 1.87 (td, J = 10.3, 3.9 Hz, 2H), 1.69 (dt, J = 13.6, 3.4 Hz, 1H), 1.37 – 1.23 (m, 2H). ^{13}C NMR (101 MHz, $CDCl_3$) δ 161.1, 158.4, 157.2, 157.2, 157.1, 155.7, 154.7, 154.7, 154.6, 147.2, 137.9, 137.7, 137.6, 131.1, 131.0, 130.9, 129.0, 126.8, 116.9, 114.7, 113.6, 113.6, 113.5, 113.5, 113.4, 113.4, 113.3, 113.3, 62.0, 57.2, 53.7, 50.7, 46.2, 46.0, 41.8, 40.7, 30.0, 25.6. HRMS (ESI-QTOF) m/z: $[M + H]^+$ Calcd for $C_{25}H_{31}ClF_2N_5O_2^+$: 506.2129; Observed: 506.2125. Enantiopurity determined to be >99%.

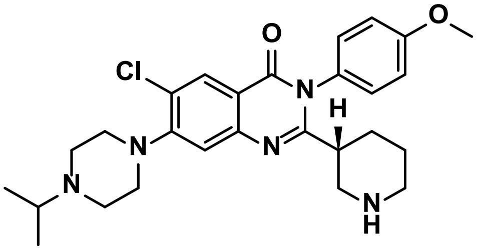

(*R*)-1t
(*R*)-6-chloro-7-(4-isopropylpiperazin-1-yl)-3-(4-methoxyphenyl)-2-(piperidin-3-yl)quinazolin-4(3*H*)-one

(*R*)-1t (0.022 g, 93.5%) was prepared from **(*R*)-7t** (0.035 g, 0.048 mmol), dichloromethane (0.75 mL), methanol (0.25 mL), and saturated potassium carbonate solution (2 mL) in the same manner described for **protocol C**. Light orange solid. Less than 1% TFA contamination. ^{1}H NMR (400 MHz, $CDCl_3$) δ 8.17 (s, 1H), 7.20 (s, 1H), 7.15 – 7.08 (m, 2H), 7.05 – 6.99 (m, 2H), 3.86 (s, 3H), 3.25 (t, J = 4.9 Hz, 4H), 3.08 (dd, J = 12.4, 3.9 Hz, 1H), 3.00 (dd, J = 12.4, 9.7 Hz, 1H), 2.93 (dt, J = 12.4, 3.7 Hz, 1H), 2.75 (q, J = 5.8 Hz, 5H), 2.66 (td, J = 12.1, 2.9 Hz, 1H), 2.51 (tt, J = 9.4, 4.4 Hz, 1H), 2.23 – 1.97 (m, 1H), 1.86 (td, J = 10.0, 4.0 Hz, 2H), 1.66 (dt, J = 13.5, 3.4 Hz, 1H), 1.25 (s, 1H), 1.11 (d, J = 6.5 Hz, 6H). ^{13}C NMR (101 MHz, $CDCl_3$) δ 161.7, 160.1, 159.6, 155.0, 147.6, 129.5, 129.3, 129.3, 128.8, 127.4, 117.1, 115.9, 115.4, 115.2, 55.6, 54.7, 51.5, 50.6, 48.8, 46.2, 41.9, 29.8, 29.8, 25.7, 18.7. HRMS (ESI-QTOF) m/z: $[M + H]^+$ Calcd for $C_{27}H_{35}ClN_5O_2^+$: 496.2474; Observed: 496.2466. Enantiopurity determined to be 93.6%.

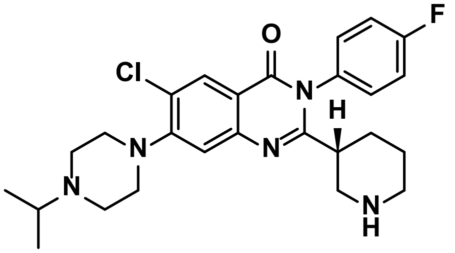

(*R*)-1u
(*R*)-6-chloro-3-(4-fluorophenyl)-7-(4-isopropylpiperazin-1-yl)-2-(piperidin-3-yl)quinazolin-4(3*H*)-one

(*R*)-1u (0.022 g, 91.5%) was prepared from **(*R*)-7u** (0.029 g, 0.049 mmol), dichloromethane (0.75 mL), methanol (0.25 mL), and saturated potassium carbonate solution (2 mL) in the same manner described for **protocol C**. Light yellow solid. Less than 1% TFA contamination. ^{1}H NMR (400 MHz, $CDCl_3$) δ 8.16 (s, 1H), 7.25 – 7.16 (m, 5H), 3.25 (t, J = 4.8 Hz, 4H), 3.08 (dd, J = 12.4, 4.0

Hz, 1H), 3.00 (dd, J = 12.3, 9.8 Hz, 1H), 2.93 (dt, J = 12.4, 3.5 Hz, 1H), 2.75 (t, J = 4.9 Hz, 5H), 2.65 (td, J = 12.2, 2.9 Hz, 1H), 2.42 (tt, J = 9.7, 4.3 Hz, 1H), 1.86 (td, J = 9.4, 3.8 Hz, 2H), 1.65 (dt, J = 13.5, 3.3 Hz, 1H), 1.29 – 1.17 (m, 2H), 1.11 (d, J = 6.5 Hz, 6H). ^{13}C NMR (101 MHz, $CDCl_3$) δ 164.1, 161.6, 161.5, 159.0, 155.2, 147.6, 133.0, 133.0, 130.3, 130.2, 128.7, 127.6, 117.4, 117.2, 117.2, 117.0, 115.7, 54.7, 51.5, 50.7, 48.8, 46.3, 42.2, 29.9, 25.9, 18.7. HRMS (ESI-QTOF) m/z: $[M + H]^+$ Calcd for $C_{26}H_{32}ClFN_5O^+$: 484.2274; Observed: 484.2270. Enantiopurity determined to be 93.5%.

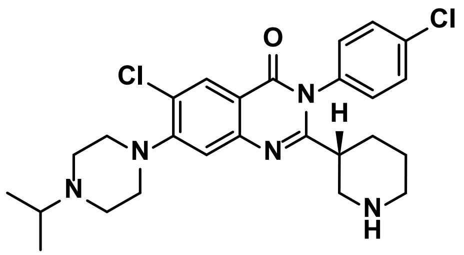

(*R*)-1v

(*R*)-6-chloro-3-(4-chlorophenyl)-7-(4-isopropylpiperazin-1-yl)-2-(piperidin-3-yl)quinazolin-4(3*H*)-one

(*R*)-1v (0.018 g, 86.8%) was prepared from **(*R*)-7v** (0.030 g, 0.041 mmol), dichloromethane (0.75 mL), methanol (0.25 mL), and saturated potassium carbonate solution (2 mL) in the same manner described for **protocol C**. Light orange solid. Less than 1% TFA contamination. ^1H NMR (400 MHz, $CDCl_3$) δ 8.17 (s, 1H), 7.55 – 7.48 (m, 2H), 7.21 (s, 1H), 7.16 (ddt, J = 8.5, 3.7, 1.7 Hz, 2H), 3.25 (t, J = 4.9 Hz, 4H), 3.07 (dd, J = 12.3, 4.0 Hz, 1H), 3.00 (dd, J = 12.3, 9.8 Hz, 1H), 2.93 (dt, J = 12.6, 3.5 Hz, 1H), 2.81 – 2.71 (m, 5H), 2.65 (td, J = 12.2, 2.9 Hz, 1H), 2.45 – 2.37 (m, 1H), 1.86 (td, J = 9.0, 3.8 Hz, 2H), 1.66 (dt, J = 13.5, 3.3 Hz, 1H), 1.24 (p, J = 8.2 Hz, 2H), 1.11 (d, J = 6.5 Hz, 6H). ^{13}C NMR (101 MHz, $CDCl_3$) δ 161.3, 158.8, 155.2, 147.6, 135.7, 135.6, 130.5, 130.3, 129.8, 128.8, 127.6, 117.2, 115.7, 54.7, 51.5, 50.7, 48.8, 46.3, 42.2, 29.9, 25.8, 18.8. HRMS (ESI-QTOF) m/z: $[M + H]^+$ Calcd for $C_{26}H_{32}Cl_2N_5O^+$: 500.1978; Observed: 500.1974. Enantiopurity determined to be 92.0%.

Protocol D (refer to Scheme S1):

Boc-protected quinazolinone was dissolved in ACS grade dichloromethane and cooled to 0 °C using an ice-water bath and allowed to stir for 10 min. To this cooled solution, trifluoroacetic acid was added dropwise over 1 min, the ice-water bath was removed and the reaction was allowed to reach to room temperature over 2 h. Saturated potassium carbonate solution was added to the reaction mixture dropwise until bubbles stopped forming and the resulting mixture was stirred vigorously for 2 h. The solution was diluted with dichloromethane (80 mL) and washed 5 times with sat. potassium carbonate solution (5 x 60 mL). The organic extract was dried over anhydrous sodium sulfate, filtered, and the solvent was evaporated under reduced pressure to afford the free base product.

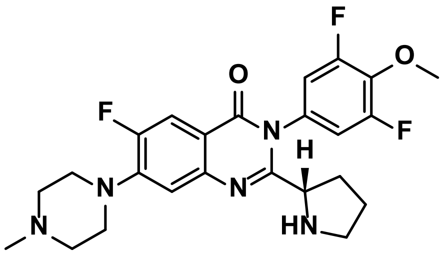

(*S*)-1a

(*S*)-3-(3,5-difluoro-4-methoxyphenyl)-6-fluoro-7-(4-methylpiperazin-1-yl)-2-(pyrrolidin-2-yl)quinazolin-4(3*H*)-one

(*S*)-1a (0.11 g, 77.4%) was prepared from **(*S*)-6a** (0.17 g, 0.29 mmol), dichloromethane (3.0 mL), trifluoroacetic acid (0.66 mL, 8.63 mmol) and saturated potassium carbonate solution (5 mL) in the same manner described for **protocol D**. Light orange solid. Less than 1% TFA contamination by ^{19}F NMR. ^{1}H NMR (400 MHz, $CDCl_3$) δ 7.75 (d, *J* = 12.9 Hz, 1H), 7.09 (d, *J* = 7.8 Hz, 1H), 6.86 (ddt, *J* = 18.7, 10.4, 2.3 Hz, 2H), 4.07 (t, *J* = 1.2 Hz, 3H), 3.76 (t, *J* = 6.7 Hz, 1H), 3.36 – 3.27 (m, 4H), 3.24 (ddd, *J* = 11.2, 6.9, 4.6 Hz, 1H), 2.80 – 2.73 (m, 1H), 2.61 (t, *J* = 4.9 Hz, 4H), 2.36 (s, 3H), 1.83 – 1.64 (m, 4H). ^{13}C NMR (101 MHz, $CDCl_3$) δ 161.3, 161.3, 157.9, 157.9, 157.1, 157.0, 156.9, 156.9, 155.6, 154.6, 154.5, 154.4, 154.4, 153.1, 146.9, 146.8, 145.1, 145.1, 137.8, 137.7, 137.5, 130.6, 130.5, 130.4, 115.5, 115.5, 114.4, 114.4, 114.2, 114.2, 114.0, 113.9, 113.8, 113.8, 113.6, 113.6, 112.9, 112.6, 62.0, 62.0, 62.0, 59.7, 55.0, 50.0, 50.0, 47.8, 46.2, 33.2, 27.1. HRMS (ESI-QTOF) m/z: $[M + H]^+$ Calcd for $C_{24}H_{27}F_3N_5O_2^+$: 474.2111; Observed: 474.2110. Enantiopurity determined to be >99%.

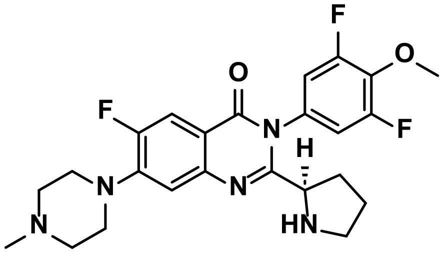

(*R*)-1a

(*R*)-3-(3,5-difluoro-4-methoxyphenyl)-6-fluoro-7-(4-methylpiperazin-1-yl)-2-(pyrrolidin-2-yl)quinazolin-4(3*H*)-one

(*R*)-1a (0.12 g, 81.1%) was prepared from **(*R*)-6a** (0.18 g, 0.31 mmol), dichloromethane (3.0 mL), trifluoroacetic acid (0.71 mL, 9.31 mmol) and saturated potassium carbonate solution (5 mL) in the same manner described for **protocol D**. Orange-brown solid. Less than 1% TFA contamination by ^{19}F NMR. ^{1}H NMR (400 MHz, $CDCl_3$) δ 7.75 (d, *J* = 12.9 Hz, 1H), 7.09 (d, *J* = 7.8 Hz, 1H), 6.86 (ddt, *J* = 18.7, 10.4, 2.2 Hz, 2H), 4.07 (d, *J* = 1.2 Hz, 3H), 3.76 (t, *J* = 6.7 Hz, 1H), 3.32 – 3.27 (m, 4H), 3.23 (ddd, *J* = 11.2, 7.0, 4.7 Hz, 1H), 2.80 – 2.72 (m, 1H), 2.61 (t, *J* = 4.9 Hz, 4H), 2.36 (s, 3H), 1.74 (tdd, *J* = 22.9, 11.6, 5.9 Hz, 4H). ^{13}C NMR (101 MHz, $CDCl_3$) δ 161.3, 161.3, 157.9, 157.9, 157.1, 157.0, 156.9, 156.8, 155.6, 154.6, 154.5, 154.4, 154.4, 153.1, 146.9, 146.8,

145.1, 145.1, 137.8, 137.7, 137.5, 130.6, 130.5, 130.4, 115.5, 115.5, 114.4, 114.4, 114.2, 114.2, 114.0, 113.9, 113.8, 113.8, 113.6, 113.6, 112.9, 112.6, 62.0, 62.0, 62.0, 59.7, 55.0, 50.0, 50.0, 47.8, 46.2, 33.2, 27.1. HRMS (ESI-QTOF) m/z: $[M + H]^+$ Calcd for $C_{24}H_{27}F_3N_5O_2^+$: 474.2111; Observed: 474.2112. Enantiopurity determined to be >99%.

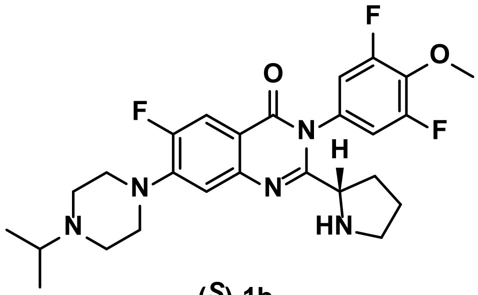

(*S*)-1b

(*S*)-3-(3,5-difluoro-4-methoxyphenyl)-6-fluoro-7-(4-isopropylpiperazin-1-yl)-2-(pyrrolidin-2-yl)quinazolin-4(3*H*)-one

(*S*)-1b (0.074 g, 77.3%) was prepared from **(*S*)-6b** (0.12 g, 0.19 mmol), dichloromethane (2.0 mL), trifluoroacetic acid (0.44 mL, 5.73 mmol) and saturated potassium carbonate solution (3 mL) in the same manner described for **protocol D**. Orange solid. Less than 1% TFA contamination by ^{19}F NMR. ^1H NMR (400 MHz, $CDCl_3$) δ 7.74 (dd, *J* = 12.9, 1.2 Hz, 1H), 7.07 (d, *J* = 7.8 Hz, 1H), 6.86 (ddt, *J* = 18.7, 10.4, 2.3 Hz, 2H), 4.07 (s, 3H), 3.76 (t, *J* = 6.8 Hz, 1H), 3.29 (dd, *J* = 6.4, 3.6 Hz, 4H), 3.23 (ddd, *J* = 11.0, 6.8, 4.9 Hz, 1H), 2.96 – 2.82 (m, 1H), 2.72 (q, *J* = 5.0 Hz, 5H), 1.74 (dddd, *J* = 26.7, 21.7, 11.2, 5.9 Hz, 4H), 1.09 (d, *J* = 6.5 Hz, 6H). ^{13}C NMR (101 MHz, $CDCl_3$) δ 161.3, 161.3, 157.8, 157.0, 157.0, 156.9, 156.8, 155.6, 154.6, 154.5, 154.4, 154.3, 153.1, 147.0, 146.9, 145.1, 137.8, 137.6, 137.5, 130.6, 130.5, 130.4, 115.4, 115.3, 114.4, 114.4, 114.2, 114.2, 113.9, 113.8, 113.8, 113.6, 113.6, 112.8, 112.6, 62.0, 62.0, 61.9, 59.7, 54.6, 50.4, 50.3, 48.6, 47.7, 33.2, 27.0, 18.6, 18.6. HRMS (ESI-QTOF) m/z: $[M + H]^+$ Calcd for $C_{26}H_{31}F_3N_5O_2^+$: 502.2424; Observed: 502.2418. Enantiopurity determined to be 95.7%.

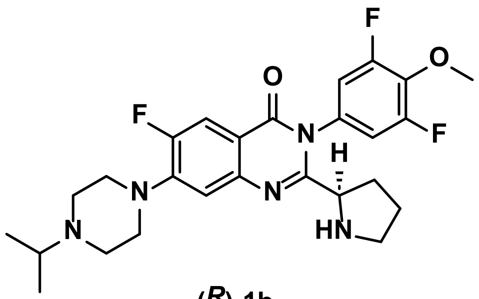

(*R*)-1b

(*R*)-3-(3,5-difluoro-4-methoxyphenyl)-6-fluoro-7-(4-isopropylpiperazin-1-yl)-2-(pyrrolidin-2-yl)quinazolin-4(3*H*)-one

(*R*)-1b (0.083 g, 73.9%) was prepared from **(*R*)-6b** (0.13 g, 0.22 mmol), dichloromethane (2.2 mL), trifluoroacetic acid (0.51 mL, 6.68 mmol) and saturated potassium carbonate solution (3 mL) in the same manner described for **protocol D**. Light orange solid. Less than 1% TFA contamination

by ^{19}F NMR. ^{1}H NMR (400 MHz, CDCl$_3$) δ 7.74 (d, *J* = 12.9 Hz, 1H), 7.07 (d, *J* = 7.8 Hz, 1H), 6.86 (ddt, *J* = 18.8, 10.4, 2.3 Hz, 2H), 4.07 (d, *J* = 1.2 Hz, 3H), 3.76 (t, *J* = 6.8 Hz, 1H), 3.29 (dd, *J* = 6.5, 3.6 Hz, 4H), 3.23 (ddd, *J* = 11.0, 6.8, 4.4 Hz, 1H), 2.88 (s, 1H), 2.81 – 2.65 (m, 6H), 1.85 – 1.62 (m, 3H), 1.08 (d, *J* = 6.5 Hz, 7H). ^{13}C NMR (101 MHz, CDCl$_3$) δ 161.3, 161.3, 157.8, 157.8, 157.0, 157.0, 156.9, 156.8, 155.6, 154.6, 154.5, 154.4, 154.3, 153.1, 147.0, 146.9, 145.1, 137.8, 137.6, 137.5, 130.6, 130.5, 130.4, 115.4, 115.3, 114.4, 114.4, 114.2, 114.2, 113.9, 113.8, 113.6, 113.6, 112.8, 112.6, 77.5, 76.8, 62.0, 62.0, 61.9, 59.7, 54.6, 50.4, 50.3, 48.6, 47.7, 33.2, 27.0, 18.6. HRMS (ESI-QTOF) m/z: [M + H]$^+$ Calcd for $C_{26}H_{31}F_3N_5O_2^+$: 502.2424; Observed: 502.2416. Enantiopurity determined to be >99%.

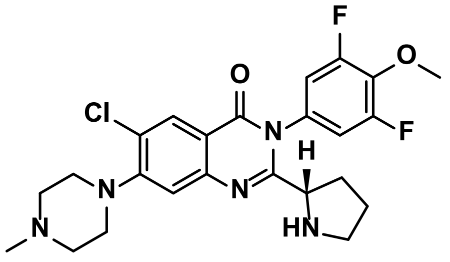

(*S*-1c

(*S*)-6-chloro-3-(3,5-difluoro-4-methoxyphenyl)-7-(4-methylpiperazin-1-yl)-2-(pyrrolidin-2-yl)quinazolin-4(3*H*)-one

(*S*)-1c (0.006 g, 49.0%) was prepared from **(*S*)-6c** (0.015 g, 0.025 mmol), dichloromethane (0.6 mL), trifluoroacetic acid (58.4 *μ*L, 0.76 mmol) and saturated potassium carbonate solution (1.5 mL) in the same manner described for **protocol D**. Yellow solid. Less than 1% TFA contamination by ^{19}F NMR. ^{1}H NMR (400 MHz, CDCl$_3$) δ 8.18 (s, 1H), 7.23 (s, 1H), 6.87 (ddt, *J* = 20.1, 10.4, 2.3 Hz, 2H), 4.10 (d, *J* = 1.3 Hz, 3H), 3.78 (t, *J* = 6.8 Hz, 1H), 3.26 (p, *J* = 5.6 Hz, 5H), 2.79 (dt, *J* = 11.4, 6.5 Hz, 1H), 2.66 (d, *J* = 5.0 Hz, 4H), 2.39 (s, 3H), 1.86 – 1.66 (m, 4H). ^{13}C NMR (101 MHz, CDCl$_3$) δ 161.1, 158.8, 157.0, 157.0, 155.3, 154.6, 147.1, 137.8, 130.4, 128.9, 128.0, 117.5, 115.7, 114.4, 114.4, 114.2, 113.9, 113.8, 113.6, 113.6, 62.1, 59.8, 55.1, 51.1, 47.8, 46.3, 33.3, 27.1. HRMS (ESI-QTOF) m/z: [M + H]$^+$ Calcd for $C_{24}H_{27}ClF_2N_5O_2^+$: 490.1816; Observed: 490.1808. Enantiopurity determined to be >99%.

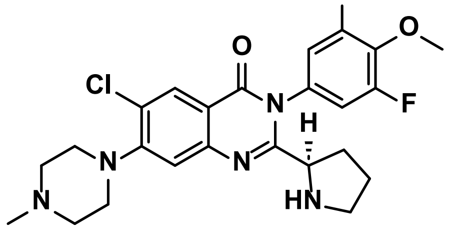

(*R*)-1c

(*R*)-6-chloro-3-(3,5-difluoro-4-methoxyphenyl)-7-(4-methylpiperazin-1-yl)-2-(pyrrolidin-2-yl)quinazolin-4(3*H*)-one

(*R*)-1c (0.057 g, 91.9%) was prepared from **(*R*)-6c** (0.075 g, 0.13 mmol), dichloromethane (1.3 mL), trifluoroacetic acid (0.29 mL, 3.81 mmol) and saturated potassium carbonate solution (3 mL) in the same manner described for **protocol D**. Light orange solid. Less than 1% TFA contamination by ^{19}F NMR. ^{1}H NMR (400 MHz, $CDCl_3$) δ 8.13 (s, 1H), 7.20 (s, 1H), 6.85 (ddt, *J* = 18.2, 10.6, 2.3 Hz, 2H), 4.07 (d, *J* = 1.2 Hz, 3H), 3.76 (t, *J* = 6.8 Hz, 1H), 3.23 (q, *J* = 6.3 Hz, 5H), 2.86 (s, 1H), 2.76 (dt, *J* = 10.8, 6.5 Hz, 1H), 2.62 (t, *J* = 4.8 Hz, 4H), 2.36 (s, 3H), 1.75 (dddd, *J* = 26.5, 13.4, 11.6, 5.7 Hz, 3H). ^{13}C NMR (101 MHz, $CDCl_3$) δ 161.0, 158.8, 157.0, 157.0, 156.9, 156.8, 155.2, 154.5, 154.5, 154.4, 154.3, 147.0, 137.8, 137.7, 137.6, 130.4, 130.3, 130.2, 128.8, 127.8, 117.4, 115.6, 114.4, 114.3, 114.1, 114.1, 113.8, 113.7, 113.6, 113.5, 62.0, 62.0, 61.9, 59.7, 55.0, 51.0, 47.8, 46.2, 33.2, 27.0. HRMS (ESI-QTOF) m/z: $[M + H]^+$ Calcd for $C_{24}H_{27}ClF_2N_5O_2^+$: 490.1816; Observed: 490.1809. Enantiopurity determined to be >99%.

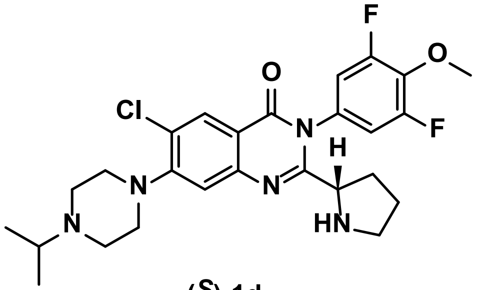

(*S*)-1d

(*S*)-6-chloro-3-(3,5-difluoro-4-methoxyphenyl)-7-(4-isopropylpiperazin-1-yl)-2-(pyrrolidin-2-yl)quinazolin-4(3*H*)-one

(*S*)-1d (0.035 g, 58.0%) was prepared from **(*S*)-6d** (0.073 g, 0.12 mmol), dichloromethane (1.2 mL), trifluoroacetic acid (0.27 mL, 3.53 mmol) and saturated potassium carbonate solution (3 mL) in the same manner described for **protocol D**. Orange solid. Less than 1% TFA contamination by ^{19}F NMR. ^{1}H NMR (400 MHz, $CDCl_3$) δ 8.15 (s, 1H), 7.19 (s, 1H), 6.86 (ddt, *J* = 18.6, 10.3, 2.2 Hz, 2H), 4.08 (d, *J* = 1.2 Hz, 3H), 3.77 (t, *J* = 6.8 Hz, 1H), 3.24 (t, *J* = 5.3 Hz, 5H), 2.74 (t, *J* = 4.8 Hz, 5H), 1.85 – 1.67 (m, 4H), 1.24 (s, 2H), 1.10 (d, *J* = 6.5 Hz, 6H). ^{13}C NMR (101 MHz, $CDCl_3$) δ 161.1, 158.7, 157.1, 157.0, 156.9, 156.9, 155.3, 154.6, 154.5, 154.4, 154.4, 147.0, 137.9, 137.7, 137.6, 130.5, 130.3, 130.2, 128.8, 127.9, 117.3, 115.5, 114.4, 114.4, 114.2, 114.1, 113.8, 113.8, 113.6, 113.6, 62.0, 62.0, 62.0, 60.5, 59.7, 54.6, 51.4, 48.7, 47.7, 33.2, 29.8, 27.0, 18.7, 18.7. HRMS (ESI-QTOF) m/z: $[M + H]^+$ Calcd for $C_{26}H_{31}ClF_2N_5O_2^+$: 518.2129; Observed: 518.2141. Enantiopurity determined to be >99%.

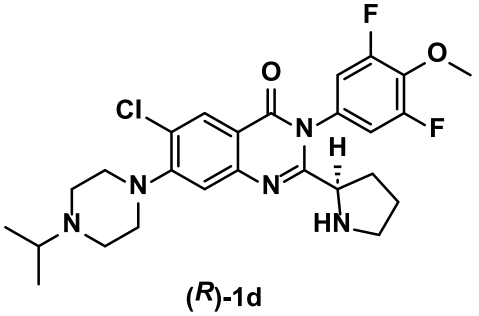

(*R*)-1d

(*R*)-6-chloro-3-(3,5-difluoro-4-methoxyphenyl)-7-(4-isopropylpiperazin-1-yl)-2-(pyrrolidin-2-yl)quinazolin-4(3*H*)-one

***(R)*-1d** (0.032 g, 78.5%) was prepared from **(*R*)-6d** (0.048 g, 0.078 mmol), dichloromethane (1.0 mL), trifluoroacetic acid (0.18 mL, 2.33 mmol) and saturated potassium carbonate solution (2 mL) in the same manner described for **protocol D**. Orange solid. 2% TFA contamination by ^{19}F NMR. ^{1}H NMR (400 MHz, $CDCl_3$) δ 8.15 (s, 1H), 7.19 (s, 1H), 6.86 (ddt, *J* = 18.7, 10.4, 2.3 Hz, 2H), 4.08 (t, *J* = 1.2 Hz, 3H), 3.77 (t, *J* = 6.8 Hz, 1H), 3.25 (d, *J* = 5.1 Hz, 5H), 2.75 (dt, *J* = 9.6, 5.7 Hz, 7H), 1.86 – 1.65 (m, 2H), 1.10 (d, *J* = 6.5 Hz, 6H). ^{13}C NMR (101 MHz, $CDCl_3$) δ 161.1, 158.7, 157.1, 157.0, 156.9, 156.9, 155.4, 154.6, 154.5, 154.4, 154.4, 147.1, 137.9, 137.7, 137.6, 130.5, 130.4, 130.2, 128.8, 127.9, 117.3, 115.5, 114.4, 114.4, 114.2, 114.1, 113.8, 113.8, 113.6, 113.6, 62.0, 62.0, 62.0, 59.8, 54.6, 51.4, 48.7, 47.8, 33.2, 27.1, 18.7, 18.7. HRMS (ESI-QTOF) m/z: [M + H]$^+$ Calcd for $C_{26}H_{31}ClF_2N_5O_2^+$: 518.2129; Observed: 518.2117. Enantiopurity determined to be >99%.

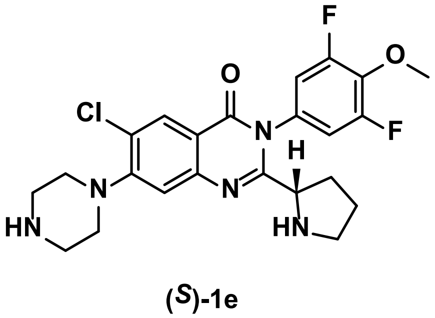

(*S*)-1e

(*S*)-6-chloro-3-(3,5-difluoro-4-methoxyphenyl)-7-(piperazin-1-yl)-2-(pyrrolidin-2-yl)quinazolin-4(3*H*)-one

(*R*)-1e (0.030 g, 86% yield) was prepared from **(*S*)-6e** (0.052 g, 0.070 mmol), dichloromethane (0.7 mL), trifluoroacetic acid (0.29 mL, 2.10 mmol) and saturated potassium carbonate solution (3 mL) in the same manner described for **protocol** D**.** Off-white solid. <1% TFA contamination. ^{1}H NMR (400 MHz, $CDCl_3$) δ 8.16 (s, 1H), 7.20 (s, 1H), 6.86 (ddt, *J* = 20.4, 10.4, 2.3 Hz, 2H), 4.08 (d, *J* = 1.2 Hz, 3H), 3.77 (t, *J* = 6.8 Hz, 1H), 3.24 (ddd, *J* = 11.3, 6.9, 4.6 Hz, 1H), 3.17 (q, *J* = 3.5, 3.0 Hz, 4H), 3.08 (t, *J* = 4.7 Hz, 4H), 2.77 (dt, *J* = 11.0, 6.8 Hz, 1H), 2.23 (s, 3H), 1.84 – 1.66 (m, 3H). ^{19}F NMR (376 MHz, $CDCl_3$) δ - 125.62 (d, *J* = 9.2 Hz), -126.14 (d, *J* = 9.1 Hz). ^{13}C NMR (101 MHz, CDCl3) δ 161.1, 158.8, 157.1, 155.7, 154.6, 147.1, 137.9, 130.4, 128.8, 128.0, 117.4,

115.6, 114.4, 113.6, 62.0, 62.0, 59.7, 52.5, 47.8, 46.1, 33.2, 29.8, 27.1. HRMS (ESI-QTOF) m/z: $[M + H]^+$ Calcd for $C_{23}H_{25}ClF_2N_5O_2^+$: 476.1659; Observed: 476.1670. Enantiopurity determined to be >99%.

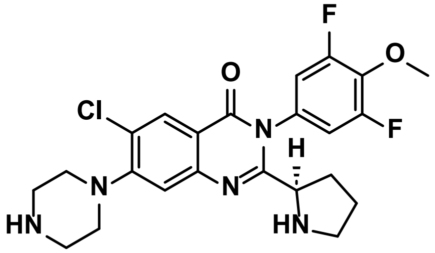

(*R*)-1e

(*R*)-6-chloro-3-(3,5-difluoro-4-methoxyphenyl)-7-(piperazin-1-yl)-2-(pyrrolidin-2-yl)quinazolin-4(3*H*)-one

(*R*)-1e (0.048 g, 71.2%) was prepared from **(*R*)-6e** (0.083 g, 0.14 mmol), dichloromethane (1.4 mL), trifluoroacetic acid (0.33 mL, 4.30 mmol) and saturated potassium carbonate solution (3 mL) in the same manner described for **protocol D**. Brown solid. Less than 1% TFA contamination by ^{19}F NMR. ^1H NMR (400 MHz, $CDCl_3$) δ 8.15 (s, 1H), 7.20 (s, 1H), 6.86 (ddt, *J* = 20.1, 10.4, 2.2 Hz, 2H), 4.08 (t, *J* = 1.2 Hz, 3H), 3.77 (t, *J* = 6.8 Hz, 1H), 3.24 (ddd, *J* = 11.2, 6.9, 4.6 Hz, 1H), 3.17 (q, *J* = 3.3 Hz, 4H), 3.08 (t, *J* = 4.6 Hz, 4H), 2.77 (dt, *J* = 10.9, 6.6 Hz, 1H), 1.76 (ddtd, *J* = 21.4, 14.8, 7.1, 3.6 Hz, 3H), 1.23 (q, *J* = 6.9 Hz, 3H). ^{13}C NMR (101 MHz, $CDCl_3$) δ 161.1, 158.8, 157.1, 157.0, 156.9, 156.9, 155.7, 154.6, 154.5, 154.4, 154.4, 147.1, 137.9, 137.7, 137.6, 130.4, 130.3, 130.2, 128.8, 128.0, 117.4, 115.6, 114.4, 114.3, 114.2, 114.1, 113.8, 113.8, 113.6, 113.6, 62.0, 62.0, 62.0, 59.7, 52.5, 47.8, 46.1, 33.2, 29.8, 27.1. HRMS (ESI-QTOF) m/z: $[M + H]^+$ Calcd for $C_{23}H_{25}ClF_2N_5O_2^+$: 476.1659; Observed: 476.1676. Enantiopurity determined to be >99%.

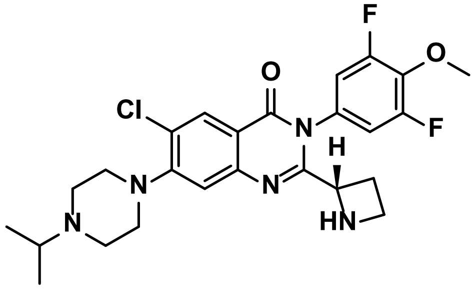

(*S*)-1f

(*S*)-2-(azetidin-2-yl)-6-chloro-3-(3,5-difluoro-4-methoxyphenyl)-7-(4-isopropylpiperazin-1-yl)quinazolin-4(3*H*)-one

(*S*)-1f (0.025 g, 81.2%) was prepared from **(*S*)-6f** (0.037 g, 0.061 mmol), dichloromethane (1.0 mL), trifluoroacetic acid (0.14 mL, 1.84 mmol) and saturated potassium carbonate solution (2 mL) in the same manner described for **protocol D**. Orange solid. Less than 1% TFA contamination by ^{19}F NMR. ^1H NMR (400 MHz, $CDCl_3$) δ 8.18 (s, 1H), 7.32 (s, 1H), 6.82 (dt, *J* = 10.4, 2.2 Hz, 1H), 6.75 (dt, *J* = 10.4, 2.2 Hz, 1H), 4.50 (dd, *J* = 8.3, 6.5 Hz, 1H), 4.09 (d, *J* = 1.2 Hz, 3H), 3.59 – 3.42

(m, 2H), 3.27 (t, J = 4.8 Hz, 3H), 2.79 – 2.69 (m, 6H), 2.46 (ddt, J = 10.9, 9.1, 6.7 Hz, 1H), 2.31 (dtd, J = 11.1, 8.3, 5.8 Hz, 1H), 1.25 (s, 1H), 1.12 (d, J = 6.5 Hz, 6H). ^{13}C NMR (101 MHz, $CDCl_3$) δ 161.0, 157.5, 155.4, 147.2, 128.9, 128.0, 117.5, 115.5, 113.8, 113.6, 113.4, 62.0, 58.5, 54.7, 51.5, 48.7, 43.4, 27.6, 18.8. HRMS (ESI-QTOF) m/z: $[M + H]^+$ Calcd for $C_{25}H_{29}ClF_2N_5O_2^+$: 504.1972; Observed: 504.1956. Enantiopurity determined to be 64.2%.

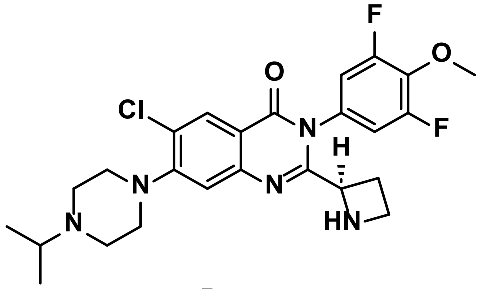

(*R*)-1f

(*R*)-2-(azetidin-2-yl)-6-chloro-3-(3,5-difluoro-4-methoxyphenyl)-7-(4-isopropylpiperazin-1-yl)quinazolin-4(3*H*)-one

(*R*)-1f (0.016 g, 94.8%) was prepared from **(*R*)-6f** (0.021 g, 0.035 mmol), dichloromethane (1.0 mL), trifluoroacetic acid (0.08 mL, 1.04 mmol) and saturated potassium carbonate solution (2 mL) in the same manner described for **protocol D**. Orange solid. Less than 1% TFA contamination by ^{19}F NMR. ^{1}H NMR (400 MHz, $CDCl_3$) δ 8.19 (s, 1H), 7.33 (s, 1H), 6.83 (dt, J = 10.4, 2.2 Hz, 1H), 6.75 (dt, J = 10.4, 2.2 Hz, 1H), 4.51 (dd, J = 8.3, 6.5 Hz, 1H), 4.09 (d, J = 1.2 Hz, 3H), 3.56 (td, J = 8.4, 5.9 Hz, 1H), 3.48 (q, J = 7.6 Hz, 1H), 3.28 (t, J = 4.8 Hz, 4H), 2.76 (dd, J = 5.9, 3.6 Hz, 5H), 2.47 (ddt, J = 11.0, 9.1, 6.7 Hz, 1H), 2.32 (dtd, J = 11.1, 8.2, 5.7 Hz, 1H), 1.25 (s, 1H), 1.12 (d, J = 6.5 Hz, 6H). ^{13}C NMR (101 MHz, $CDCl_3$) δ 161.1, 157.6, 155.5, 147.2, 128.9, 128.0, 117.6, 115.6, 113.9, 113.7, 113.5, 62.1, 58.6, 54.7, 51.5, 48.8, 43.4, 27.6, 18.8. HRMS (ESI-QTOF) m/z: $[M + H]^+$ Calcd for $C_{25}H_{29}ClF_2N_5O_2^+$: 504.1972; Observed: 504.1956. Enantiopurity determined to be 40.5%.

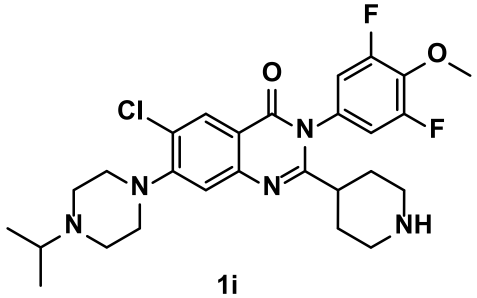

1i

6-chloro-3-(3,5-difluoro-4-methoxyphenyl)-7-(4-isopropylpiperazin-1-yl)-2-(piperidin-4-yl)quinazolin-4(3*H*)-one

1i (0.024 g, 95.8%) was prepared from **6i** (0.030 g, 0.048 mmol), dichloromethane (1.0 mL), trifluoroacetic acid (0.11 mL, 1.42 mmol) and saturated potassium carbonate solution (2 mL) in the same manner described for **protocol D**. Brown solid. Less than 1% TFA contamination by ^{19}F

NMR. ^{1}H NMR (400 MHz, $CDCl_3$) δ 8.16 (s, 1H), 7.20 (s, 1H), 6.86 – 6.78 (m, 2H), 4.10 (d, *J* = 1.2 Hz, 3H), 3.25 (t, *J* = 4.8 Hz, 4H), 3.10 (dt, *J* = 12.7, 3.1 Hz, 2H), 2.75 (t, *J* = 4.8 Hz, 4H), 2.43 (ddd, *J* = 12.0, 10.1, 2.4 Hz, 4H), 1.90 (qd, *J* = 12.6, 3.9 Hz, 2H), 1.70 (d, *J* = 12.2 Hz, 2H), 1.11 (d, *J* = 6.5 Hz, 6H). ^{13}C NMR (101 MHz, $CDCl_3$) δ 161.3, 159.4, 157.2, 157.1, 155.4, 154.7, 154.6, 147.6, 137.7, 132.3, 132.2, 132.1, 131.2, 131.1, 131.0, 128.7, 128.6, 127.7, 117.3, 115.4, 113.6, 113.5, 113.4, 113.3, 62.1, 54.7, 51.4, 48.7, 46.1, 41.2, 31.7, 29.8, 18.7. HRMS (ESI-QTOF) m/z: $[M + H]^+$ Calcd for $C_{27}H_{33}ClF_2N_5O_2^+$: 532.2285; Observed: 532.2276.

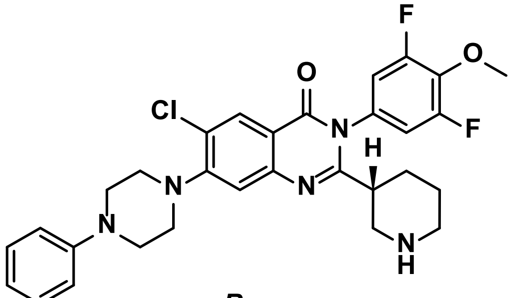

(*R*)-1k

(*R*)-6-chloro-3-(3,5-difluoro-4-methoxyphenyl)-7-(4-phenylpiperazin-1-yl)-2-(piperidin-3-yl)quinazolin-4(3*H*)-one

***(R)*-1k** (0.054 g, 82.2%) was prepared from **(R)-6k** (0.077 g, 0.12 mmol), dichloromethane (1.2 mL), trifluoroacetic acid (0.27 mL, 3.47 mmol) and saturated potassium carbonate solution (3 mL) in the same manner described for **protocol D**. Yellow solid. Less than 1% TFA contamination by ^{19}F NMR. ^{1}H NMR (400 MHz, $CDCl_3$) δ 8.18 (s, 1H), 7.34 – 7.26 (m, 2H), 7.25 (s, 1H), 7.03 – 6.96 (m, 2H), 6.94 – 6.86 (m, 1H), 6.86 – 6.78 (m, 2H), 4.09 (t, *J* = 1.2 Hz, 3H), 3.39 (t, *J* = 4.8 Hz, 7H), 3.10 (dd, *J* = 12.4, 3.9 Hz, 1H), 3.06 – 3.00 (m, 1H), 2.96 (dt, *J* = 13.3, 3.5 Hz, 1H), 2.67 (td, *J* = 12.2, 2.9 Hz, 1H), 2.47 (tt, *J* = 9.3, 4.3 Hz, 1H), 1.88 (td, *J* = 10.2, 3.9 Hz, 2H), 1.78 (s, 2H), 1.69 (dt, *J* = 13.5, 3.2 Hz, 1H), 1.38 – 1.16 (m, 1H). ^{13}C NMR (101 MHz, $CDCl_3$) δ 161.1, 158.6, 157.2, 155.1, 154.7, 151.2, 147.4, 137.9, 137.7, 137.6, 131.0, 130.9, 130.8, 129.3, 128.8, 127.8, 120.3, 117.4, 116.4, 115.8, 113.6, 113.5, 113.5, 113.4, 113.3, 113.3, 113.3, 113.2, 62.0, 51.2, 50.8, 49.4, 46.2, 42.0, 30.0, 29.8, 25.7. HRMS (ESI-QTOF) m/z: $[M + H]^+$ Calcd for $C_{30}H_{31}ClF_2N_5O_2^+$: 566.2129; Observed: 566.2127.

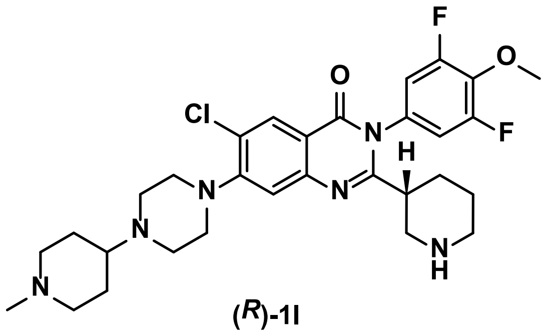

(*R*)-1l

(*R*)-6-chloro-3-(3,5-difluoro-4-methoxyphenyl)-7-(4-(1-methylpiperidin-4-yl)piperazin-1-yl)-2-(piperidin-3-yl)quinazolin-4(3*H*)-one

(*R*)-1l (0.064 g, 70.7%) was prepared from **(*R*)-6l** (0.11 g, 0.16 mmol), dichloromethane (1.5 mL), trifluoroacetic acid (0.36 mL, 4.64 mmol) and saturated potassium carbonate solution (3 mL) in the same manner described for **protocol D**. Orange solid. Less than 1% TFA contamination by ^{19}F NMR. ^{1}H NMR (400 MHz, $CDCl_3$) δ 8.09 (s, 1H), 7.15 (s, 1H), 6.84 – 6.73 (m, 2H), 4.05 (s, 3H), 3.20 (t, *J* = 4.6 Hz, 4H), 3.05 (dd, *J* = 12.3, 4.0 Hz, 1H), 3.00 – 2.84 (m, 4H), 2.74 (t, *J* = 4.7 Hz, 4H), 2.62 (td, *J* = 12.1, 2.9 Hz, 1H), 2.42 (tt, *J* = 9.2, 4.2 Hz, 1H), 2.34 – 2.18 (m, 5H), 1.93 (td, *J* = 11.9, 2.3 Hz, 2H), 1.87 – 1.77 (m, 4H), 1.62 (dtd, *J* = 20.5, 10.6, 5.7 Hz, 3H), 1.33 – 1.17 (m, 1H). ^{13}C NMR (101 MHz, $CDCl_3$) δ 161.0, 158.4, 157.1, 157.1, 157.0, 155.1, 154.6, 154.6, 154.5, 147.3, 137.7, 137.6, 137.5, 131.0, 130.9, 130.8, 128.6, 127.6, 117.1, 115.4, 113.5, 113.4, 113.4, 113.4, 113.3, 113.2, 113.2, 113.1, 61.9, 61.6, 55.4, 51.3, 50.7, 49.0, 46.1, 46.1, 41.9, 29.9, 28.2, 25.6. HRMS (ESI-QTOF) m/z: $[M + H]^+$ Calcd for $C_{30}H_{38}ClF_2N_6O_2^+$: 587.2707; Observed: 587.2708. Enantiopurity determined to be 85.4%.

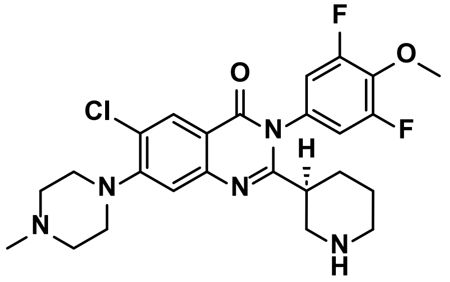

(*S*)-1m

(*S*)-6-chloro-3-(3,5-difluoro-4-methoxyphenyl)-7-(4-methylpiperazin-1-yl)-2-(piperidin-3-yl)quinazolin-4(3*H*)-one

(*S*)-1m (0.057 g, 75.4%) was prepared from **(*S*)-6m** (0.090 g, 0.15 mmol), dichloromethane (1.5 mL), trifluoroacetic acid (0.34 mL, 4.47 mmol) and saturated potassium carbonate solution (3 mL) in the same manner described for **protocol D**. Light orange solid. Less than 1% TFA contamination by ^{19}F NMR. ^{1}H NMR (400 MHz, $CDCl_3$) δ 8.11 (s, 1H), 7.18 (s, 1H), 6.85 – 6.74 (m, 2H), 4.06 (s, 3H), 3.28 – 3.16 (m, 4H), 3.07 (dd, *J* = 12.2, 3.9 Hz, 1H), 3.03 – 2.97 (m, 1H), 2.97 – 2.89 (m, 1H), 2.62 (dt, *J* = 9.5, 3.0 Hz, 5H), 2.45 (tt, *J* = 9.3, 4.3 Hz, 1H), 2.36 (s, 3H), 2.02 – 1.77 (m, 3H), 1.66 (dp, *J* = 13.5, 3.4 Hz, 1H), 1.28 (tt, *J* = 11.3, 6.9 Hz, 1H). ^{13}C NMR (101 MHz, $CDCl_3$) δ 161.0, 158.5, 157.1, 157.1, 157.0, 155.2, 154.7, 154.6, 154.5, 147.3, 137.8, 137.7, 137.5, 131.0, 130.9, 130.8, 128.6, 127.7, 117.3, 115.5, 113.5, 113.5, 113.4, 113.4, 113.3, 113.3, 113.2, 113.2, 61.9, 55.0, 51.0, 50.7, 46.2, 42.0, 30.0, 25.6. HRMS (ESI-QTOF) m/z: $[M + H]^+$ Calcd for $C_{25}H_{29}ClF_2N_5O_2^+$: 504.1972; Observed: 504.1967. Enantiopurity determined to be 94.4%.

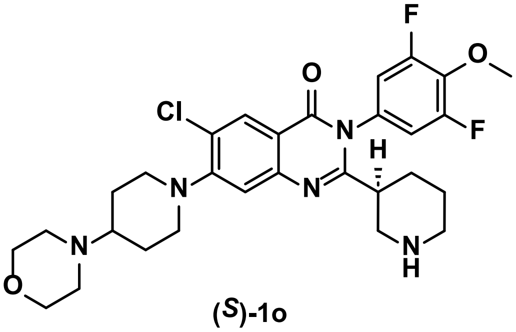

(*S*)-1o

(*S*)-6-chloro-3-(3,5-difluoro-4-methoxyphenyl)-7-(4-morpholinopiperidin-1-yl)-2-(piperidin-3-yl)quinazolin-4(3*H*)-one

(*S*)-1o (0.043 g, 80.5%) was prepared from **(*S*)-1o** (0.063 g, 0.093 mmol), dichloromethane (1.0 mL), trifluoroacetic acid (0.22 mL, 2.80 mmol) and saturated potassium carbonate solution (3 mL) in the same manner described for **protocol D**. Pale yellow solid. Less than 1% TFA contamination by ^{19}F NMR. ^{1}H NMR (400 MHz, $CDCl_3$) δ 8.12 (s, 1H), 7.19 (s, 1H), 6.86 – 6.76 (m, 2H), 4.08 (s, 3H), 3.73 (t, *J* = 4.5 Hz, 4H), 3.64 (d, *J* = 11.9 Hz, 2H), 3.08 (dd, *J* = 12.3, 3.9 Hz, 1H), 3.03 – 2.90 (m, 2H), 2.75 (td, *J* = 11.9, 2.2 Hz, 2H), 2.69 – 2.54 (m, 5H), 2.41 (dddt, *J* = 25.9, 11.0, 7.3, 3.9 Hz, 1H), 1.96 (dd, *J* = 13.0, 3.6 Hz, 2H), 1.92 – 1.62 (m, 5H), 1.36 – 1.19 (m, 2H). ^{13}C NMR (101 MHz, $CDCl_3$) δ 161.1, 158.4, 157.2, 157.1, 157.1, 155.6, 154.7, 154.6, 154.6, 147.3, 137.8, 137.7, 137.5, 131.1, 131.0, 130.8, 128.6, 127.9, 117.3, 115.4, 113.5, 113.5, 113.4, 113.4, 113.3, 113.3, 113.2, 113.2, 67.4, 62.0, 61.8, 51.0, 50.8, 49.9, 46.2, 42.0, 30.0, 28.4, 25.7. HRMS (ESI-QTOF) m/z: $[M + H]^+$ Calcd for $C_{29}H_{34}ClF_2N_5O_3^+$: 574.2391; Observed: 574.2373. Enantiopurity determined to be 92.6%.

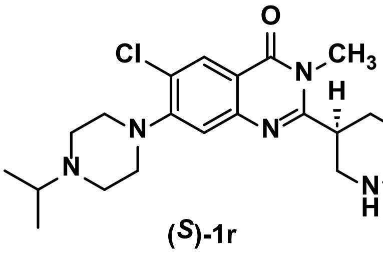

(*S*)-1r

(*S*)-6-chloro-7-(4-isopropylpiperazin-1-yl)-3-methyl-2-(piperidin-3-yl)quinazolin-4(3*H*)-one

(*S*)-1r (0.057 g, 84.7%) was prepared from **(*S*)-6r** (0.084 g, 0.17 mmol), dichloromethane (1.7 mL), trifluoroacetic acid (0.38 mL, 5.00 mmol) and saturated potassium carbonate solution (3 mL) in the same manner described for **protocol D**. Orange solid. Less than 1% TFA contamination by ^{19}F NMR. ^{1}H NMR (400 MHz, $CDCl_3$) δ 8.17 (d, *J* = 3.8 Hz, 1H), 7.12 (d, *J* = 2.5 Hz, 1H), 3.62 (d, *J* = 2.9 Hz, 3H), 3.31 – 3.15 (m, 5H), 3.11 – 2.91 (m, 3H), 2.72 (dt, *J* = 8.8, 4.0 Hz, 6H), 2.17 – 1.95 (m, 2H), 1.92 – 1.75 (m, 1H), 1.72 – 1.52 (m, 1H), 1.24 (d, *J* = 2.7 Hz, 1H), 1.09 (dd, *J* = 6.8, 2.8 Hz, 6H). ^{13}C NMR (101 MHz, $CDCl_3$) δ 161.5, 159.0, 154.7, 147.3, 128.5, 127.3, 117.0, 115.5, 56.0, 54.6, 52.2, 51.5, 50.7, 48.8, 46.6, 41.8, 41.3, 30.2, 29.9, 29.6, 26.0, 25.3, 18.7. HRMS

(ESI-QTOF) m/z: [M + H]$^+$ Calcd for $C_{21}H_{31}ClN_5O^+$: 404.2212; Observed: 404.2202. Enantiopurity determined to be >99%.

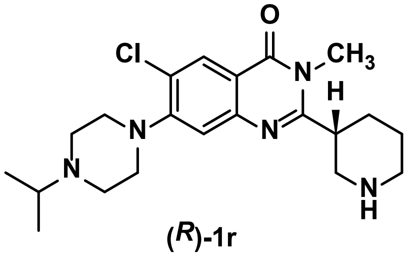

(*R*)-1r

(*R*)-6-chloro-7-(4-isopropylpiperazin-1-yl)-3-methyl-2-(piperidin-3-yl)quinazolin-4(3*H*)-one

(*R*)-1r (0.058 g, 82.2%) was prepared from **(*R*)-6r** (0.088 g, 0.18 mmol), dichloromethane (1.8 mL), trifluoroacetic acid (0.40 mL, 5.24 mmol) and saturated potassium carbonate solution (3 mL) in the same manner described for **protocol D**. Pale yellow solid. Less than 1% TFA contamination by ^{19}F NMR. ^{1}H NMR (400 MHz, $CDCl_3$) δ 8.15 (d, *J* = 1.3 Hz, 1H), 7.10 (s, 1H), 3.60 (t, *J* = 1.3 Hz, 3H), 3.19 (q, *J* = 6.5 Hz, 5H), 3.10 – 2.88 (m, 3H), 2.71 (dt, *J* = 9.3, 5.5 Hz, 6H), 2.04 (ddd, *J* = 26.9, 14.6, 8.5 Hz, 2H), 1.91 – 1.73 (m, 1H), 1.70 – 1.52 (m, 1H), 1.07 (dd, *J* = 6.6, 1.0 Hz, 6H). ^{13}C NMR (101 MHz, $CDCl_3$) δ 161.4, 159.0, 154.6, 147.3, 147.2, 128.4, 127.3, 116.9, 115.5, 56.0, 54.6, 52.2, 51.5, 50.6, 48.7, 46.5, 41.8, 41.3, 30.1, 29.8, 29.6, 26.0, 25.3, 18.7. HRMS (ESI-QTOF) m/z: [M + H]$^+$ Calcd for $C_{21}H_{31}ClN_5O^+$: 404.2212; Observed: 404.2204. Enantiopurity determined to be >99%.

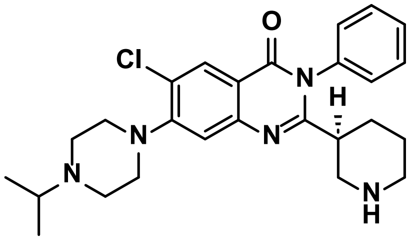

(*S*)-1s

(*S*)-6-chloro-7-(4-isopropylpiperazin-1-yl)-3-phenyl-2-(piperidin-3-yl)quinazolin-4(3*H*)-one

(*S*)-1s (0.076 g, 87.1%) was prepared from **(*S*)-6s** (0.106 g, 0.19 mmol), dichloromethane (1.9 mL), trifluoroacetic acid (0.43 mL, 5.62 mmol) and saturated potassium carbonate solution (3 mL) in the same manner described for **protocol D**. Light orange solid. Less than 1% TFA contamination by ^{19}F NMR. ^{1}H NMR (400 MHz, $CDCl_3$) δ 8.16 (s, 1H), 7.56 – 7.45 (m, 3H), 7.22 – 7.17 (m, 3H), 3.24 (t, *J* = 4.7 Hz, 4H), 3.07 (dd, *J* = 12.4, 3.9 Hz, 1H), 2.99 (dd, *J* = 12.3, 9.9 Hz, 1H), 2.89 (dt, *J* = 12.5, 3.4 Hz, 1H), 2.74 (q, *J* = 5.8 Hz, 5H), 2.62 (td, *J* = 12.2, 3.0 Hz, 1H), 2.41 (tt, *J* = 9.2, 4.2 Hz, 1H), 1.85 (td, *J* = 10.4, 3.9 Hz, 2H), 1.62 (dp, *J* = 13.4, 3.3 Hz, 1H), 1.51 (s, 1H), 1.26 – 1.13 (m, 1H), 1.10 (d, *J* = 6.5 Hz, 6H). ^{13}C NMR (101 MHz, $CDCl_3$) δ 161.3, 159.2, 155.0, 147.6, 137.1, 130.1, 129.9, 129.4, 128.7, 128.3, 127.4, 117.1, 115.9, 54.6, 51.5, 50.7, 48.7, 46.2,

42.2, 29.9, 25.9, 18.7. HRMS (ESI-QTOF) m/z: $[M + H]^+$ Calcd for $C_{26}H_{32}ClN_5O^+$: 466.2368; Observed: 466.2365. Enantiopurity determined to be 89.8%.

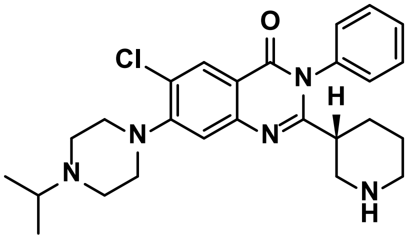

(*R*)-1s

(*R*)-6-chloro-7-(4-isopropylpiperazin-1-yl)-3-phenyl-2-(piperidin-3-yl)quinazolin-4(3*H*)-one

(*R*)-1s (0.041 g, 848.5%) was prepared from **(R)-6s** (0.056 g, 0.099 mmol), dichloromethane (1.0 mL), trifluoroacetic acid (0.23 mL, 2.97 mmol) and saturated potassium carbonate solution (2 mL) in the same manner described for **protocol D**. Light orange solid. Less than 1% TFA contamination by ^{19}F NMR. ^{1}H NMR (400 MHz, $CDCl_3$) δ 8.17 (s, 1H), 7.56 – 7.45 (m, 3H), 7.23 – 7.17 (m, 3H), 3.25 (t, *J* = 4.7 Hz, 4H), 3.12 – 3.05 (m, 1H), 3.00 (dd, *J* = 12.3, 9.9 Hz, 1H), 2.90 (dt, *J* = 12.4, 3.5 Hz, 1H), 2.75 (q, *J* = 5.8 Hz, 5H), 2.64 (td, *J* = 12.2, 3.0 Hz, 1H), 2.47 – 2.38 (m, 1H), 1.86 (ddd, *J* = 11.4, 9.8, 3.8 Hz, 2H), 1.63 (dp, *J* = 13.3, 3.2 Hz, 1H), 1.27 – 1.14 (m, 1H), 1.11 (d, *J* = 6.5 Hz, 6H). ^{13}C NMR (101 MHz, $CDCl_3$) δ 161.4, 159.2, 155.0, 147.7, 137.2, 130.1, 129.9, 129.4, 128.7, 128.4, 127.4, 117.1, 115.9, 54.6, 51.5, 50.8, 48.7, 46.3, 42.2, 29.9, 25.9, 18.7. HRMS (ESI-QTOF) m/z: $[M + H]^+$ Calcd for $C_{26}H_{32}ClN_5O^+$: 466.2368; Observed: 466.2366. Enantiopurity determined to be 95.3%.

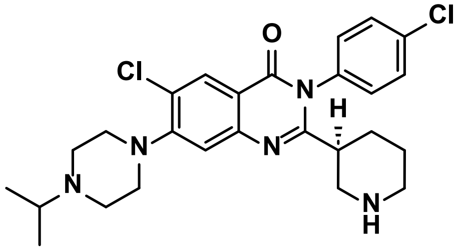

(*S*)-1v

(*S*)-6-chloro-3-(4-chlorophenyl)-7-(4-isopropylpiperazin-1-yl)-2-(piperidin-3-yl)quinazolin-4(3*H*)-one

(*S*)-1v (0.066 g, 99.0%) was prepared from **(*S*)-6v** (0.080 g, 0.13 mmol), dichloromethane (1.4 mL), trifluoroacetic acid (0.31 mL, 4.00 mmol) and saturated potassium carbonate solution (3 mL) in the same manner described for **protocol D**. Light orange solid. Less than 1% TFA contamination by ^{19}F NMR. ^{1}H NMR (400 MHz, $CDCl_3$) δ 8.16 (d, *J* = 0.7 Hz, 1H), 7.54 – 7.48 (m, 2H), 7.20 (s, 1H), 7.19 – 7.13 (m, 2H), 3.25 (t, *J* = 4.8 Hz, 4H), 3.07 (dd, *J* = 12.3, 4.0 Hz, 1H), 3.00 (dd, *J* = 12.3, 9.8 Hz, 1H), 2.93 (dt, *J* = 12.4, 3.6 Hz, 1H), 2.80 – 2.70 (m, 5H), 2.65 (td, *J* = 12.1, 2.9 Hz, 1H), 2.46 – 2.37 (m, 1H), 1.87 (dt, *J* = 9.7, 4.8 Hz, 2H), 1.65 (dt, *J* = 13.5, 3.3 Hz, 1H), 1.23 (qd, *J* = 8.8, 6.1 Hz, 2H), 1.11 (d, *J* = 6.5 Hz, 6H). ^{13}C NMR (101 MHz, $CDCl_3$) δ 161.3, 158.8, 155.2,

147.6, 135.7, 135.6, 130.5, 130.3, 129.8, 128.7, 127.6, 117.2, 115.7, 54.7, 51.5, 50.8, 48.7, 46.3, 42.2, 29.9, 25.8, 18.7. HRMS (ESI-QTOF) m/z: $[M + H]^+$ Calcd for $C_{26}H_{32}Cl_2N_5O^+$: 500.1978; Observed: 500.1964. Enantiopurity determined to be 91.7%.

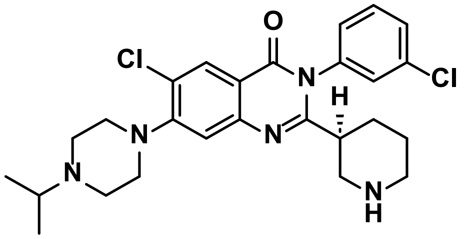

(*S*)-1w

(*S*)-6-chloro-3-(3-chlorophenyl)-7-(4-isopropylpiperazin-1-yl)-2-(piperidin-3-yl)quinazolin-4(3*H*)-one

(*S*)-1w (0.079 g, 89.3%) was prepared from **(*S*)-6w** (0.11 g, 0.18 mmol), dichloromethane (1.8 mL), trifluoroacetic acid (0.41 mL, 5.30 mmol) and saturated potassium carbonate solution (3 mL) in the same manner described for **protocol D**. Light orange solid. Less than 1% TFA contamination by ^{19}F NMR. ^{1}H NMR (400 MHz, $CDCl_3$) δ 8.14 (s, 1H), 7.49 – 7.42 (m, 2H), 7.22 (dq, *J* = 3.9, 1.3 Hz, 1H), 7.19 (s, 1H), 7.11 (ddt, *J* = 6.9, 3.8, 1.9 Hz, 1H), 3.24 (t, *J* = 4.8 Hz, 4H), 3.12 – 2.96 (m, 2H), 2.95 – 2.86 (m, 1H), 2.75 (dt, *J* = 9.7, 5.8 Hz, 5H), 2.65 (td, *J* = 12.1, 2.9 Hz, 1H), 2.47 – 2.37 (m, 2H), 1.84 (dddd, *J* = 15.5, 10.3, 5.4, 2.9 Hz, 2H), 1.65 (ddq, *J* = 13.9, 10.1, 3.3 Hz, 1H), 1.33 – 1.17 (m, 1H), 1.10 (d, *J* = 6.5 Hz, 6H). ^{13}C NMR (101 MHz, $CDCl_3$) δ 161.1, 161.1, 158.5, 158.4, 155.2, 147.4, 138.2, 138.2, 135.7, 135.5, 131.0, 130.8, 129.9, 129.8, 128.8, 128.7, 127.6, 126.8, 126.8, 117.1, 115.6, 54.6, 51.4, 50.6, 50.4, 48.7, 46.1, 41.8, 29.9, 29.6, 25.5, 18.7. HRMS (ESI-QTOF) m/z: $[M + H]^+$ Calcd for $C_{26}H_{32}Cl_2N_5O^+$: 500.1978; Observed: 500.1961. Enantiopurity determined to be 86.0%.

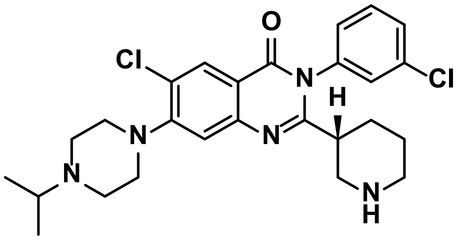

(*R*)-1w

(*R*)-6-chloro-3-(3-chlorophenyl)-7-(4-isopropylpiperazin-1-yl)-2-(piperidin-3-yl)quinazolin-4(3*H*)-one

(*R*)-1w (0.058 g, 89.5%) was prepared from **(*R*)-6w** (0.078 g, 0.13 mmol), dichloromethane (1.3 mL), trifluoroacetic acid (0.3 mL, 3.90 mmol) and saturated potassium carbonate solution (3 mL) in the same manner described for **protocol D**. Orange solid. Less than 1% TFA contamination by ^{19}F NMR. ^{1}H NMR (400 MHz, $CDCl_3$) δ 8.15 (s, 1H), 7.50 – 7.44 (m, 2H), 7.23 (dq, *J* = 4.0, 1.5 Hz, 1H), 7.20 (s, 1H), 7.12 (dhept, *J* = 7.2, 2.4 Hz, 1H), 3.24 (t, *J* = 4.7 Hz, 4H), 3.08 (ddd, *J* = 12.9, 8.2, 4.2 Hz, 1H), 3.03 – 2.94 (m, 1H), 2.91 (td, *J* = 9.5, 4.9 Hz, 1H), 2.75 (dt, *J* = 9.7, 5.7 Hz,

5H), 2.64 (ddd, *J* = 14.8, 8.0, 2.9 Hz, 1H), 2.40 (tt, *J* = 9.5, 4.3 Hz, 1H), 1.92 – 1.79 (m, 2H), 1.65 (dtt, *J* = 14.1, 6.6, 3.3 Hz, 1H), 1.24 (q, *J* = 6.3 Hz, 2H), 1.10 (d, *J* = 6.5 Hz, 6H). ^{13}C NMR (101 MHz, $CDCl_3$) δ 161.2, 158.6, 158.6, 155.2, 147.5, 138.3, 135.7, 135.6, 131.0, 130.8, 129.9, 129.8, 128.8, 128.8, 128.7, 127.6, 126.8, 126.8, 117.2, 115.6, 54.6, 51.4, 50.8, 50.6, 48.7, 46.2, 42.2, 42.1, 30.1, 29.8, 25.8, 18.7. HRMS (ESI-QTOF) m/z: $[M + H]^+$ Calcd for $C_{26}H_{32}Cl_2N_5O^+$: 500.1978; Observed: 500.1961. Enantiopurity determined to be 86.0%.

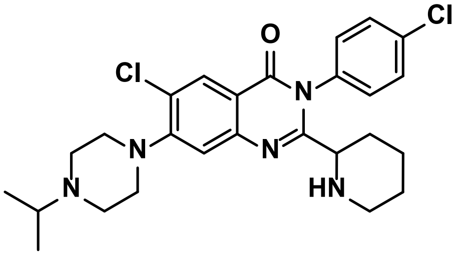

***rac*-1x**

6-chloro-3-(4-chlorophenyl)-7-(4-isopropylpiperazin-1-yl)-2-(piperidin-2-yl)quinazolin-4(3*H*)-one

***rac*-1x** (0.021 g, 42% yield) was prepared from ***rac*-6x** (0.061 g, 0.10 mmol), dichloromethane (1.0 mL), trifluoroacetic acid (0.23 mL, 3.04 mmol), and saturated potassium carbonate solution (3 mL) in the same manner described for **protocol D.** Tan solid. <1% TFA contamination. ^{1}H NMR (400 MHz, $CDCl_3$) δ 8.17 (s, 1H), 7.52 (ddd, *J* = 8.7, 5.2, 2.3 Hz, 2H), 7.32 – 7.24 (m, 2H), 7.18 (dd, *J* = 8.6, 2.6 Hz, 1H), 3.38 – 3.29 (m, 1H), 3.27 (dt, *J* = 9.2, 5.5 Hz, 4H), 3.14 (ddt, *J* = 13.5, 3.9, 1.9 Hz, 1H), 2.79 (dt, *J* = 8.1, 5.6 Hz, 5H), 2.47 (td, *J* = 13.6, 13.1, 3.0 Hz, 1H), 1.85 – 1.77 (m, 1H), 1.73 (dd, *J* = 12.9, 3.2 Hz, 1H), 1.60 – 1.50 (m, 1H), 1.54 – 1.46 (m, 1H), 1.39 (qt, *J* = 12.8, 4.0 Hz, 1H), 1.27 – 1.16 (m, 1H), 1.14 (d, *J* = 6.5 Hz, 6H). ^{13}C NMR (101 MHz, $CDCl_3$) δ 161.1, 158.3, 155.1, 147.5, 135.6, 135.1, 130.3, 130.2, 130.1, 129.5, 127.8, 117.4, 115.8, 58.1, 54.9, 51.2, 48.7 (2C), 46.2 (2C), 32.1, 26.4, 24.4, 18.7, 18.6. HRMS (ESI-QTOF) m/z: $[M + H]^+$ Calcd for $C_{26}H_{32}ClN_5O^+$: 500.1978; Observed: 500.1977.

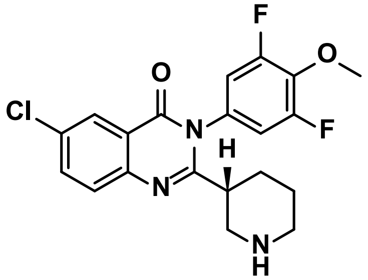

(*R*)-1ab

(*R*)-6-chloro-3-(3,5-difluoro-4-methoxyphenyl)-2-(piperidin-3-yl)quinazolin-4(3*H*)-one

(*R*)-1ab (0.13 g, 77.5%) was prepared from **(*R*)-4h** (0.21 g, 0.42 mmol), dichloromethane (4.3 mL), trifluoroacetic acid (0.97 mL, 12.73 mmol) and saturated potassium carbonate solution (6 mL) in the same manner described for **protocol D**. Light brown solid. 1.5% TFA contamination

by ^{19}F NMR. ^{1}H NMR (400 MHz, $CDCl_3$) δ 8.14 (dd, *J* = 3.8, 1.9 Hz, 1H), 7.67 (dq, *J* = 8.9, 2.2 Hz, 1H), 7.62 (dt, *J* = 8.7, 1.4 Hz, 1H), 6.84 (ddd, *J* = 10.3, 4.2, 2.1 Hz, 2H), 4.09 (q, *J* = 1.1 Hz, 3H), 3.09 (dd, *J* = 12.3, 3.9 Hz, 1H), 3.05 – 3.00 (m, 1H), 2.96 (dt, *J* = 12.6, 4.0 Hz, 1H), 2.66 (td, *J* = 12.2, 2.9 Hz, 1H), 2.48 (tt, *J* = 9.3, 4.4 Hz, 1H), 1.92 – 1.83 (m, 2H), 1.77 – 1.65 (m, 2H), 1.30 (dtd, *J* = 20.3, 10.8, 4.0 Hz, 1H). ^{13}C NMR (101 MHz, $CDCl_3$) δ 161.4, 158.1, 157.2, 157.2, 157.1, 154.8, 154.7, 154.6, 145.9, 138.0, 137.8, 137.7, 135.2, 132.8, 130.8, 130.7, 130.6, 129.2, 126.3, 121.6, 113.5, 113.4, 113.4, 113.4, 113.3, 113.2, 113.2, 113.1, 62.0, 50.8, 46.2, 42.0, 30.0, 25.7. HRMS (ESI-QTOF) m/z: $[M + H]^+$ Calcd for $C_{20}H_{19}ClF_2N_3O_2^+$: 406.1128; Observed: 406.1131. Enantiopurity determined to be 95.5%.

Protocol E (refer to Scheme S2)[4]:

Secondary amine was dissolved in a 1:1 mixture of ACS grade dichloromethane and methanol. Acetic acid was added dropwise over 1 min, then 37% formaldehyde was added dropwise over 1 min, and the reaction mixture was cooled to 0 °C using an ice-water bath for 15 min. Sodium cyanoborohydride was slowly added to the reaction mixture portion-wise over 1 min. The reaction was removed from the ice-water bath and allowed to reach rt and stirred for 16 h. The reaction mixture was concentrated *in vacuo*. The residue was neutralized with sat. sodium bicarbonate solution (5 mL) and then extracted three times with dichloromethane (3 x 40 mL). The organic layers were combined and washed once with sat. brine solution (70 mL). The organic layer was dried over anhydrous sodium sulfate, filtered, and the solvent was evaporated under reduced pressure. The residue was purified by flash chromatography (0% methanol / dichloromethane ~ 10% methanol / dichloromethane) to afford methylated product.

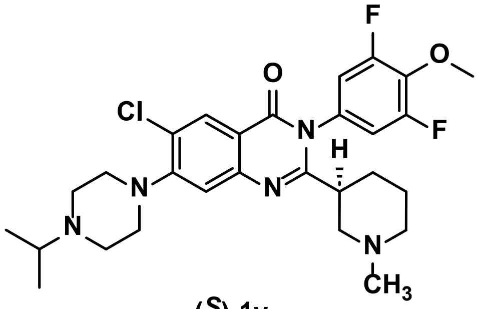

(*S*)-1y

(*S*)-6-chloro-3-(3,5-difluoro-4-methoxyphenyl)-7-(4-isopropylpiperazin-1-yl)-2-(1-methylpiperidin-3-yl)quinazolin-4(3*H*)-one

(*S*)-1z (0.023 g, 74.7%) was prepared from **(S)-1g** (0.030 g, 0.056 mmol), dichloromethane (1.1 mL), methanol (1.1 mL), acetic acid (10.6 μL, 0.19 mmol), 37% formaldehyde (9.2 μL, 0.11 mmol), and sodium cyanoborohydride (0.005 g, 0.085 mmol) in the same manner described for **protocol E**. Light yellow solid. ^{1}H NMR (400 MHz, $CDCl_3$) δ 8.15 (s, 1H), 7.19 (s, 1H), 6.84 – 6.77 (m, 2H), 4.08 (s, 3H), 3.25 (t, *J* = 4.6 Hz, 4H), 2.92 – 2.85 (m, 1H), 2.75 (t, *J* = 5.0 Hz, 6H), 2.64 (tt, *J* = 11.1, 3.6 Hz, 1H), 2.39 (t, *J* = 10.9 Hz, 1H), 2.28 (s, 3H), 1.97 (td, *J* = 11.8, 2.6 Hz,

1H), 1.83 (d, J = 12.2 Hz, 1H), 1.75 – 1.56 (m, 2H), 1.42 (ddq, J = 17.7, 9.0, 4.7 Hz, 1H), 1.11 (d, J = 6.5 Hz, 6H). ^{13}C NMR (101 MHz, $CDCl_3$) δ 161.2, 158.3, 157.3, 157.2, 157.2, 157.1, 155.3, 154.8, 154.7, 154.7, 154.6, 147.4, 137.9, 137.8, 137.6, 130.9, 130.8, 130.7, 128.8, 127.8, 117.2, 115.5, 113.6, 113.6, 113.4, 113.4, 113.3, 113.1, 113.1, 62.0, 62.0, 61.9, 59.4, 55.4, 54.7, 51.4, 48.7, 46.5, 41.3, 29.4, 25.1, 18.7. HRMS (ESI-QTOF) m/z: $[M + H]^+$ Calcd for $C_{28}H_{35}ClF_2N_5O_2^+$: 546.2442; Observed: 546.2430. Enantiopurity determined to be >99%.

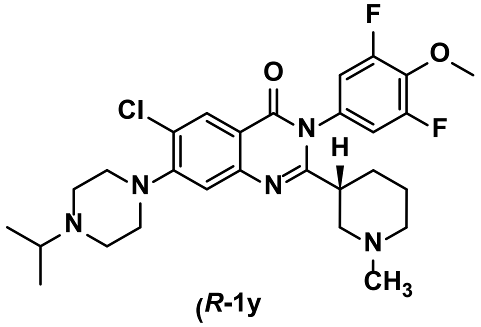

(*R*-1y

(*R*)-6-chloro-3-(3,5-difluoro-4-methoxyphenyl)-7-(4-isopropylpiperazin-1-yl)-2-(1-methylpiperidin-3-yl)quinazolin-4(3*H*)-one

(*R*)-1y (0.027 g, 51.7%) was prepared from **(*R*)-1g** (0.076 g, 0.10 mmol), dichloromethane (2.0 mL), methanol (2.0 mL), acetic acid (18.8 μL, 0.33 mmol), 37% formaldehyde (16.3 μL, 0.20 mmol), and sodium cyanoborohydride (0.009 g, 0.15 mmol) in the same manner described for **protocol E**. Pale yellow solid. ^{1}H NMR (400 MHz, $CDCl_3$) δ 8.15 (s, 1H), 7.19 (s, 1H), 6.84 – 6.77 (m, 2H), 4.08 (d, J = 1.2 Hz, 3H), 3.25 (t, J = 4.8 Hz, 4H), 2.89 (ddt, J = 11.1, 3.5, 1.6 Hz, 1H), 2.82 – 2.71 (m, 5H), 2.64 (tt, J = 11.1, 3.5 Hz, 1H), 2.39 (t, J = 10.9 Hz, 1H), 2.28 (s, 3H), 1.97 (td, J = 11.8, 2.7 Hz, 1H), 1.83 (d, J = 13.0 Hz, 1H), 1.74 – 1.66 (m, 1H), 1.66 – 1.56 (m, 1H), 1.42 (dddd, J = 17.0, 12.9, 8.7, 4.1 Hz, 1H), 1.12 (d, J = 6.5 Hz, 6H). ^{13}C NMR (101 MHz, $CDCl_3$) δ 161.2, 158.3, 157.3, 157.2, 157.2, 157.1, 155.3, 154.8, 154.7, 154.7, 154.6, 147.4, 137.9, 137.8, 137.6, 130.9, 130.8, 130.7, 128.8, 127.8, 117.2, 115.5, 113.6, 113.6, 113.4, 113.4, 113.3, 113.1, 113.1, 62.0, 62.0, 61.9, 59.4, 55.4, 54.7, 51.4, 48.7, 46.5, 41.3, 29.4, 25.1, 18.7. HRMS (ESI-QTOF) m/z: $[M + H]^+$ Calcd for $C_{28}H_{35}ClF_2N_5O_2^+$: 546.2442; Observed: 546.2437. Enantiopurity determined to be >99%.

Protocol F (refer to Scheme S2):

To an oven-dried, inert gas-cooled flask was added the quinazolinone, bromobenzene, $Pd_2(dba)_3$, Xantphos, cesium carbonate, and anhydrous toluene. The reaction mixture was sparged with N_2 for 5 min, heated to 100 °C, and allowed to stir for 24 h. After cooling to rt, the mixture was diluted with ethyl acetate (60 mL) and washed three times with sat. brine solution (3 x 40 mL). The organic layer was dried over anhydrous sodium sulfate, filtered, and the solvent was evaporated under reduced pressure. The residue was purified by flash chromatography (0% methanol / dichloromethane ~ 10% methanol / dichloromethane) to afford the benzylated product.

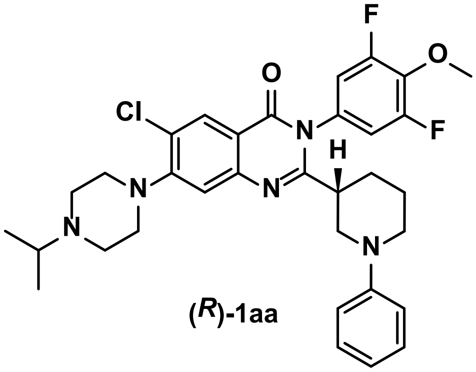

(*R*)-1aa

(*R*)-6-chloro-3-(3,5-difluoro-4-methoxyphenyl)-7-(4-isopropylpiperazin-1-yl)-2-(1-phenylpiperidin-3-yl)quinazolin-4(3*H*)-one

(R)-1aa (0.014 g, 36.1%) was prepared from **(*R*)-1g** (0.033 g, 0.062 mmol), bromobenzene (6.15 μL, 0.059 mmol), $Pd_2(dba)_3$ (0.003 g, 0.003 mmol), Xantphos (0.003 g, 0.006 mmol), cesium carbonate (0.027 g, 0.083 mmol), and anhydrous toluene (0.75 mL) in the same manner described for **protocol F**. Yellow solid. Less than 1% TFA contamination by ^{19}F NMR. ^{1}H NMR (400 MHz, $CDCl_3$) δ 8.18 (s, 1H), 7.25 – 7.19 (m, 3H), 6.89 (dt, *J* = 10.3, 2.2 Hz, 1H), 6.84 (dd, *J* = 4.2, 1.7 Hz, 1H), 6.80 (dd, *J* = 8.5, 1.8 Hz, 3H), 4.08 (d, *J* = 1.2 Hz, 3H), 3.74 (dt, *J* = 12.7, 2.4 Hz, 1H), 3.67 (dt, 1H), 3.28 (d, *J* = 5.7 Hz, 4H), 3.17 (dd, *J* = 12.5, 10.7 Hz, 1H), 2.83 – 2.76 (m, 5H), 2.74 – 2.66 (m, 1H), 1.95 (td, *J* = 9.5, 3.7 Hz, 2H), 1.76 (dt, *J* = 13.3, 3.0 Hz, 1H), 1.61 – 1.47 (m, 1H), 1.30 – 1.22 (m, 1H), 1.13 (d, *J* = 6.5 Hz, 6H). ^{13}C NMR (101 MHz, $CDCl_3$) δ 161.2, 158.0, 157.2, 155.3, 154.8, 150.8, 147.4, 137.8, 132.3, 132.2, 132.1, 131.0, 130.9, 130.7, 129.3, 128.8, 128.7, 128.6, 127.9, 119.8, 117.3, 116.7, 115.6, 113.6, 113.6, 113.5, 113.4, 113.4, 113.3, 113.2, 62.0, 54.8, 54.2, 51.3, 49.9, 48.7, 40.5, 30.0, 24.2, 18.7. HRMS (ESI-QTOF) m/z: $[M + H]^+$ Calcd for $C_{33}H_{37}ClF_2N_5O_2^+$: 608.2598; Observed: 608.2575. Enantiopurity determined to be 90.0%.

Scheme S3. Stepwise Formation of Quinazolinone Intermediates

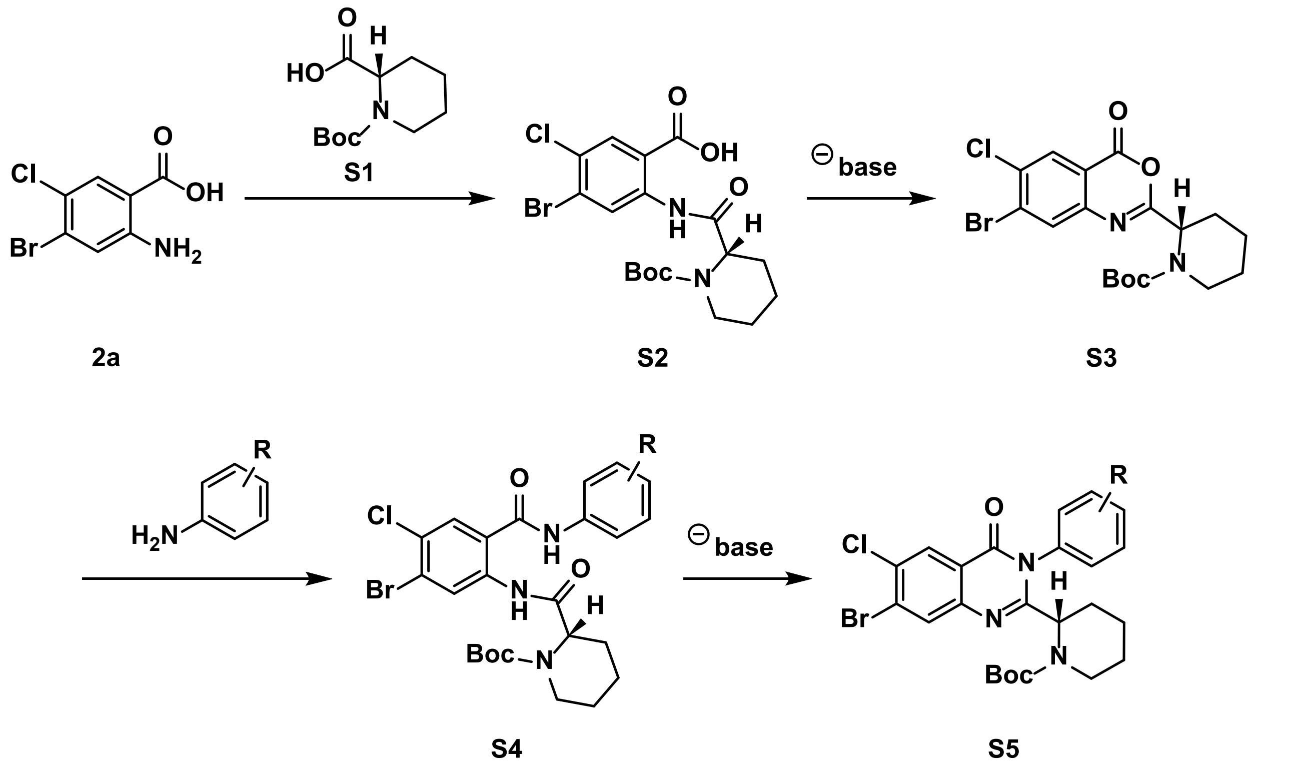

Scheme S4. Synthetic Route to 2-Piperidin-2-yl Quinazolinone Intermediates

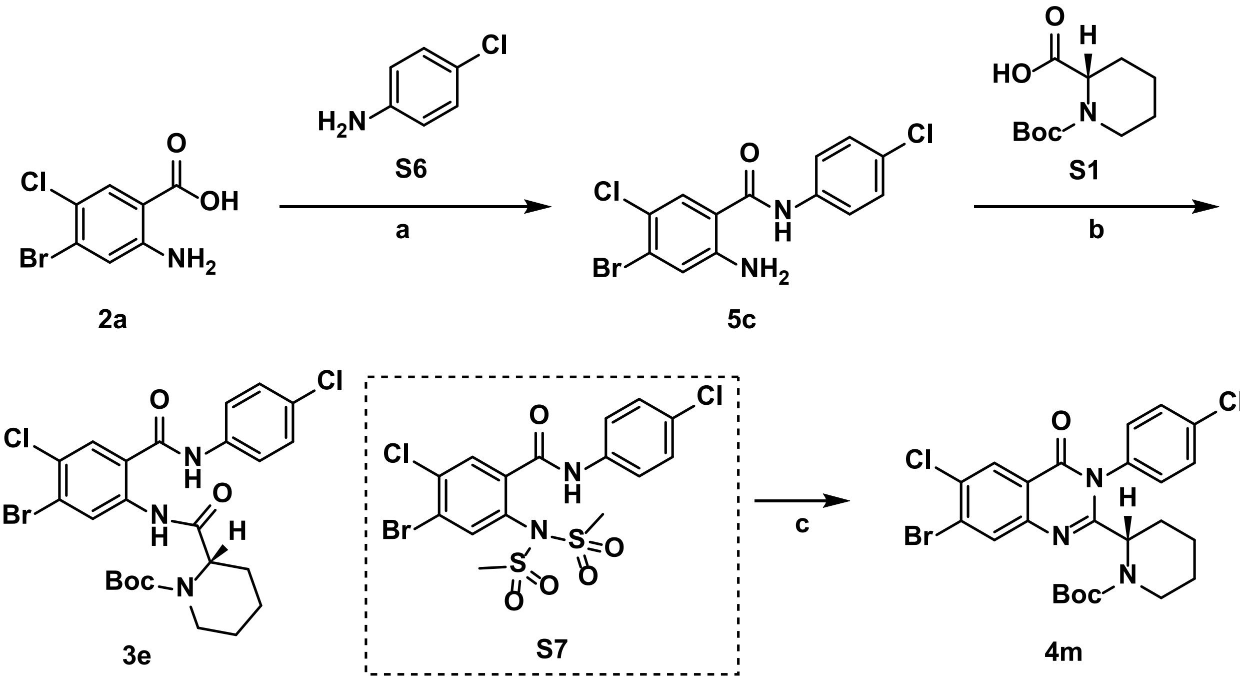

Table S1. Methods Screened to Yield 2-piperidin-2-yl Quinazolinones

	(a)	(b)	(c)	Outcome
**1**	HATU (1.2 eq), TEA (1.5 eq), DMF, rt, 16 h	-	-	70-85% yield of **5c**
**2**	-	MsCl (1.47 eq), NMI (4 eq). $CuCl_2$, DCM, rt, 24 h	-	Recollected **5c** and deprotected **3e**
**3**	-	MsCl (4 eq), NMI (9 eq). $CuCl_2$, DCM, rt, 24 h	-	Collected bis-mesylated amine **S7**
**4**	-	MsCl (1.4 eq), NMI (4 eq). $CuCl_2$, DCM, rt, 120 h	-	Collected deprotected **3e**
**5**	-	HATU (1.2 eq), TEA (2 eq), DMF, rt, 48h	-	No reaction
**6**[5]	-	COMU (1 eq), DIPEA (2 eq), DMF, rt, 48 h	-	No reaction
**7**[6]	-	Collidine (3 eq), TCFH (2 eq), DCM, rt, 24 h	-	No reaction
**8**[7]	-	NMI (2.1 eq), TCFH (1.1 eq), MeCN, rt, 24 h	-	No reaction
**9**	-	PyAOP (1 eq), TEA (2 eq), DCM, rt, 96 h	-	38% yield of **3e**, 70%ee
**10**[8]	-	PyBrOP (2 eq), DIPEA (6 eq, DCM, rt, 48 h	-	30% yield of **3e**, 90%ee
**11**	-	PyBrOP (2.3 eq), DIPEA (6 eq), DCM, rt, 48 h	-	30% yield of **3e**, 91%ee
**12**	-	PyBrOP (2 eq), DIPEA (6 eq), DCM, 40°C, 48 h	-	30% yield of **3e**, 93%ee
**13**[9]	-	PyBrOP (1.1 eq) , TEA (2 eq), DCE, 120°C, MWI, 20 min	-	No reaction
**14**	-	T3P (1 eq), TEA (2 eq), DCE, rt, 144 h	-	13% yield of **3e**
**15**[10]	-	$POCl_3$ (0.83 eq), TEA (2.9 eq), DMAP (0.3 eq), DCM, rt, 24 h	-	25% yield of **3e**, 99% ee
**16**	-	-	TEA (8.7 eq), TMSCl (6.8 eq), DCE, 85°C, 24 h	No reaction
**17**[2]	-	-	N,O-bis (trimethylsilyl) acetamide (15 eq), TEA (30 eq), MeCN, 85°C, 24 h	60-80% yield of **4m**, 98-99%ee

Scheme S5. Coupling Method Using $POCl_3$

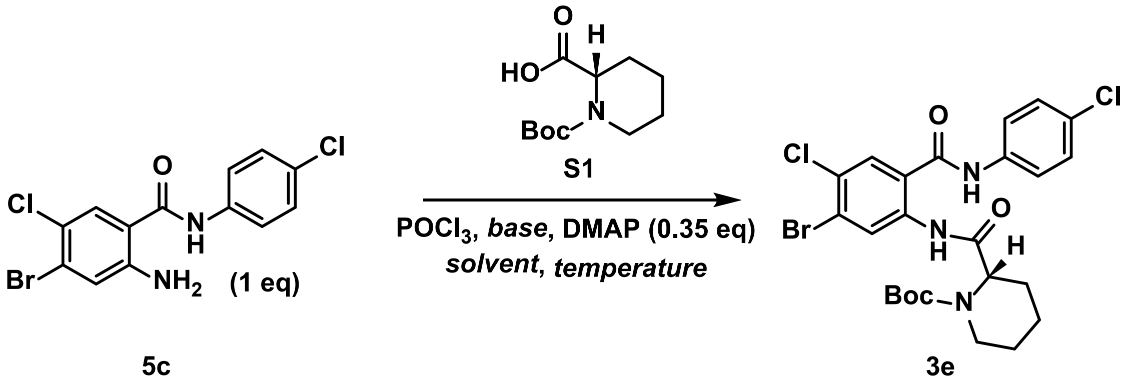

Table S2. Optimization of $POCl_3$ Coupling Step

	S1 (eq)	Base (2.9 eq)	$POCl_3$ (eq)	Solvent	Temp	3e (% yield)	%ee	Notes
**1**	0.83	TEA[a]	0.83	DCM	RT	25	99	-
**2**	0.83	TEA[a]	0.83	DCM	42°C	17	98	-
**3**	1.2	TEA	1.2	DCM	RT	27	96	-
**4**	1.2	DMAP	1.2	DCM	RT	-	-	1:1 **5c**:**3e**[b]
**5**	1.2	Pyridine	1.2	DCM	RT	-	-	1:1 side product:**3e**[b]
**6**	1.2	DBU	1.2	DCM	RT	-	-	4:1 **5c**:**3e**[b]
**7**	1.2	DIPEA	1.2	DCM	RT	-	-	1:1 **S8**:**S6**[b]
**8**	1.2	NMI	1.2	DCM	RT	6	-	Large product peak[b] not evidenced in collection
**9**	1.2	NMI	1.2	DCE	RT	12	-	Large product peak[b] not evidenced in collection
**10**	1.2	TEA	1.2	THF	RT	-	-	9:1 **S8**:**S6**[b]
**11**	1.2	TEA	1.2	Toluene	RT	-	-	1:1 side product:**S6**[b]
**12**	1.2	TEA	1.2	MeCN	RT	-	-	5:1 **S8**:**S6**[b]
**13**	1.2	TEA	1.2	DCE	RT	33	94	-
**14**	1.2	TEA	1.2	DCE	85°C	-	-	Degradation[b]
**15**	1.2	TEA[c]	1.2 + 1.2[d]	DCM	RT	-	-	Forms side product[b]
**16**	1.2	TEA[c]	1.2 + 1.2[d]	DCE	RT	-	-	Forms side product[b]
**17**	2	TEA[e]	2	DCM	RT	31	95	80% consumption of **S8**[b]
**18**	2	TEA[e]	2	DCE	RT	29	97	100% consumption of **S8**[b]
**19**	2	TEA[e]	2	DCE	RT	33 (27)[f]	97 (88)[f]	No work-up
**20**	2	TEA[e]	2	DCE	RT	-	-	Analysis of work-up shows product in saturated sodium bicarbonate wash
**21**	2	TEA[e]	2	DCE	RT	38 (10)[g]	96 (88)[g]	Extract sodium bicarbonate wash with 20% IPA in chloroform
**22**	2	TEA[e]	2	DCE	RT	36	97	Skip saturated sodium bicarbonate wash

[a]2 equivalents, [b]by LCMS, [c] 2.9 + 2.9 eq after 24 h, [d] after 24 h, [e]4.8 eq, [f]second product peak observed on column, [g]Extracted from quenched sodium bicarbonate wash

Scheme S6. Synthetic Route to Enantioenriched 2-(Piperidin-2-yl) Quinazolinones

(Reproduced from Scheme 3 in main manuscript)

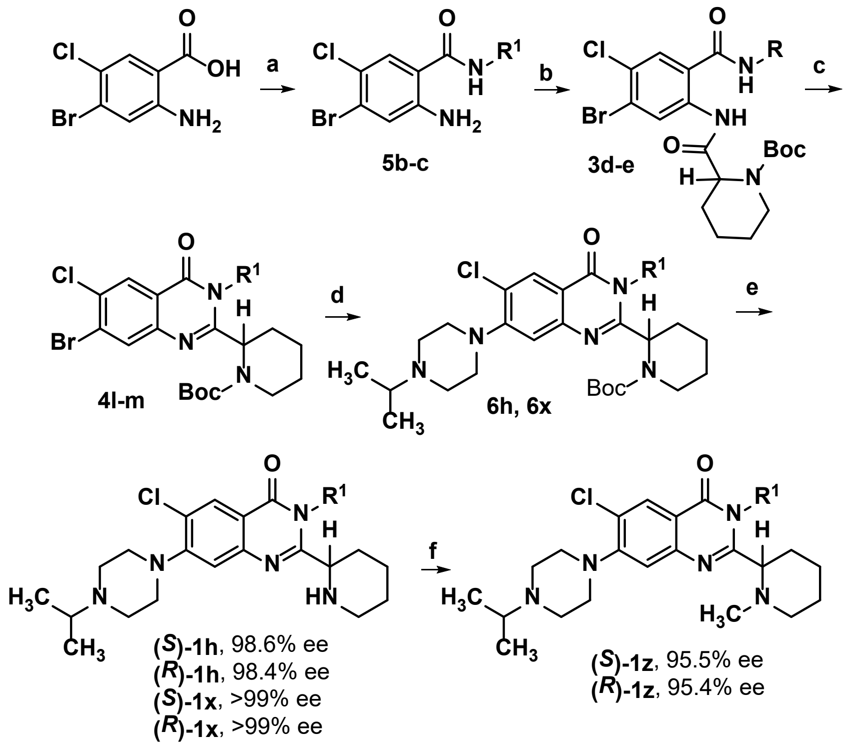

Protocol A (refer to Scheme S6):

Anthranilic acid, HATU, and triethylamine were added to dimethylformamide in a flask and cooled to 0 °C using an ice-water bath and allowed to stir for 15 min. Aniline was added to the reaction mixture, the ice-water bath was removed, and the reaction was allowed to reach room temperature and stirred for 24 h. The resulting mixture was slowly dripped into rapidly stirring water (600 mL). The resulting precipitate was isolated via vacuum filtration in a fritted funnel. The precipitate was then dissolved with ethyl acetate (150 mL) and washed once with sat. brine solution (80 mL). The organic layer was dried over anhydrous sodium sulfate, filtered, and the solvent was evaporated under reduced pressure to afford anthranilic amide product.

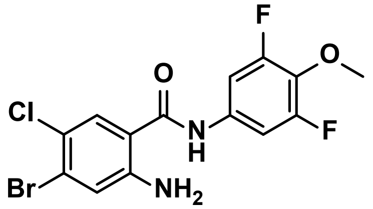

5b

2-amino-4-bromo-5-chloro-*N*-(3,5-difluoro-4-methoxyphenyl)benzamide

5b (2.36 g, 75.3%) was prepared from 2-amino-4-bromo-5-chlorobenzoic acid (2.00 g, 8.00 mmol), HATU (3.65 g, 9.60 mmol), triethylamine (2.23 mL, 16.00 mmol), dimethylformamide (32 mL), and 3,5-difluoro-4-methoxyaniline (1.91 g, 12.00 mmol) in the same manner described for **protocol A**. Brown solid. ^{1}H NMR (400 MHz, DMSO) δ 10.31 (s, 1H), 7.82 (s, 1H), 7.54 – 7.45 (m, 2H), 7.18 (s, 1H), 6.64 (s, 2H), 3.88 (s, 3H). ^{13}C NMR (101 MHz, DMSO) δ 166.0, 156.0, 155.9, 153.6, 153.5, 149.5, 134.6, 134.5, 134.3, 131.7, 131.6, 131.4, 129.6, 125.4, 120.4, 117.5, 114.9, 104.6, 104.5, 104.4, 104.3, 62.0.

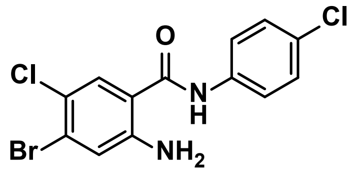

5c

2-amino-4-bromo-5-chloro-*N*-(4-chlorophenyl)benzamide

5c (2.57 g, 89.2%) was prepared from 2-amino-4-bromo-5-chlorobenzoic acid (2.00 g, 8.00 mmol), HATU (3.65 g, 9.60 mmol), triethylamine (2.23 mL, 16.00 mmol), dimethylformamide (32 mL), and 4-chloroaniline (**S6**) (1.53 g, 12.00 mmol) in the same manner described for **protocol A**. Brown solid. ^{1}H NMR (400 MHz, DMSO) δ 10.25 (s, 1H), 7.85 (s, 1H), 7.76 – 7.69 (m, 2H), 7.43 – 7.37 (m, 2H), 7.17 (s, 1H), 6.62 (s, 2H). ^{13}C NMR (101 MHz, DMSO) δ 165.9, 149.4, 137.8, 129.7, 128.5, 127.4, 125.2, 122.1, 120.3, 117.5, 115.3.

Protocol B (refer to Scheme S6)[10]:

To an oven-dried vial under nitrogen atmosphere was added anthranilic amide and Boc-2-piperidine carboxylic acid. Anhydrous dichloroethane and triethylamine were added to the vial, and the reaction mixture was allowed to stir at rt for 5 min. DMAP was dissolved in a small amount of anhydrous dichloroethane in a separate vessel and subsequently added to the reaction mixture and allowed to stir at rt for 15 min. Phosphorous oxychloride was dissolved in a small amount of anhydrous dichloroethane in a separate vessel and subsequently added to the reaction mixture and allowed to stir at rt for 24 h. The reaction mixture was diluted with dichloromethane (80 mL) and washed sequentially with cold water (60 mL), 1 N HCl (60 mL), and sat. brine solution (60 mL). The organic layer was dried over anhydrous sodium sulfate, filtered, and the solvent was evaporated under reduced pressure. The residue was purified by reverse-phase chromatography (20% methanol / water ~ 100% methanol) to afford diamide product.

Caution: Phosphorus oxychloride ($POCl_3$) is corrosive, water reactive, and causes skin, eye, and lung irritation. It is toxic by inhalation or if swallowed. Use is restricted to a chemical fume hood and should be handled with suitable protective eyewear, clothing, and gloves.

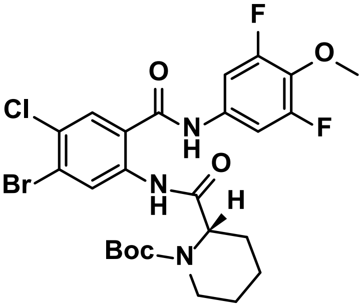

(*S*)-3d
***tert*-butyl (*S*)-2-((5-bromo-4-chloro-2-((3,5-difluoro-4-methoxyphenyl)carbamoyl)phenyl)carbamoyl)piperidine-1-carboxylate**

(*S*)-3d (0.13 g, 26.1%) was prepared from **5b** (0.31 g, 0.80 mmol), (*S*)-Boc-2-piperidine carboxylic acid (**S1**) (0.22 g, 0.96 mmol), phosphorous oxychloride (89.8 μL, 0.96 mmol), triethylamine (0.32 mL, 2.30 mmol), DMAP (0.035 g, 0.29 mmol), and anhydrous dichloromethane (8 mL) in the same manner described for **protocol B**. Light brown solid. ^{1}H NMR (400 MHz, $CDCl_3$) δ 11.30 (s, 1H), 8.95 (s, 1H), 8.42 (s, 1H), 7.63 (s, 1H), 7.36 – 7.27 (m, 2H), 4.93 (s, 1H), 4.19 – 4.07 (m, 1H), 3.99 (s, 3H), 2.98 (s, 1H), 2.35 (d, J = 13.3 Hz, 1H), 1.76 – 1.56 (m, 2H), 1.55 – 1.41 (m, 12H). ^{13}C NMR (101 MHz, $CDCl_3$) δ 171.0, 165.4, 157.0, 156.9, 154.5, 154.4, 138.6, 133.8, 128.5, 127.8, 126.1, 105.4, 105.3, 105.2, 105.1, 81.0, 62.2, 55.7, 42.8, 29.8, 28.5, 26.0, 24.9, 20.8.

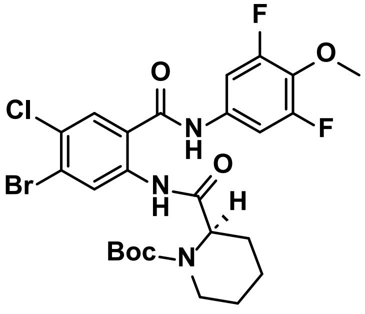

(*R*)-3d
***tert*-butyl (*R*)-2-((5-bromo-4-chloro-2-((3,5-difluoro-4-methoxyphenyl)carbamoyl)phenyl)carbamoyl)piperidine-1-carboxylate**

(*R*)-3d (0.055 g, 30.4%) was prepared from **5b** (0.12 g, 0.30 mmol), (*R*)-Boc-2-piperidine carboxylic acid (0.14 g, 0.60 mmol), phosphorous oxychloride (56.1 μL, 0.60 mmol), triethylamine (0.20 mL, 1.44 mmol), DMAP (0.022 g, 0.18 mmol), and anhydrous dichloroethane (3 mL) in the same manner described for **protocol B**. Light brown solid. ^{1}H NMR (400 MHz, $CDCl_3$) δ 11.29 (s, 1H), 8.97 (d, J = 1.9 Hz, 1H), 8.36 (s, 1H), 7.37 – 7.27 (m, 2H), 4.93 (s, 1H), 4.19 – 4.08 (m, 1H), 3.99 (s, 3H), 2.97 (s, 1H), 2.40 – 2.31 (m, 1H), 1.80 – 1.55 (m, 3H), 1.53 – 1.41 (m, 12H). ^{13}C NMR (101 MHz, $CDCl_3$) δ 171.0, 165.4, 157.0, 156.9, 154.5, 154.5, 138.6, 128.5, 127.8, 126.2, 105.4, 105.3, 105.2, 105.1, 81.0, 62.2, 55.8, 28.5, 25.9, 24.9, 20.8.

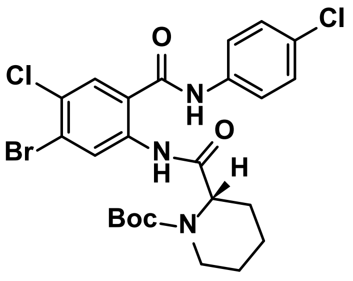

(*S*)-3e

***tert*-butyl (*S*)-2-((5-bromo-4-chloro-2-((4-chlorophenyl)carbamoyl)phenyl)carbamoyl)piperidine-1-carboxylate**

(*S*)-3e (0.13 g, 28.4%) was prepared from **5c** (0.29 g, 0.80 mmol), (*S*)-Boc-2-piperidine carboxylic acid (**S1**) (0.22 g, 0.96 mmol), phosphorous oxychloride (89.8 μL, 0.96 mmol), triethylamine (0.32 mL, 2.30 mmol), DMAP (0.035 g, 0.29 mmol), and anhydrous dichloromethane (8 mL) in the same manner described for **protocol B**. Light brown solid. ^{1}H NMR (400 MHz, $CDCl_3$) δ 11.28 (s, 1H), 8.98 (s, 1H), 8.29 (d, *J* = 44.0 Hz, 1H), 7.66 (s, 1H), 7.61 – 7.54 (m, 2H), 7.35 – 7.27 (m, 2H), 4.94 (s, 1H), 4.11 (s, 1H), 2.95 (s, 1H), 2.35 (dd, *J* = 13.5, 3.3 Hz, 1H), 1.74 – 1.57 (m, 3H), 1.50 – 1.36 (m, 11H). ^{13}C NMR (101 MHz, $CDCl_3$) δ 171.0, 165.4, 138.5, 135.8, 130.5, 129.2, 128.4, 127.9, 127.4, 126.2, 122.4, 80.8, 55.7, 42.8, 29.8, 28.5, 25.9, 24.9, 20.8.

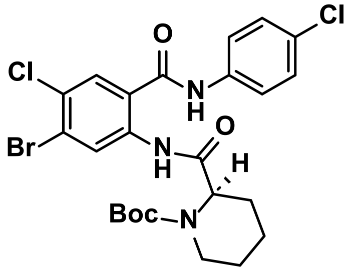

(*R*)-3e

***tert*-butyl (*R*)-2-((5-bromo-4-chloro-2-((4-chlorophenyl)carbamoyl)phenyl)carbamoyl)piperidine-1-carboxylate**

(*R*)-3e (0.12 g, 26.7%) was prepared from **5c** (0.29 g, 0.80 mmol), (*R*)-Boc-2-piperidine carboxylic acid (0.22 g, 0.96 mmol), phosphorous oxychloride (89.8 μL, 0.96 mmol), triethylamine (0.32 mL, 2.30 mmol), DMAP (0.035 g, 0.29 mmol), and anhydrous dichloromethane (8 mL) in the same manner described for **protocol B**. Light brown solid. ^{1}H NMR (400 MHz, $CDCl_3$) δ 11.26 (s, 1H), 9.00 (s, 1H), 8.23 (s, 1H), 7.65 (s, 1H), 7.58 (d, *J* = 8.5 Hz, 2H), 7.32 (d, *J* = 8.4 Hz, 2H), 4.95 (s, 1H), 4.12 (s, 1H), 2.94 (s, 1H), 2.36 (d, *J* = 13.1 Hz, 1H), 1.74 – 1.53 (m, 3H), 1.51 – 1.37 (m, 11H). ^{13}C NMR (101 MHz, $CDCl_3$) δ 171.0, 165.3, 138.5, 135.8, 130.6, 129.2, 128.4, 127.8, 127.5, 126.3, 122.3, 80.8, 55.7, 42.7, 29.8, 28.5, 25.9, 25.0, 20.8.

Protocol C (refer to Scheme S6)[2]:

To oven-dried, inert gas-cooled glassware was added diamide and acetonitrile. Triethylamine was added to the reaction mixture and the vessel was purged with nitrogen and stirred at rt for 20 min. *N,O*-bis(trimethylsilyl)acetamide was added to the reaction mixture over 1 min. The reaction was then heated to 85 °C and allowed to stir for 20 h. After cooling to rt, the reaction mixture was concentrated *in vacuo*. The residue was diluted with ethyl acetate (120 mL) and washed two times with water (2x 80 mL) and once with sat. brine solution (80 mL). The organic layer was dried over anhydrous sodium sulfate, filtered, and the solvent was evaporated under reduced pressure. The residue was purified by flash chromatography (0% 3:1 dichloromethane : ethyl acetate / hexanes ~ 15% 3:1 dichloromethane : ethyl acetate / hexanes) to afford quinazolinone product.

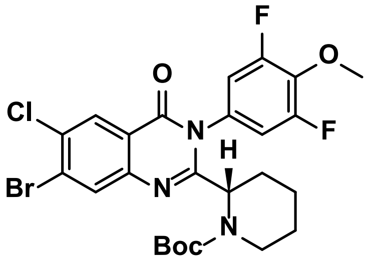

(*S*)-4l

***tert*-butyl (*S*)-2-(7-bromo-6-chloro-3-(3,5-difluoro-4-methoxyphenyl)-4-oxo-3,4-dihydroquinazolin-2-yl)piperidine-1-carboxylate**

(*S*)-4l (0.31 g, 88.4%) was prepared from **(*S*)-3d** (0.36 g, 0.60 mmol), acetonitrile (24 mL), triethylamine (2.49 mL, 17.91 mmol), and *N,O*-bis(trimethylsilyl)acetamide (2.19 mL, 8.97 mmol) in the same manner described for **protocol C**. Light brown solid. ^{1}H NMR (400 MHz, $CDCl_3$) δ 8.26 (s, 1H), 8.01 (s, 1H), 7.16 (s, 1H), 6.88 (d, *J* = 10.4 Hz, 1H), 4.92 (dd, *J* = 6.3, 2.7 Hz, 1H), 4.09 (d, *J* = 1.3 Hz, 3H), 3.83 (s, 1H), 3.64 (td, *J* = 12.7, 3.5 Hz, 1H), 1.82 (d, *J* = 12.4 Hz, 1H), 1.72 (d, *J* = 13.2 Hz, 1H), 1.64 – 1.49 (m, 3H), 1.40 (s, 9H). ^{13}C NMR (101 MHz, $CDCl_3$) δ 161.1, 154.3, 154.3, 145.8, 137.9, 137.8, 137.6, 133.5, 133.4, 132.9, 129.9, 127.9, 120.9, 114.0, 113.8, 80.4, 62.0, 52.2, 42.7, 28.5, 27.1, 24.8, 18.8.

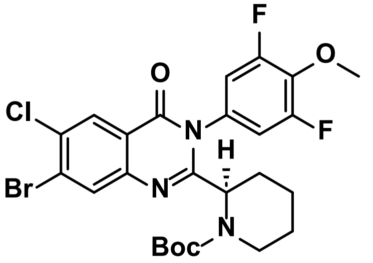

(*R*)-4l

***tert*-butyl (*R*)-2-(7-bromo-6-chloro-3-(3,5-difluoro-4-methoxyphenyl)-4-oxo-3,4-dihydroquinazolin-2-yl)piperidine-1-carboxylate**

(*R*)-4l (0.24 g, 78.2%) was prepared from **(*R*)-3d** (0.32 g, 0.52 mmol), acetonitrile (21 mL), triethylamine (2.19 mL, 15.69 mmol), and *N,O*-bis(trimethylsilyl)acetamide (1.92 mL, 7.83 mmol) in the same manner described for **protocol C**. Light brown solid. ^{1}H NMR (400 MHz, $CDCl_3$) δ

8.27 (s, 1H), 8.01 (s, 1H), 7.16 (s, 1H), 6.87 (d, J = 10.3 Hz, 1H), 4.96 – 4.89 (m, 1H), 4.09 (t, J = 1.2 Hz, 3H), 3.83 (s, 1H), 3.64 (td, J = 12.7, 3.5 Hz, 1H), 1.82 (d, J = 12.0 Hz, 1H), 1.72 (d, J = 13.2 Hz, 1H), 1.59 (s, 4H), 1.40 (s, 9H). ^{13}C NMR (101 MHz, $CDCl_3$) δ 161.1, 157.1, 156.8, 154.3, 145.8, 137.8, 133.4, 132.9, 129.8, 129.8, 127.9, 120.9, 114.1, 113.7, 80.4, 62.0, 52.2, 28.5, 27.1, 24.8, 18.8.

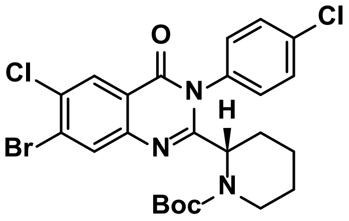

(*S*)-4m

***tert*-butyl (*S*)-2-(7-bromo-6-chloro-3-(4-chlorophenyl)-4-oxo-3,4-dihydroquinazolin-2-yl)piperidine-1-carboxylate**

(*S*)-4m (0.19 g, 55.6%) was prepared from **(*S*)-3e** (0.35 g, 0.61 mmol), acetonitrile (25 mL), triethylamine (2.56 mL, 18.38 mmol), and *N,O*-bis(trimethylsilyl)acetamide (2.25 mL, 9.18 mmol) in the same manner described for **protocol C**. Light brown solid. ^{1}H NMR (400 MHz, $CDCl_3$) δ 8.27 (s, 1H), 8.01 (s, 1H), 7.58 – 7.41 (m, 3H), 7.22 (dd, J = 8.4, 2.4 Hz, 1H), 4.91 (dd, J = 6.6, 2.4 Hz, 1H), 3.85 (s, 1H), 3.68 (t, J = 13.1 Hz, 1H), 1.83 – 1.67 (m, 2H), 1.65 – 1.54 (m, 2H), 1.50 (ddt, J = 12.5, 5.8, 2.7 Hz, 1H), 1.40 (s, 9H), 1.30 – 1.18 (m, J = 5.6, 4.9 Hz, 1H). ^{13}C NMR (101 MHz, $CDCl_3$) δ 161.2, 145.9, 135.7, 134.5, 133.2, 132.9, 130.5, 130.2, 129.8, 129.6, 128.0, 121.0, 80.2, 42.6, 28.6, 26.9, 24.8, 18.7.

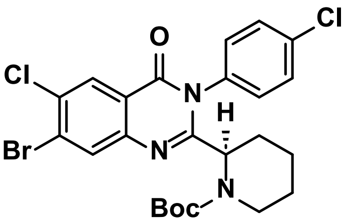

(*R*)-4l

***tert*-butyl (*R*)-2-(7-bromo-6-chloro-3-(4-chlorophenyl)-4-oxo-3,4-dihydroquinazolin-2-yl)piperidine-1-carboxylate**

(*R*)-4l (0.29 g, 88.0%) was prepared from **(*R*)-3e** (0.35 g, 0.60 mmol), acetonitrile (24 mL), triethylamine (2.52 mL, 18.12 mmol), and *N,O*-bis(trimethylsilyl)acetamide (2.22 mL, 9.06 mmol) in the same manner described for **protocol C**. Light brown solid. ^{1}H NMR (400 MHz, $CDCl_3$) δ 8.28 (s, 1H), 8.01 (s, 1H), 7.60 – 7.40 (m, 3H), 7.22 (dd, J = 8.4, 2.4 Hz, 1H), 4.91 (dd, J = 6.5, 2.4 Hz, 1H), 3.85 (s, 1H), 3.68 (t, J = 12.8 Hz, 1H), 1.82 – 1.67 (m, 2H), 1.64 – 1.55 (m, 2H), 1.50 (ddt, J = 12.1, 5.7, 2.6 Hz, 1H), 1.40 (s, 9H), 1.36 – 1.16 (m, 1H). ^{13}C NMR (101 MHz, $CDCl_3$) δ 161.2, 146.0, 135.7, 134.5, 133.2, 132.9, 130.5, 130.2, 129.8, 129.7, 128.0, 121.0, 80.3, 42.7, 28.6, 26.9, 24.8, 18.7.

Protocol D (refer to Scheme S6):

To an oven-dried, inert gas-cooled flask was added the quinazolinone, amine, $Pd_2(dba)_3$, Xantphos, cesium carbonate, and anhydrous toluene. The reaction mixture was sparged with N_2 for 5 min, heated to 100 °C, and allowed to stir for 24 h. After cooling to rt, the mixture was diluted with ethyl acetate (120 mL) and washed three times with sat. brine solution (3x 80 mL). The organic layer was dried over anhydrous sodium sulfate, filtered, and the solvent was evaporated under reduced pressure. The residue was purified by flash chromatography (0% methanol / dichloromethane ~ 10% methanol / dichloromethane) to afford the aminated product.

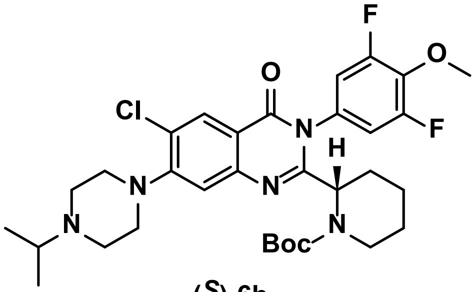

(*S*)-6h

***tert*-butyl (*S*)-2-(6-chloro-3-(3,5-difluoro-4-methoxyphenyl)-7-(4-isopropylpiperazin-1-yl)-4-oxo-3,4-dihydroquinazolin-2-yl)piperidine-1-carboxylate**

(*S*)-6h (0.063 g, 20.2%) was prepared from **(*S*)-4l** (0.29 g, 0.50 mmol), 1-isopropylpiperazine (74.9 μL, 0.52 mmol), $Pd_2(dba)_3$ (0.023 g, 0.025 mmol), Xantphos (0.029 g, 0.050 mmol), cesium carbonate (0.23 g, 0.69 mmol), and anhydrous toluene (5.0 mL) in the same manner described for **protocol D**. Yellow solid. ^{1}H NMR (400 MHz, $CDCl_3$) δ 8.15 (s, 1H), 7.17 (s, 1H), 7.06 (s, 1H), 6.87 (dd, *J* = 10.7, 2.4 Hz, 1H), 4.92 (dd, *J* = 6.4, 2.6 Hz, 1H), 4.07 (t, *J* = 1.2 Hz, 3H), 3.69 – 3.58 (m, 1H), 3.25 (t, *J* = 4.7 Hz, 4H), 2.83 – 2.65 (m, 5H), 2.16 – 1.98 (m, 1H), 1.84 (d, *J* = 12.8 Hz, 1H), 1.79 – 1.64 (m, 2H), 1.62 – 1.46 (m, 2H), 1.39 (s, 9H), 1.32 – 1.20 (m, 1H), 1.11 (d, *J* = 6.5 Hz, 6H). ^{13}C NMR (101 MHz, $CDCl_3$) δ 161.3, 157.1, 157.0, 156.8, 156.7, 156.0, 155.3, 154.6, 154.5, 154.3, 154.2, 146.8, 137.6, 137.5, 137.4, 130.4, 130.3, 130.2, 128.7, 127.8, 117.6, 115.7, 114.0, 80.3, 61.9, 54.7, 52.4, 52.4, 51.4, 48.7, 42.4, 28.5, 27.1, 24.8, 18.9, 18.7, 18.7.

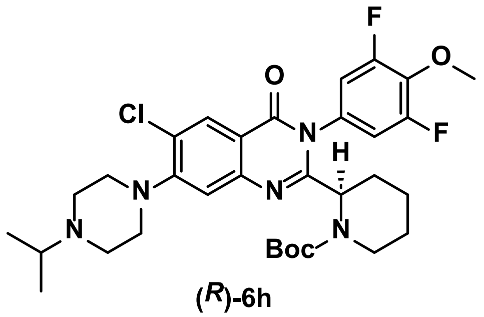

(*R*)-6h

***tert*-butyl (*R*)-2-(6-chloro-3-(3,5-difluoro-4-methoxyphenyl)-7-(4-isopropylpiperazin-1-yl)-4-oxo-3,4-dihydroquinazolin-2-yl)piperidine-1-carboxylate**

(*R*)-6h (0.042 g, 17.5%) was prepared from **(*R*)-4l** (0.22 g, 0.38 mmol), 1-isopropylpiperazine (57.6 μL, 0.40 mmol), $Pd_2(dba)_3$ (0.018 g, 0.019 mmol), Xantphos (0.022 g, 0.038 mmol), cesium carbonate (0.17 g, 0.53 mmol), and anhydrous toluene (4.0 mL) in the same manner described for **protocol D**. Yellow solid. ^{1}H NMR (400 MHz, $CDCl_3$) δ 8.16 (s, 1H), 7.17 (s, 1H), 7.07 (s, 1H), 6.90 – 6.83 (m, 1H), 4.93 (dd, *J* = 6.4, 2.6 Hz, 1H), 4.23 – 4.14 (m, 1H), 4.07 (d, *J* = 1.1 Hz, 3H), 3.84 (d, *J* = 10.8 Hz, 1H), 3.69 – 3.59 (m, 1H), 3.25 (t, *J* = 4.6 Hz, 4H), 2.77 (dt, *J* = 6.6, 4.1 Hz, 5H), 1.84 (d, *J* = 13.0 Hz, 1H), 1.72 (d, *J* = 12.7 Hz, 1H), 1.55 (tdd, *J* = 11.7, 9.0, 4.3 Hz, 2H), 1.39 (s, 9H), 1.31 – 1.21 (m, 1H), 1.12 (d, *J* = 6.4 Hz, 6H). ^{13}C NMR (101 MHz, $CDCl_3$) δ 161.3, 155.3, 146.9, 137.5, 132.3, 132.2, 130.4, 128.8, 128.7, 128.6, 127.9, 117.6, 115.7, 114.0, 113.7, 80.3, 62.0, 54.7, 51.4, 48.7, 31.8, 28.6, 27.1, 19.4, 19.0, 18.7, 18.7, 14.0.

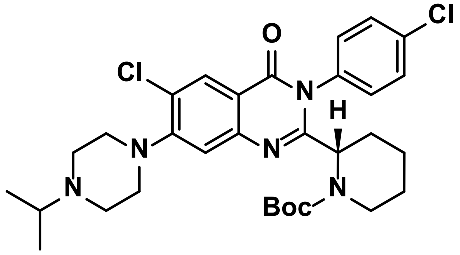

(*S*)-6x

***tert*-butyl (*S*)-2-(6-chloro-3-(4-chlorophenyl)-7-(4-isopropylpiperazin-1-yl)-4-oxo-3,4-dihydroquinazolin-2-yl)piperidine-1-carboxylate**

(*S*)-6x (0.046 g, 24.8%) was prepared from **(*S*)-4m** (0.17 g, 0.31 mmol), 1-isopropylpiperazine (46.4 μL, 0.32 mmol), $Pd_2(dba)_3$ (0.014 g, 0.015 mmol), Xantphos (0.018 g, 0.031 mmol), cesium carbonate (0.14 g, 0.43 mmol), and anhydrous toluene (3.0 mL) in the same manner described for **protocol D**. Light orange solid. ^{1}H NMR (400 MHz, $CDCl_3$) δ 8.17 (s, 1H), 7.49 (td, *J* = 9.6, 2.4 Hz, 2H), 7.42 (s, 1H), 7.23 – 7.17 (m, 2H), 4.91 (dd, *J* = 6.5, 2.5 Hz, 1H), 3.85 (s, 1H), 3.68 (td, *J* = 12.7, 3.4 Hz, 1H), 3.35 – 3.22 (m, 5H), 2.89 – 2.76 (m, 5H), 2.27 (t, *J* = 7.5 Hz, 1H), 1.89 – 1.76 (m, 2H), 1.75 – 1.57 (m, 1H), 1.50 (dq, *J* = 10.6, 6.3 Hz, 1H), 1.39 (s, 9H), 1.16 (d, *J* = 6.5 Hz, 6H). ^{13}C NMR (101 MHz, $CDCl_3$) δ 161.4, 156.0, 154.8, 147.0, 135.4, 134.9, 130.2, 129.7, 128.8, 127.7, 117.7, 80.1, 79.4, 54.9, 50.8, 48.3, 28.6, 27.0, 24.8, 18.9, 18.3, 18.3.

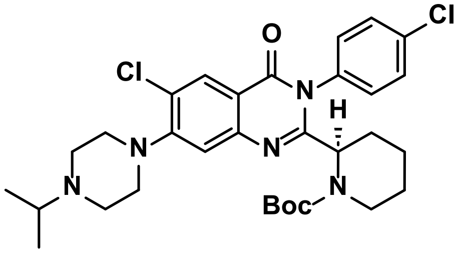

(*R*)-6x

***tert*-butyl (*R*)-2-(6-chloro-3-(4-chlorophenyl)-7-(4-isopropylpiperazin-1-yl)-4-oxo-3,4-dihydroquinazolin-2-yl)piperidine-1-carboxylate**

(*R*)-6x (0.050 g, 16.5%) was prepared from **(*R*)-4m** (0.28 g, 0.50 mmol), 1-isopropylpiperazine (75.9 μL, 0.34 mmol), $Pd_2(dba)_3$ (0.023 g, 0.025 mmol), Xantphos (0.029 g, 0.050 mmol), cesium carbonate (0.23 g, 0.70 mmol), and anhydrous toluene (5.0 mL) in the same manner described for **protocol D**. Pale yellow solid. ^{1}H NMR (400 MHz, $CDCl_3$) δ 8.17 (s, 1H), 7.52 – 7.46 (m, 2H), 7.41 (s, 1H), 7.21 (dd, *J* = 8.3, 2.6 Hz, 1H), 7.17 (s, 1H), 4.91 (dd, *J* = 6.5, 2.5 Hz, 1H), 3.84 (s, 1H), 3.68 (td, *J* = 12.7, 3.5 Hz, 1H), 3.25 (t, *J* = 4.7 Hz, 4H), 2.82 – 2.71 (m, 5H), 1.86 – 1.57 (m, 3H), 1.48 (tq, *J* = 6.1, 3.2 Hz, 2H), 1.39 (s, 9H), 1.29 – 1.18 (m, 1H), 1.12 (d, *J* = 6.5 Hz, 6H). ^{13}C NMR (101 MHz, $CDCl_3$) δ 161.4, 156.0, 155.1, 147.0, 135.3, 134.9, 130.5, 130.2, 129.6, 128.7, 127.7, 117.6, 115.9, 80.2, 54.7, 52.4, 51.4, 48.7, 42.5, 28.6, 27.0, 24.8, 18.9, 18.7, 18.7.

Protocol E (refer to Scheme S6):

Boc-protected quinazolinone was dissolved in ACS grade dichloromethane and cooled to 0 °C using an ice-water bath and allowed to stir for 10 min. To this cooled solution, trifluoroacetic acid was added dropwise over 1 min, the ice-water bath was removed, and the reaction was allowed to reach rt over 2 h. Saturated potassium carbonate solution was added to the reaction mixture dropwise until bubbles stopped forming and the resulting mixture was stirred vigorously for 2 h. The solution was diluted with dichloromethane (80 ml) and washed 5 times with sat. potassium carbonate solution (5 x 60 mL). The organic extract was dried over anhydrous sodium sulfate, filtered, and the solvent was evaporated under reduced pressure to afford the free base product.

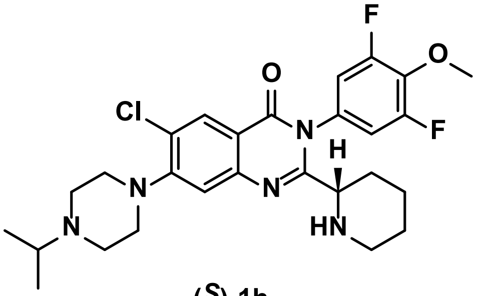

(*S*)-1h

(*S*)-6-chloro-3-(3,5-difluoro-4-methoxyphenyl)-7-(4-isopropylpiperazin-1-yl)-2-(piperidin-2-yl)quinazolin-4(3*H*)-one

(*S*)-1h (0.036 g, 69.1%) was prepared from **(*S*)-6h** (0.061 g, 0.097 mmol), dichloromethane (1.0 mL), trifluoroacetic acid (0.22 mL, 2.90 mmol) and saturated potassium carbonate solution (2 mL) in the same manner described for **protocol E**. Yellow solid. Less than 1% TFA contamination by ^{19}F NMR. ^{1}H NMR (400 MHz, $CDCl_3$) δ 8.15 (s, 1H), 7.23 (s, 1H), 6.97 (dt, *J* = 10.6, 2.2 Hz, 1H), 6.83 (dt, *J* = 10.4, 2.2 Hz, 1H), 4.09 (d, *J* = 1.2 Hz, 3H), 3.32 (dd, *J* = 11.0, 2.7 Hz, 1H), 3.24 (q, *J* = 3.8 Hz, 4H), 3.17 – 3.10 (m, 1H), 2.76 – 2.70 (m, 4H), 2.49 (ddd, *J* = 13.7, 12.3, 3.0 Hz, 1H), 2.10 (s, 2H), 1.88 – 1.80 (m, 1H), 1.74 (dq, *J* = 13.2, 3.7 Hz, 1H), 1.58 – 1.48 (m, 2H), 1.38 (qt, *J* = 12.5, 3.8 Hz, 1H), 1.31 – 1.18 (m, 1H), 1.11 (d, *J* = 6.5 Hz, 6H). ^{13}C NMR (101 MHz, $CDCl_3$) δ 161.0, 158.1, 157.1, 157.0, 156.9, 156.8, 155.3, 154.6, 154.5, 154.4, 154.3, 147.4, 137.8, 137.7, 137.5, 130.6, 130.5, 130.4, 128.8, 127.9, 117.4, 115.6, 113.8, 113.8, 113.8, 113.8, 113.6, 113.6, 113.6, 113.5, 62.0, 58.2, 54.7, 51.4, 48.7, 46.2, 32.2, 26.5, 24.5, 18.7, 18.7. HRMS (ESI-QTOF) m/z: $[M + H]^+$ Calcd for $C_{27}H_{33}ClF_2N_5O_2^+$: 532.2285; Observed: 532.2265. Enantiopurity determined to be 98.6%.

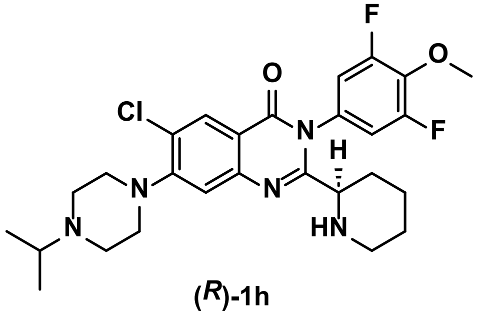

(*R*)-1h

(*R*)-6-chloro-3-(3,5-difluoro-4-methoxyphenyl)-7-(4-isopropylpiperazin-1-yl)-2-(piperidin-2-yl)quinazolin-4(3*H*)-one

(*R*)-1h (0.024 g, 81.0%) was prepared from **(*R*)-6h** (0.035 g, 0.056 mmol), dichloromethane (1.0 mL), trifluoroacetic acid (0.13 mL, 1.67 mmol) and saturated potassium carbonate solution (3 mL) in the same manner described for **protocol E**. Yellow solid. Less than 1% TFA contamination by ^{19}F NMR. ^{1}H NMR (400 MHz, $CDCl_3$) δ 8.17 (s, 1H), 7.25 (s, 1H), 6.98 (dt, *J* = 10.6, 2.2 Hz, 1H), 6.84 (dt, *J* = 10.5, 2.2 Hz, 1H), 4.10 (d, *J* = 1.2 Hz, 3H), 3.33 (dd, *J* = 11.0, 2.7 Hz, 1H), 3.25 (q, *J* = 3.9 Hz, 4H), 3.18 – 3.11 (m, 1H), 2.83 – 2.71 (m, 5H), 2.50 (ddd, *J* = 13.6, 12.2, 3.0 Hz, 1H), 1.85 (d, *J* = 13.4 Hz, 1H), 1.74 (dd, *J* = 13.0, 3.3 Hz, 1H), 1.63 – 1.50 (m, 2H), 1.39 (qt, *J* = 12.5, 3.8 Hz, 1H), 1.32 – 1.19 (m, 2H), 1.12 (d, *J* = 6.5 Hz, 6H). ^{13}C NMR (101 MHz, $CDCl_3$) δ 161.0, 158.1, 157.1, 157.1, 156.9, 156.9, 155.3, 154.6, 154.6, 154.4, 154.4, 147.4, 137.9, 137.7, 137.6, 130.6, 130.5, 130.4, 128.8, 127.9, 117.4, 115.7, 113.9, 113.8, 113.8, 113.8, 113.7, 113.6, 113.6, 113.6, 113.6, 62.1, 58.2, 54.8, 51.4, 48.7, 46.3, 32.3, 26.5, 24.5, 18.7, 18.7. ^{19}F NMR (376 MHz, $CDCl_3$) δ -125.72 (d, *J* = 9.2 Hz), -126.22 (d, *J* = 9.0 Hz). HRMS (ESI-QTOF) m/z: $[M + H]^+$ Calcd for $C_{27}H_{33}ClF_2N_5O_2^+$: 532.2285; Observed: 532.2273. Enantiopurity determined to be 96.8%.

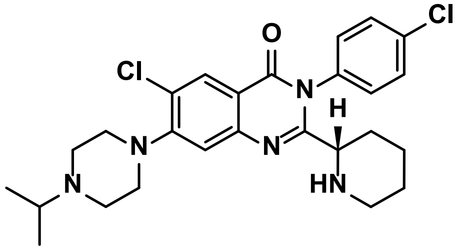

(*S*)-1x

(*S*)-6-chloro-3-(4-chlorophenyl)-7-(4-isopropylpiperazin-1-yl)-2-(piperidin-2-yl)quinazolin-4(3*H*)-one

(*S*)-1x (0.018 g, 49.1%) was prepared from **(*S*)-6x** (0.044 g, 0.073 mmol), dichloromethane (1.0 mL), trifluoroacetic acid (0.17 mL, 2.20 mmol) and saturated potassium carbonate solution (2 mL) in the same manner described for **protocol E**. Light orange solid. Less than 1% TFA contamination by ^{19}F NMR. ^{1}H NMR (400 MHz, $CDCl_3$) δ 8.18 (s, 1H), 7.52 (ddt, *J* = 7.7, 5.4, 2.4 Hz, 2H), 7.31 – 7.26 (m, 2H), 7.18 (dd, *J* = 8.6, 2.6 Hz, 1H), 3.34 – 3.20 (m, 5H), 3.17 – 3.09 (m, 1H), 2.77 (q, *J* = 6.0 Hz, 5H), 2.52 – 2.42 (m, 1H), 1.81 (d, *J* = 13.1 Hz, 1H), 1.77 – 1.68 (m, 1H), 1.58 – 1.47 (m, 2H), 1.38 (qt, *J* = 12.8, 4.0 Hz, 1H), 1.28 – 1.16 (m, 1H), 1.12 (d, *J* = 6.5 Hz, 6H). ^{13}C NMR (101 MHz, $CDCl_3$) δ 161.2, 158.4, 155.2, 147.5, 135.6, 135.2, 130.3, 130.2, 130.1, 130.0, 128.8, 127.8, 117.4, 115.9, 58.2, 54.7, 51.4, 48.8, 46.2, 32.2, 26.5, 24.5, 18.7. HRMS (ESI-QTOF) m/z: $[M + H]^+$ Calcd for $C_{26}H_{32}ClN_5O^+$: 500.1978; Observed: 500.1968. Enantiopurity determined to be >99%.

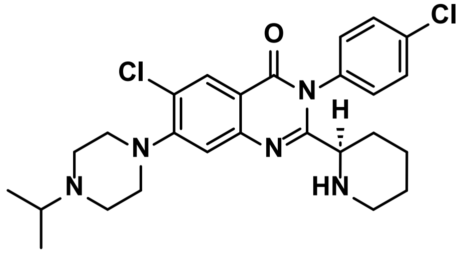

(*R*)-1x

(*R*)-6-chloro-3-(4-chlorophenyl)-7-(4-isopropylpiperazin-1-yl)-2-(piperidin-2-yl)quinazolin-4(3*H*)-one

(*R*)-1x (0.028 g, 69.5%) was prepared from **(*R*)-6x** (0.048 g, 0.080 mmol), dichloromethane (1.0 mL), trifluoroacetic acid (0.18 mL, 2.40 mmol) and saturated potassium carbonate solution (2 mL) in the same manner described for **protocol E**. Pale yellow solid. Less than 1% TFA contamination by ^{19}F NMR. ^{1}H NMR (400 MHz, $CDCl_3$) δ 8.17 (s, 1H), 7.52 (ddd, *J* = 8.2, 5.5, 2.4 Hz, 2H), 7.30 – 7.26 (m, 2H), 7.18 (dd, *J* = 8.7, 2.6 Hz, 1H), 3.34 – 3.20 (m, 5H), 3.17 – 3.10 (m, 1H), 2.76 (q, *J* = 6.0 Hz, 5H), 2.46 (td, *J* = 12.9, 3.0 Hz, 1H), 1.81 (d, *J* = 13.0 Hz, 1H), 1.73 (dq, *J* = 13.2, 3.3 Hz, 1H), 1.59 – 1.48 (m, 2H), 1.38 (qt, *J* = 12.7, 4.0 Hz, 1H), 1.28 – 1.16 (m, 1H), 1.12 (d, *J* = 6.5 Hz, 6H). ^{13}C NMR (101 MHz, $CDCl_3$) δ 161.1, 158.4, 155.2, 147.5, 135.6, 135.1, 130.3, 130.2, 130.0, 130.0, 128.8, 127.8, 117.4, 115.8, 58.2, 54.7, 51.4, 48.7, 46.2, 32.2, 26.5, 24.5, 18.7, 18.7.

HRMS (ESI-QTOF) m/z: $[M + H]^+$ Calcd for $C_{26}H_{32}ClN_5O^+$: 500.1978; Observed: 500.1968. Enantiopurity determined to be >99%.

Protocol F (refer to Scheme S6)[4]:

Secondary amine was dissolved in a 1:1 mixture of ACS grade dichloromethane and methanol. Acetic acid was added dropwise over 1 min, 37% formaldehyde was added dropwise over 1 min, and the reaction mixture was cooled to 0 °C using an ice-water bath and allowed to stir for 15 min. Sodium cyanoborohydride was slowly added portion wise over 1 min to the reaction mixture, the ice water bath was removed, and the reaction was allowed to reach rt, and stirred for 16 h. The reaction mixture was then concentrated *in vacuo*. The residue was neutralized with sat. sodium bicarbonate solution (5 mL) and then extracted three times with dichloromethane (3 x 50 mL). The organics were combined and washed once with sat. brine solution (80 mL). The organic layer was dried over anhydrous sodium sulfate, filtered, and the solvent was evaporated under reduced pressure. The residue was purified by flash chromatography (0% methanol / dichloromethane ~ 10% methanol / dichloromethane) to afford methylated product.

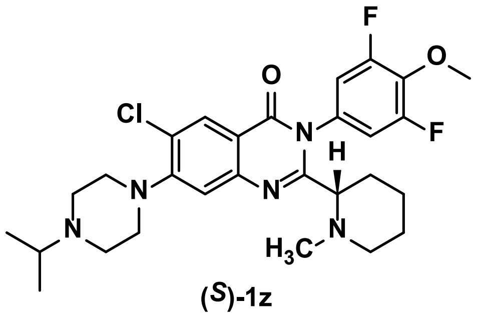

(*S*)-1z

(*S*)-6-chloro-3-(3,5-difluoro-4-methoxyphenyl)-7-(4-isopropylpiperazin-1-yl)-2-(1-methylpiperidin-2-yl)quinazolin-4(3*H*)-one

(*S*)-1z (0.013 g, 86.3%) was prepared from **(*S*)-1h** (0.015 g, 0.028 mmol), dichloromethane (0.5 mL), methanol (0.5 mL), acetic acid (5.3 μL, 0.093 mmol), 37% formaldehyde (4.6 μL, 0.056 mmol), and sodium cyanoborohydride (0.003 g, 0.042 mmol) in the same manner described for **protocol F**. Light yellow solid. ^{1}H NMR (400 MHz, $CDCl_3$) δ 8.18 (s, 1H), 7.48 (s, 1H), 6.80 – 6.74 (m, 2H), 4.12 (d, J = 1.1 Hz, 3H), 3.41 – 3.25 (m, 5H), 3.02 (d, J = 11.2 Hz, 1H), 2.87 (s, 5H), 2.69 (dd, J = 10.2, 2.7 Hz, 1H), 2.19 (s, 3H), 1.93 (td, J = 11.7, 2.8 Hz, 1H), 1.77 (tdd, J = 14.2, 10.9, 3.6 Hz, 5H), 1.59 (d, J = 13.6 Hz, 1H), 1.19 (d, J = 6.5 Hz, 6H). HRMS (ESI-QTOF) m/z: $[M + H]^+$ Calcd for $C_{28}H_{35}ClF_2N_5O_2^+$: 546.2442; Observed: 546.2437. Enantiopurity determined to be 95.5%.

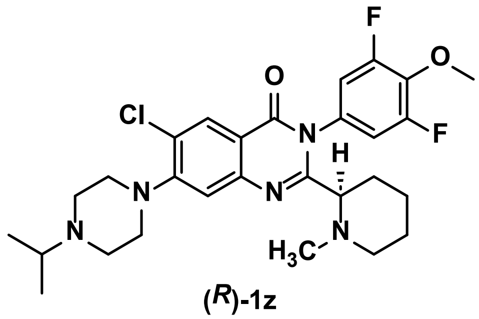

(*R*)-1z

(*R*)-6-chloro-3-(3,5-difluoro-4-methoxyphenyl)-7-(4-isopropylpiperazin-1-yl)-2-(1-methylpiperidin-2-yl)quinazolin-4(3*H*)-one

(*R*)-1z (0.007 g, 65.1%) was prepared from **(*R*)-1h** (0.011 g, 0.020 mmol), dichloromethane (0.5 mL), methanol (0.5 mL), acetic acid (3.7 μL, 0.040 mmol), 37% formaldehyde (3.2 μL, 0.065 mmol), and sodium cyanoborohydride (0.002 g, 0.030 mmol) in the same manner described for **protocol F**. Light yellow solid. ^{1}H NMR (400 MHz, $CDCl_3$) δ 8.17 (s, 1H), 7.48 (s, 1H), 6.81 – 6.75 (m, 2H), 4.11 (t, *J* = 1.2 Hz, 3H), 3.33 – 3.17 (m, 4H), 3.02 (d, *J* = 11.3 Hz, 1H), 2.76 (q, *J* = 5.8 Hz, 5H), 2.69 (dd, *J* = 10.4, 2.7 Hz, 1H), 2.19 (s, 3H), 1.93 (td, *J* = 11.7, 2.8 Hz, 1H), 1.76 (tdd, *J* = 13.6, 10.5, 3.9 Hz, 5H), 1.59 (d, *J* = 13.9 Hz, 1H), 1.12 (d, *J* = 6.5 Hz, 6H). HRMS (ESI-QTOF) m/z: $[M + H]^+$ Calcd for $C_{28}H_{35}ClF_2N_5O_2^+$: 546.2442; Observed: 546.2438. Enantiopurity determined to be 95.4%.

LCMS and Chiral HPLC Chromatograms for (*R*)-1h

LCMS Conditions:

<u>Instrument:</u> Shimadzu SIL-40 XR UHPLC with a Shimadzu LCMS-2020 Quadrupole Mass Spectrometer
<u>Column:</u> Shim-pack Velox SP-C18, 2.7 μm column
<u>Temperature:</u> Ambient
<u>Mobile Phase A:</u> 5% LC-MS grade methanol and 0.1% LC-MS grade formic acid in LC-MS grade water
<u>Mobile Phase B:</u> 0.1% LC-MS grade formic acid in LC-MS grade methanol
<u>Gradient:</u> 20-100% over 3.2 min
<u>Flow rate:</u> 1.0 mL/min
<u>UV Wavelength:</u> 254 nm
<u>Scanning Mode:</u> a scanning multimode source that simultaneously acquires ESI+ and ESI-

LCMS Trace for (*R*)-1h:

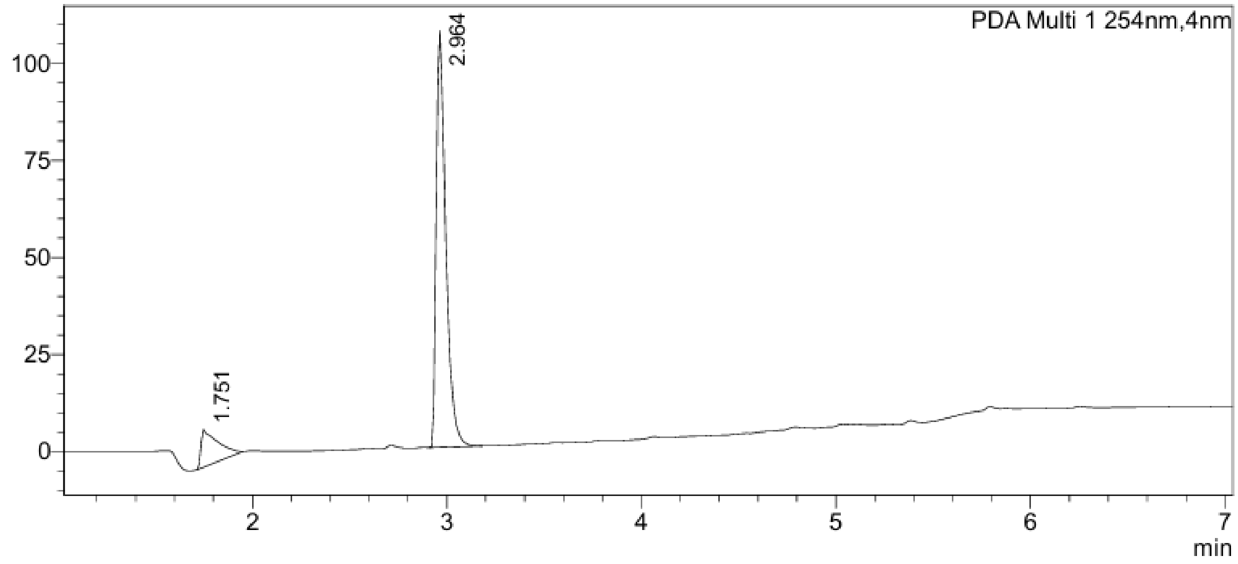

Chiral HPLC Conditions:

Instrument: Agilent 1200-Series HPLC
Column: (*R,R*)-Whelk-O1, 5/100 Kromasil, 25 cm x 4.6mm, 5 micron column
Temperature: Ambient
Mobile Phase A: 0.1% HPLC grade triethylamine in HPLC grade hexanes
Mobile Phase B: 0.1% HPLC grade triethylamine in HPLC grade ethanol
Gradient: 30-80% over 15 min
Flow rate: 1.0 mL/min
UV Wavelength: 254 nm

HPLC Trace for Mixture of (*S*)-1h and (*R*)-1h:

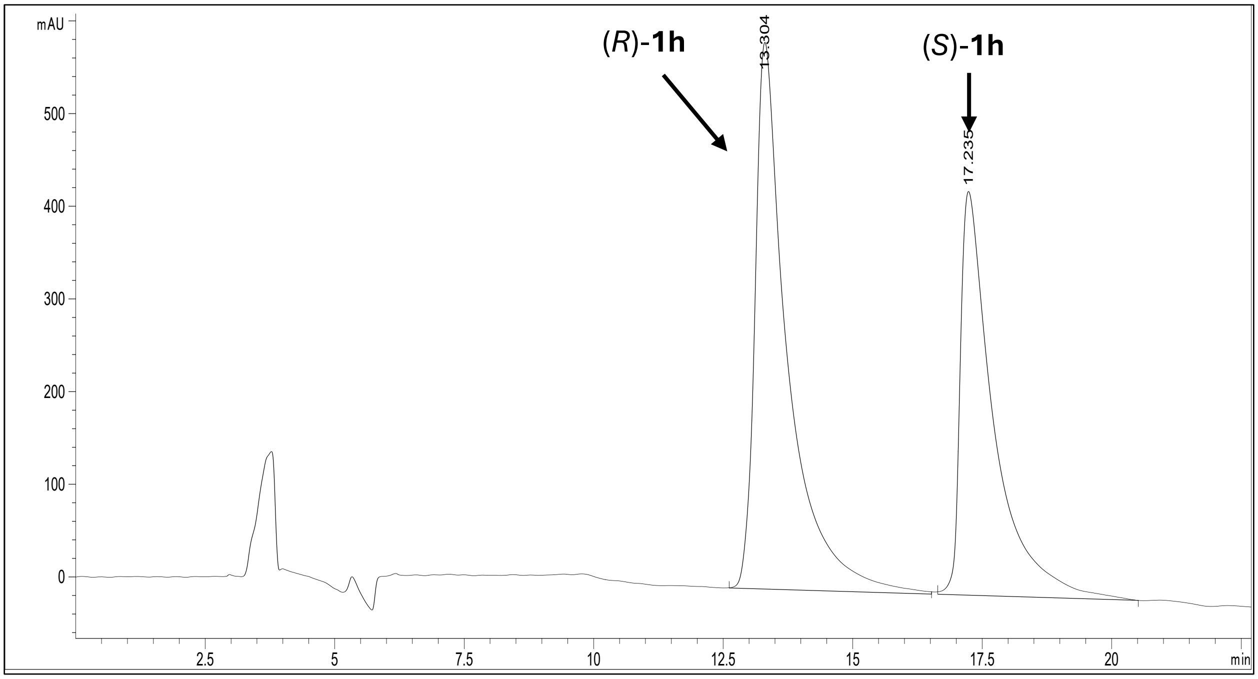

Time	Area	Height	Width	Area %
13.304	25934.9	590.0	0.6188	56.991
17.235	19572.3	435..8	0.6380	43.009

HPLC Trace for (*R*)-1h:

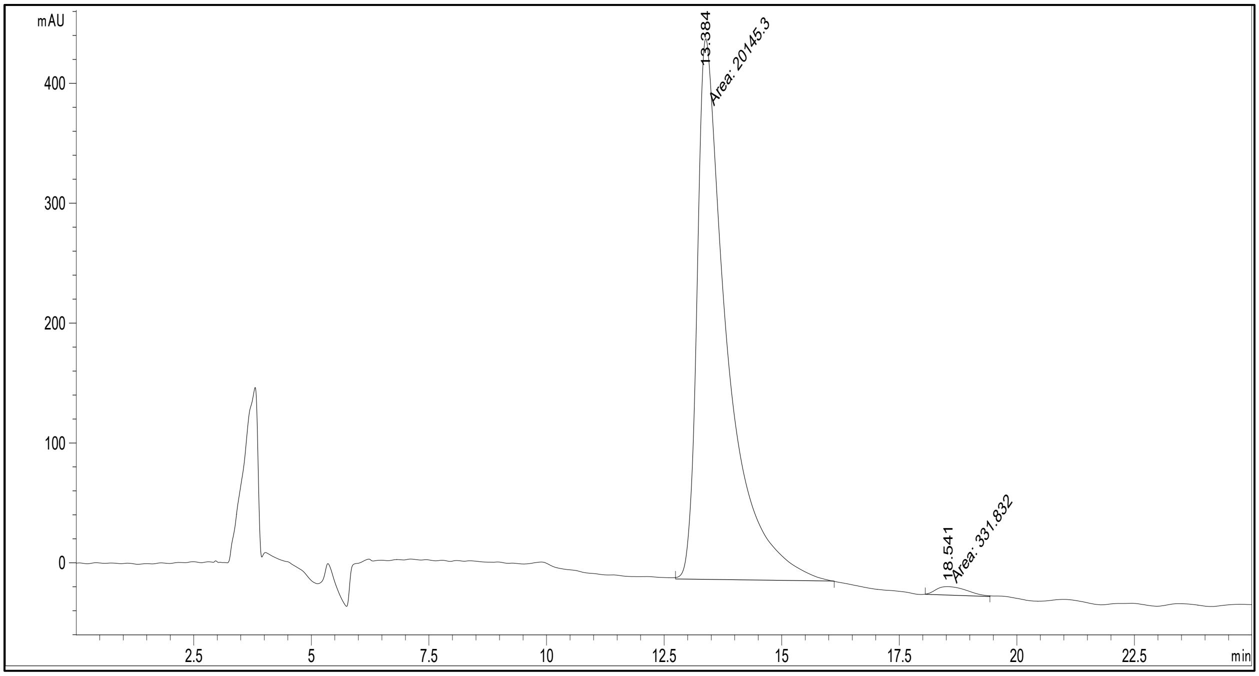

Time	Area	Height	Width	Area %
13.384	20145.3	451.2	0.7442	98.379
18.541	331.8	7.1	0.7739	1.621

Assay Protocols

CHIKV Antiviral Assay (3 point, 10-fold dilution series)

Overall: 3-point drug concentration antiviral titer reduction assays (10, 1, 0.1 μM) against CHIKV were performed for the UW compounds. SR-42718 was used as a positive control, a CHIKV nsP4 polymerase inhibitor. For the assay, confluent 48-well plates of NHDFs (approximately 40,000 cells per well) were treated within 10 minutes following infection with either DMSO or compounds. Compound dilutions were diluted with DMEM supplemented with 5% FBS and 1x PSG. NHDFs were inoculated with CHIKV/25mKate (titer = 8.23e6 PFU/mL) at a multiplicity of infection (MOI) of 1 PFU/cell. At 2 hpi, infection medium was removed and cells were washed with 200 μL of PBS. Fresh medium containing compound was then added to each well. At 24 hpi, 20 μL medium of each well was collected into 96-well plates and frozen at -80°C. Yields of progeny virus were determined by limiting dilution plaque assays. The frozen 96-well plates were thawed, a 10-fold dilution series (6 dilutions) was made in the 96-well plates, and 100 μL of each dilution was transferred to confluent monolayers of Vero cells in 48-well plates. At 2 hpi, the cells were overlaid with 250μL of medium containing 0.3% high viscosity and 0.3% low viscosity carboxy-methyl cellulose. At 72 hpi, the plates were fixed with 3.7% formalin for 10 minutes, then washed and stained with 0.2% methylene blue dye for 5 min. Plaques were counted using a stereomicroscope and dilutions were counted for wells with 50 or less plaques per well. Data was tabulated in Excel.

Cytotoxicity Assay (10 point, 2-fold dilution series)

Overall: Compound cytotoxicity is measured at concentration ranging from 40 μM to 0.156 μM following the Promega CellTiter Glo protocol for cytotoxicity assays. Briefly, 24 hours before treatment, NHDFs were plated in 96-well plates. Dilutions of compounds are added and at 72 hours post treatment, CellTiter Glo substrate solution was added to each well, following the manufacturer's methods. Luminescence was measured using a Promega GloMax plate reader. Control wells were left untreated and blank wells without cells were used to determine the background values.

Cells: Primary normal human dermal fibroblasts (NHDF; ATCC PCS-201-012)

Log D Determination

Compound (2 μL of a 10 mM DMSO stock solution) was transferred into tubes in duplicate, and to each tube was added phosphate buffer (pH 7.4)-1-octanol solution (pH 7.4). After shaking of the tubes for 1 h at rt, contents were centrifuged, and buffer layer samples and 1-octanol layer samples were aliquoted. The samples were diluted with equal volumes of labetalol and tolbutamide in 50% methanol) and analyzed by LC-MS/MS.

Kinetic Solubility Determination

To the lower chamber of a mini-uniprep vial was added separate dilutions of compound prepared from DMSO stock solutions, followed by the appropriate volume of phosphate buffer (pH 7.4). After shaking for 24 h at rt, the sample was centrifuged for 30 min, and mini-unipreps were compressed to collect filtrates which were analyzed by HPLC.

Liver Microsomal Stability

To incubation plates containing mouse microsomes (CD-1 mouse, Corning, cat. no. 452413) in phosphate buffer was added acetonitrile-diluted DMSO stock solution of compound with mixing. Samples were with and without NADPH cofactor. Plates were incubated at 37 °C for 60 min while shaking. At incremental timepoints ranging from 5 to 60 min, samples were quenched using equal concentrations of tolbutamide and labetalol. Samples were centrifuged, diluted with water, and supernatant was sampled for LC-MS/MS analysis. Controls included: testosterone, diclofenac, and propafenone.

Mouse Plasma Stability

DMSO stock solutions of test compound and controls (enalapril maleate, bisacodyl, and procaine HCl) were prepared. To a 96-well plate containing plasma (CD-1 mouse, EDTA-K2 anticoagulant, minimum of 20 male donors) was added compound or control, and the mixtures were incubated at 37 °C for 0 min, 10 min, 30 min, 60 min or 120 min. At the indicated incubation time, tolbutamide and labetalol in acetonitrile were added to precipitate protein. After shaking for 20 min and centrifuging for 20 min, supernatant was added to water, shaken for 10 min, and then samples were analyzed by LCMS using an internal standard.

Plasma Protein Binding

A 96-well equilibrium dialysis device (HTDialysis LLC, cat. No. 1101) was employed using CD-1 male mouse plasma with EDTA-K2 anticoagulant. Warfarin was used as a positive control. An aliquot of the compound in phosphate buffer was transferred to the donor side of each dialysis well in triplicate, and the dialysis buffer was loaded to the receiver side of the well. The dialysis plate was incubated at 37 °C with 5% CO2 on a slow shaking platform for 4 h. Samples from each side of the dialysis device were transferred to new 96-well plates and diluted with a solution of tolbutamide, labetalol, and metformin in acetonitrile which contained an internal standard. After mixing and centrifuging samples for 20 min, supernatants were analyzed by LCMS/MS

High-Content Microscopy Anti-CHIKV Screen

Vero E6 cells were seeded at 1.75X10^4/well Vero E6 Passage 4 into a 96-well glass-bottom plate and incubated overnight (150 µL) of DMEM in 2% fetal bovine serum (FBS). The next day, serial dilutions of compound were added to the appropriate well in triplicate in a total volume of 50 µL of DMEM and incubated for 2 hours. Ten µL of chikungunya virus strain 181/25 was added to the proper wells to bring the MOI to 1 and incubated for 18 h. Media was removed and cells were fixed with 4% paraformaldehyde at RT for 30 min. Cells were washed 3X with PBS and

permeabilized with 0.2% Triton-X at RT for 10 minutes, rewashed, and incubated with 1% BSA in PBS for 1 hour to block nonspecific binding. Chikungunya Virus Capsid Polyclonal primary antibody (Invitrogen™ PA5143450) at 1:2000 was added, and plates were incubated in a humidified chamber for 2 hours at RT. Plates were washed, and Goat anti-Rabbit IgG (H+L) Cross-Adsorbed Secondary Antibody, Alexa Fluor™ 488 (Invitrogen™ Catalog # A-11008) 1:1000 in PBS were added and incubated for 1 hour at RT in the dark. Cells were then stained with DAPI and 20 images per well were captured per well using a CellVoyager CQ1, a high-content confocal microscope analyzer (Yokogawa). Images were assessed using the high-content analysis system software, CellPathfinder to identify and quantify individual cells. Nuclei were first identified and segmented based on the DAPI signal. The DAPI-defined nuclear mask was used as a seed to identify the cell boundary via a signal-based expansion into the anti-CHIKV A488 channel; a low fluorescence threshold was applied to include cytoplasm with even faint viral antigen signal. The mean fluorescence intensity of the anti-CHIKV antibody signal was then quantified for each cell.

The resulting single-cell data was exported as CSV files and analyzed using R (version 4.4.2). A quality control gate was first applied to the data based on nuclear area and DAPI intensity to exclude cellular debris and aggregates. An infection threshold was then statistically defined as the 99.9th percentile of the anti-CHIKV antibody intensity measured in control wells containing uninfected cells (Mock). Any cell with an intensity value above this threshold was classified as infected. The primary endpoint for each well was the percentage of infected cells, calculated as (number of infected cells / total number of quality-controlled cells) × 100. The half-maximal inhibitory concentration (IC_{50}) of the compound was determined by fitting the data to a four-parameter logistic (4PL) curve.

Figure S1. CHIKV Titer Reduction Assay Dose Response Curve for (*R*)-1h

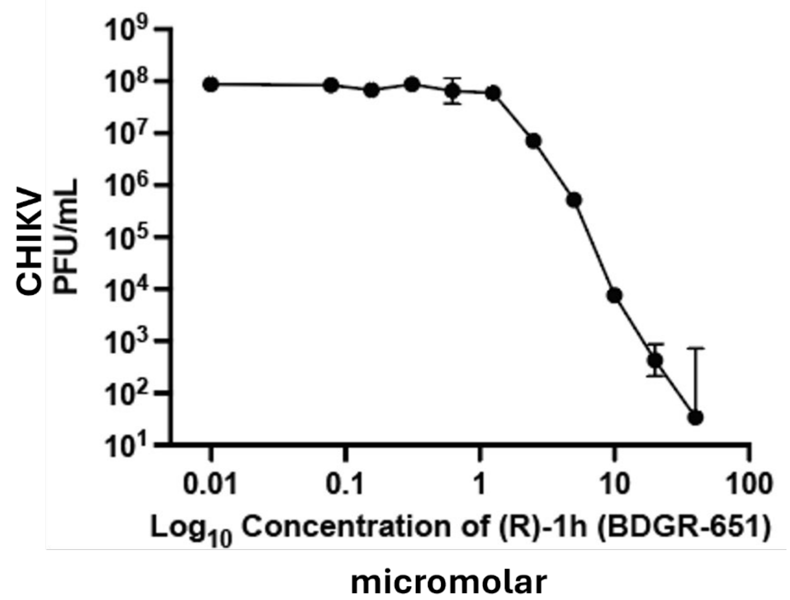

References

(1) Li, X.; Golden, J. E. Construction of *N*-Boc-2-Alkylaminoquinazolin-4(3*H*)-Ones via a Three-Component, One-Pot Protocol Mediated by Copper(II) Chloride that Spares Enantiomeric Purity. *Adv. Synth. Catal.* **2021**, *363* (6), 1638-1645. DOI: 10.1002/adsc.202001279.

(2) Xi, N.; Wang, L.; Wu, Z.; Feng, X.; Wu, Y. Substituted Aminopyrimidine Compounds and Methods of Use. US Patent 0318913 A1, Nov. 3, 2016.

(3) Hadd, M. J.; Holladay, M. W.; Rowbottom, M. Quinazolinone Compounds and Methods of Use Thereof. US Patent 0053174 A1, Mar. 1, 2012.

(4) Gardelli, C.; Nizi, E.; Muraglia, E.; Crescenzi, B.; Ferrara, M.; Orvieto, F.; Pace, P.; Pescatore, G.; Poma, M.; Rico Ferreira, M. D. R.; et al. Discovery and Synthesis of HIV Integrase Inhibitors: Development of Potent and Orally Bioavailable N-Methyl Pyrimidones. *J. Med. Chem.* **2007**, *50* (20), 4953-4975. DOI: 10.1021/jm0704705.

(5) Fan, W.; Nakamura, Y.; Yamago, S. Synthesis of Multivalent Organotellurium Chain-Transfer Agents by Post-modification and Their Applications in Living Radical Polymerization. *Chem. Eur. J.* **2016**, *22* (47), 17006-17010. DOI: 10.1002/chem.201603682.

(6) Phillip Kennedy, J.; Lindsley, C. W. Progress towards the synthesis of piperazimycin A: synthesis of the non-proteogenic amino acids and elaboration into dipeptides. *Tet. Lett.* **2010**, *51* (18), 2493-2496. DOI: 10.1016/j.tetlet.2010.02.168.

(7) Beutner, G. L.; Young, I. S.; Davies, M. L.; Hickey, M. R.; Park, H.; Stevens, J. M.; Ye, Q. TCFH–NMI: Direct Access to *N*-Acyl Imidazoliums for Challenging Amide Bond Formations. *Org. Lett.* **2018**, *20* (14), 4218-4222. DOI: 10.1021/acs.orglett.8b01591.

(8) Coste, J.; Frerot, E.; Jouin, P. Coupling N-Methylated Amino Acids Using PyBroP and PyCloP Halogenophosphonium Salts: Mechanism and Fields of Application. *J Org. Chem.* **1994**, *59* (9), 2437-2446. DOI: 10.1021/jo00088a027.
(9) Zheng, Y.; Van Den Kerkhof, M.; Ibrahim, M.; De Esch, I. J. P.; Maes, L.; Sterk, G. J.; Caljon, G.; Leurs, R. Lead Optimization of the 5-Phenylpyrazolopyrimidinone NPD-2975 toward Compounds with Improved Antitrypanosomal Efficacy. *J. Med. Chem.* **2024**, *67* (4), 2849-2863. DOI: 10.1021/acs.jmedchem.3c01976.
(10) Chen, H.; Xu, X.; Liu, L. L.; Tang, G.; Zhao, Y. Phosphorus oxychloride as an efficient coupling reagent for the synthesis of esters, amides and peptides under mild conditions. *RSC Adv.* **2013**, *3* (37), 16247-16250. DOI: 10.1039/C3RA42887G.

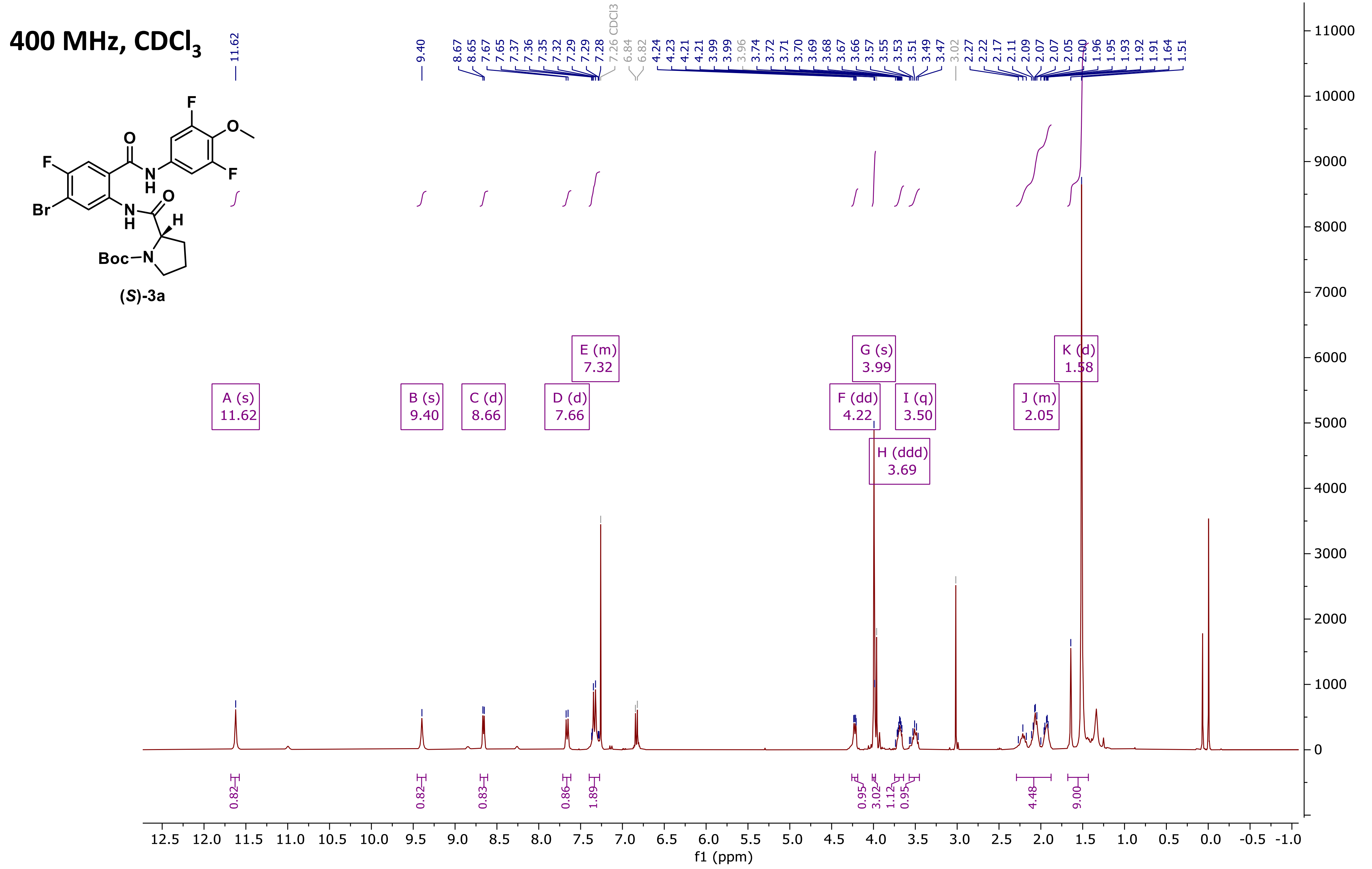
400 MHz, CDCl3
(S)-3a
A (s) 11.62
B (s) 9.40
C (d) 8.66
D (d) 7.66
E (m) 7.32
F (dd) 4.22
G (s) 3.99
H (ddd) 3.69
I (q) 3.50
J (m) 2.05
K (d) 1.58
f1 (ppm)

101 MHz, $CDCl_3$

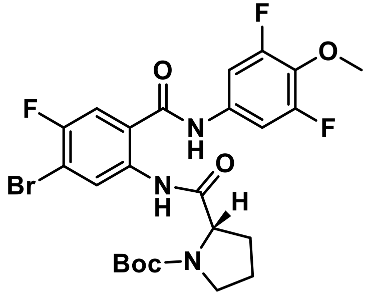

(*S*)-3a

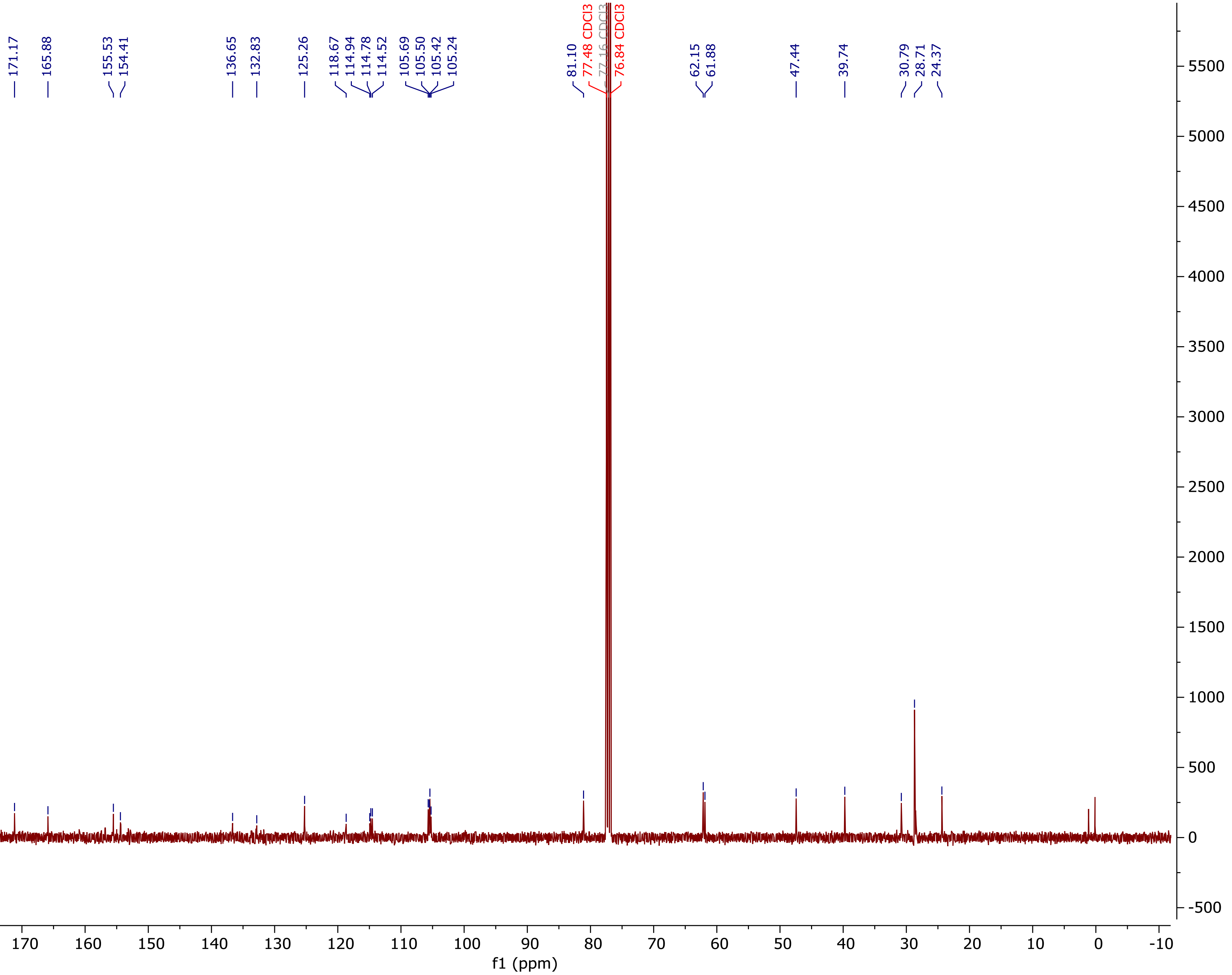

400 MHz, $CDCl_3$

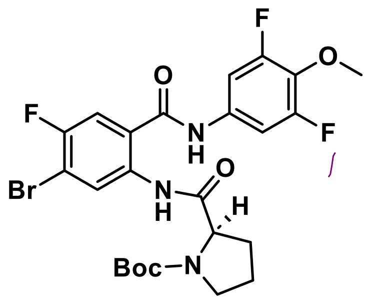

(*R*)-3a

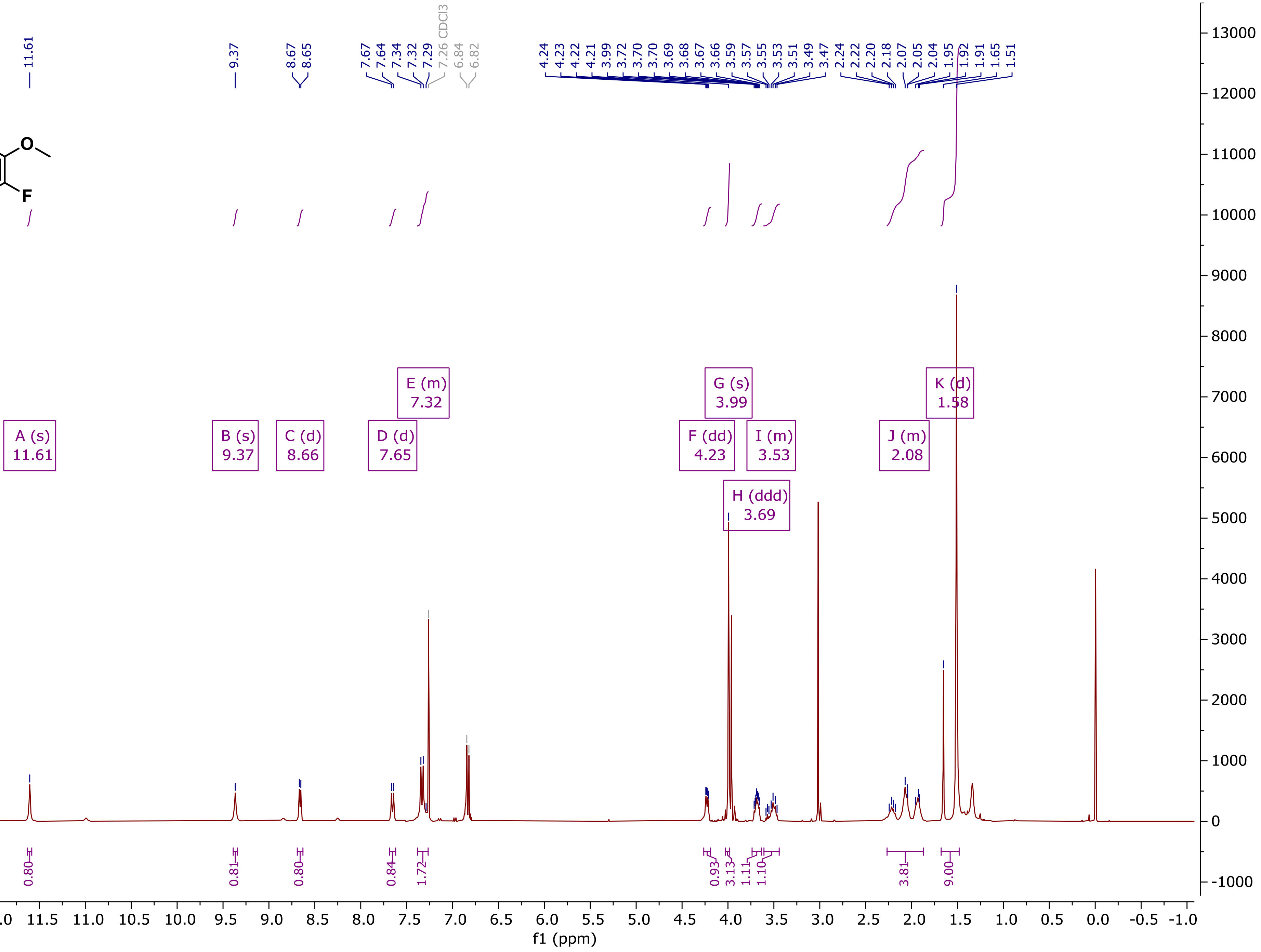

101 MHz, $CDCl_3$

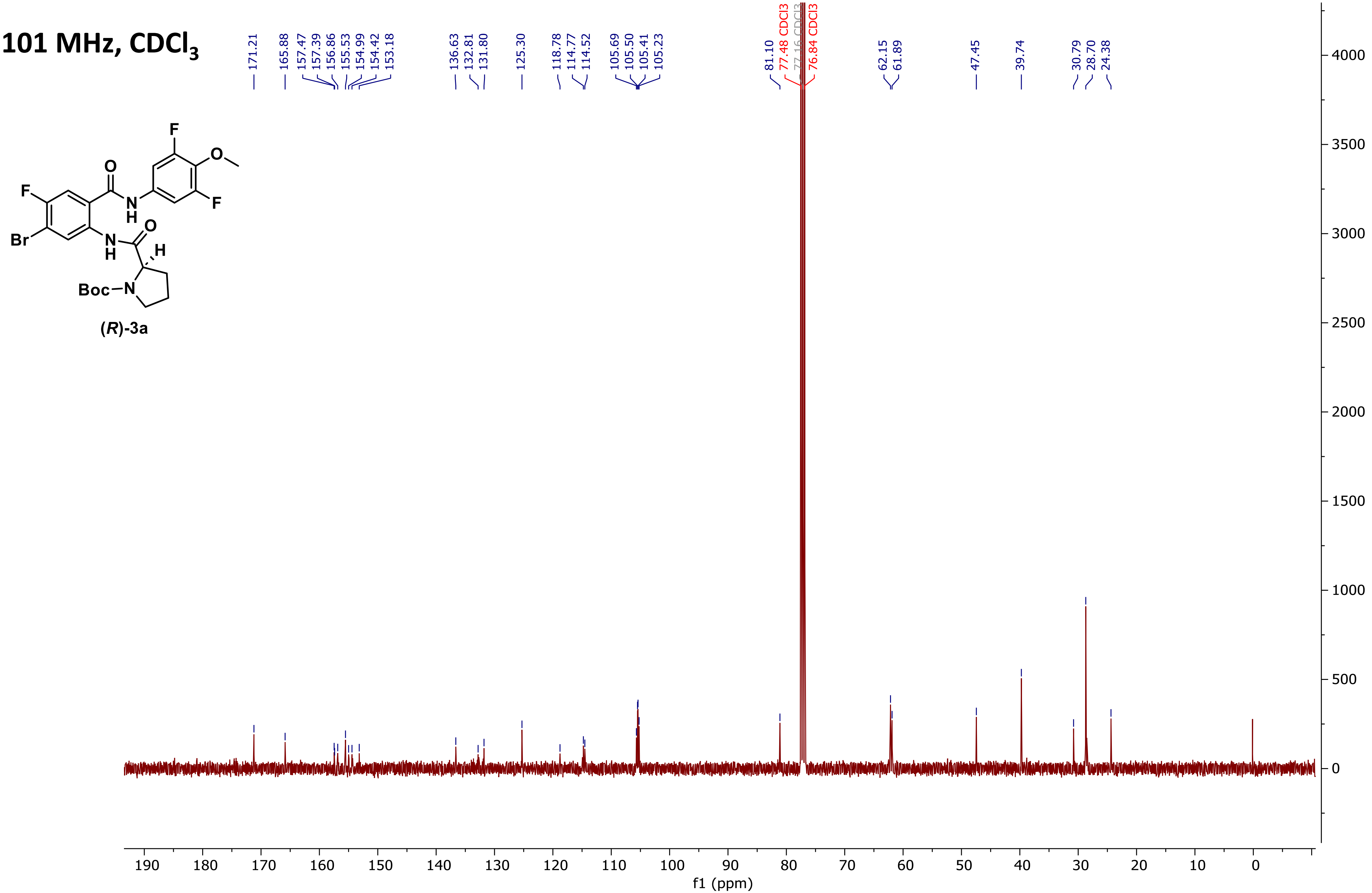

(*R*)-3a

400 MHz, $CDCl_3$

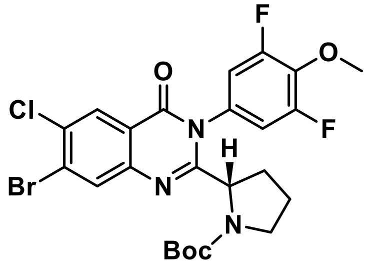

(*S*)-4d

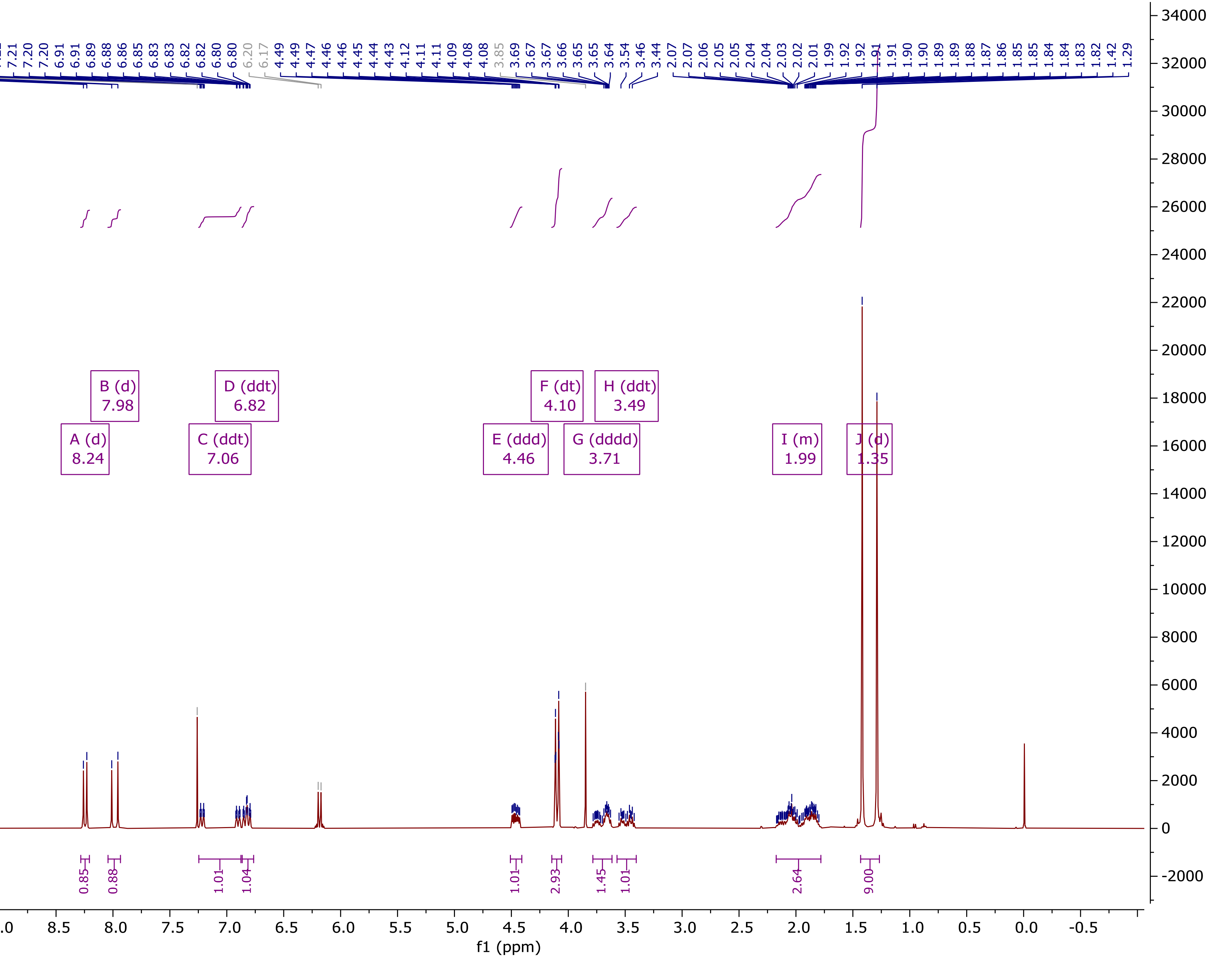

101 MHz, $CDCl_3$

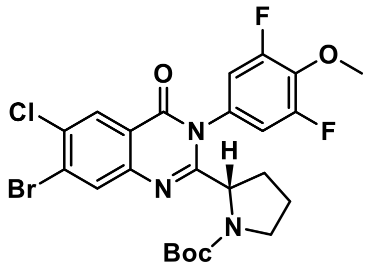

(*S*)-4d

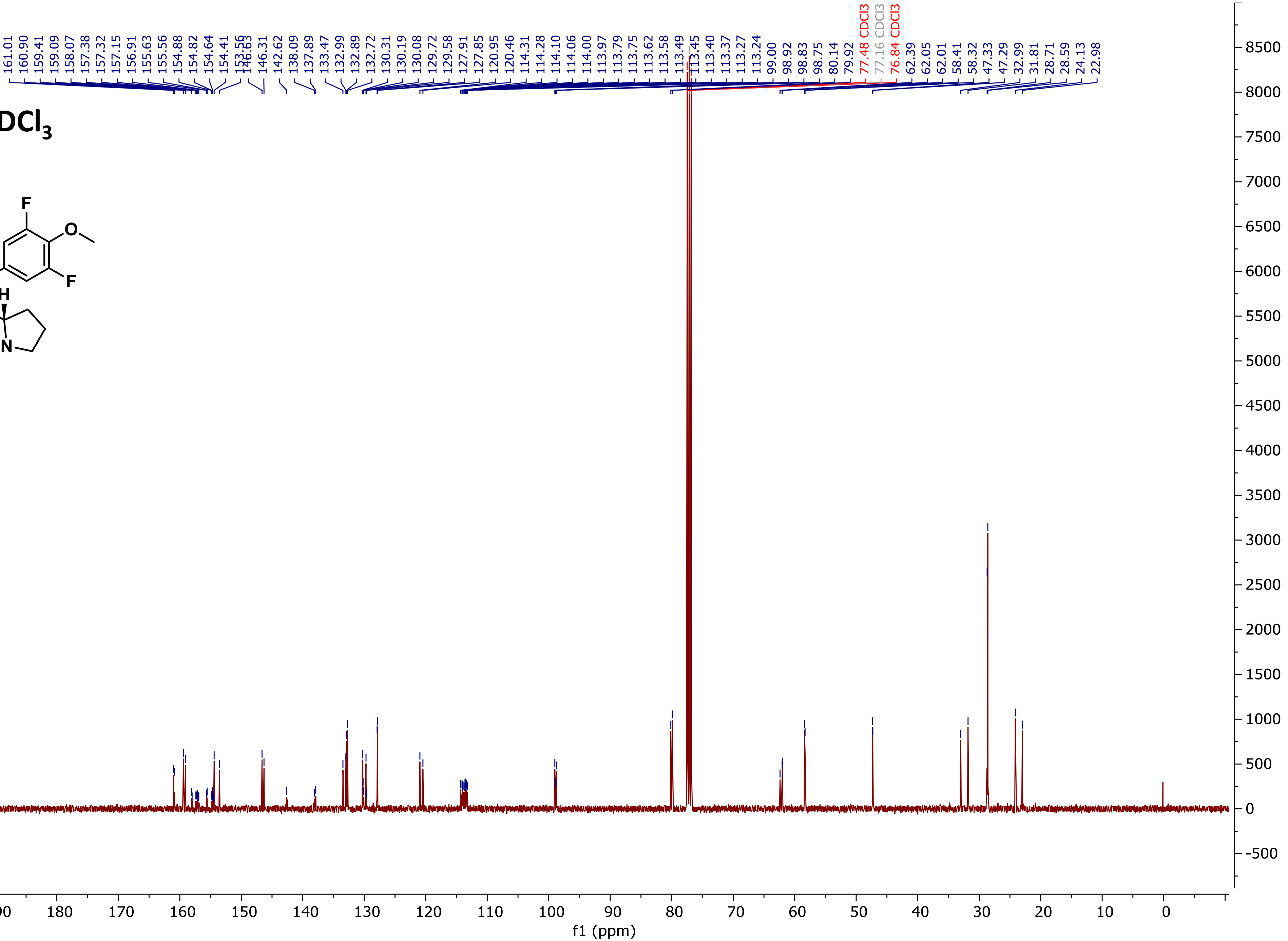

400 MHz, $CDCl_3$

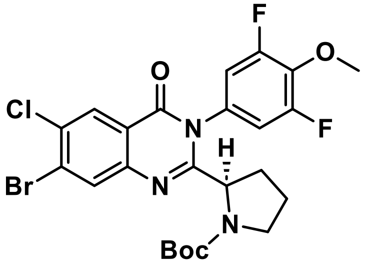

(*R*)-4d

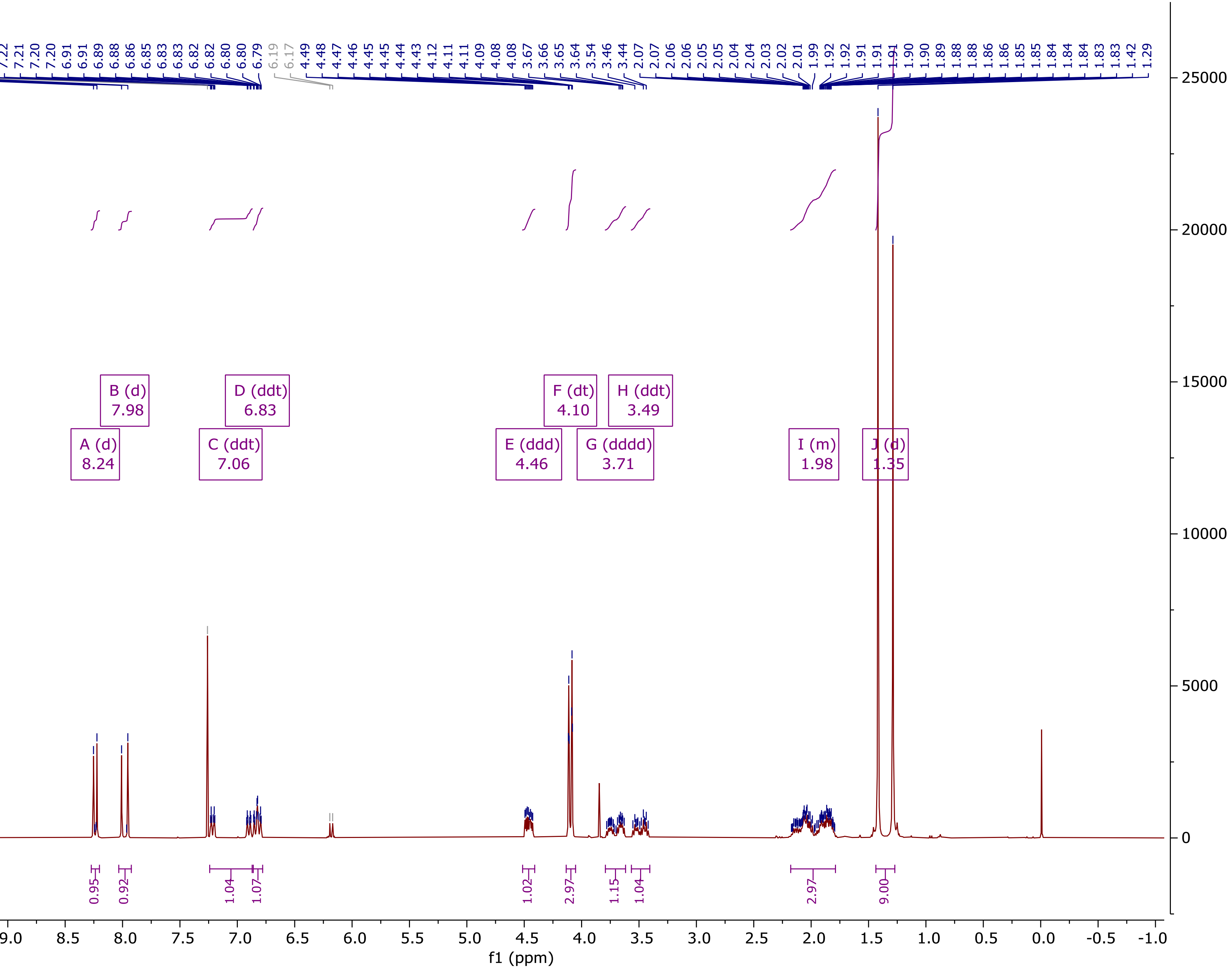

101 MHz, $CDCl_3$

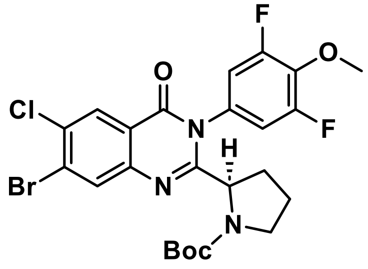

(*R*)-4d

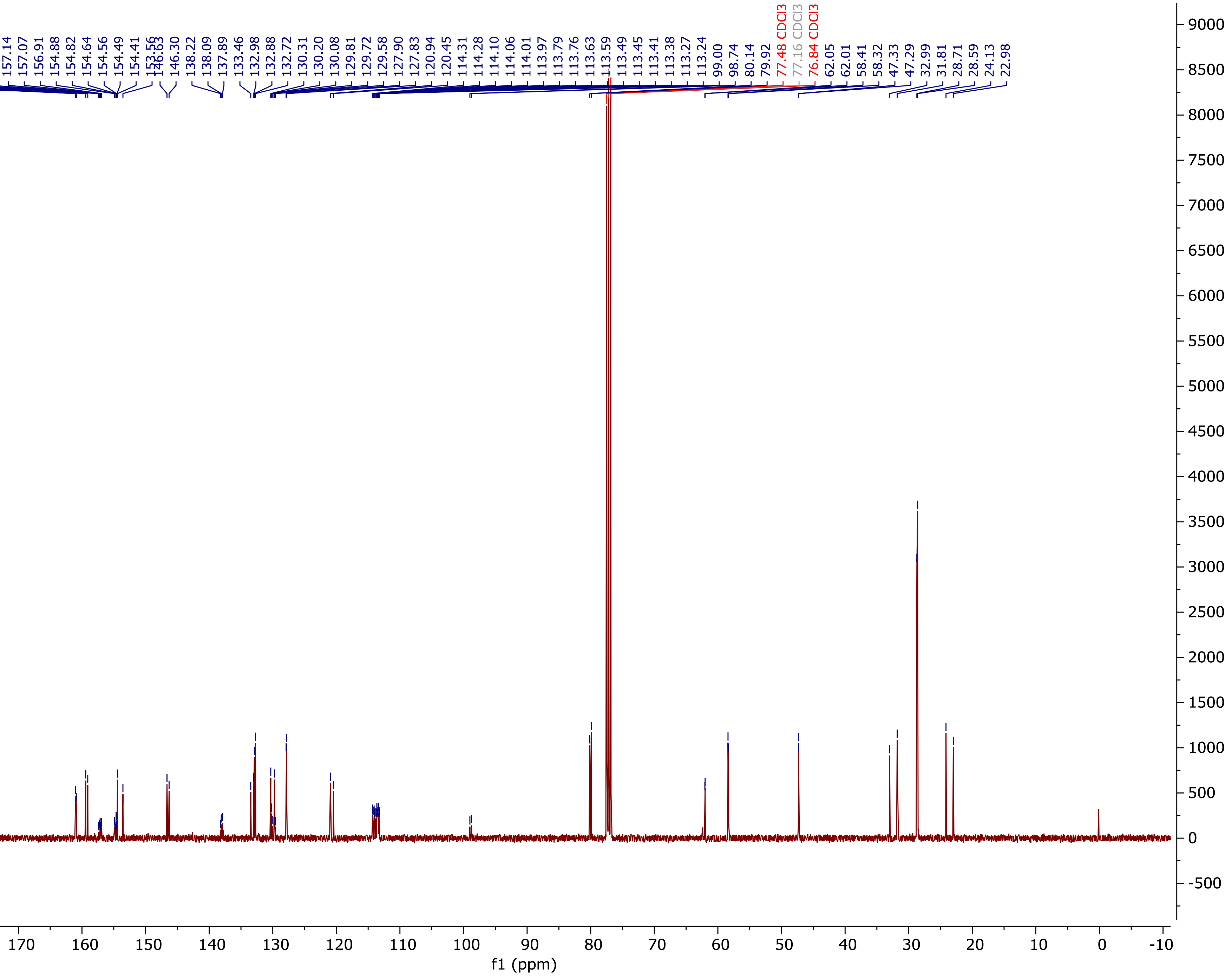

400 MHz, $CDCl_3$

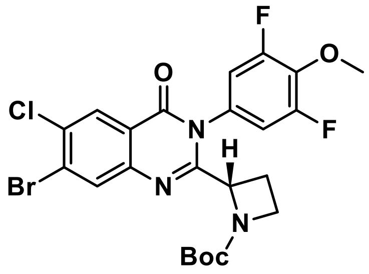

(*S*)-4e

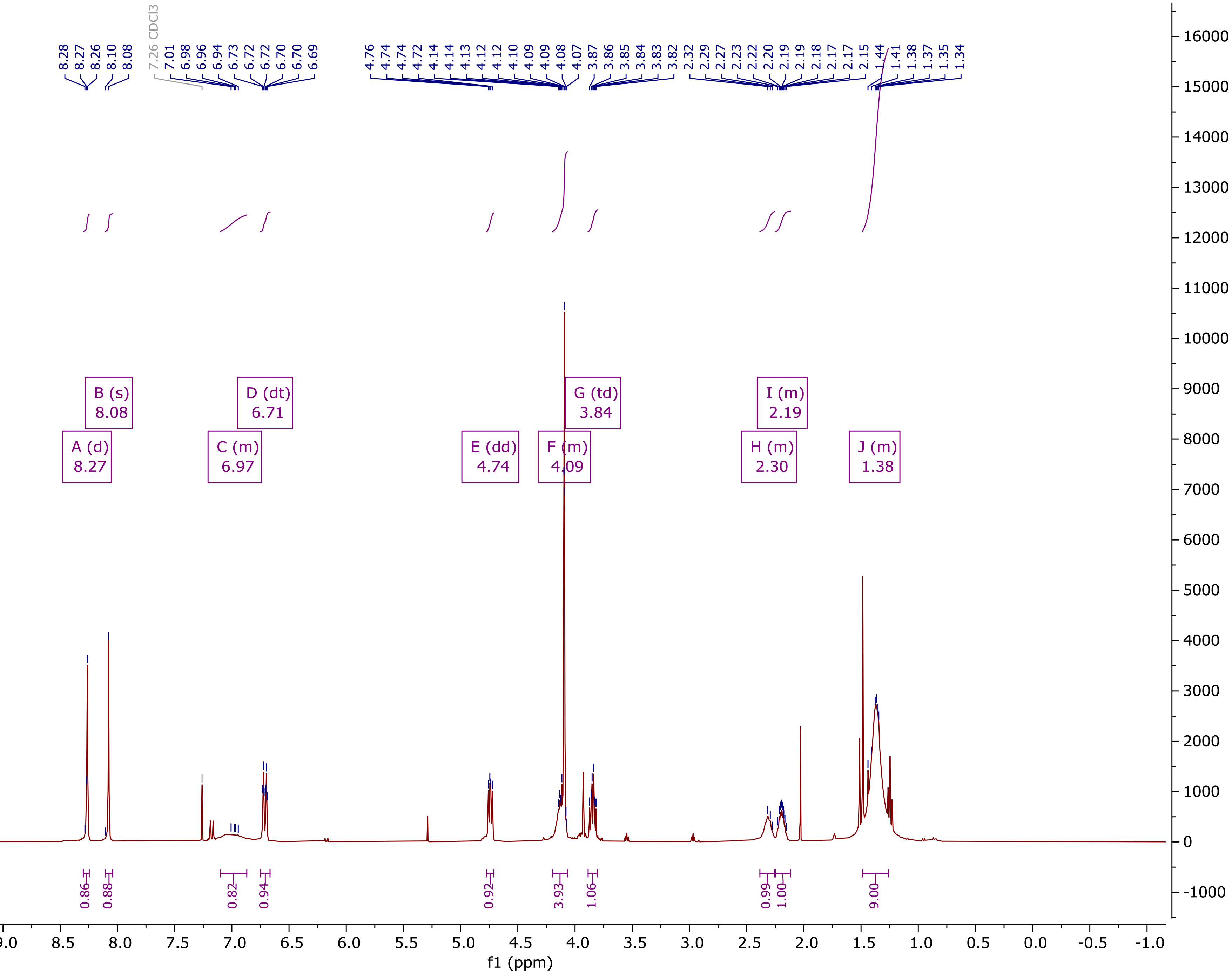

101 MHz, $CDCl_3$

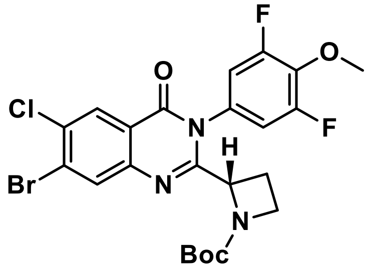

(*S*)-4e

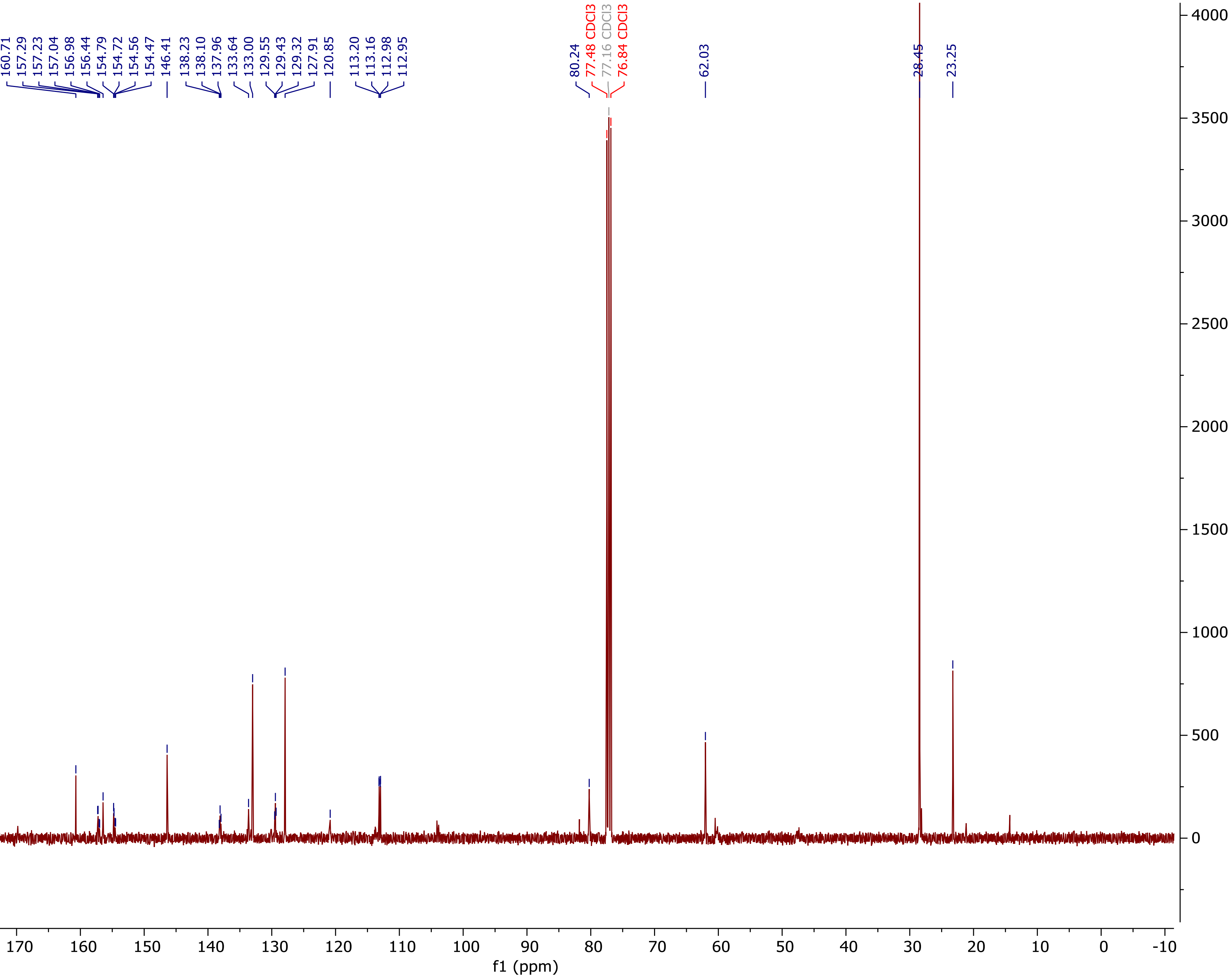

400 MHz, $CDCl_3$

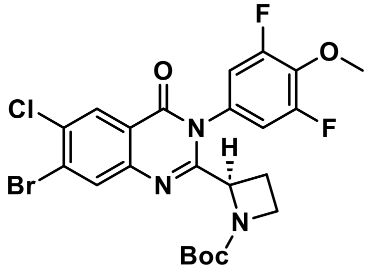

(*R*)-4e

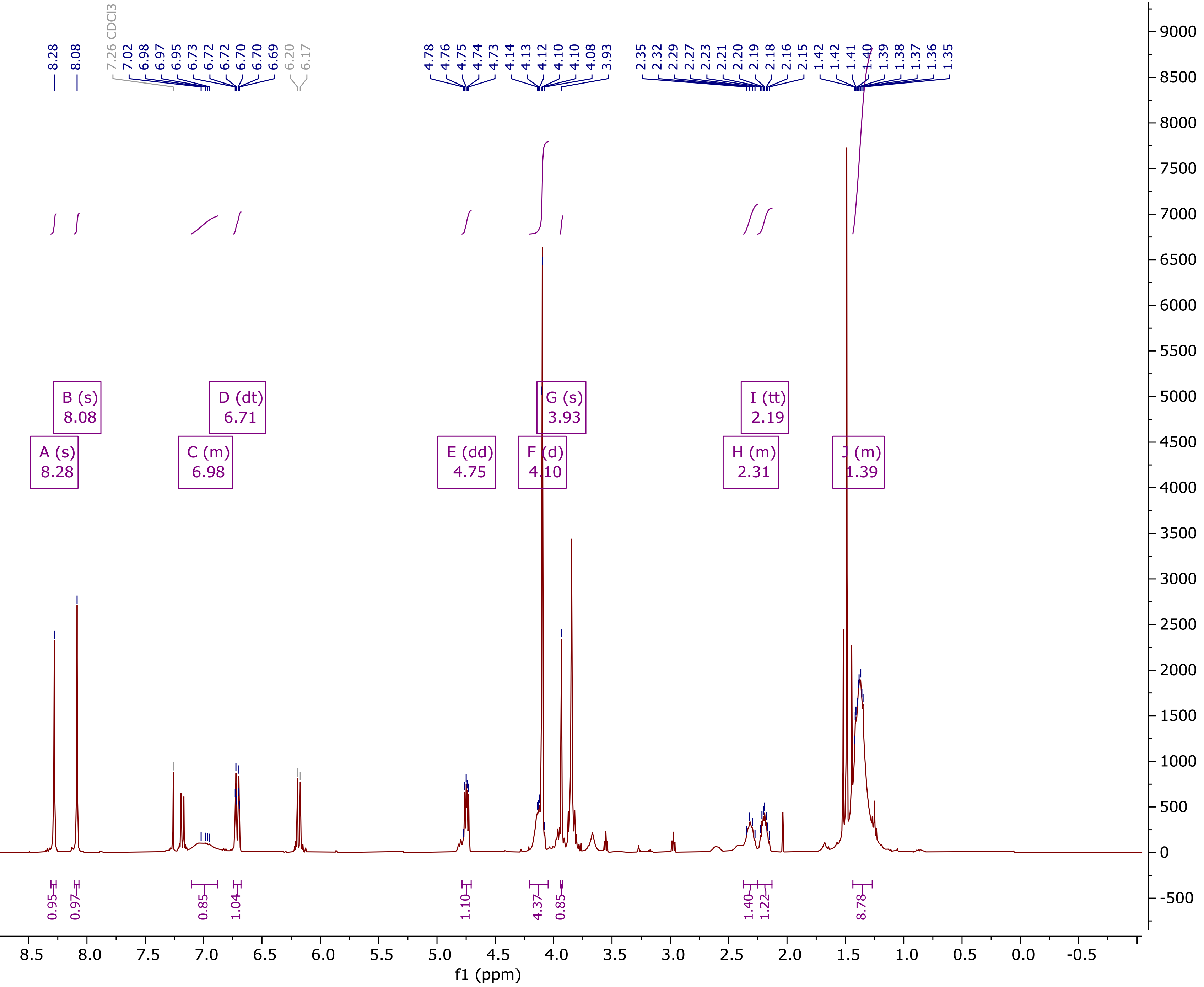

101 MHz, $CDCl_3$

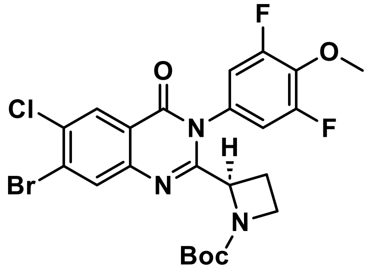

(R)-4e

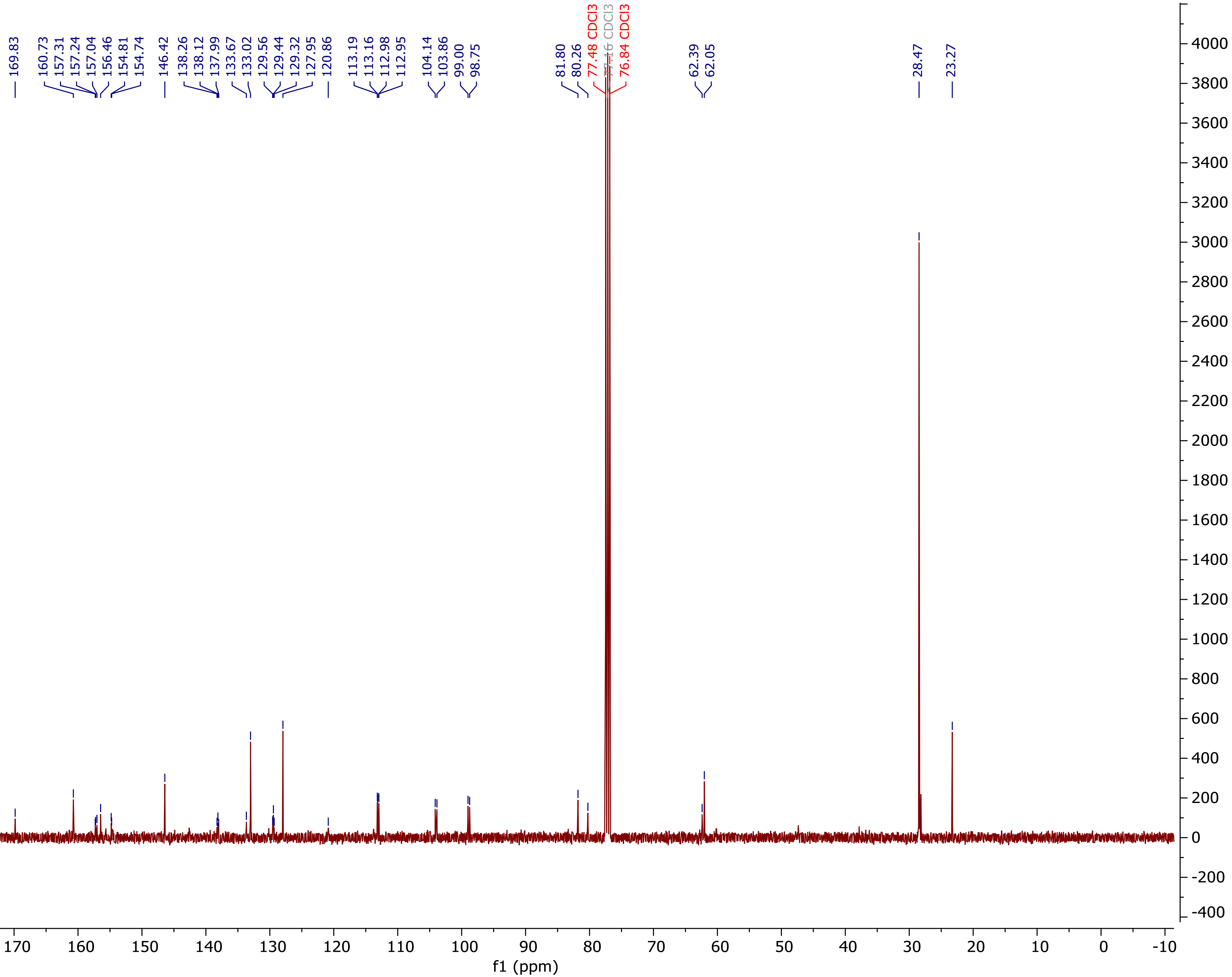

400 MHz, $CDCl_3$

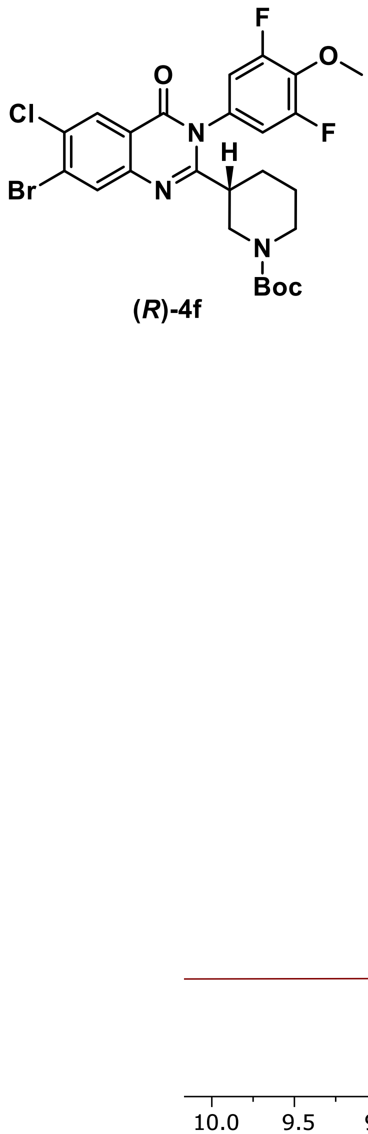

(*R*)-4f

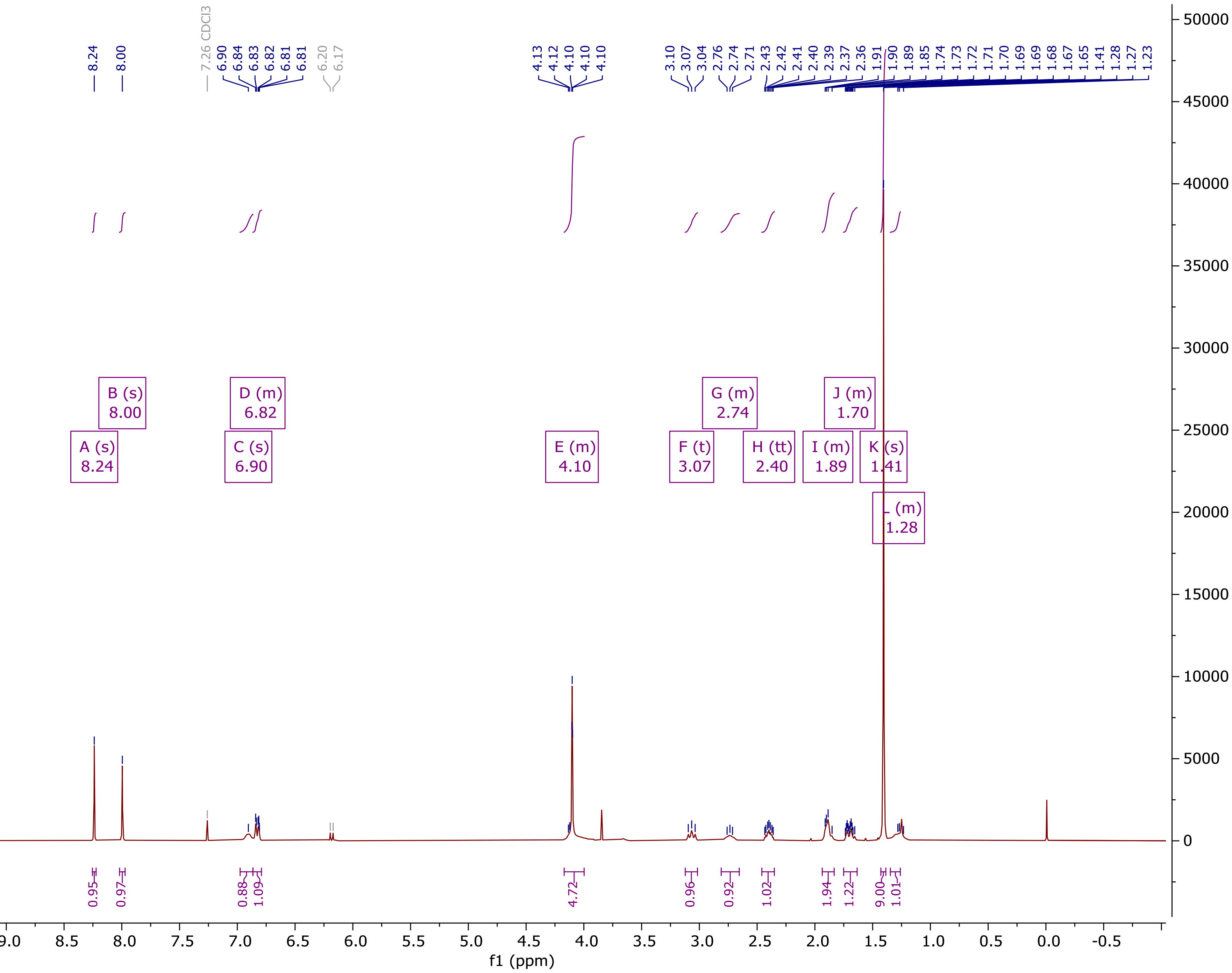

101 MHz, $CDCl_3$

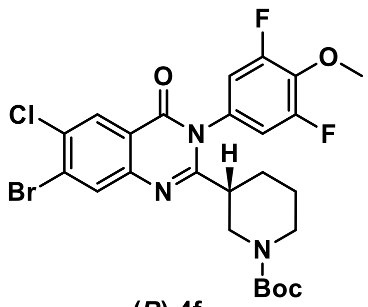

(*R*)-4f

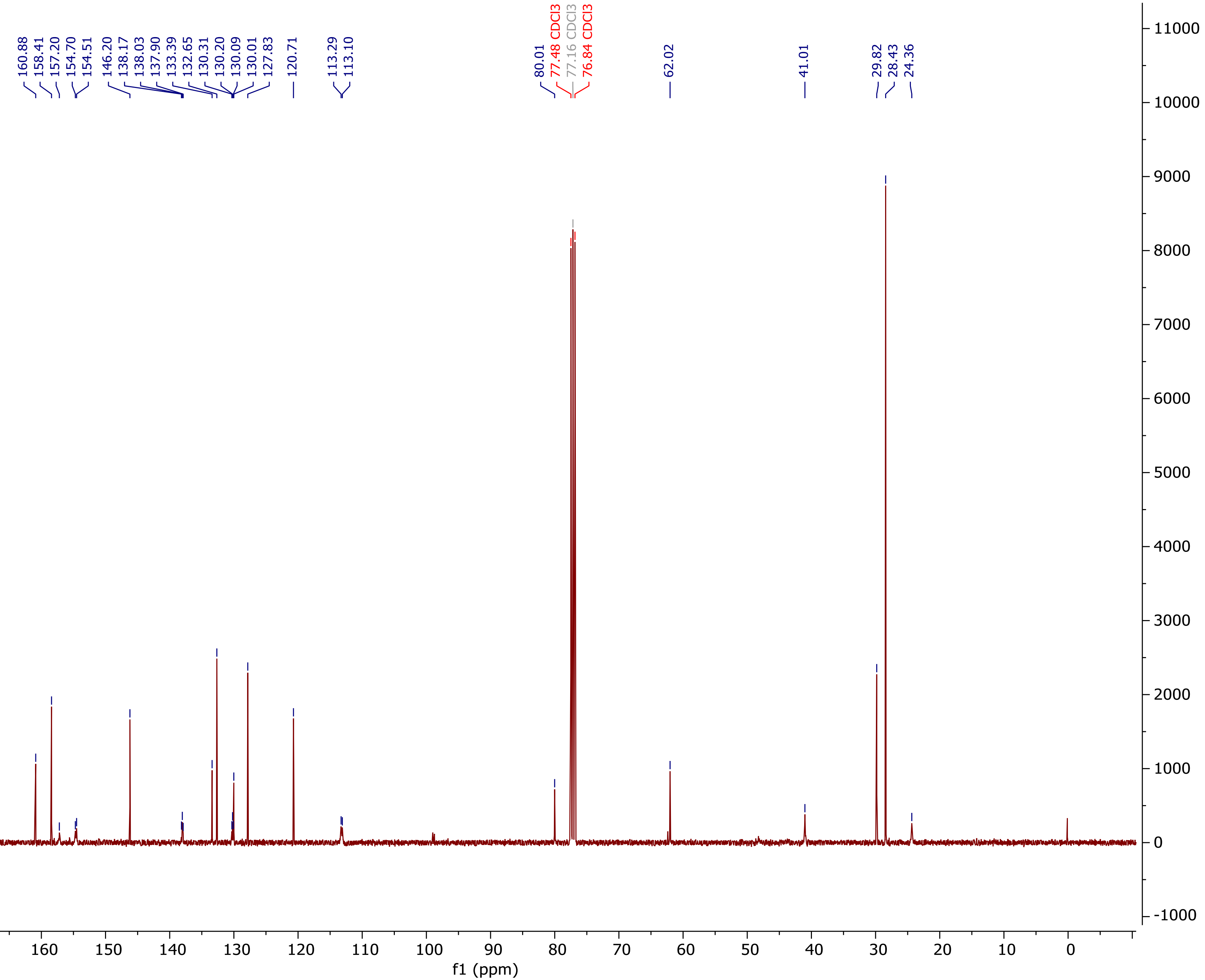

400 MHz, $CDCl_3$

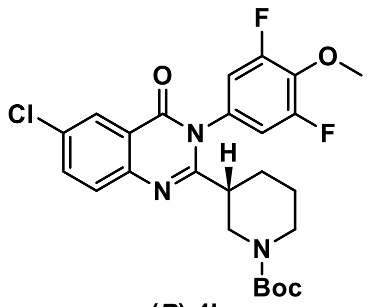

(*R*)-4h

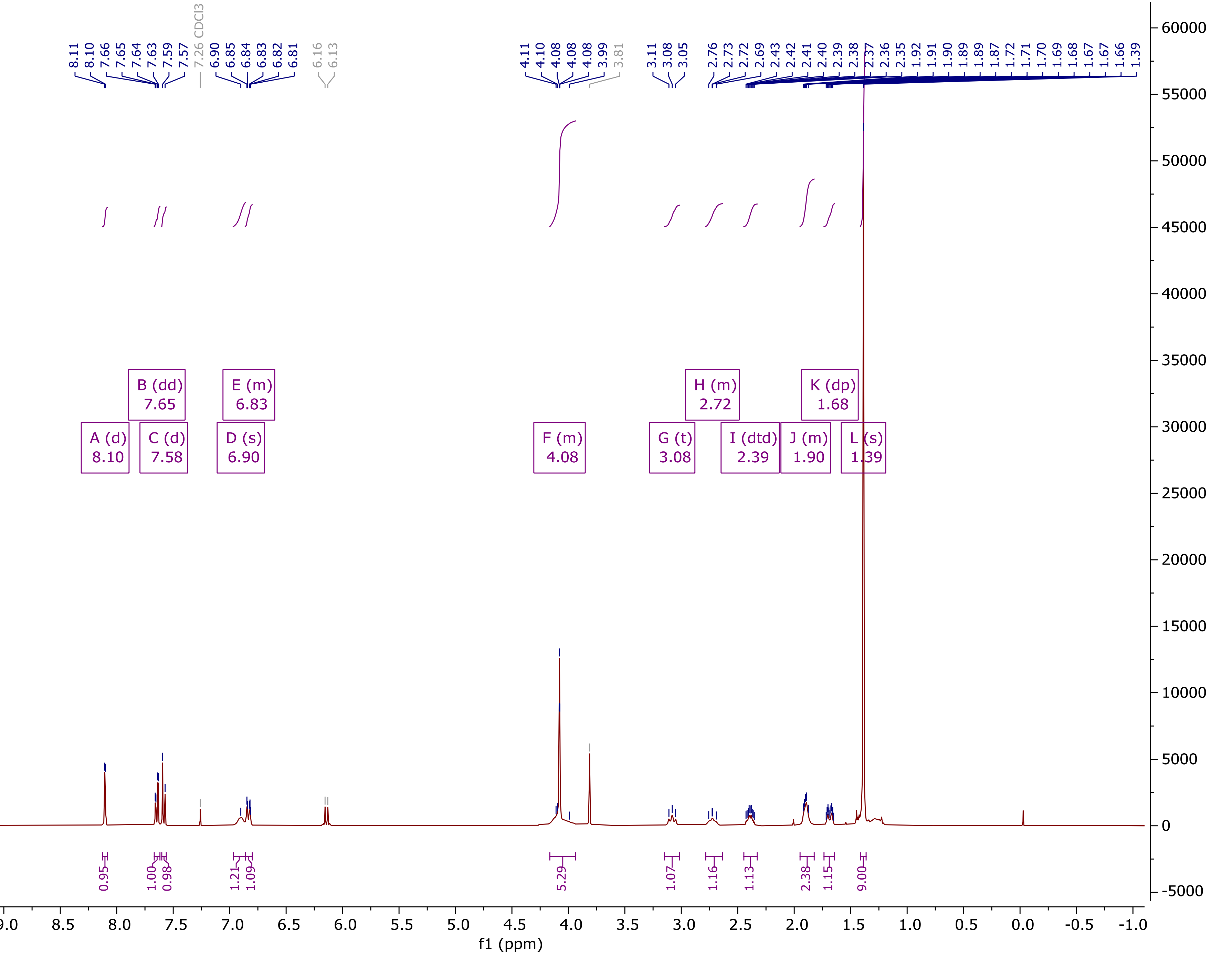

101 MHz, $CDCl_3$

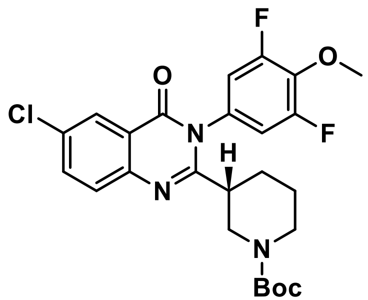

(*R*)-4h

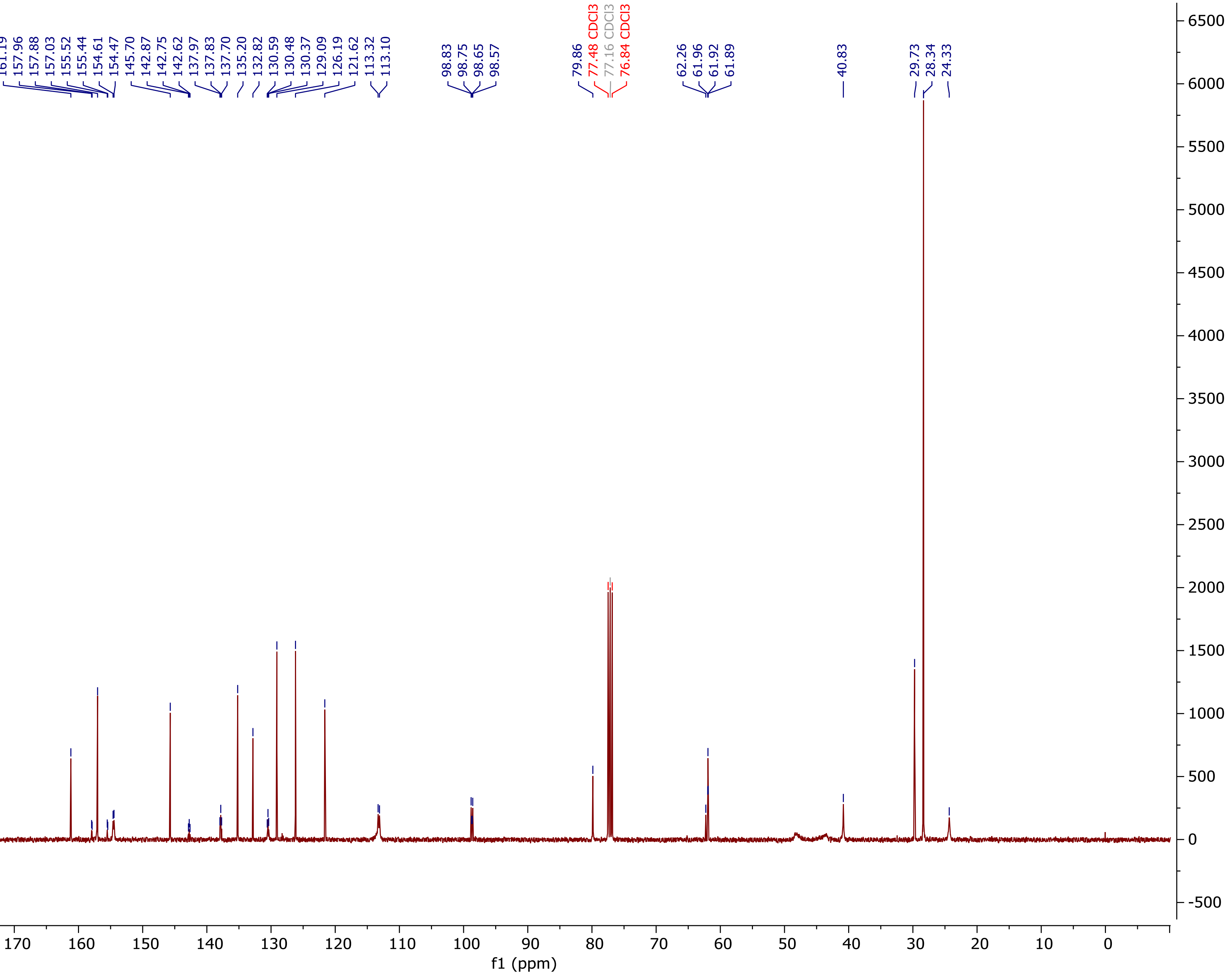

400 MHz, $CDCl_3$

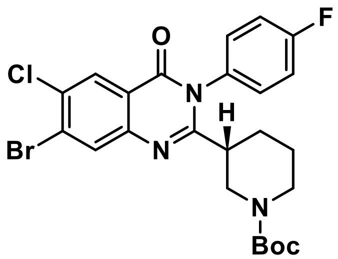

(*R*)-4i

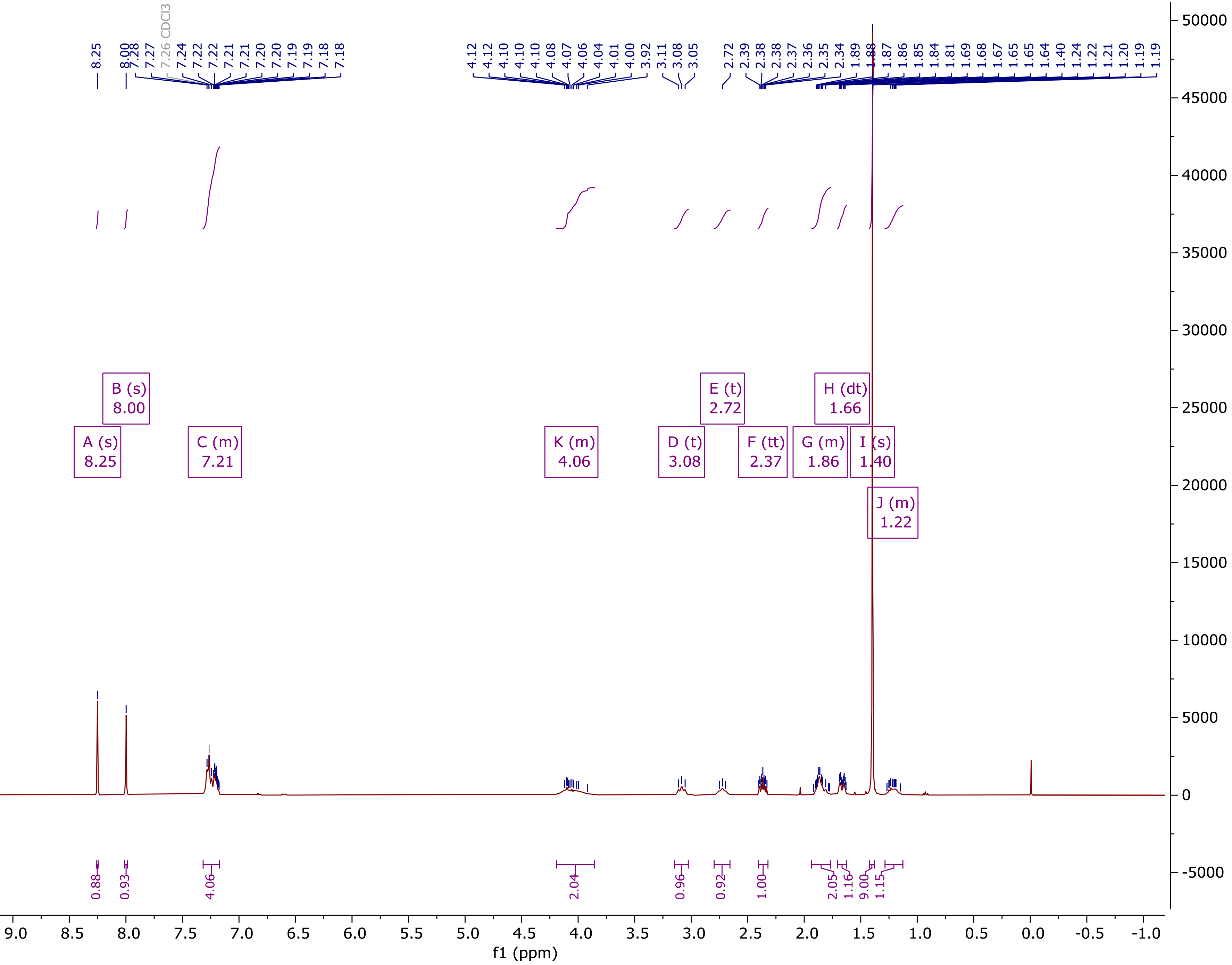

101 MHz, $CDCl_3$

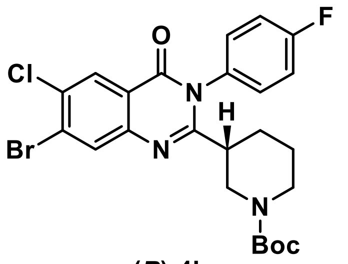

(*R*)-4i

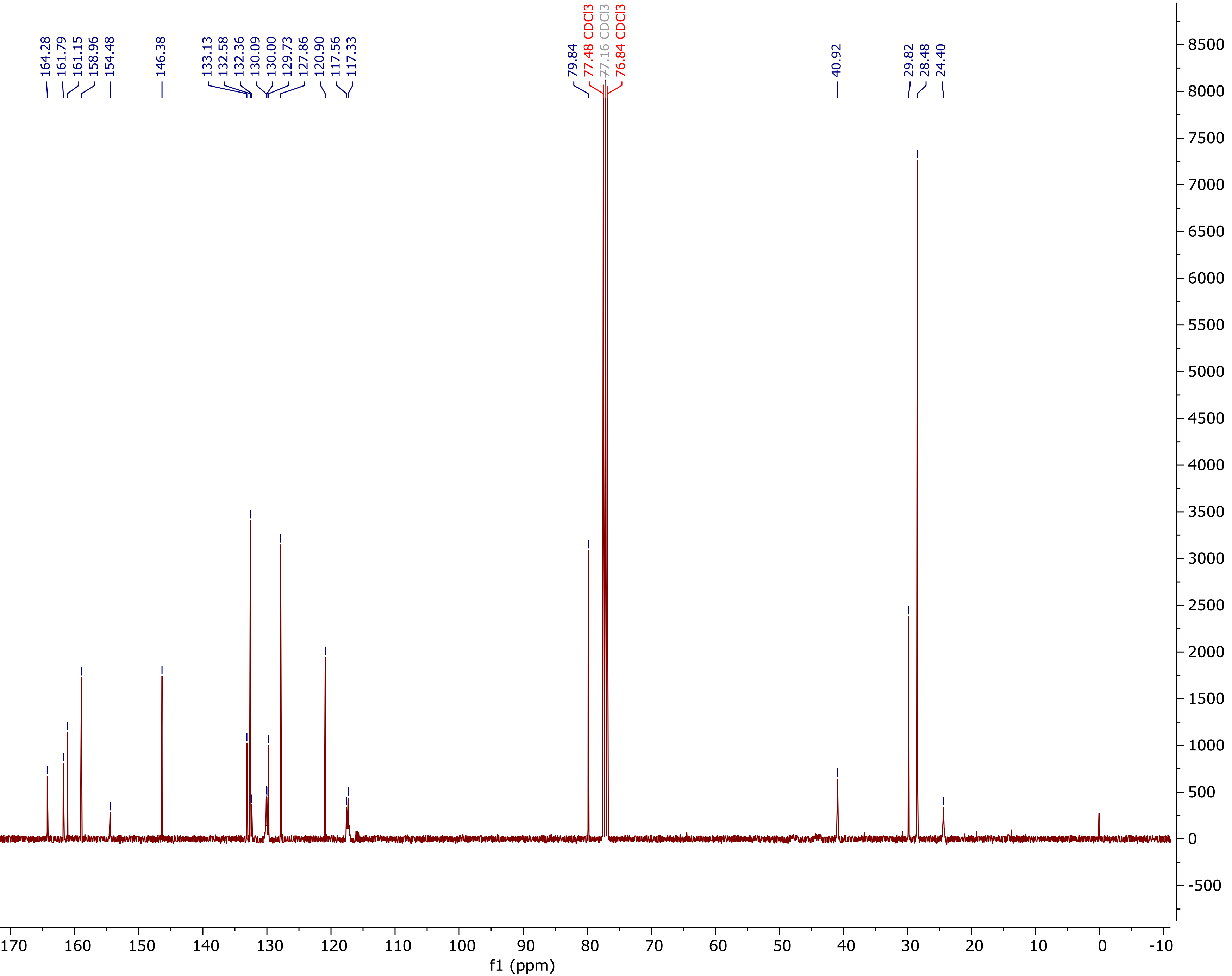

400 MHz, $CDCl_3$

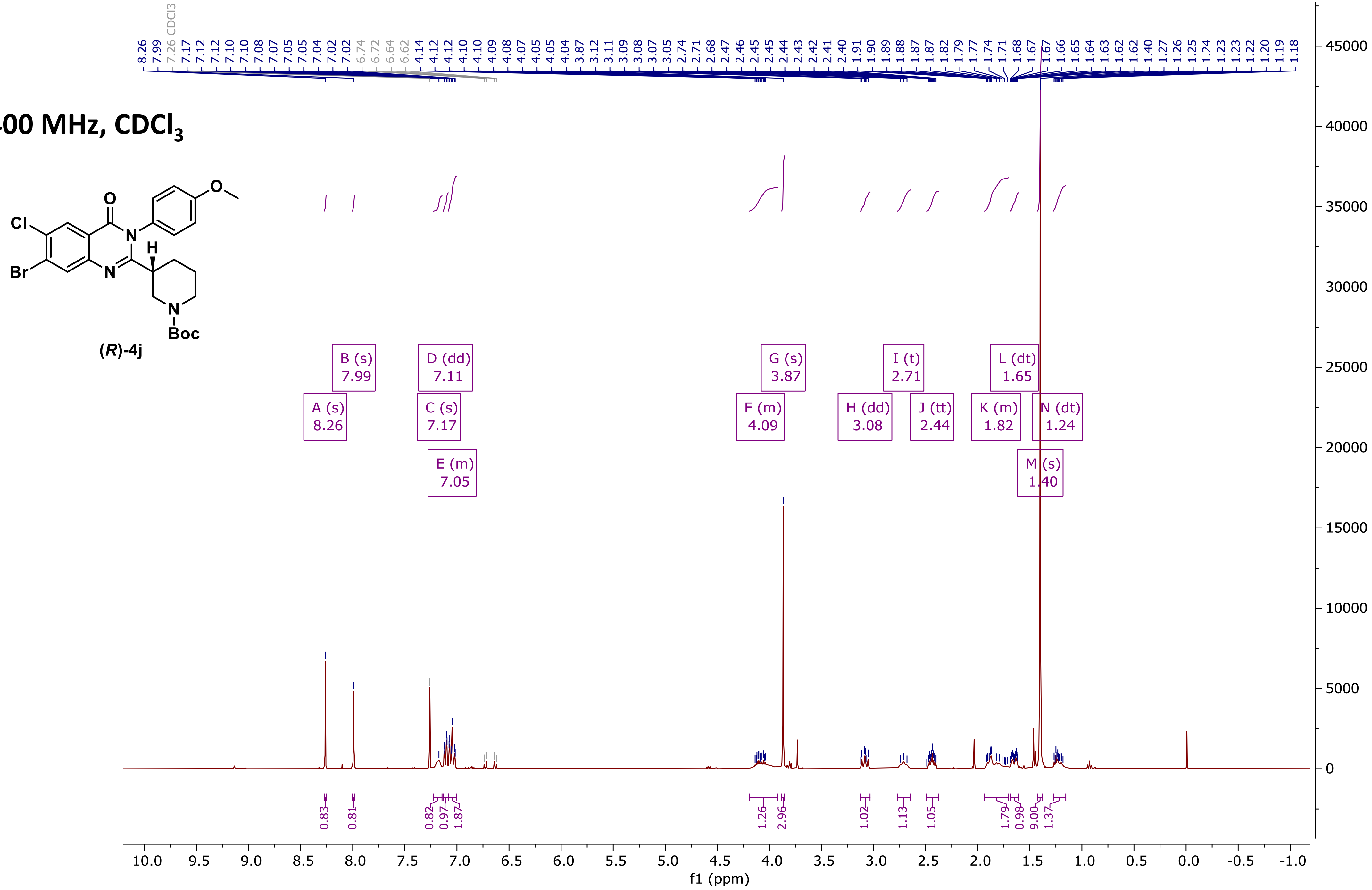

(*R*)-4j

101 MHz, $CDCl_3$

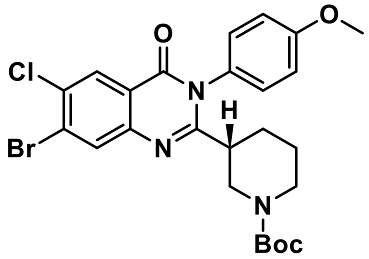

(*R*)-4j

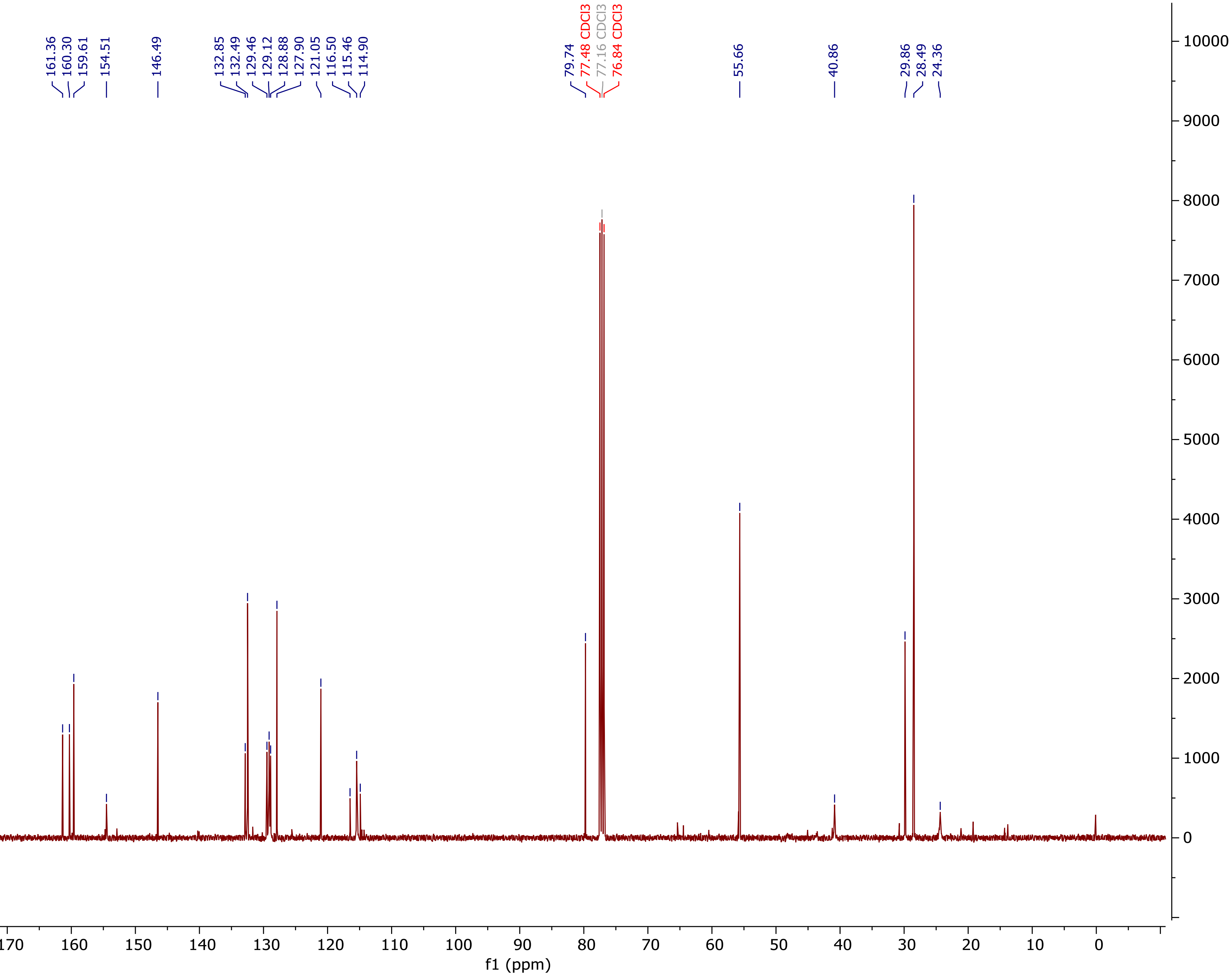

400 MHz, $CDCl_3$

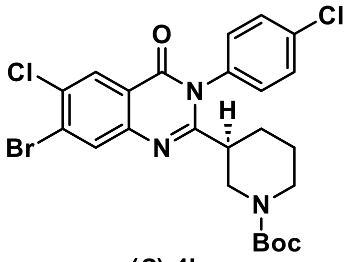

(*S*)-4k

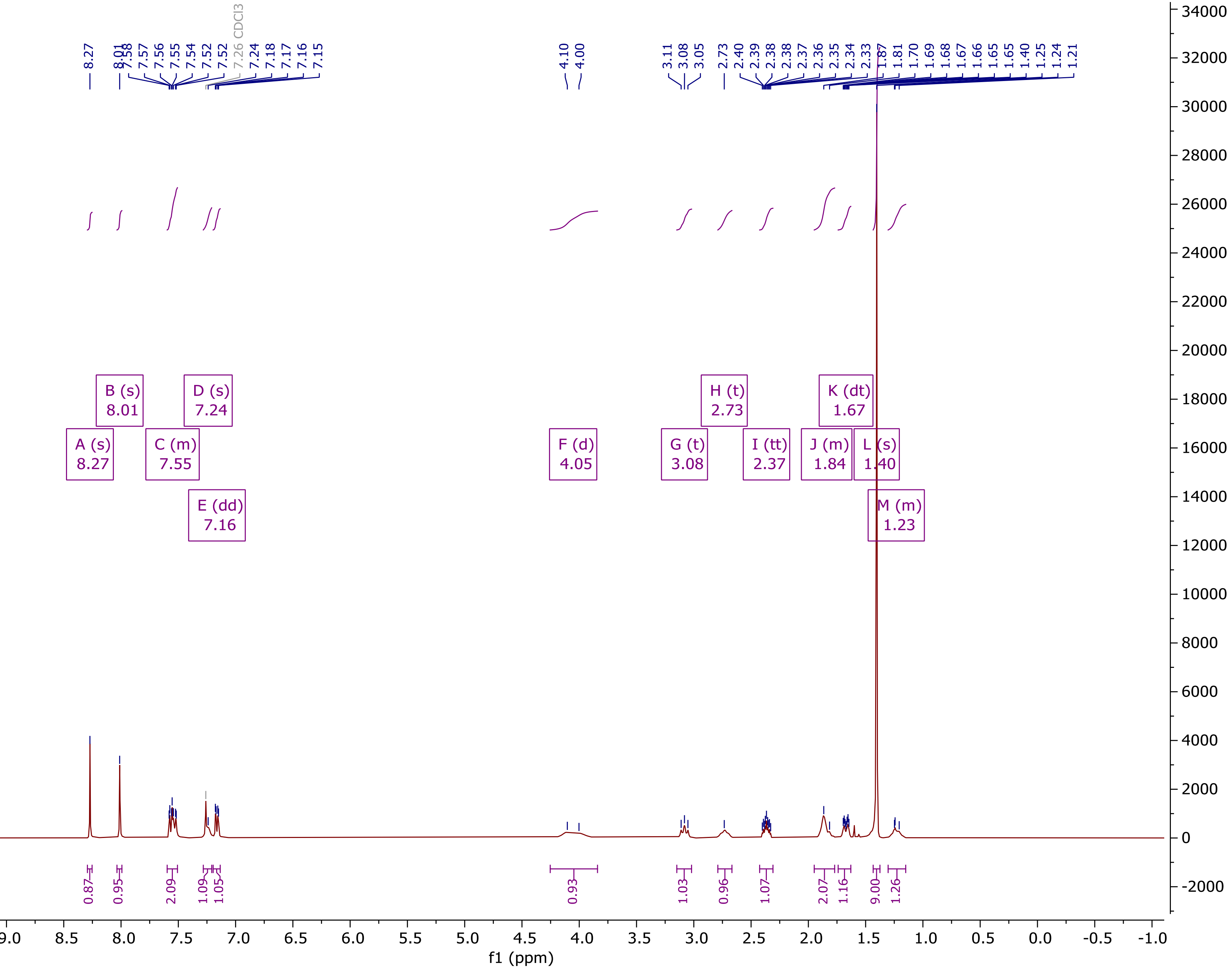

101 MHz, $CDCl_3$

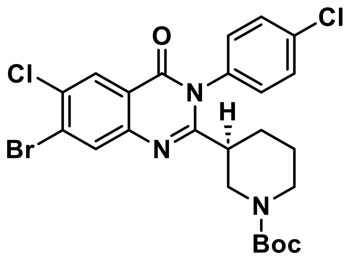

(*S*)-4k

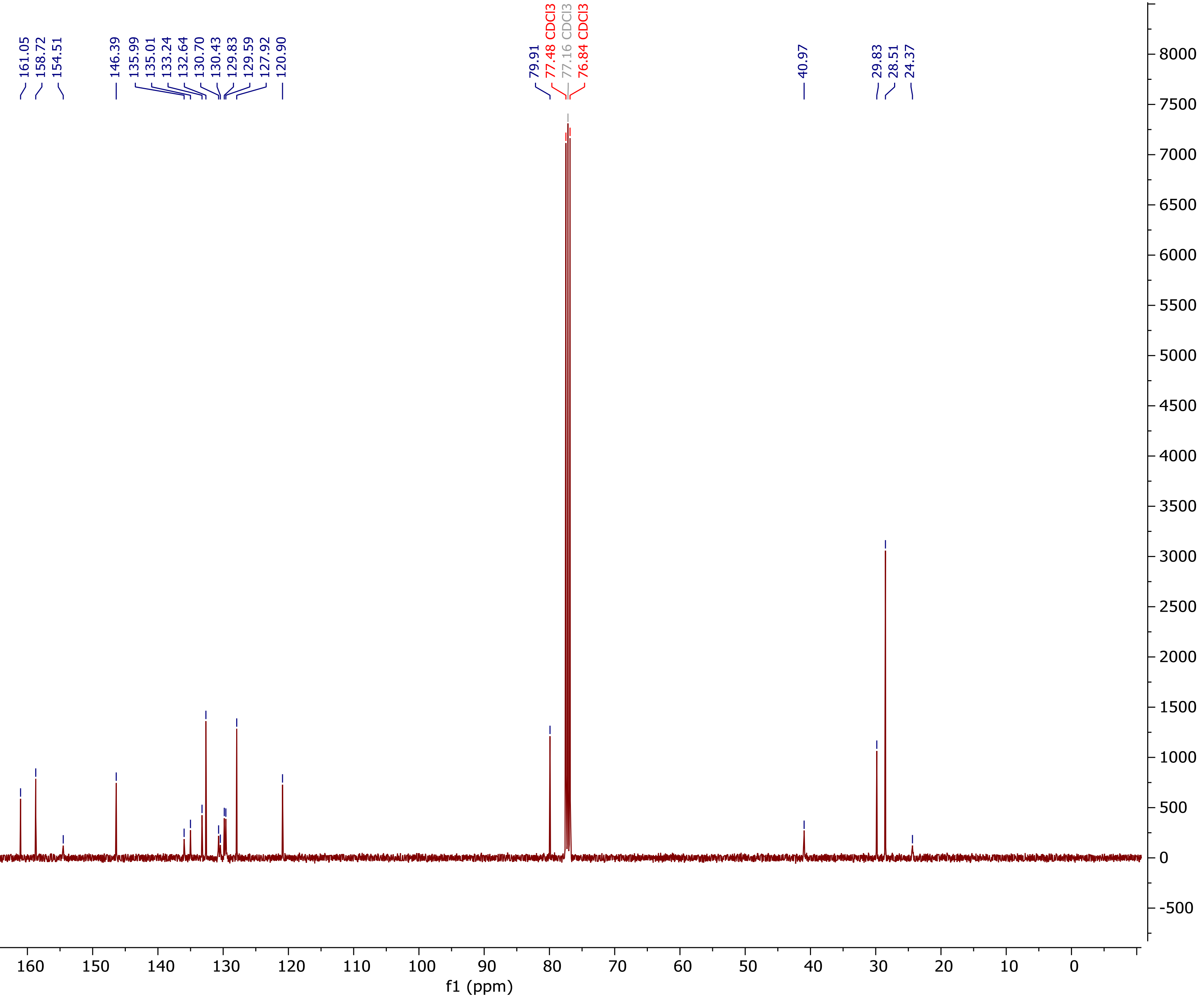

400 MHz, $CDCl_3$

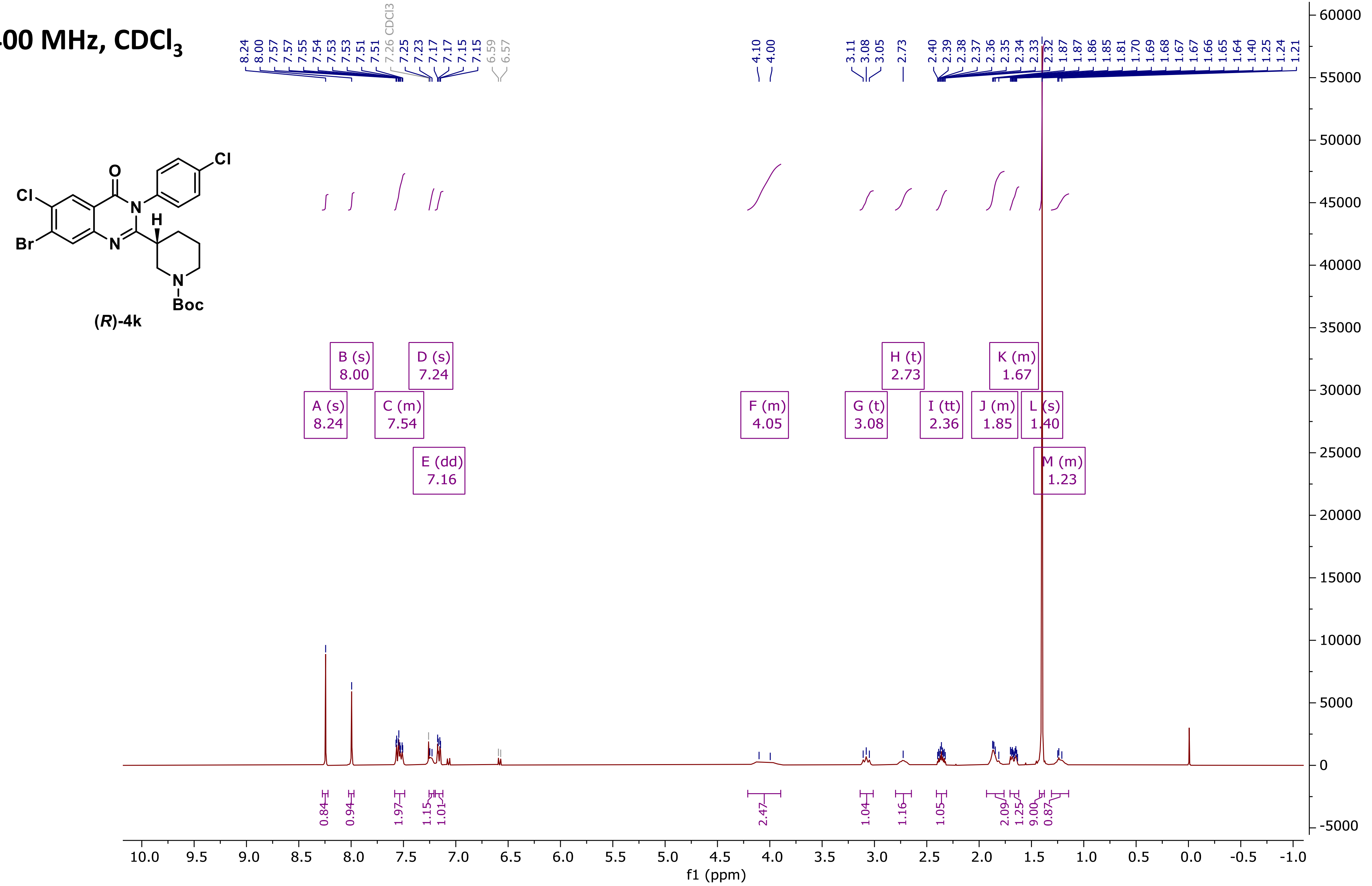

101 MHz, $CDCl_3$

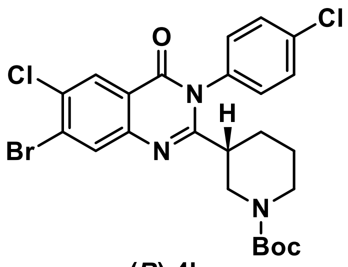

(*R*)-4k

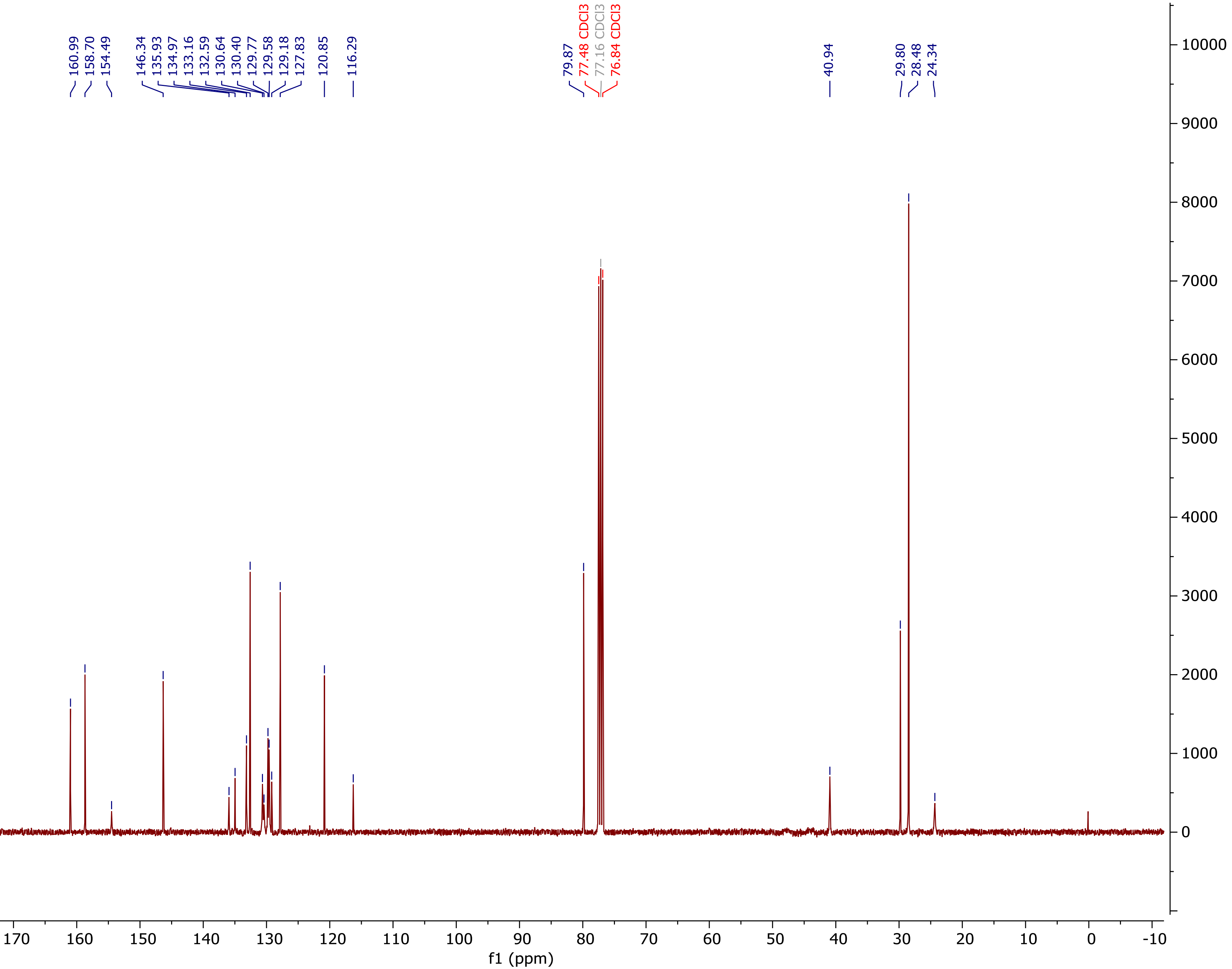

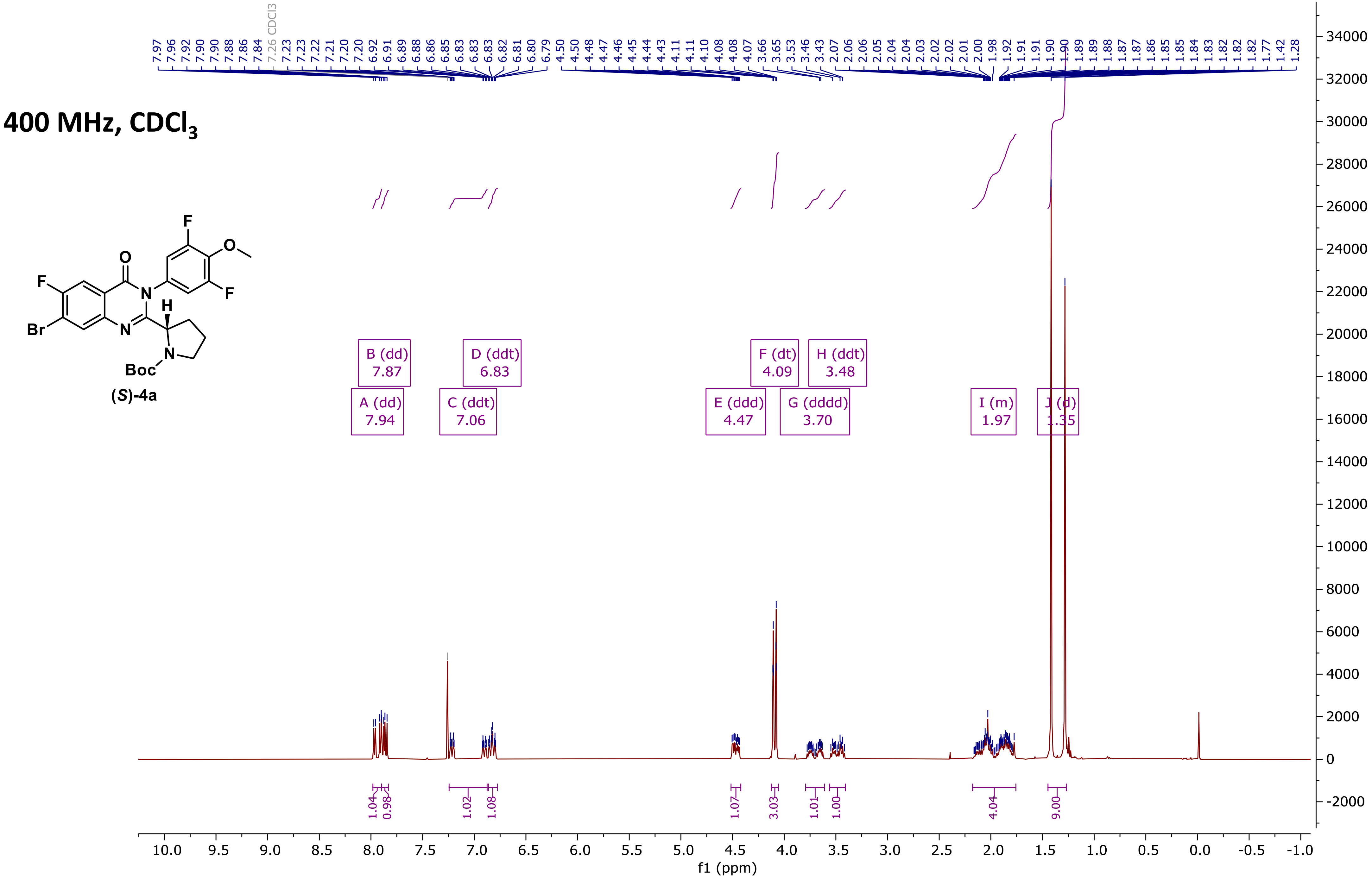
400 MHz, CDCl3
(S)-4a
A (dd) 7.94
B (dd) 7.87
C (ddt) 7.06
D (ddt) 6.83
E (ddd) 4.47
F (dt) 4.09
G (dddd) 3.70
H (ddt) 3.48
I (m) 1.97
J (d) 1.35
f1 (ppm)

101 MHz, CDCl$_3$

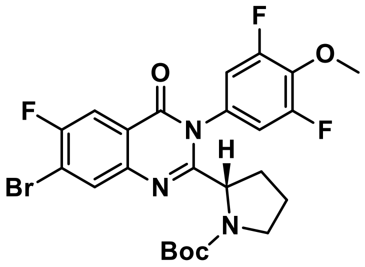

(*S*)-4a

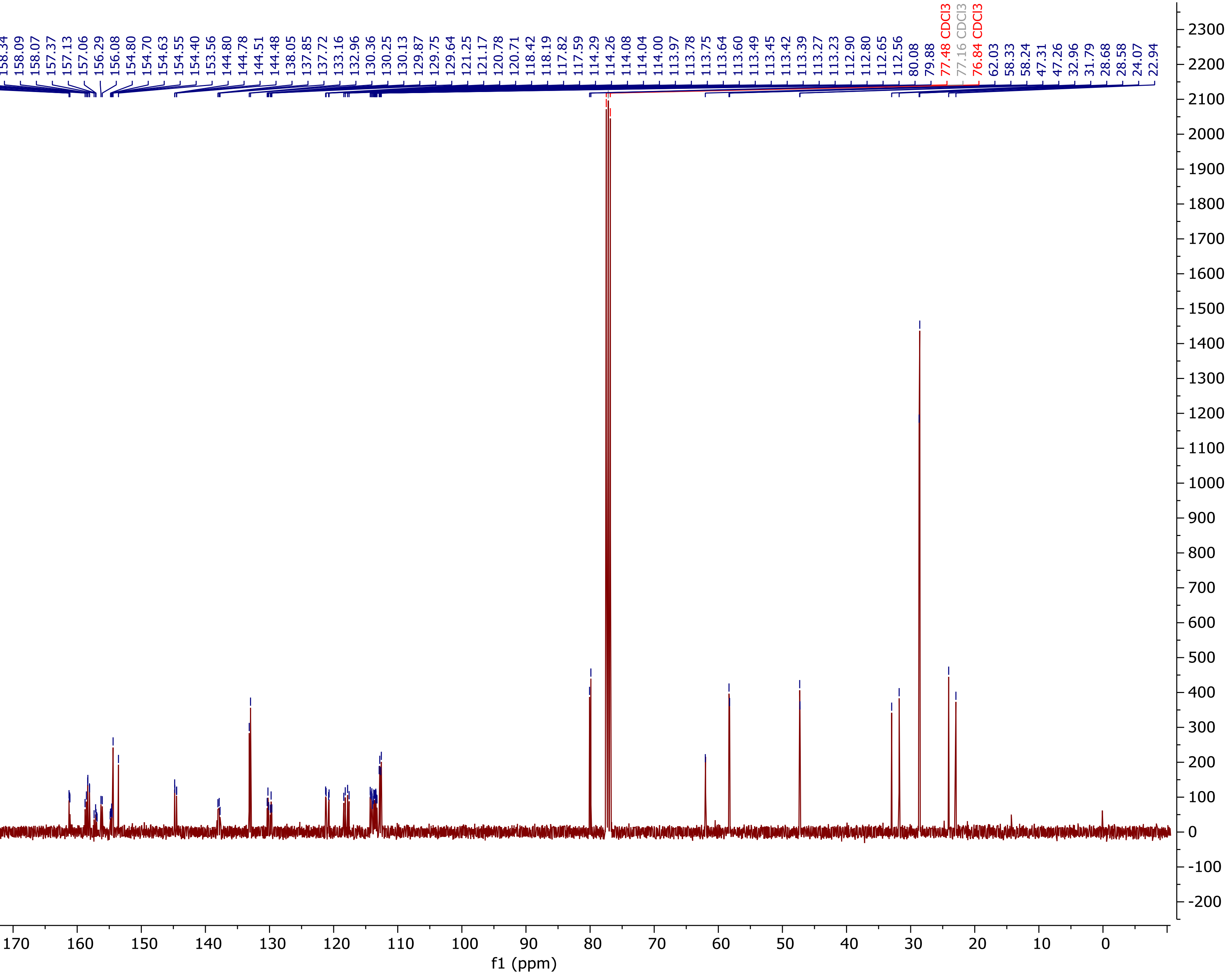

400 MHz, $CDCl_3$

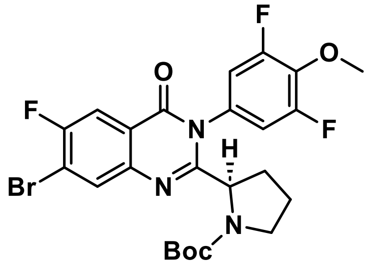

(*R*)-4a

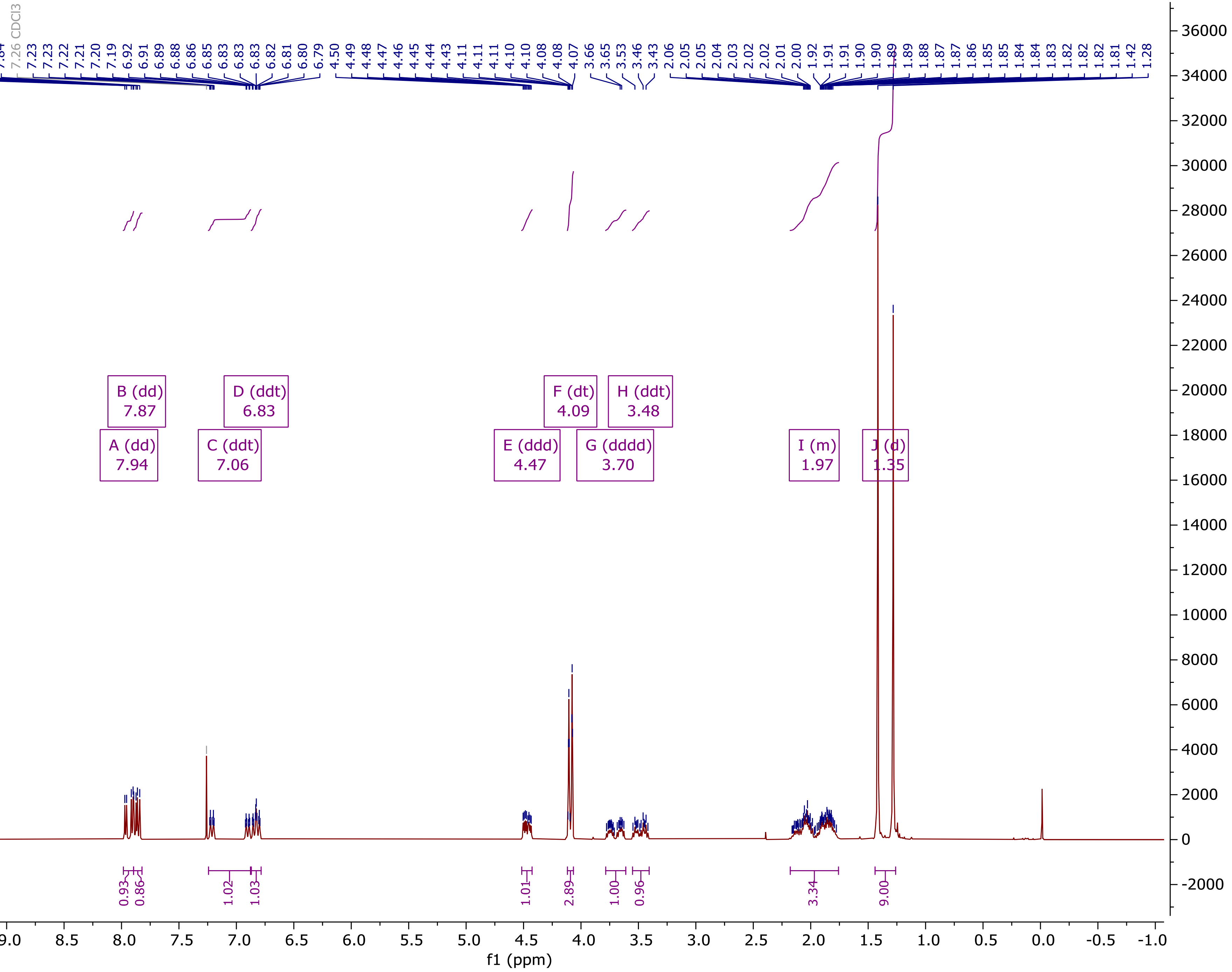

101 MHz, $CDCl_3$

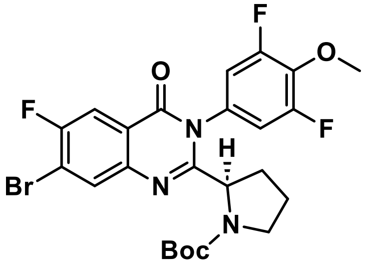

(*R*)-4a

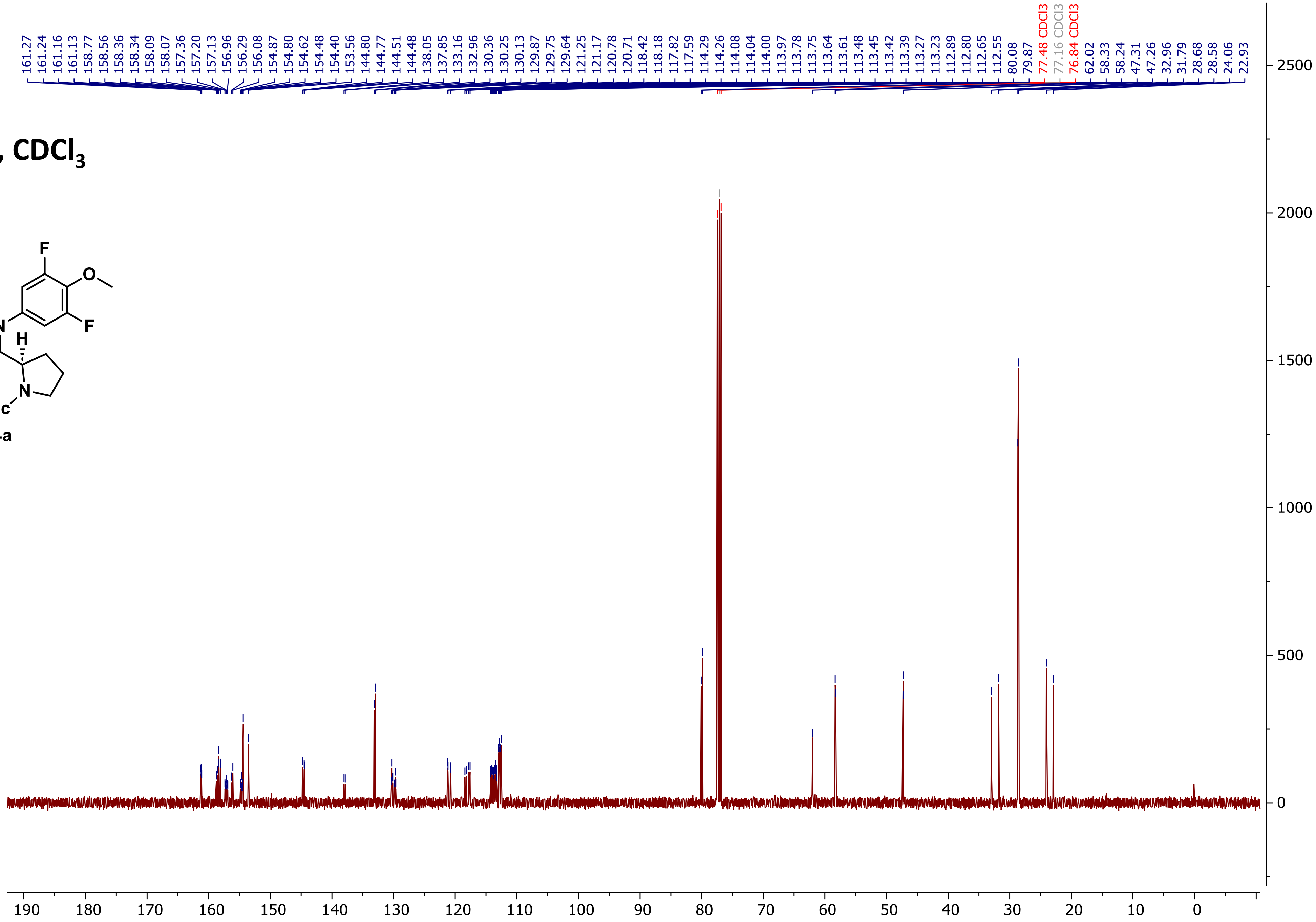

400 MHz, CDCl$_3$

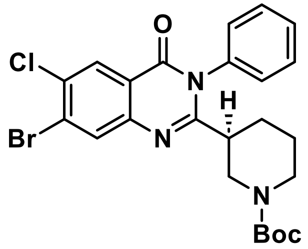

(*S*)-4b

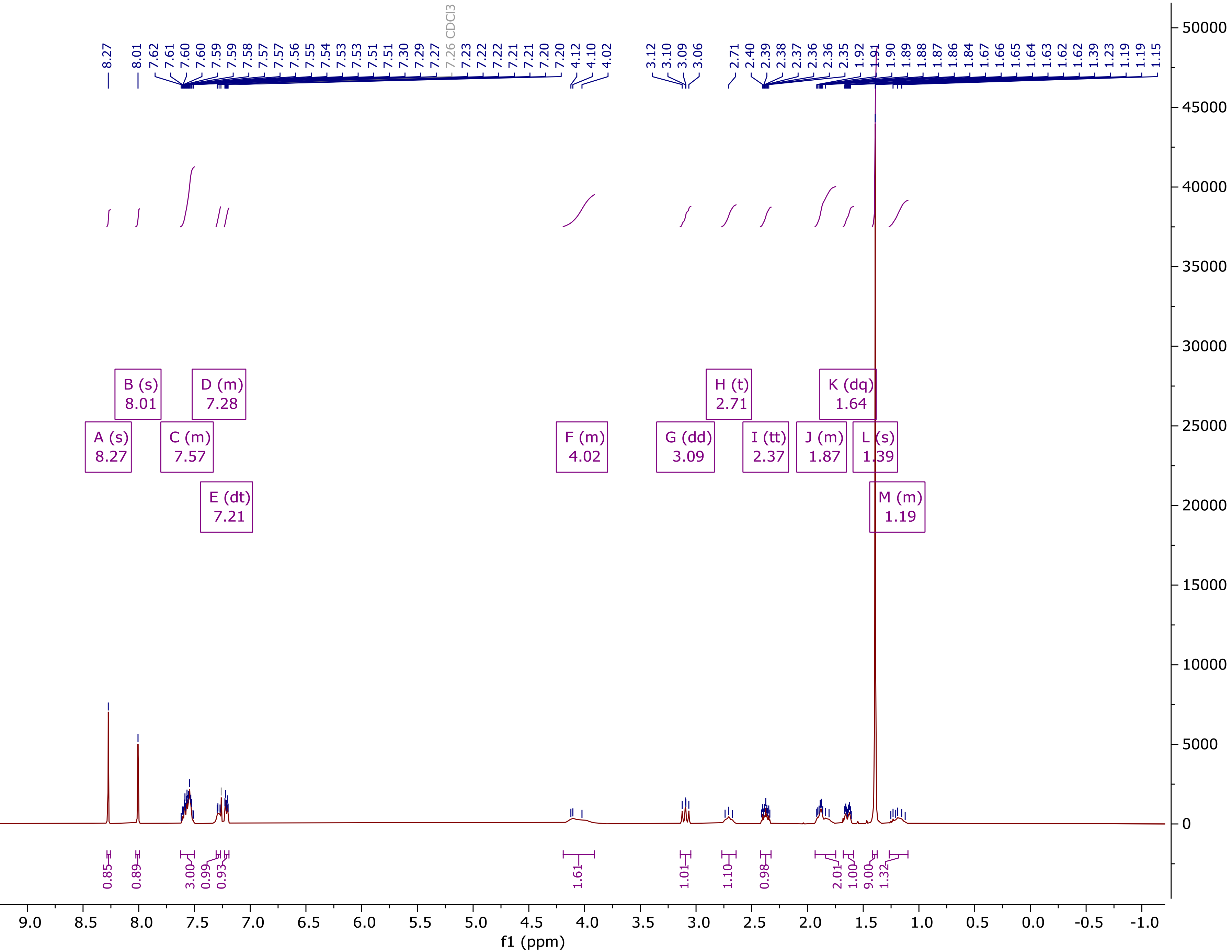

101 MHz, $CDCl_3$

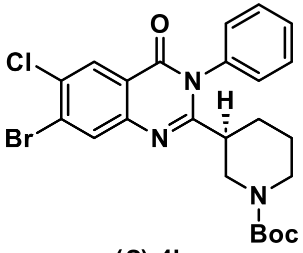

(*S*)-4b

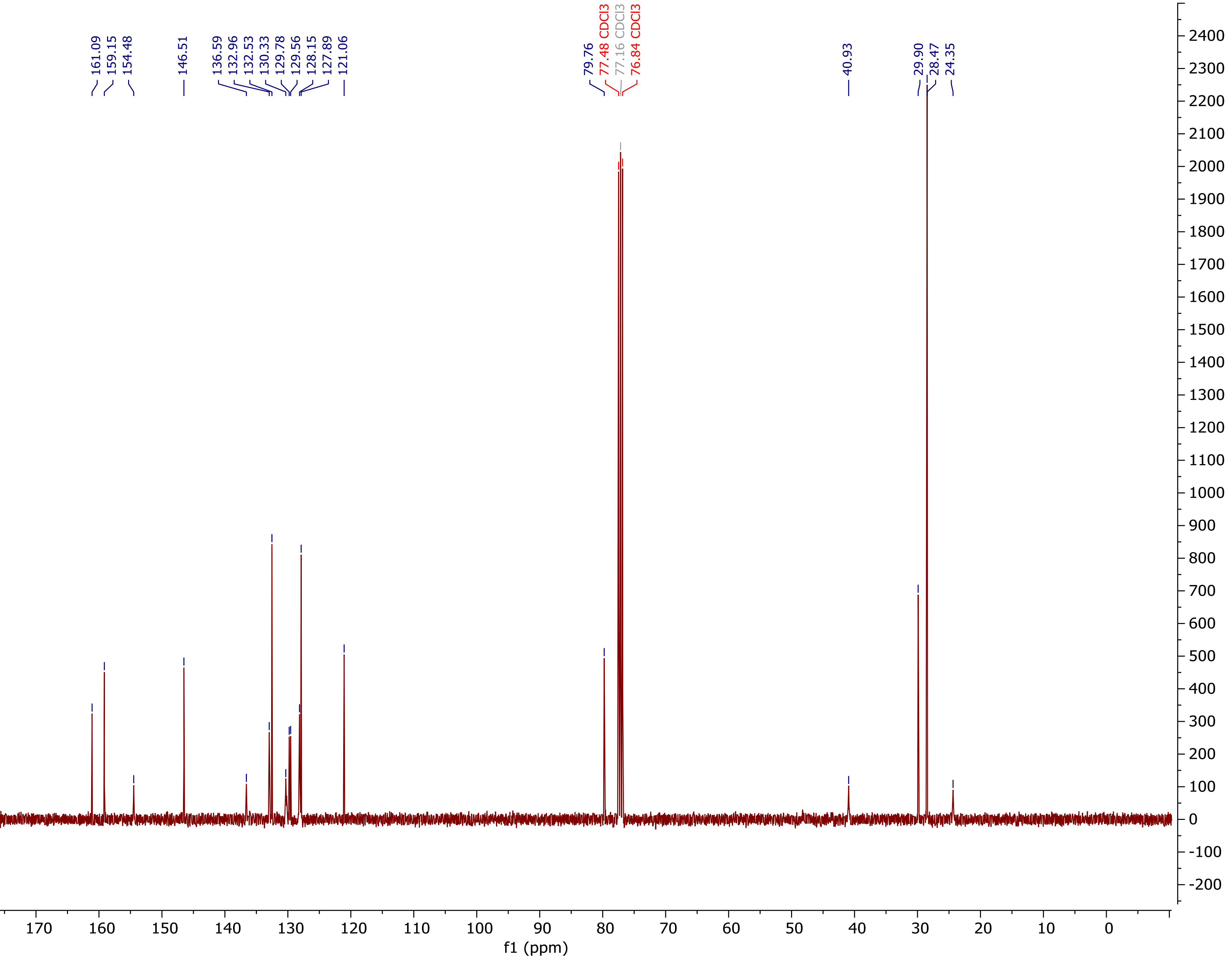

400 MHz, $CDCl_3$

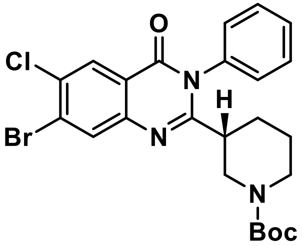

(*R*)-4b

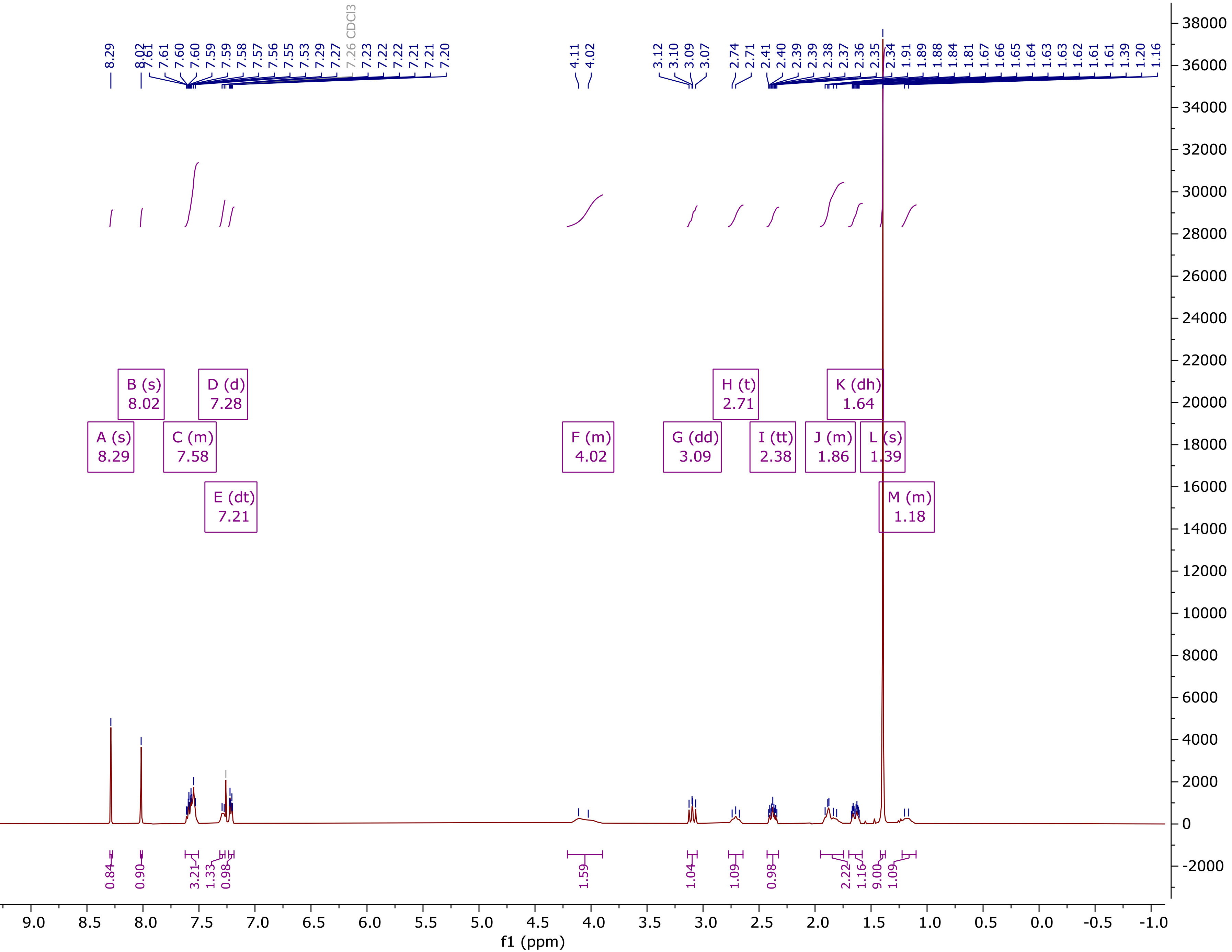

101 MHz, $CDCl_3$

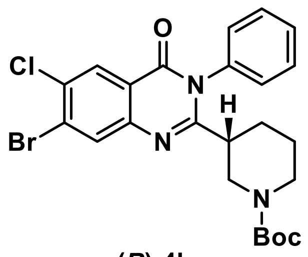

(*R*)-4b

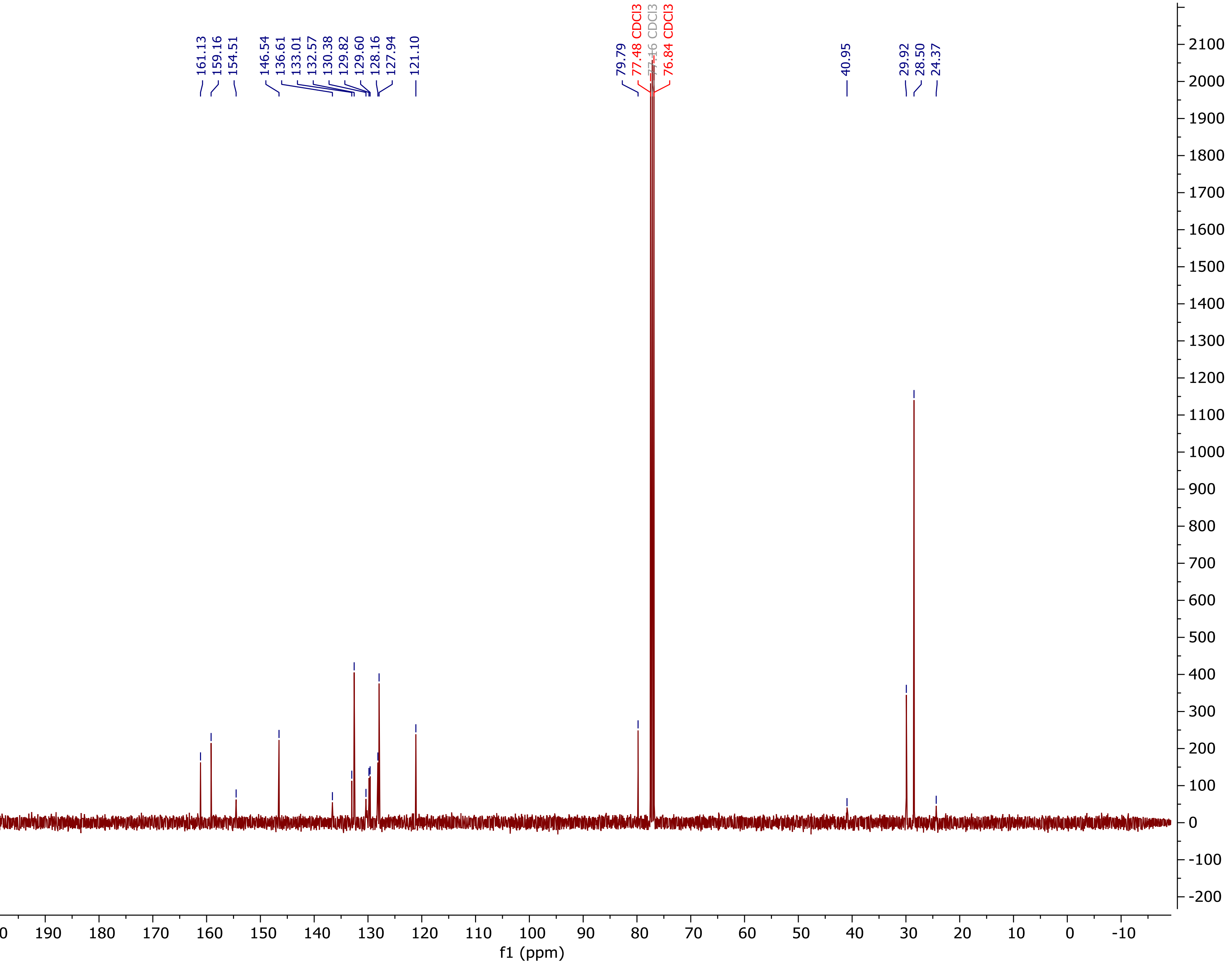

400 MHz, $CDCl_3$

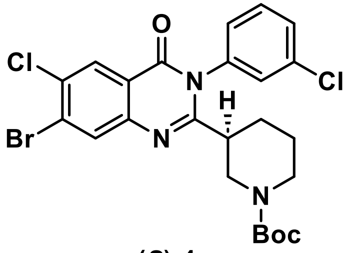

(*S*)-4c

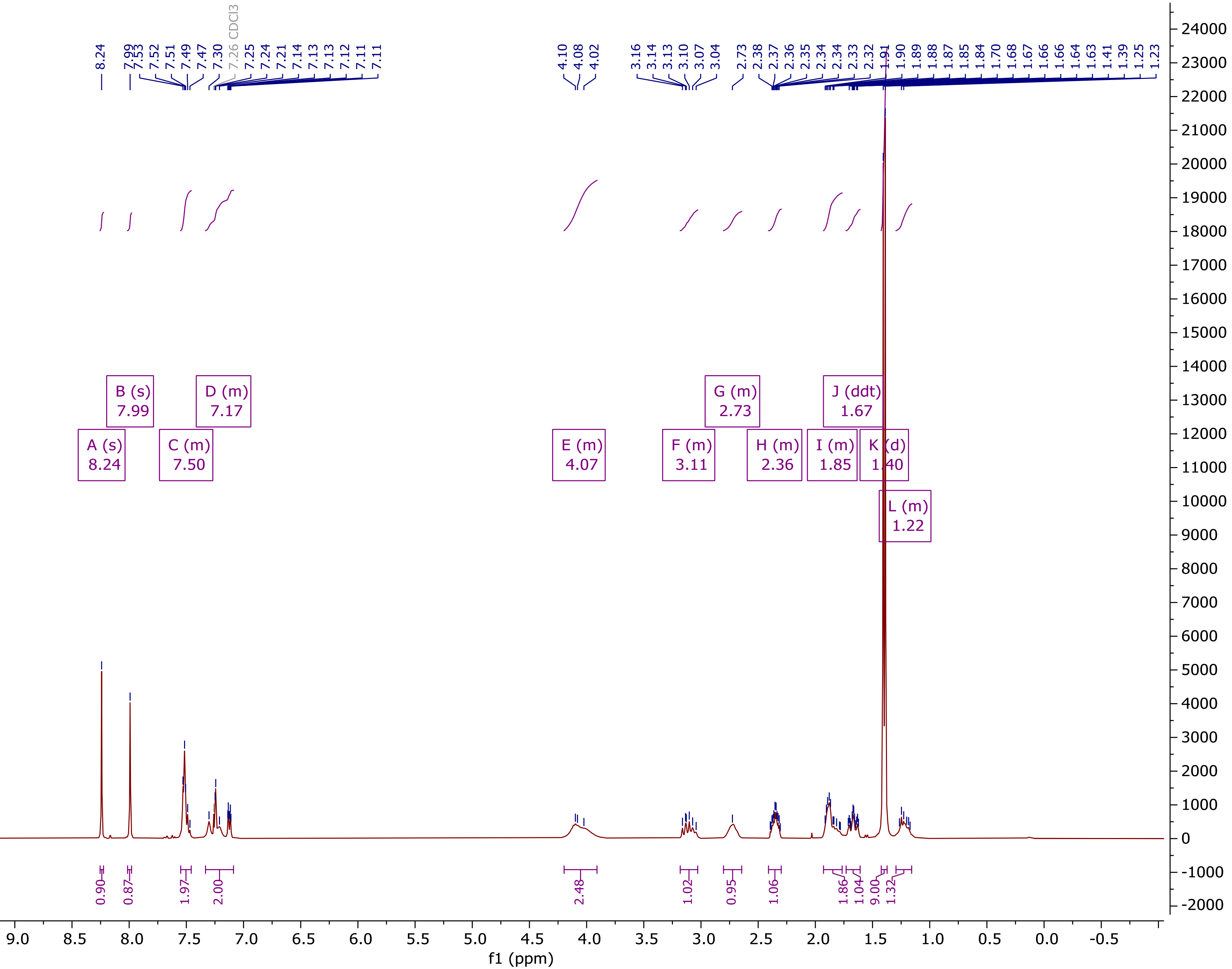

101 MHz, $CDCl_3$

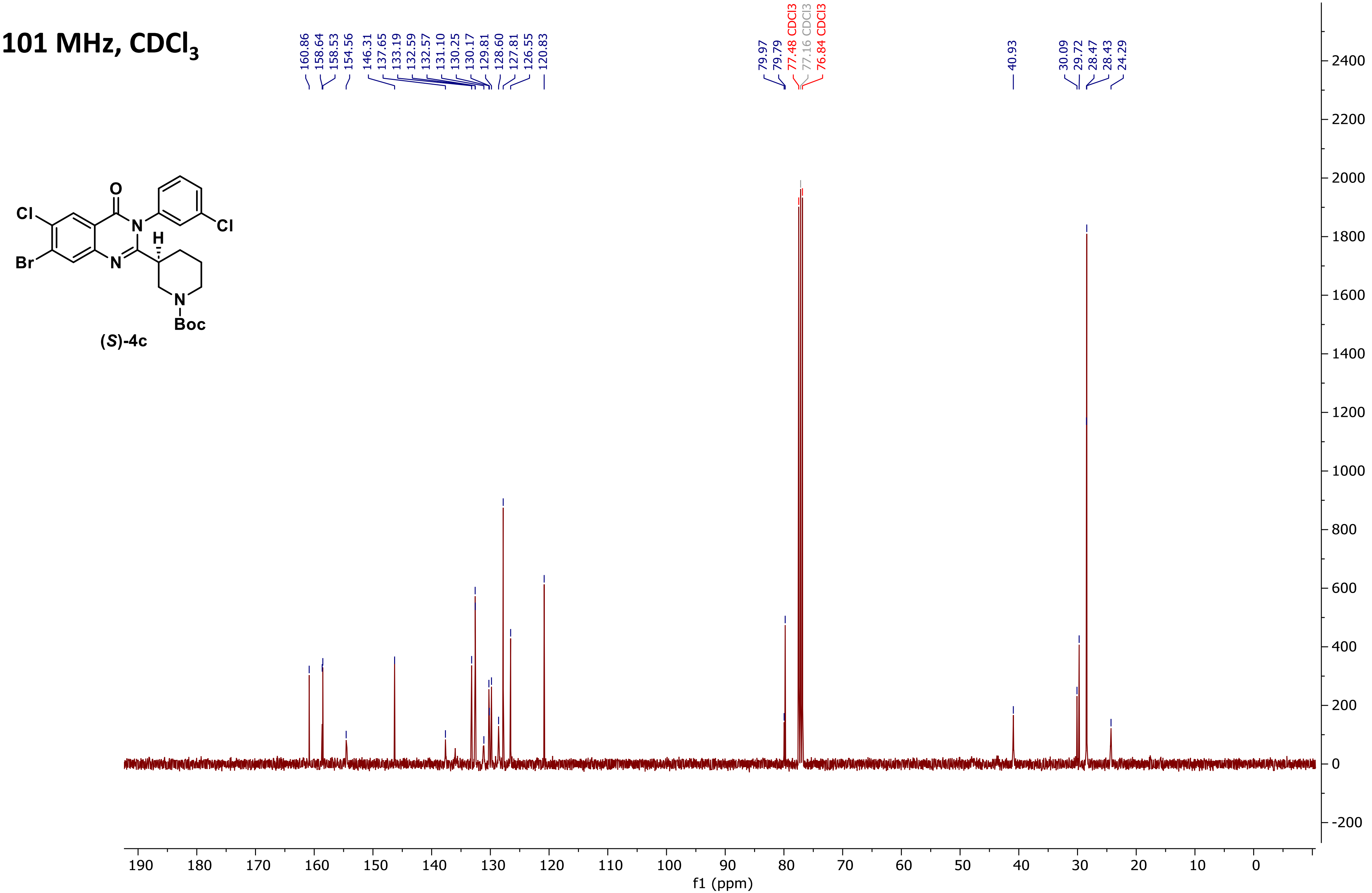

(*S*)-4c

400 MHz, $CDCl_3$

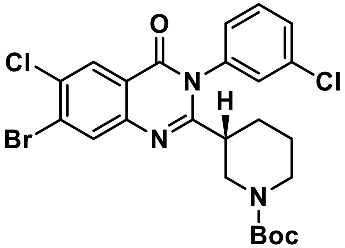

(*R*)-4c

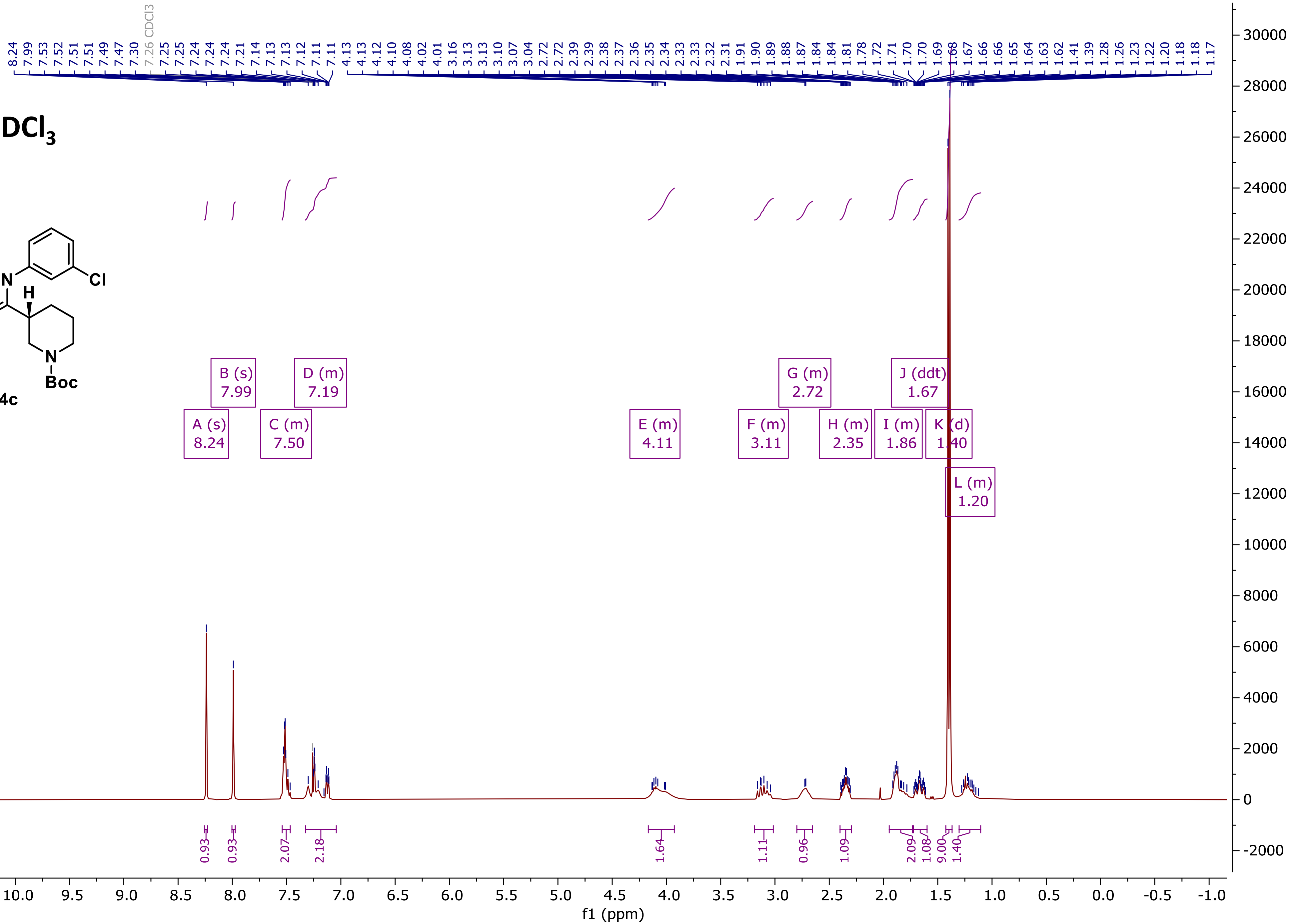

101 MHz, $CDCl_3$

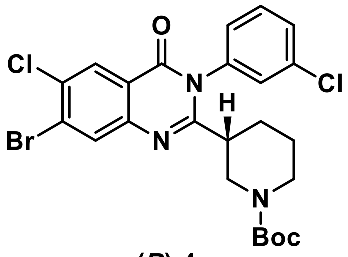

(*R*)-4c

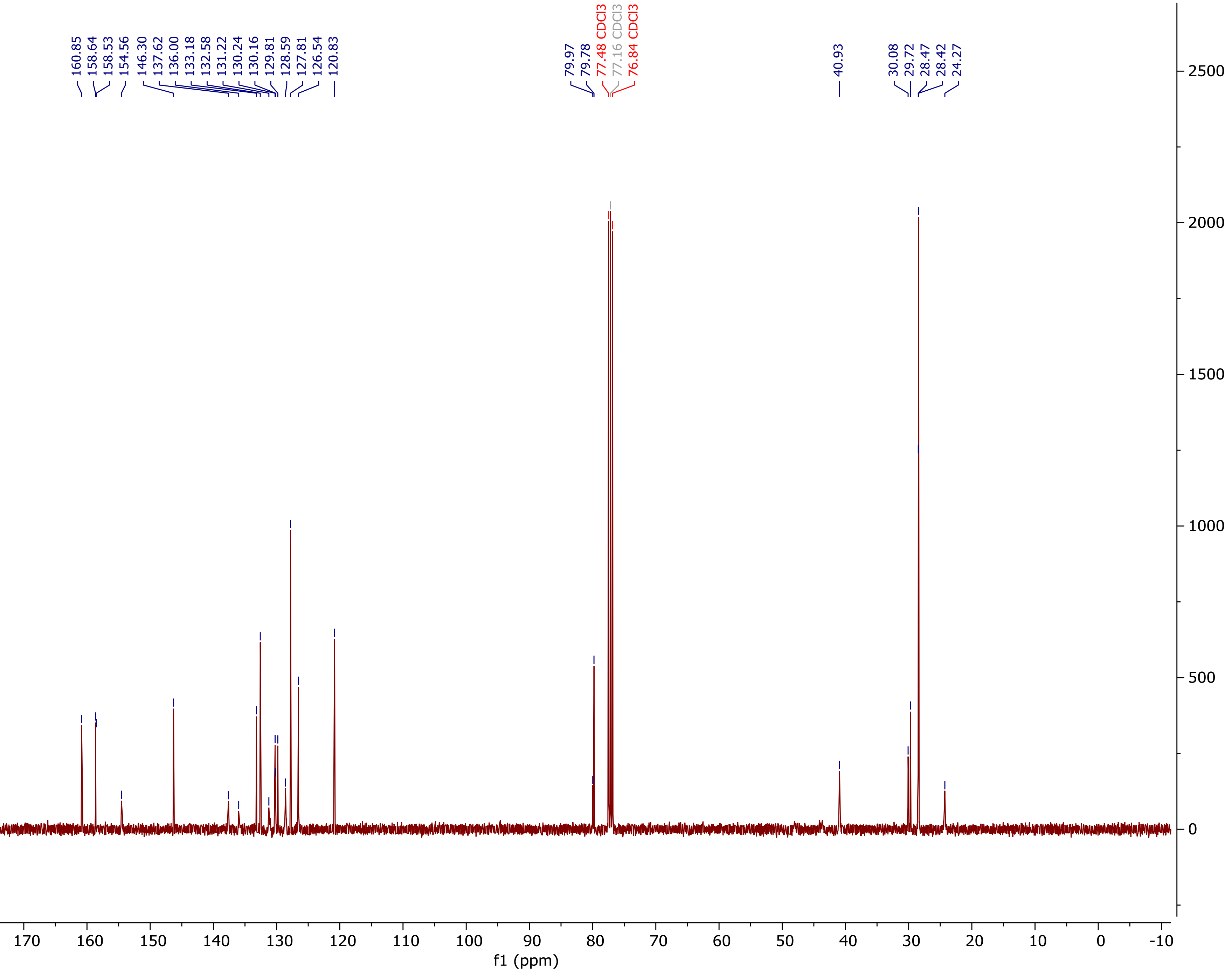

400 MHz, DMSO-d_6

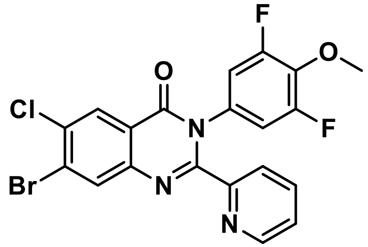

4o

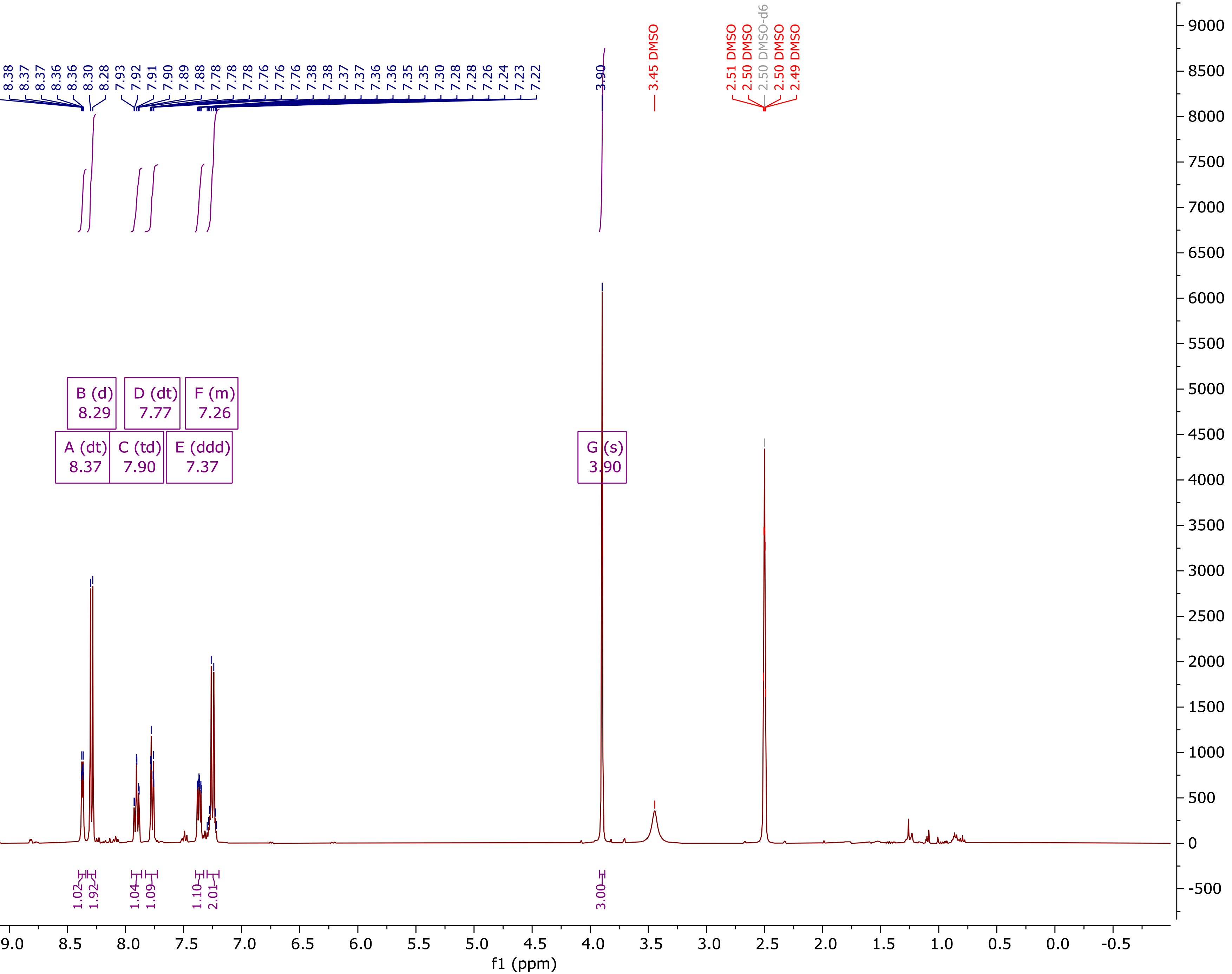

101 MHz, DMSO-d$_6$

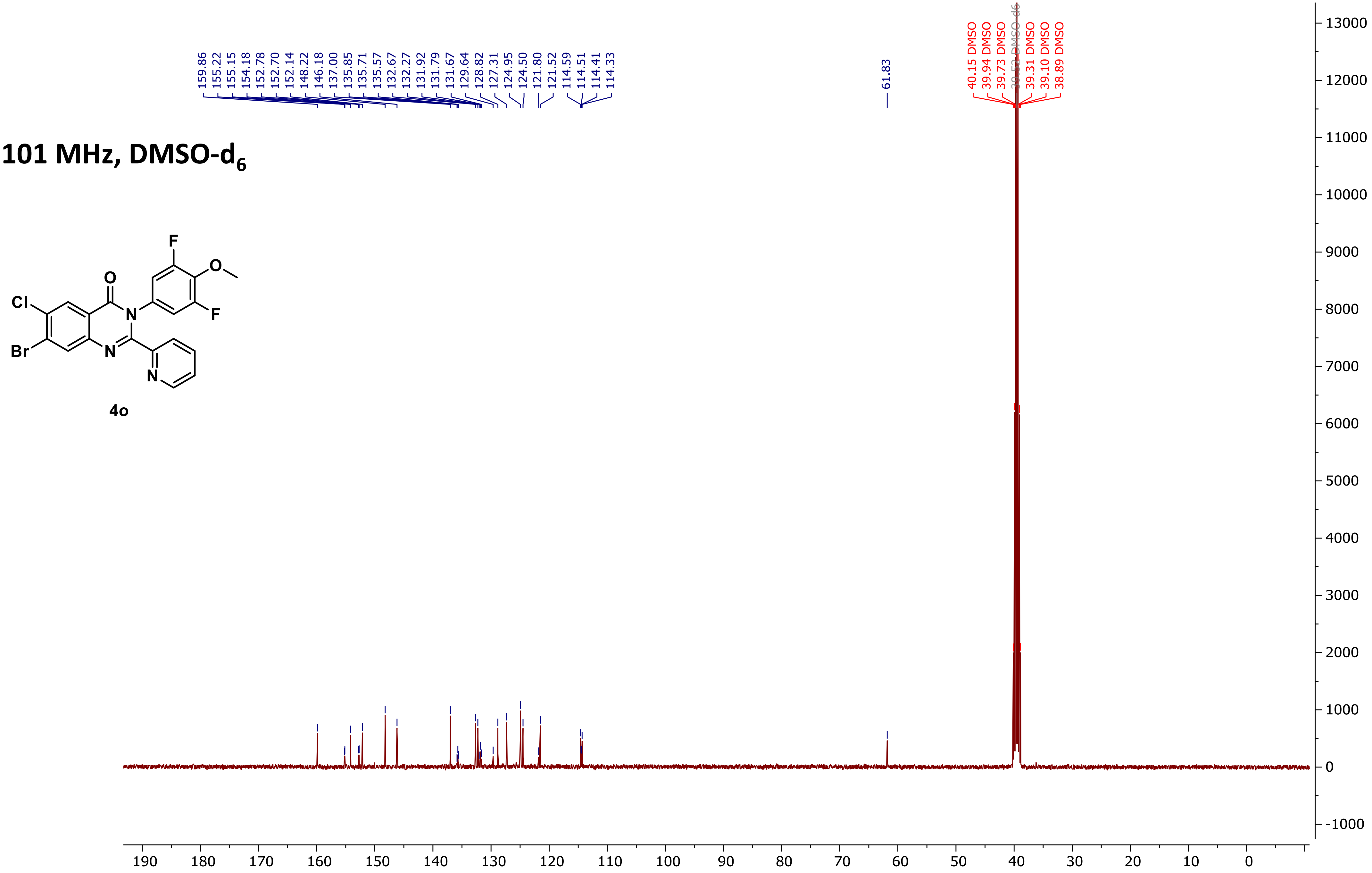

4o

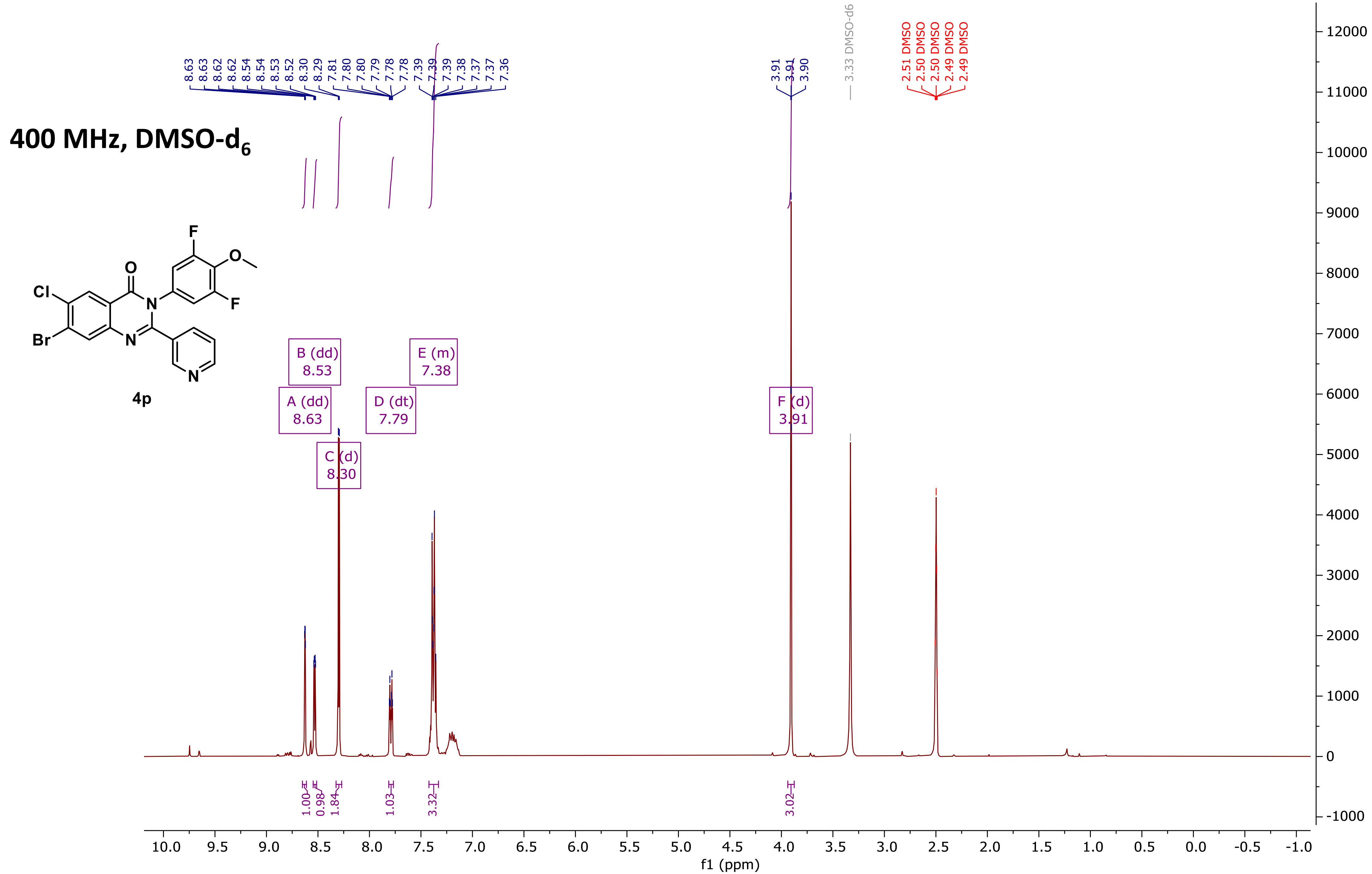
400 MHz, DMSO-d6
4p
A (dd) 8.63
B (dd) 8.53
C (d) 8.30
D (dt) 7.79
E (m) 7.38
F (d) 3.91
3.33 DMSO-d6
2.51 DMSO
2.50 DMSO
2.50 DMSO
2.49 DMSO
2.49 DMSO
1.00
0.98
1.84
1.03
3.32
3.02
f1 (ppm)

101 MHz, DMSO-d$_6$

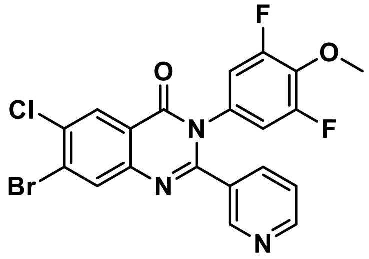

4p

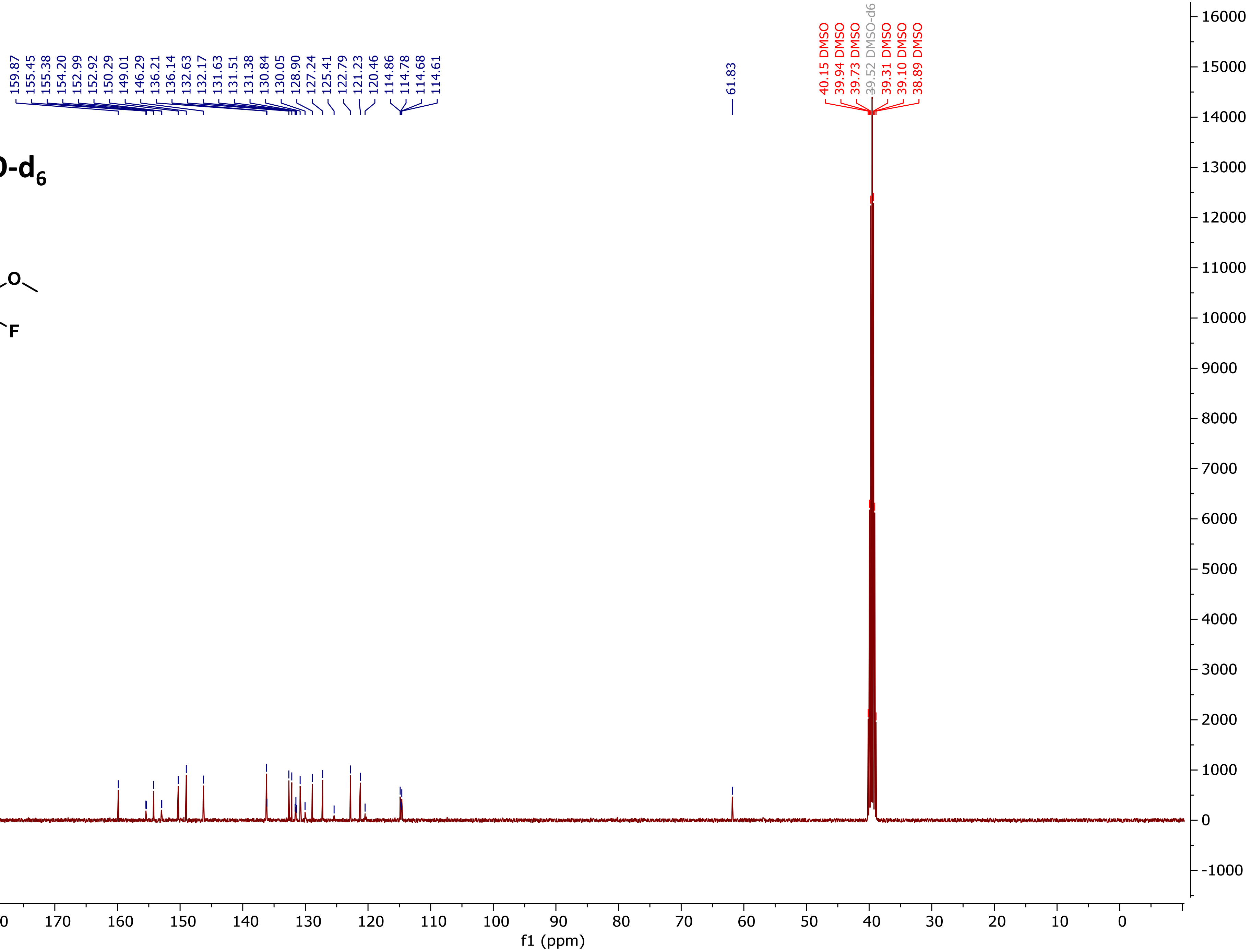

400 MHz, DMSO-d_6

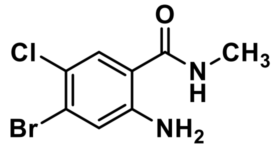

5a

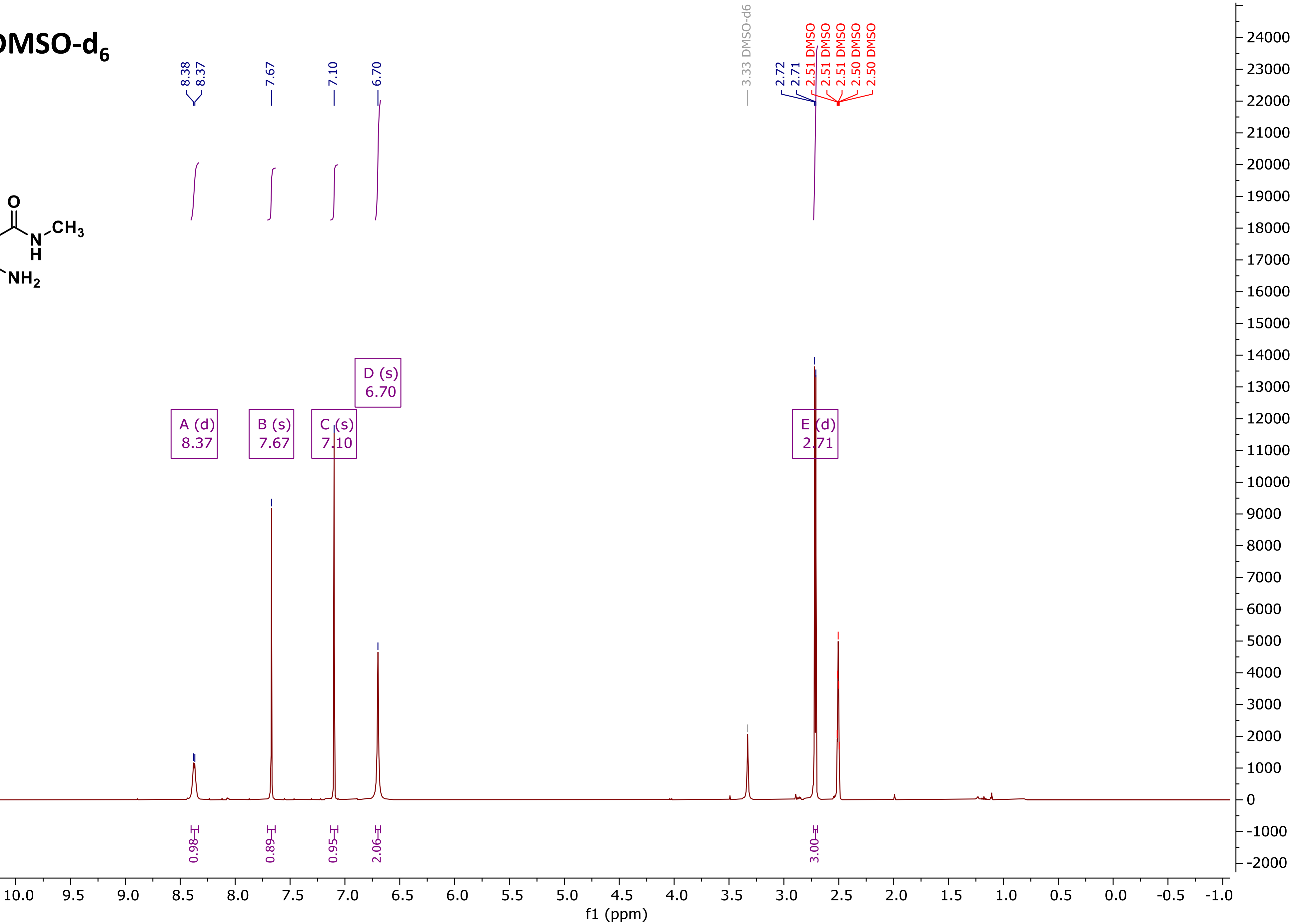

101 MHz, DMSO-d$_6$

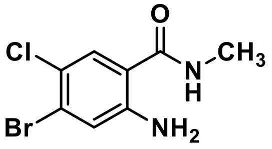

5a

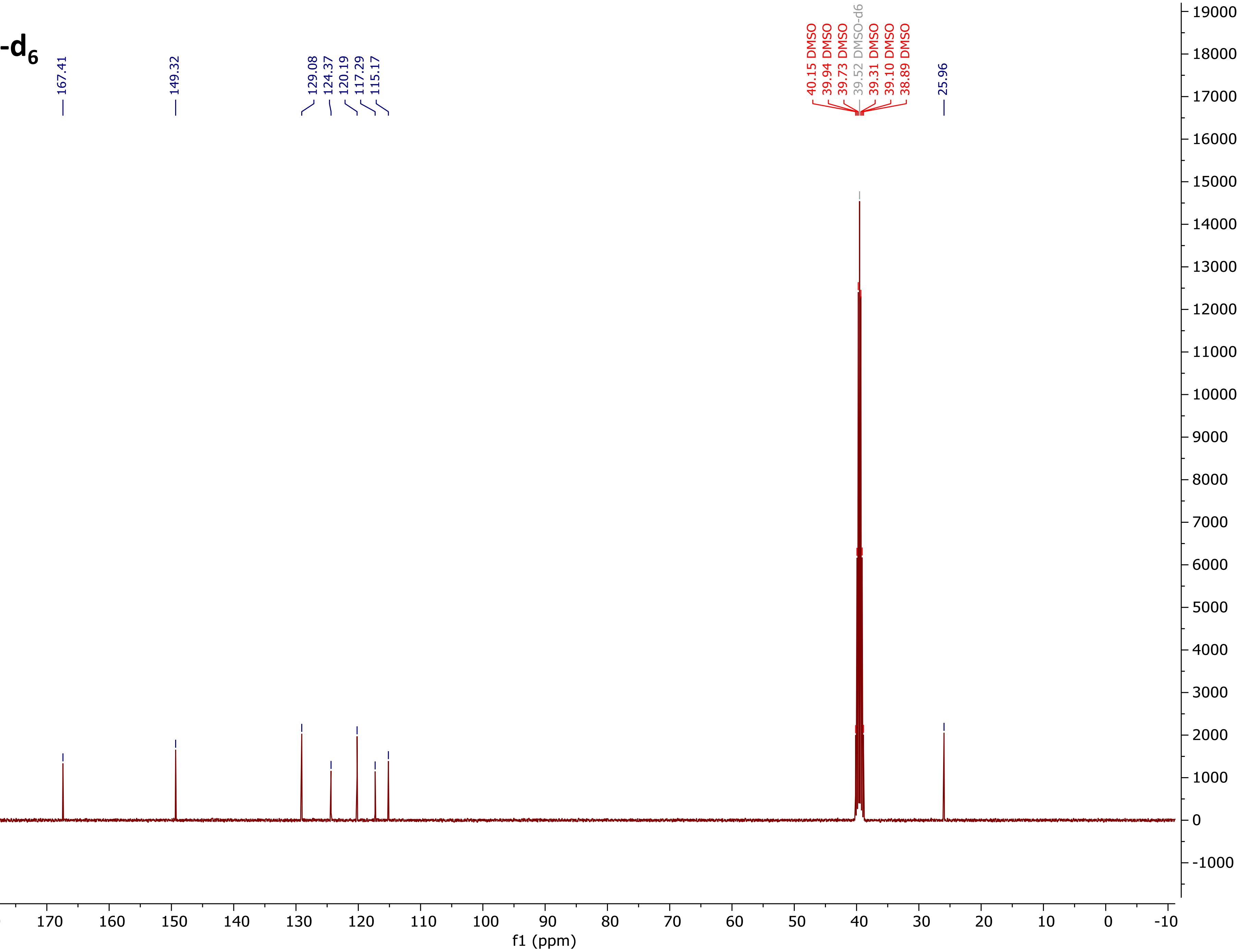

400 MHz, $CDCl_3$

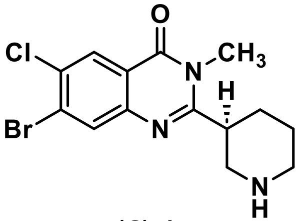

(*S*)-4n

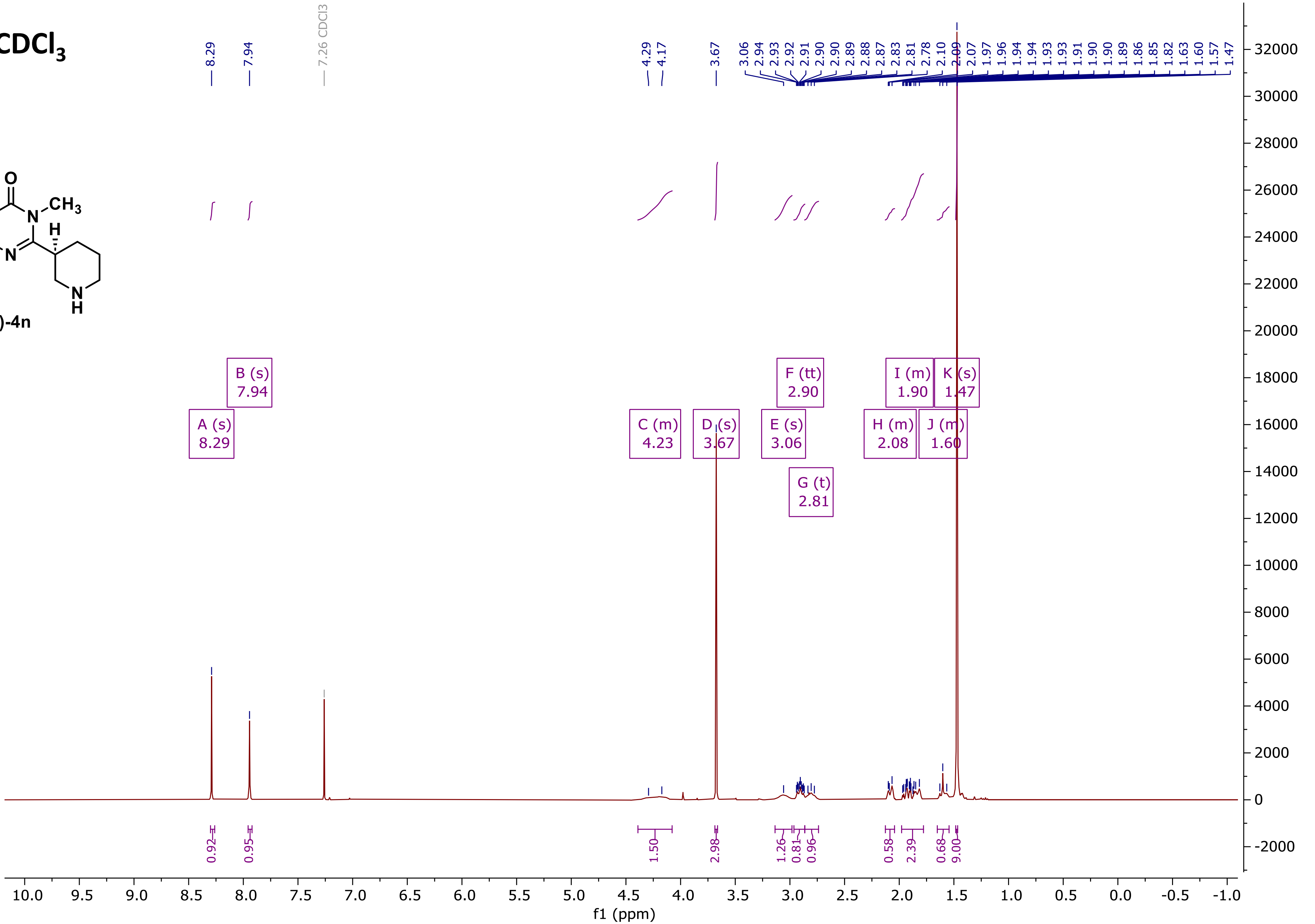

101 MHz, $CDCl_3$

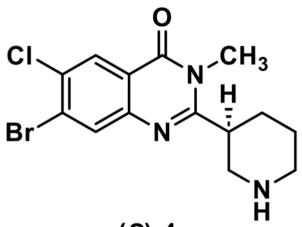

(*S*)-4n

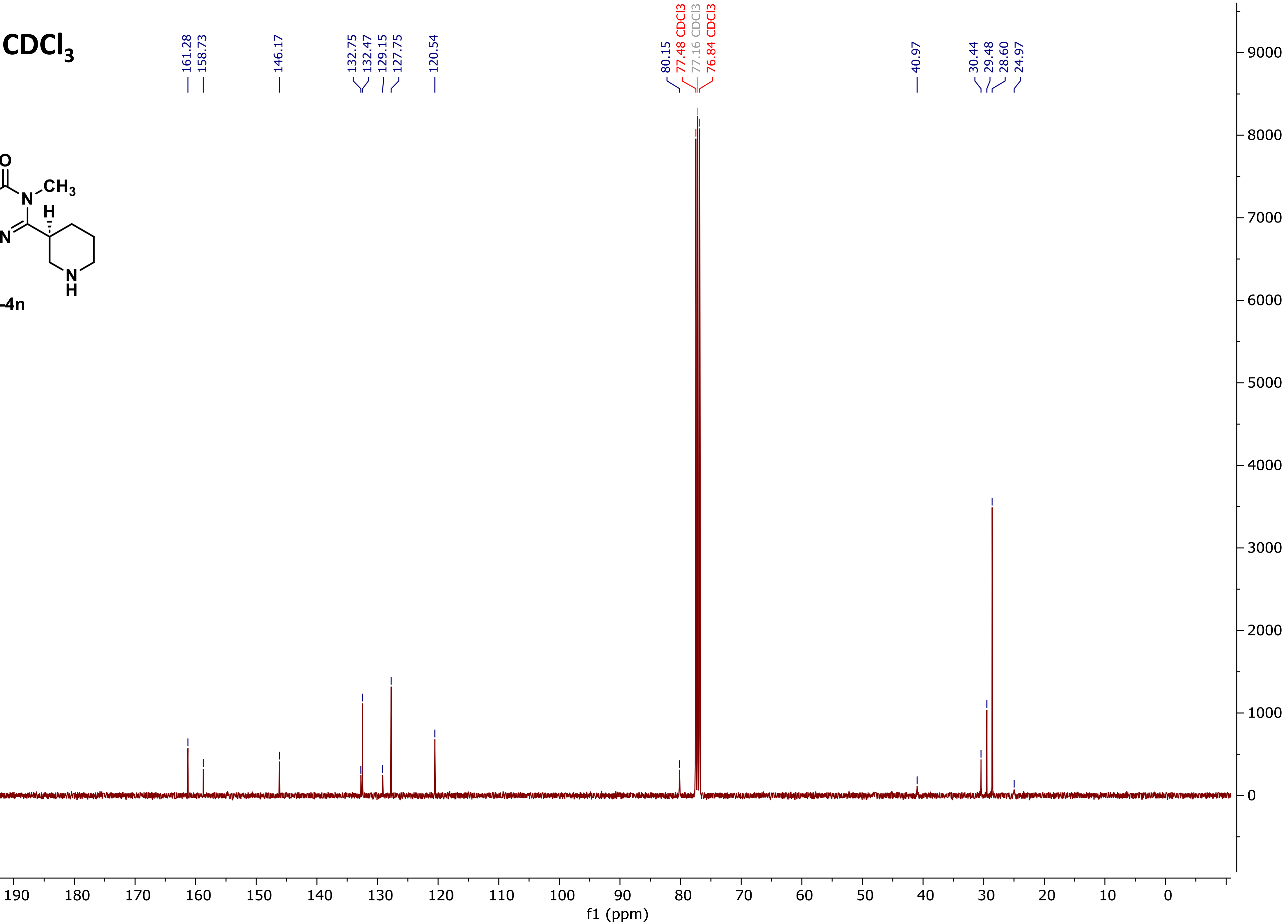

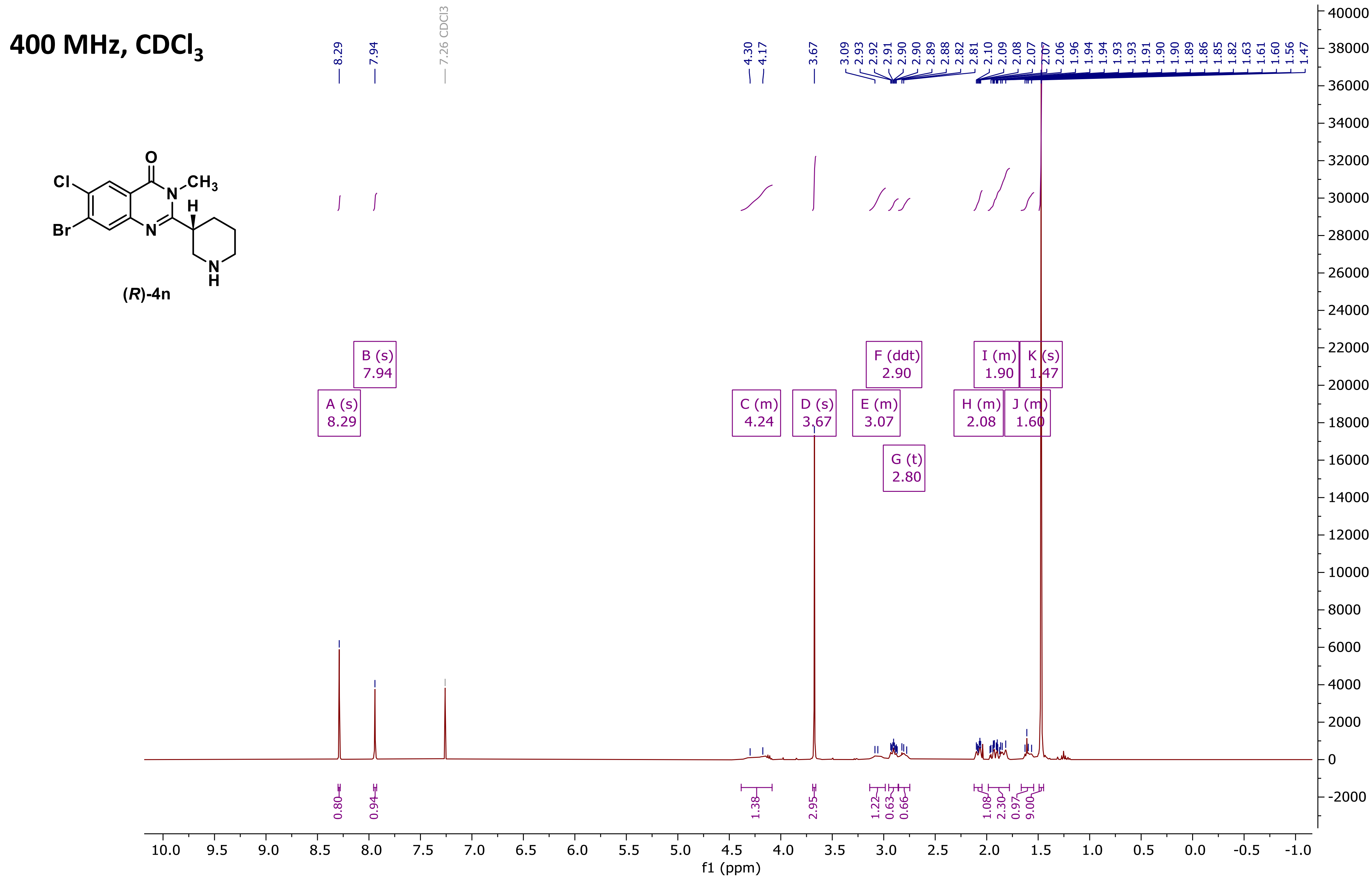
400 MHz, CDCl3
(R)-4n
8.29
7.94
7.26 CDCl3
4.30
4.17
3.67
A (s) 8.29
B (s) 7.94
C (m) 4.24
D (s) 3.67
E (m) 3.07
F (ddt) 2.90
G (t) 2.80
H (m) 2.08
I (m) 1.90
J (m) 1.60
K (s) 1.47
0.80
0.94
1.38
2.95
1.22
0.63
0.66
1.08
2.30
0.97
9.00
f1 (ppm)

101 MHz, $CDCl_3$

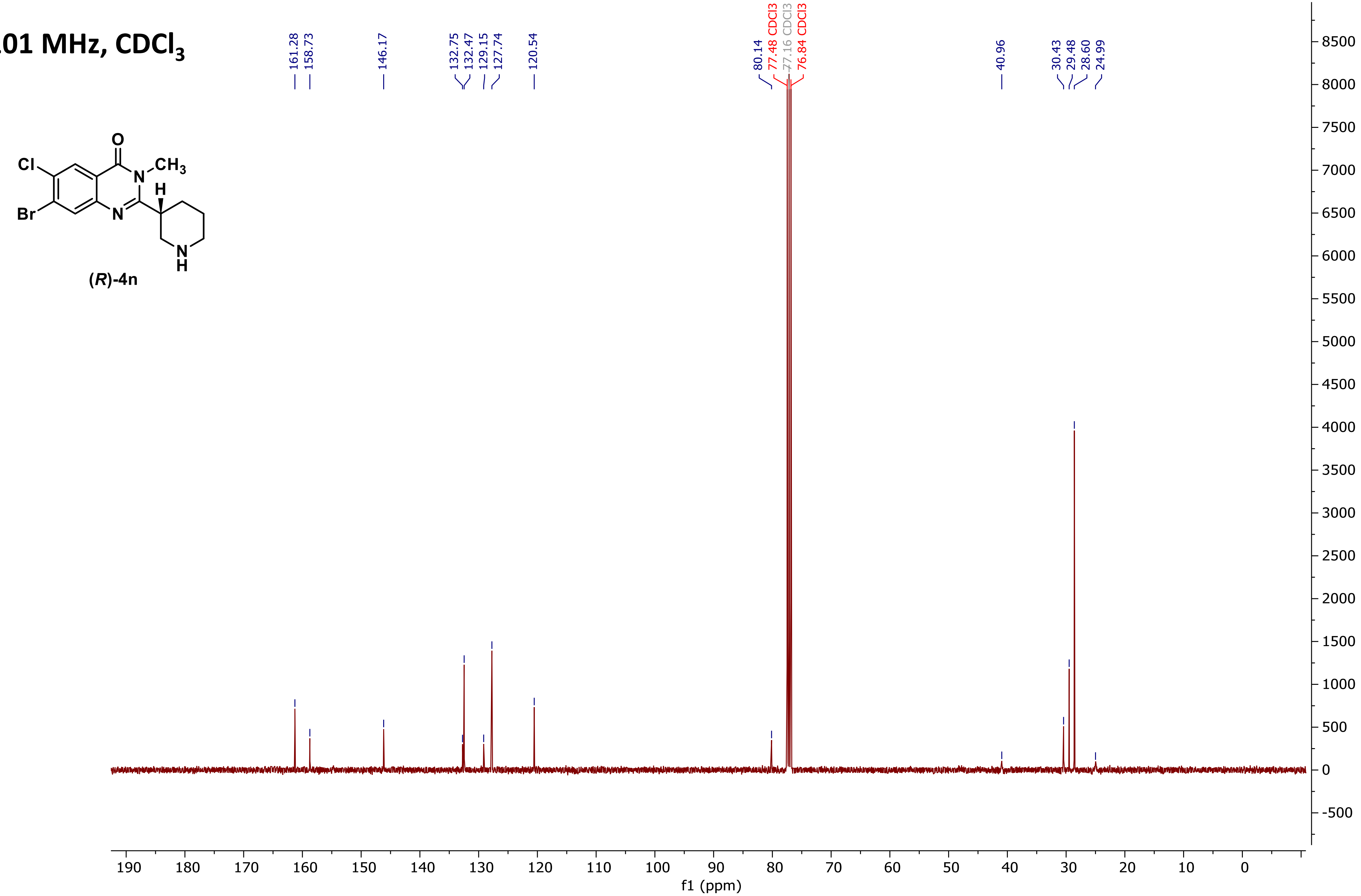

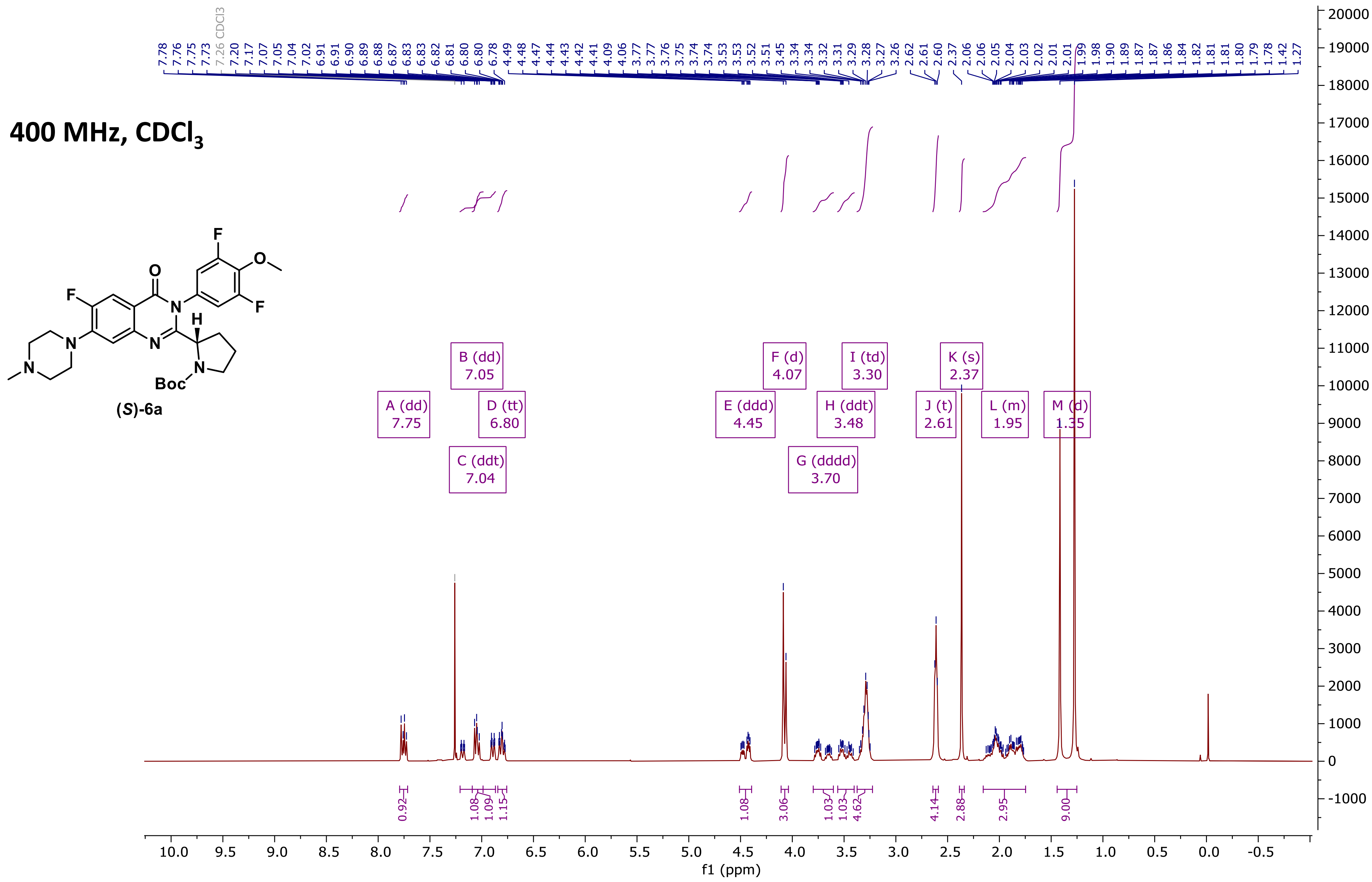

400 MHz, CDCl3
(S)-6a
A (dd) 7.75
B (dd) 7.05
C (ddt) 7.04
D (tt) 6.80
E (ddd) 4.45
F (d) 4.07
G (dddd) 3.70
H (ddt) 3.48
I (td) 3.30
J (t) 2.61
K (s) 2.37
L (m) 1.95
M (d) 1.35
f1 (ppm)

101 MHz, $CDCl_3$

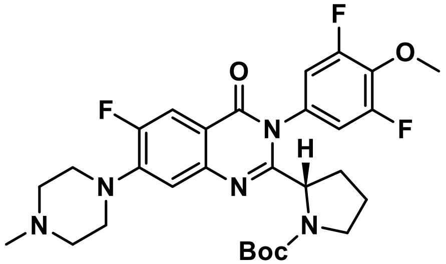

(*S*)-6a

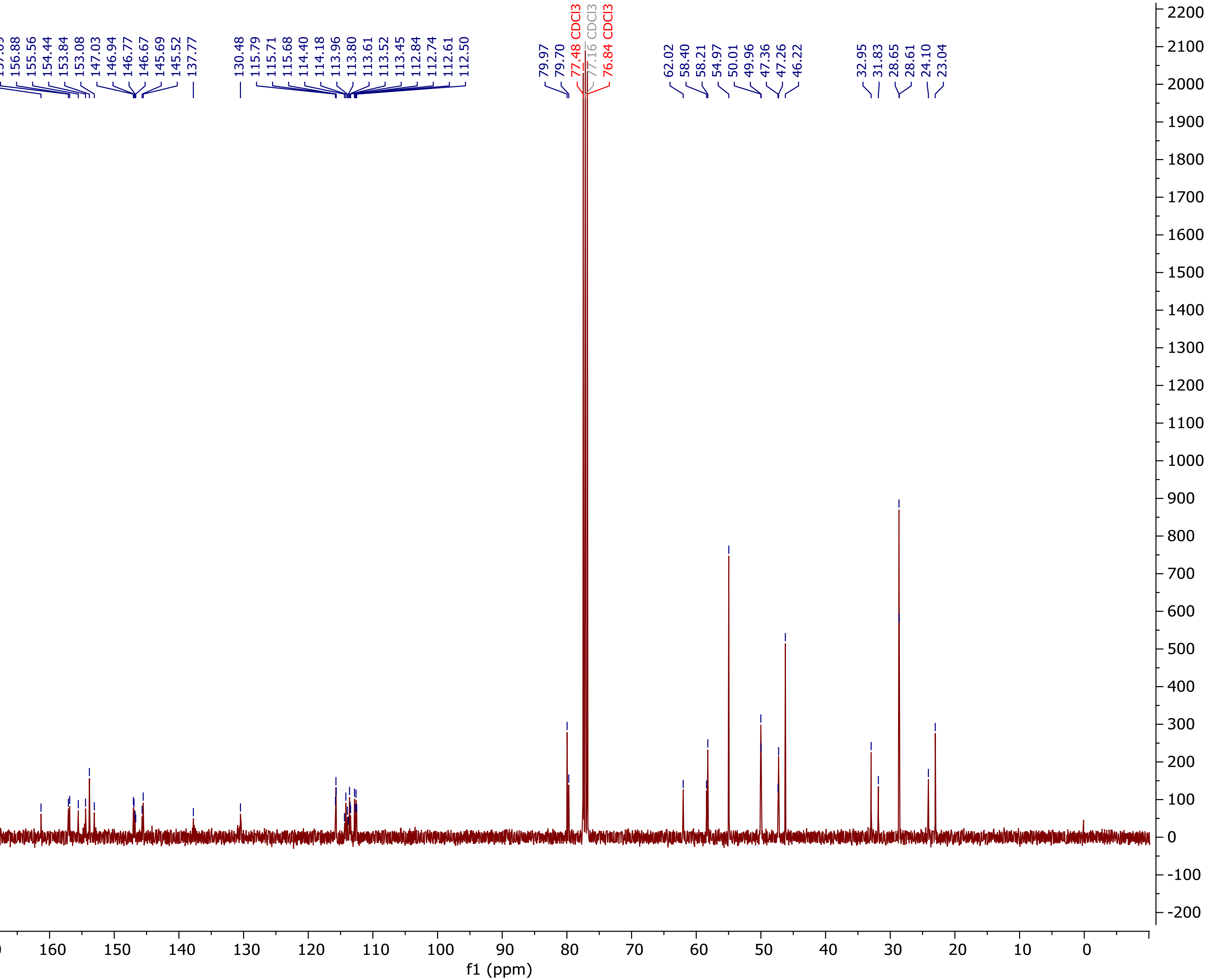

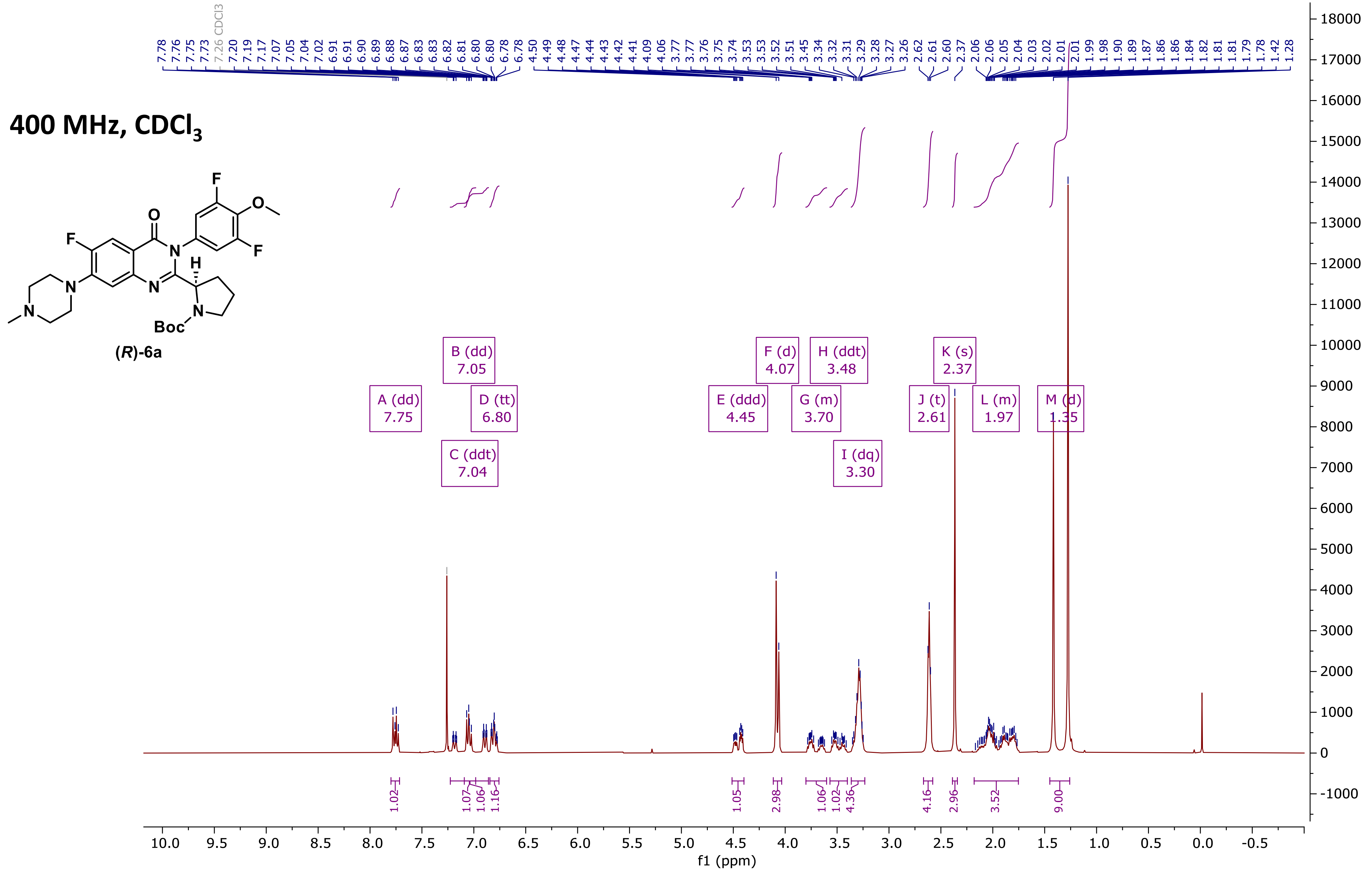
400 MHz, CDCl3
(R)-6a
A (dd) 7.75
B (dd) 7.05
C (ddt) 7.04
D (tt) 6.80
E (ddd) 4.45
F (d) 4.07
G (m) 3.70
H (ddt) 3.48
I (dq) 3.30
J (t) 2.61
K (s) 2.37
L (m) 1.97
M (d) 1.35
f1 (ppm)

101 MHz, $CDCl_3$

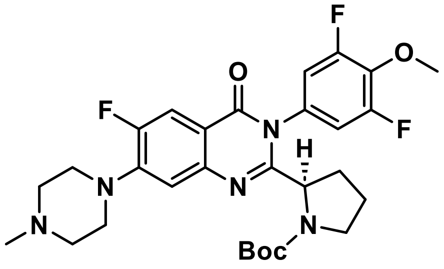

(*R*)-6a

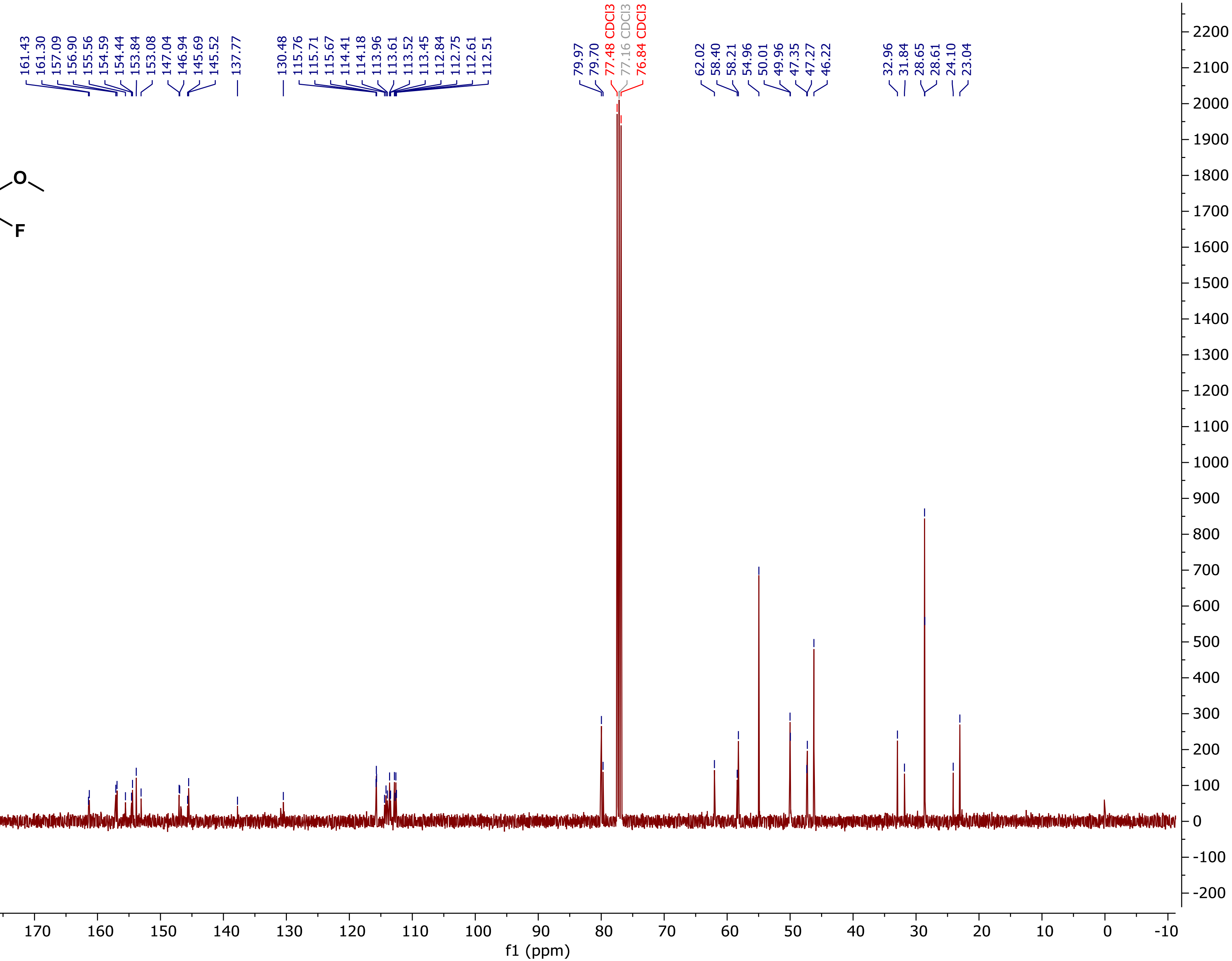

400 MHz, $CDCl_3$

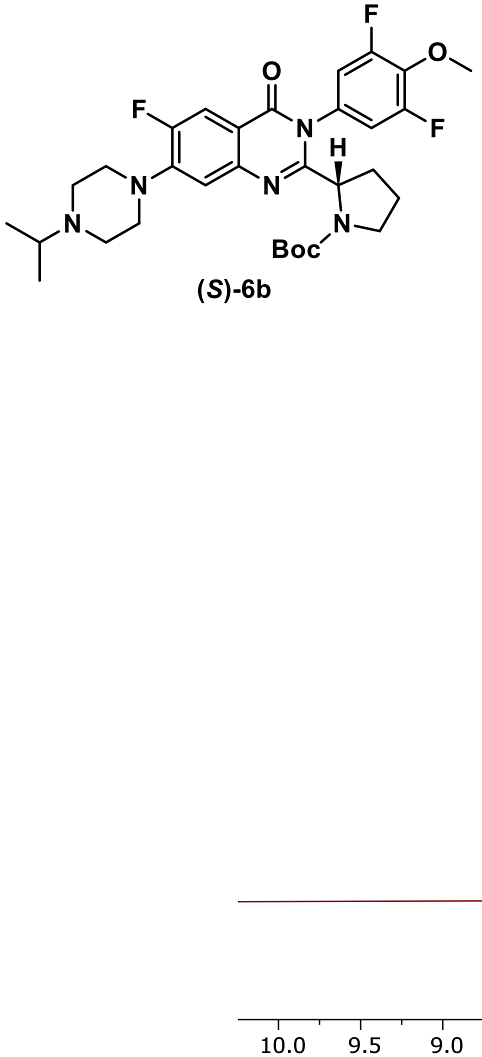

(*S*)-6b

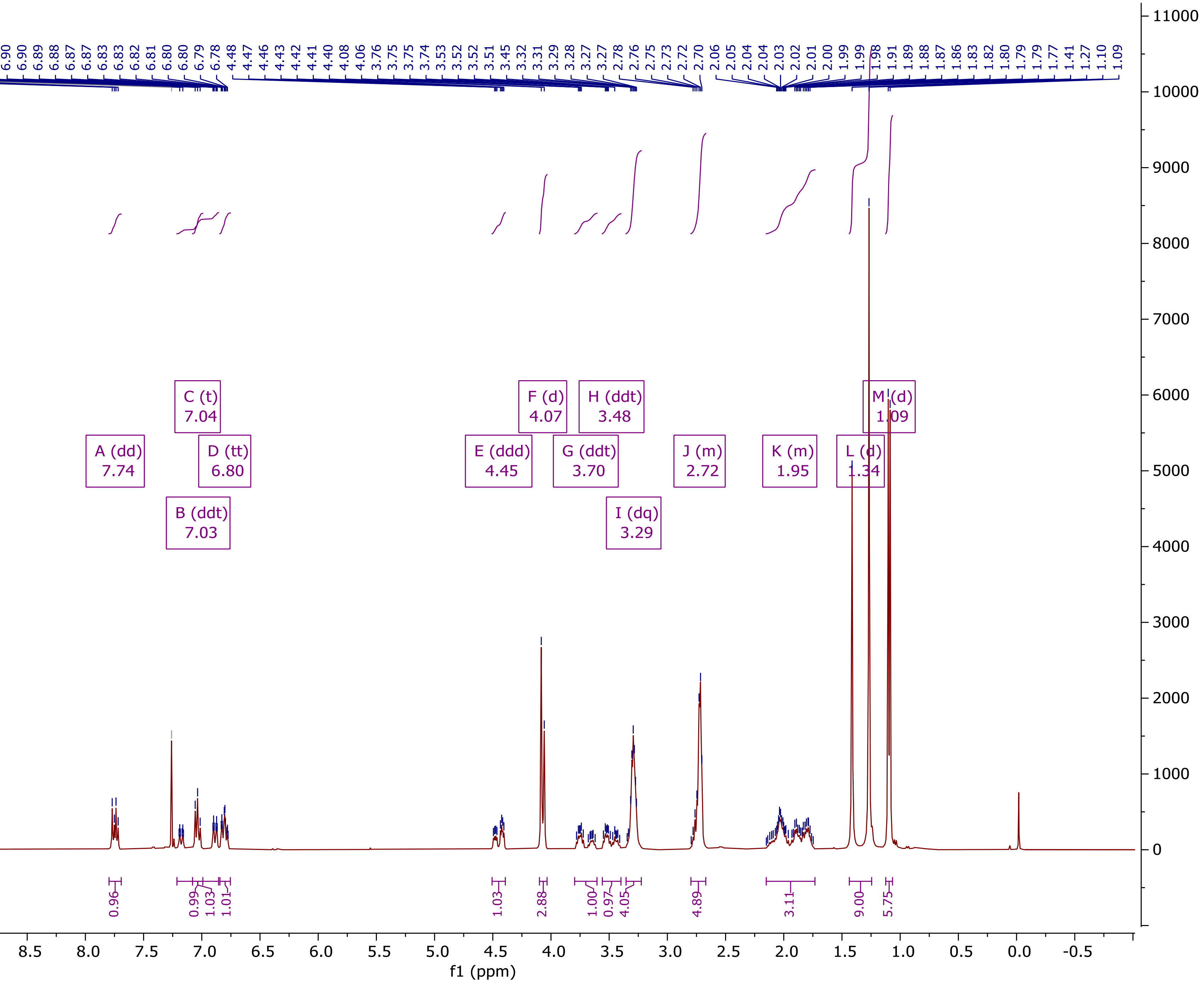

101 MHz, $CDCl_3$

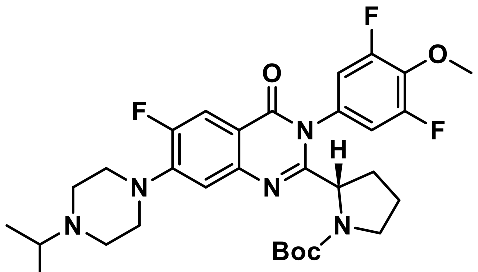

(*S*)-6b

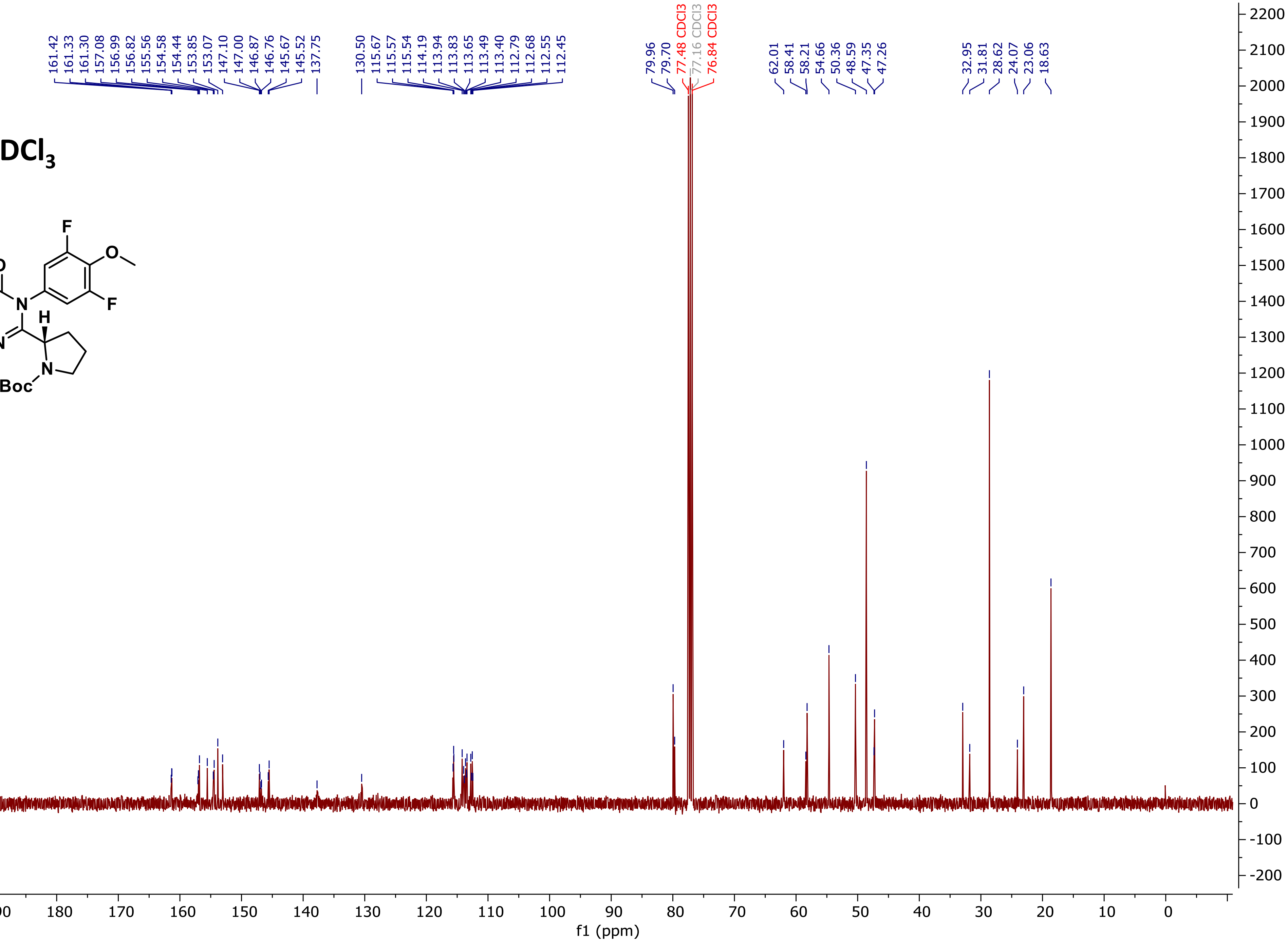

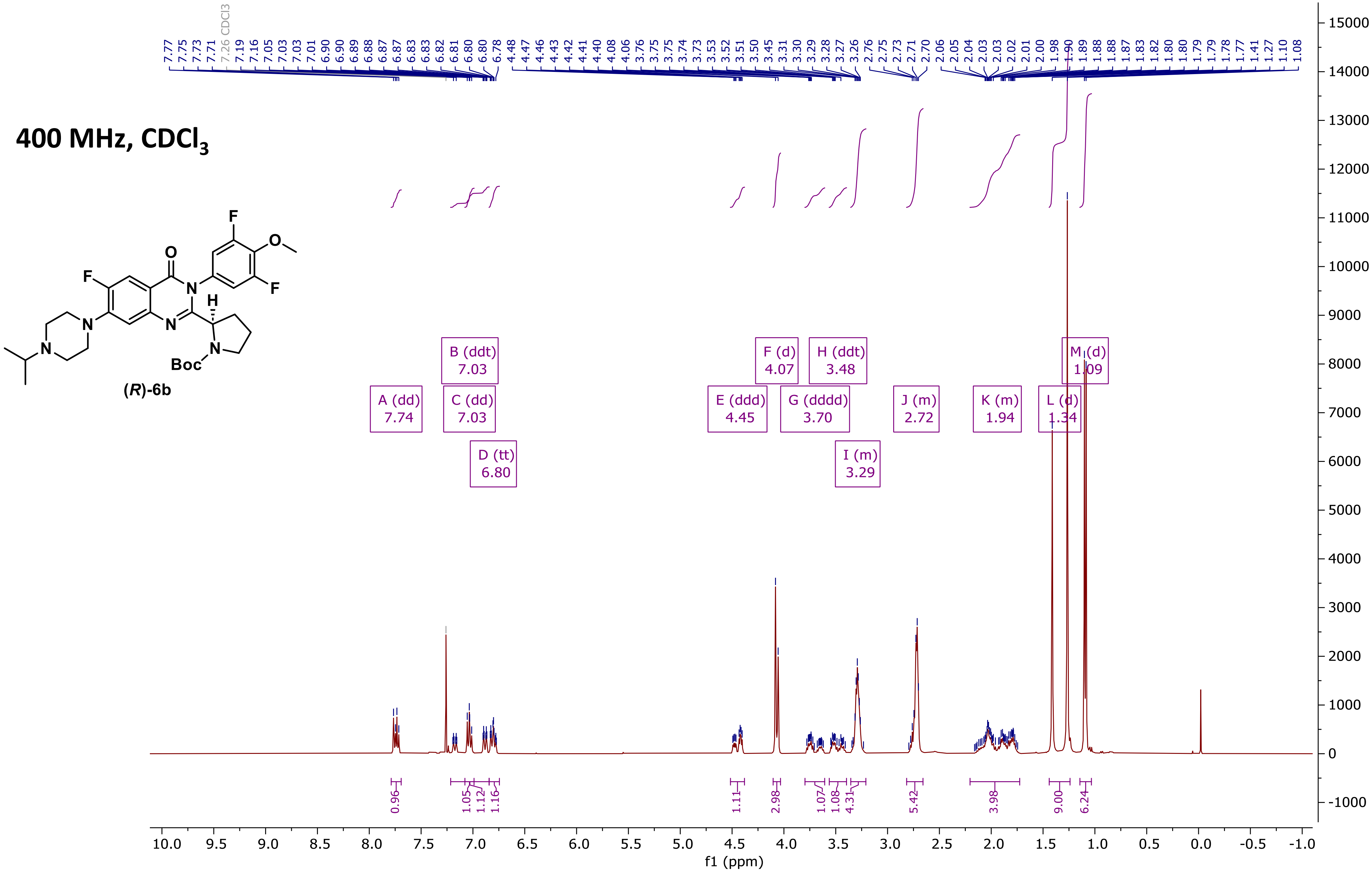
400 MHz, CDCl3
(R)-6b
A (dd) 7.74
B (ddt) 7.03
C (dd) 7.03
D (tt) 6.80
E (ddd) 4.45
F (d) 4.07
G (dddd) 3.70
H (ddt) 3.48
I (m) 3.29
J (m) 2.72
K (m) 1.94
L (d) 1.34
M (d) 1.09
f1 (ppm)

101 MHz, $CDCl_3$

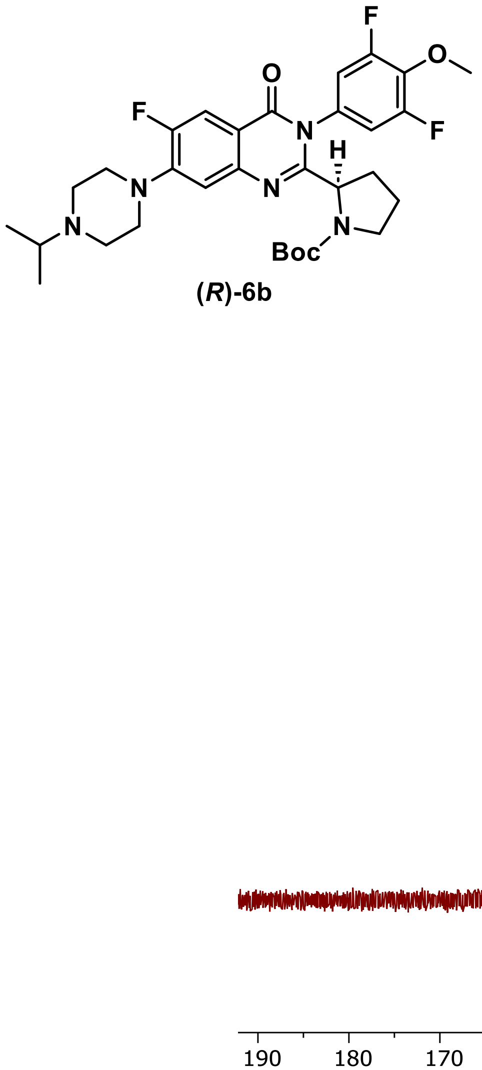

(*R*)-6b

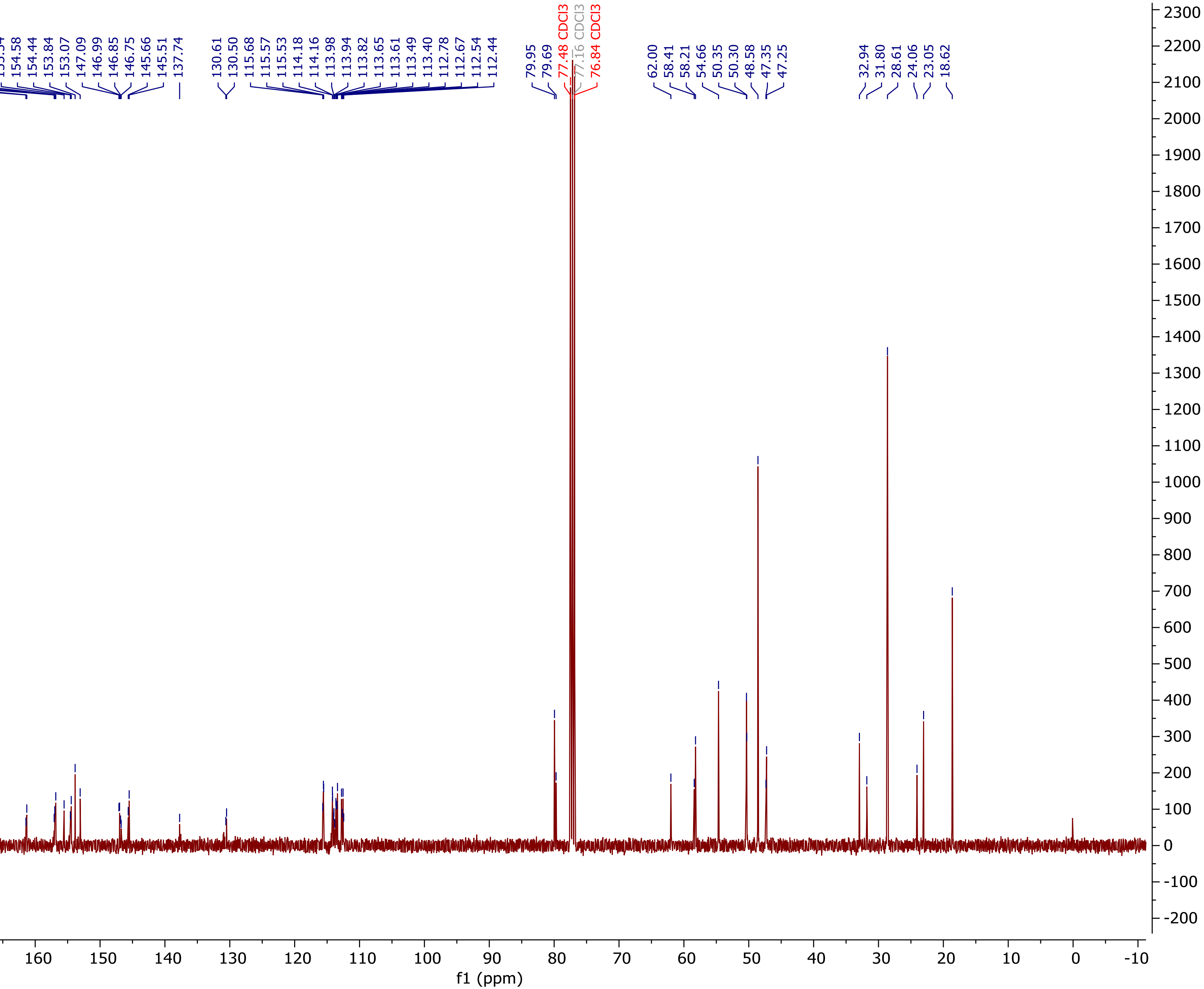

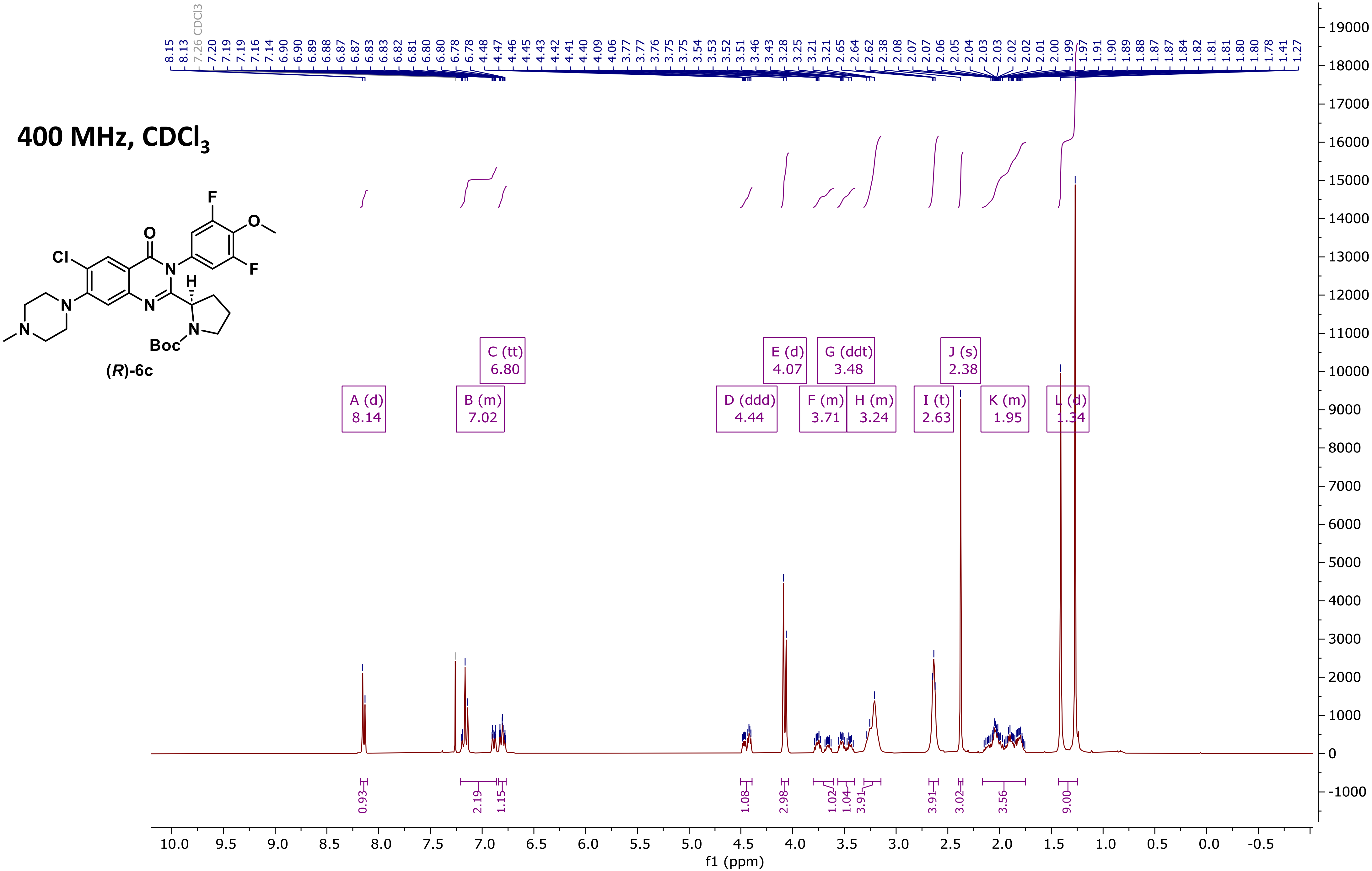
400 MHz, CDCl3
(R)-6c
A (d) 8.14
B (m) 7.02
C (tt) 6.80
D (ddd) 4.44
E (d) 4.07
F (m) 3.71
G (ddt) 3.48
H (m) 3.24
I (t) 2.63
J (s) 2.38
K (m) 1.95
L (d) 1.34
f1 (ppm)

101 MHz, $CDCl_3$

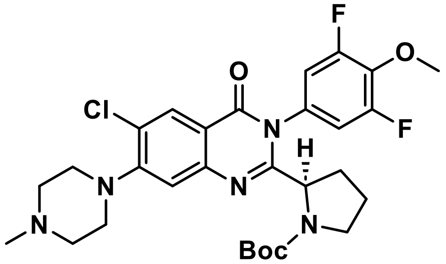

(*R*)-6c

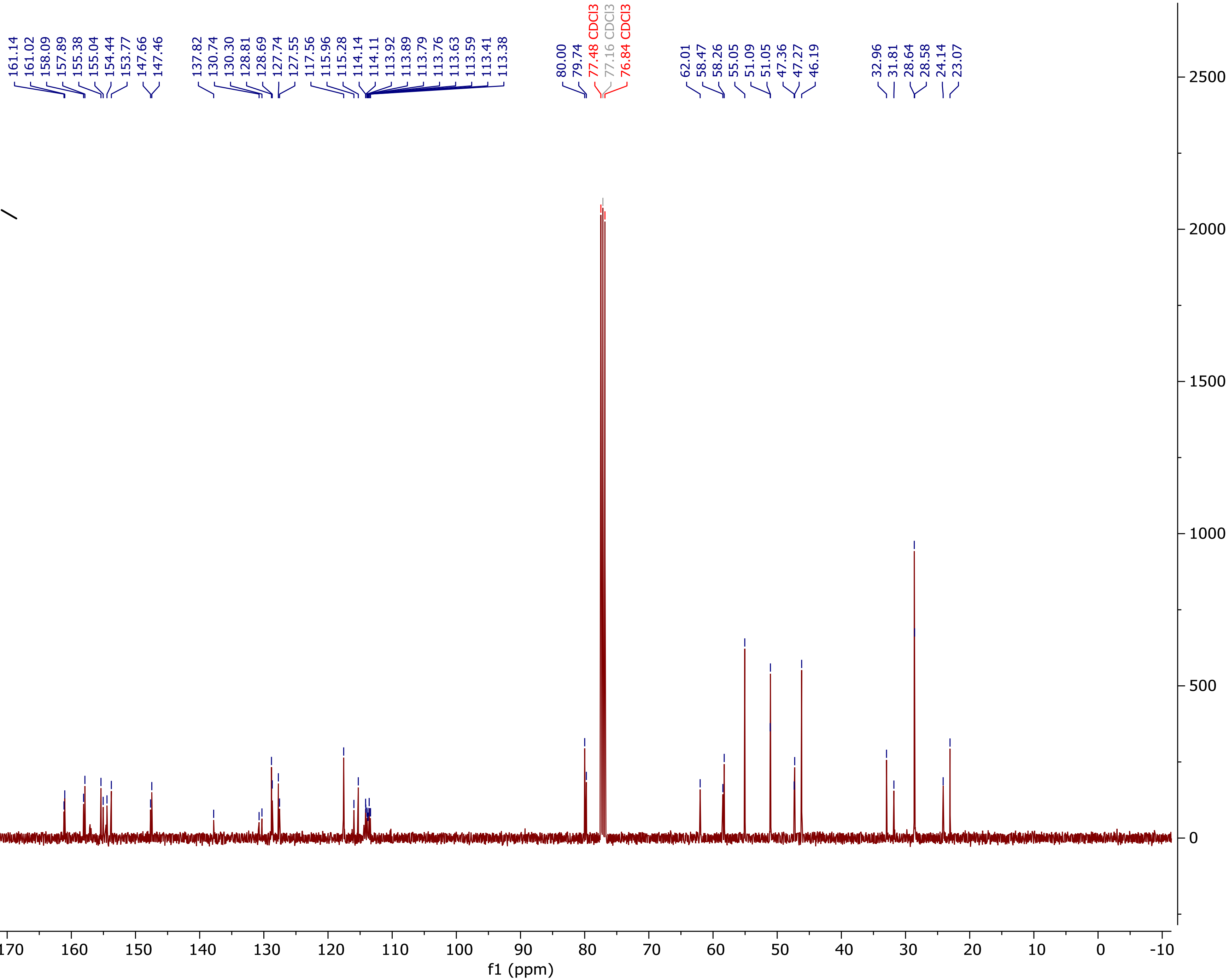

400 MHz, $CDCl_3$

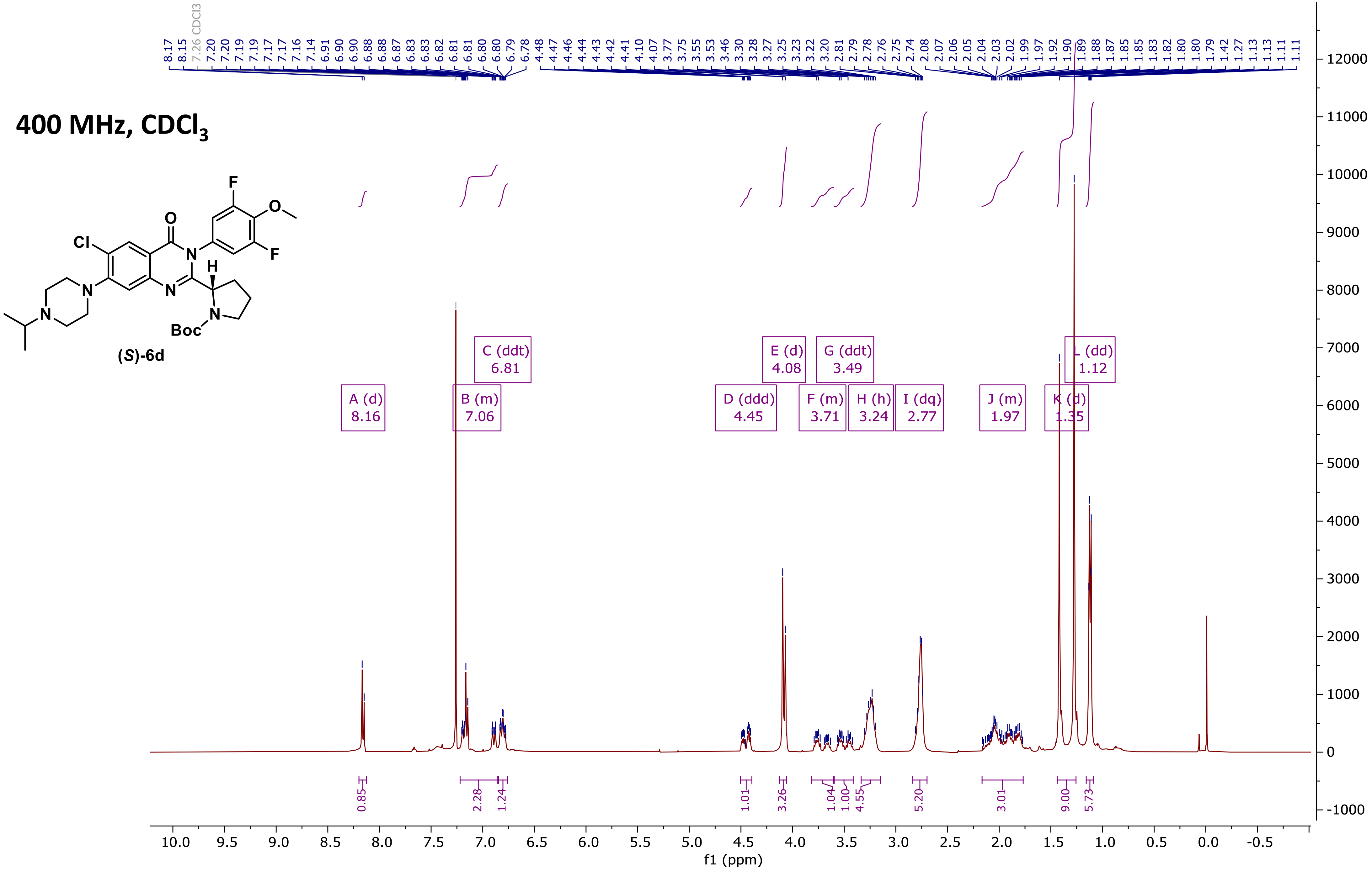

(*S*)-6d

101 MHz, CDCl$_3$

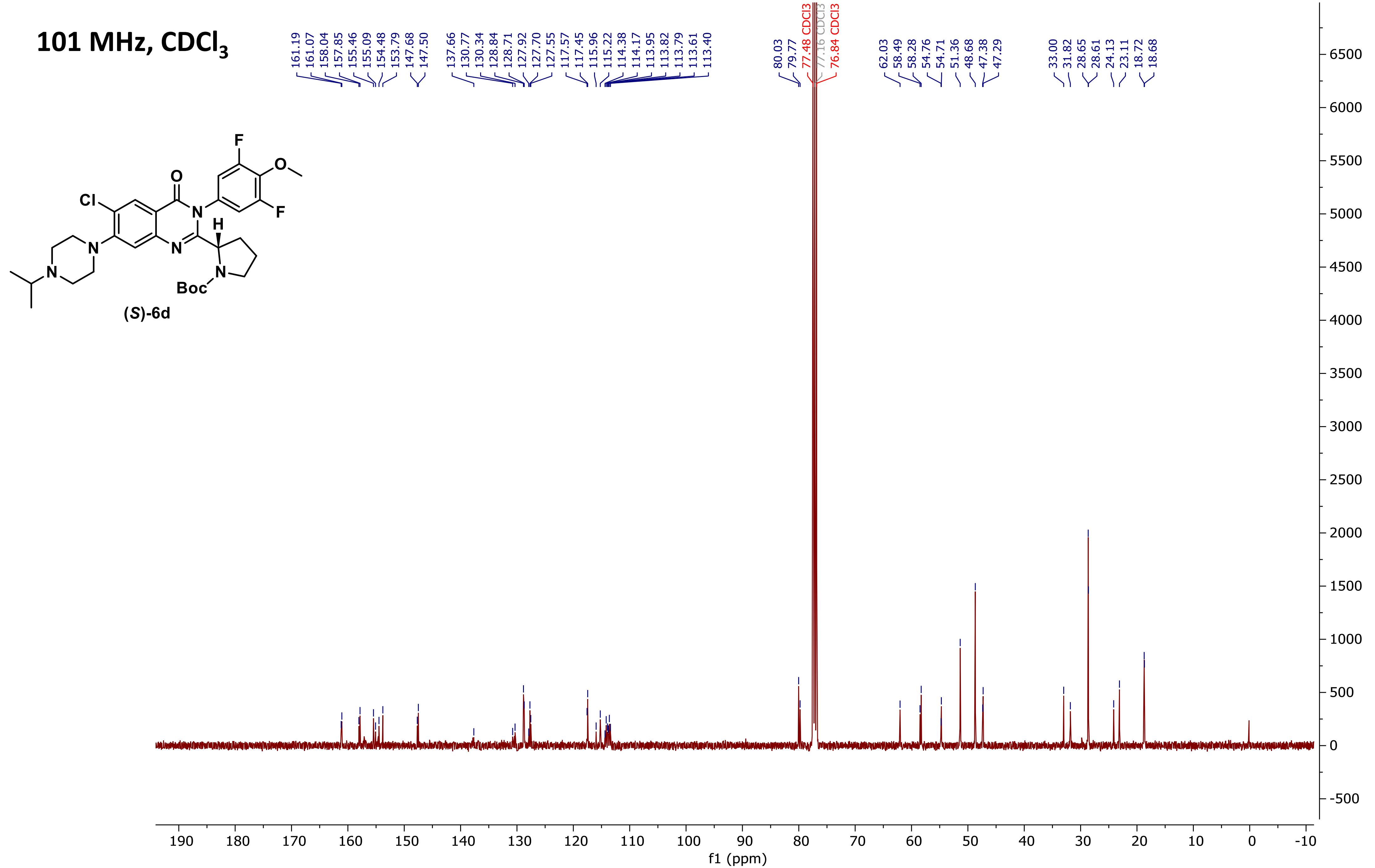

(*S*)-6d

400 MHz, $CDCl_3$

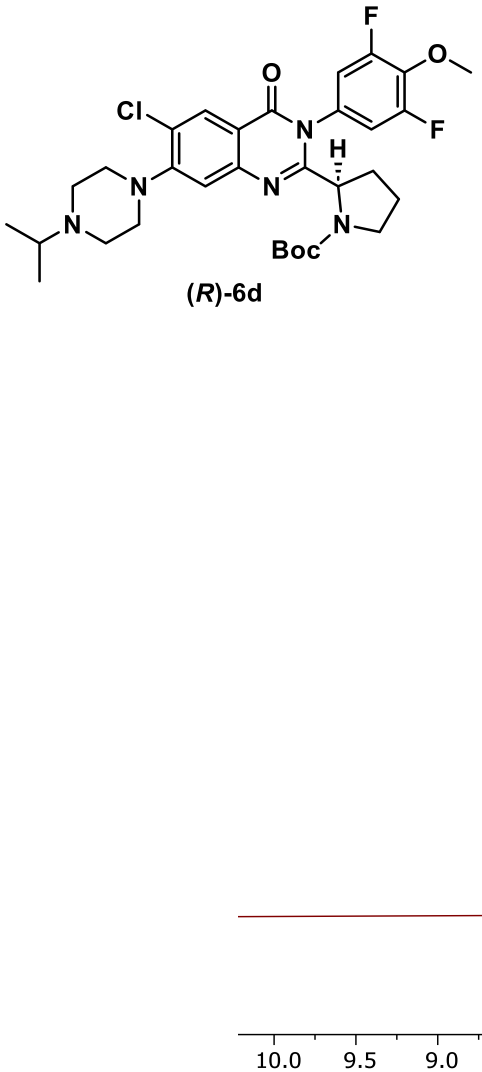

(*R*)-6d

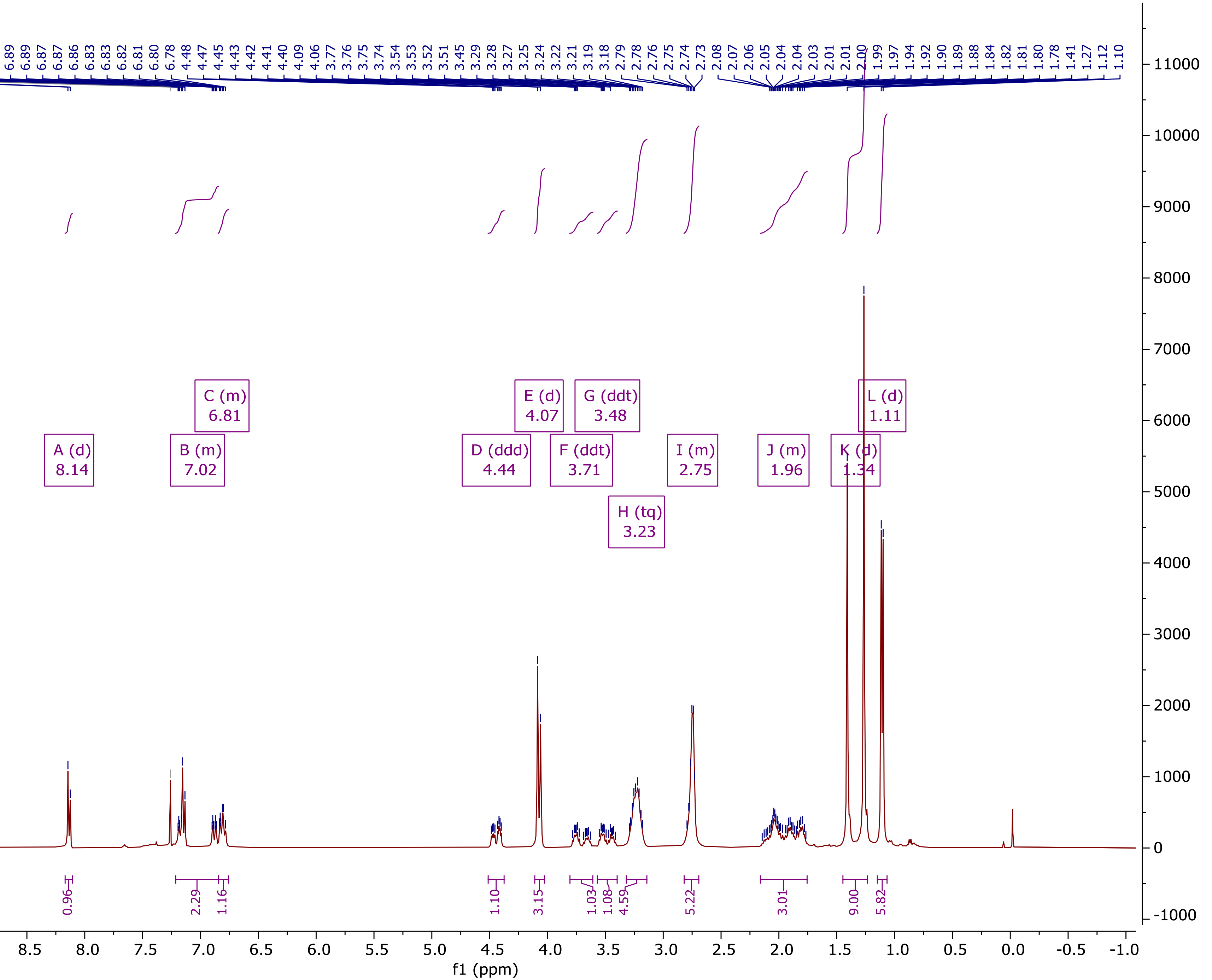

101 MHz, $CDCl_3$

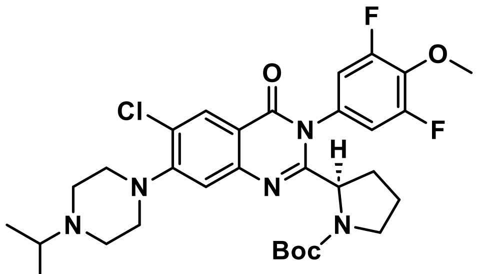

(*R*)-6d

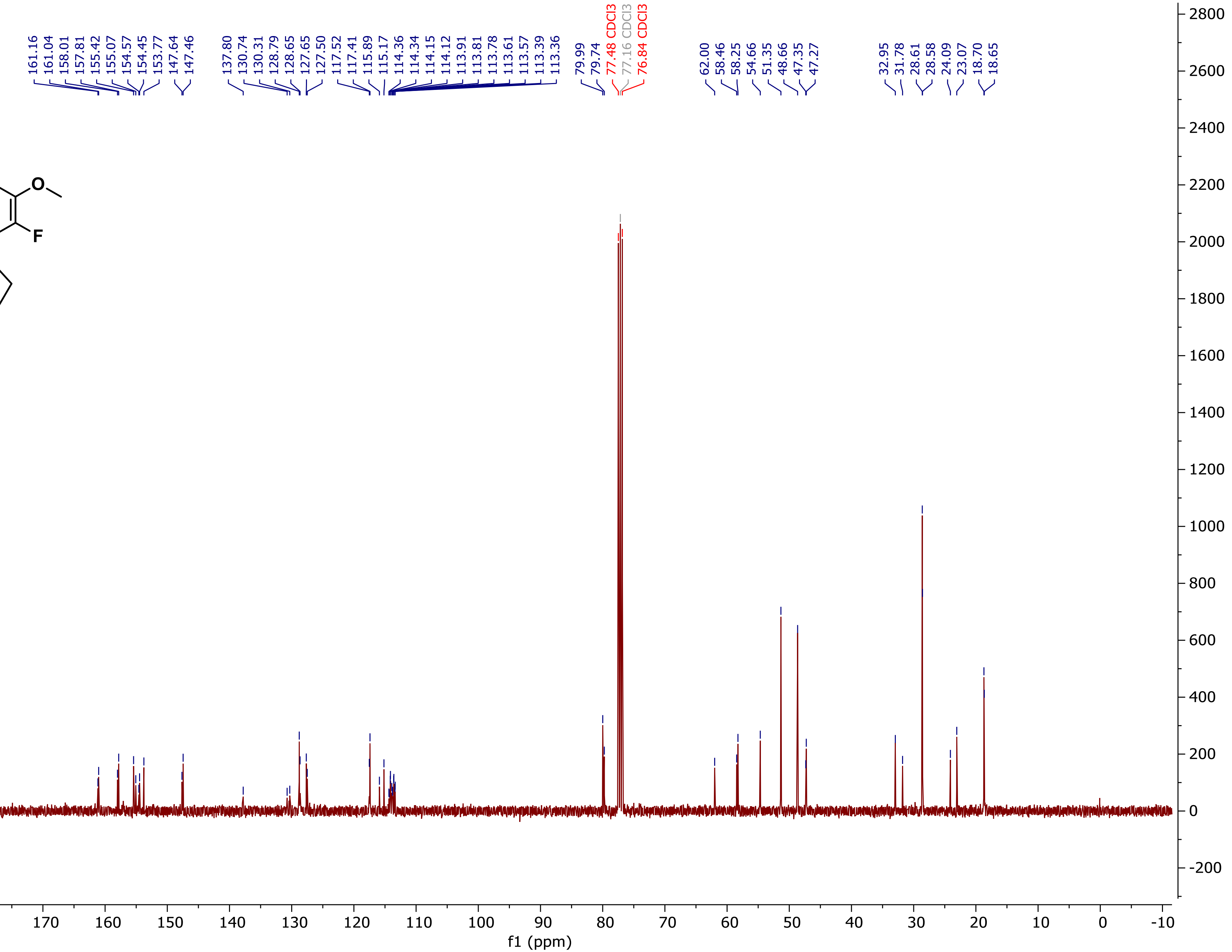

400 MHz, $CDCl_3$

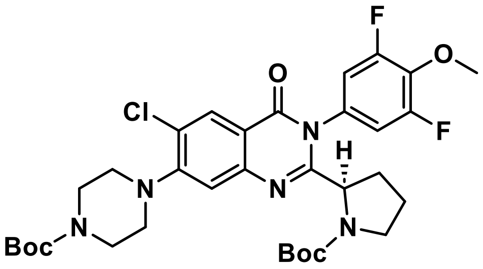

(*R*)-6e

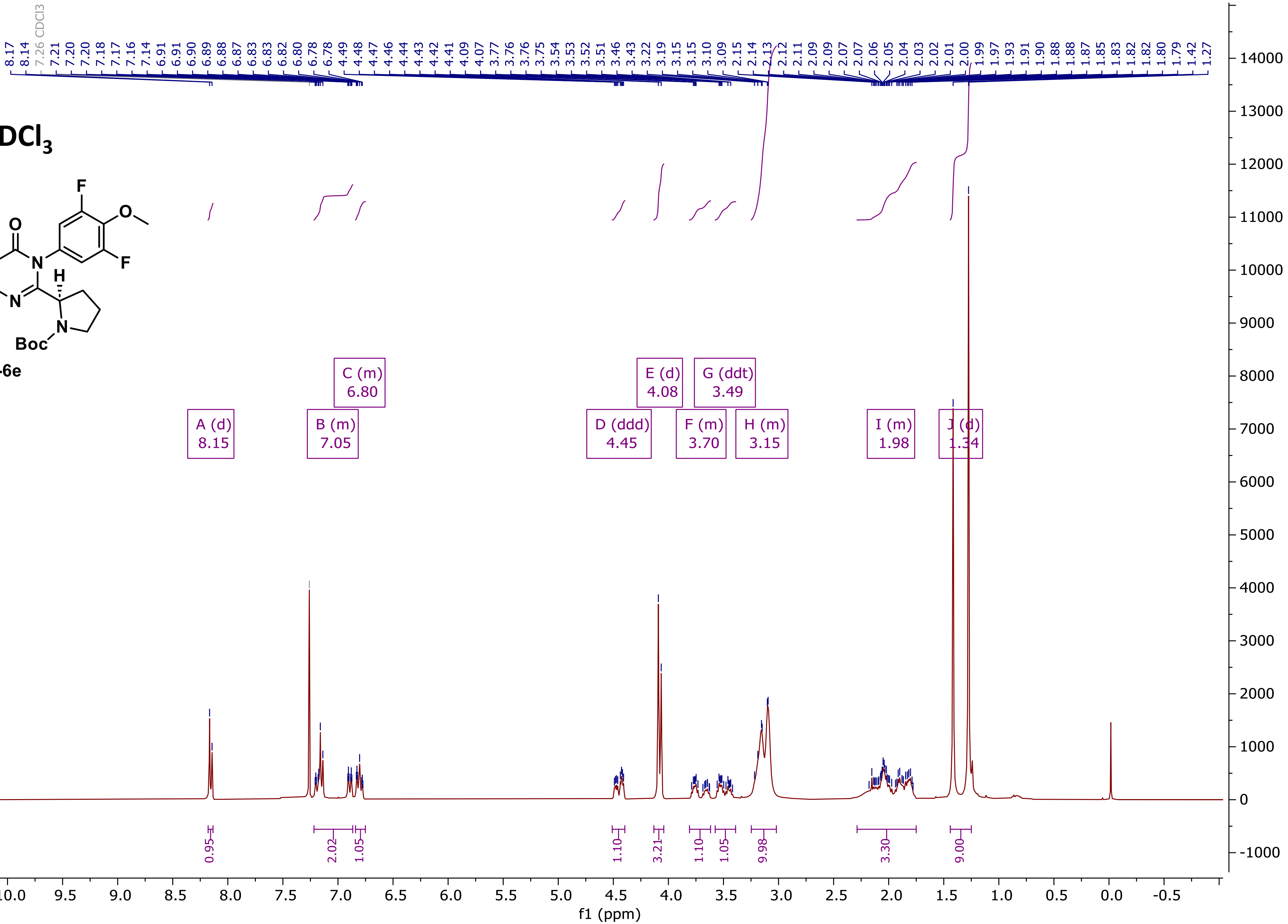

101 MHz, $CDCl_3$

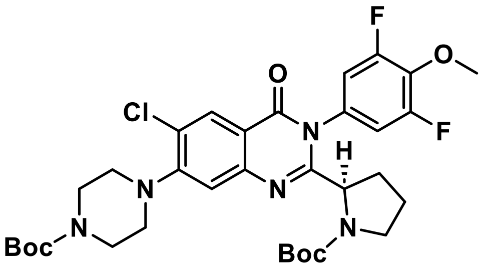

(*R*)-6e

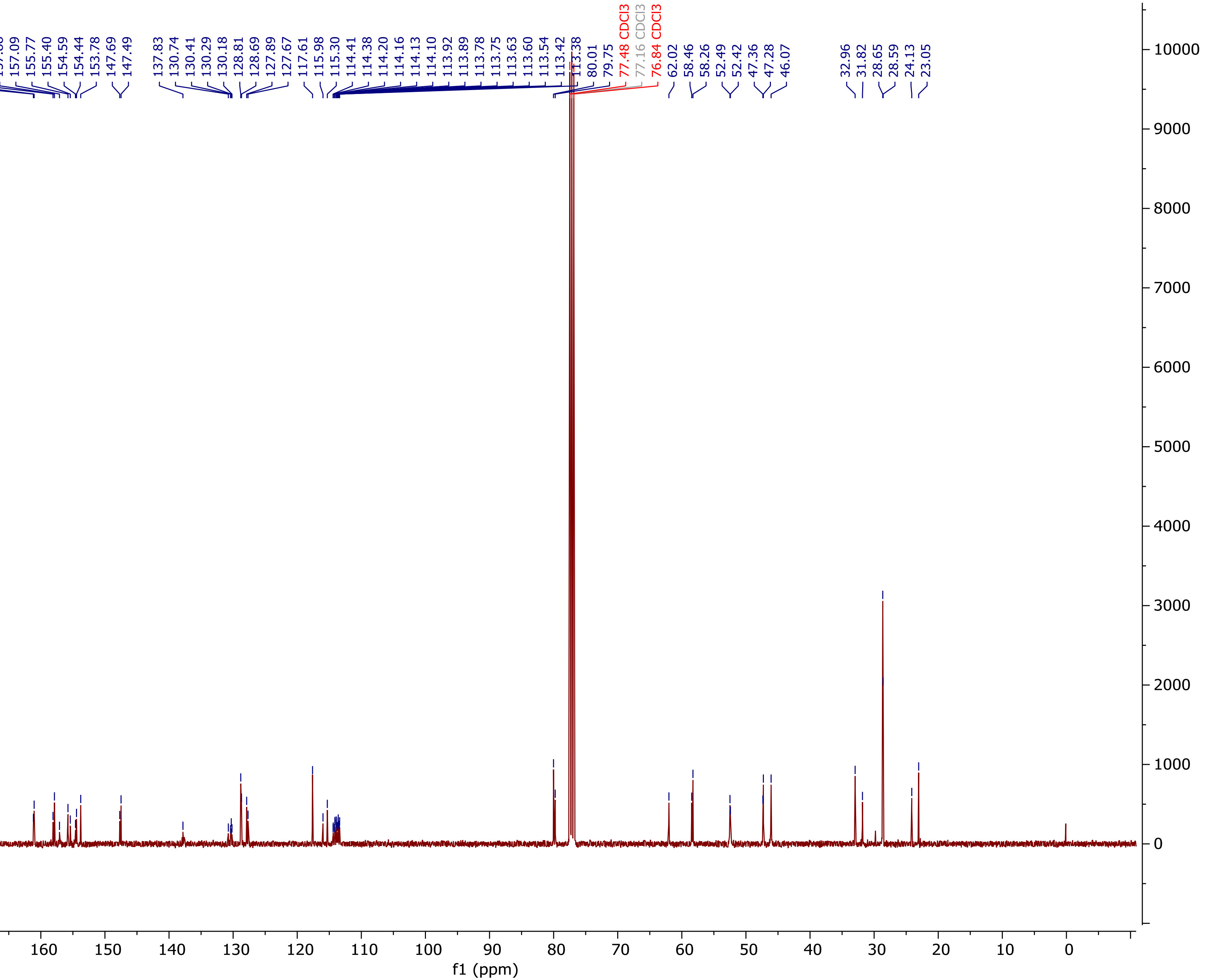

400 MHz, $CDCl_3$

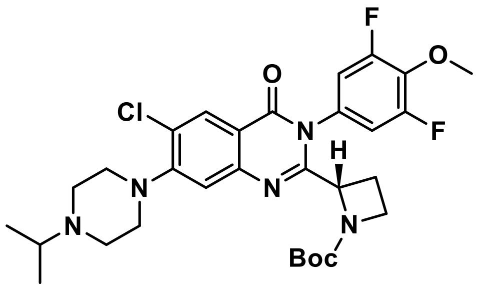

(*S*)-6f

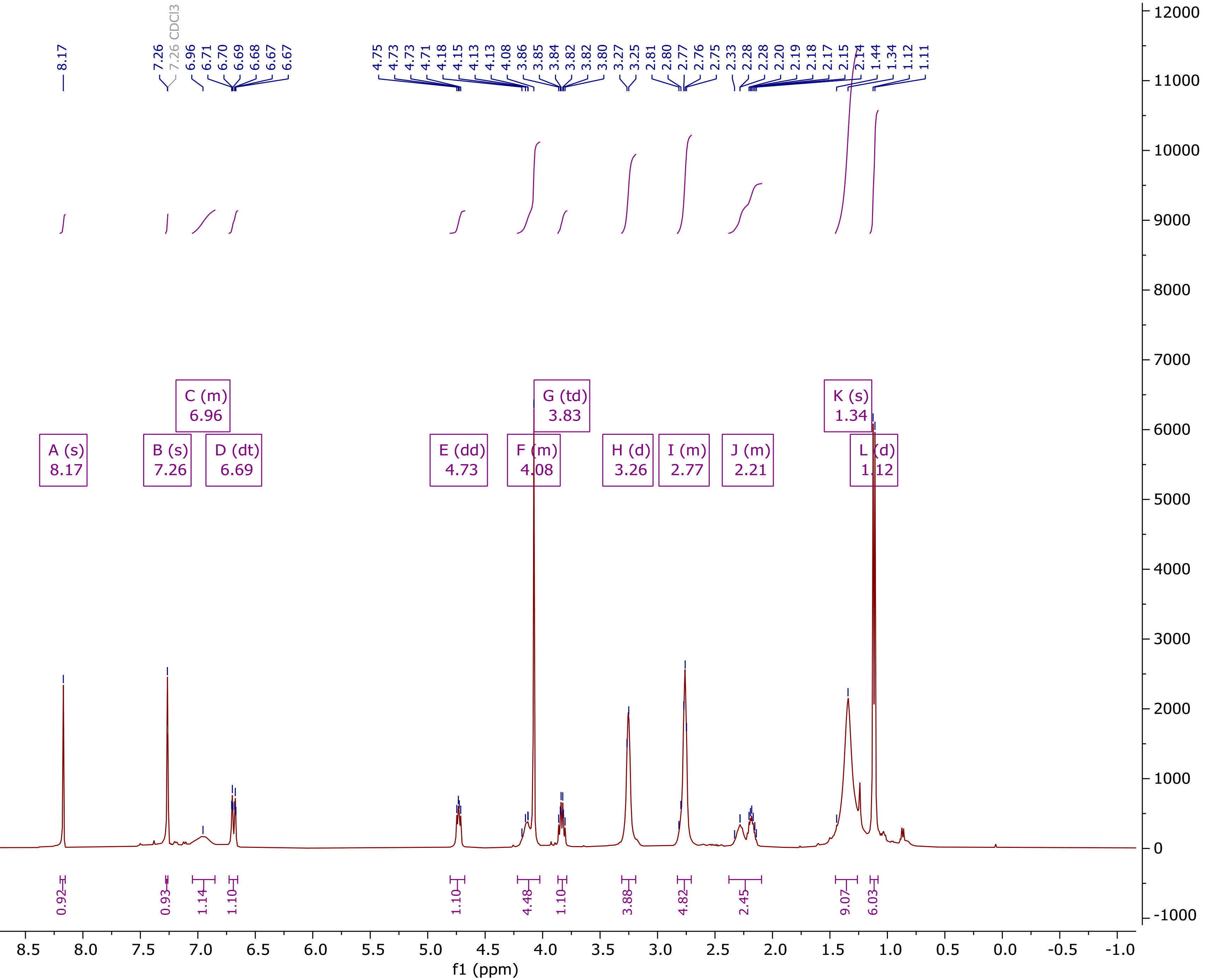

101 MHz, $CDCl_3$

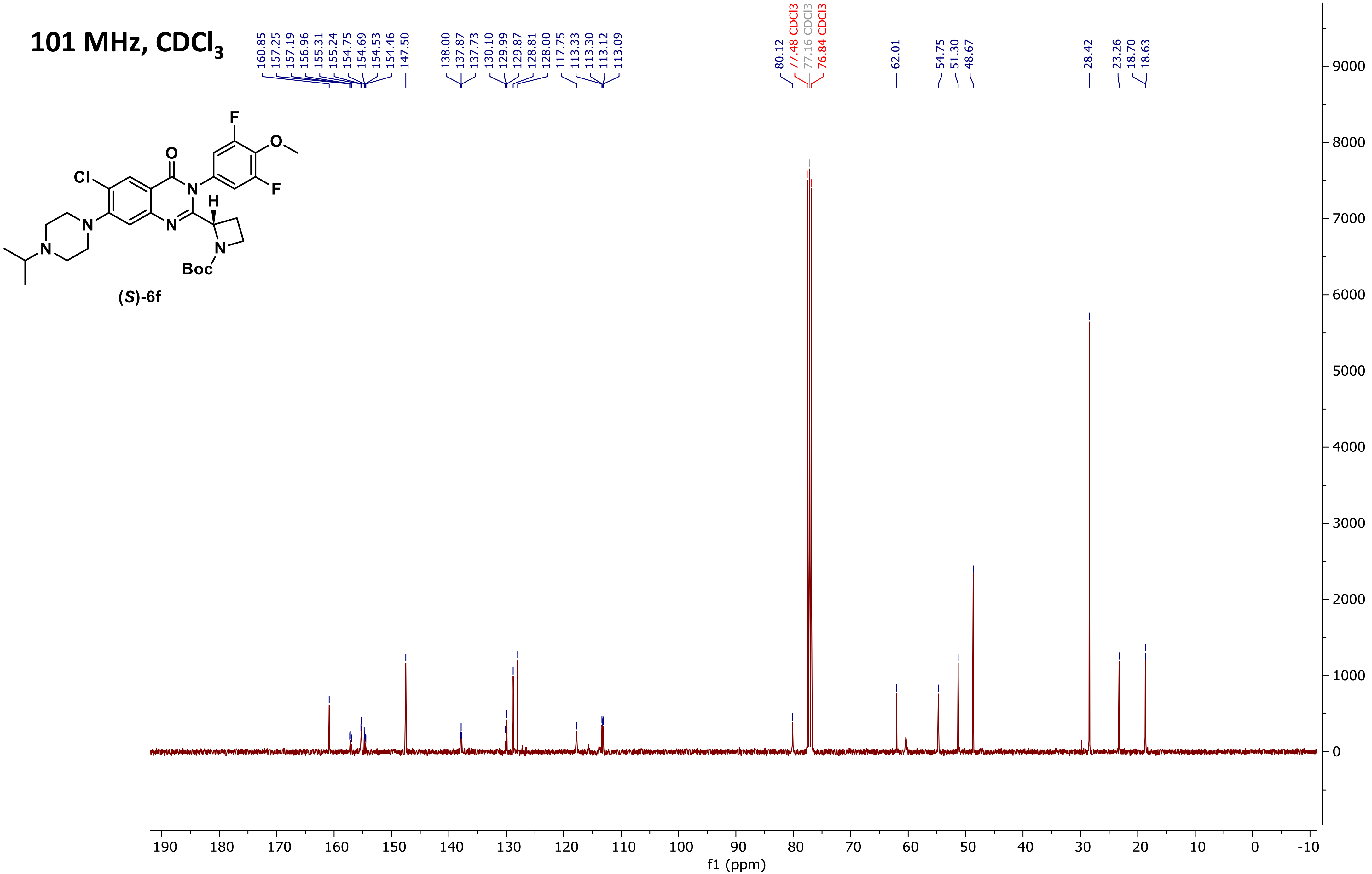

400 MHz, $CDCl_3$

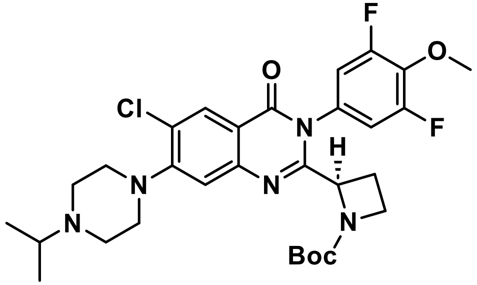

(*R*)-6f

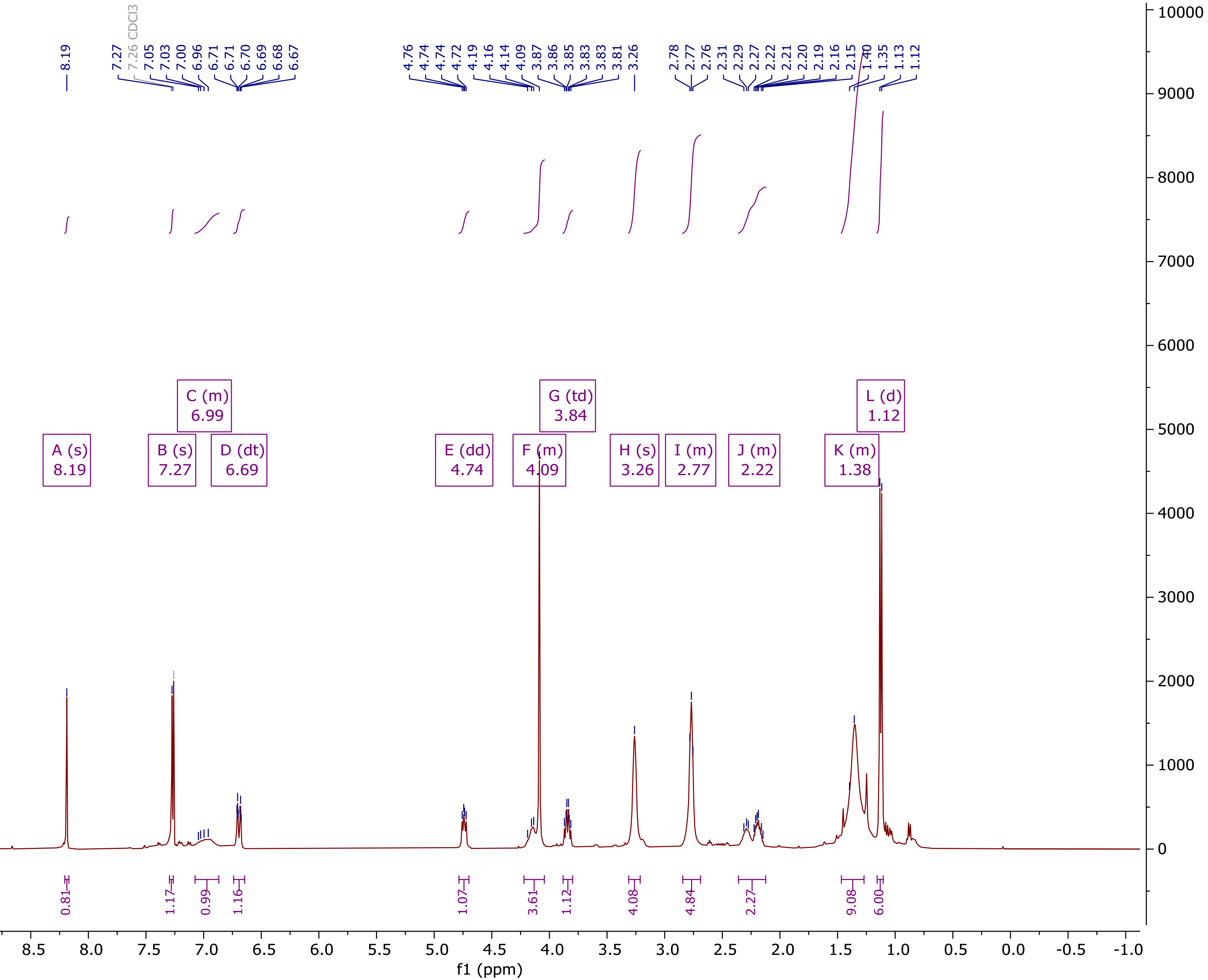

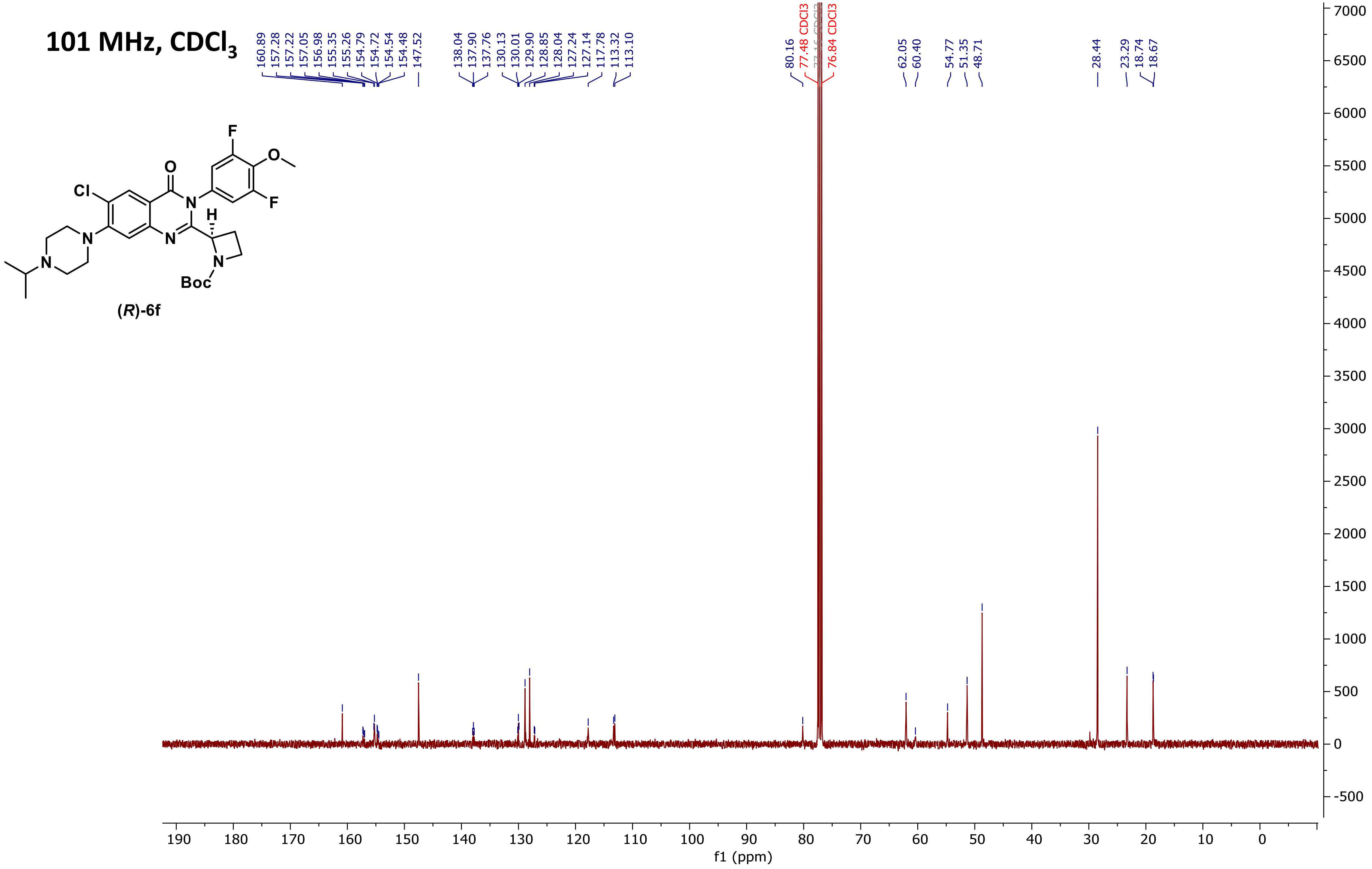
101 MHz, CDCl3
160.89
157.28
157.22
157.05
156.98
155.35
155.26
154.79
154.72
154.54
154.48
147.52
138.04
137.90
137.76
130.13
130.01
129.90
128.85
128.04
127.24
127.14
117.78
113.32
113.10
80.16
77.48 CDCl3
76.84 CDCl3
62.05
60.40
54.77
51.35
48.71
28.44
23.29
18.74
18.67
(R)-6f
f1 (ppm)

400 MHz, CDCl$_3$

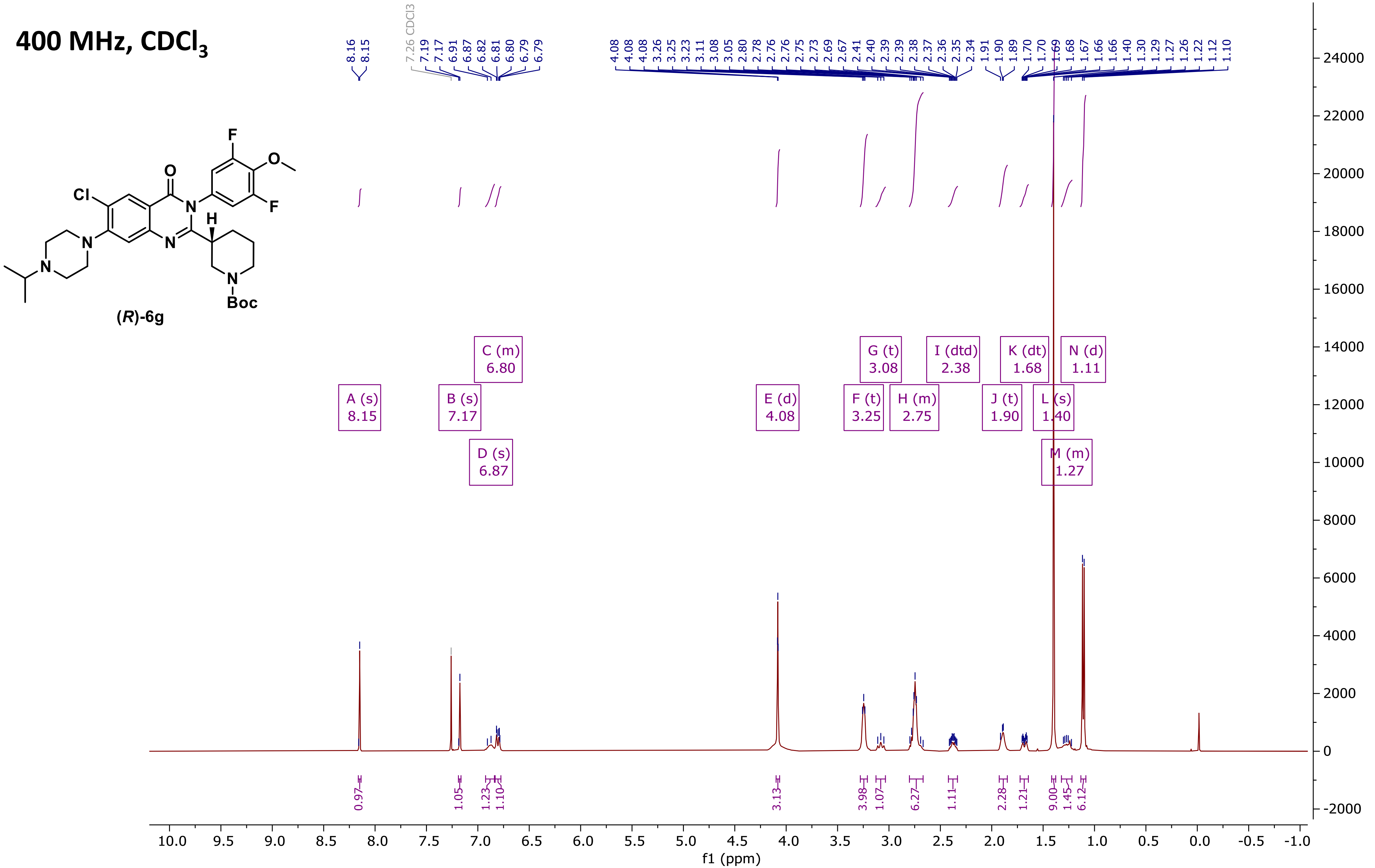

101 MHz, $CDCl_3$

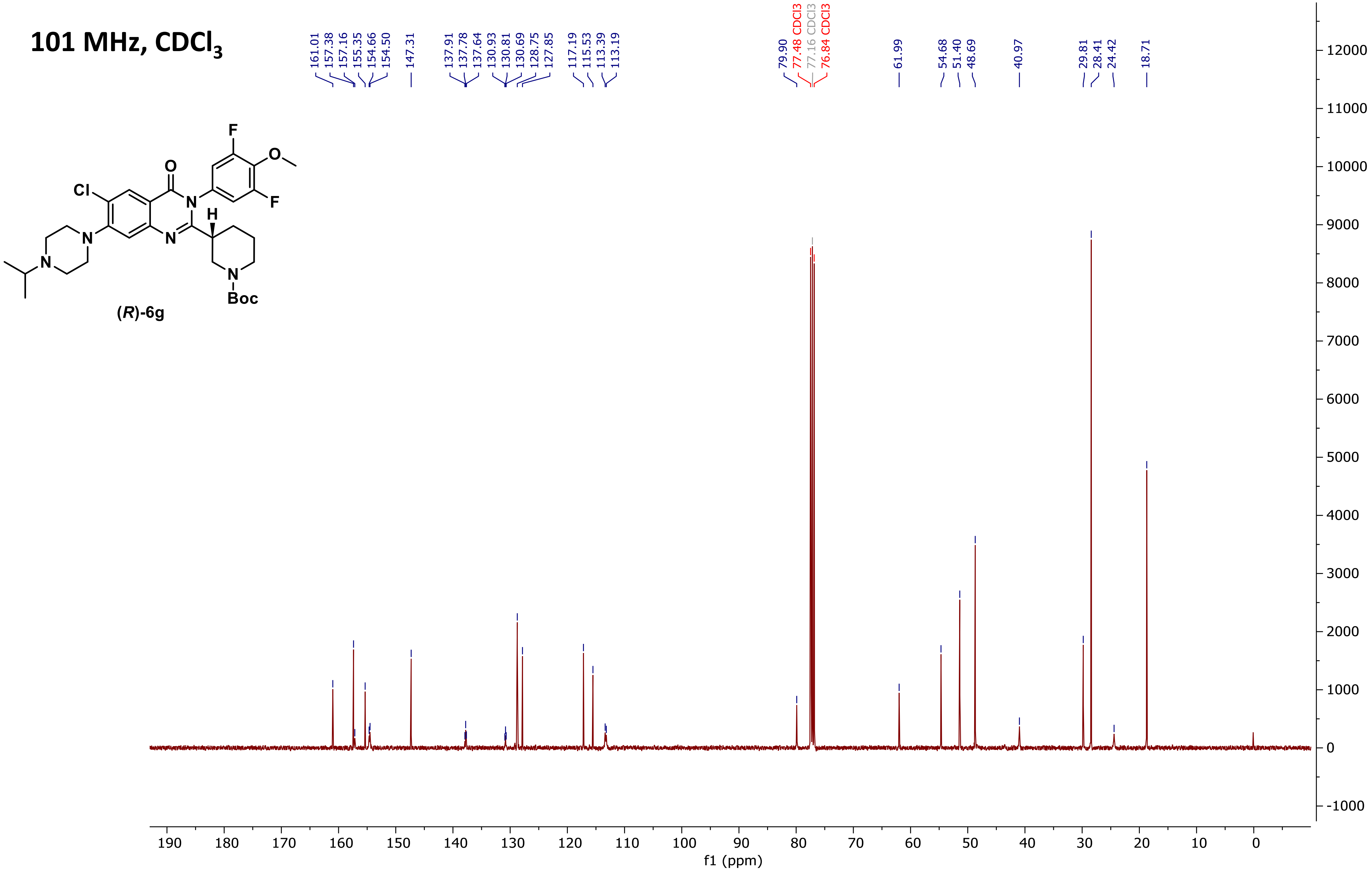

(*R*)-6g

400 MHz, $CDCl_3$

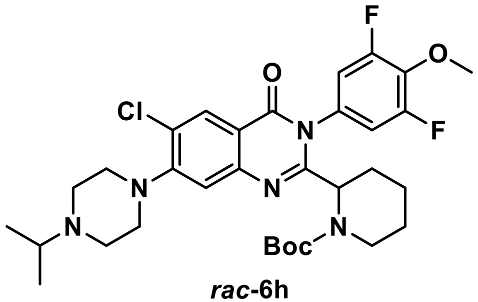

rac-**6h**

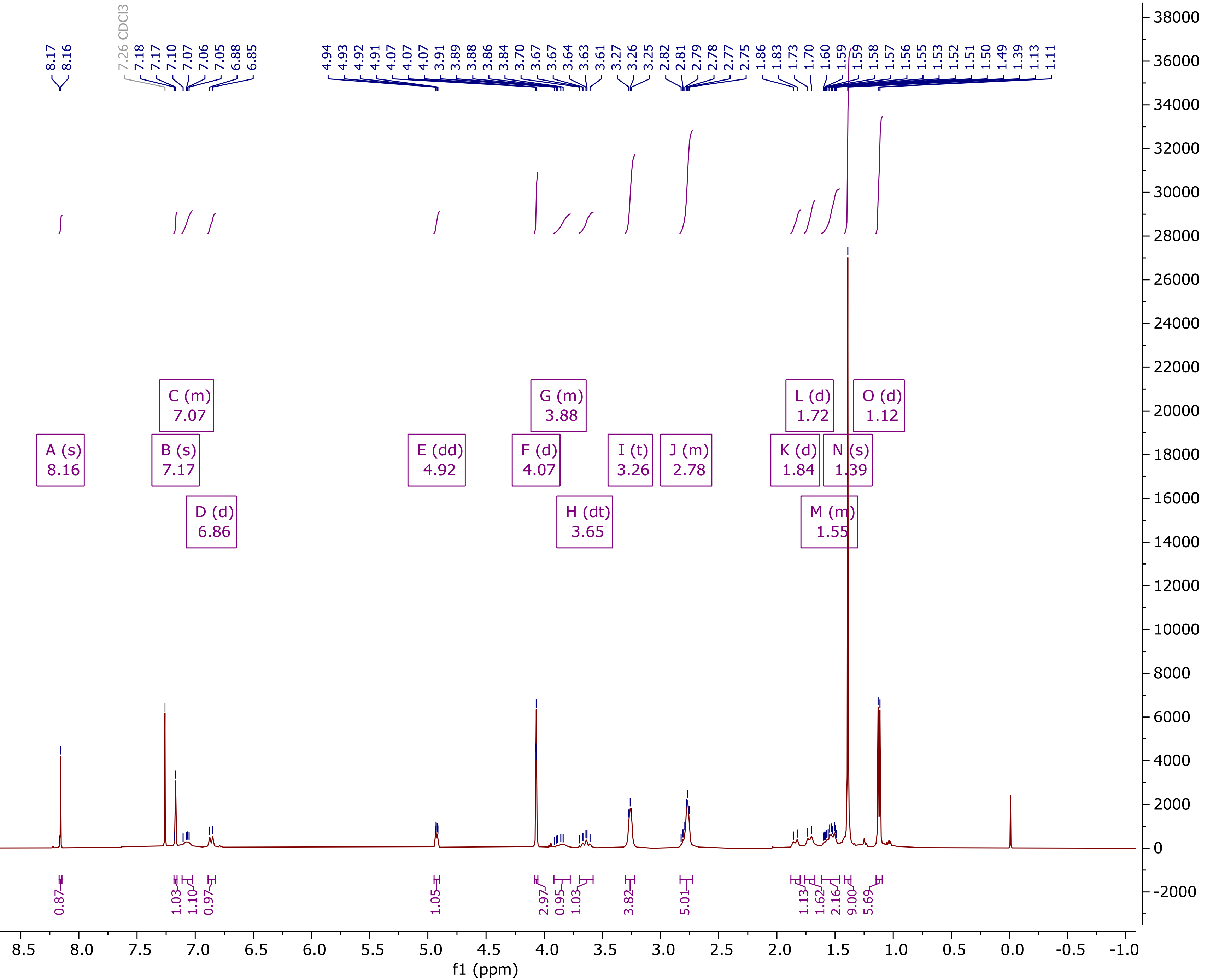

101 MHz, $CDCl_3$

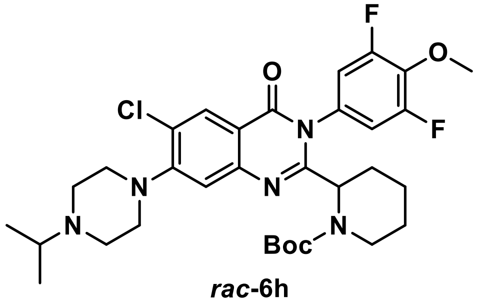

***rac*-6h**

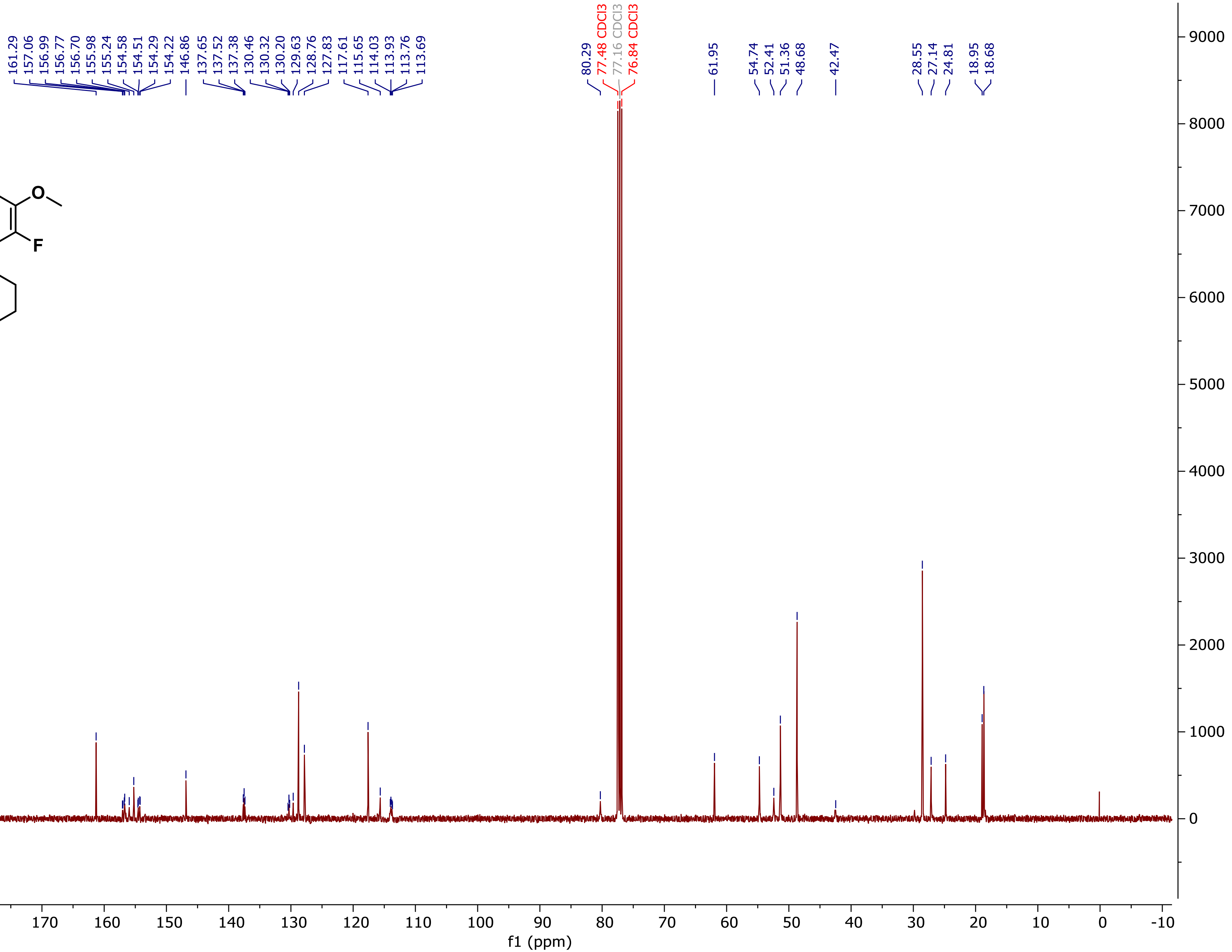

400 MHz, $CDCl_3$

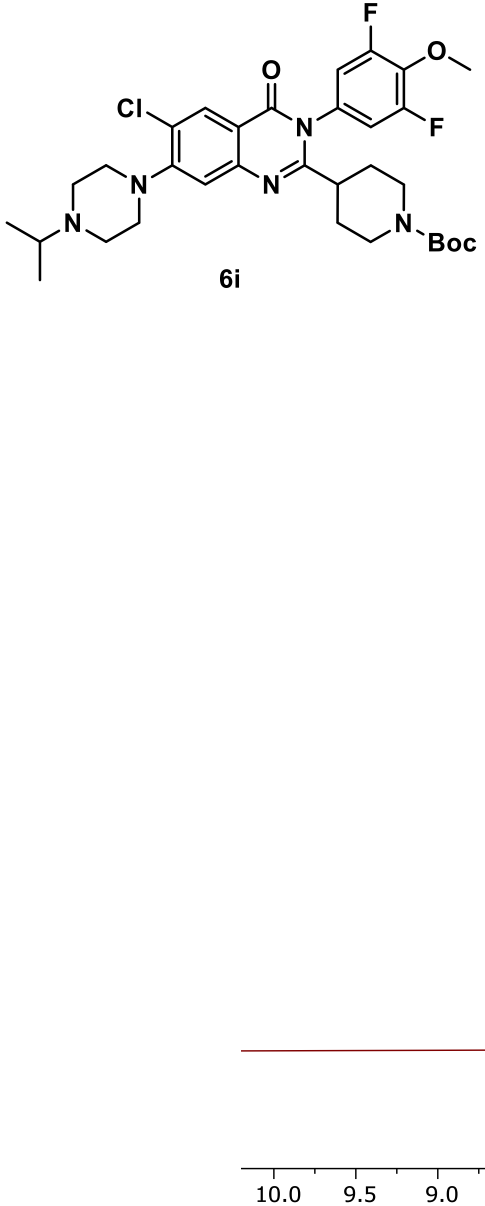

6i

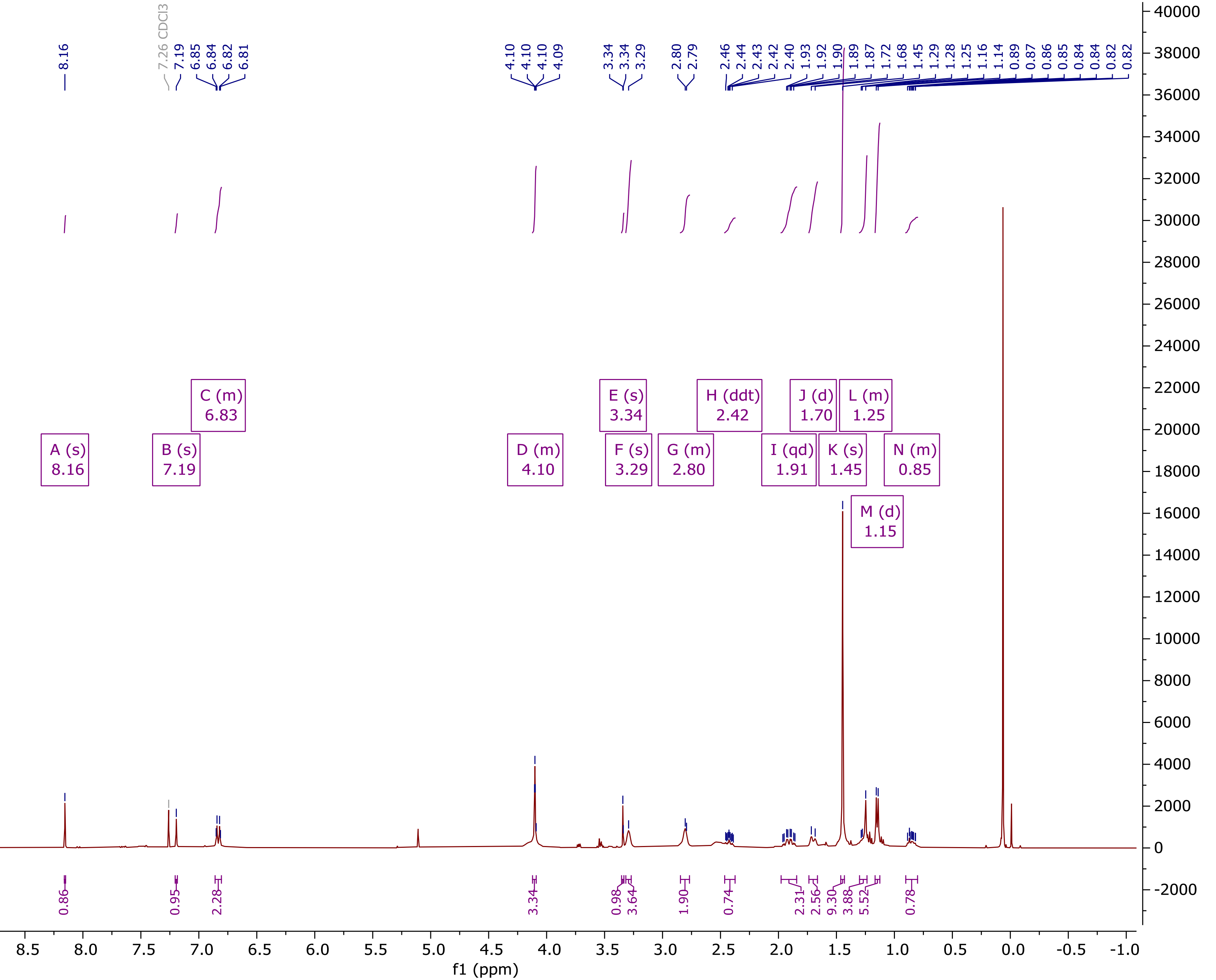

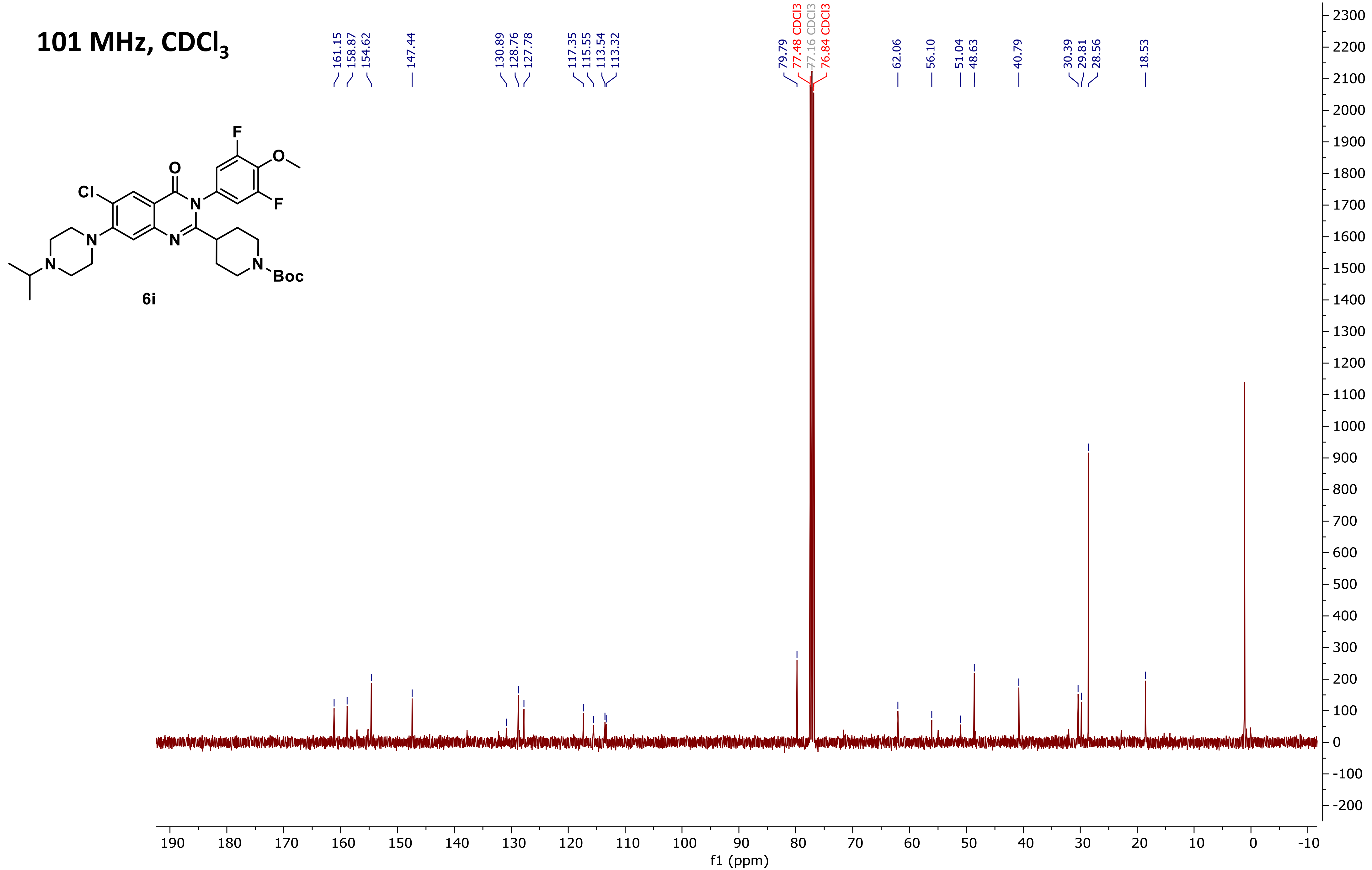
101 MHz, CDCl3
6i
Cl
O
N
N
F
O
F
N
N
N
Boc
161.15
158.87
154.62
147.44
130.89
128.76
127.78
117.35
115.55
113.54
113.32
79.79
77.48 CDCl3
77.16 CDCl3
76.84 CDCl3
62.06
56.10
51.04
48.63
40.79
30.39
29.81
28.56
18.53
f1 (ppm)

400 MHz, $CDCl_3$

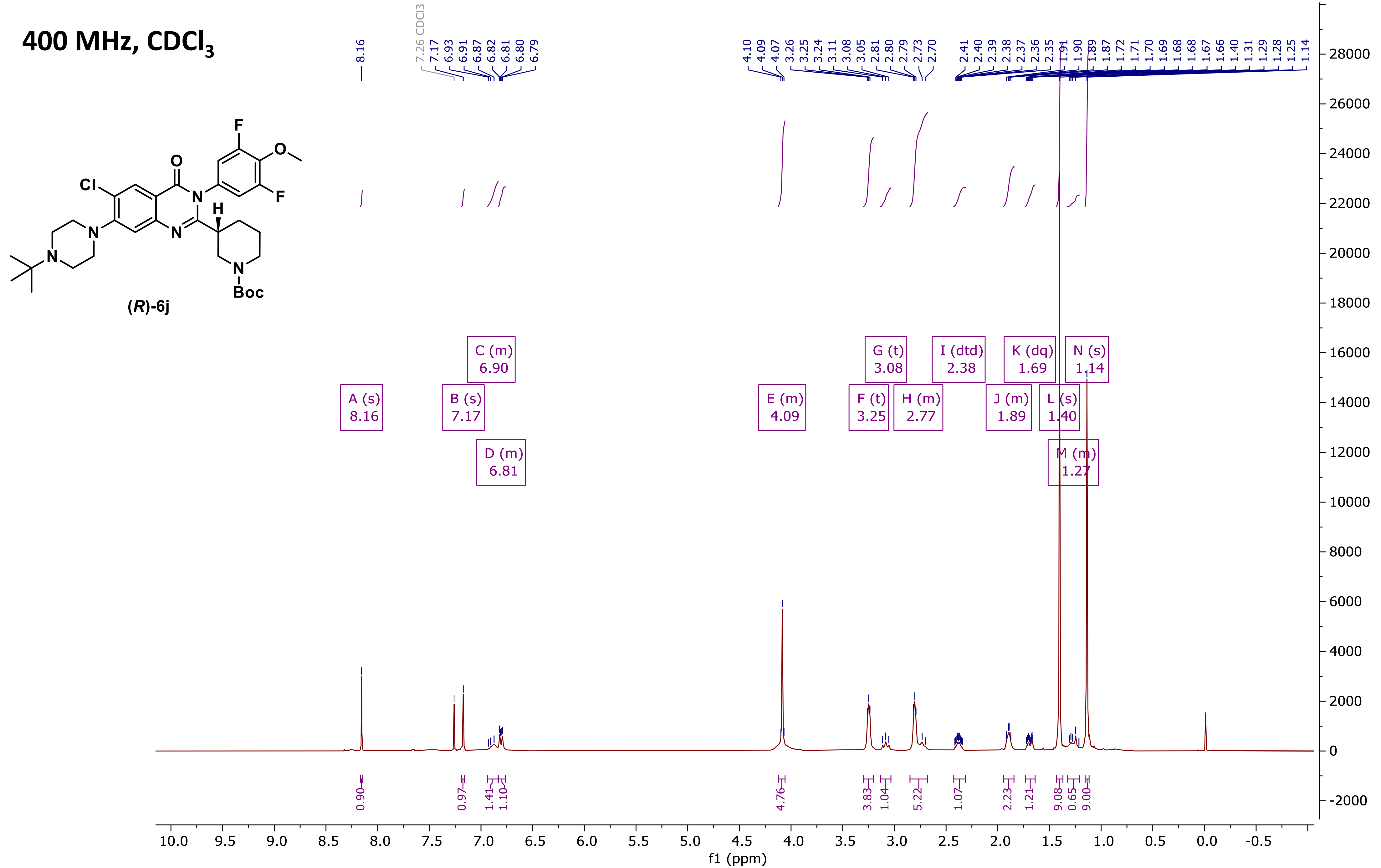

(*R*)-6j

101 MHz, $CDCl_3$

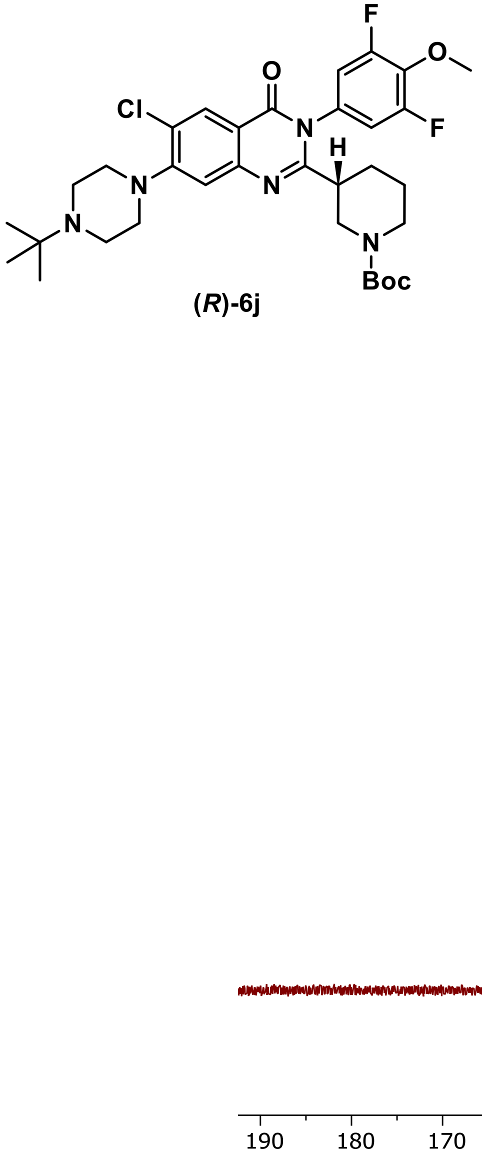

(*R*)-6j

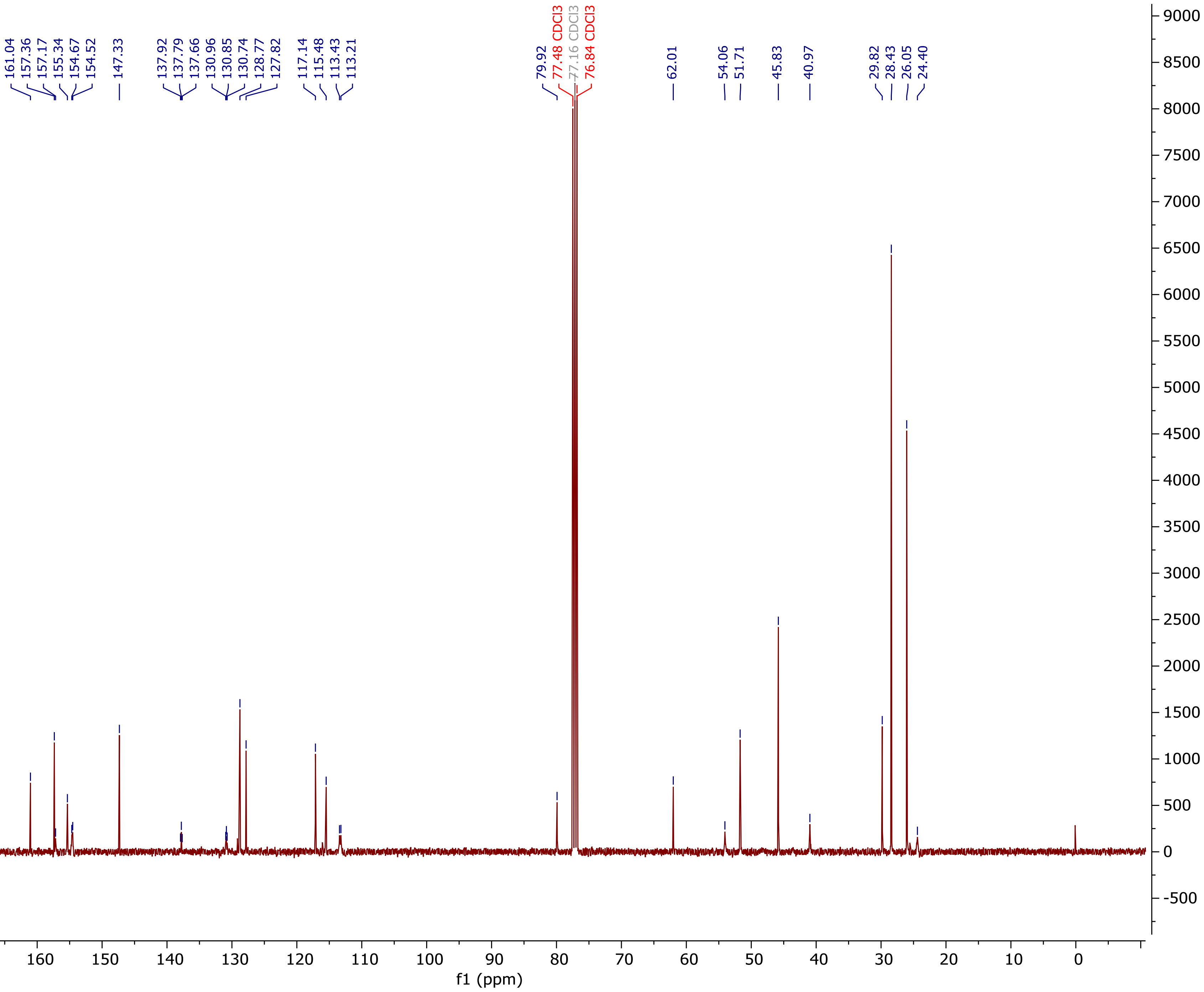

400 MHz, CDCl$_3$

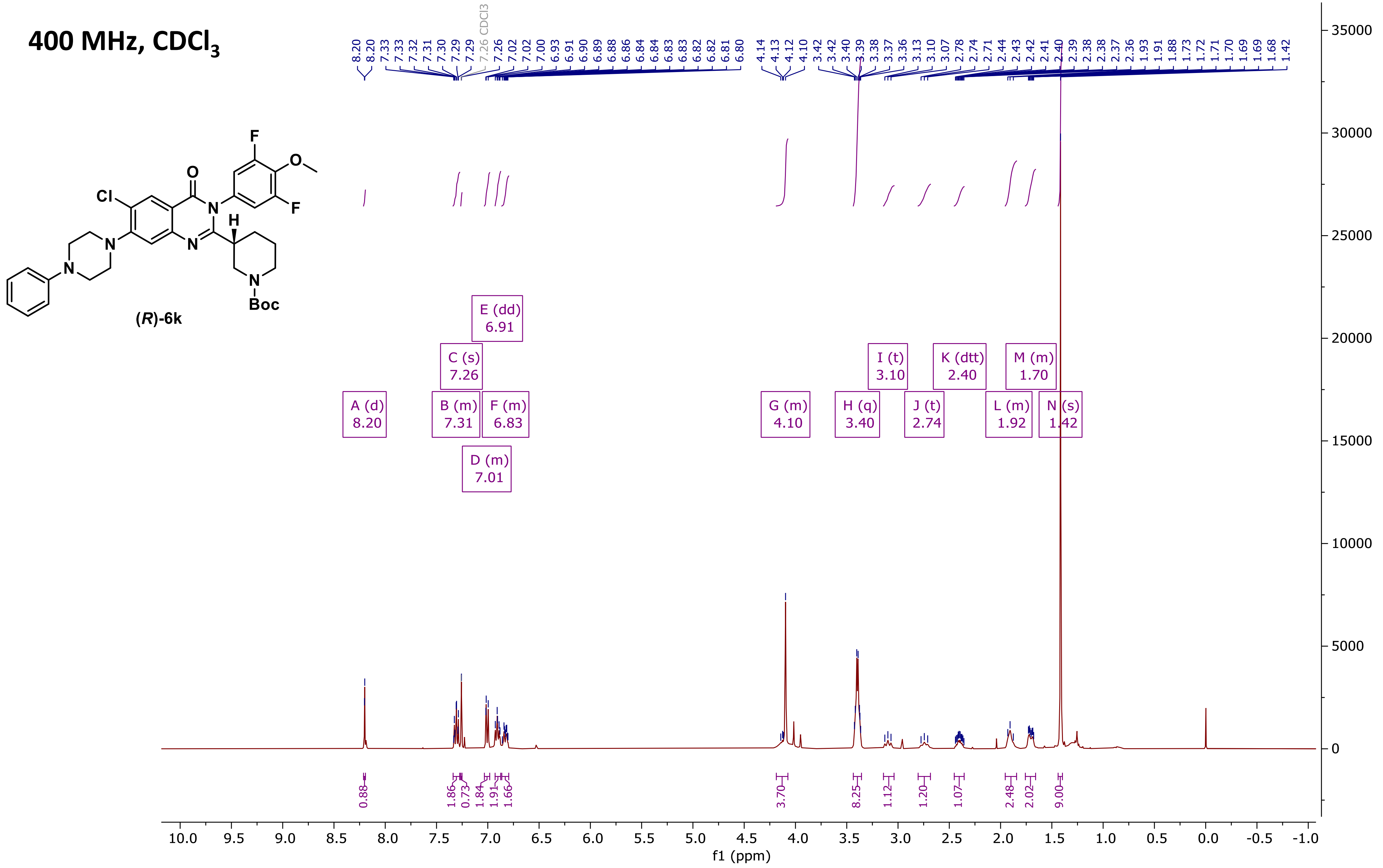

101 MHz, $CDCl_3$

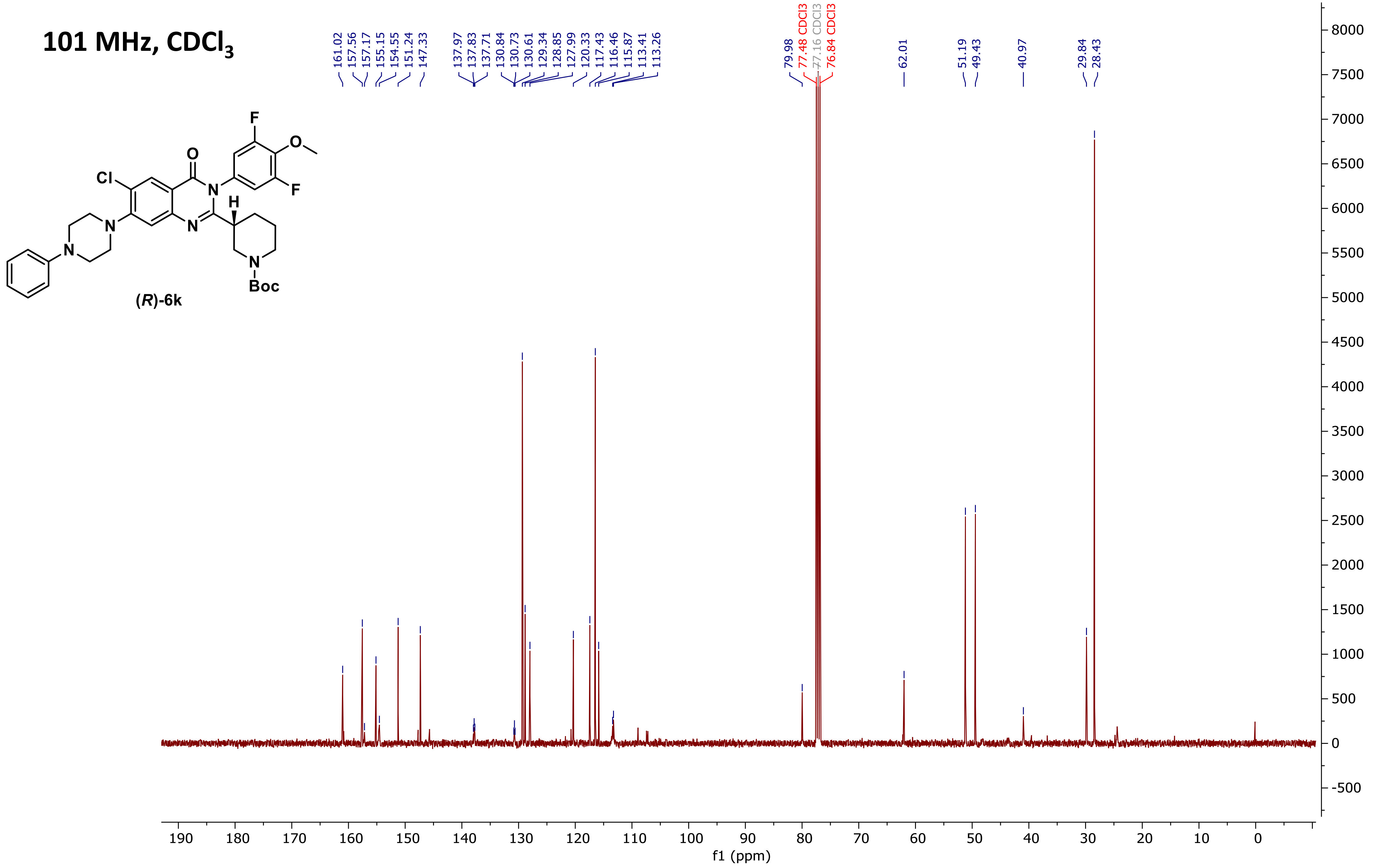

400 MHz, $CDCl_3$

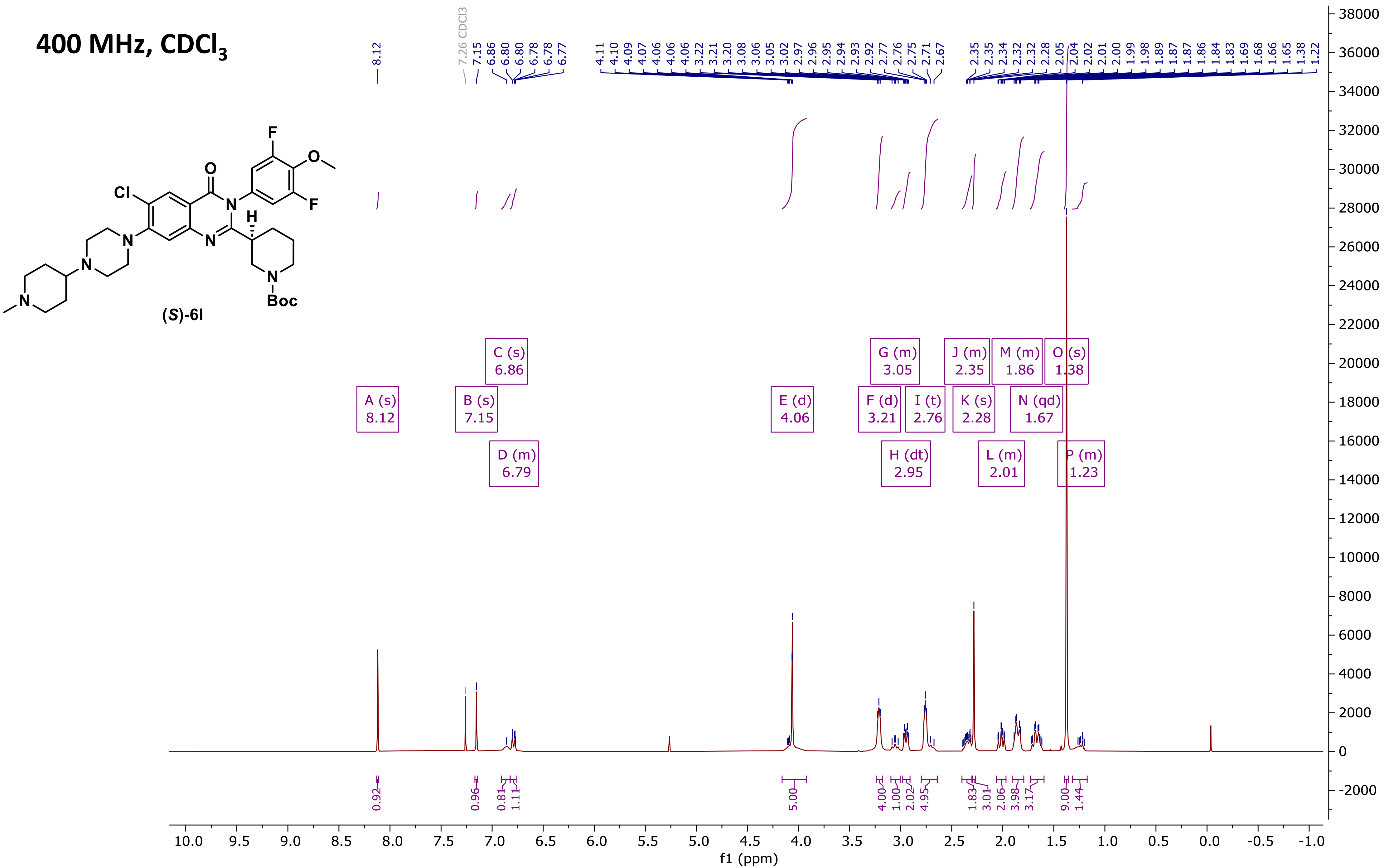

101 MHz, $CDCl_3$

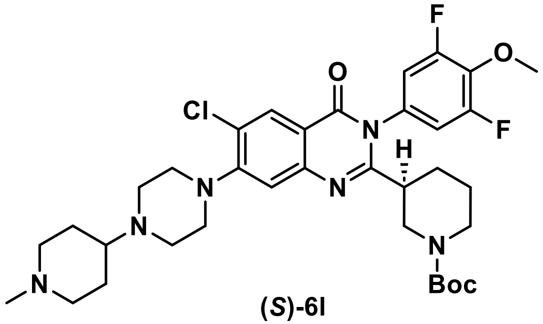

(*S*)-6l

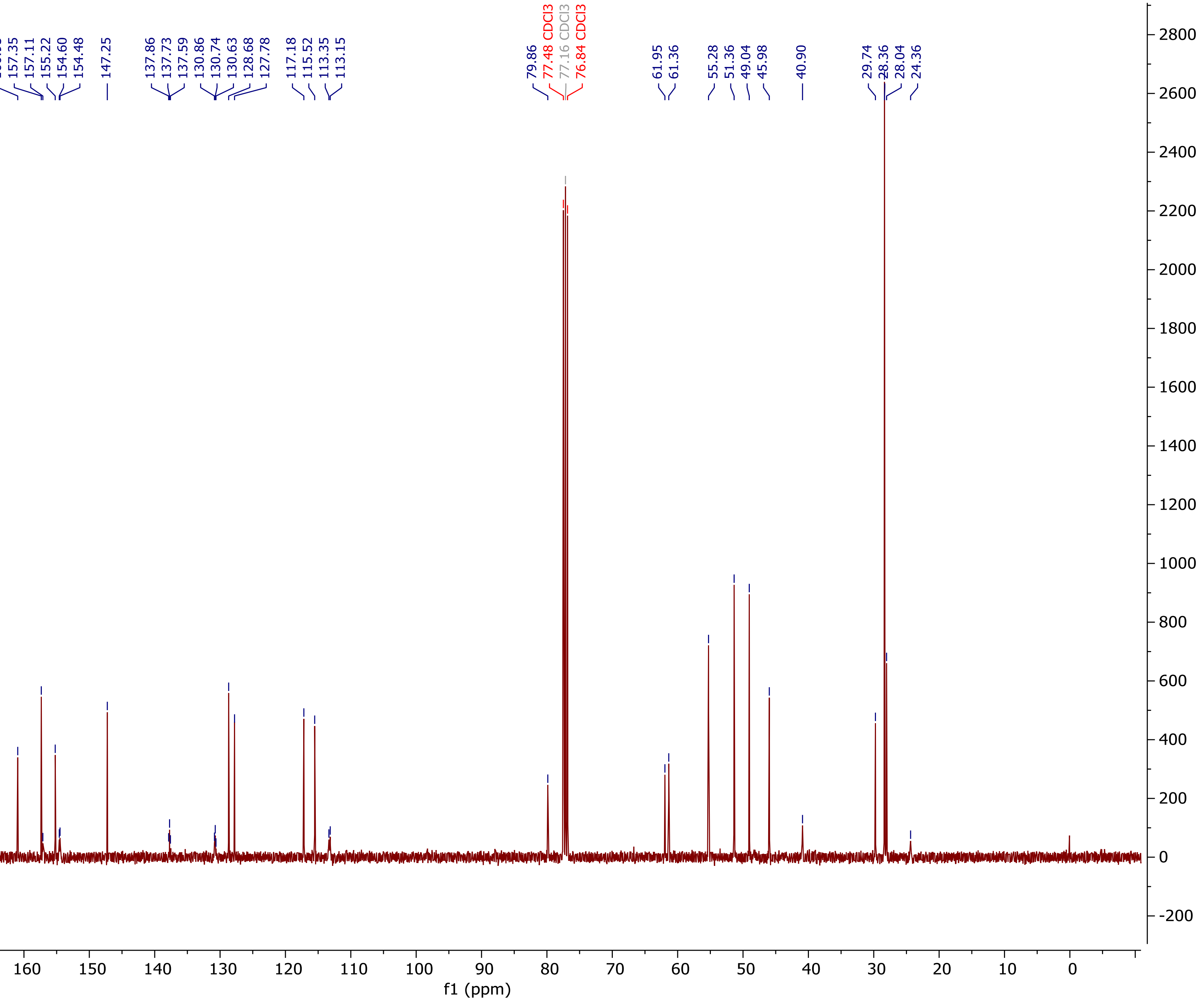

400 MHz, $CDCl_3$

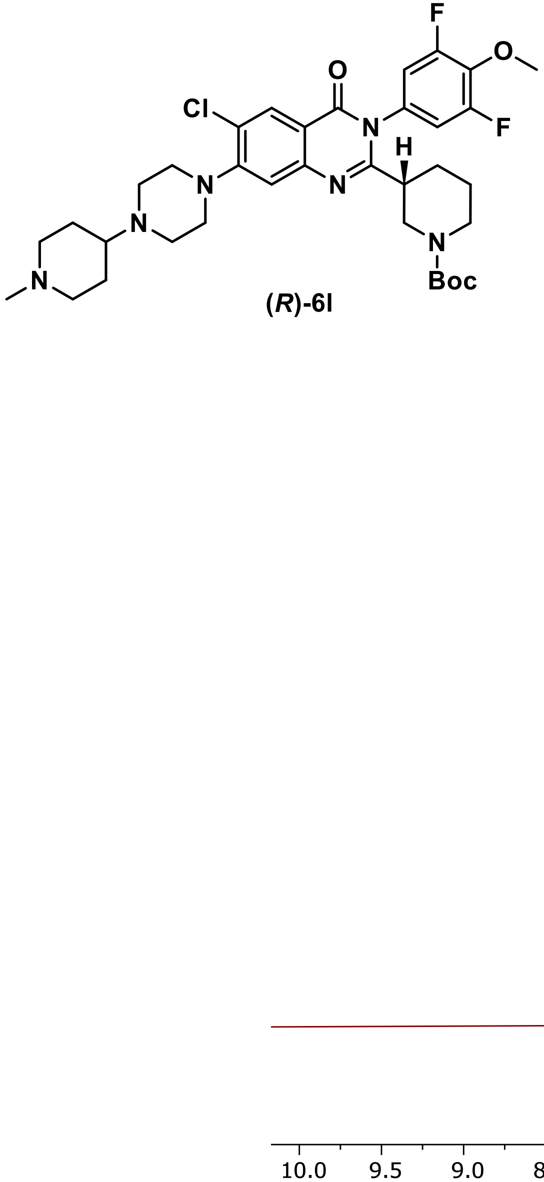

(*R*)-6l

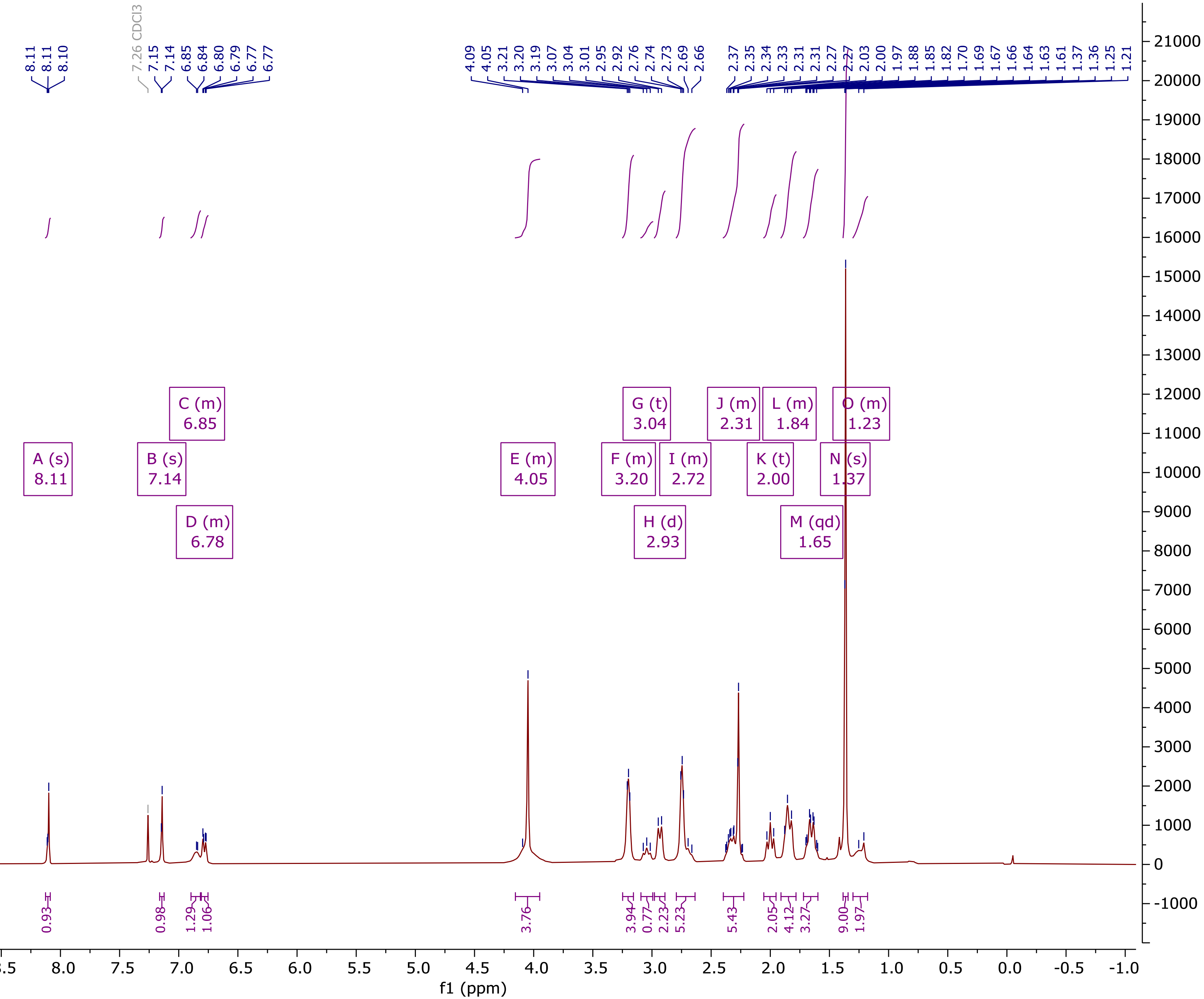

101 MHz, $CDCl_3$

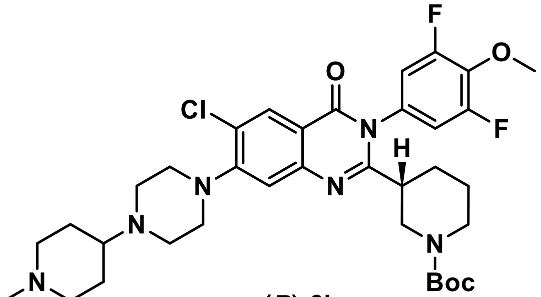

(*R*)-6l

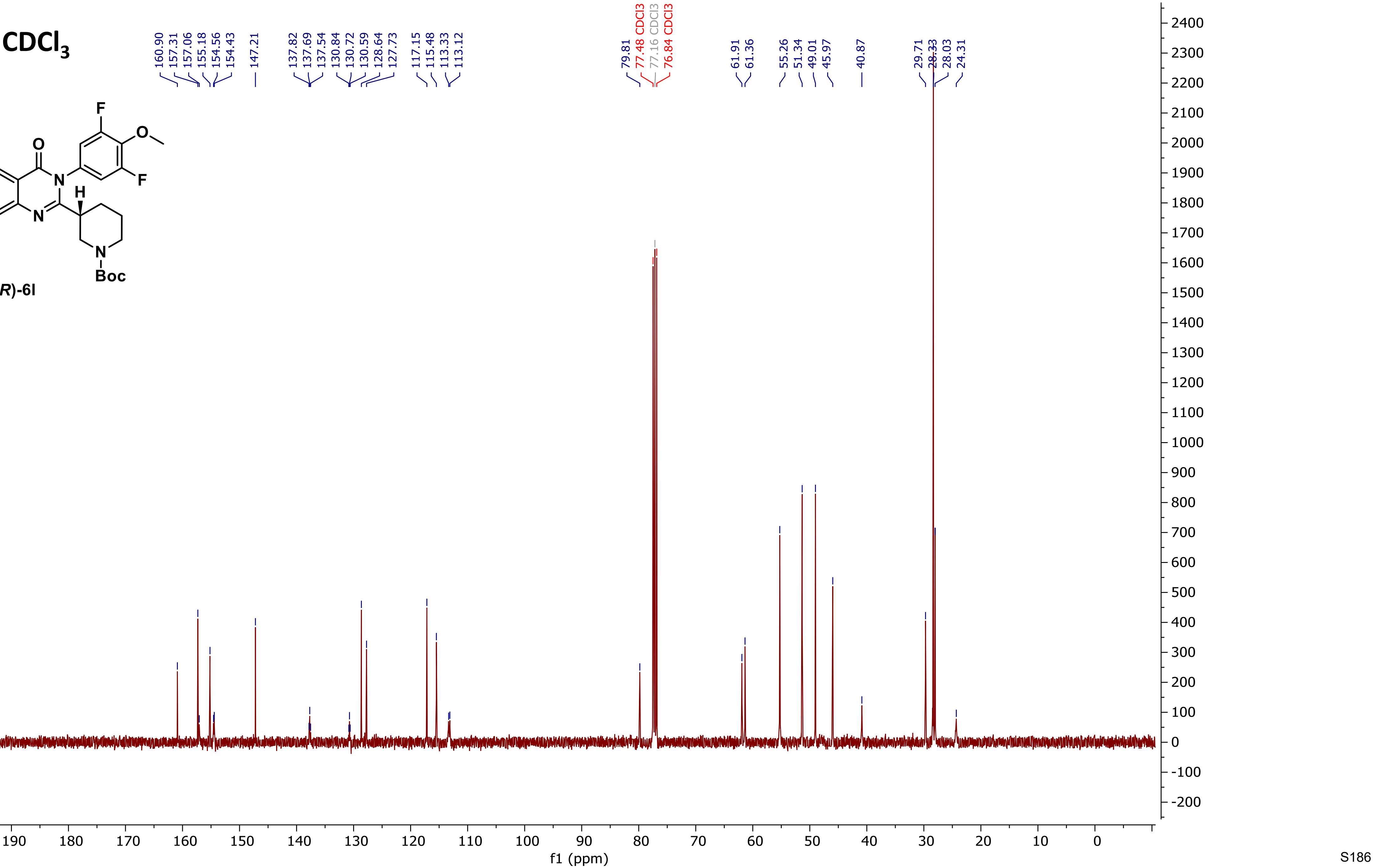

400 MHz, $CDCl_3$

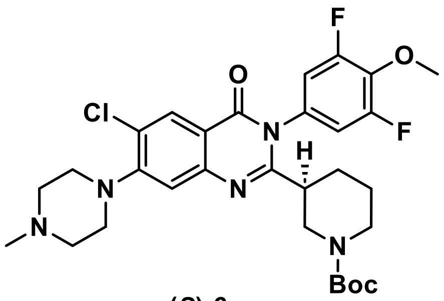

(*S*)-6m

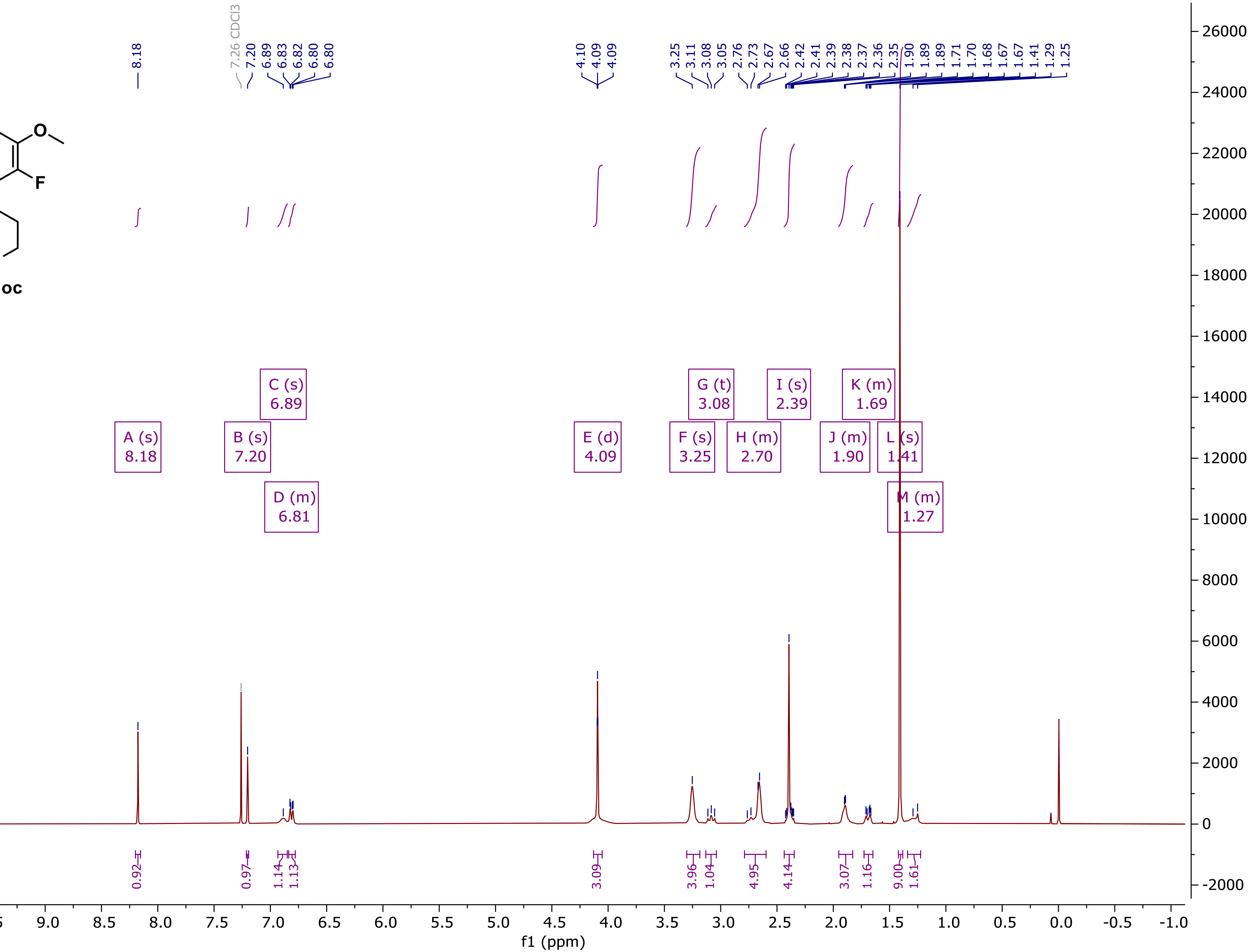

101 MHz, $CDCl_3$

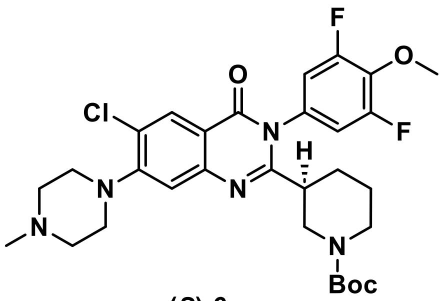

(*S*)-6m

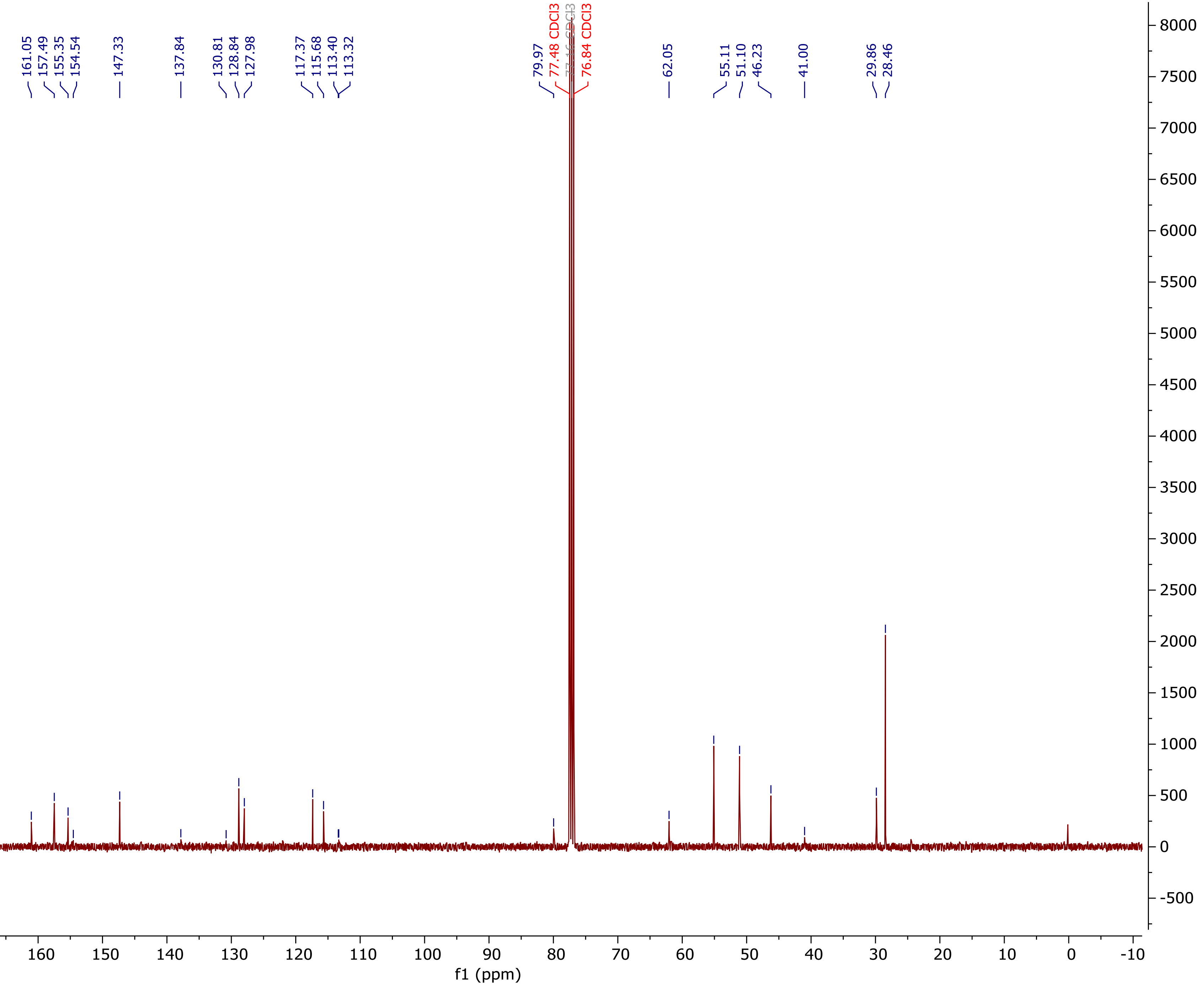

400 MHz, $CDCl_3$

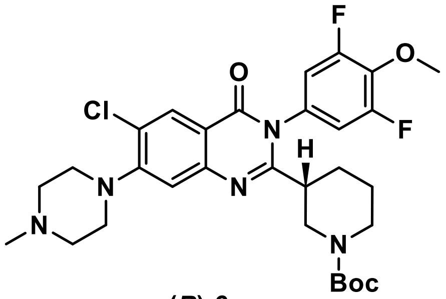

(*R*)-6m

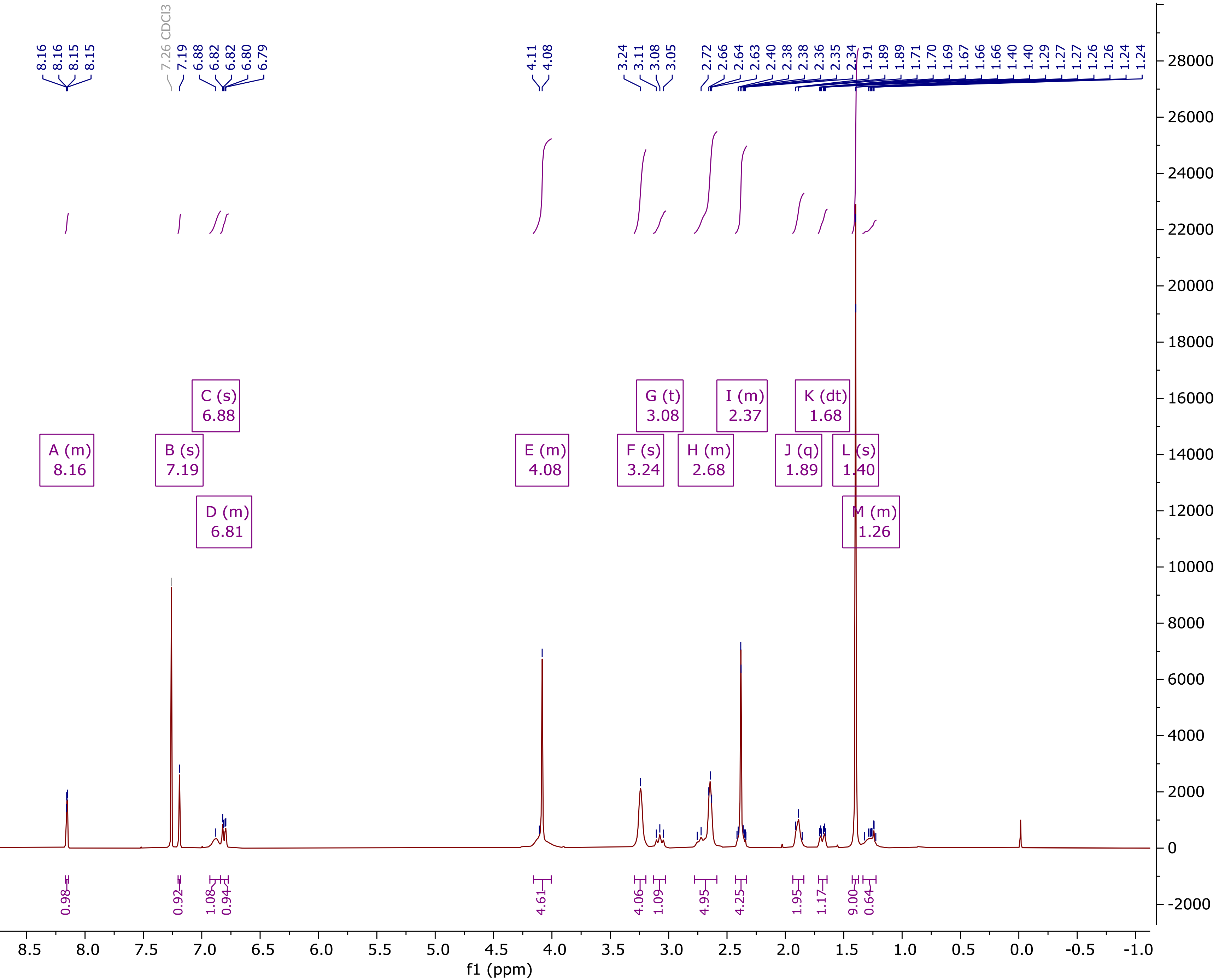

101 MHz, $CDCl_3$

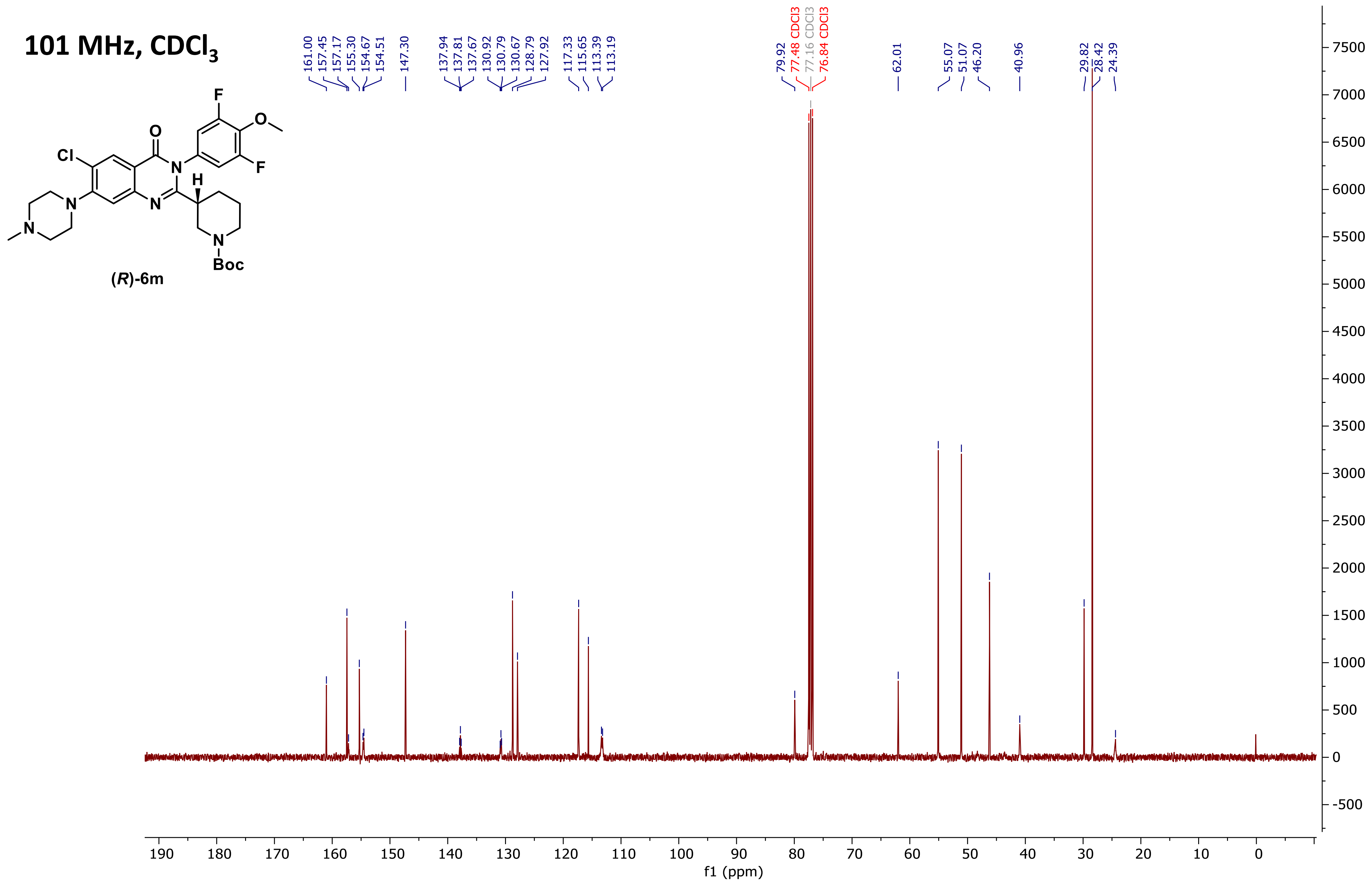

400 MHz, $CDCl_3$

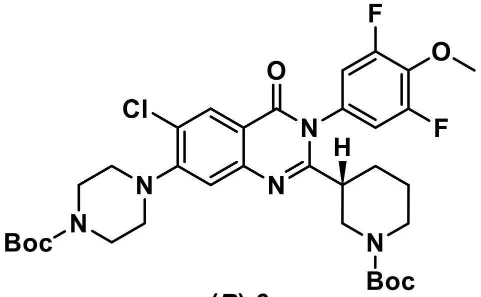

(*R*)-6n

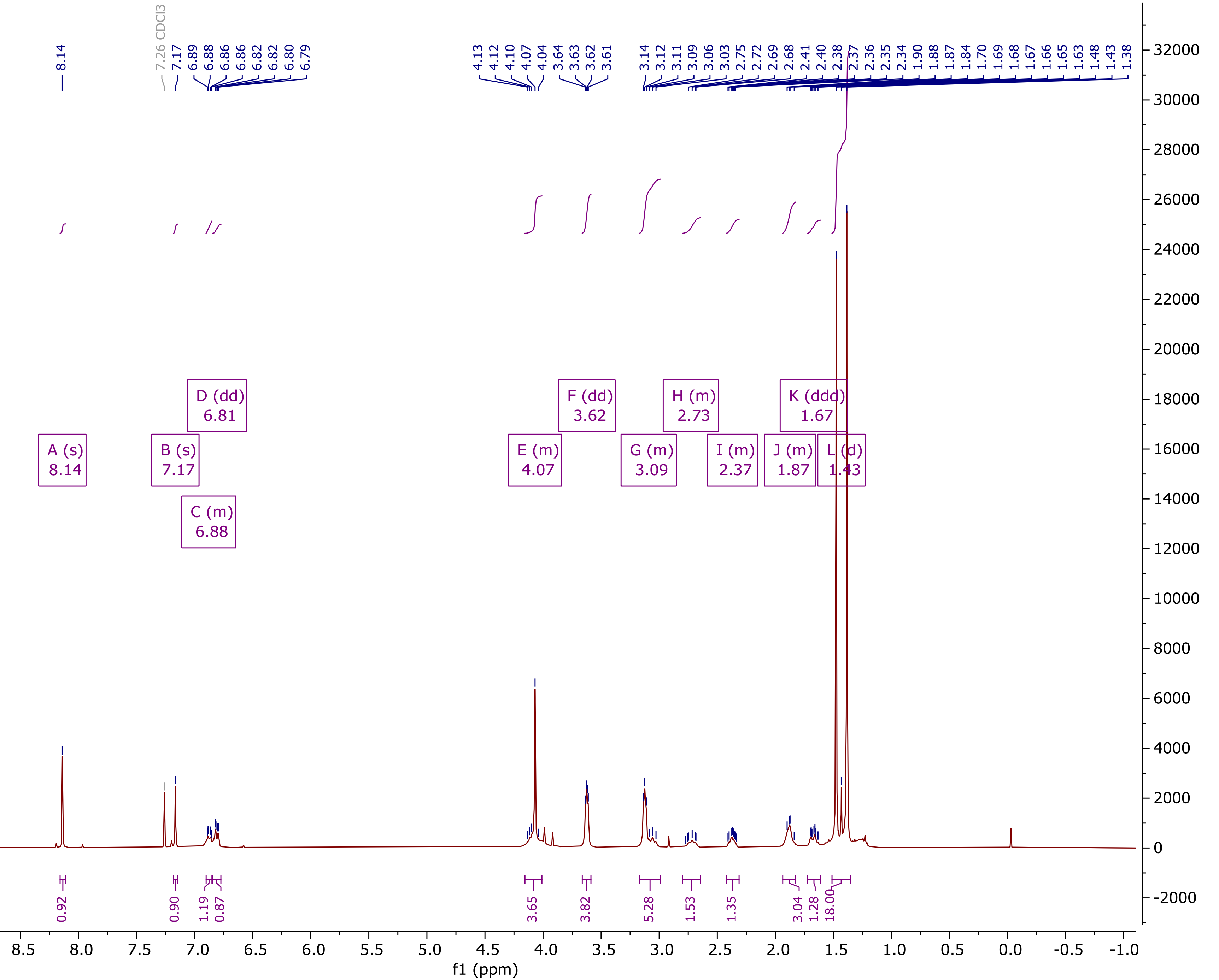

101 MHz, $CDCl_3$

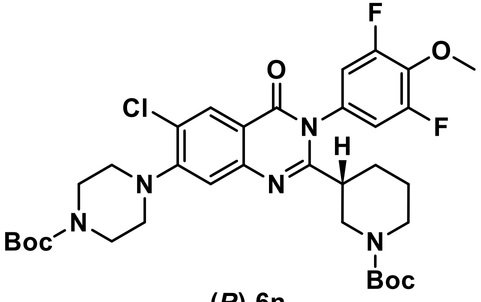

(*R*)-6n

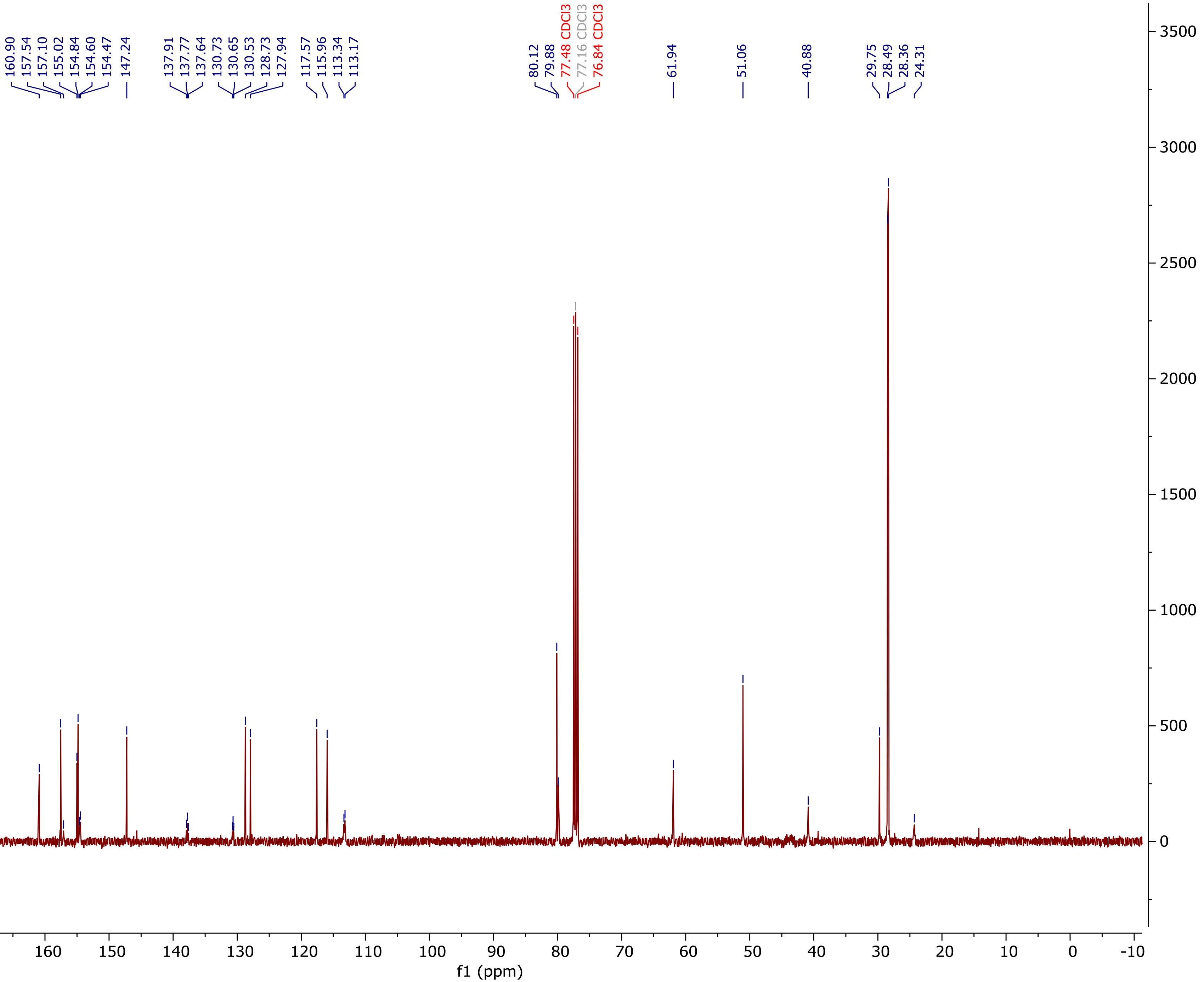

400 MHz, $CDCl_3$

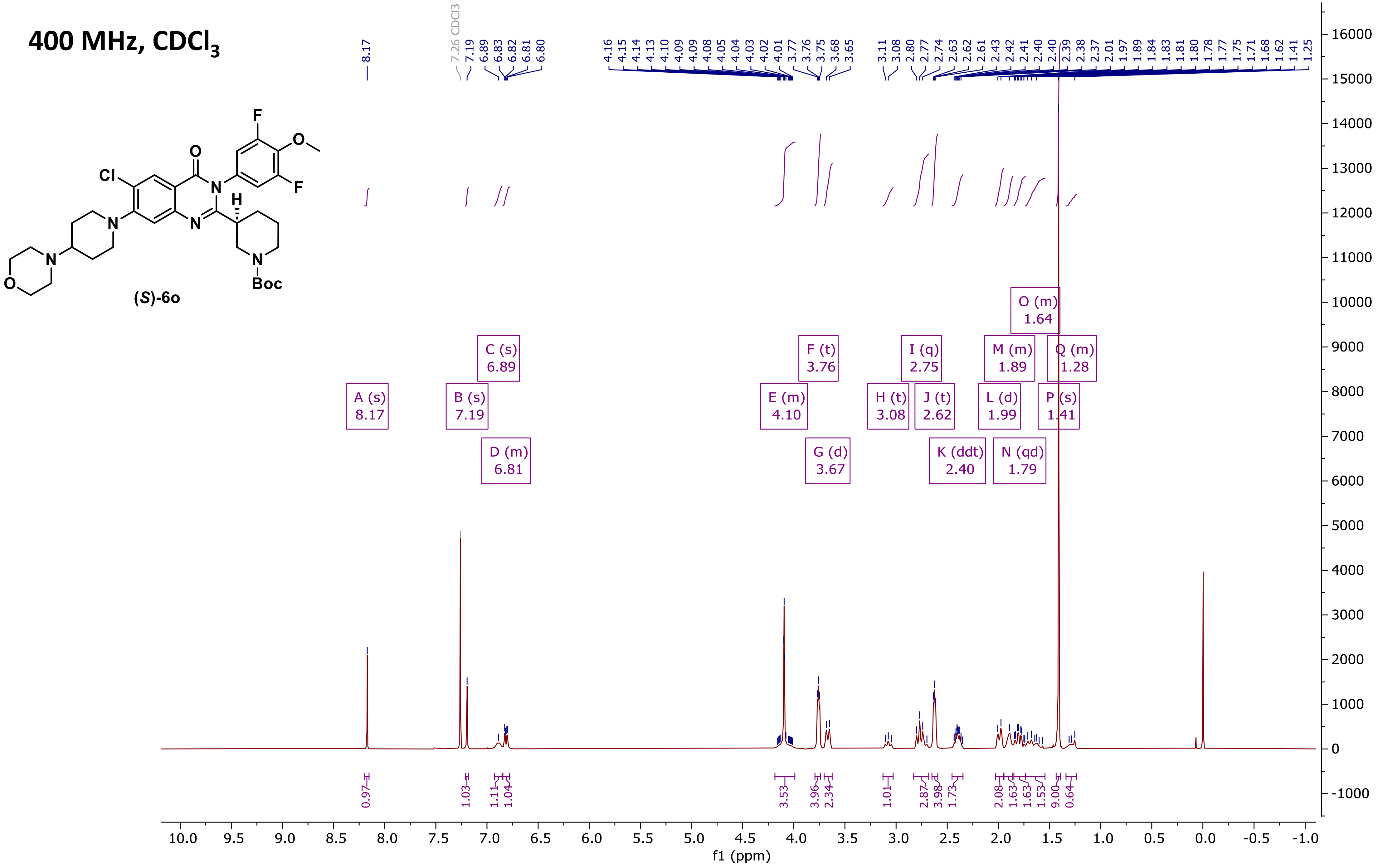

(*S*)-6o

101 MHz, $CDCl_3$

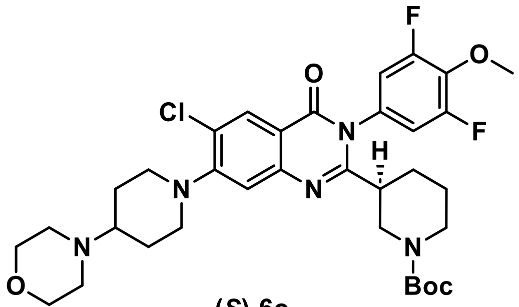

(*S*)-6o

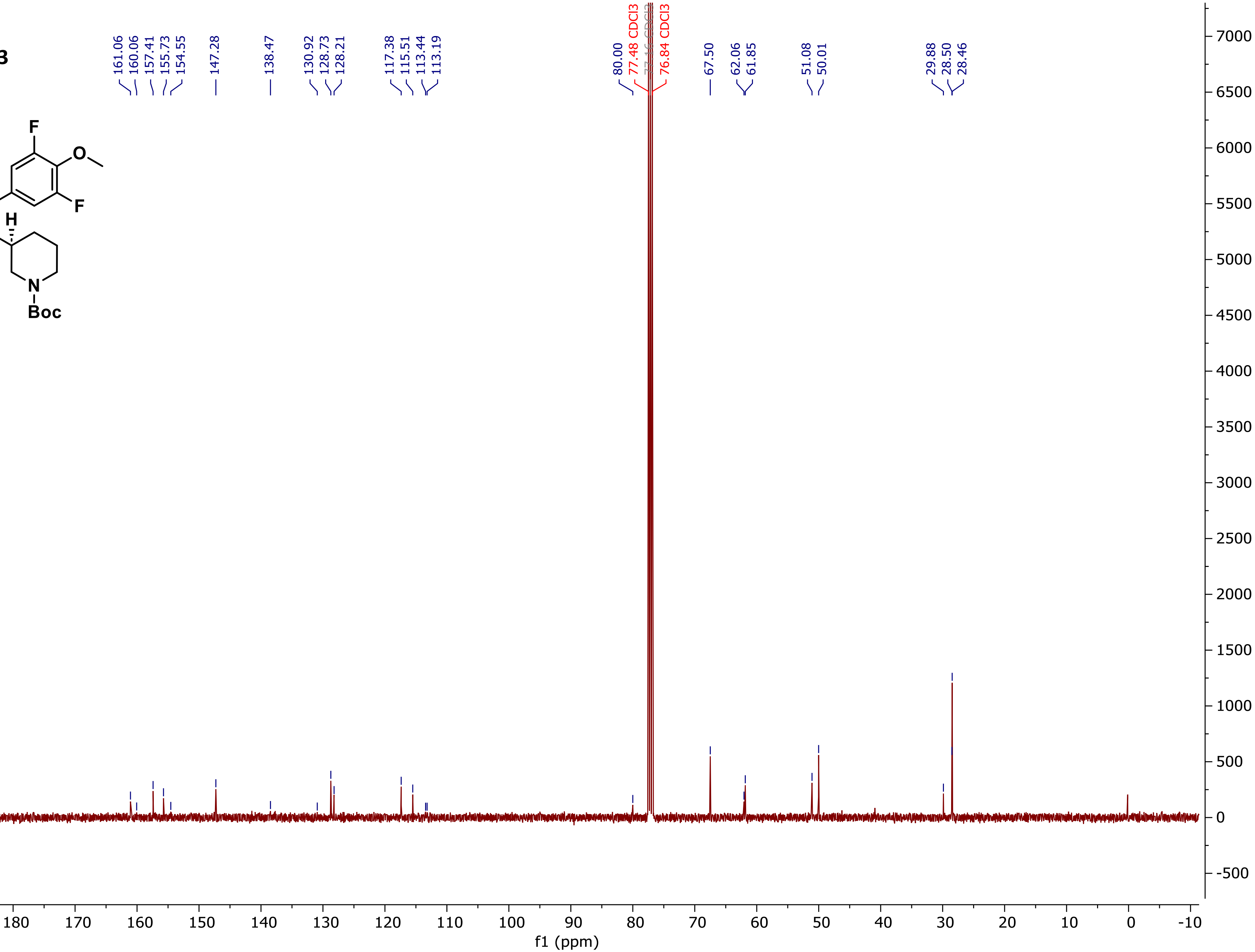

400 MHz, $CDCl_3$

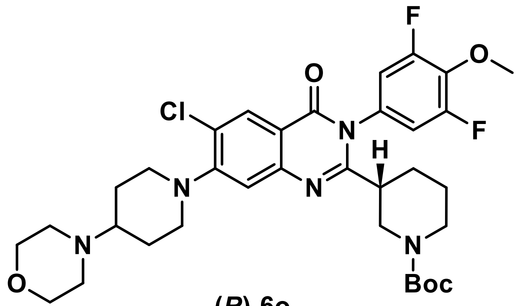

(*R*)-6o

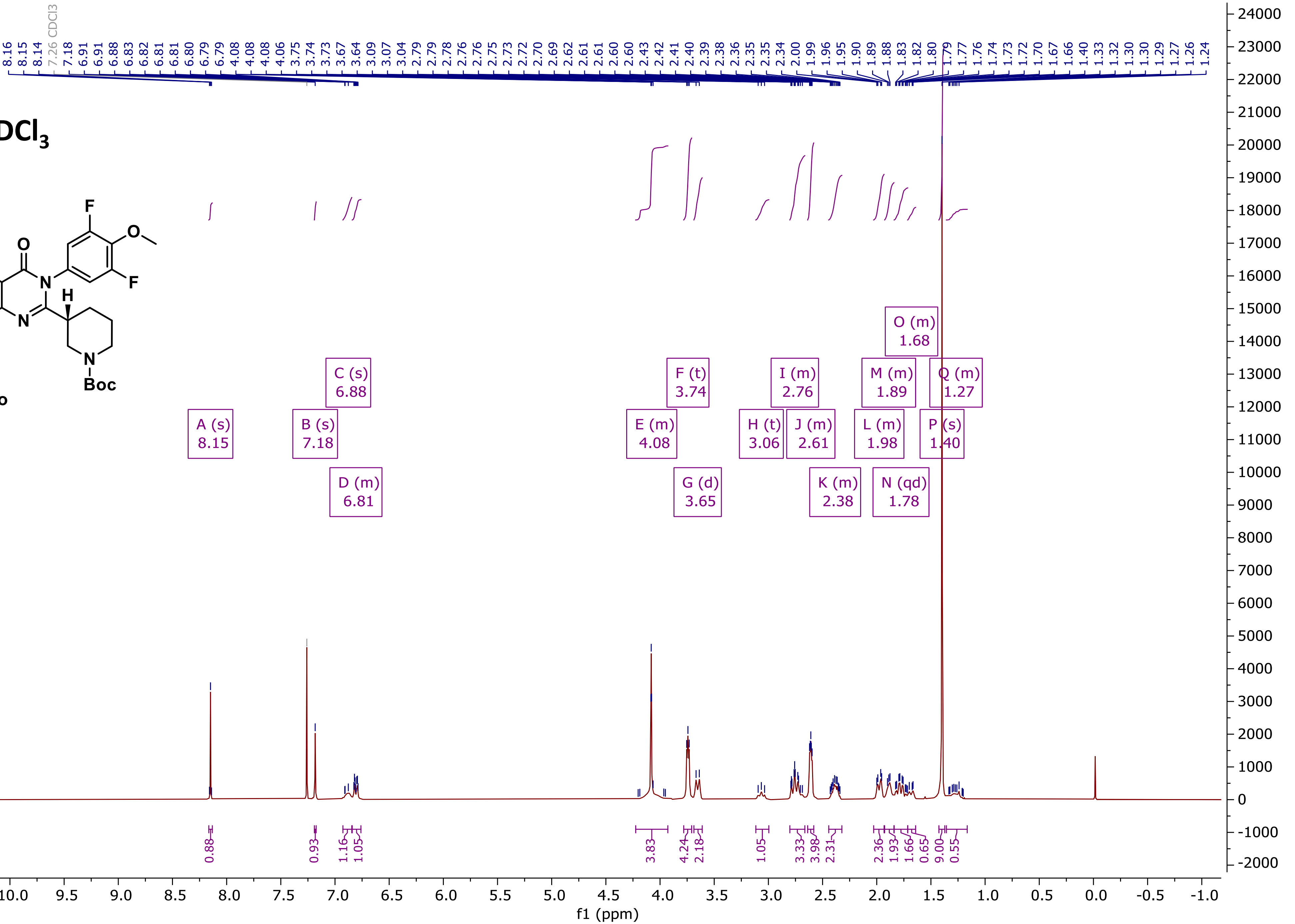

101 MHz, CDCl$_3$

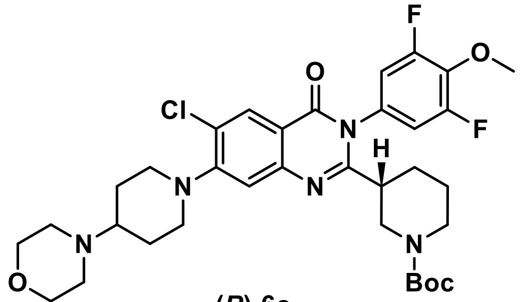
(R)-6o

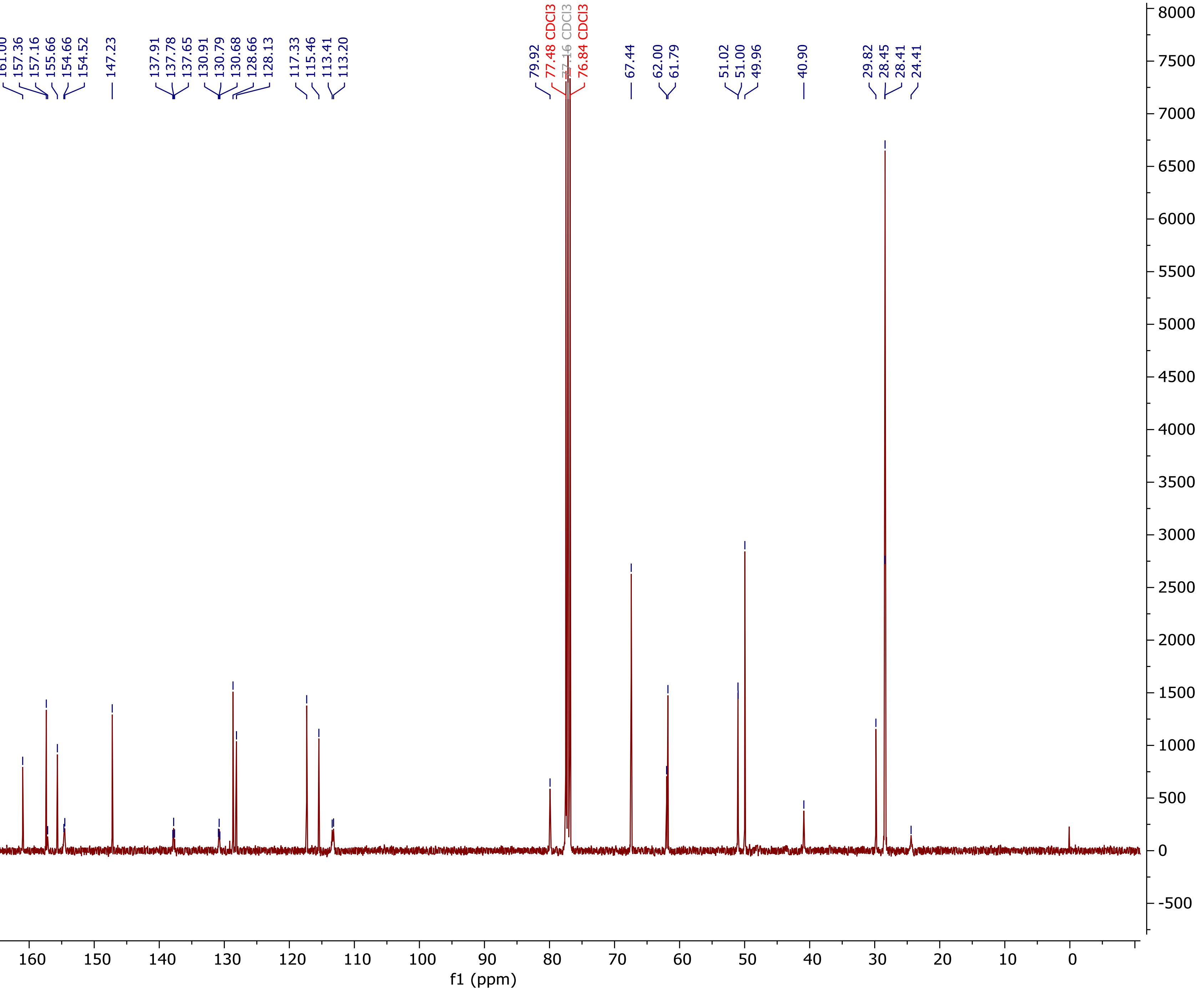

400 MHz, $CDCl_3$

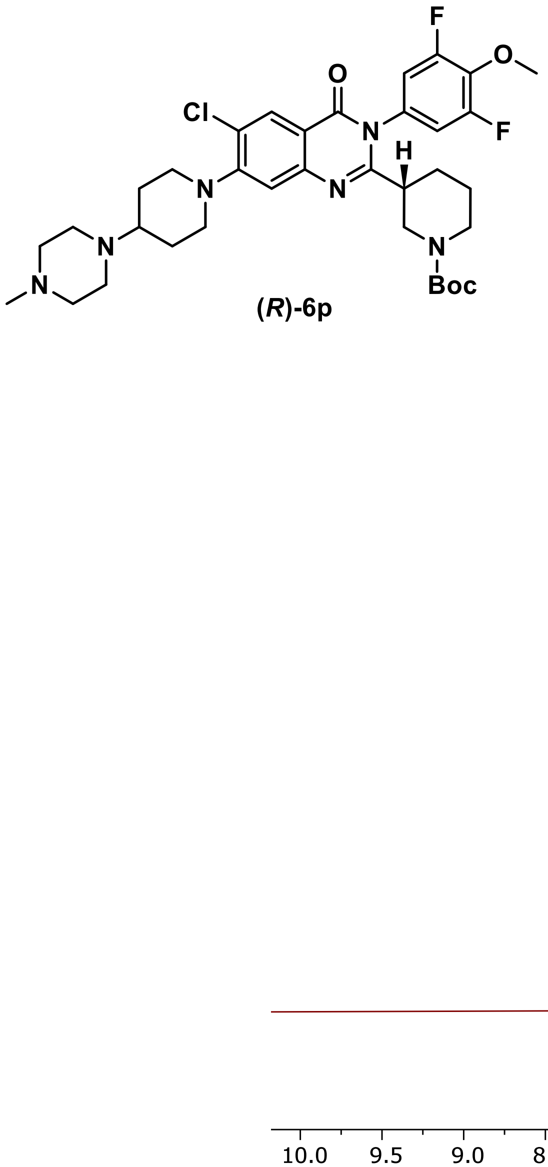

(*R*)-6p

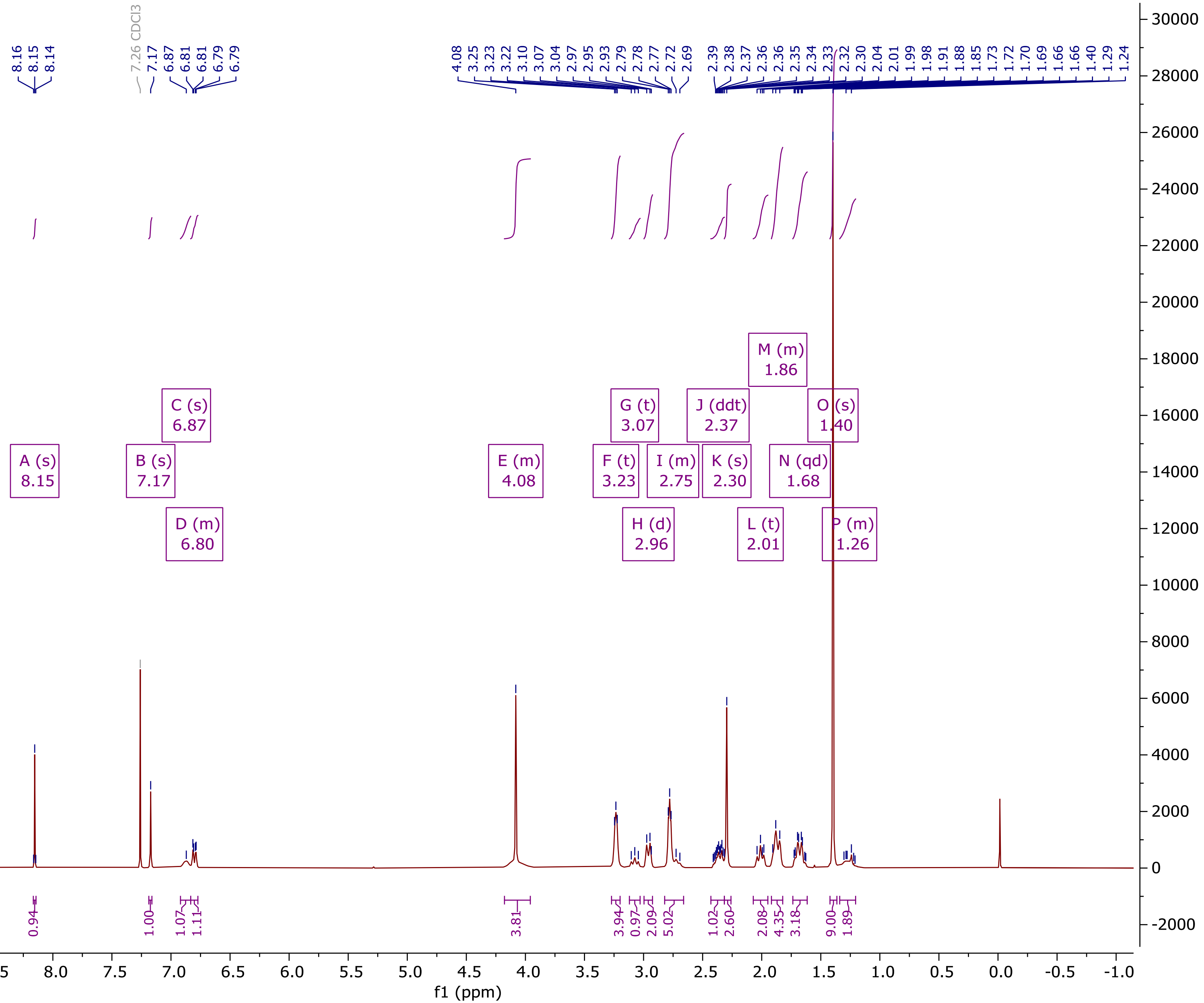

101 MHz, $CDCl_3$

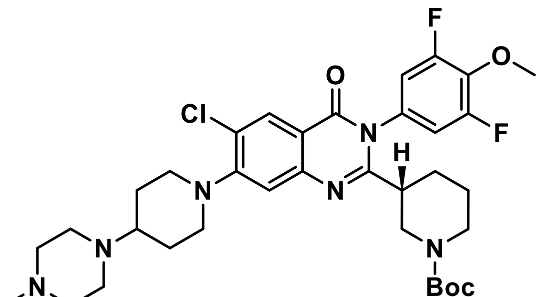

(*R*)-6p

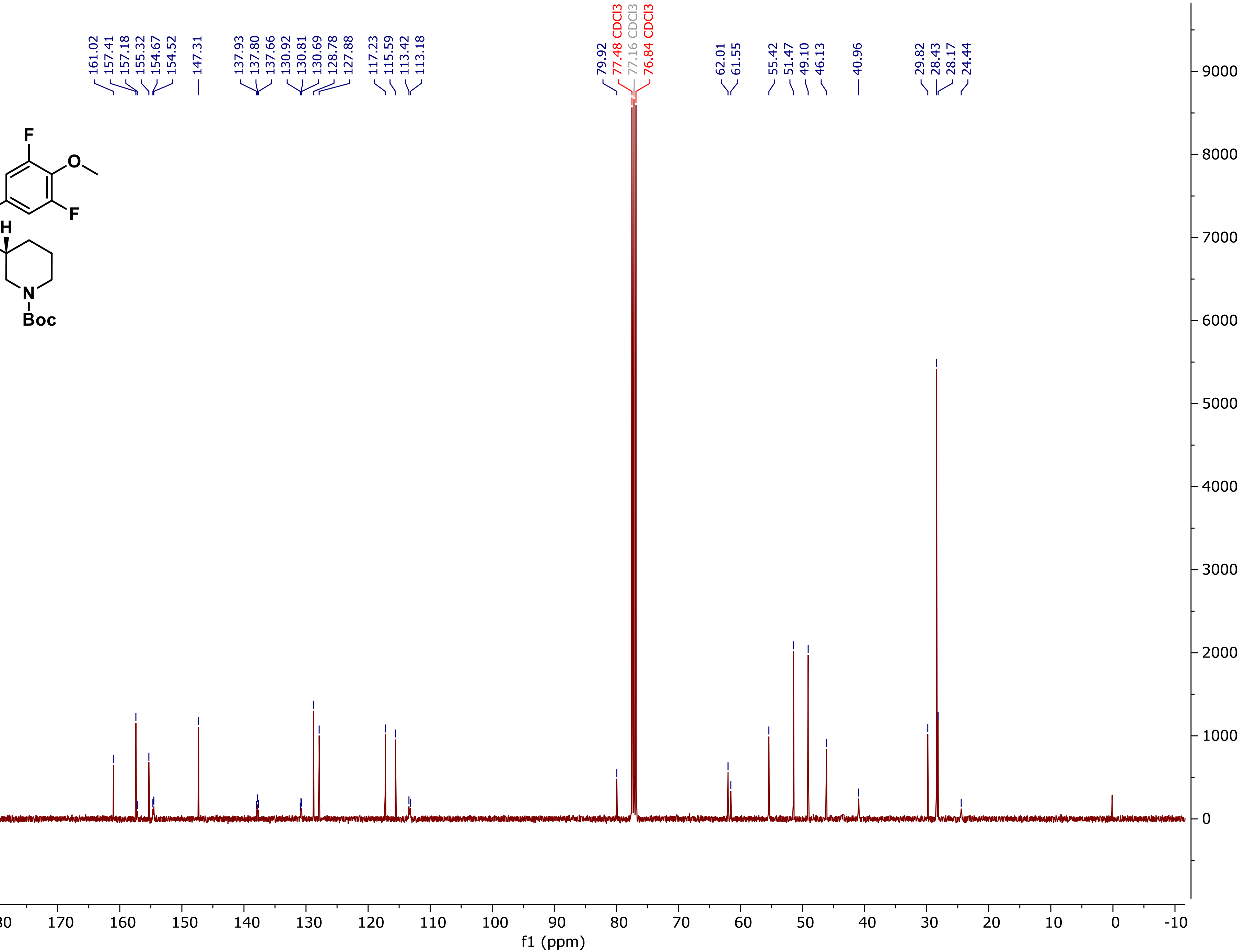

400 MHz, CDCl$_3$

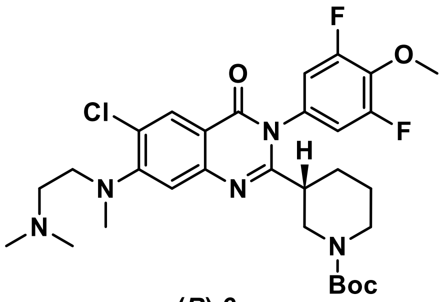

(*R*)-6q

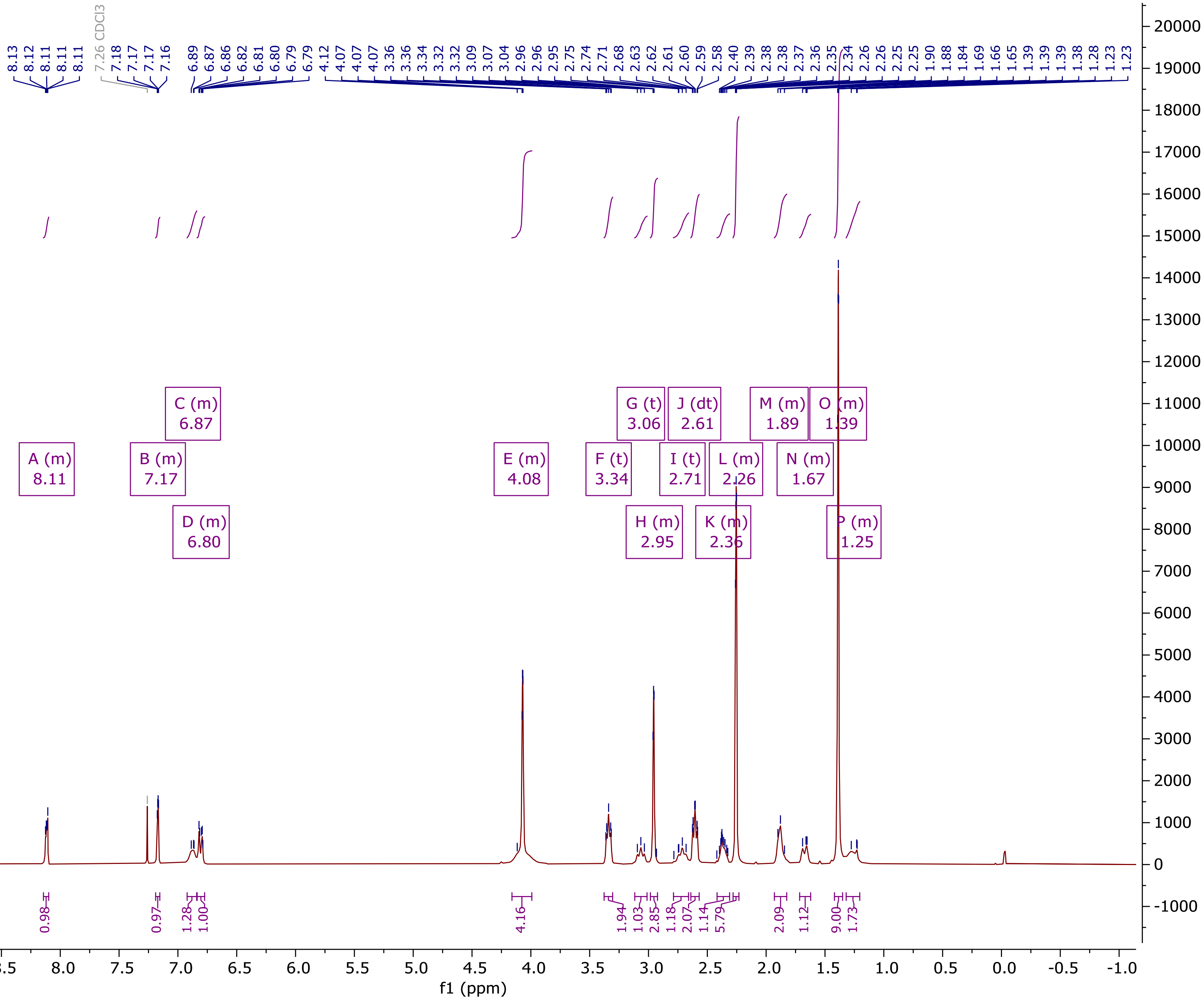

101 MHz, $CDCl_3$

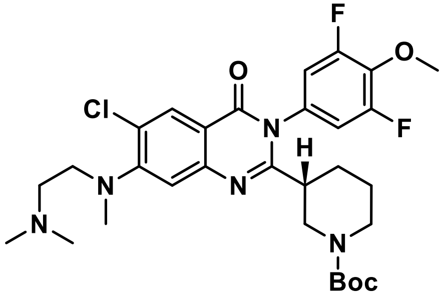

(*R*)-6q

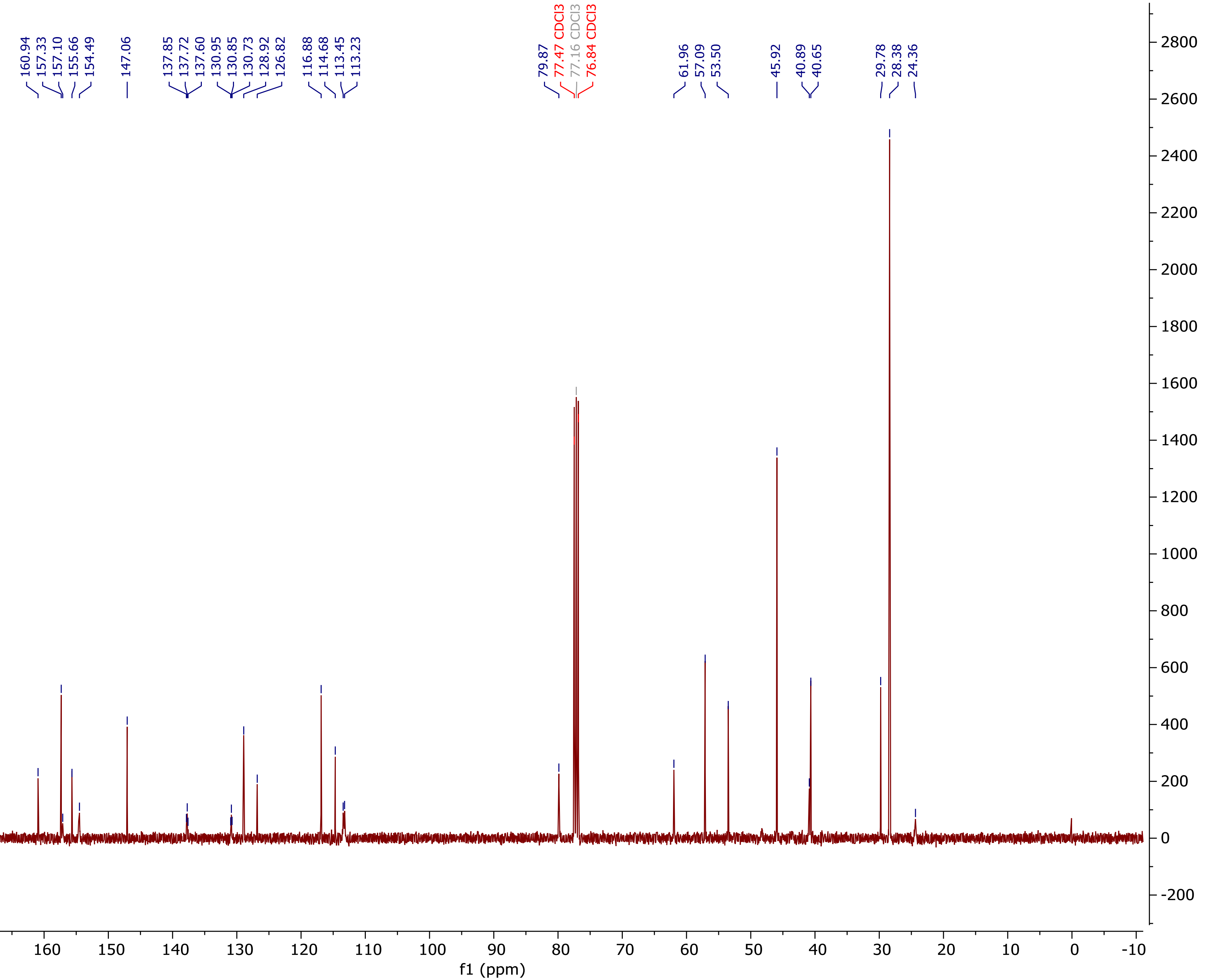

400 MHz, $CDCl_3$

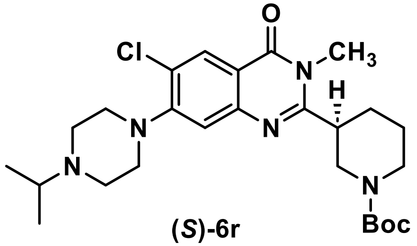

(S)-6r

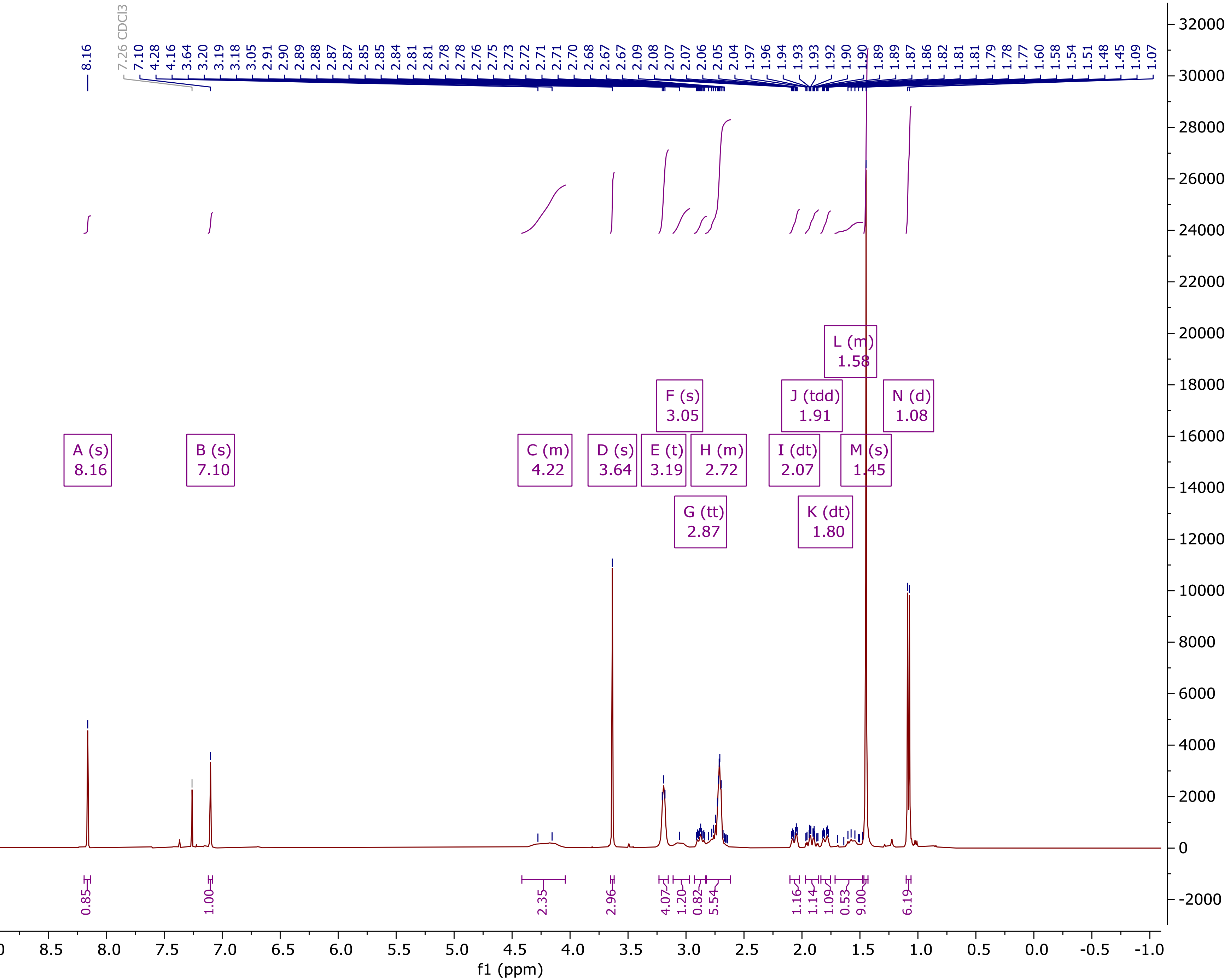

101 MHz, $CDCl_3$

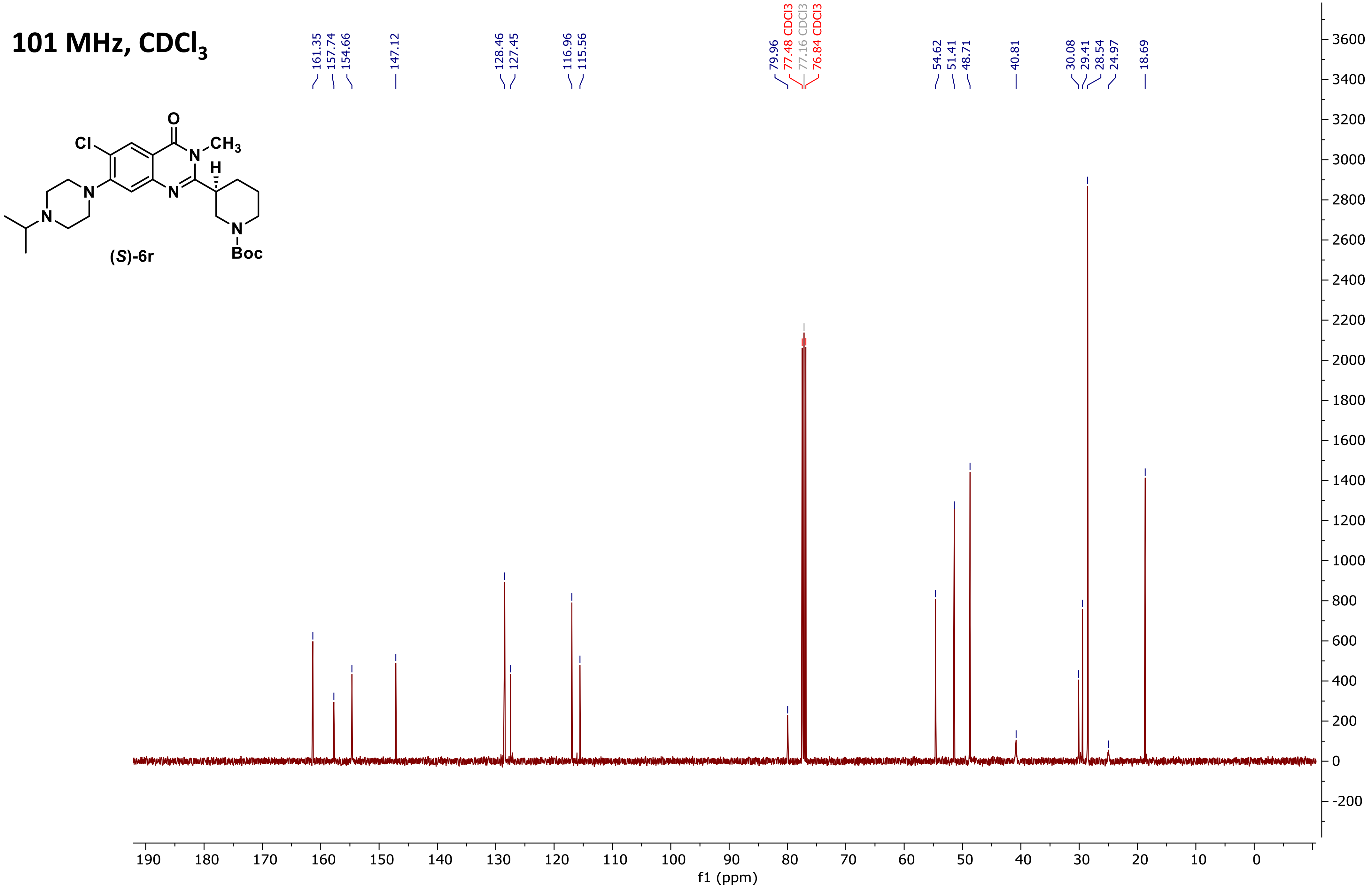

400 MHz, $CDCl_3$

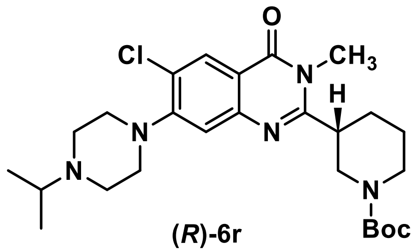

(*R*)-6r

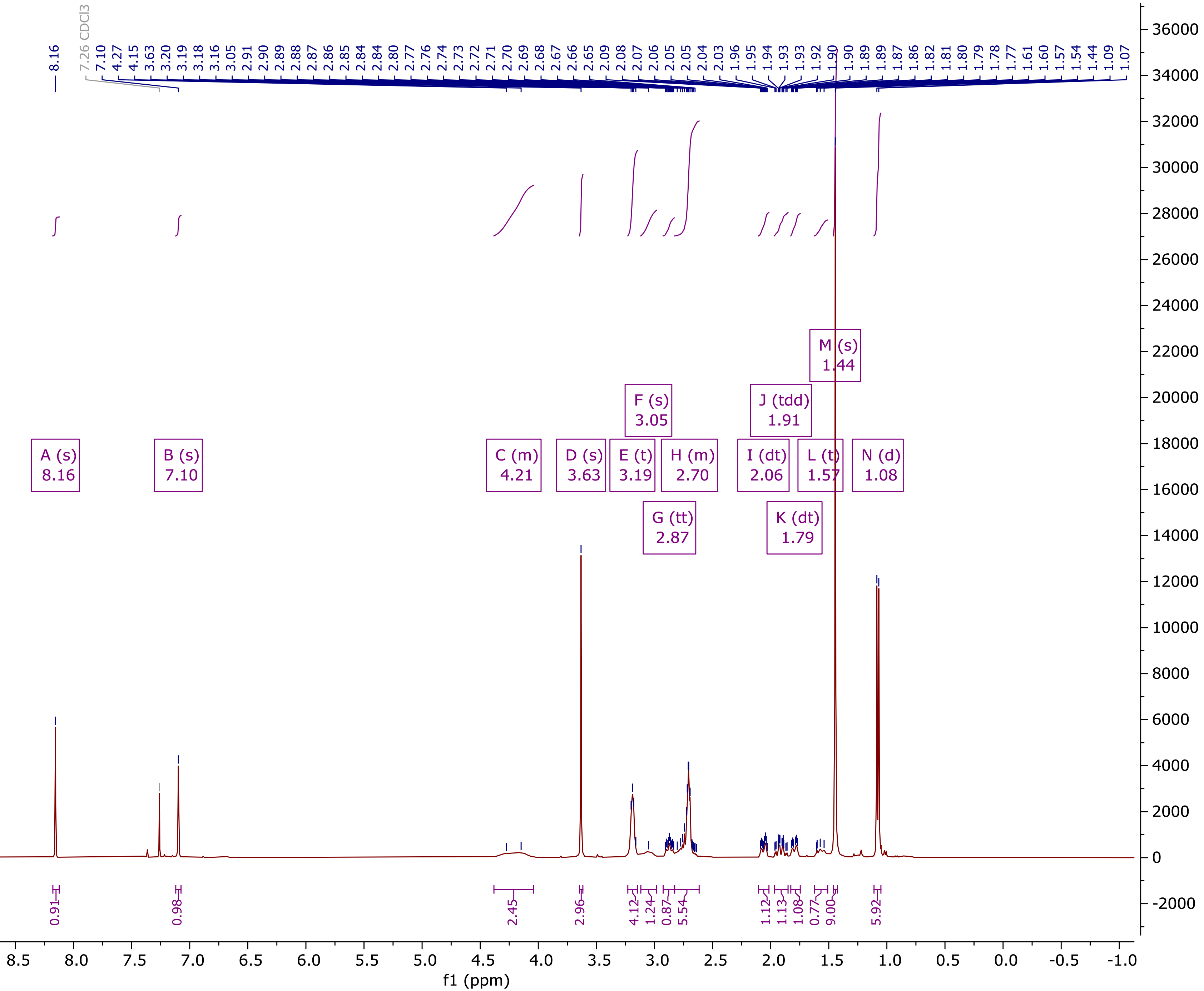

101 MHz, $CDCl_3$

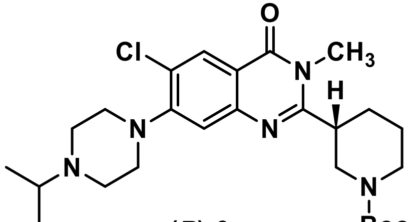

(*R*)-6r

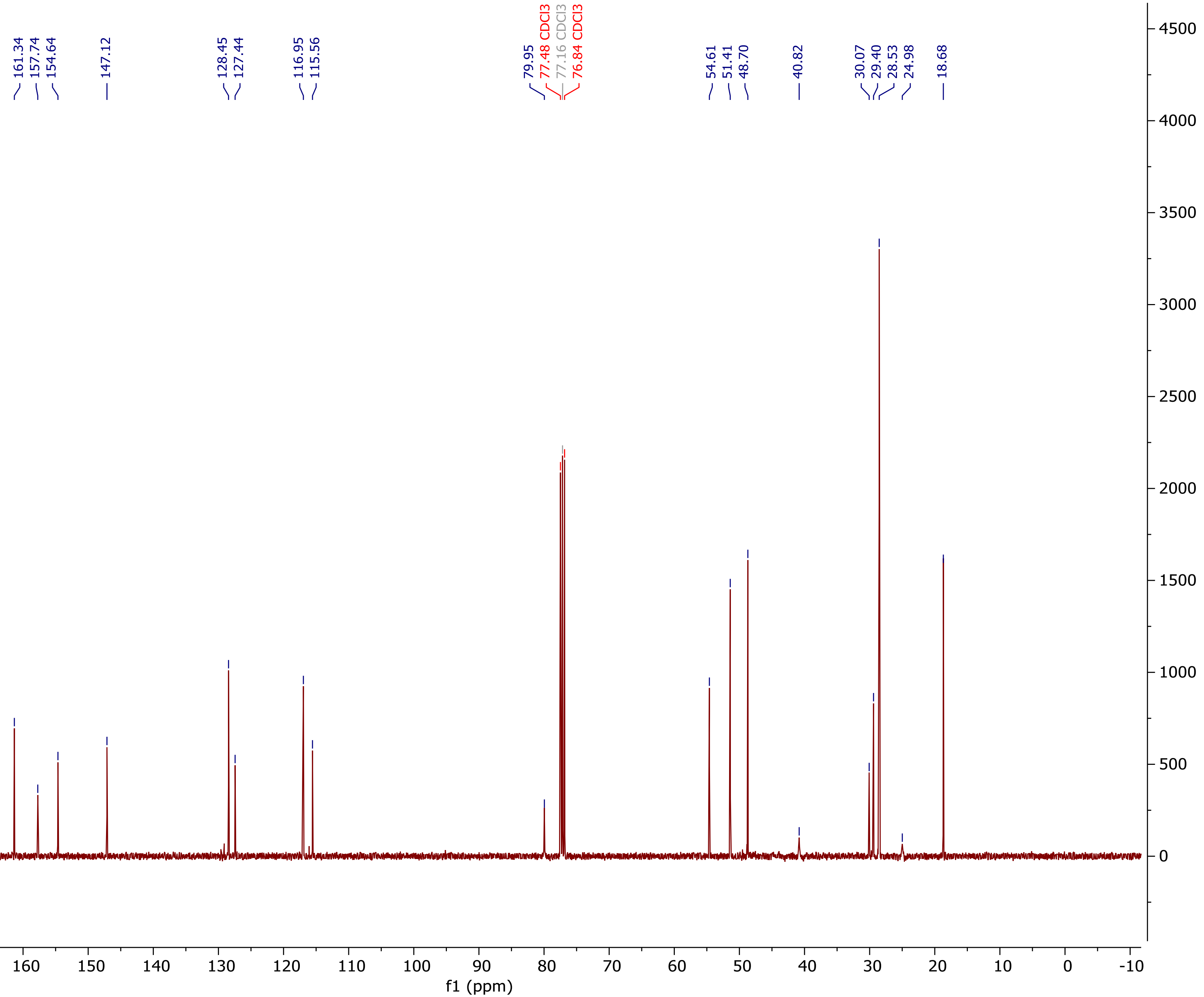

400 MHz, $CDCl_3$

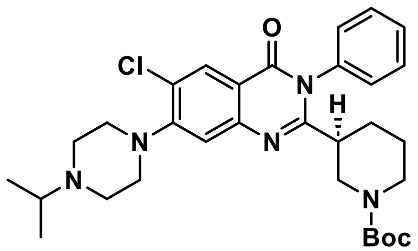

(*S*)-6s

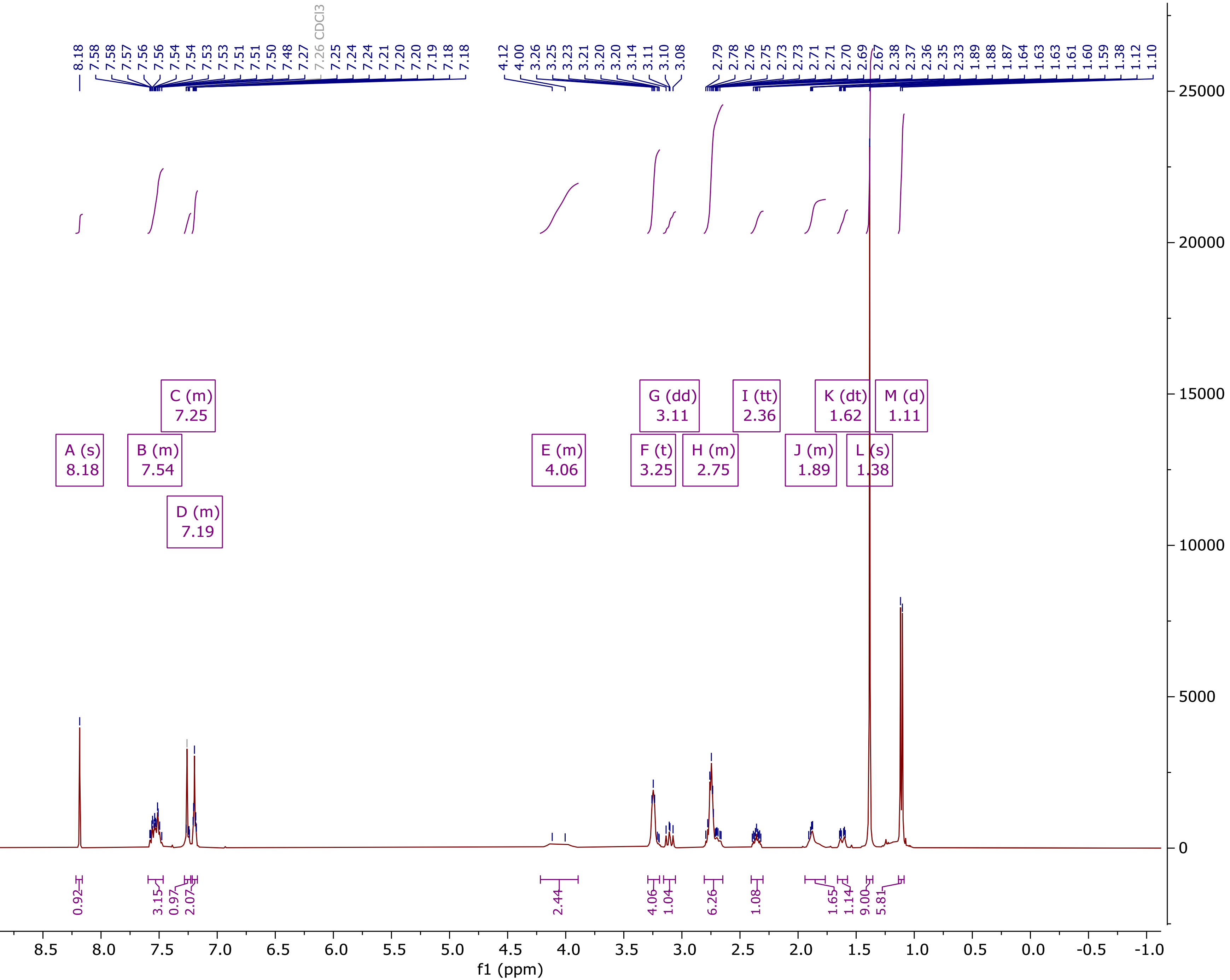

101 MHz, $CDCl_3$

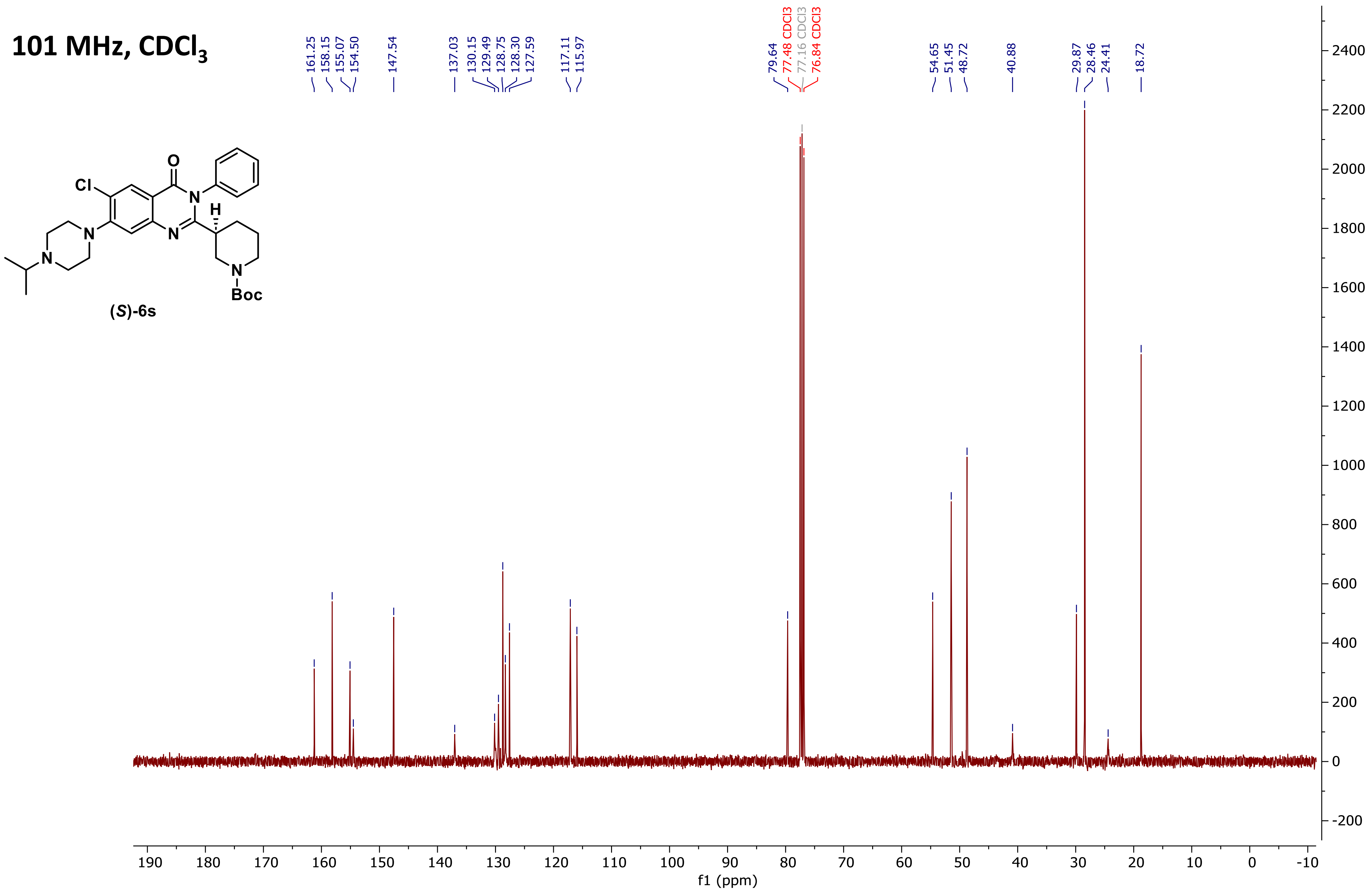

(*S*)-6s

400 MHz, $CDCl_3$

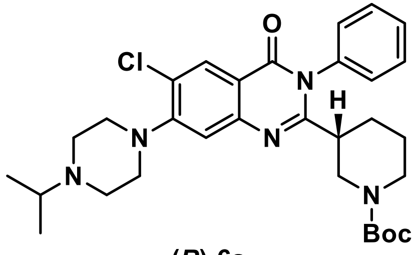

(*R*)-6s

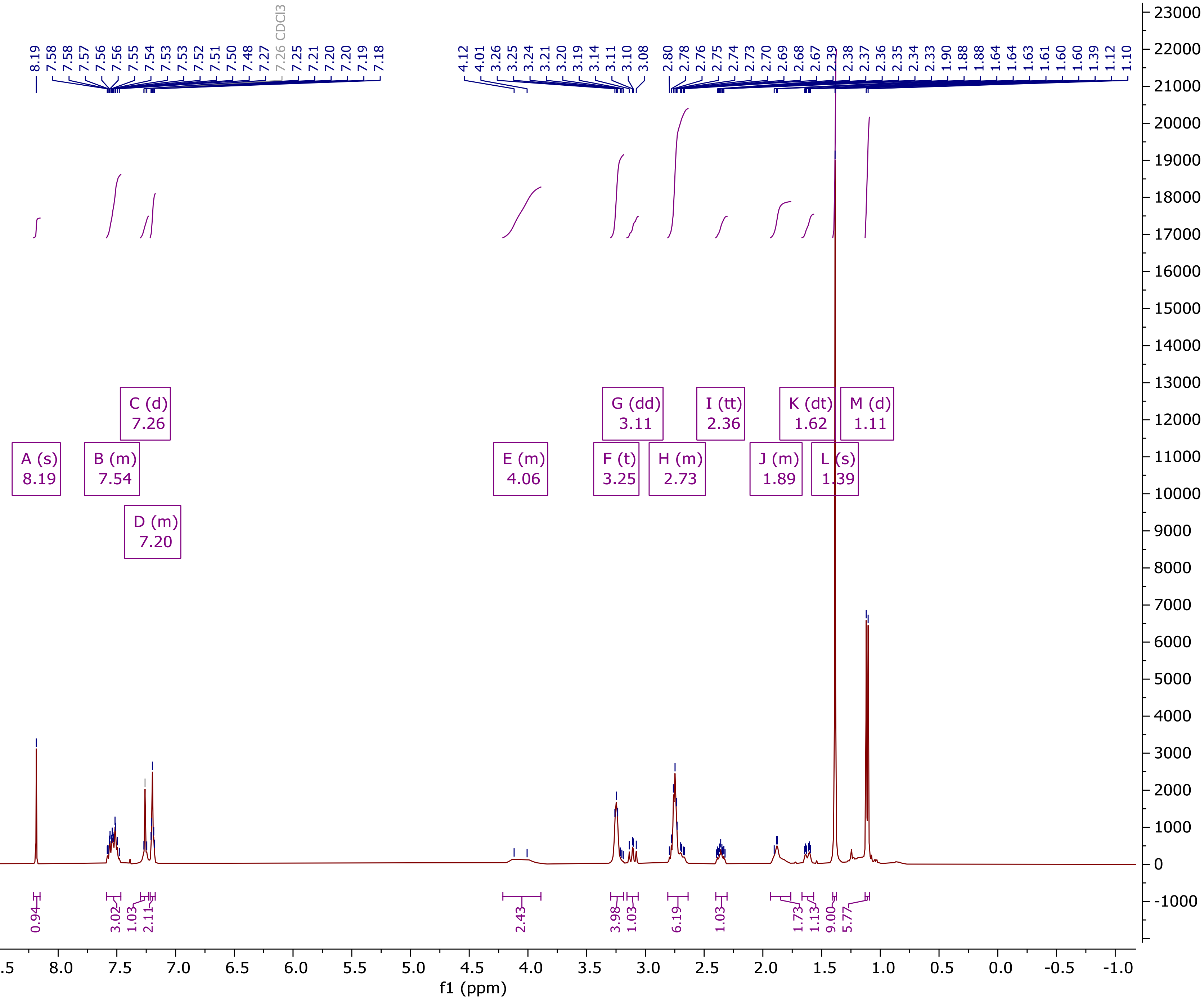

101 MHz, $CDCl_3$

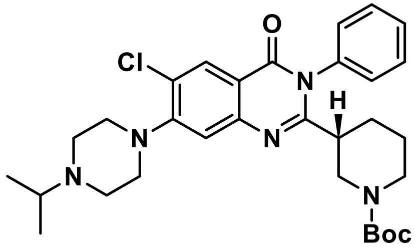

(*R*)-6s

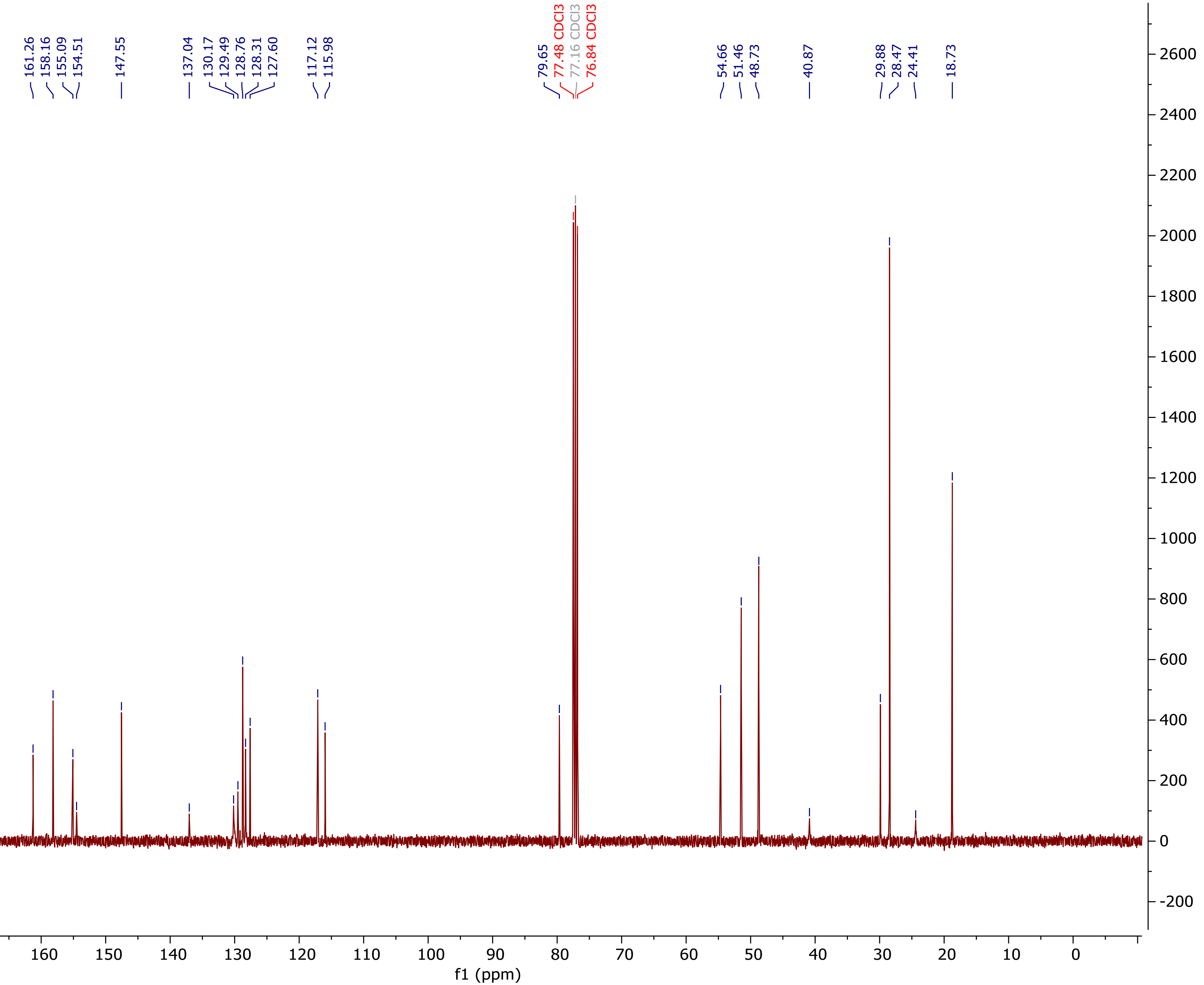

400 MHz, $CDCl_3$

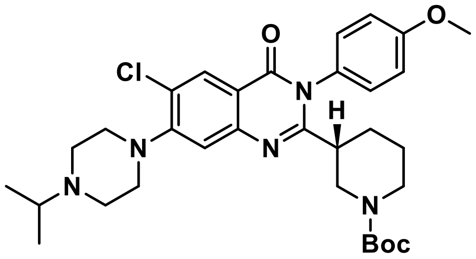

(*R*)-6t

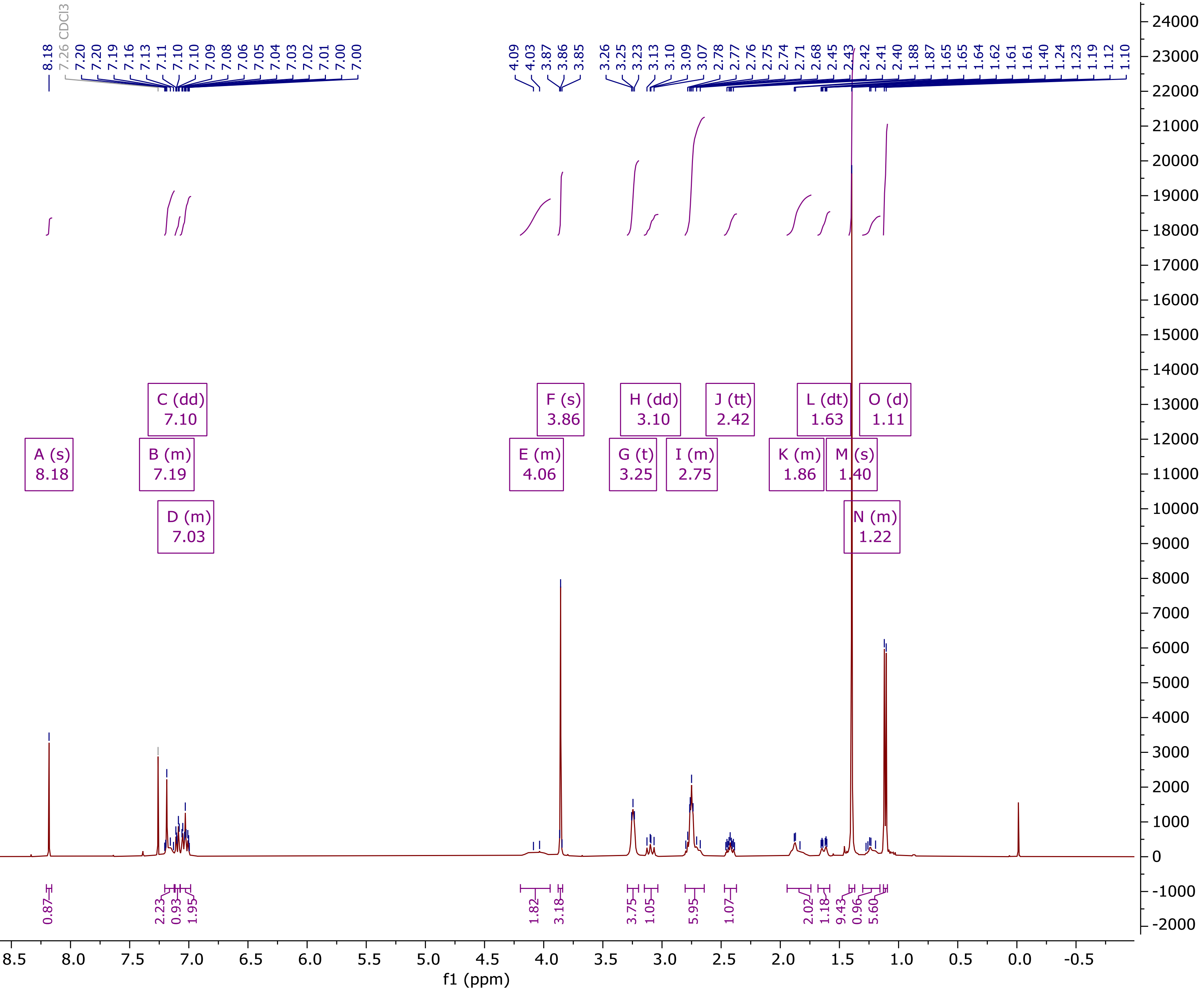

101 MHz, $CDCl_3$

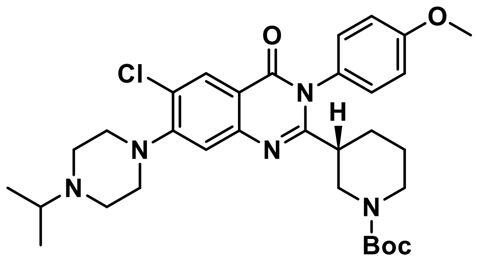

(*R*)-6t

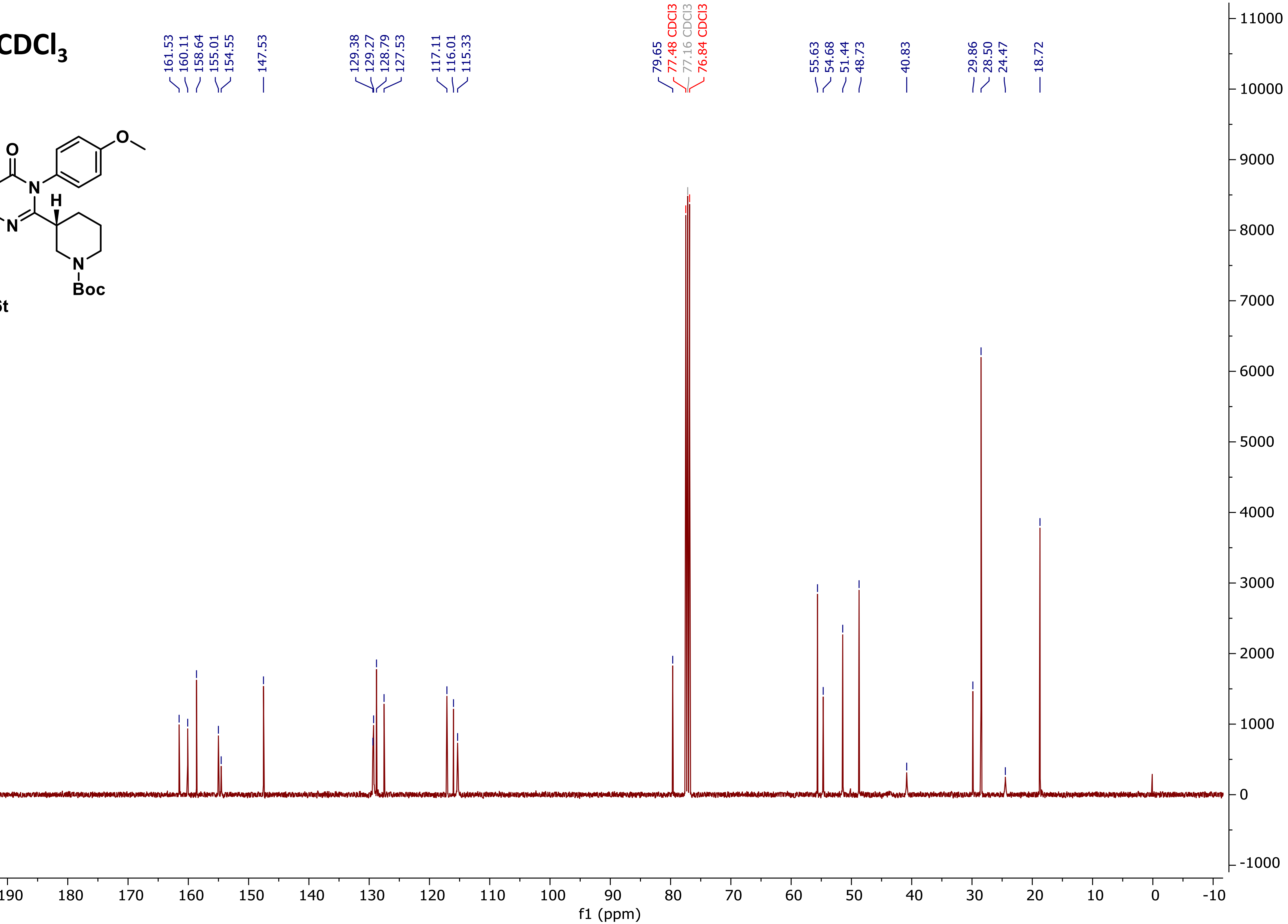

400 MHz, $CDCl_3$

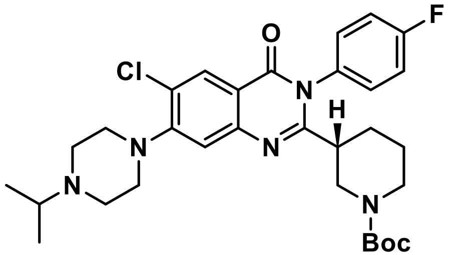

(*R*)-6u

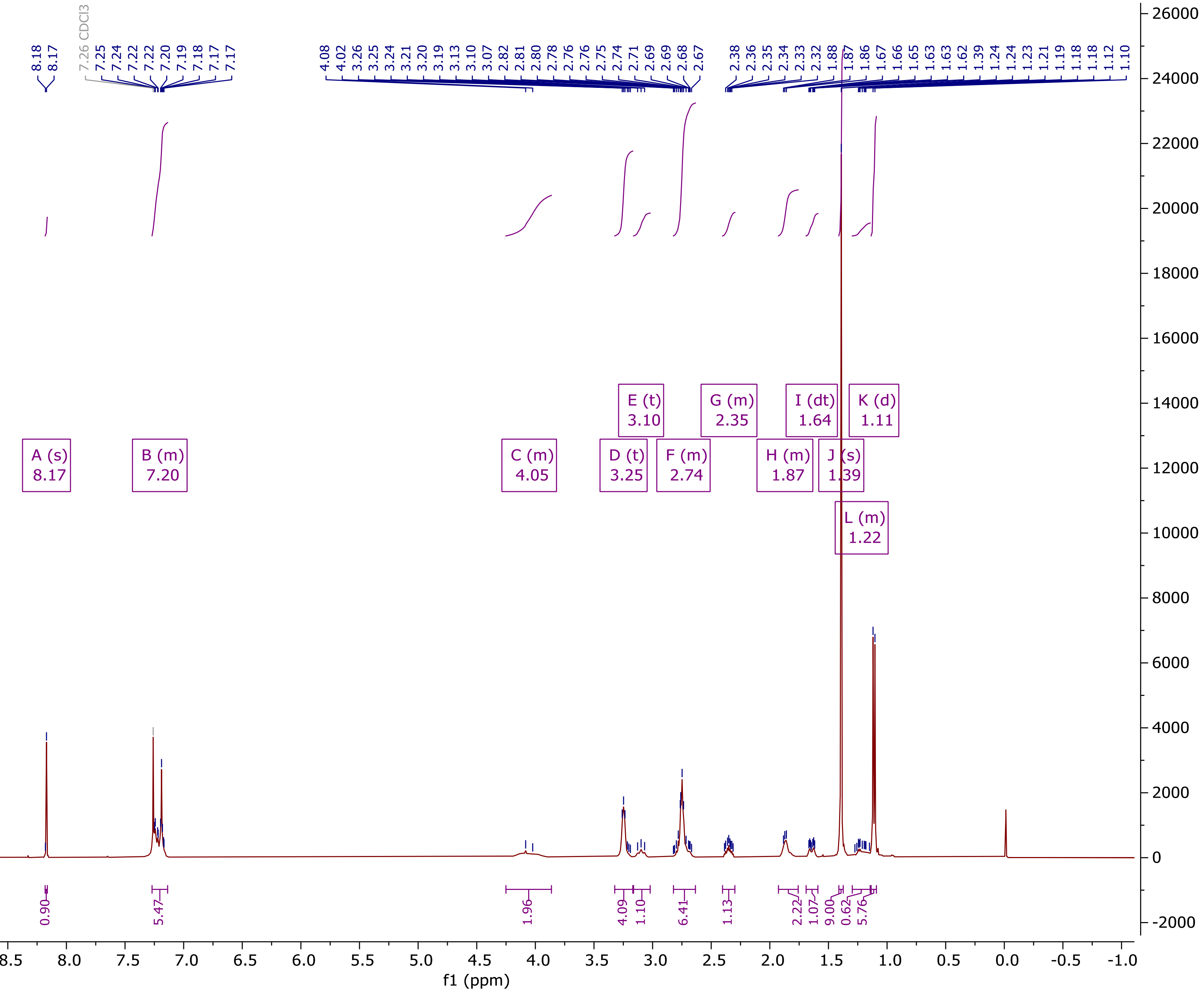

101 MHz, $CDCl_3$

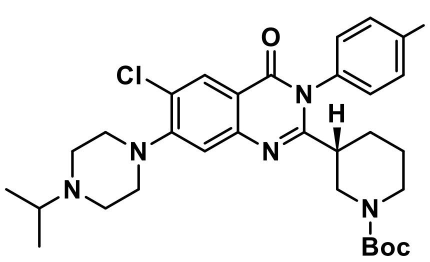

(*R*)-6u

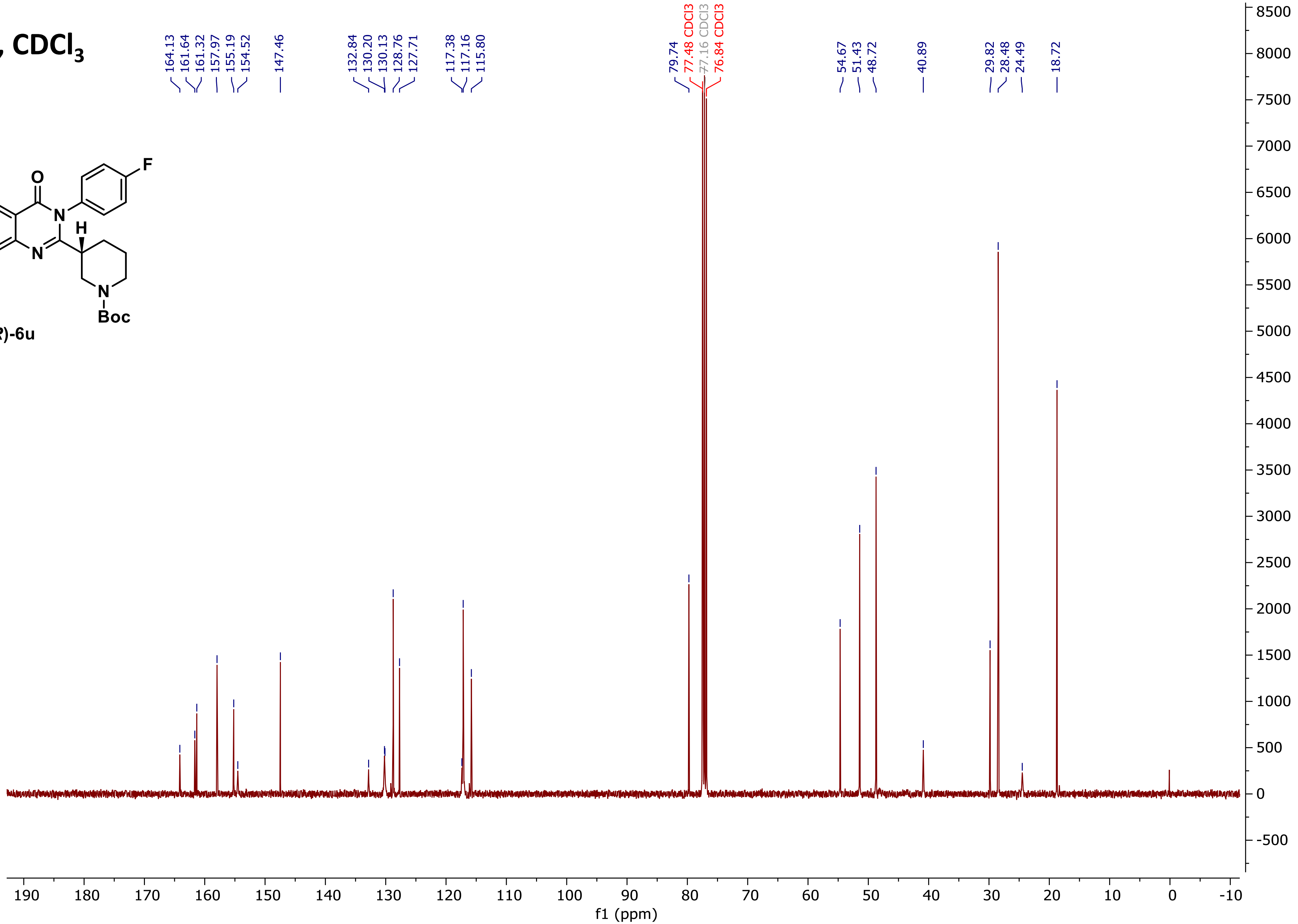

400 MHz, $CDCl_3$

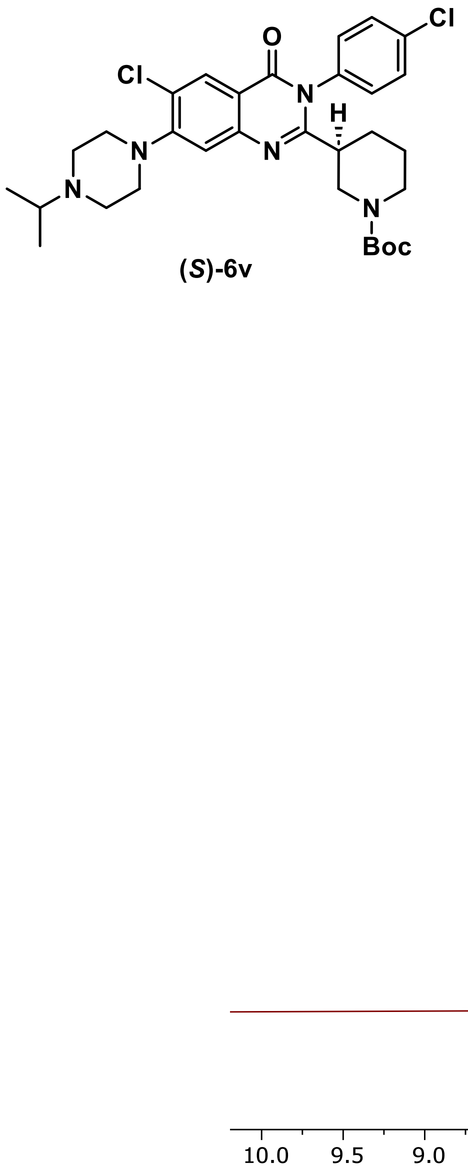

(*S*)-6v

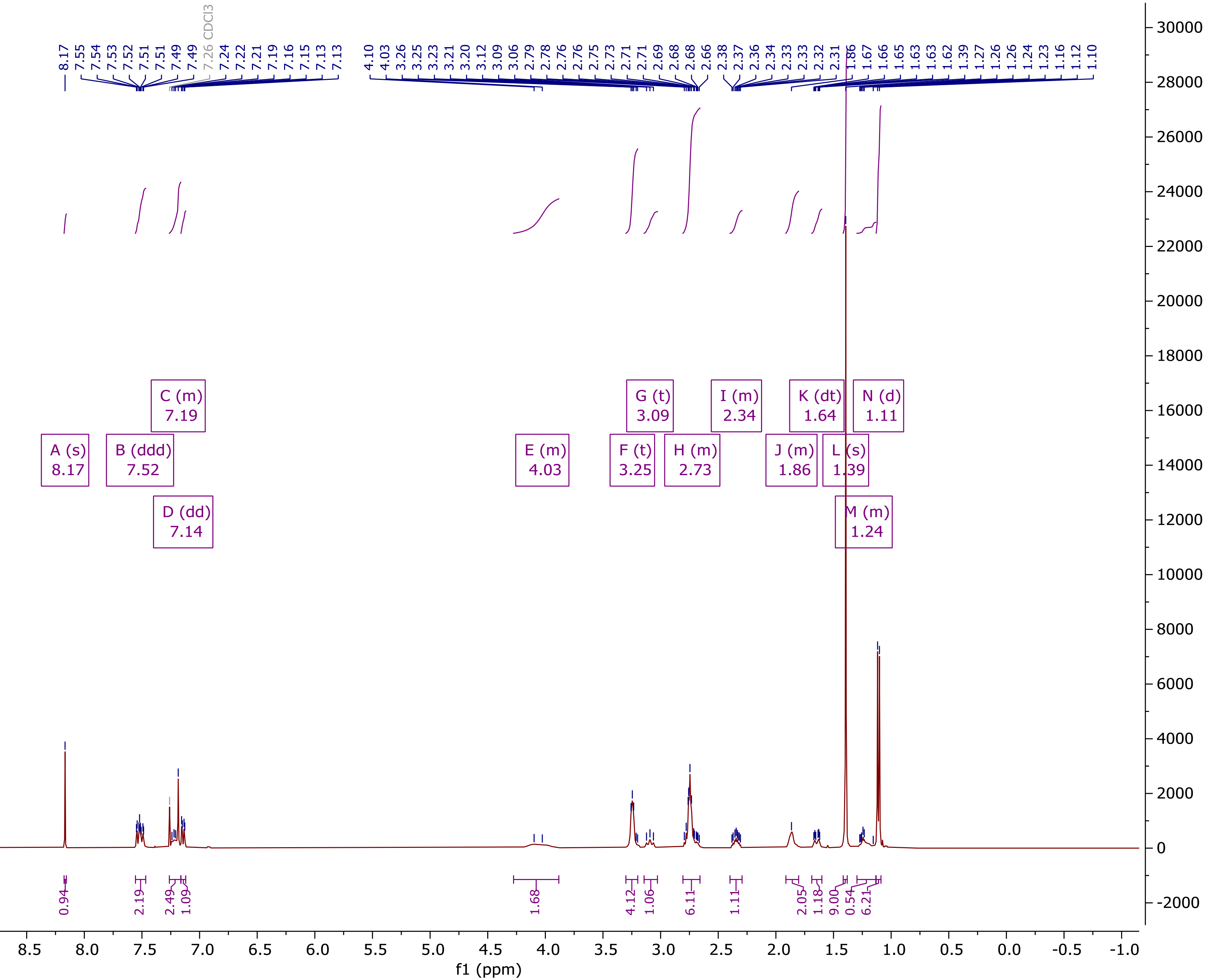

101 MHz, $CDCl_3$

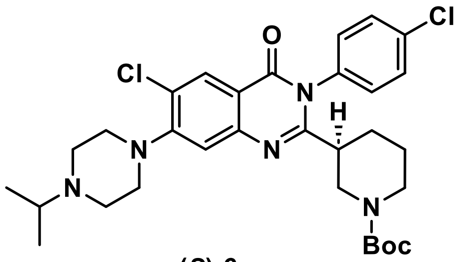

(*S*)-6v

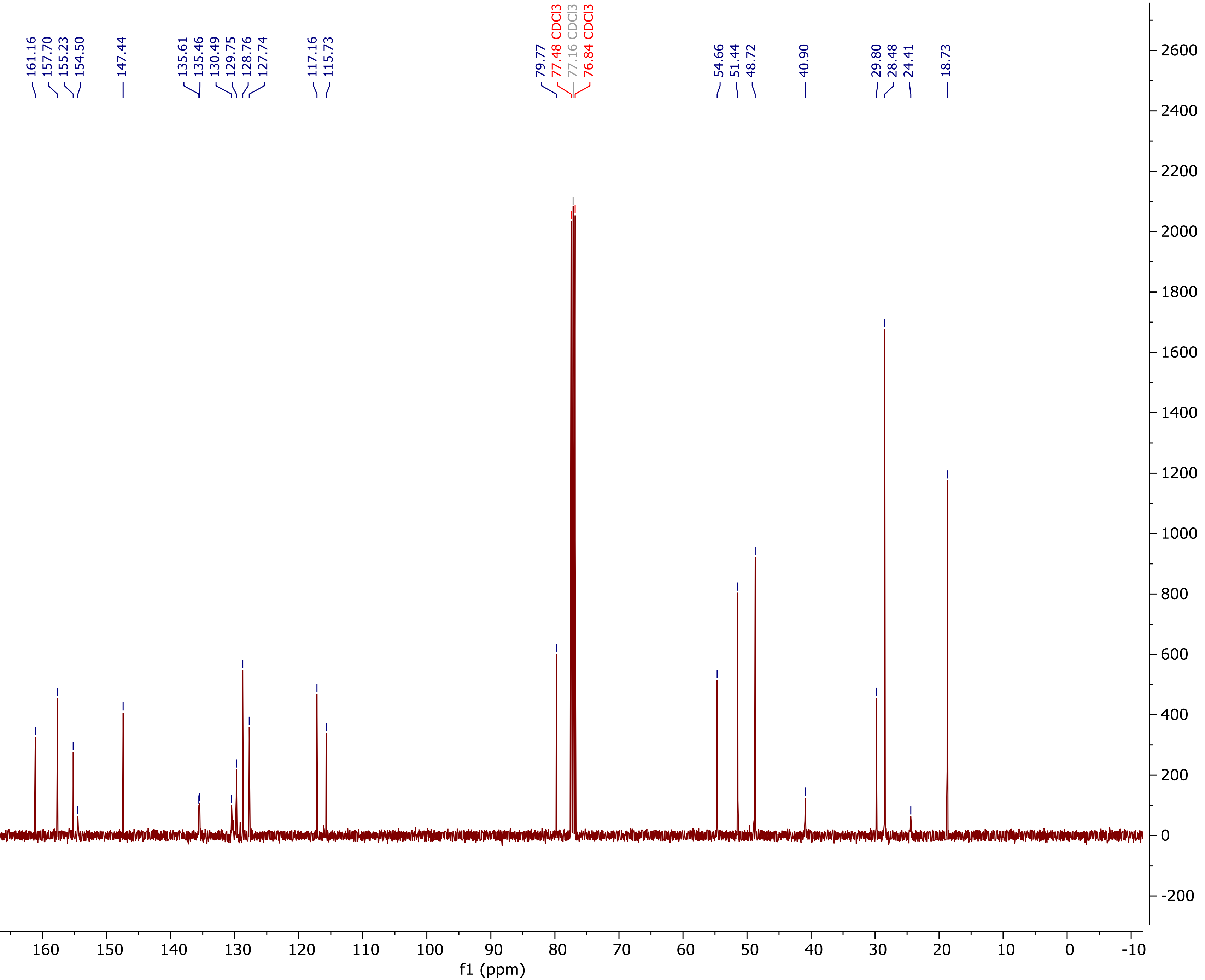

400 MHz, $CDCl_3$

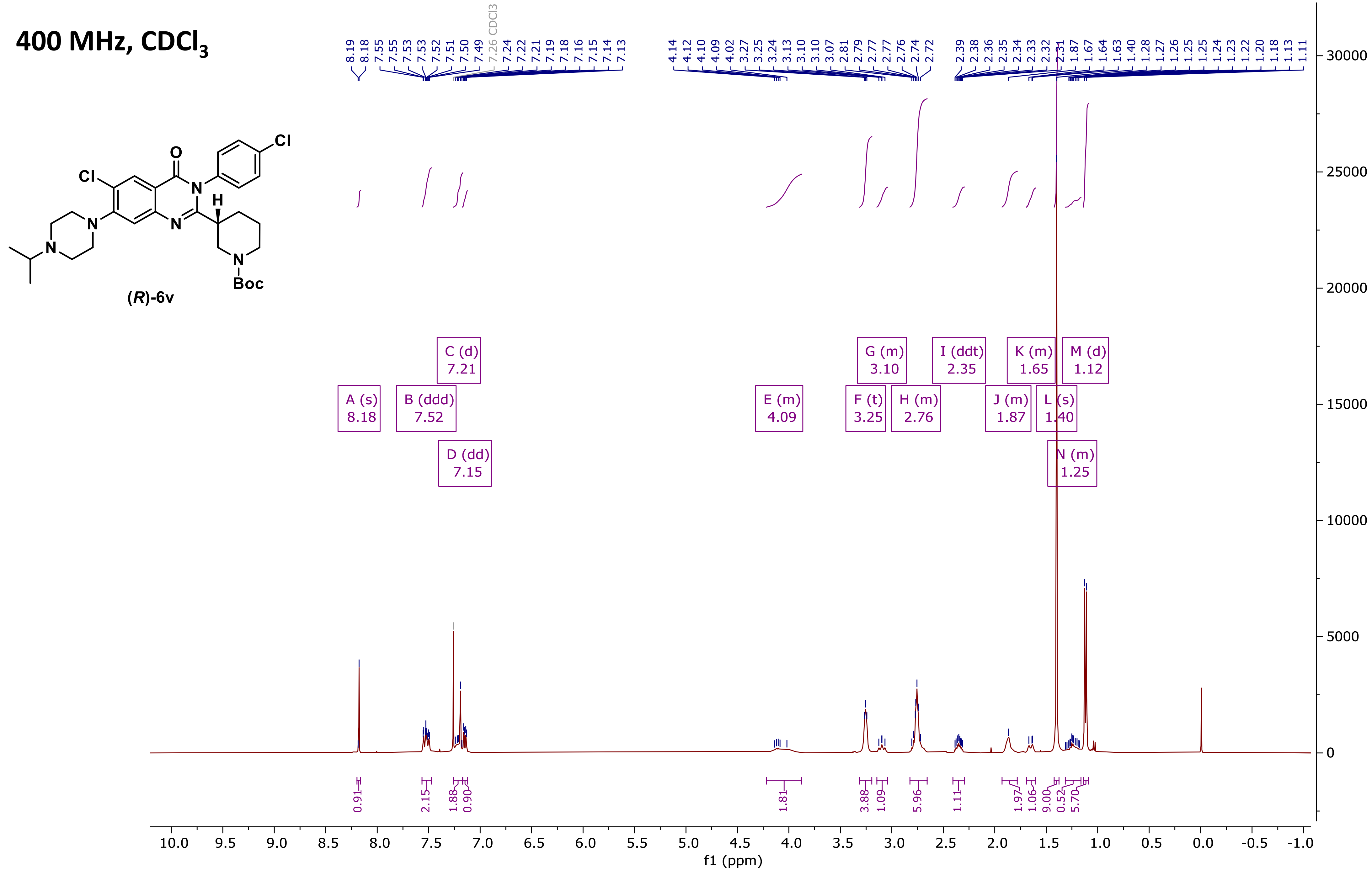

101 MHz, $CDCl_3$

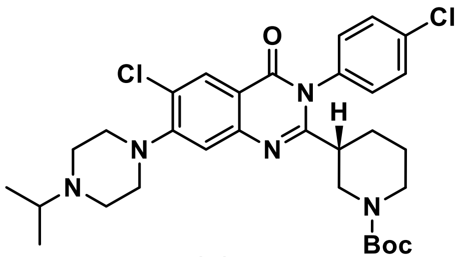

(*R*)-6v

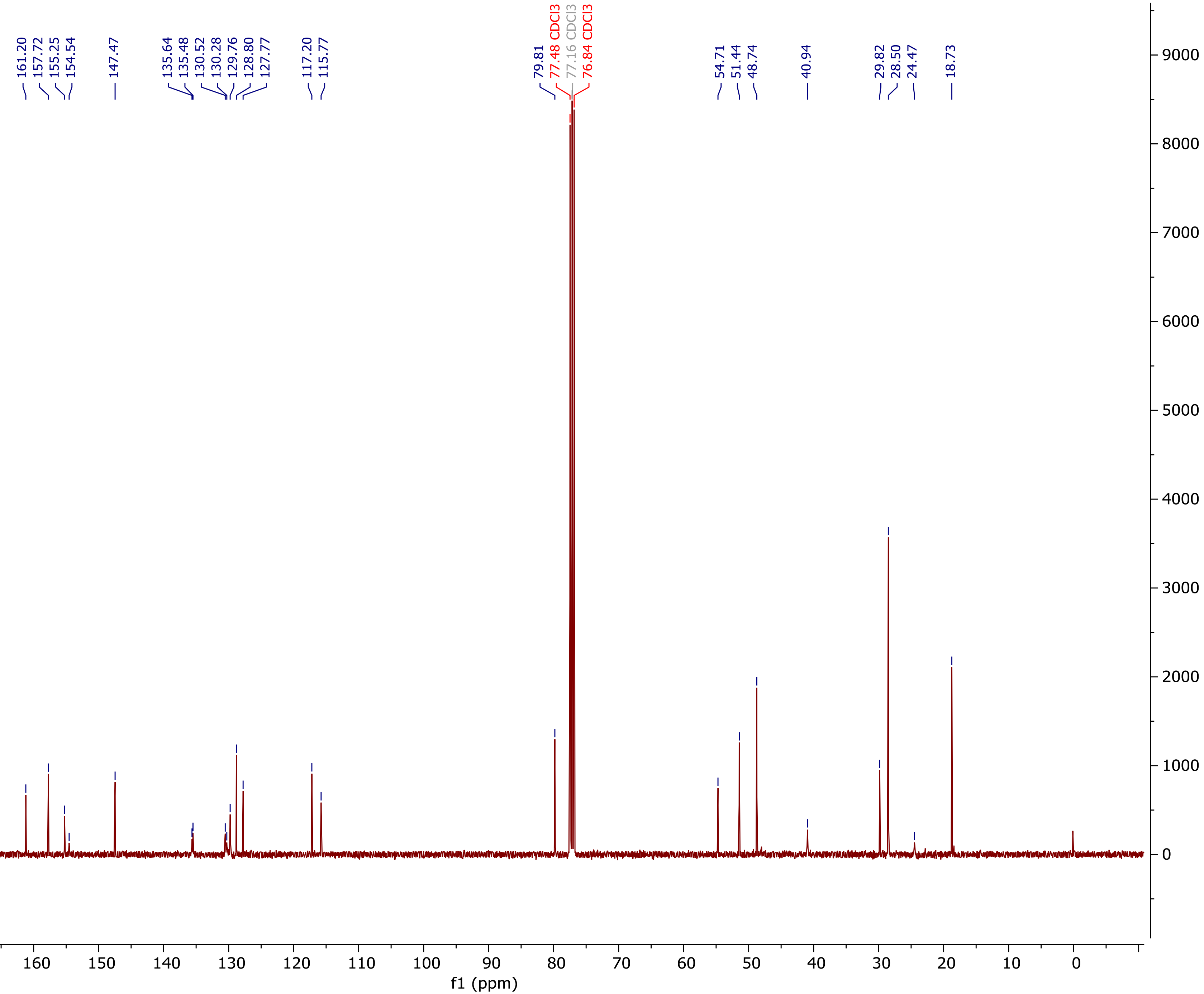

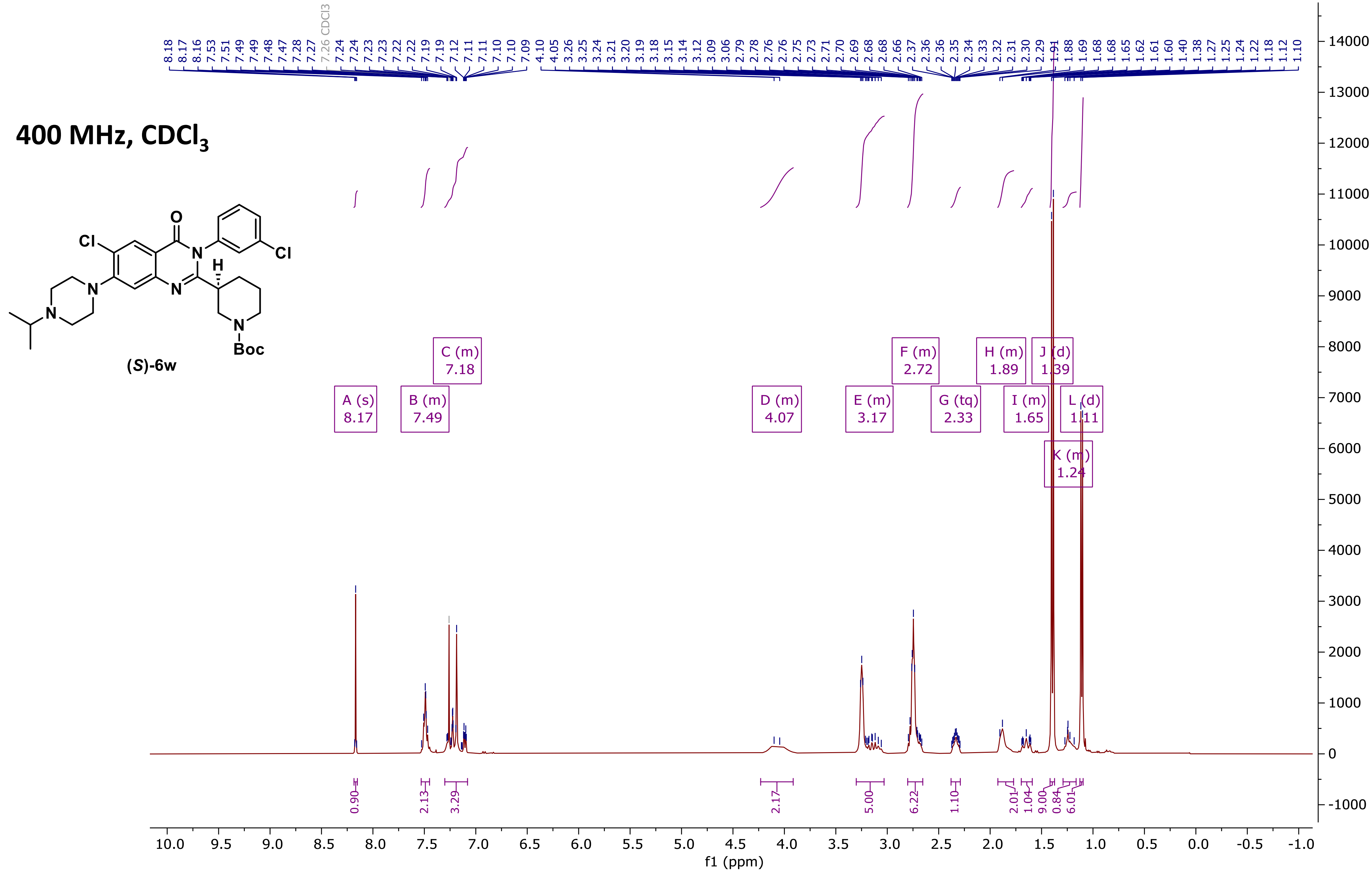
400 MHz, CDCl3
(S)-6w
A (s) 8.17
B (m) 7.49
C (m) 7.18
D (m) 4.07
E (m) 3.17
F (m) 2.72
G (tq) 2.33
H (m) 1.89
I (m) 1.65
J (d) 1.39
K (m) 1.24
L (d) 1.11
f1 (ppm)

101 MHz, $CDCl_3$

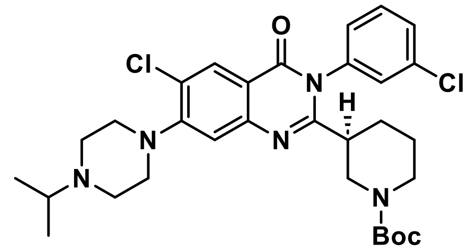

(*S*)-6w

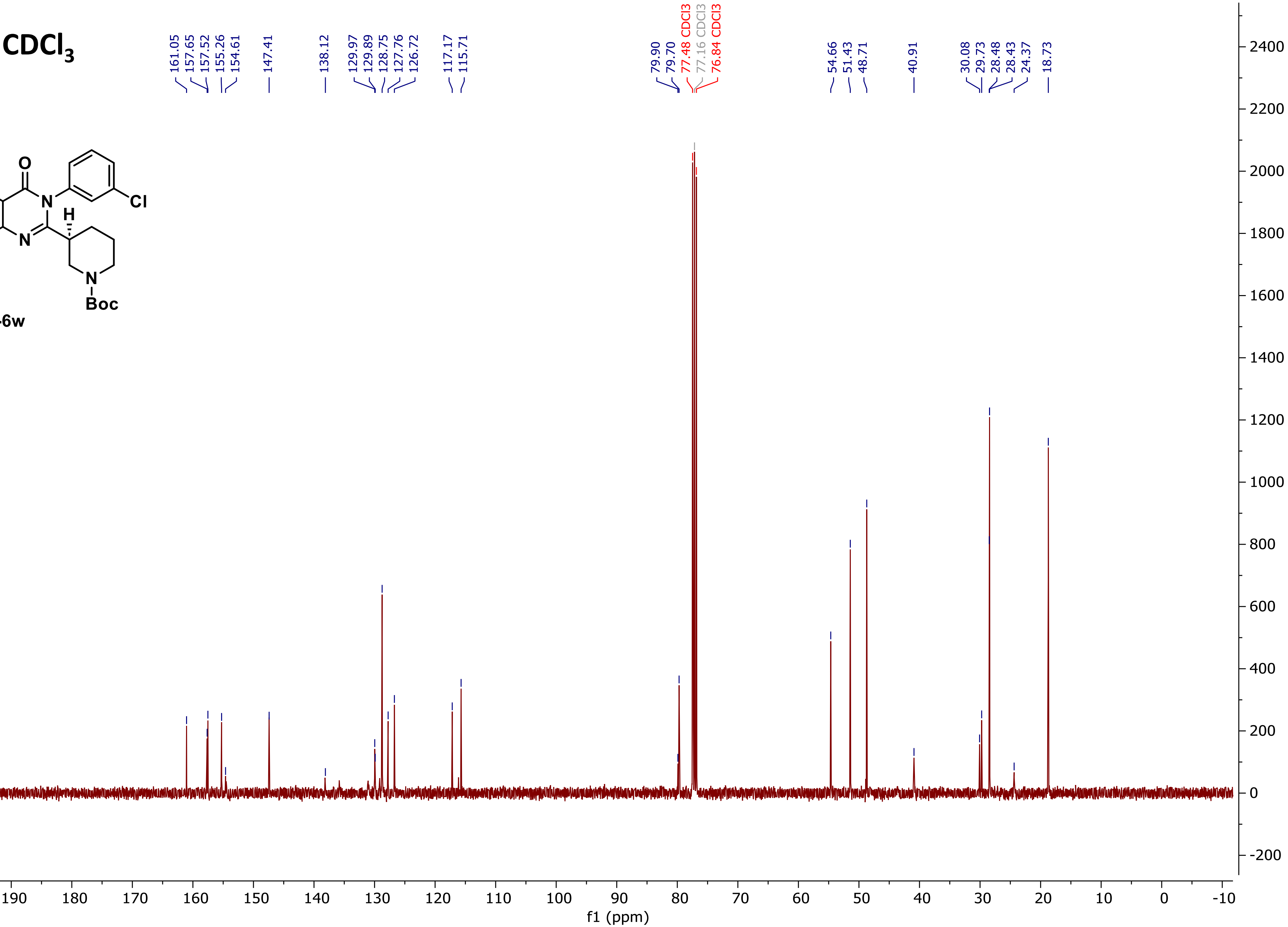

400 MHz, $CDCl_3$

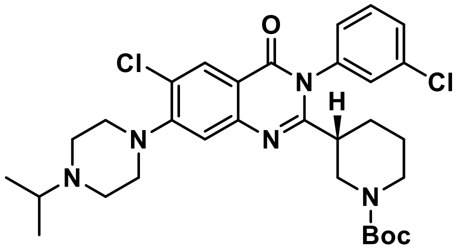

(*R*)-6w

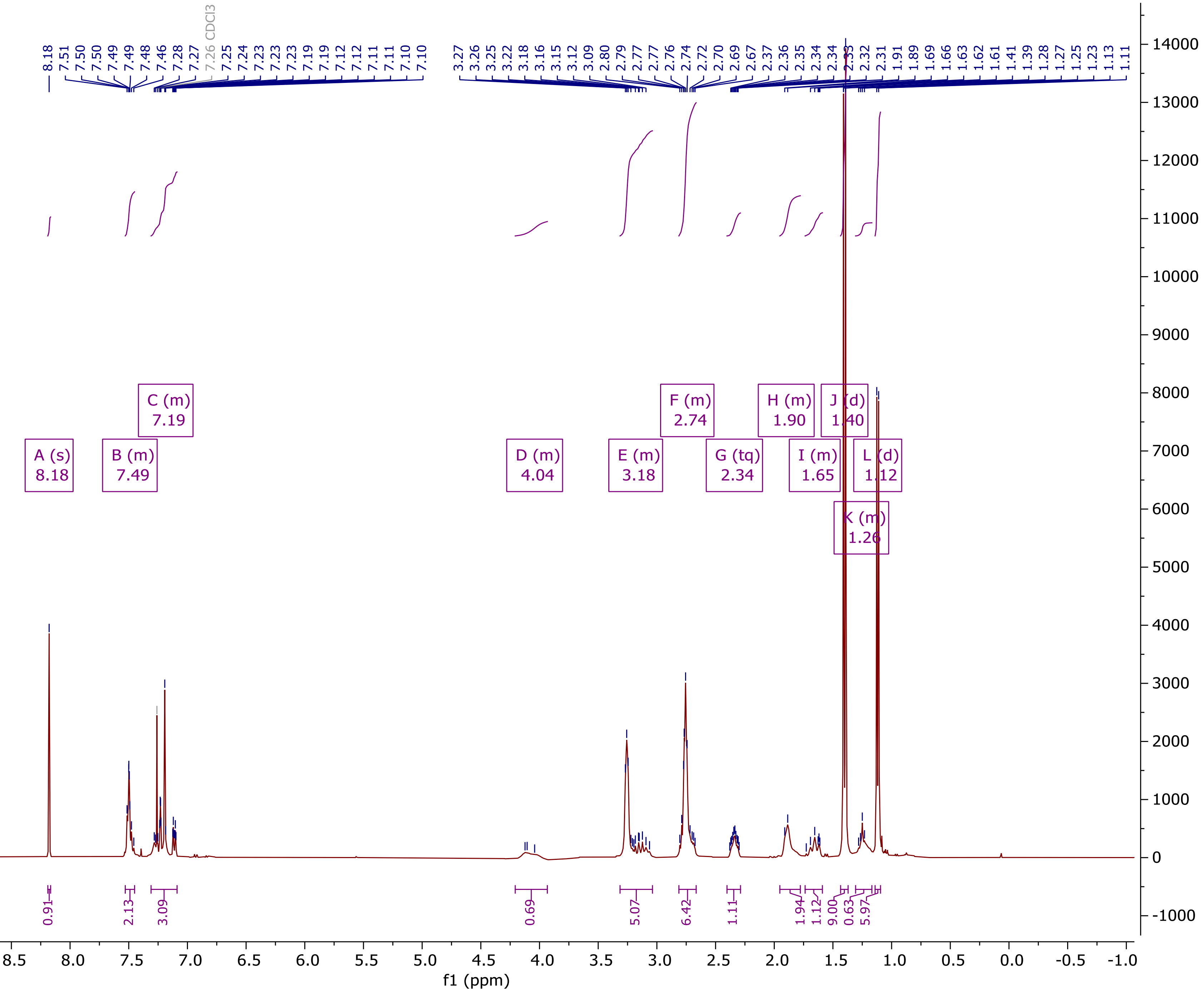

101 MHz, $CDCl_3$

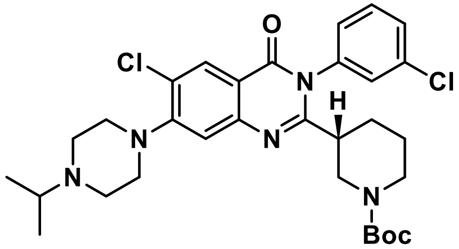

(*R*)-6w

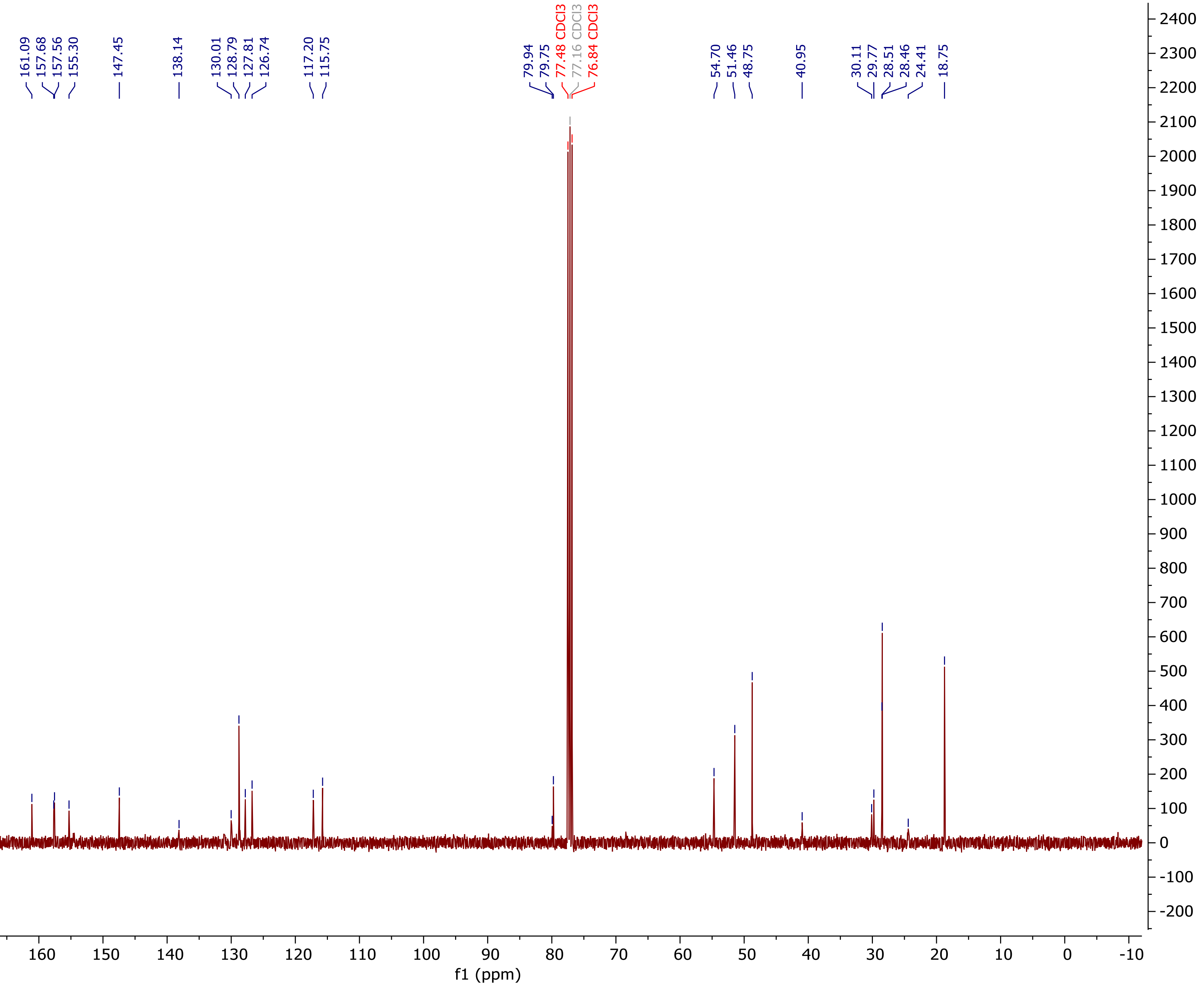

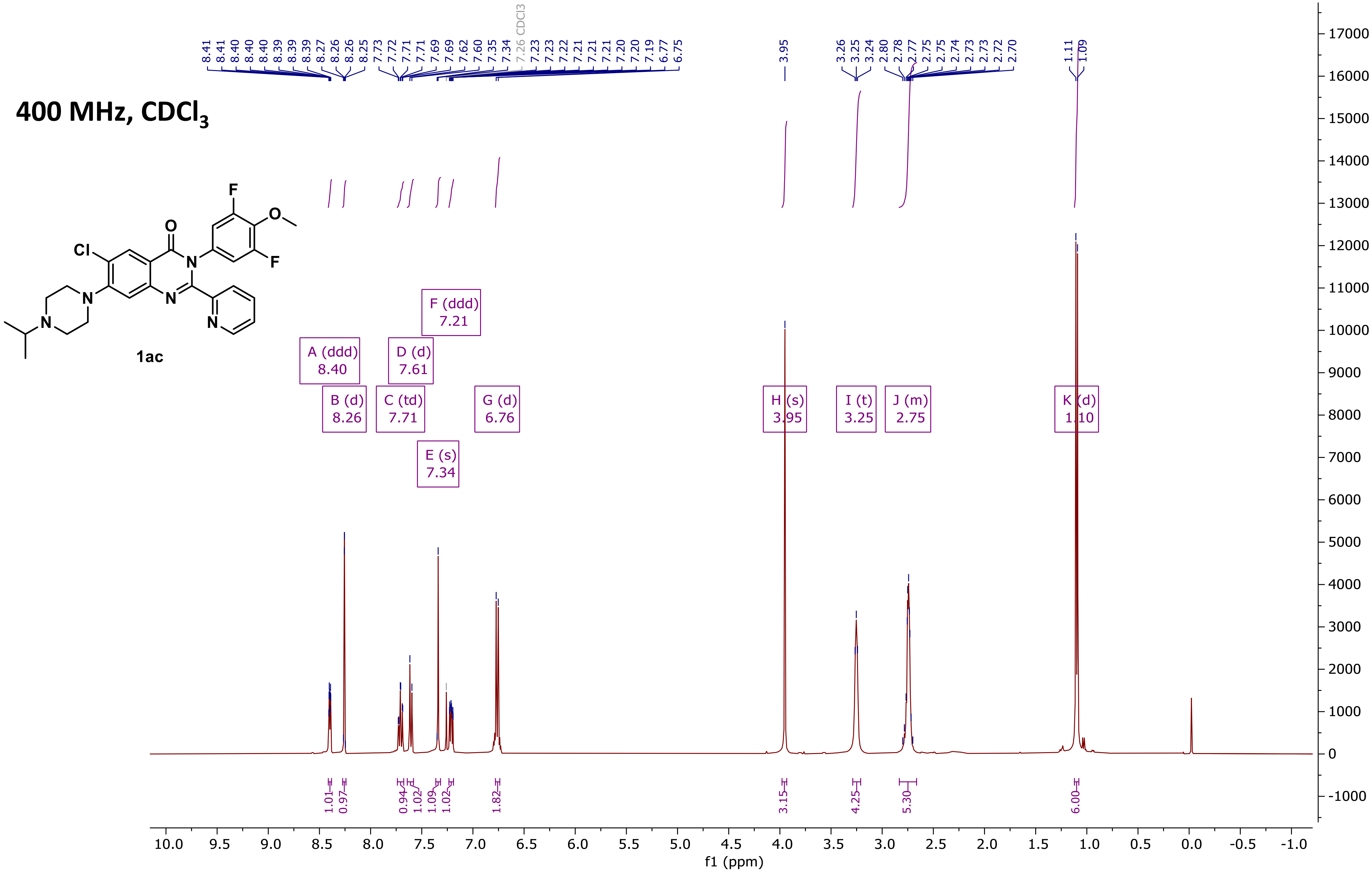

400 MHz, CDCl3
1ac
A (ddd) 8.40
B (d) 8.26
C (td) 7.71
D (d) 7.61
E (s) 7.34
F (ddd) 7.21
G (d) 6.76
H (s) 3.95
I (t) 3.25
J (m) 2.75
K (d) 1.10
f1 (ppm)

101 MHz, $CDCl_3$

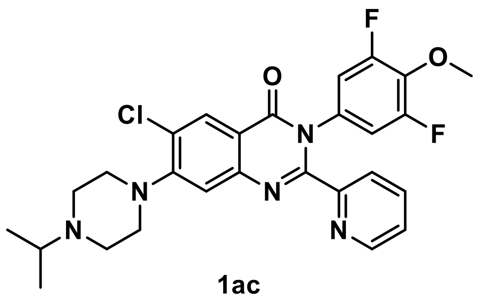

1ac

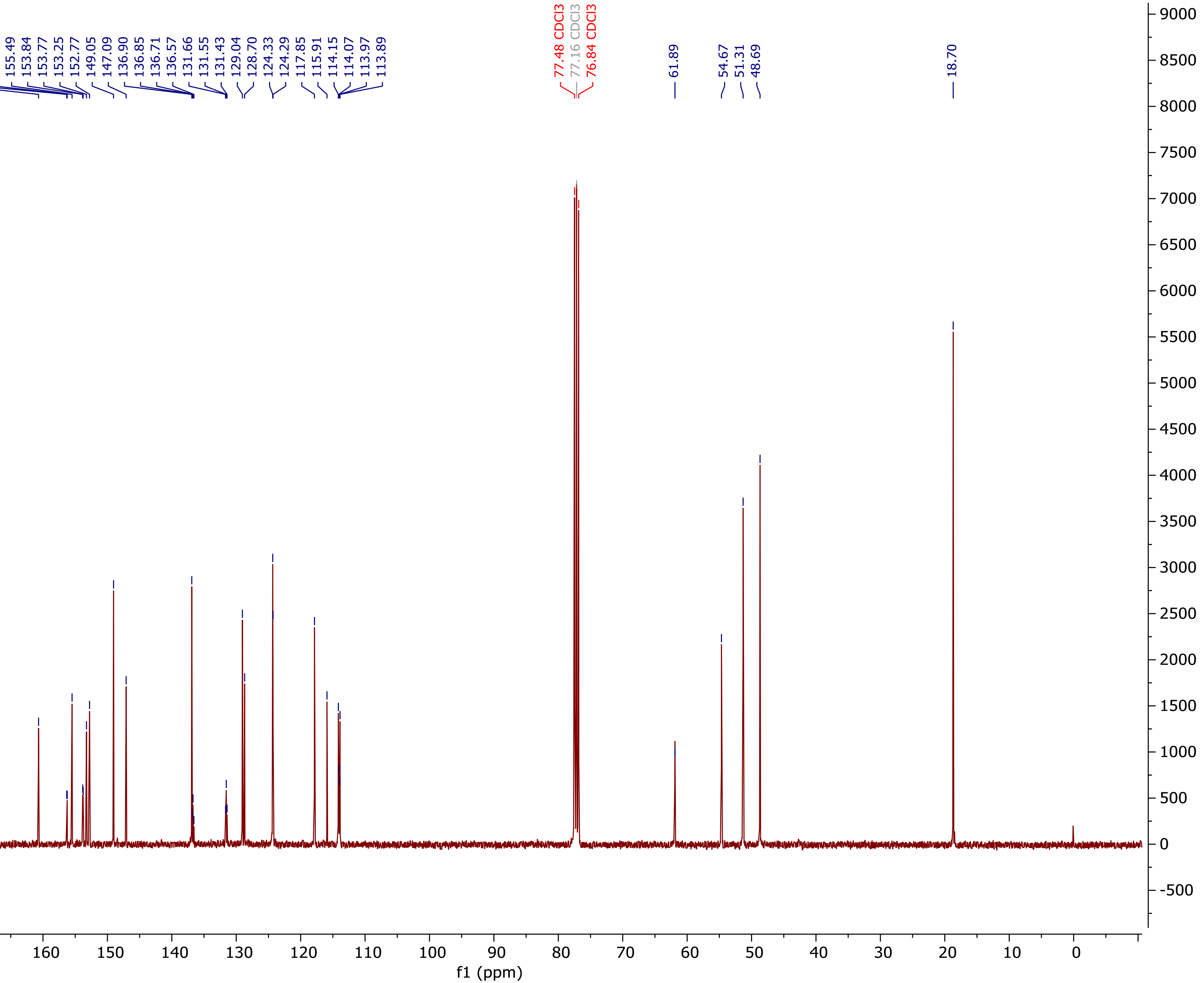

400 MHz, CDCl$_3$

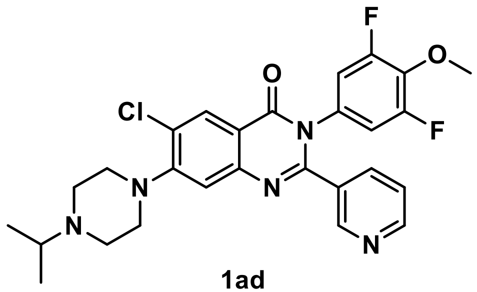

1ad

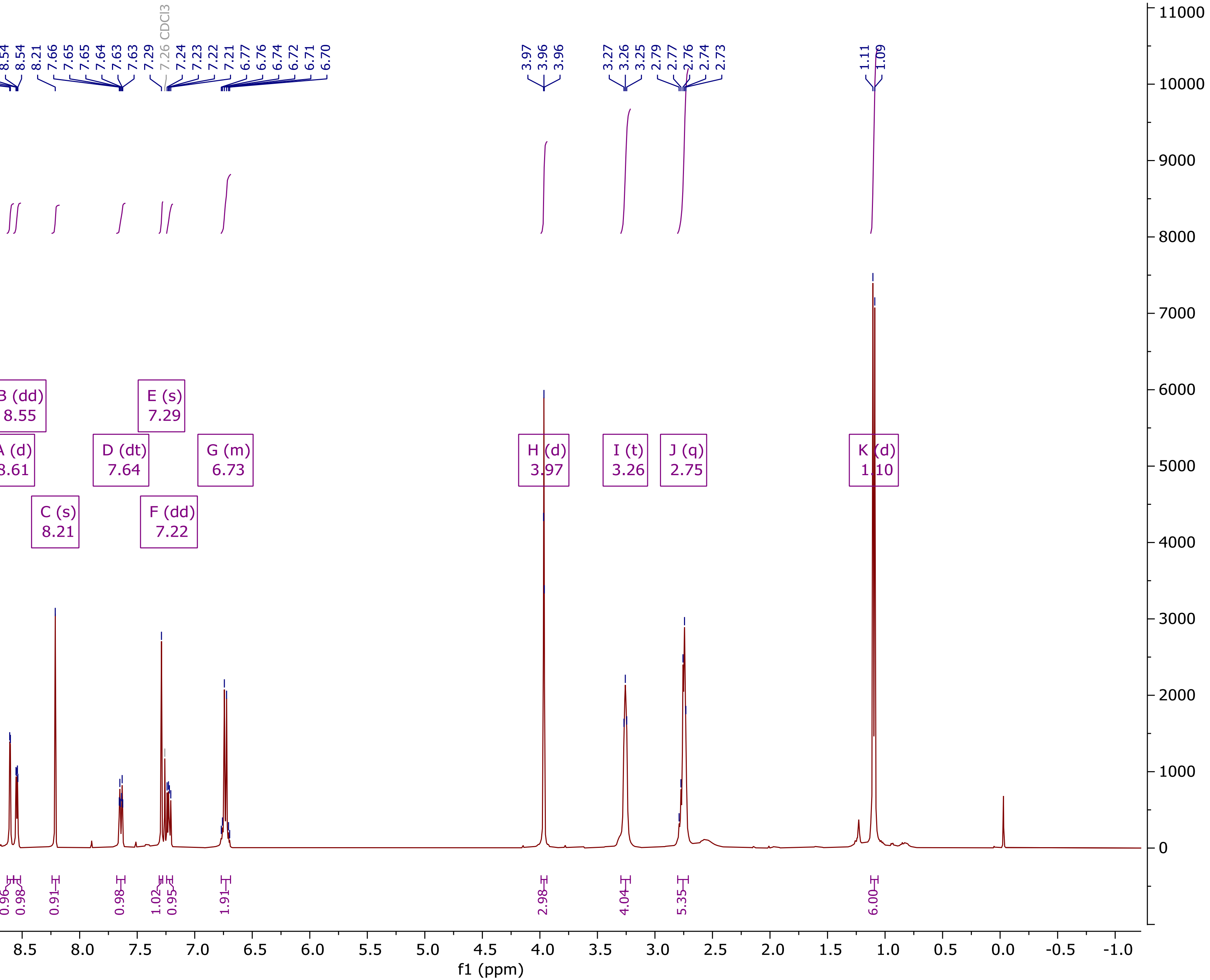

101 MHz, $CDCl_3$

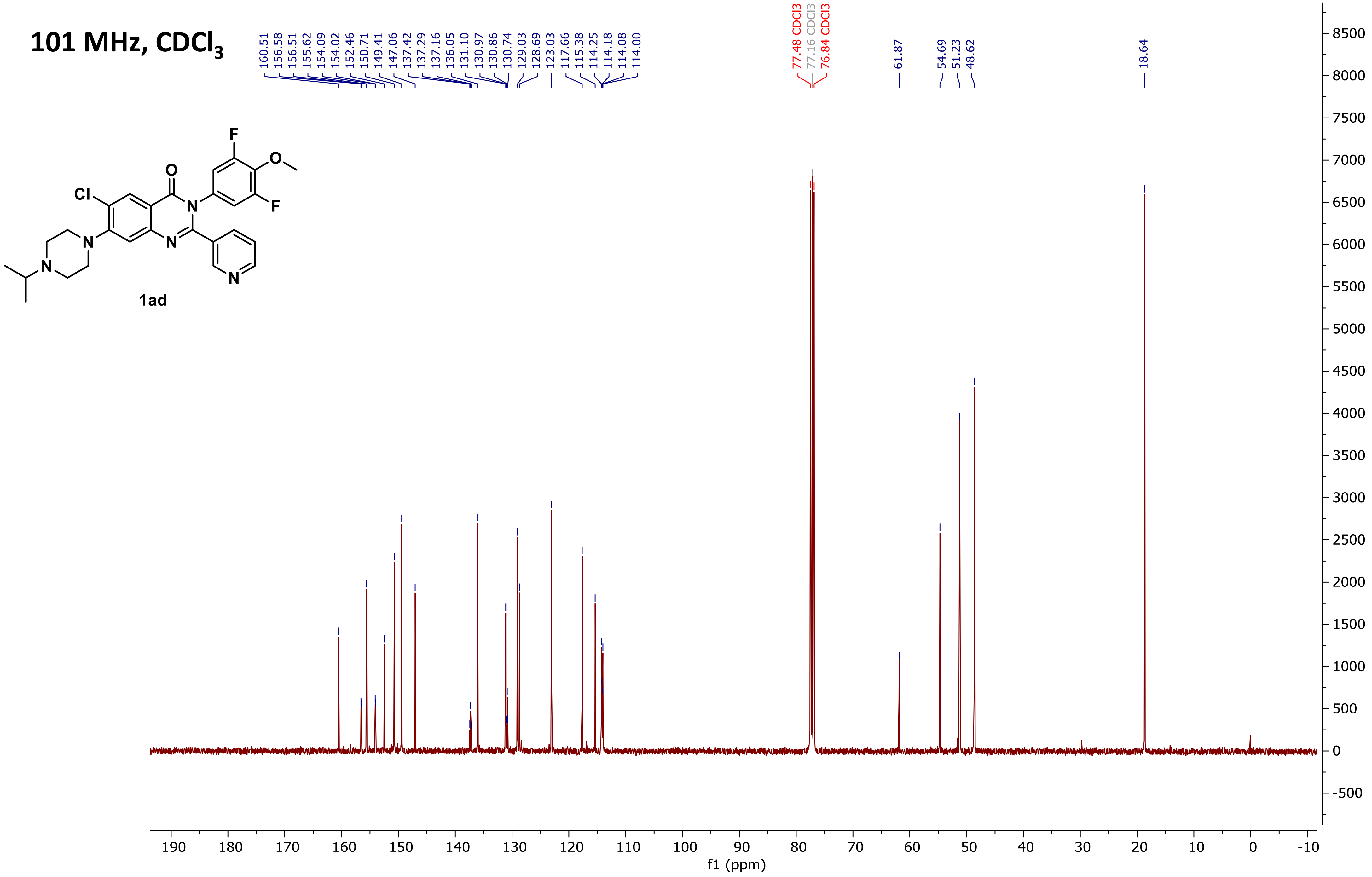

1ad

376 MHz, Methanol-d_4

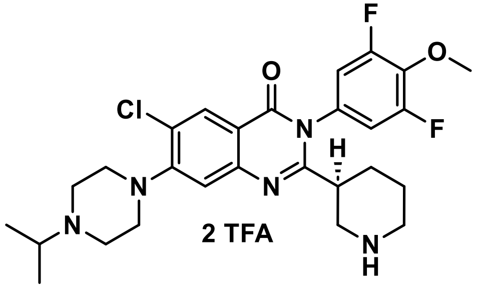

(*S*)-7g

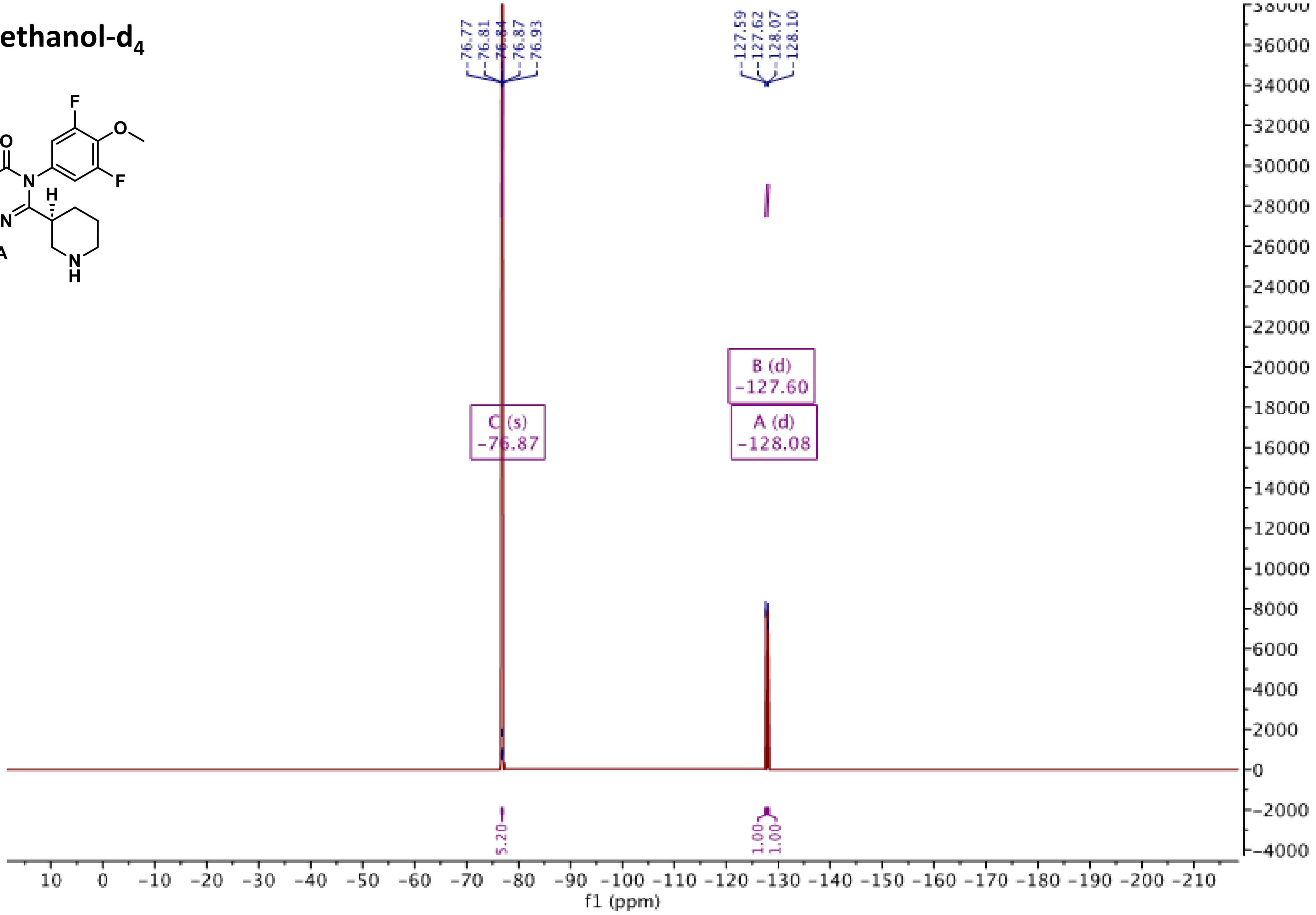

101 MHz, Methanol-d_4

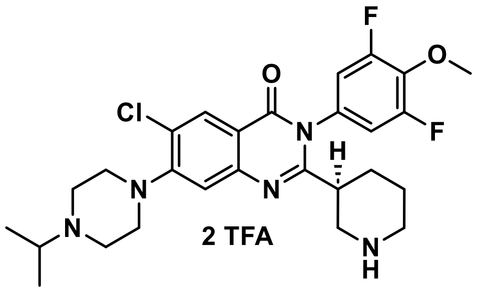

(*S*)-7g

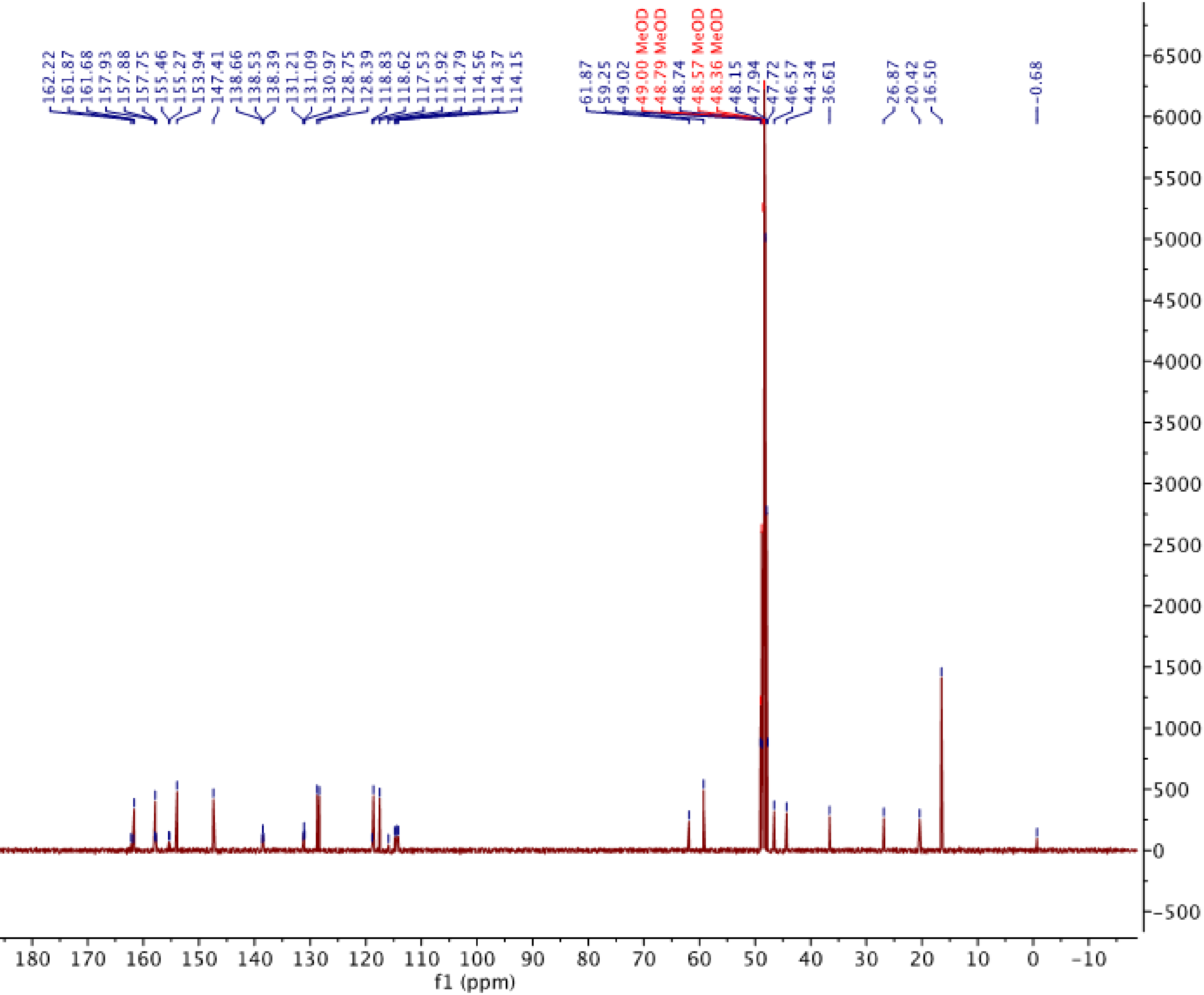

400 MHz, Methanol-d_4

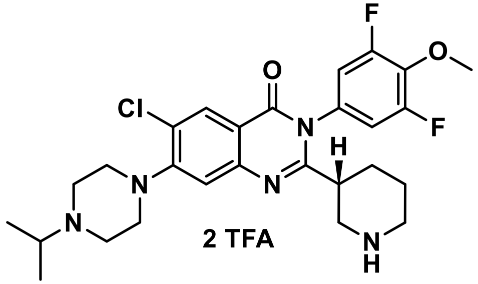

(*R*)-7g

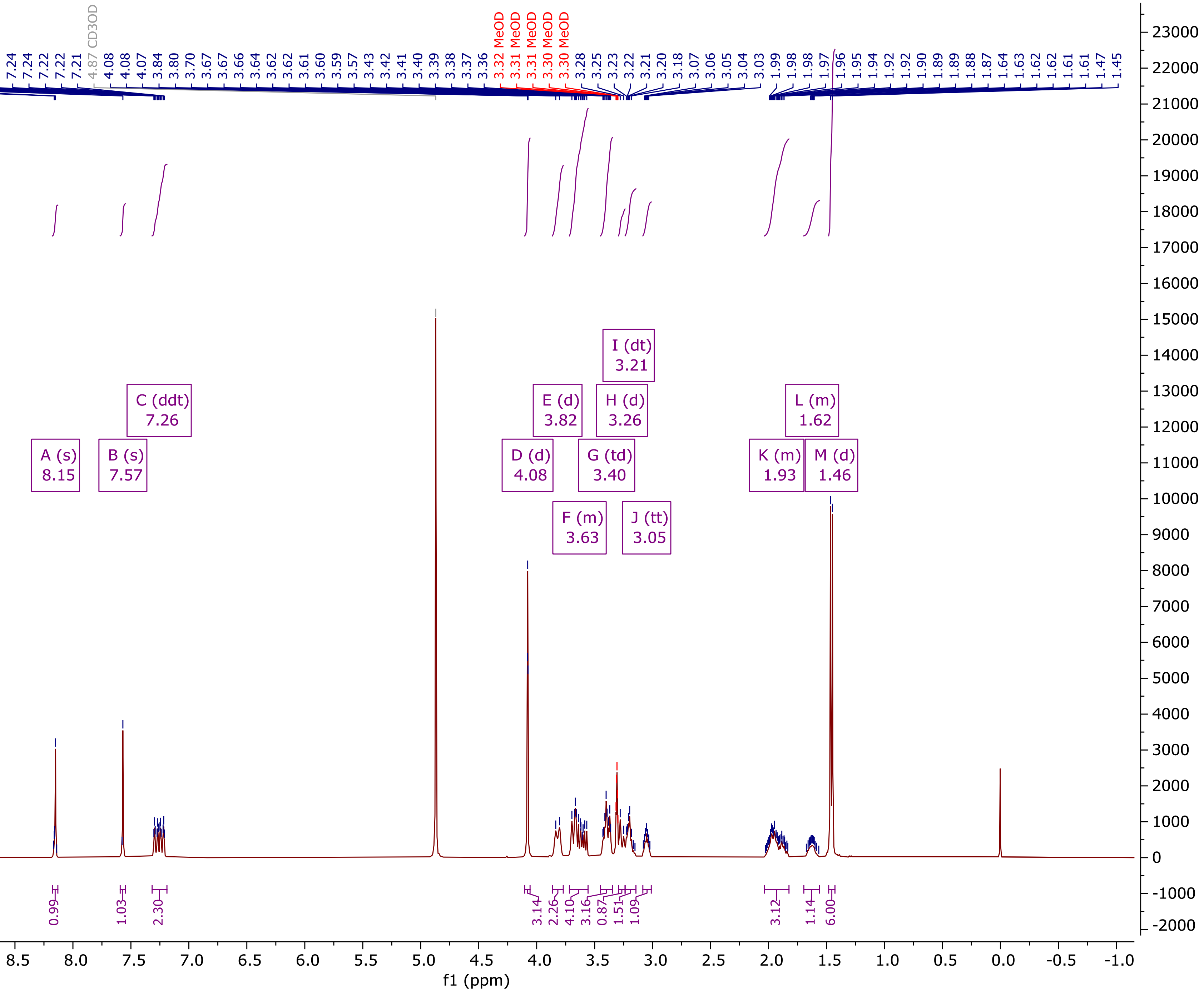

376 MHz, Methanol-d_4

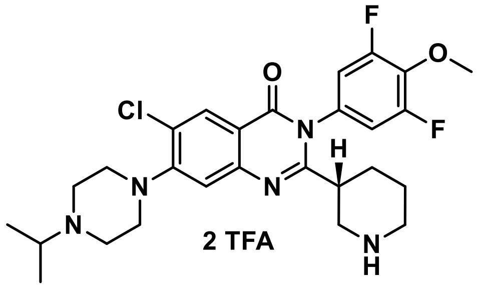

(*R*)-7g

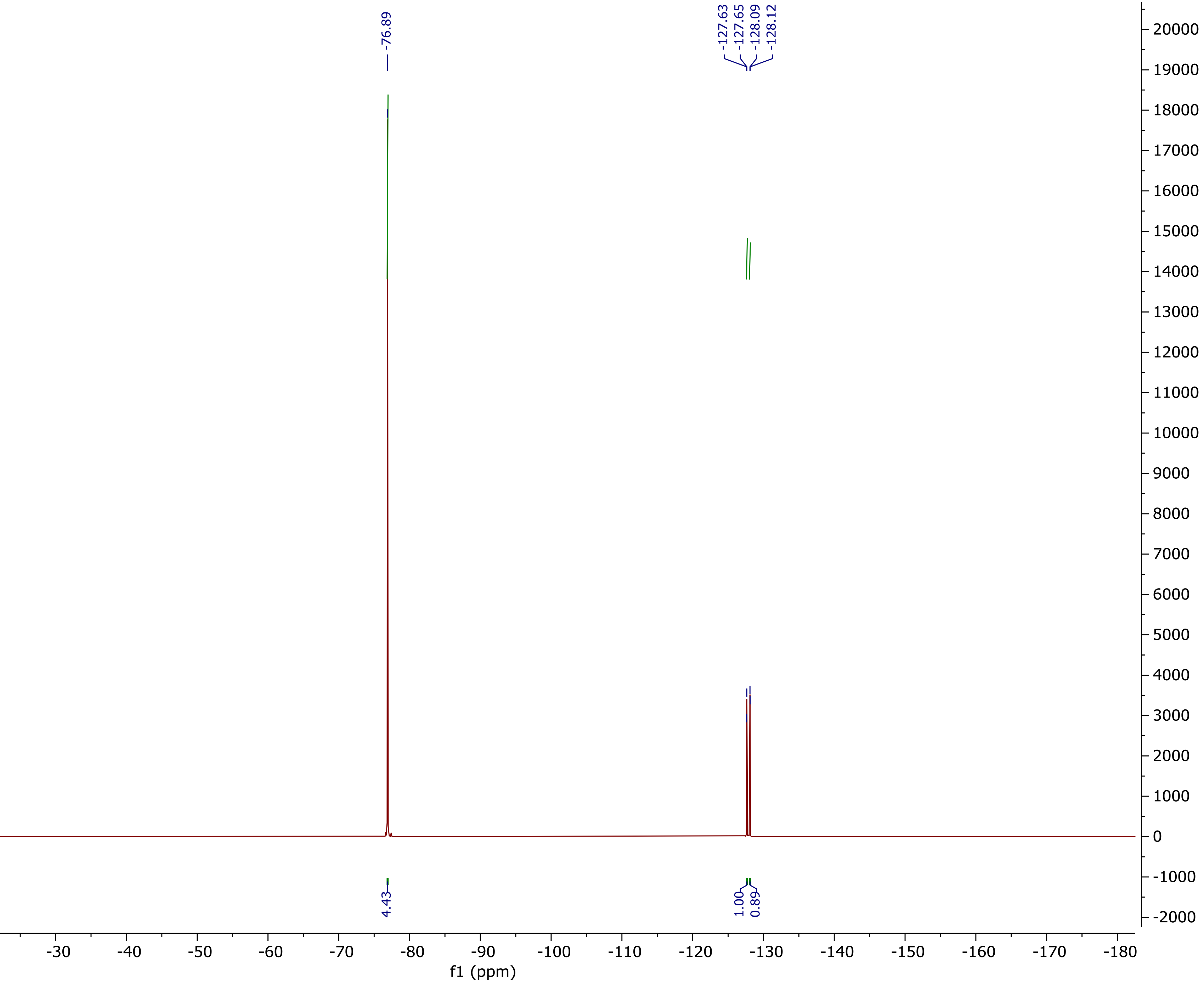

101 MHz, Methanol-d_4

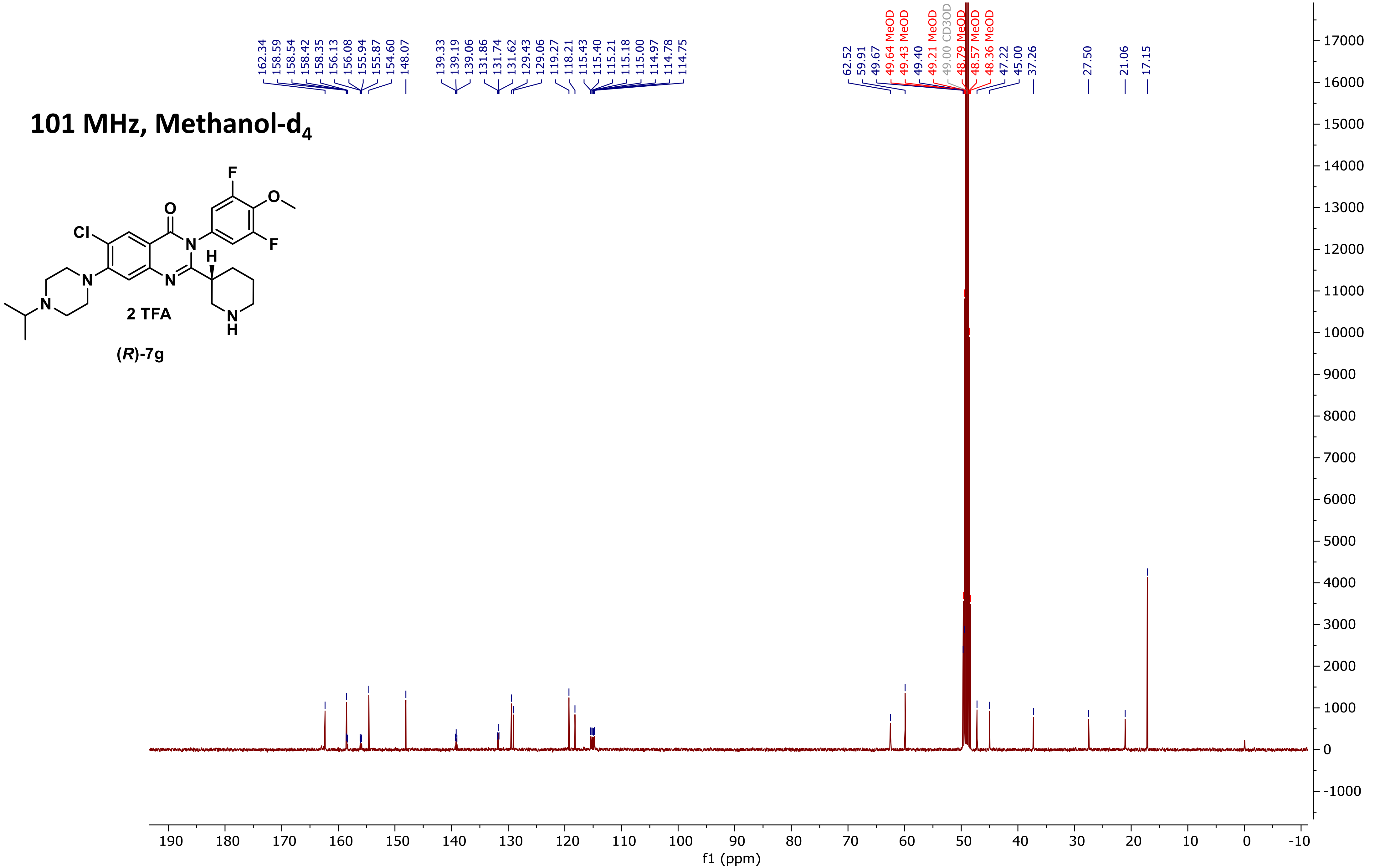

(*R*)-7g

400 MHz, Methanol-d_4

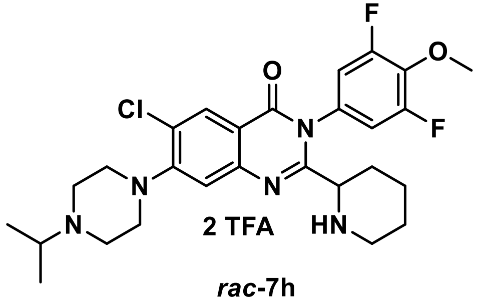

rac-7h

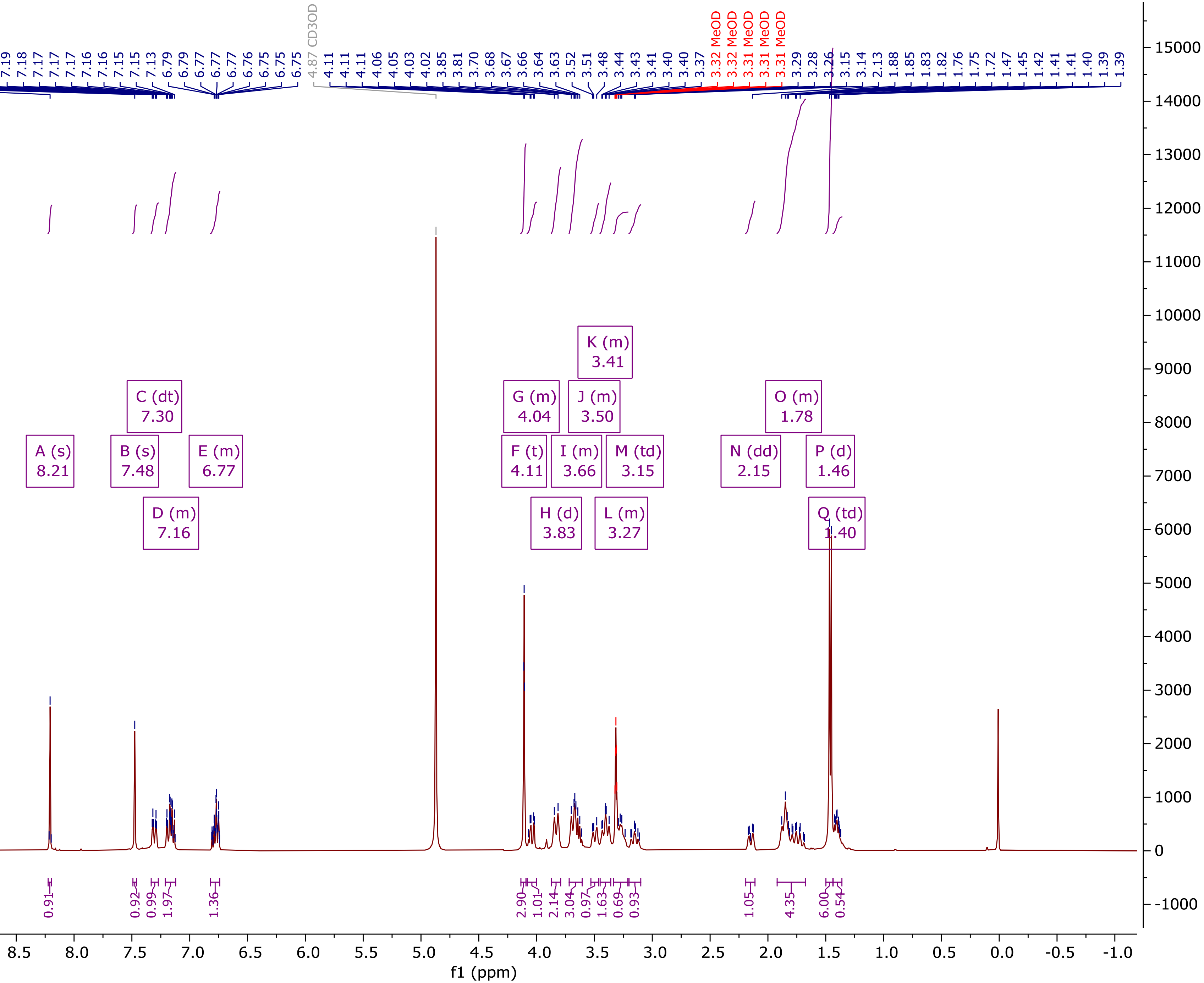

376 MHz, Methanol-d_4

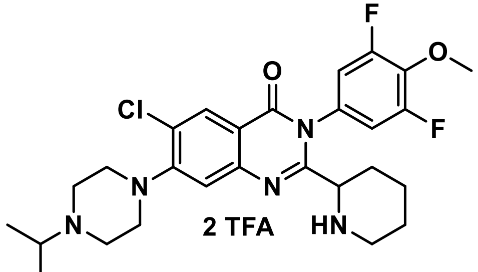

rac-7h

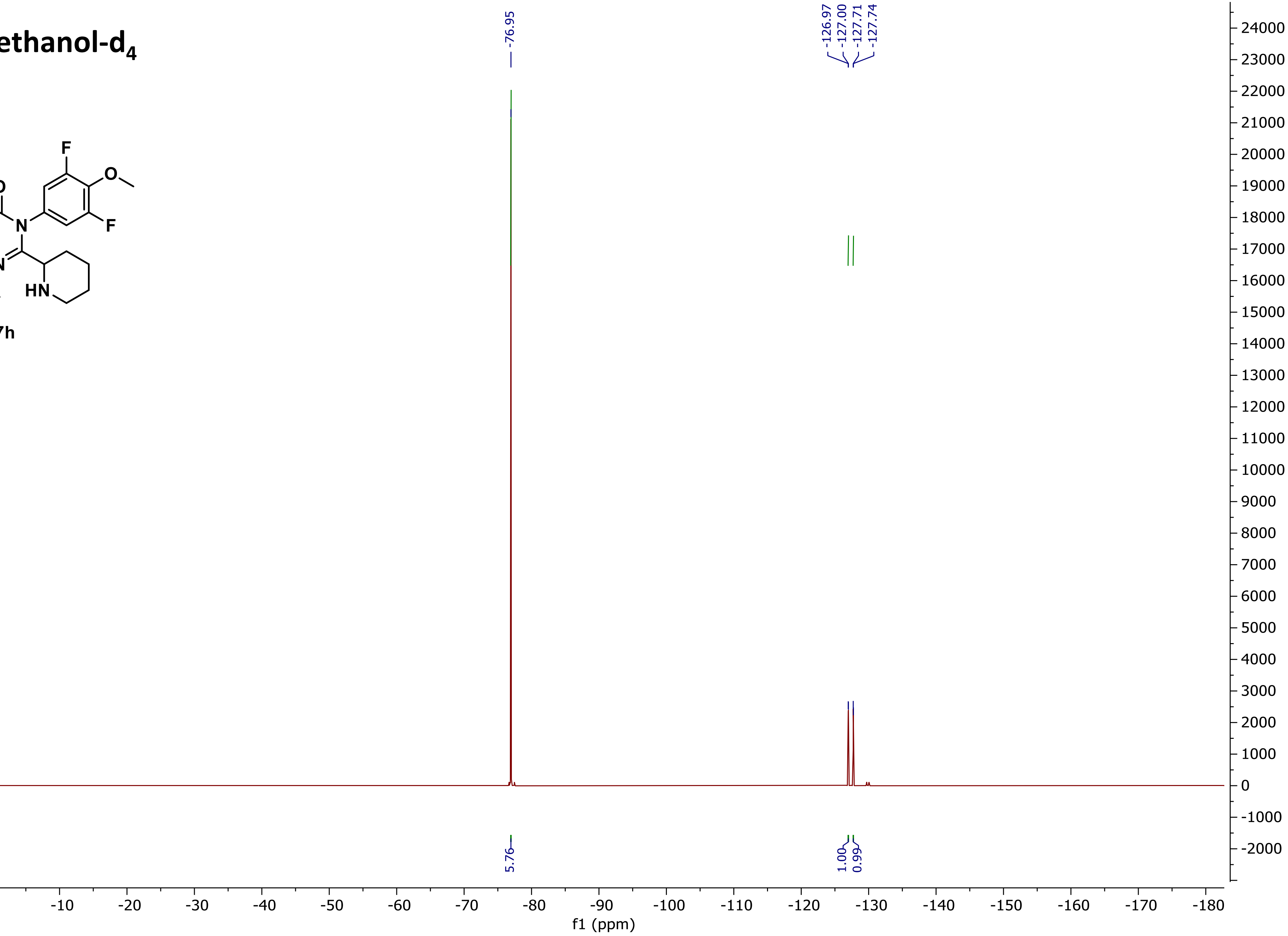

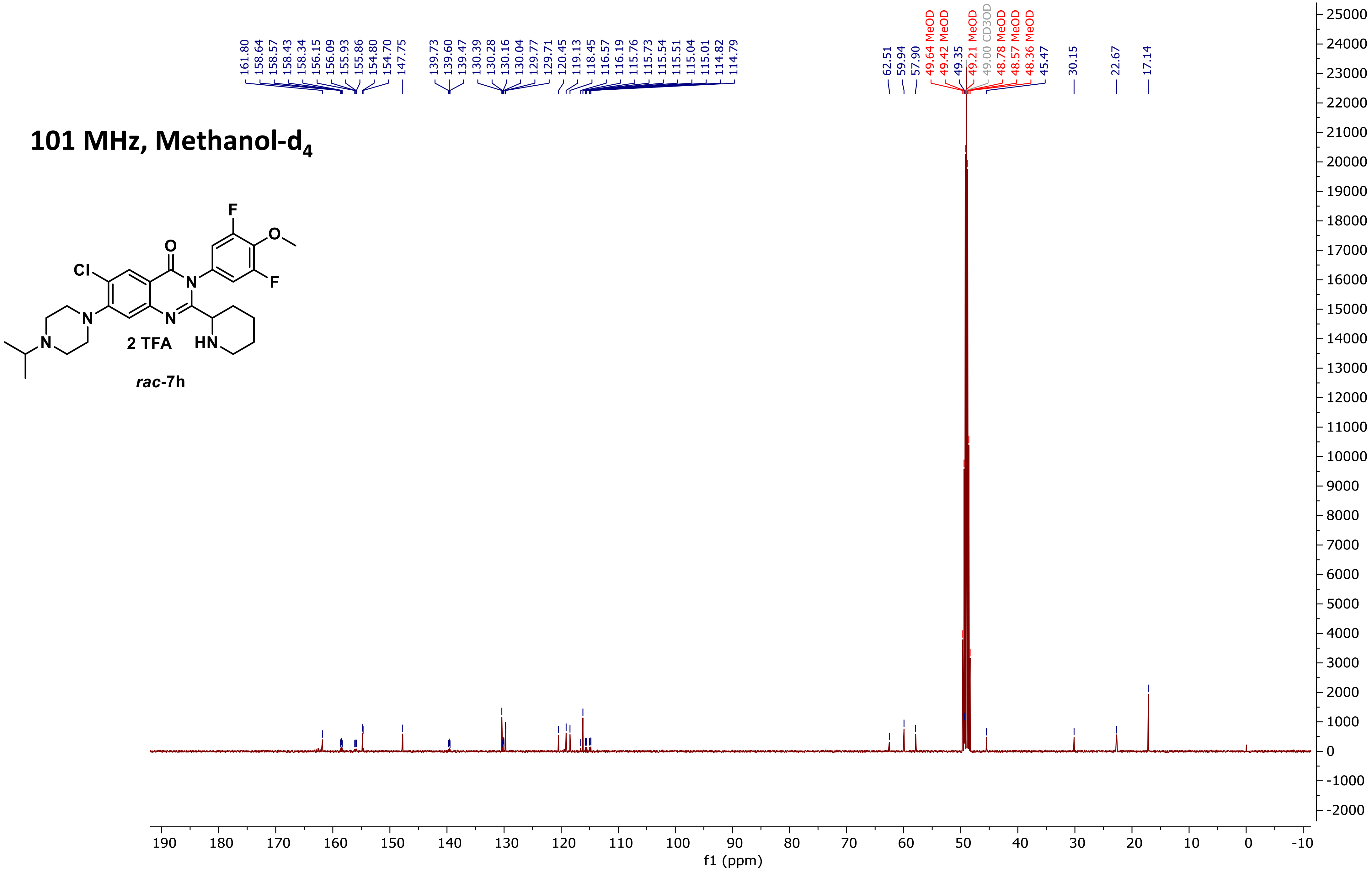
101 MHz, Methanol-d4
rac-7h
2 TFA
161.80 158.64 158.57 158.43 158.34 156.15 156.09 155.93 155.86 154.80 154.70 147.75 139.73 139.60 139.47 130.39 130.28 130.16 130.04 129.77 129.71 120.45 119.13 118.45 116.57 116.19 115.76 115.73 115.54 115.51 115.04 115.01 114.82 114.79
62.51 59.94 57.90 49.64 MeOD 49.42 MeOD 49.35 49.21 MeOD 49.00 CD3OD 48.78 MeOD 48.57 MeOD 48.36 MeOD 45.47 30.15 22.67 17.14
f1 (ppm)

376 MHz, Methanol-d_4

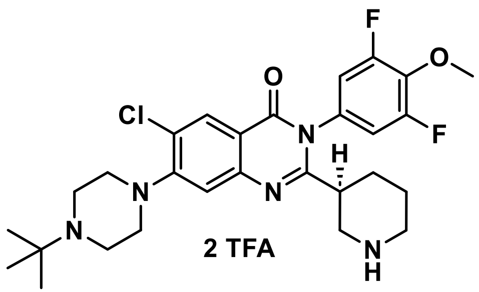

(*S*)-7j

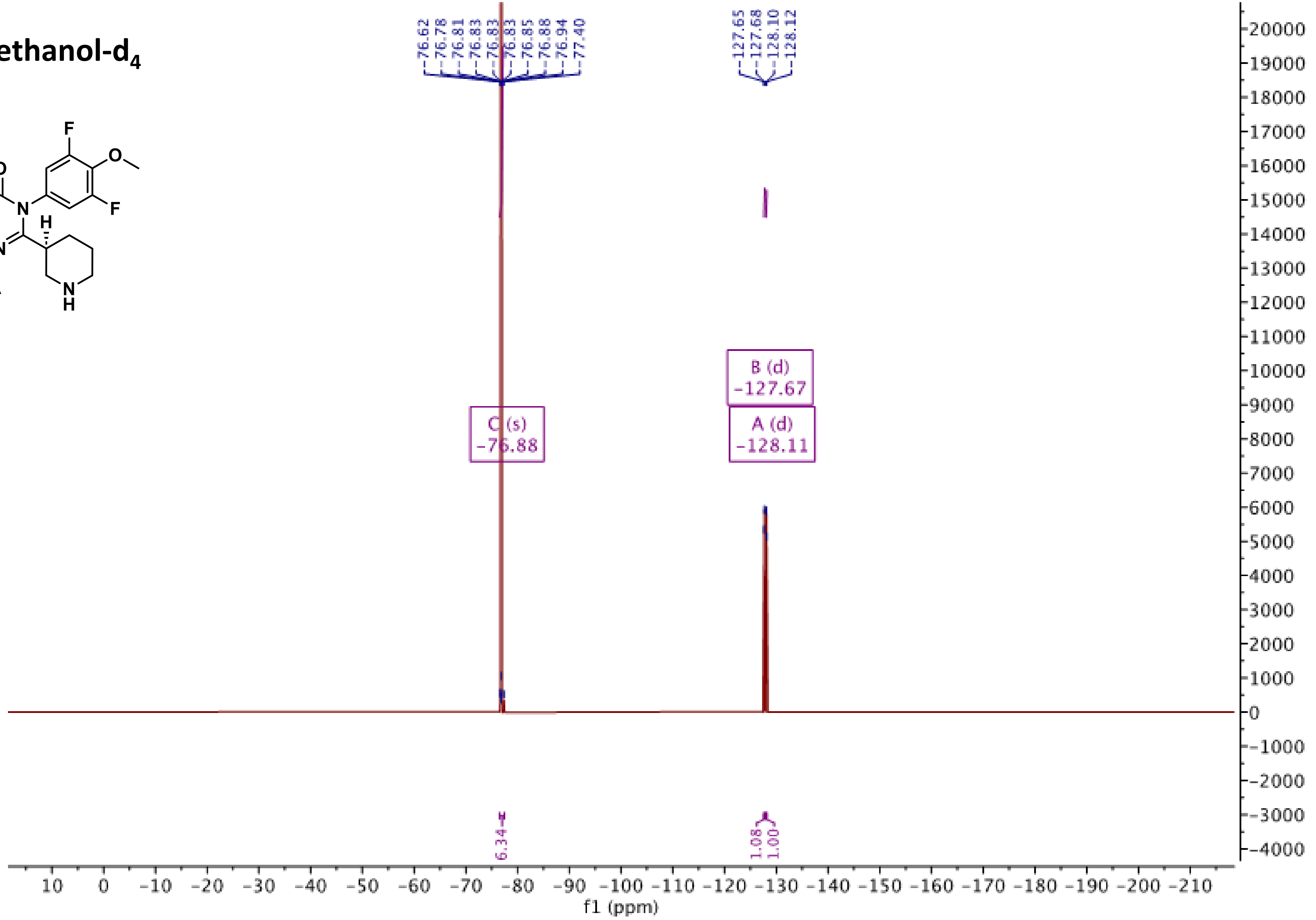

101 MHz, Methanol-d_4

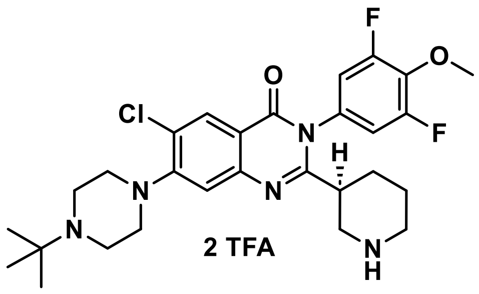

(*S*)-7j

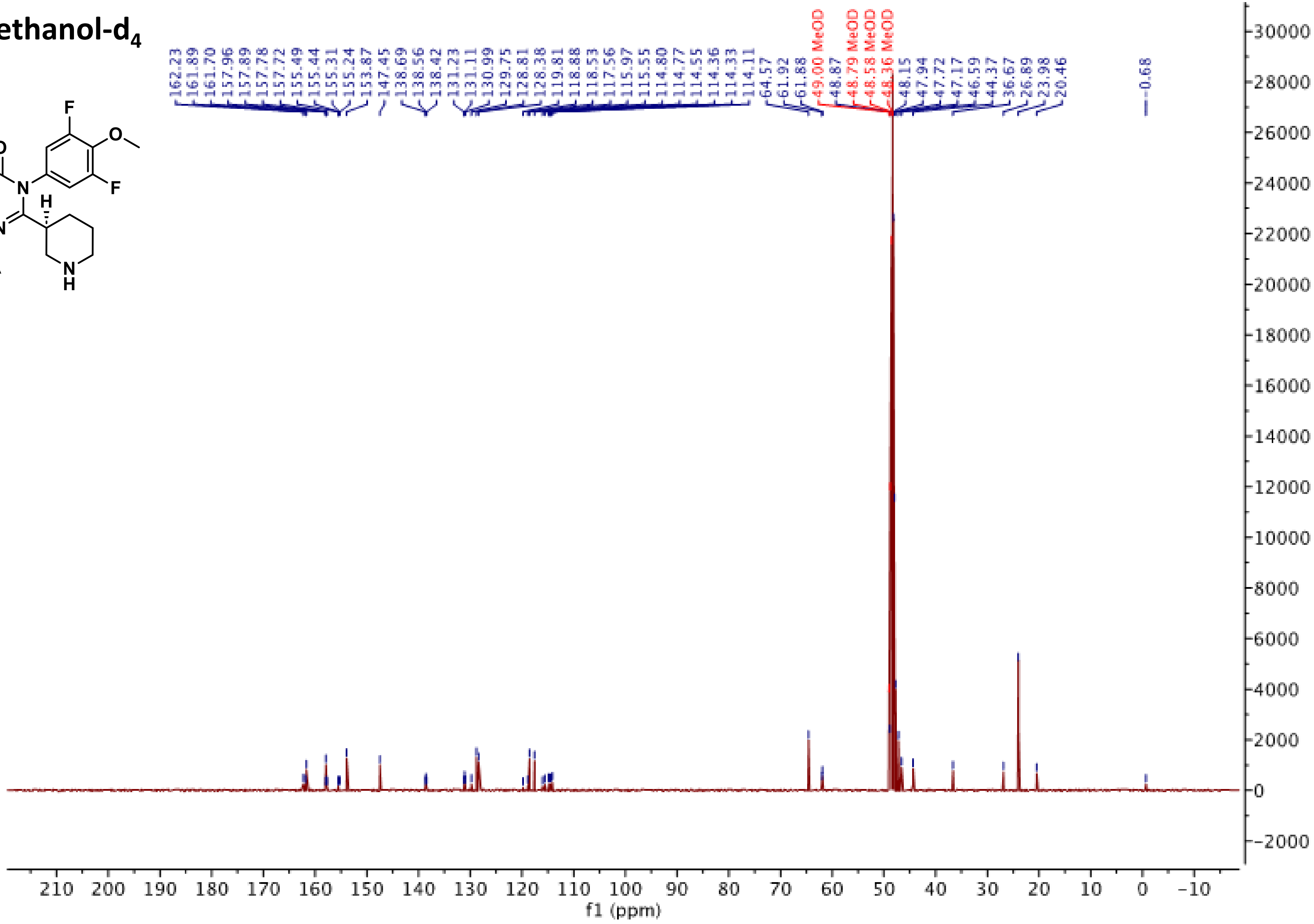

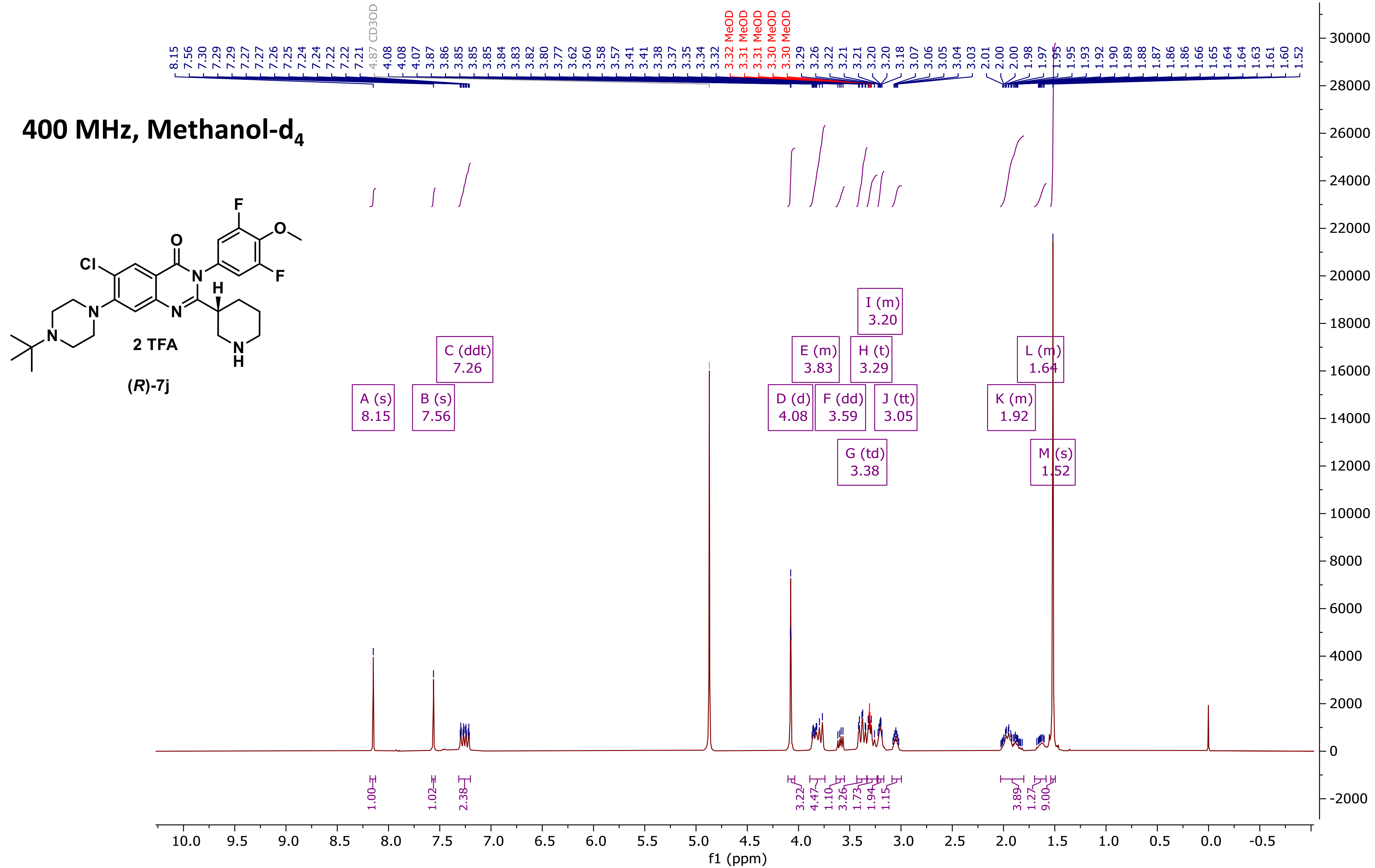

400 MHz, Methanol-d4
2 TFA
(R)-7j
A (s) 8.15
B (s) 7.56
C (ddt) 7.26
D (d) 4.08
E (m) 3.83
F (dd) 3.59
G (td) 3.38
H (t) 3.29
I (m) 3.20
J (tt) 3.05
K (m) 1.92
L (m) 1.64
M (s) 1.52
f1 (ppm)

376 MHz, Methanol-d_4

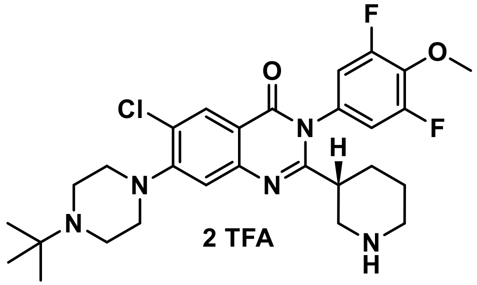

(*R*)-7j

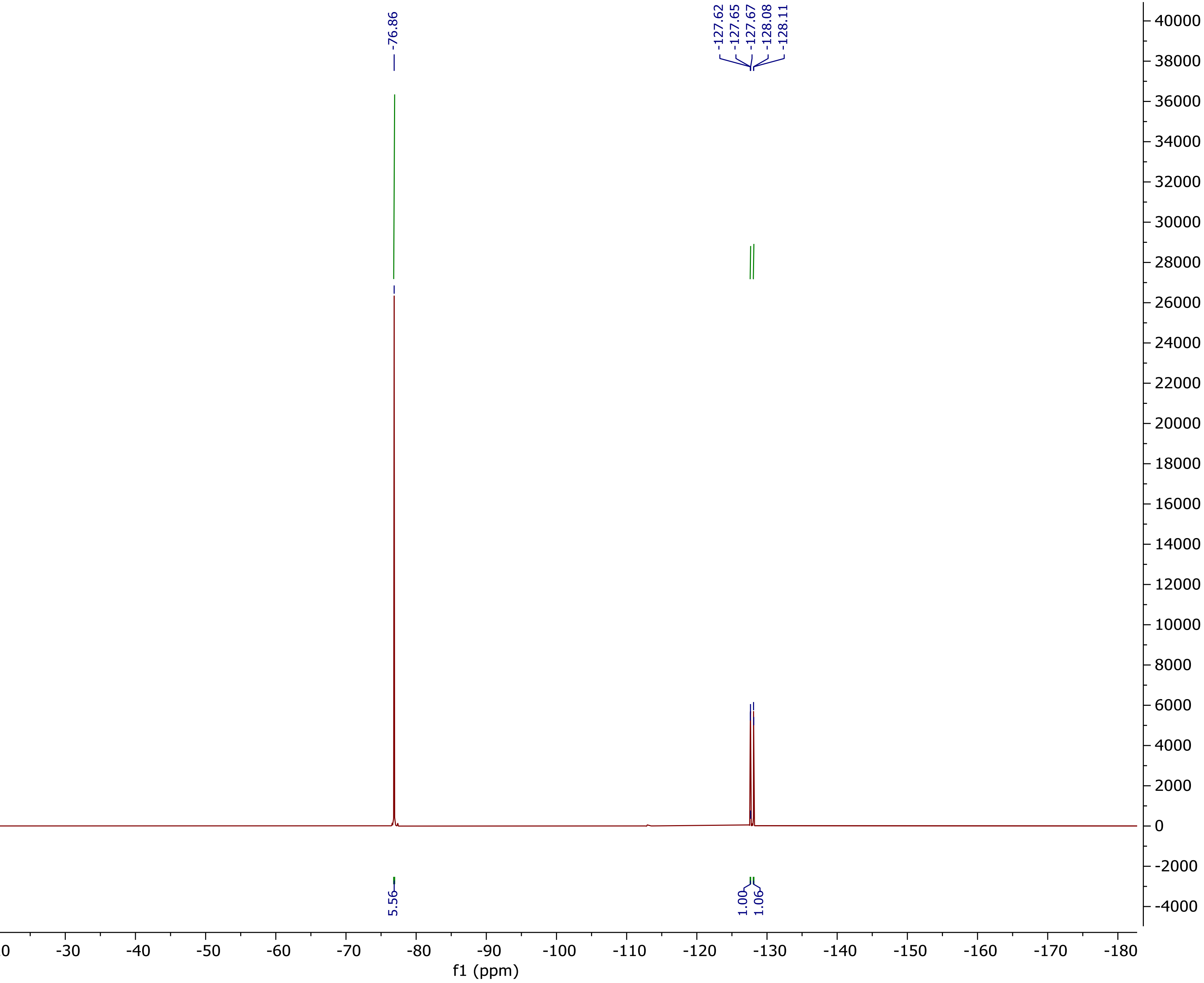

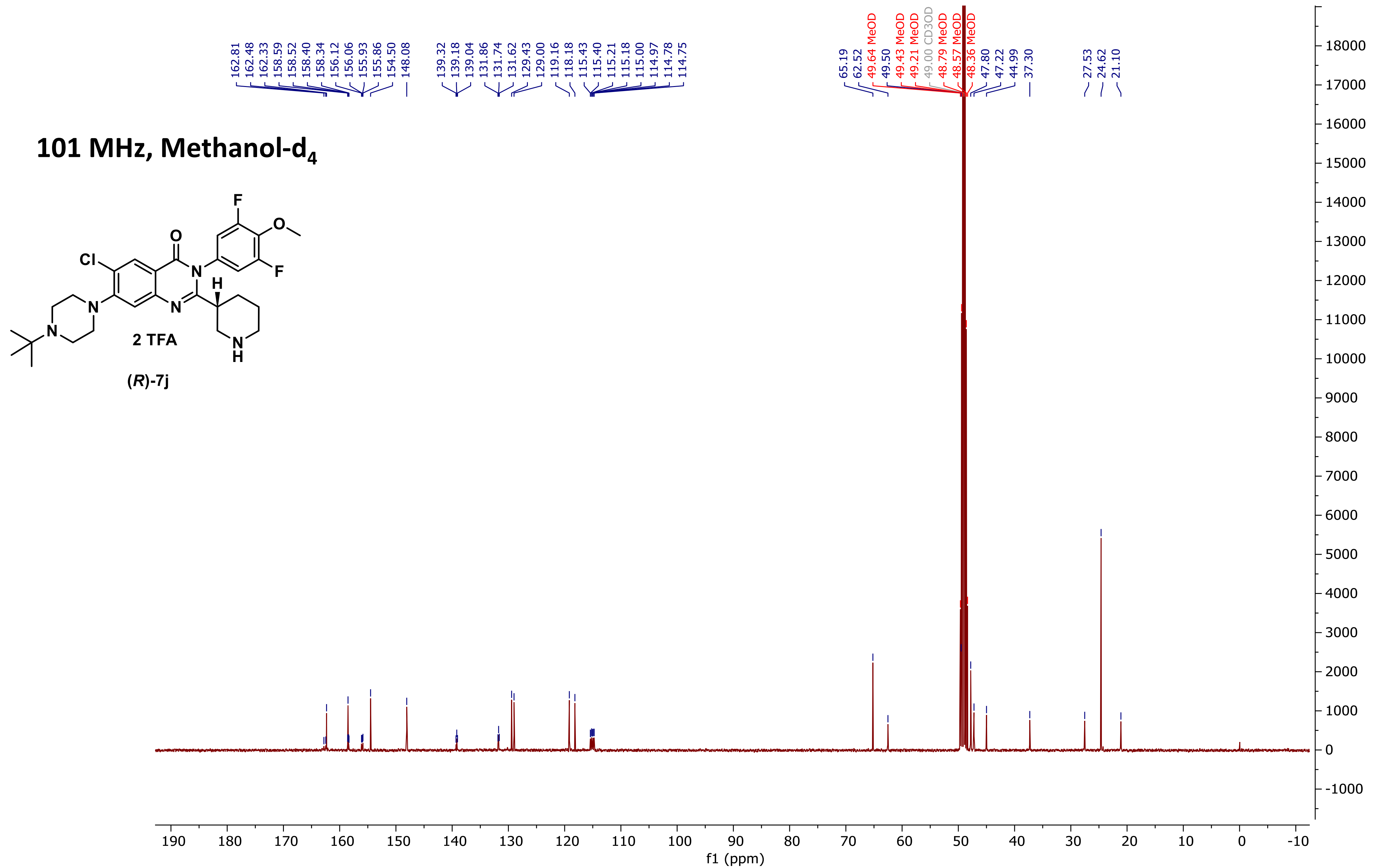
101 MHz, Methanol-d_4
(R)-7j
2 TFA
162.81 162.48 162.33 158.59 158.52 158.40 158.34 156.12 156.06 155.93 155.86 154.50 148.08
139.32 139.18 139.04 131.86 131.74 131.62 129.43 129.00 119.16 118.18 115.43 115.40 115.21 115.18 115.00 114.97 114.78 114.75
65.19 62.52 49.64 MeOD 49.50 49.43 MeOD 49.21 MeOD 49.00 CD3OD 48.79 MeOD 48.57 MeOD 48.36 MeOD 47.80 47.22 44.99 37.30
27.53 24.62 21.10
f1 (ppm)

376 MHz, Methanol-d_4

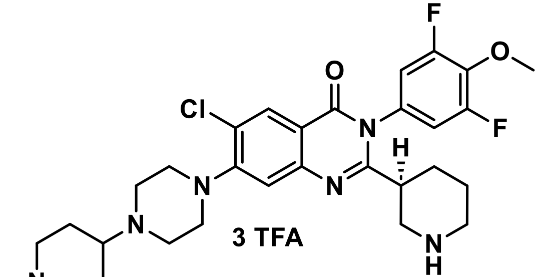

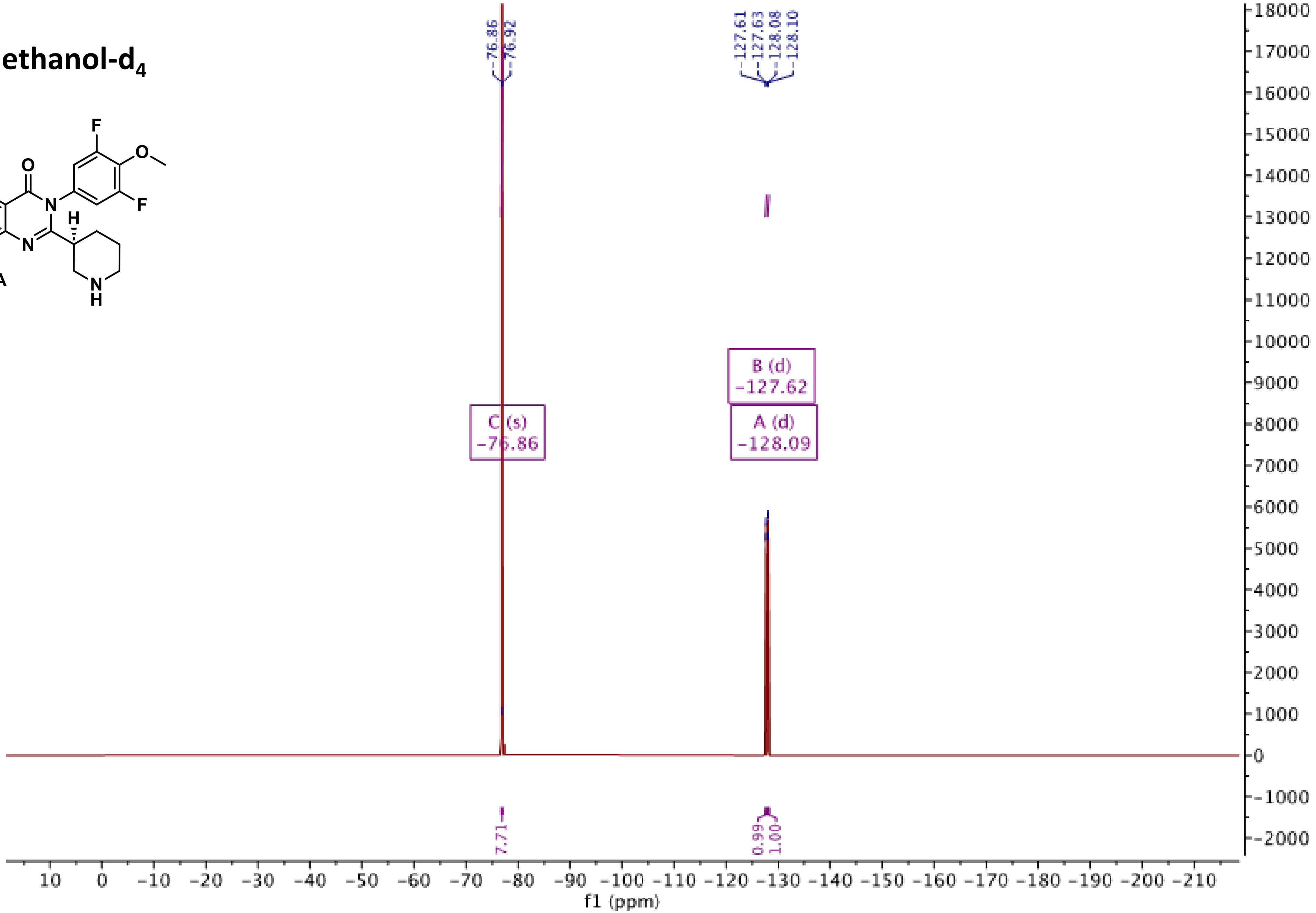

101 MHz, Methanol-d_4

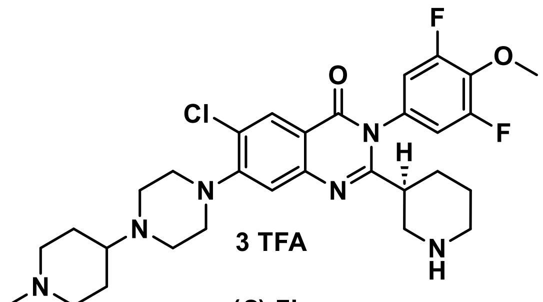

3 TFA

(*S*)-7l

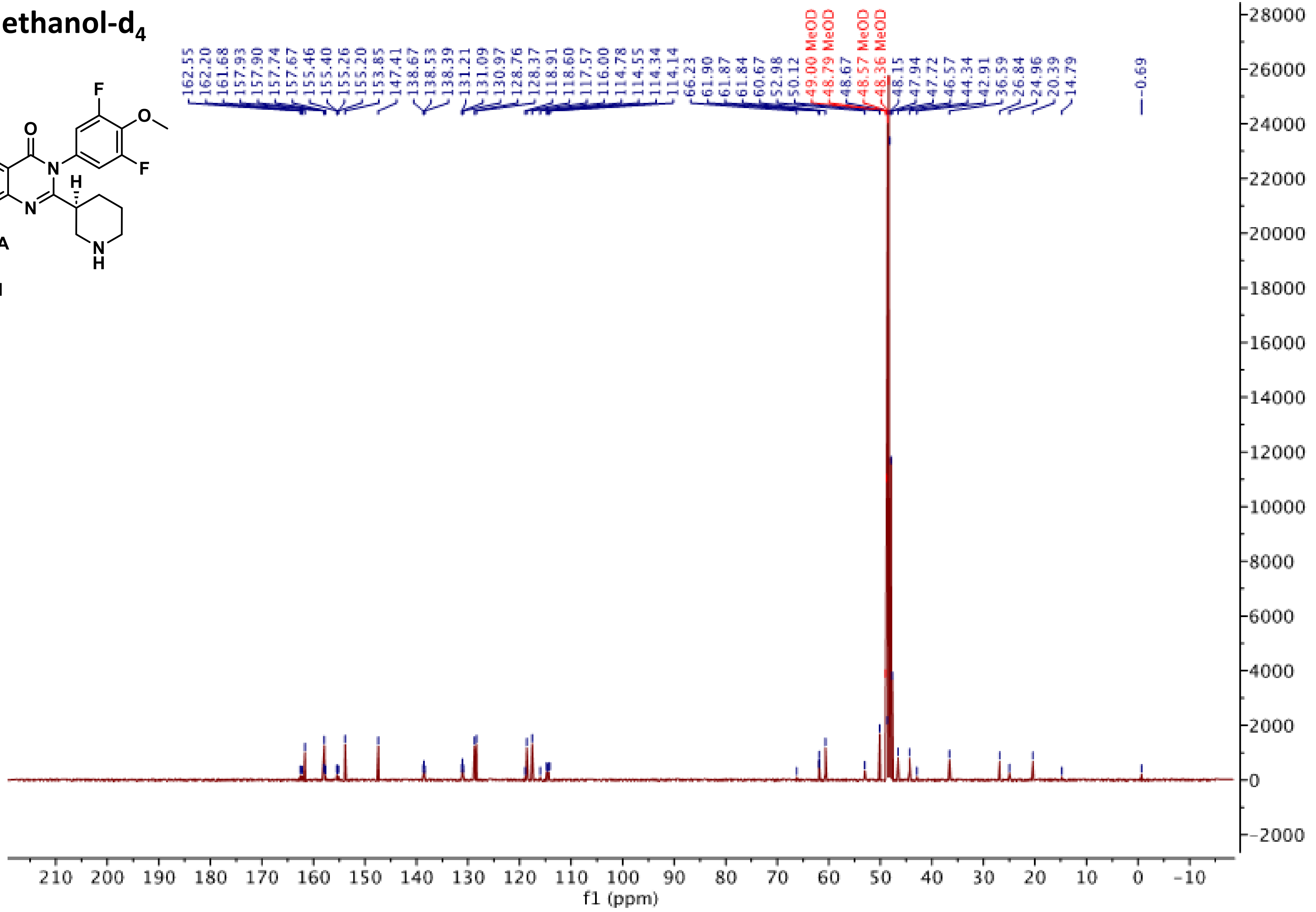

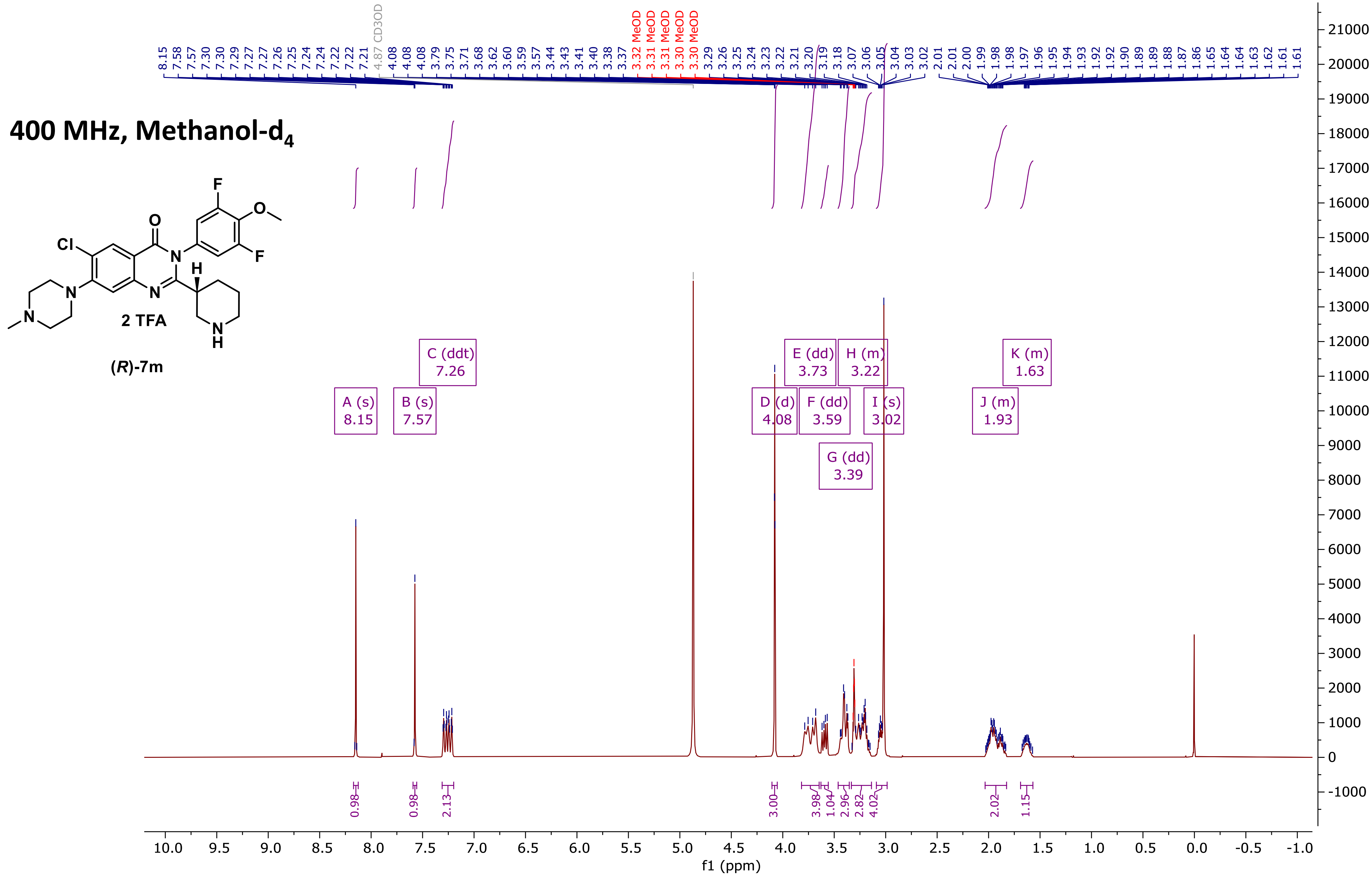
400 MHz, Methanol-d4
2 TFA
(R)-7m
A (s) 8.15
B (s) 7.57
C (ddt) 7.26
D (d) 4.08
E (dd) 3.73
F (dd) 3.59
G (dd) 3.39
H (m) 3.22
I (s) 3.02
J (m) 1.93
K (m) 1.63
f1 (ppm)

376 MHz, Methanol-d_4

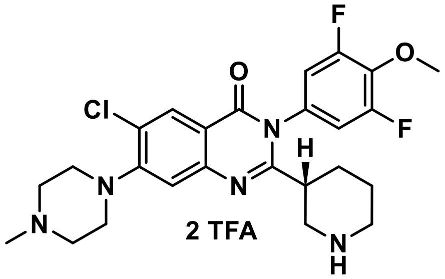

(*R*)-7m

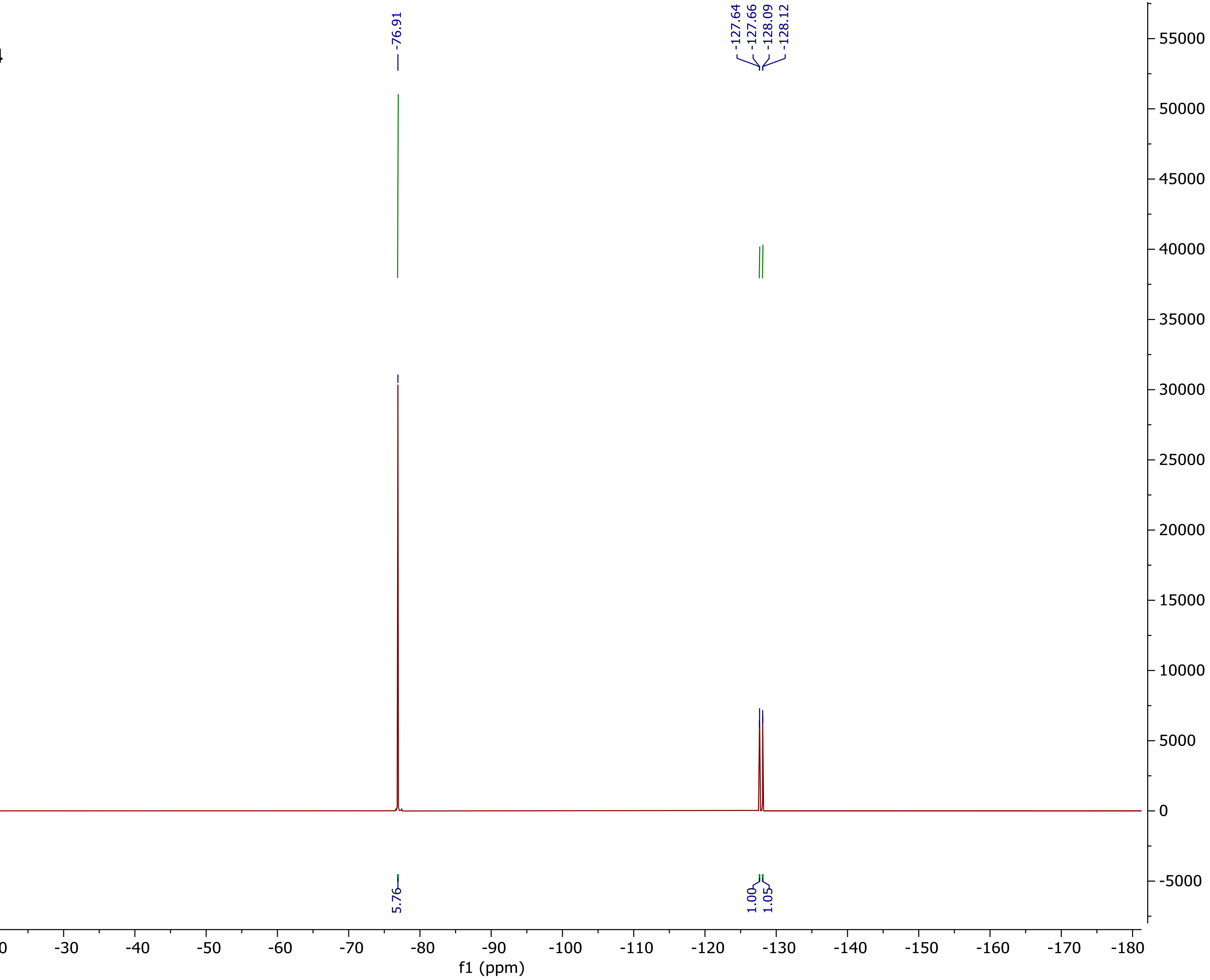

101 MHz, Methanol-d_4

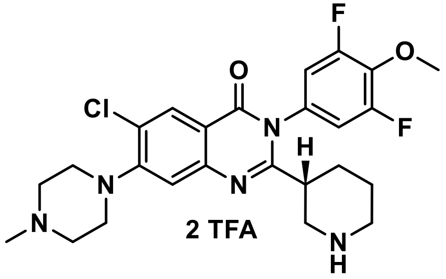

(*R*)-7m

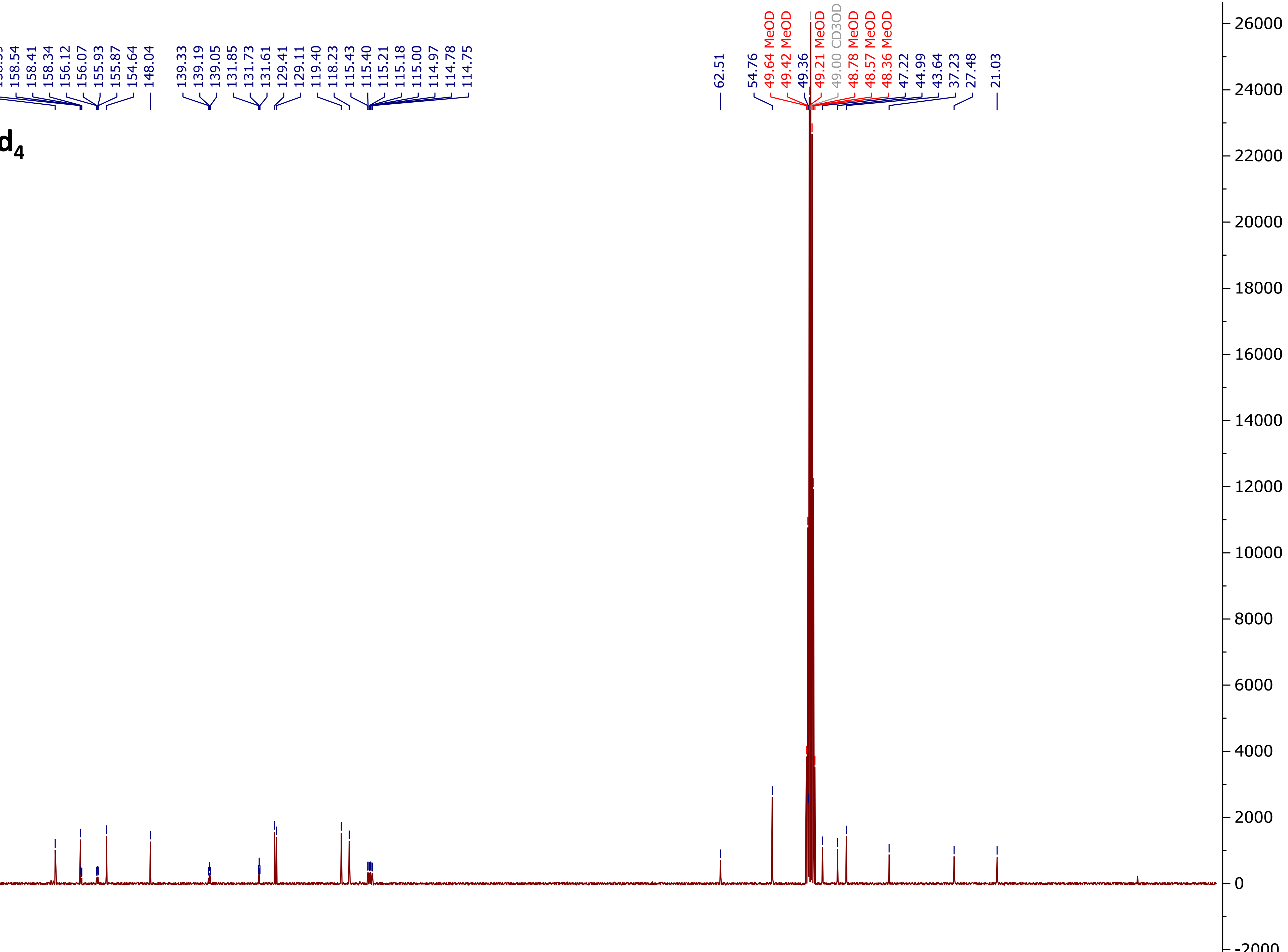

376 MHz, Methanol-d_4

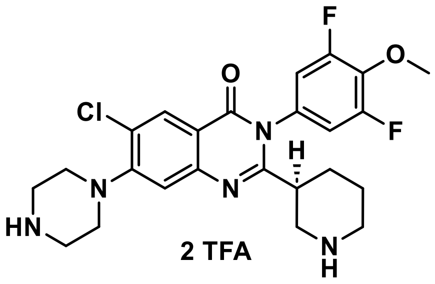

2 TFA

(*S*)-7n

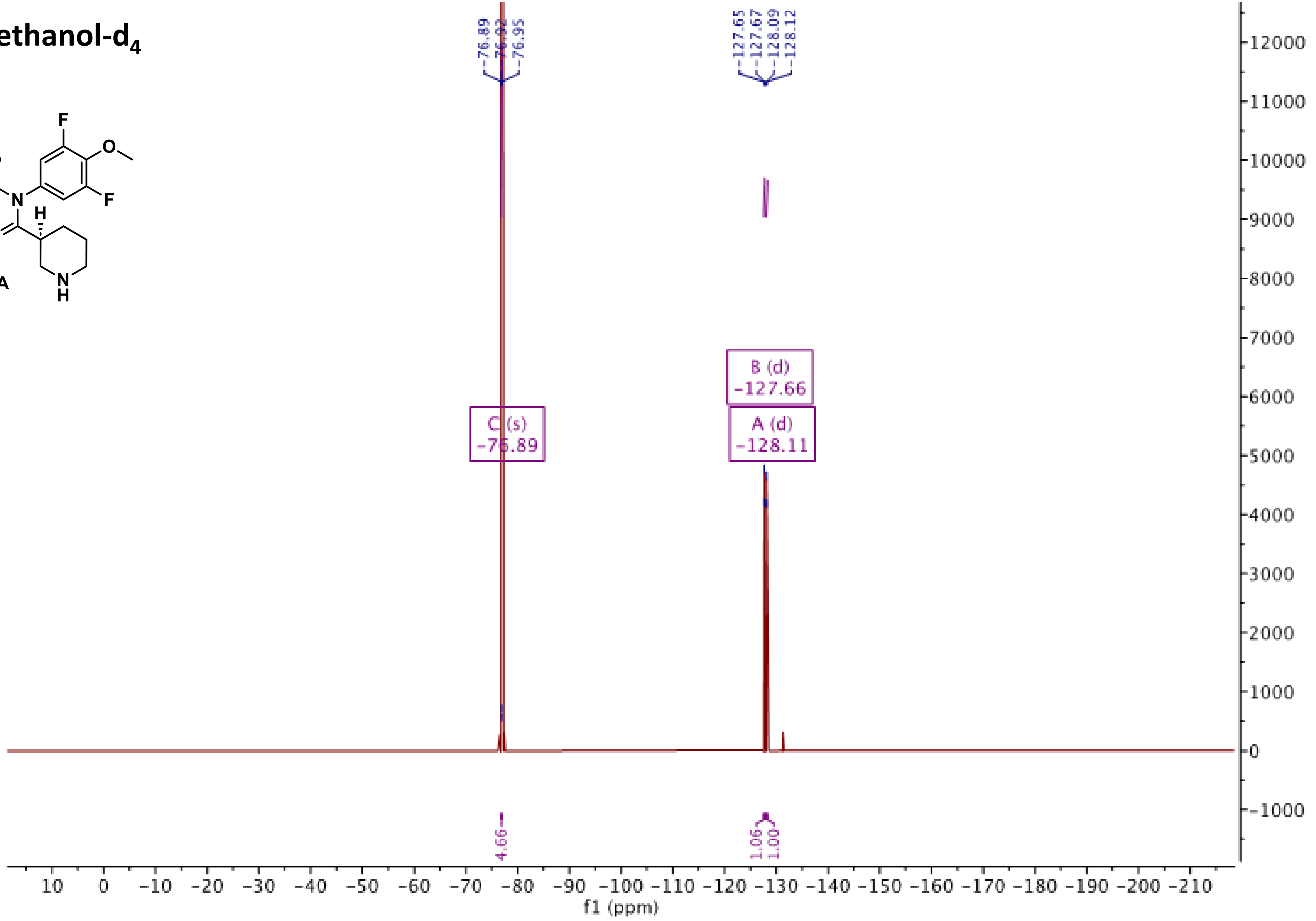

101 MHz, Methanol-d_4

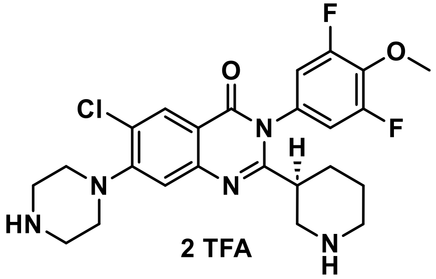

(*S*)-7n

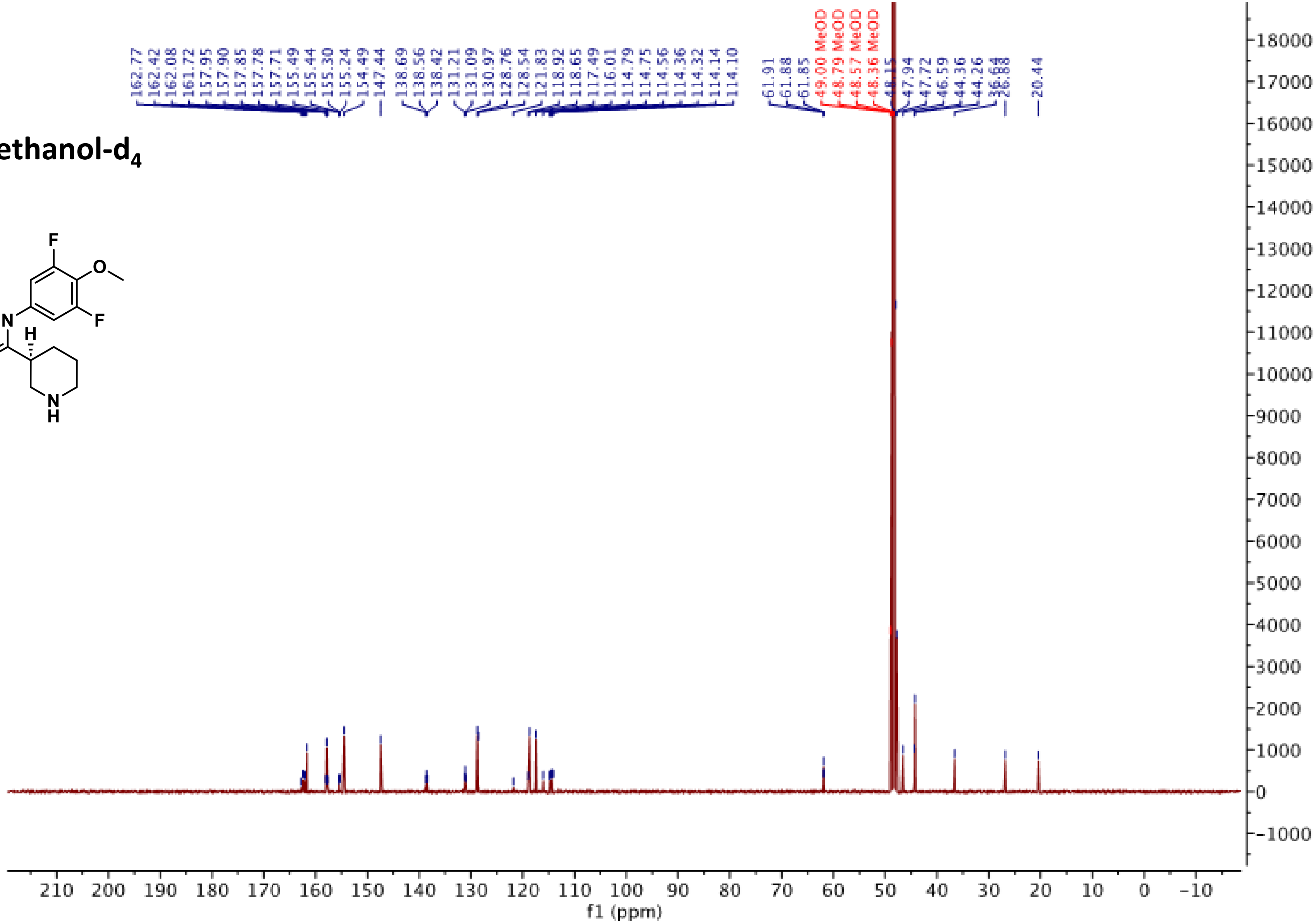

400 MHz, Methanol-d_4

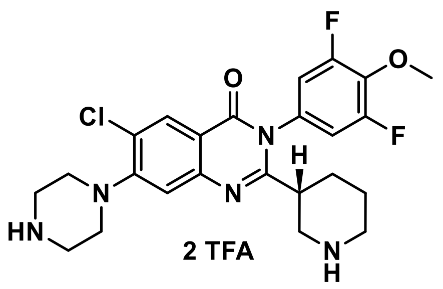

(*R*)-7n

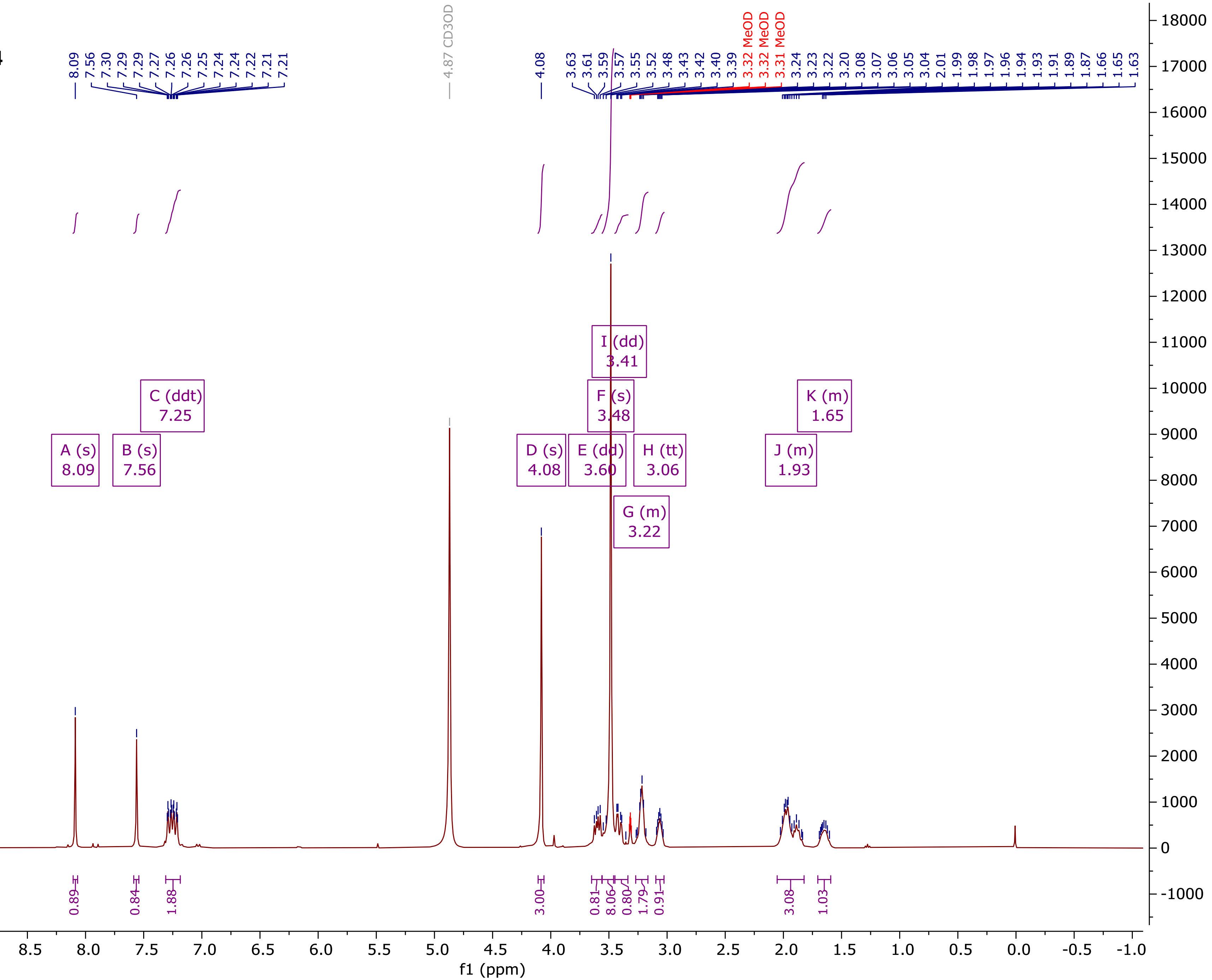

376 MHz, Methanol-d_4

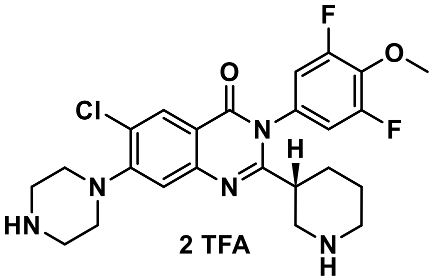

2 TFA

(*R*)-7n

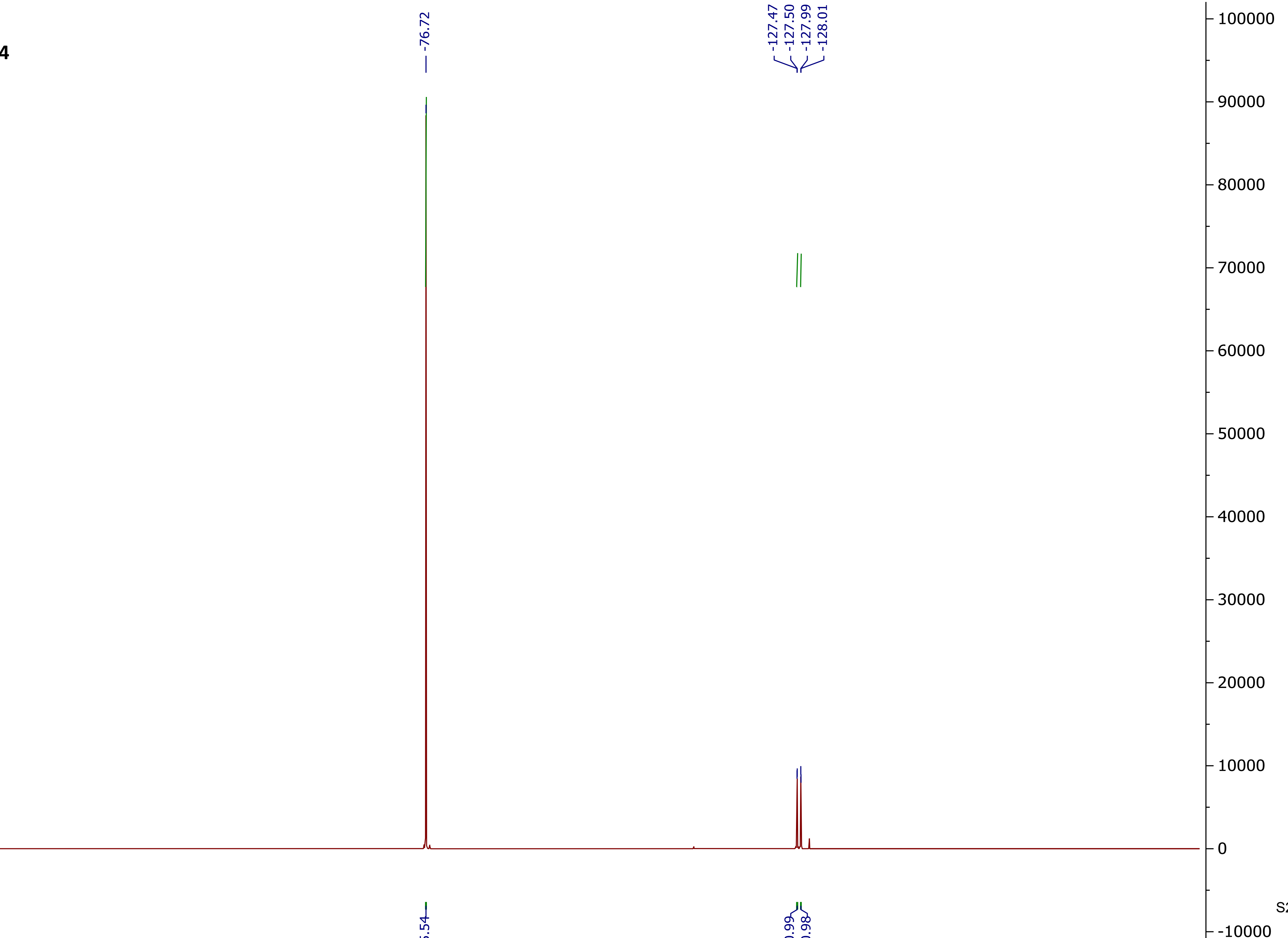

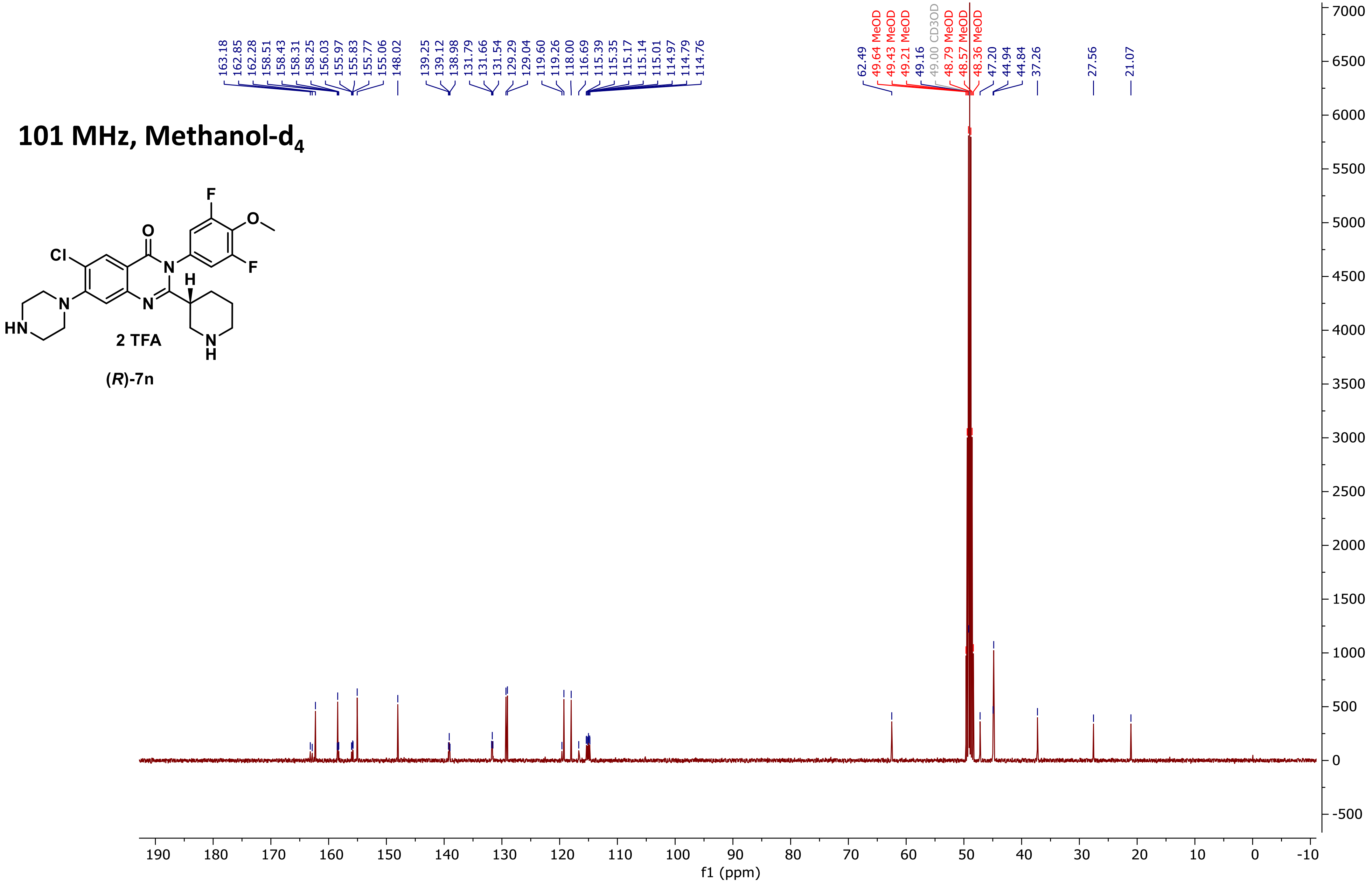
163.18
162.85
162.28
158.51
158.43
158.31
158.25
156.03
155.97
155.83
155.77
155.06
148.02
139.25
139.12
138.98
131.79
131.66
131.54
129.29
129.04
119.60
119.26
118.00
116.69
115.39
115.35
115.17
115.14
115.01
114.97
114.79
114.76
62.49
49.64 MeOD
49.43 MeOD
49.21 MeOD
49.16
49.00 CD3OD
48.79 MeOD
48.57 MeOD
48.36 MeOD
47.20
44.94
44.84
37.26
27.56
21.07
101 MHz, Methanol-d4
2 TFA
(R)-7n
f1 (ppm)

400 MHz, Methanol-d4

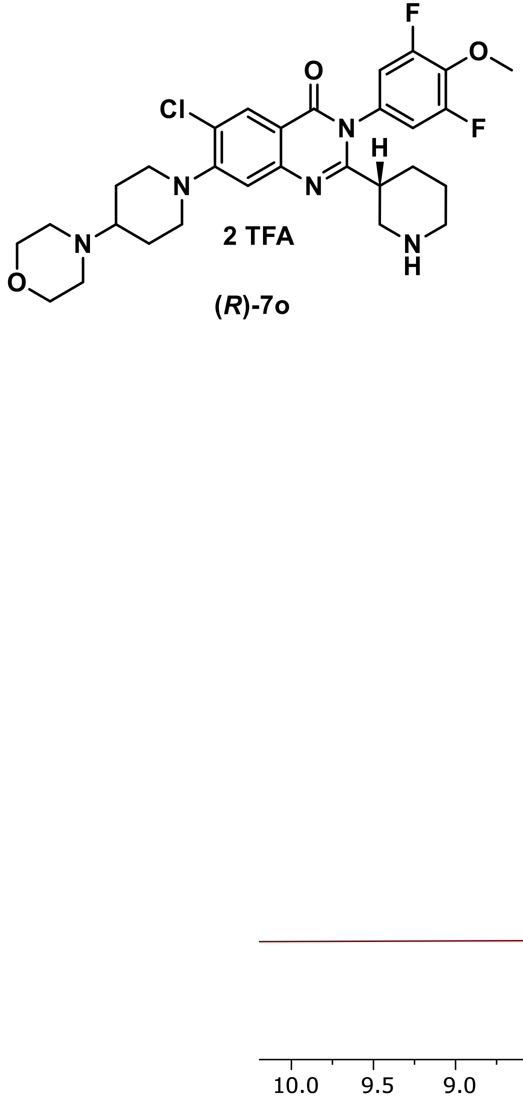

2 TFA

(*R*)-7o

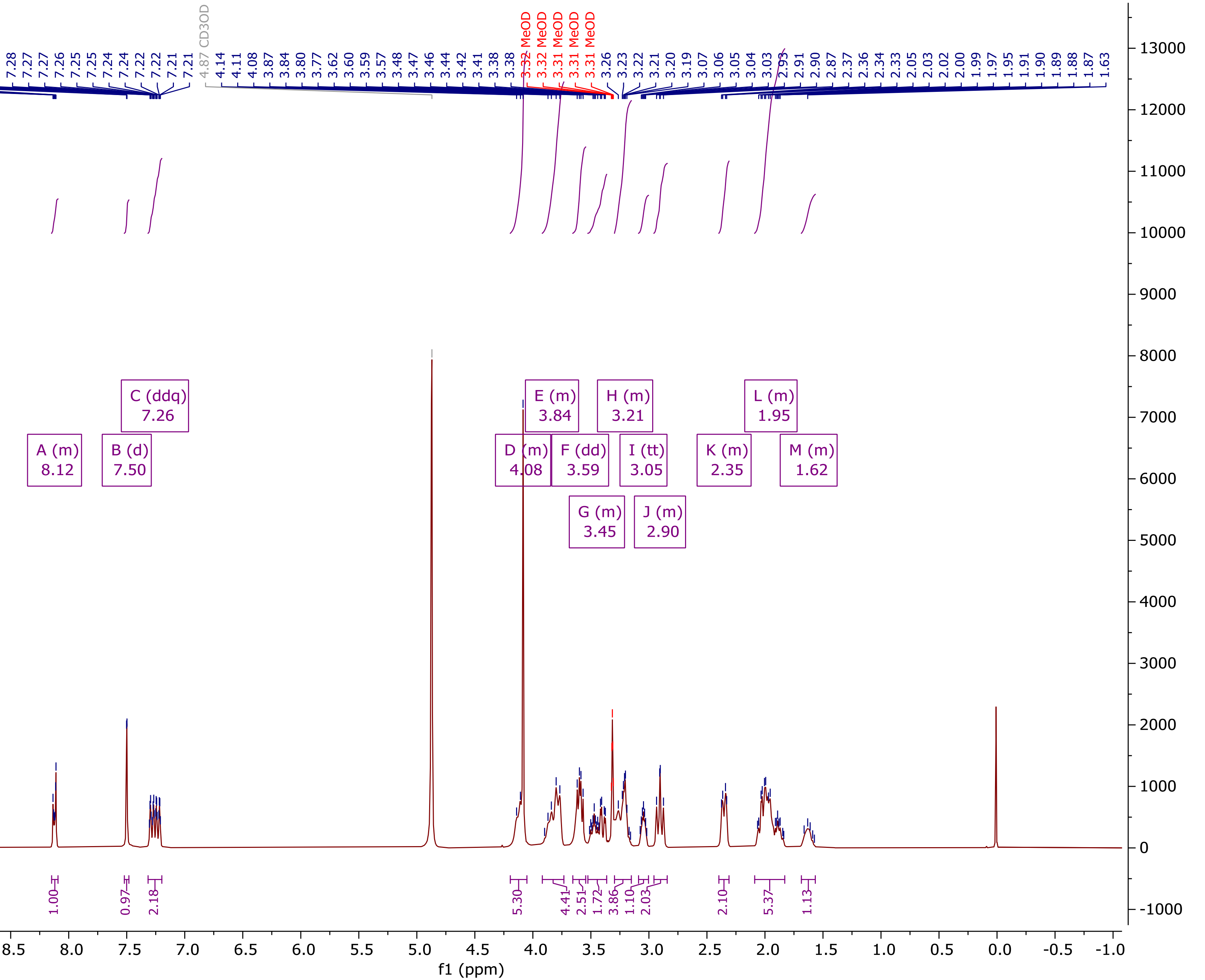

376 MHz, Methanol-d_4

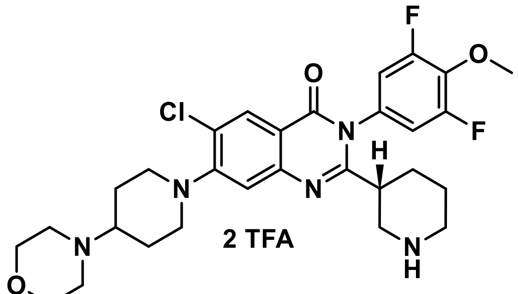

2 TFA

(*R*)-7o

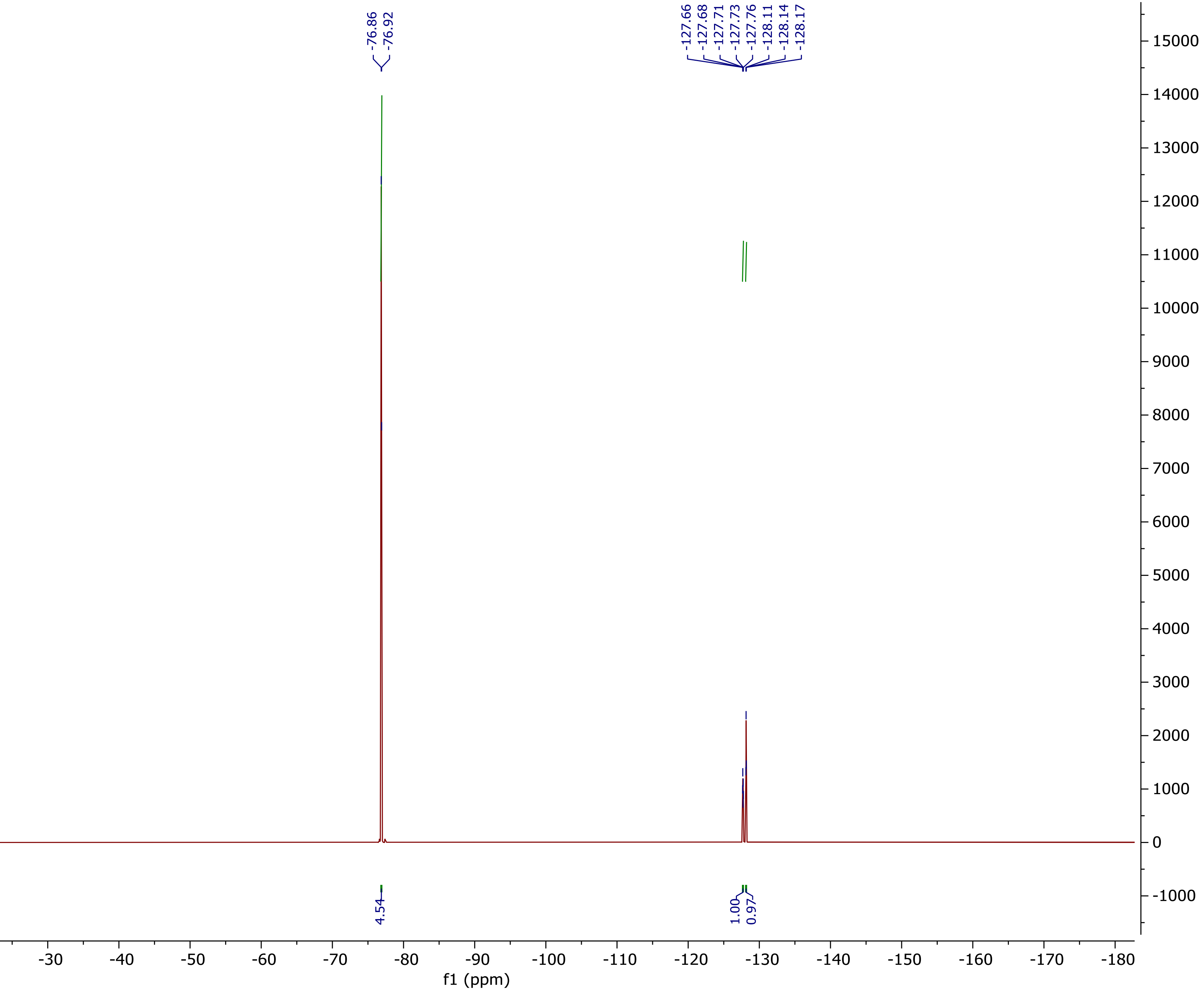

101 MHz, Methanol-d$_4$

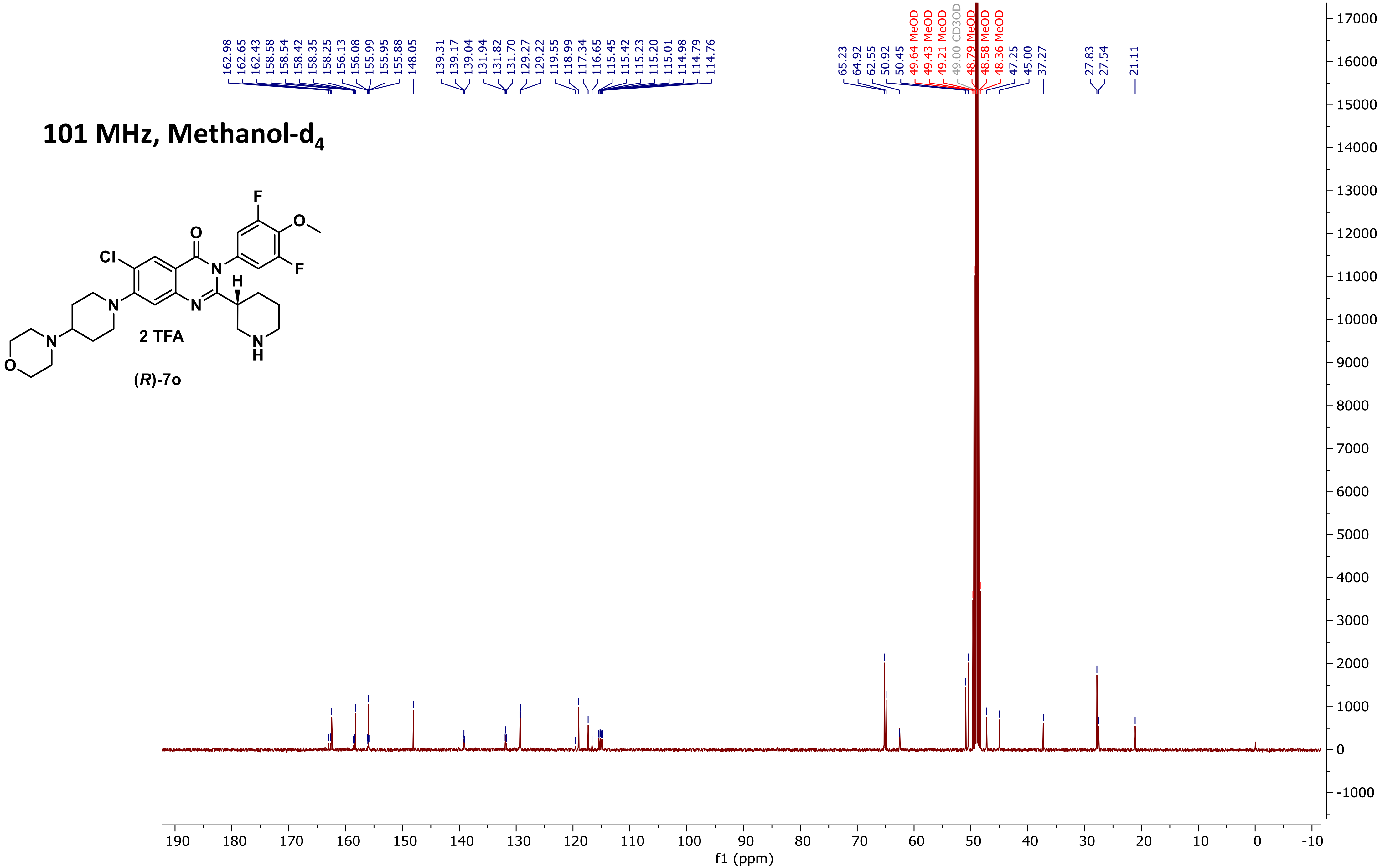

(*R*)-7o

376 MHz, Methanol-d_4

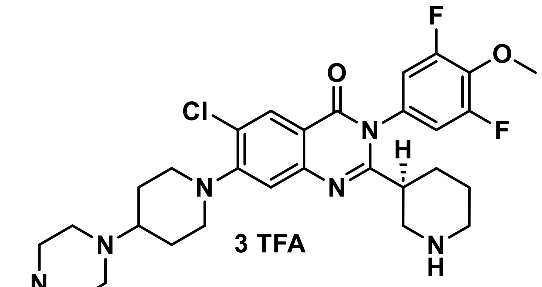

(*S*)-7p

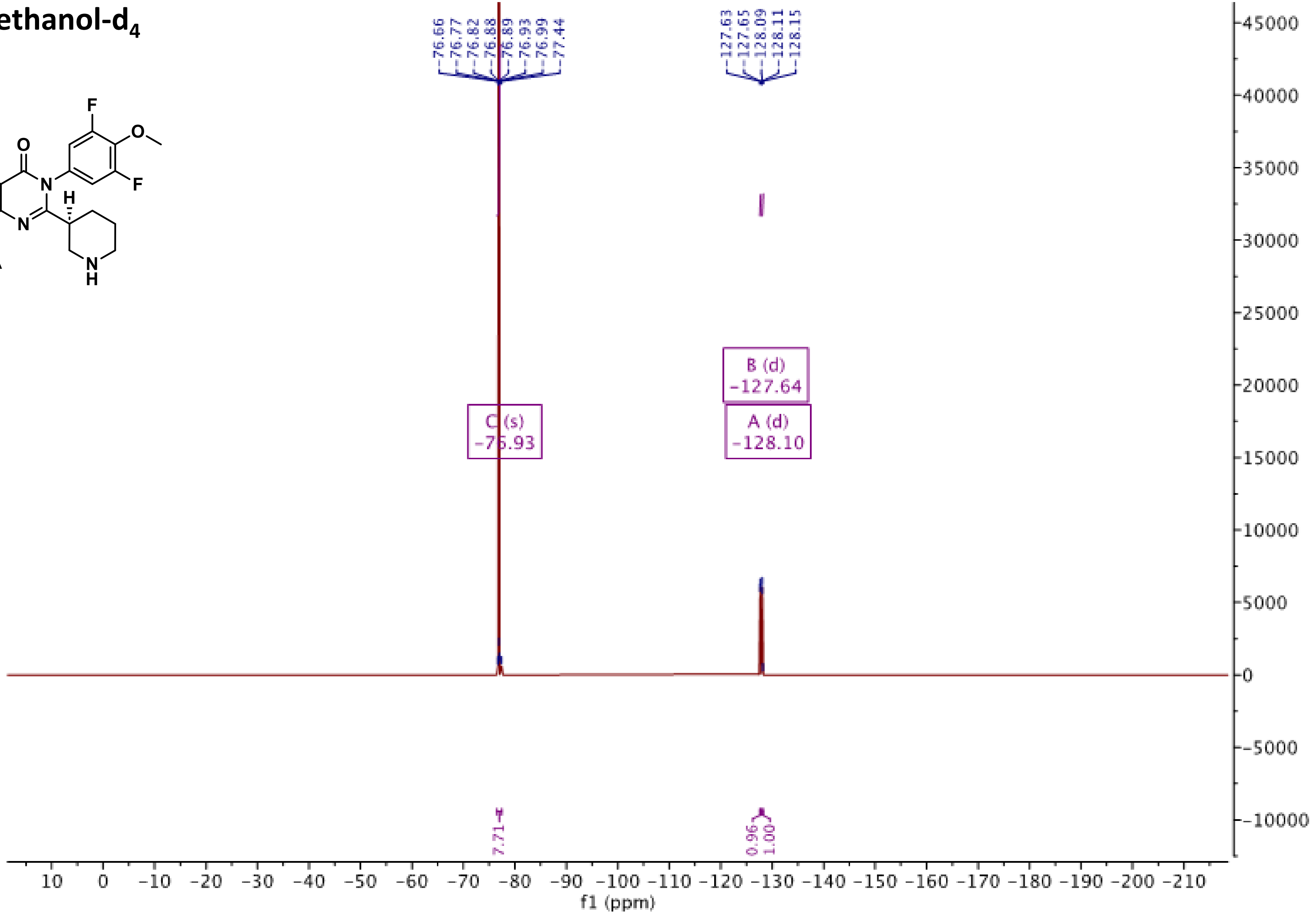

101 MHz, Methanol-d_4

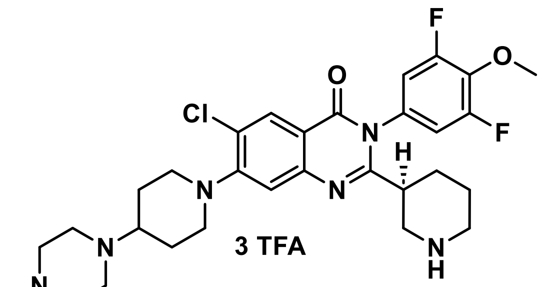

(*S*)-7p

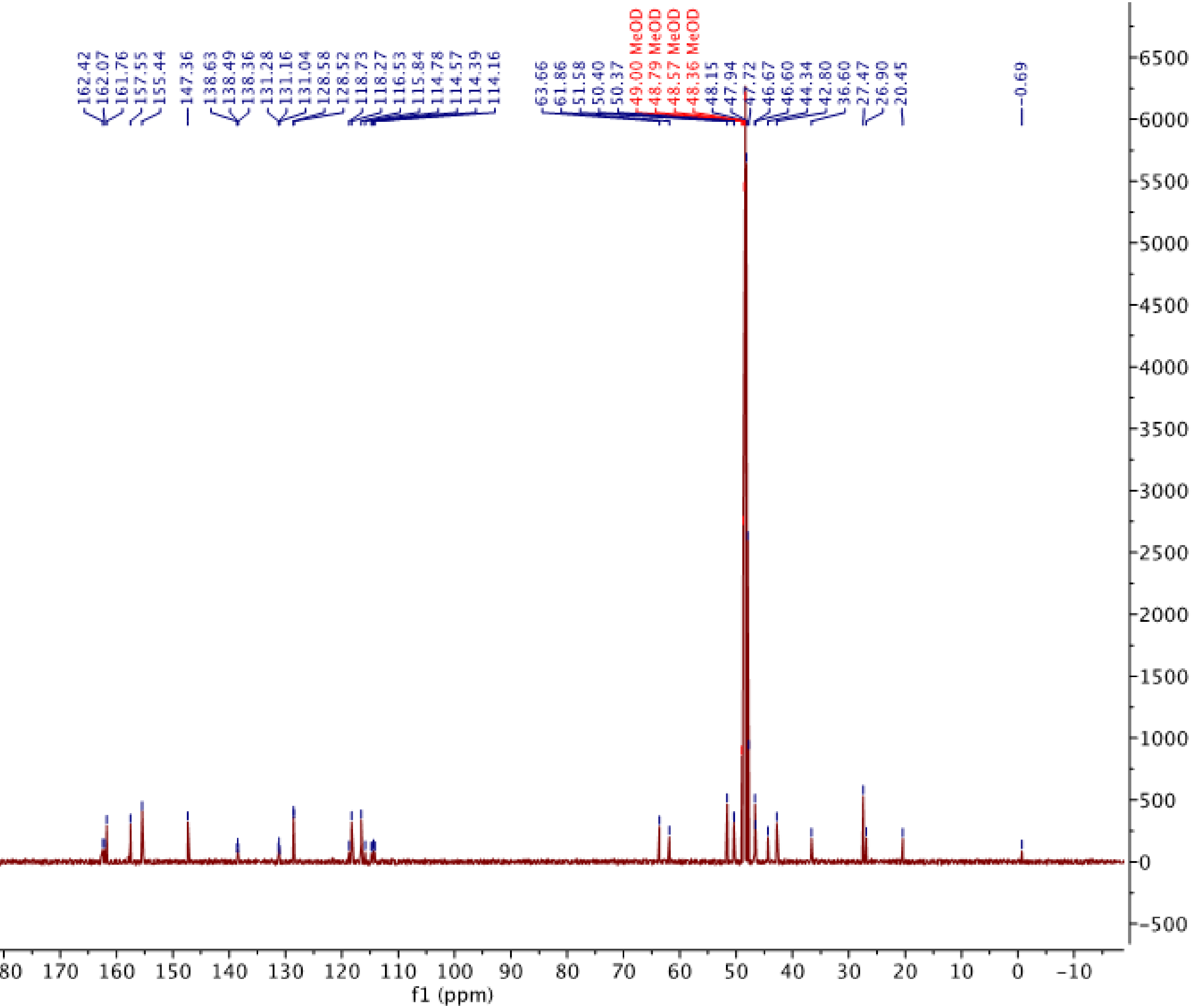

400 MHz, Methanol-d_4

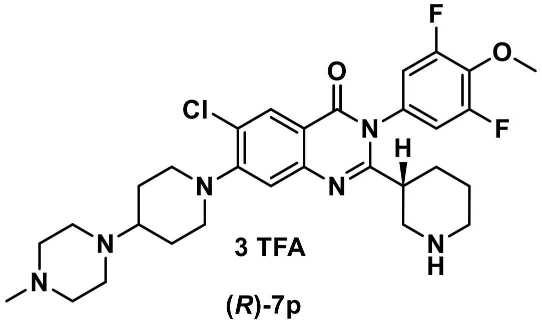

3 TFA

(*R*)-7p

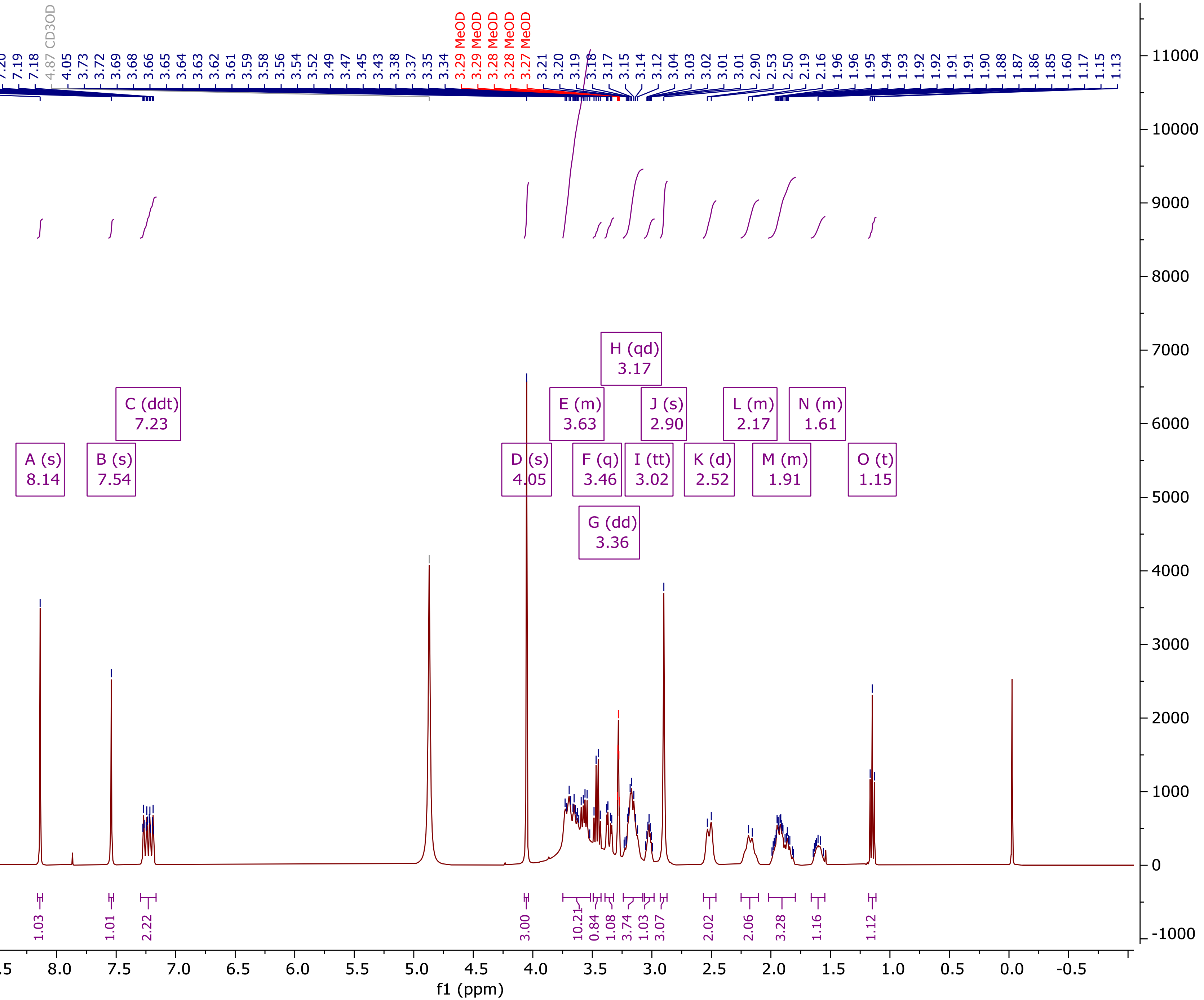

376 MHz, Methanol-d_4

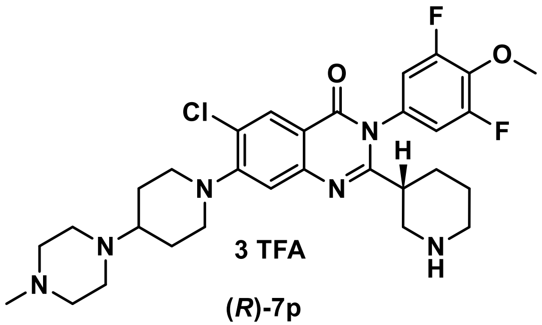

3 TFA

(*R*)-7p

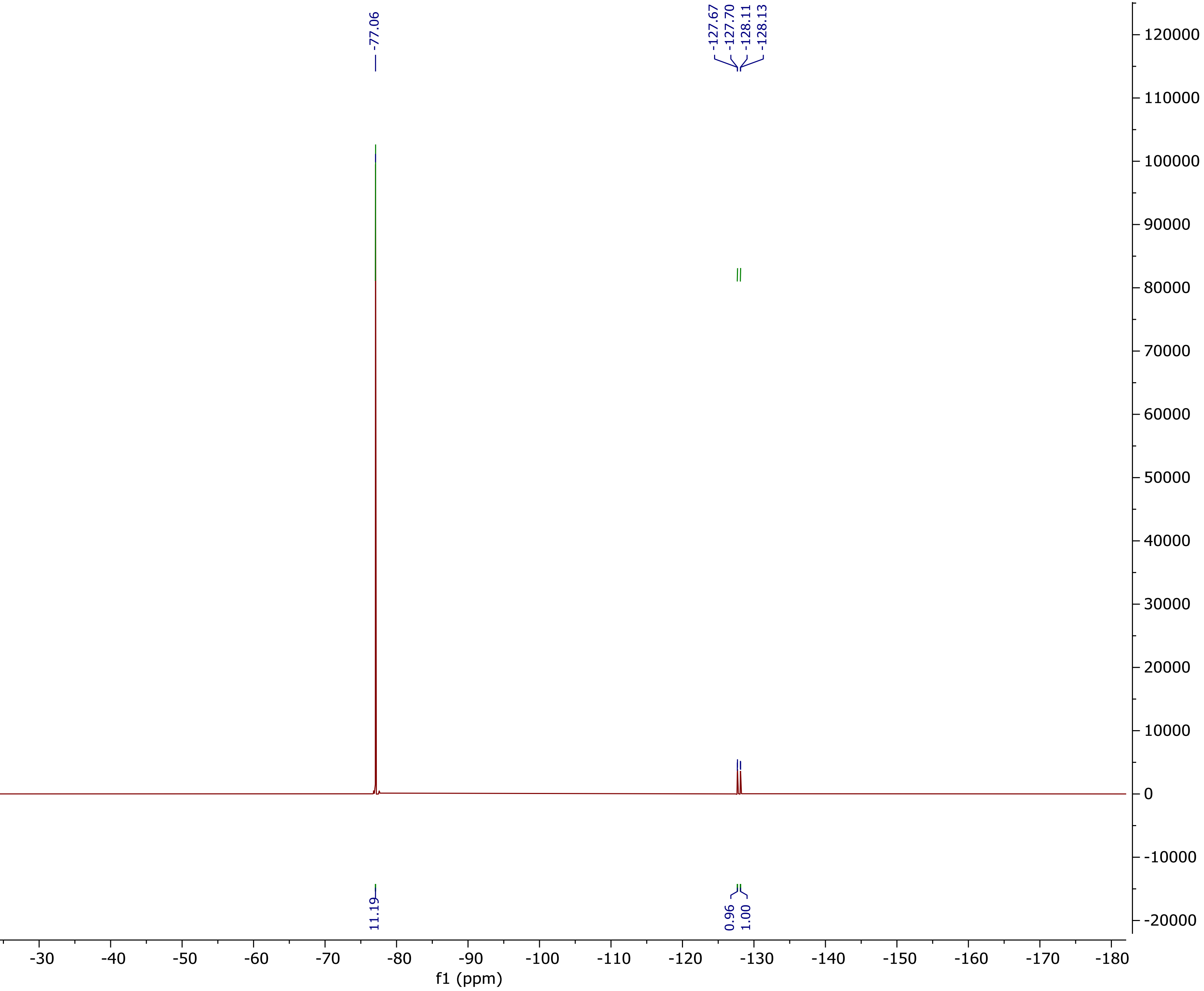

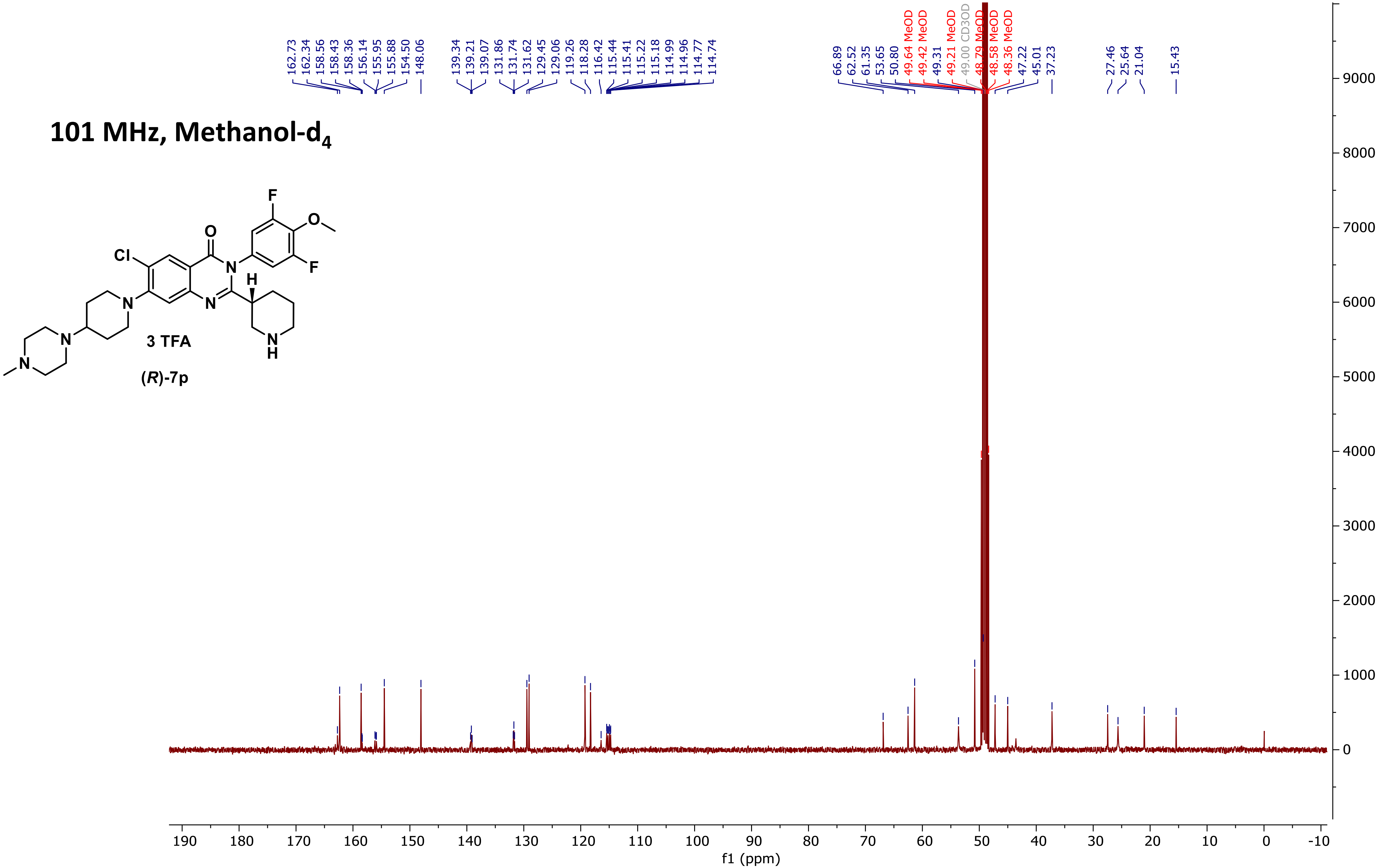
101 MHz, Methanol-d4
3 TFA
(R)-7p
162.73
162.34
158.56
158.43
158.36
156.14
155.95
155.88
154.50
148.06
139.34
139.21
139.07
131.86
131.74
131.62
129.45
129.06
119.26
118.28
116.42
115.44
115.41
115.22
115.18
114.99
114.96
114.77
114.74
66.89
62.52
61.35
53.65
50.80
49.64 MeOD
49.42 MeOD
49.31
49.21 MeOD
49.00 CD3OD
48.79 MeOD
48.58 MeOD
48.36 MeOD
47.22
45.01
37.23
27.46
25.64
21.04
15.43
f1 (ppm)

376 MHz, Methanol-d$_4$

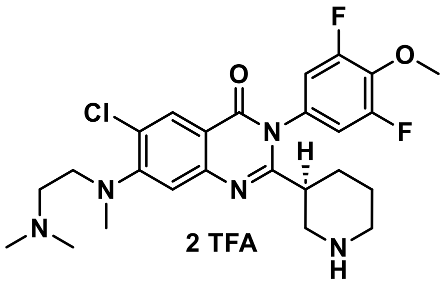

(*S*)-7q

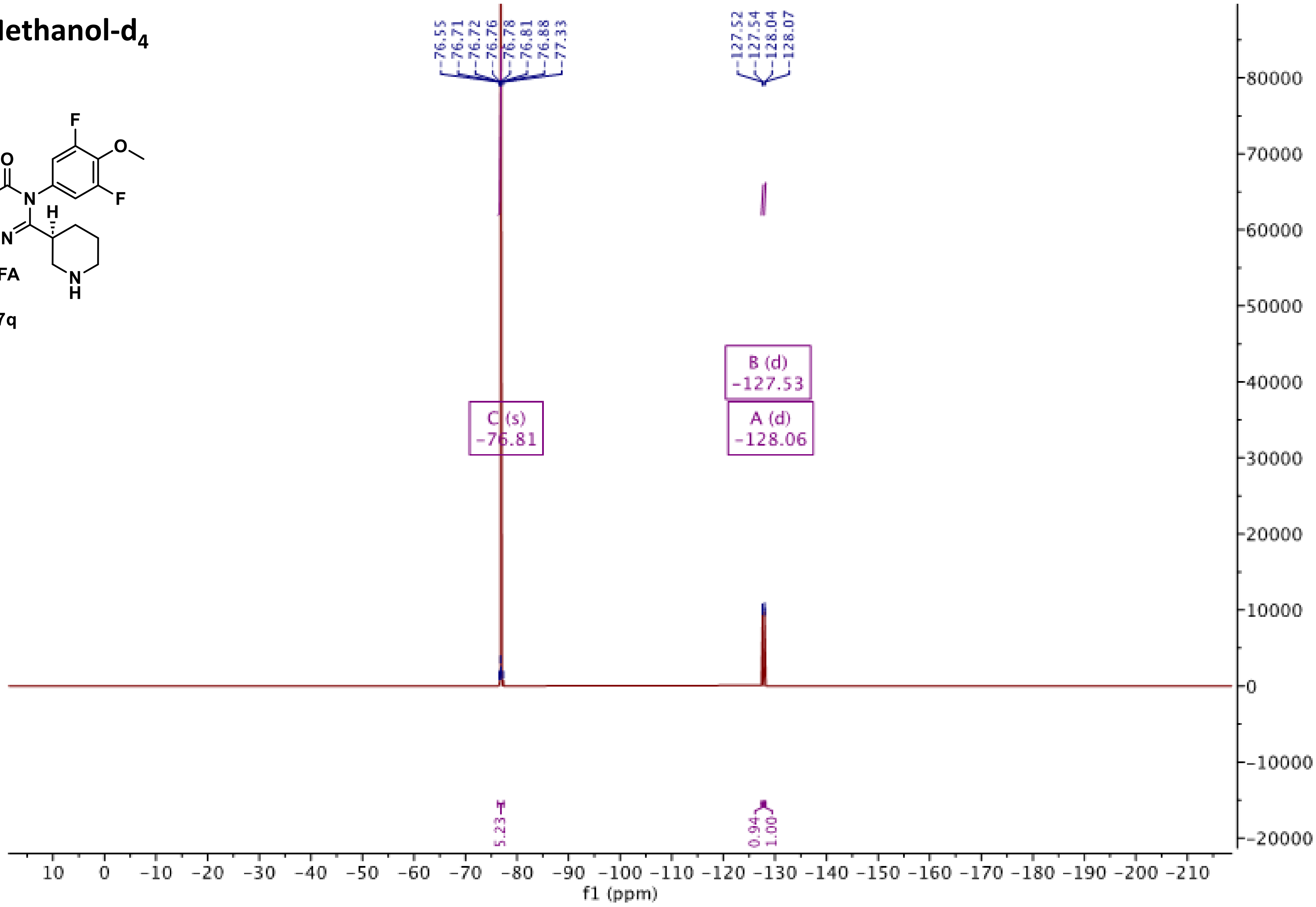

101 MHz, Methanol-d$_4$

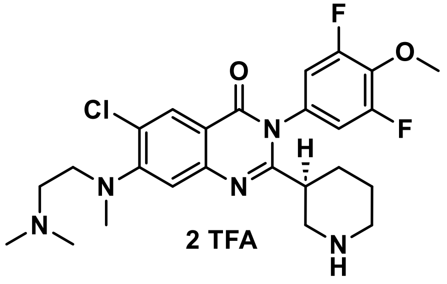

(*S*)-7q

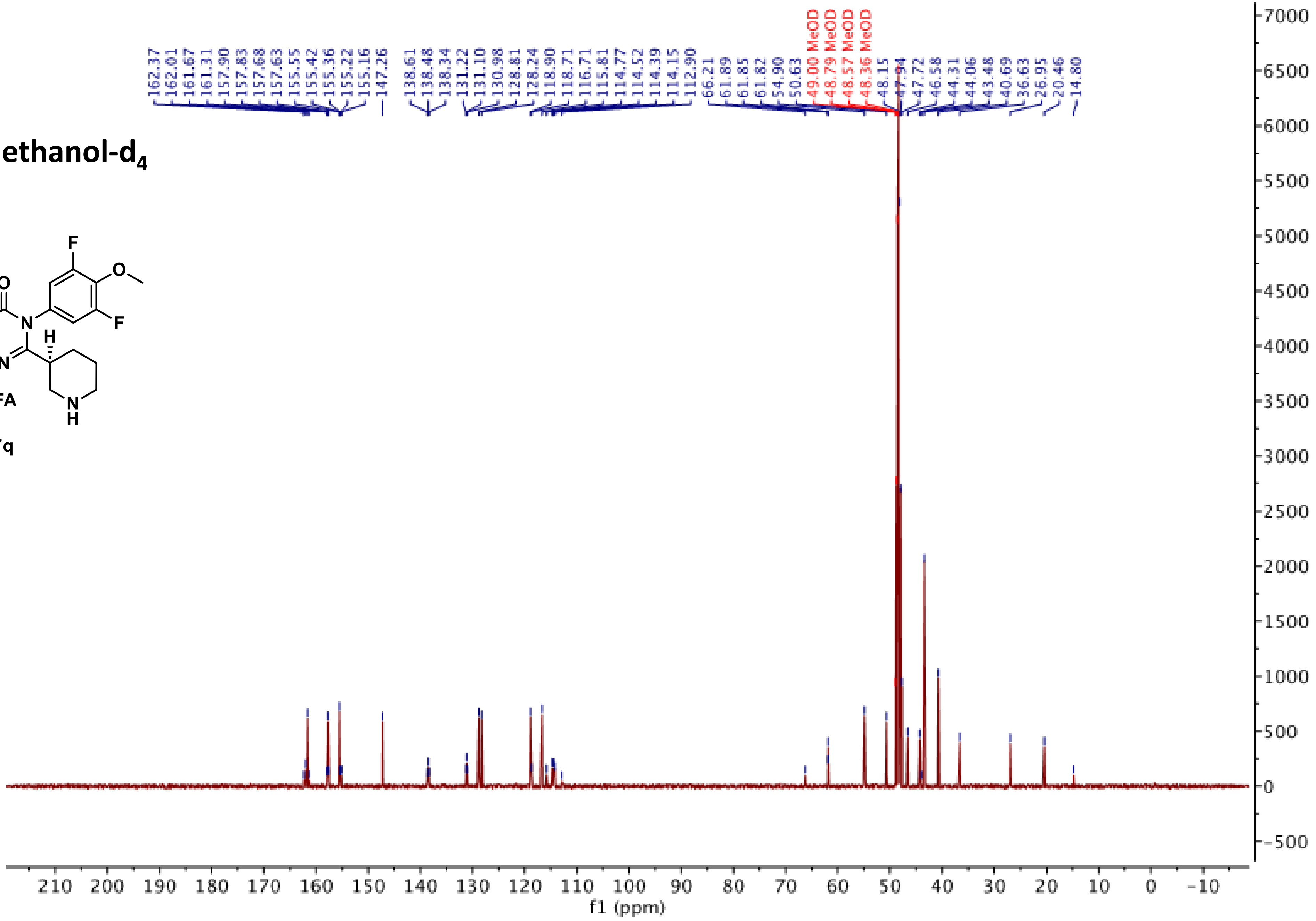

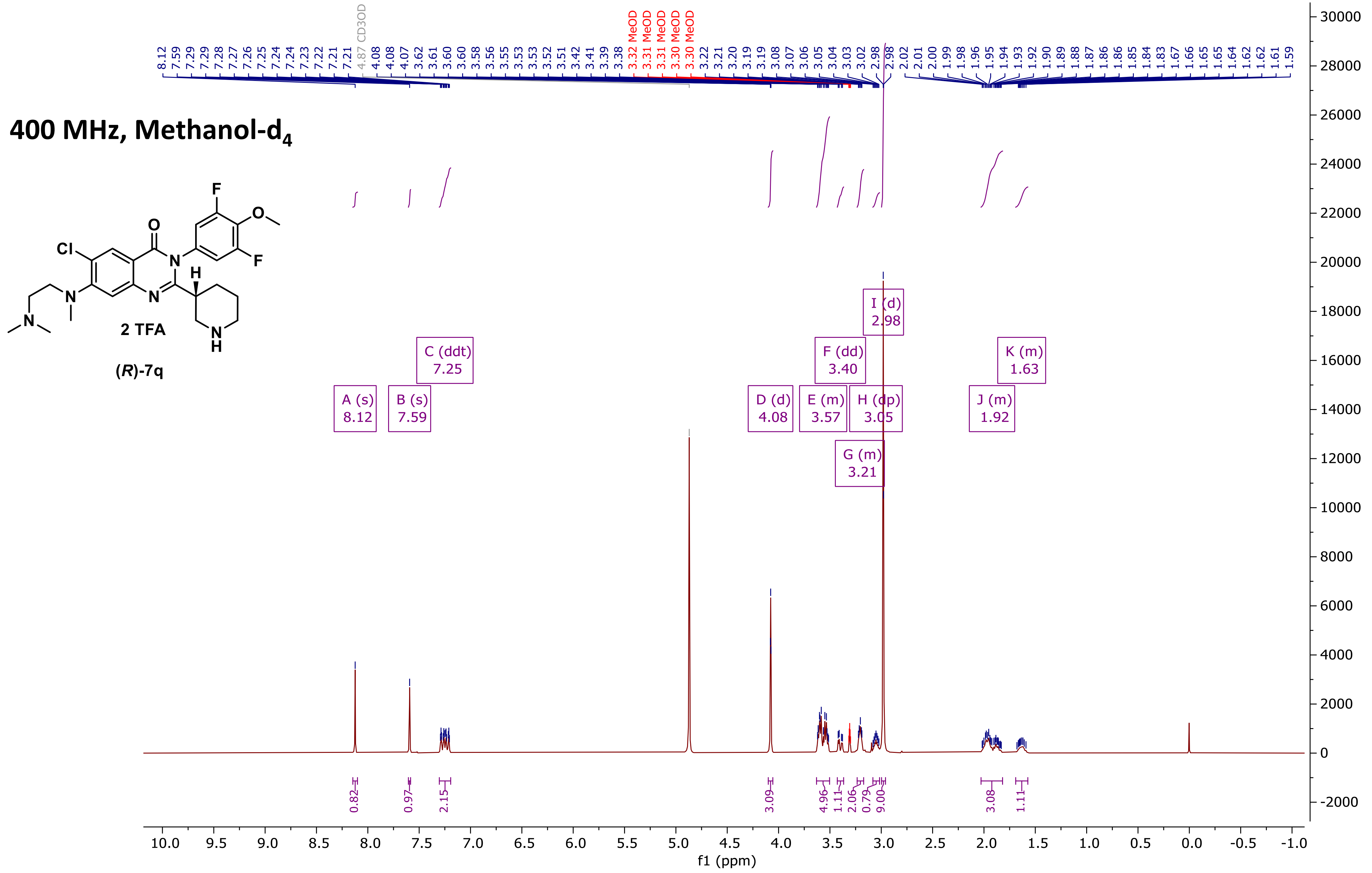

400 MHz, Methanol-d4
Cl
N
O
F
O
F
H
N
N
N
N
H
2 TFA
(R)-7q
A (s) 8.12
B (s) 7.59
C (ddt) 7.25
D (d) 4.08
E (m) 3.57
F (dd) 3.40
G (m) 3.21
H (dp) 3.05
I (d) 2.98
J (m) 1.92
K (m) 1.63
f1 (ppm)

376 MHz, Methanol-d_4

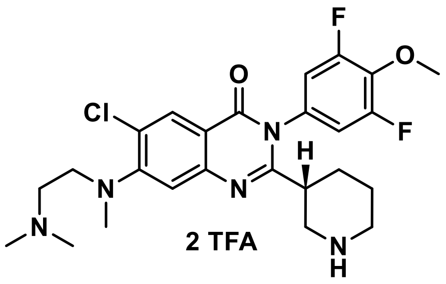

(*R*)-7q

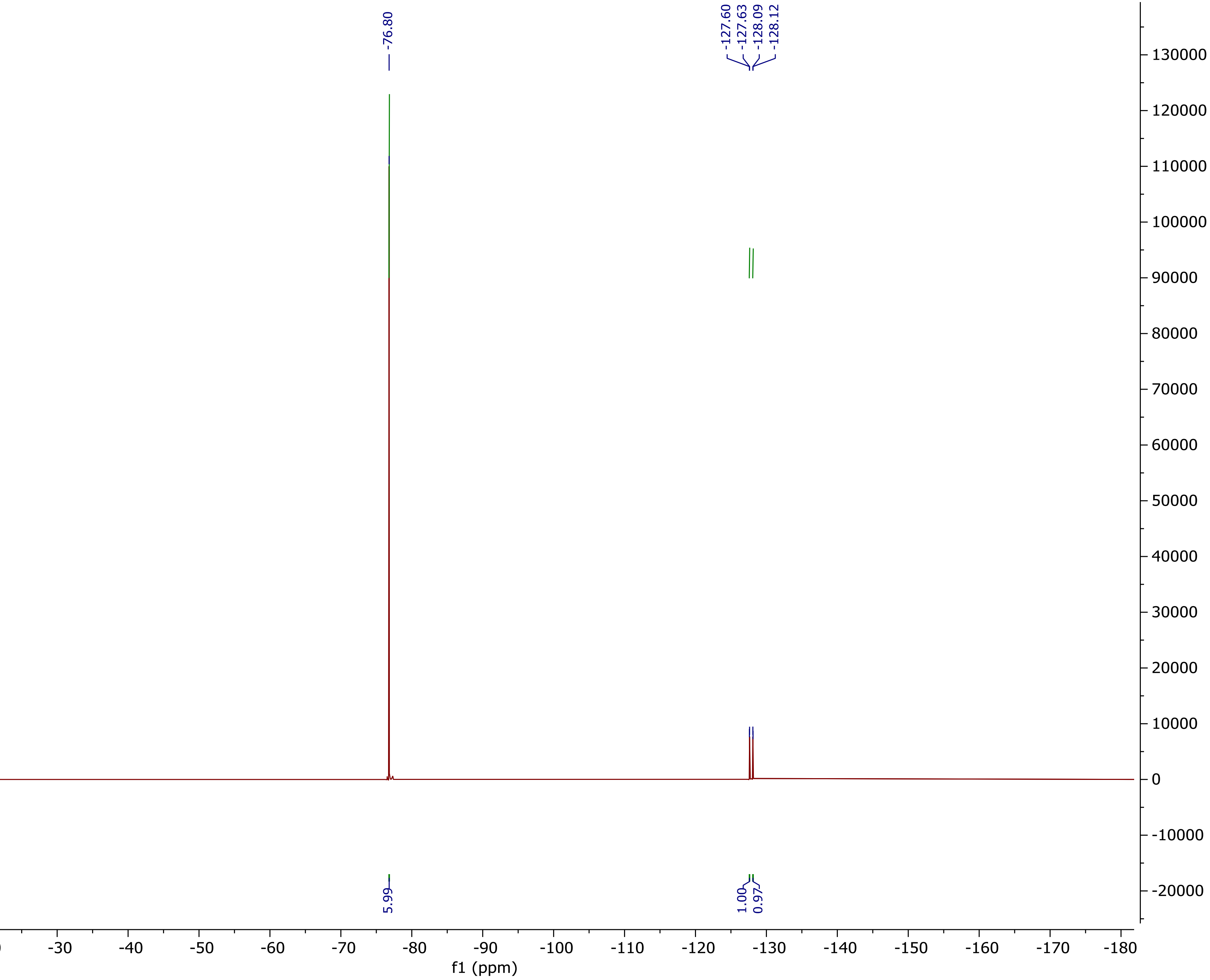

101 MHz, Methanol-d_4

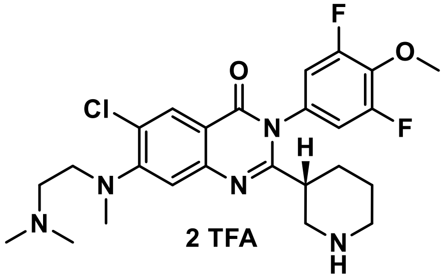

(*R*)-7q

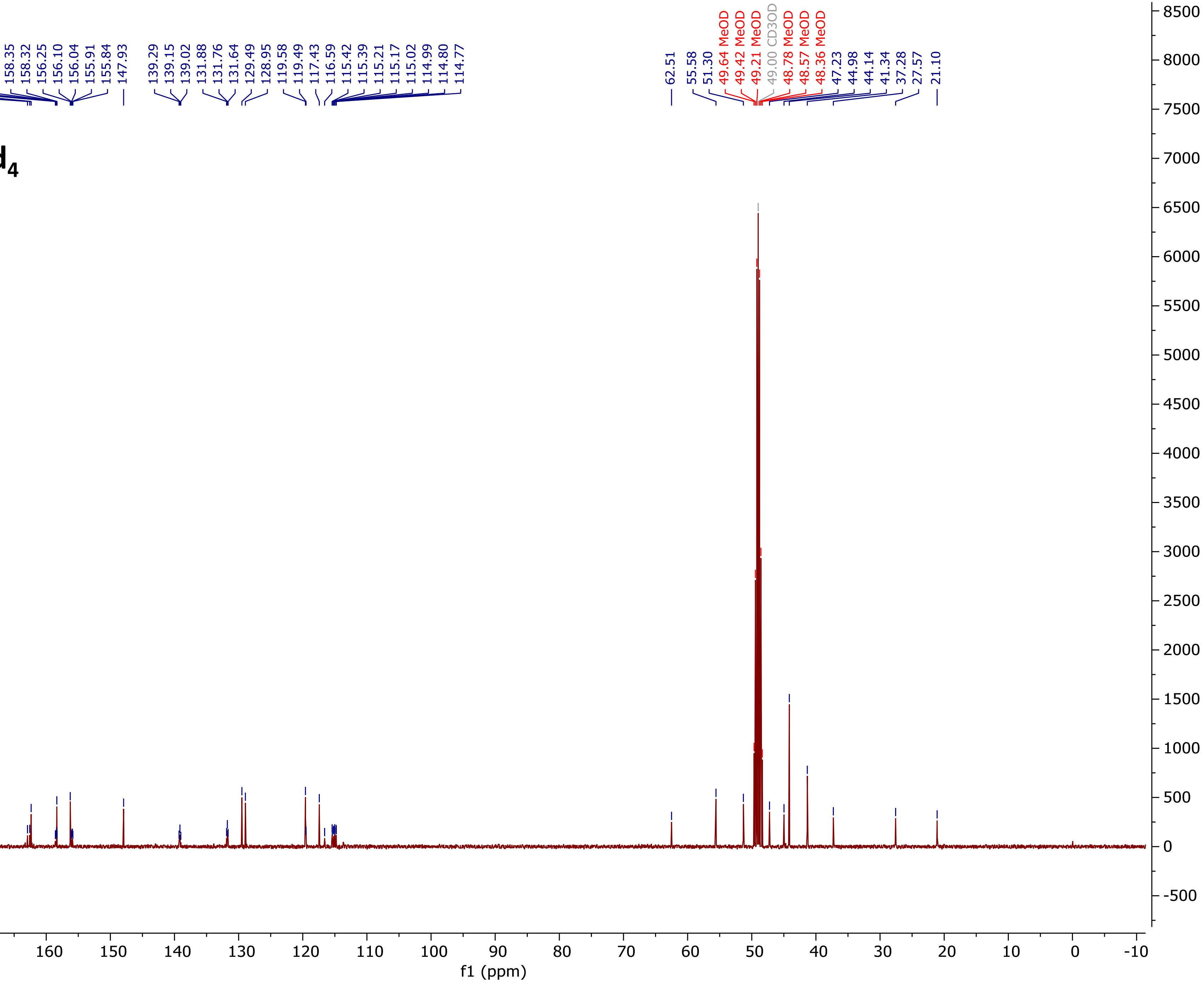

400 MHz, Methanol-d_4

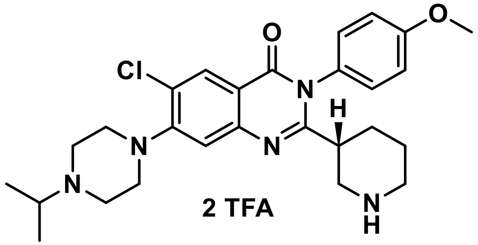

(*R*)-7t

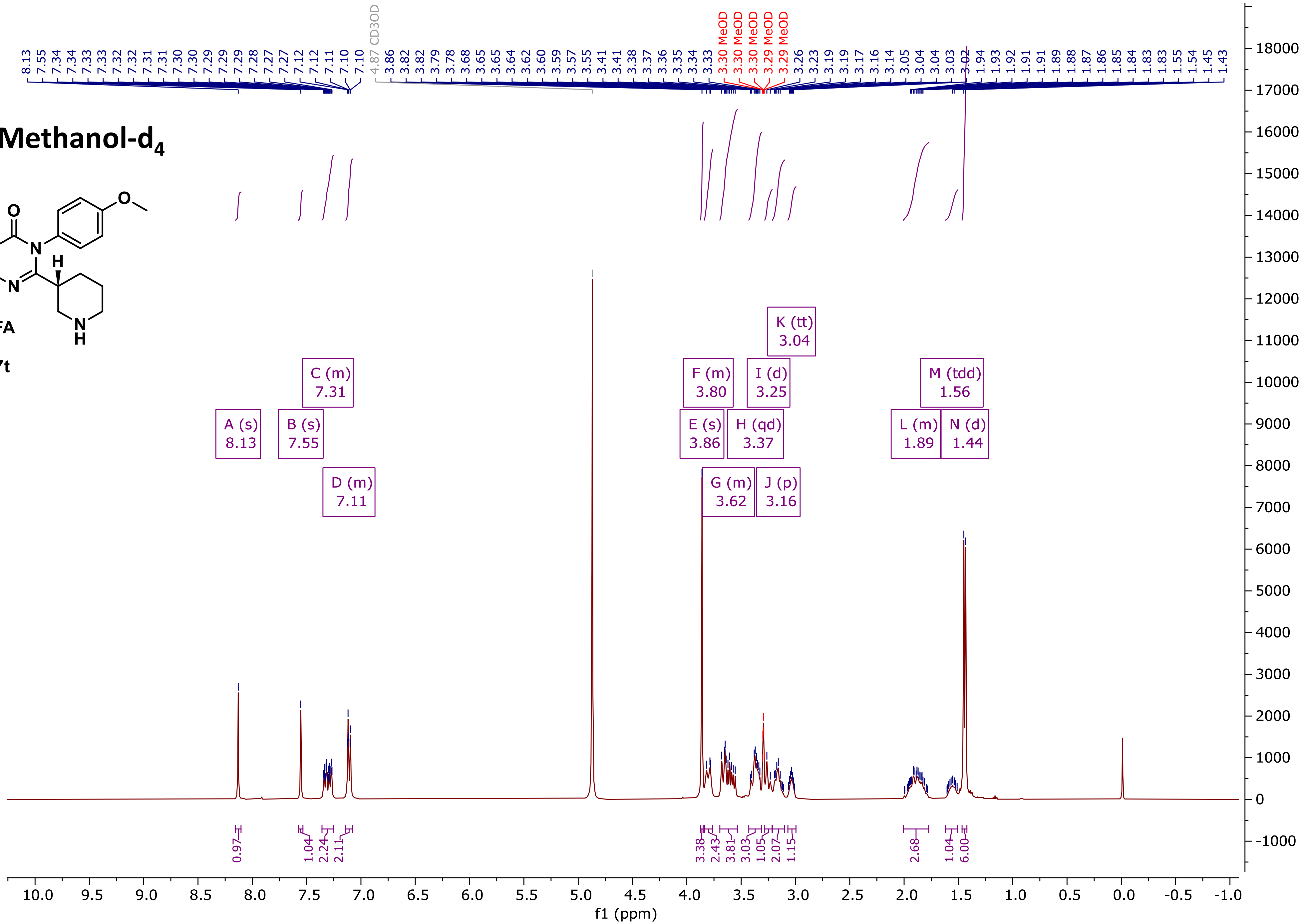

376 MHz, Methanol-d_4

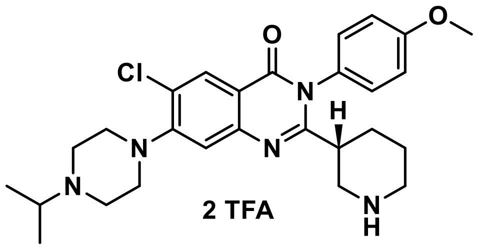

(*R*)-7t

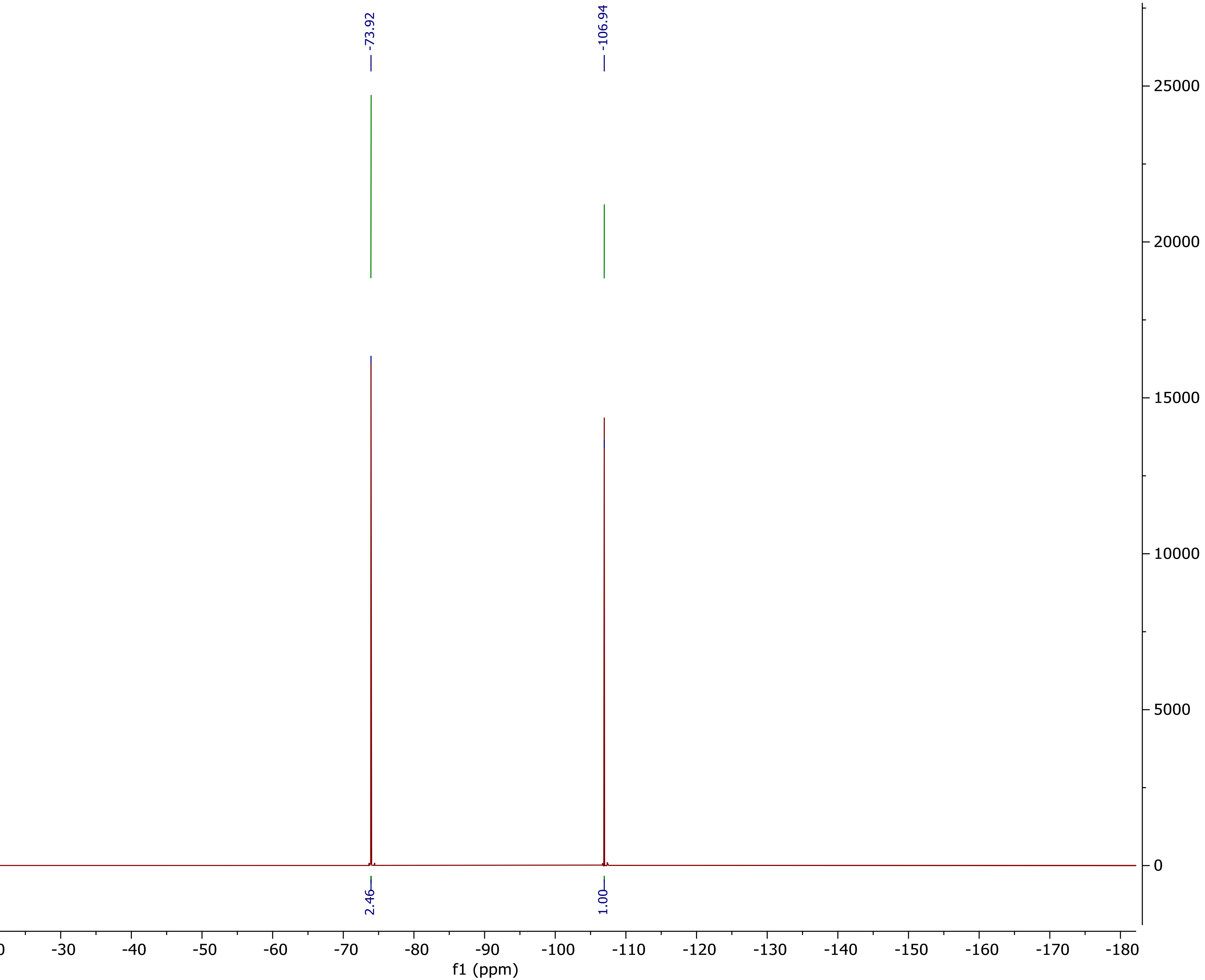

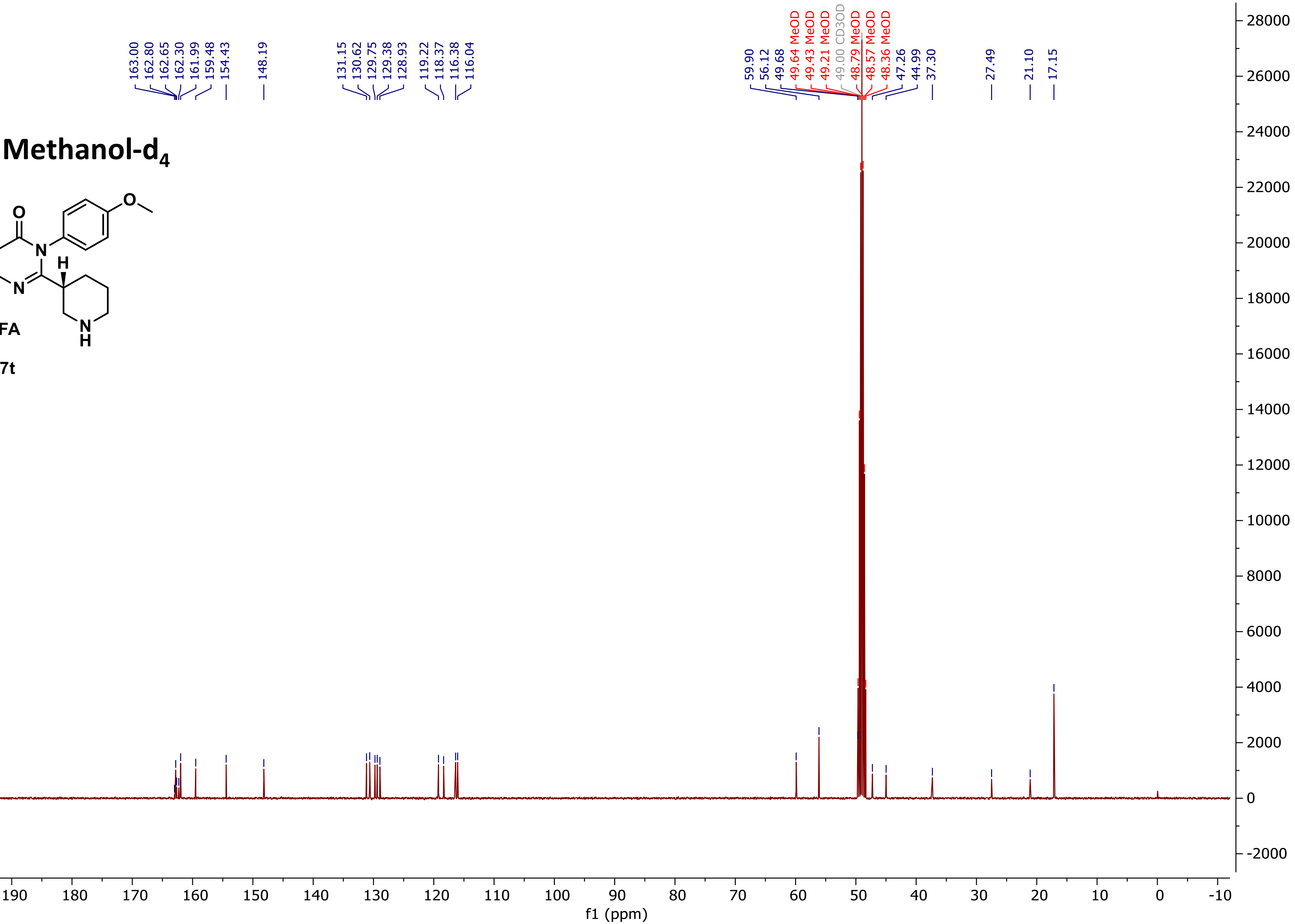

101 MHz, Methanol-d_4

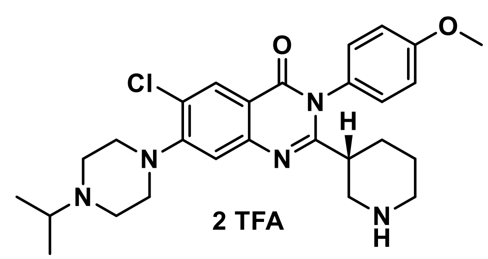

(*R*)-7t

400 MHz, Methanol-d$_4$

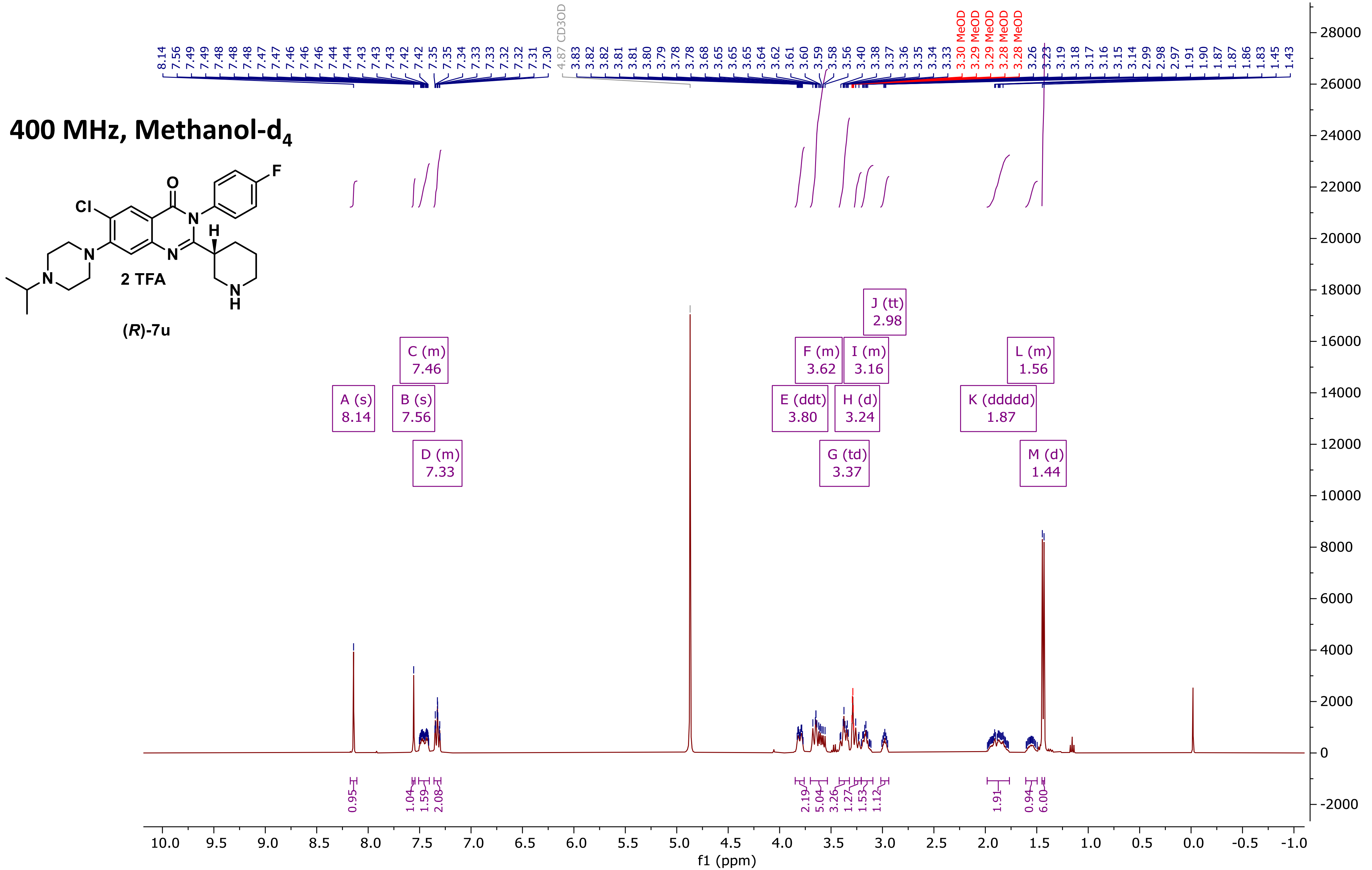

376 MHz, Methanol-d_4

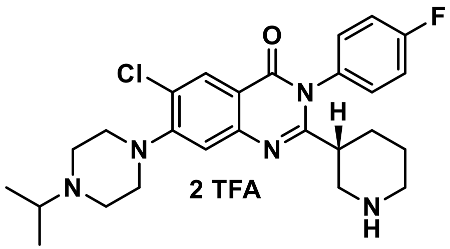

(*R*)-7u

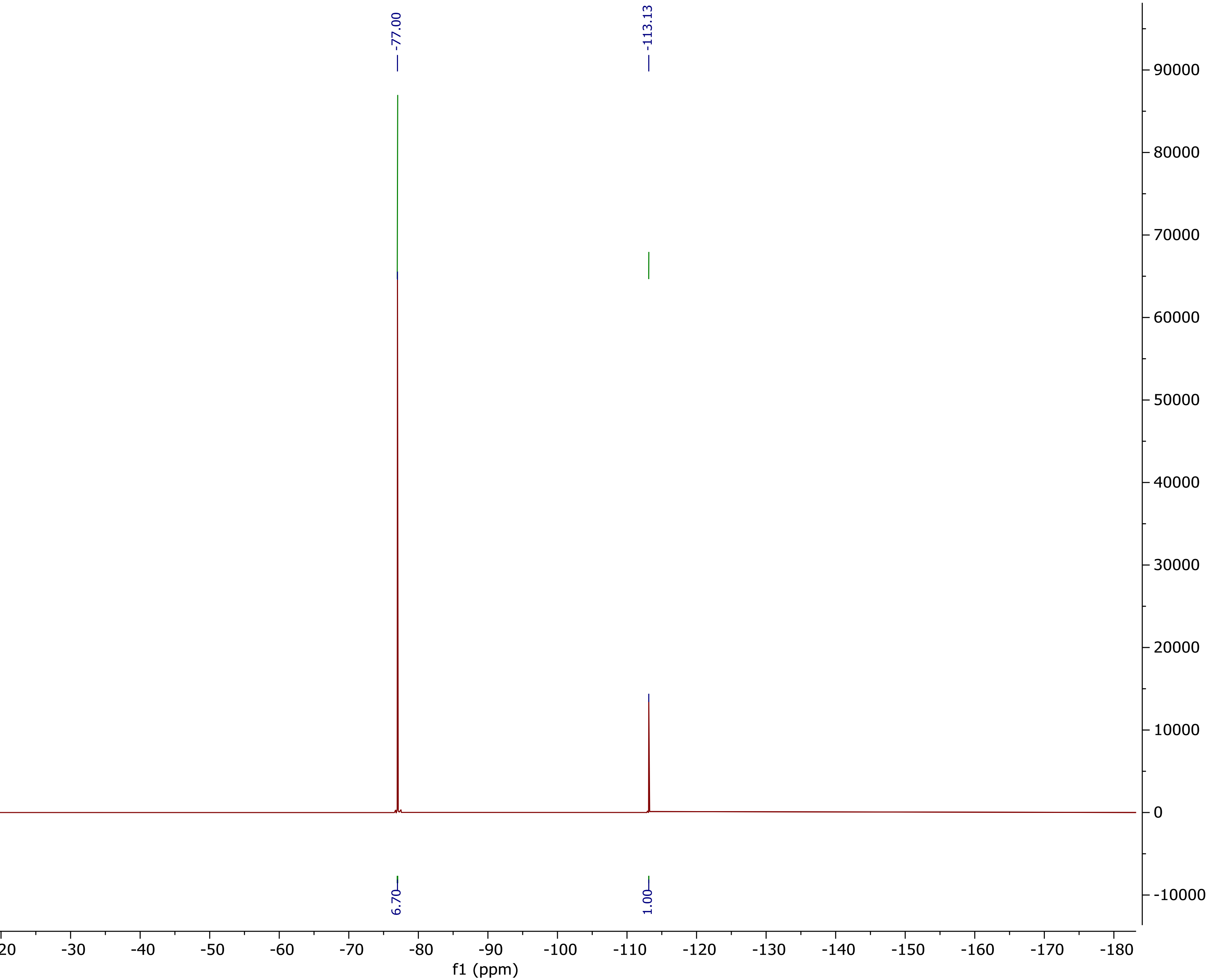

101 MHz, Methanol-d_4

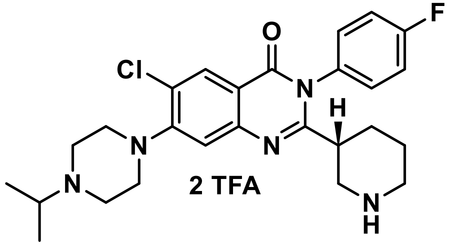

(*R*)-7u

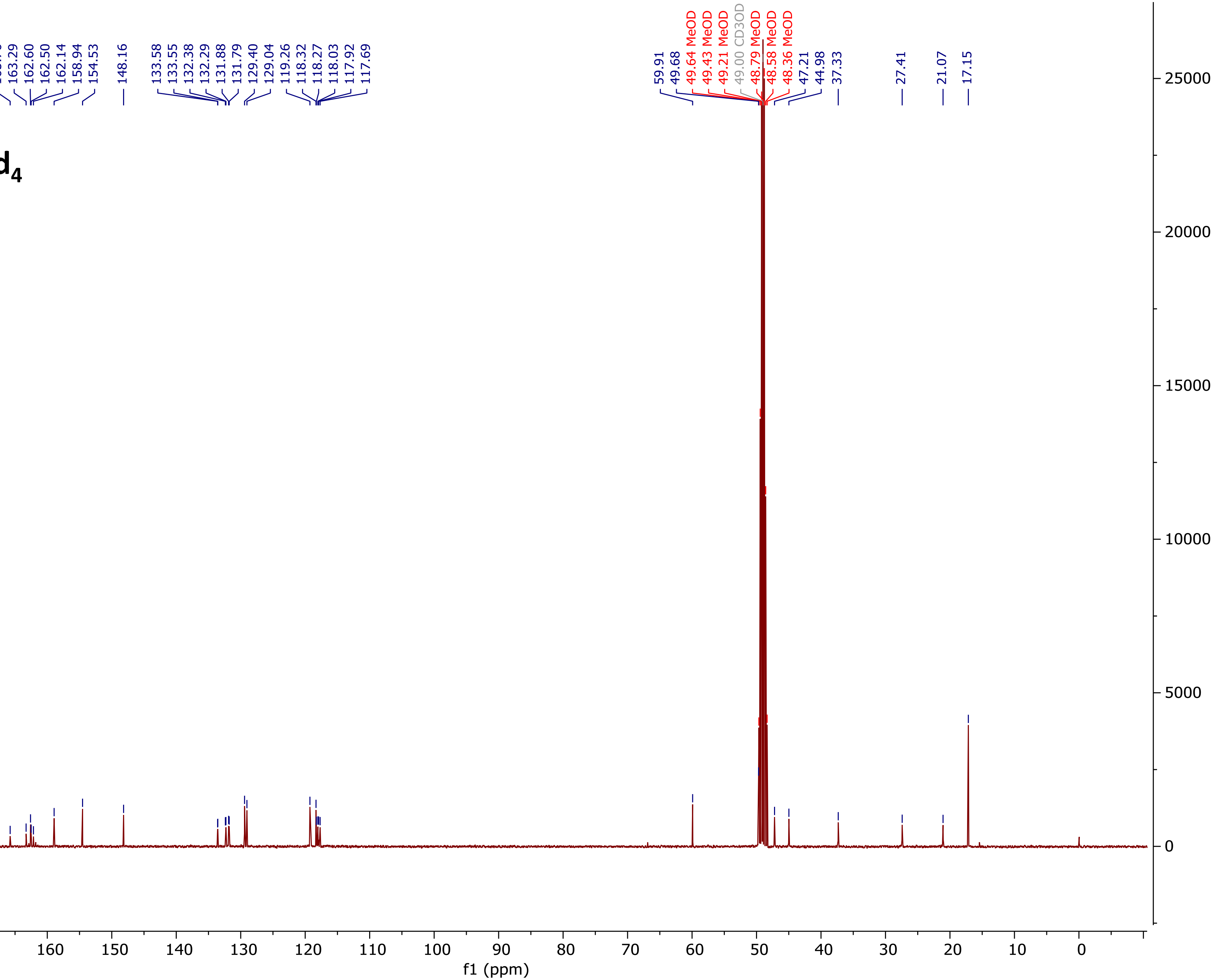

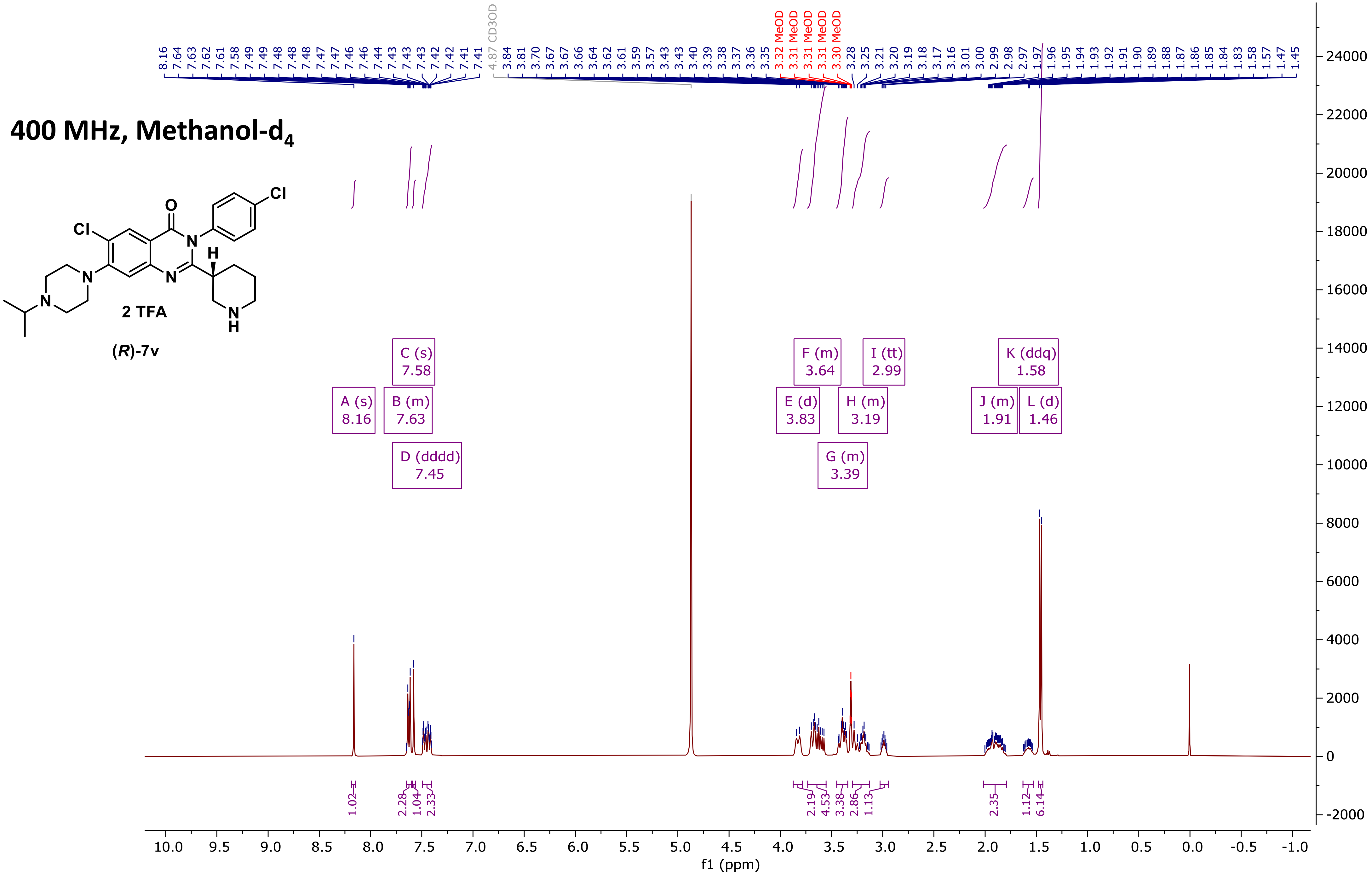
400 MHz, Methanol-d4
2 TFA
(R)-7v
A (s) 8.16
B (m) 7.63
C (s) 7.58
D (dddd) 7.45
E (d) 3.83
F (m) 3.64
G (m) 3.39
H (m) 3.19
I (tt) 2.99
J (m) 1.91
K (ddq) 1.58
L (d) 1.46
f1 (ppm)

376 MHz, Methanol-d_4

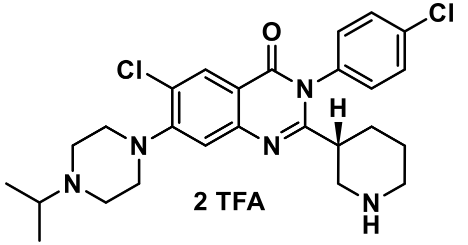

(*R*)-7v

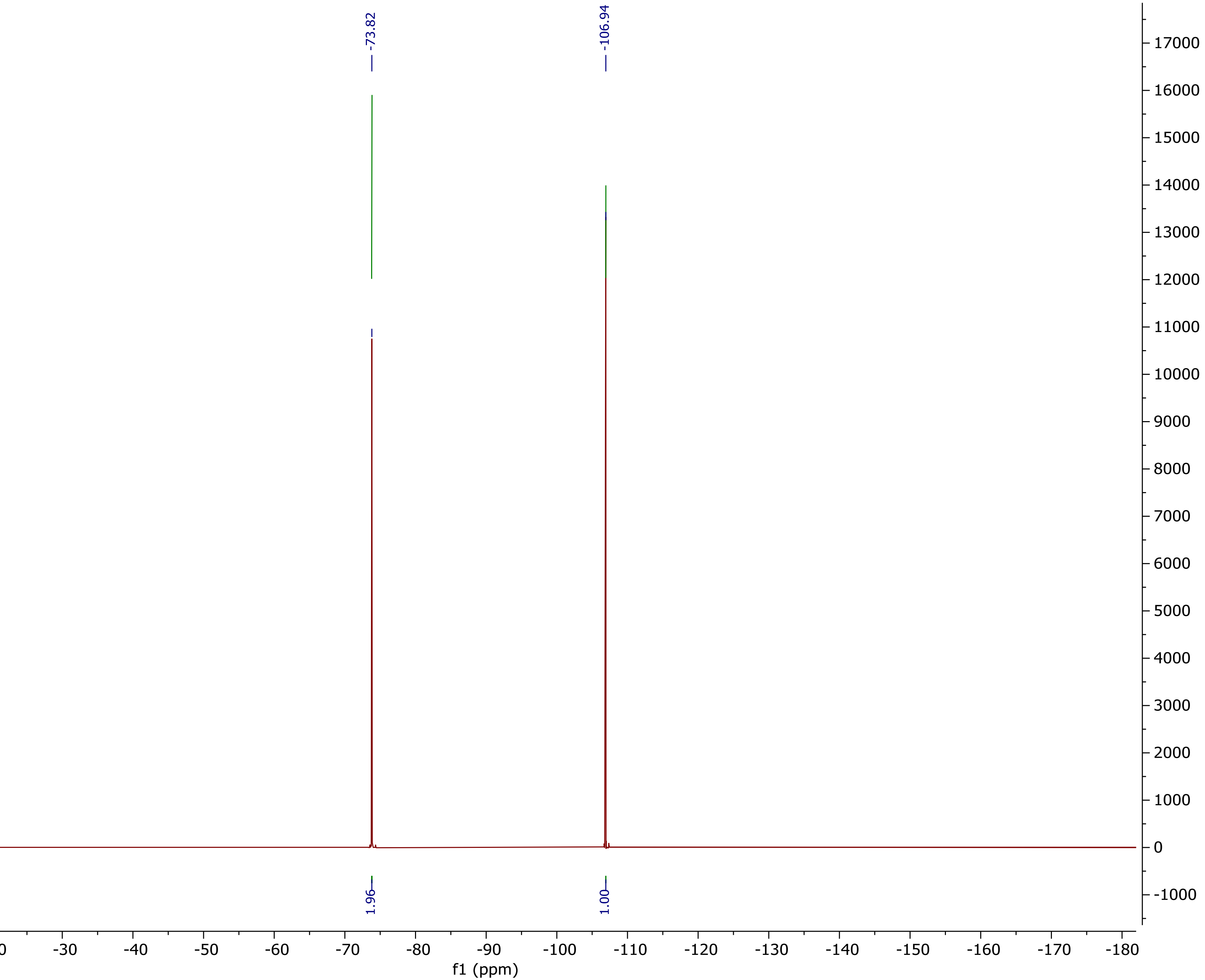

101 MHz, Methanol-d_4

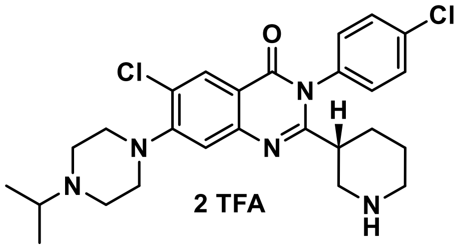

(*R*)-7v

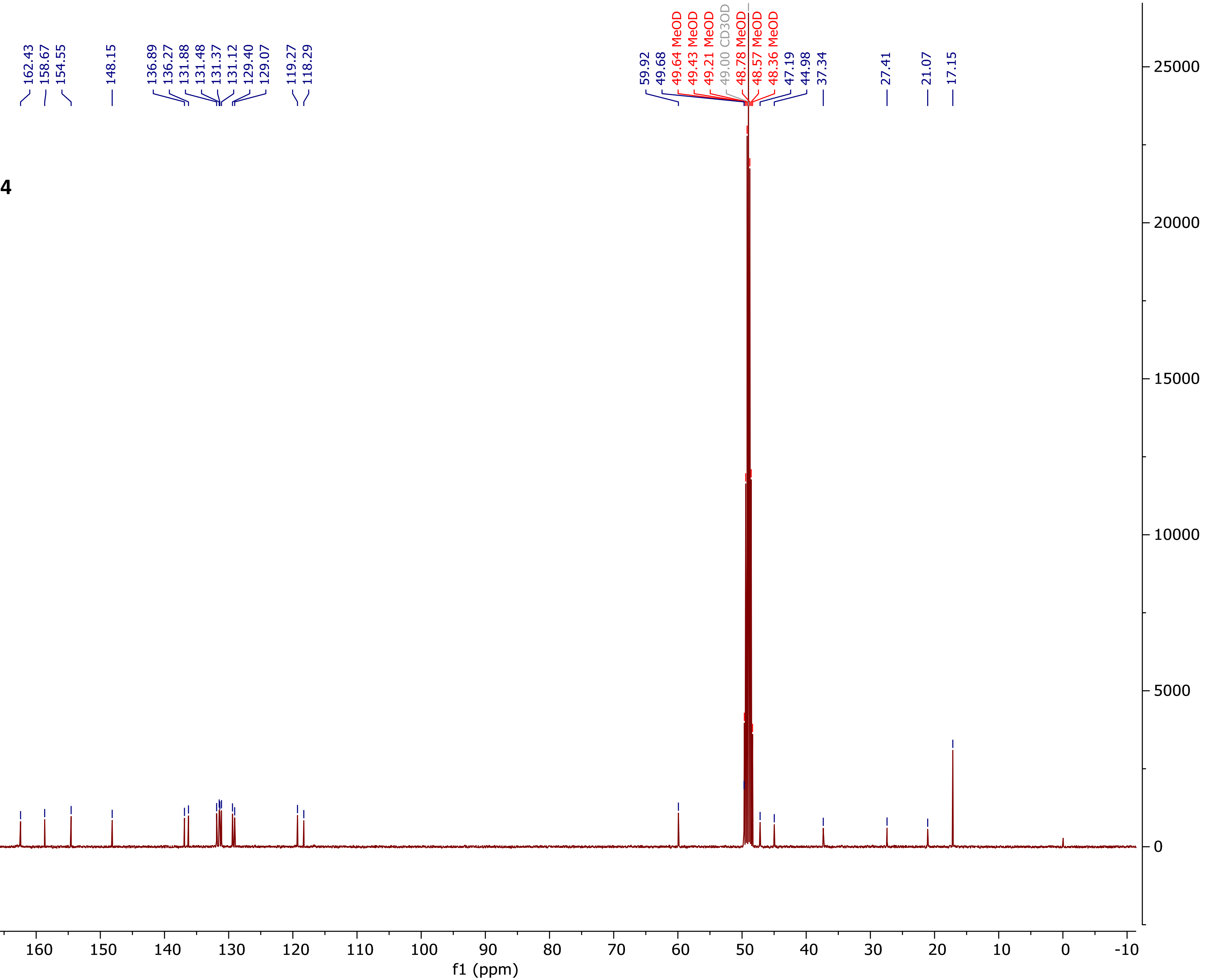

400 MHz, $CDCl_3$

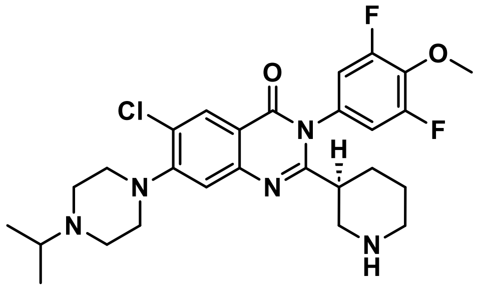

(*S*)-1g

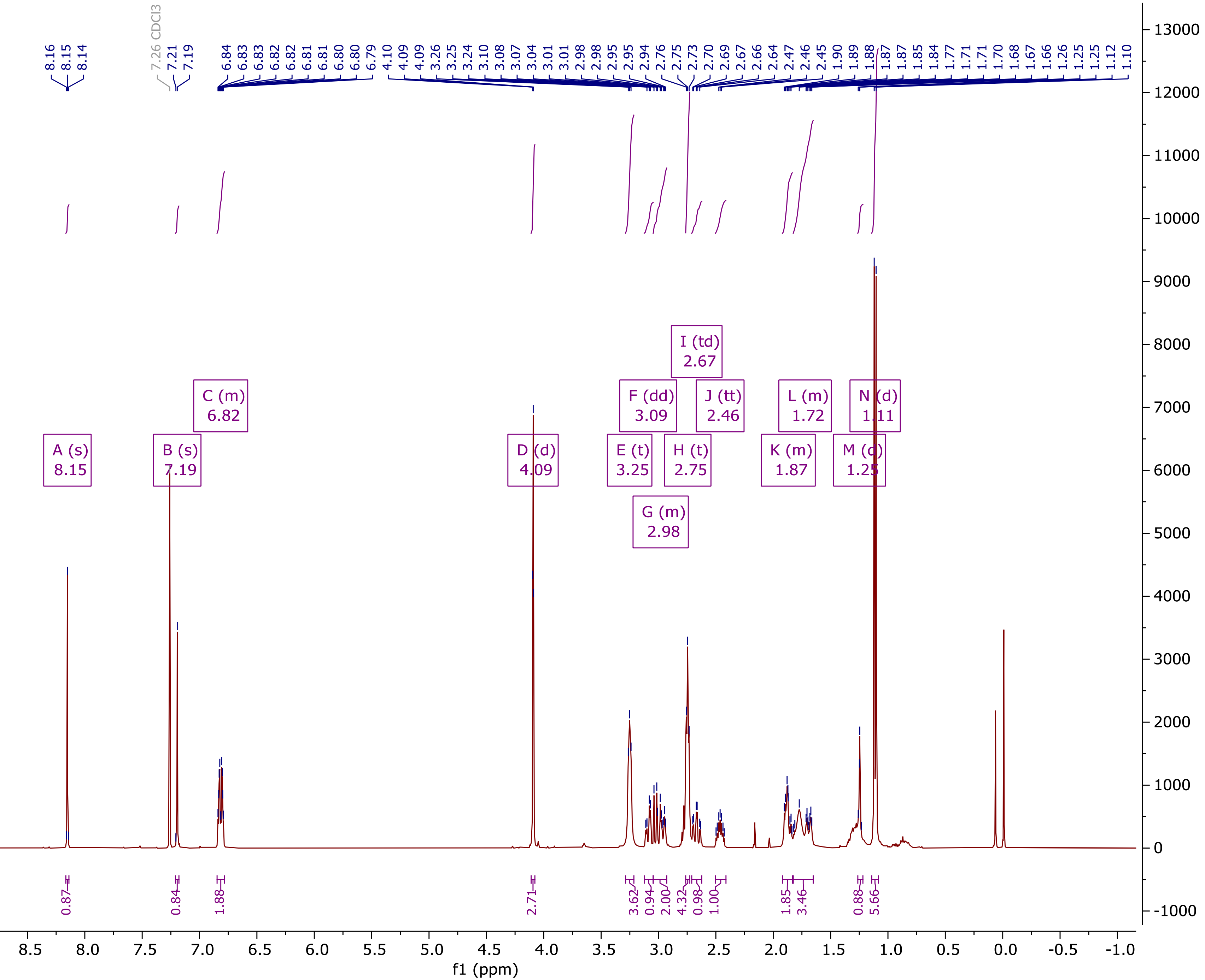

101 MHz, $CDCl_3$

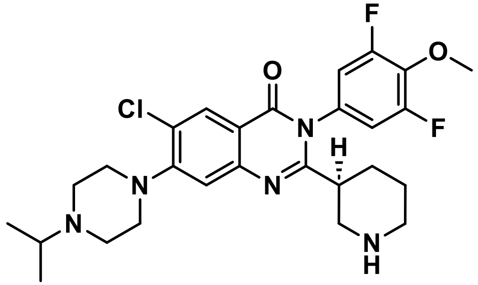

(*S*)-1g

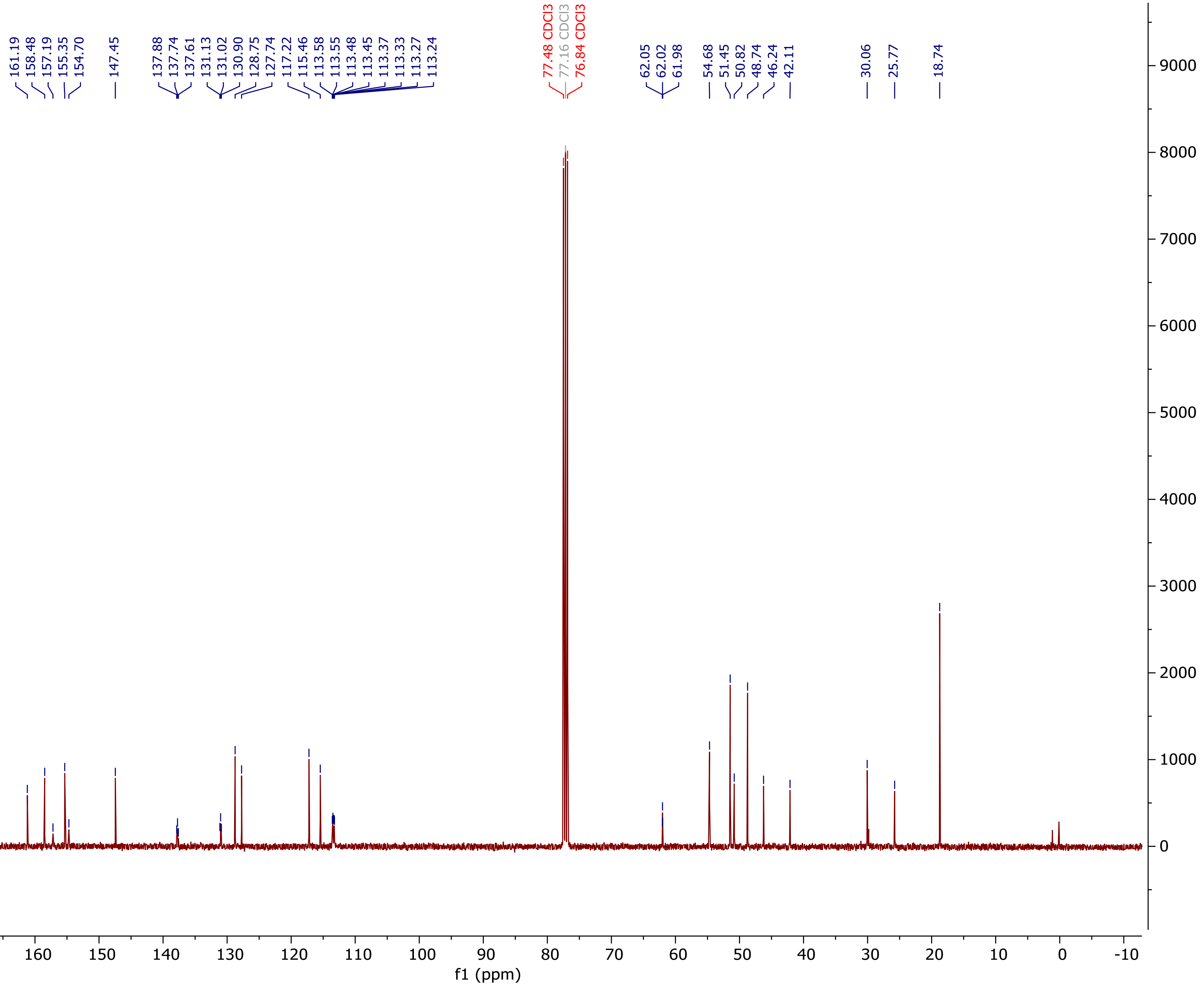

400 MHz, $CDCl_3$

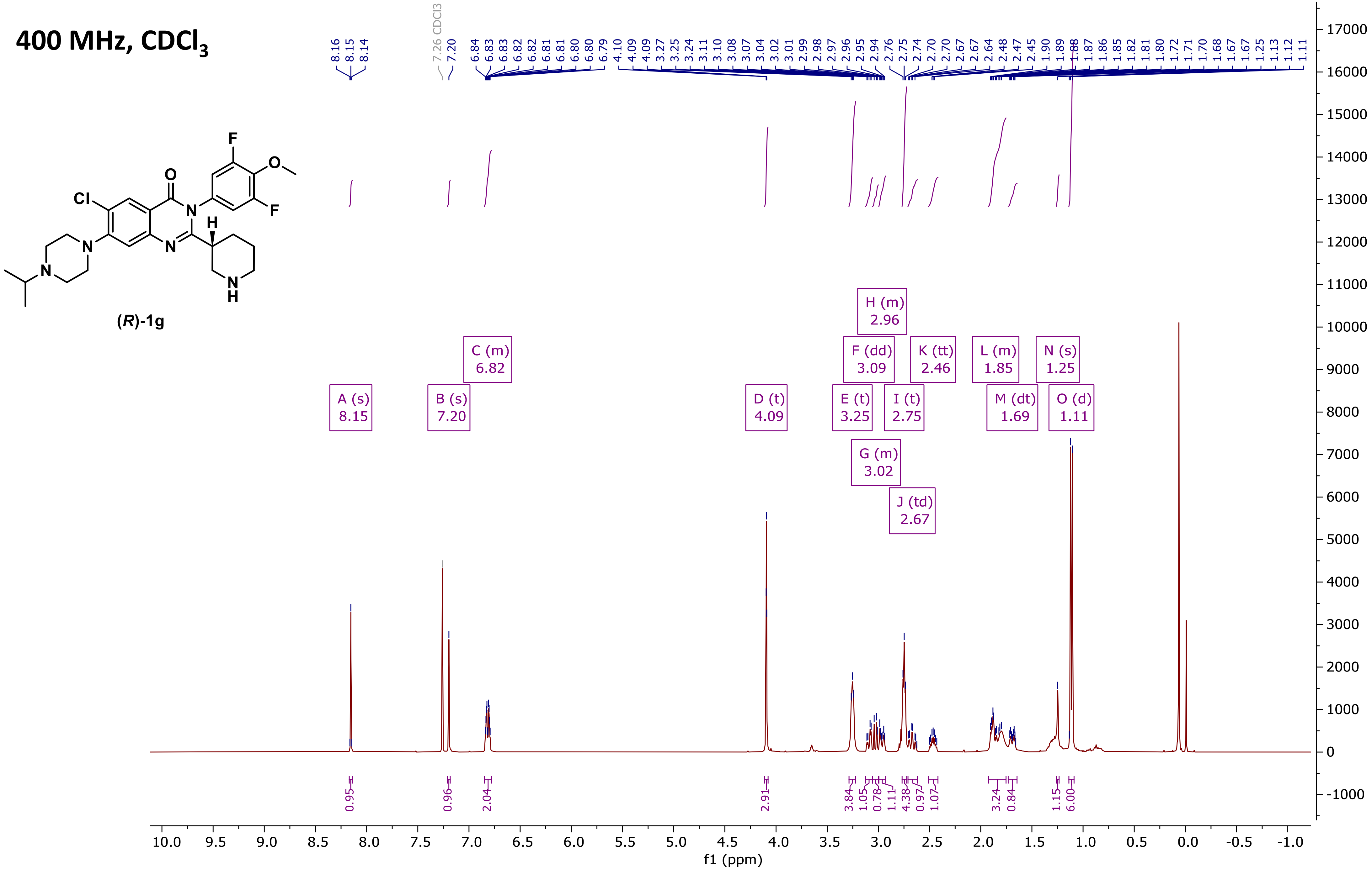

101 MHz, $CDCl_3$

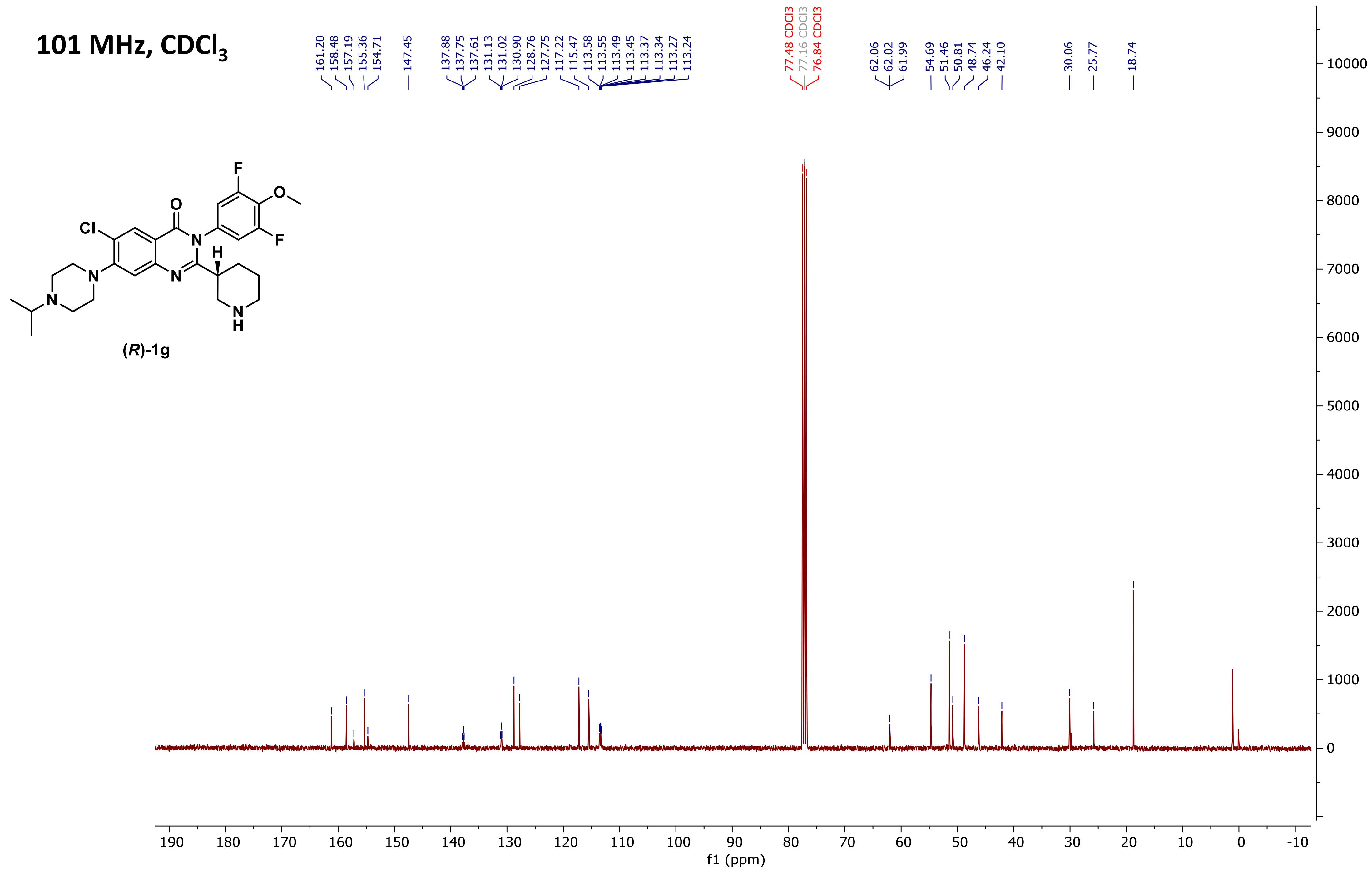

400 MHz, $CDCl_3$

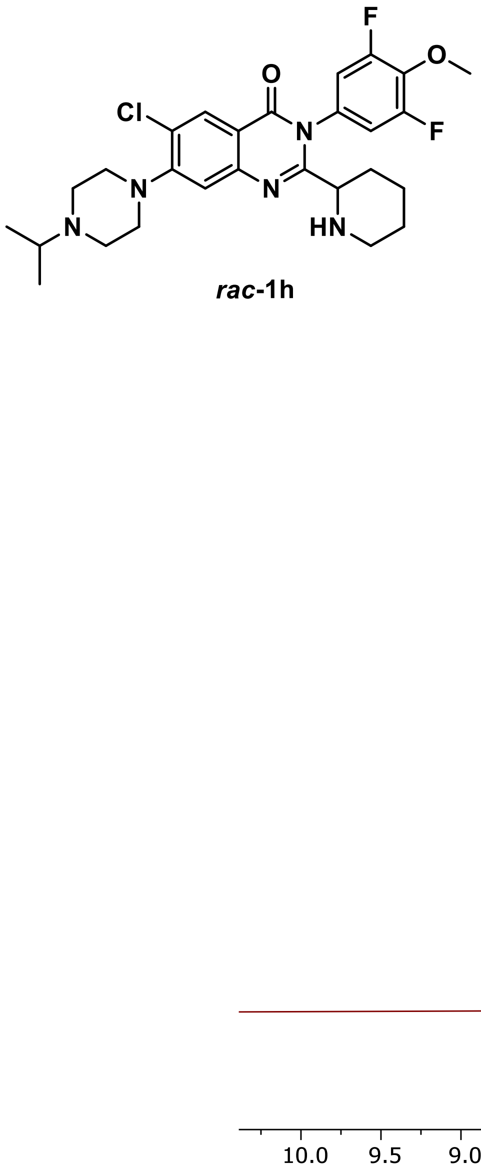

***rac*-1h**

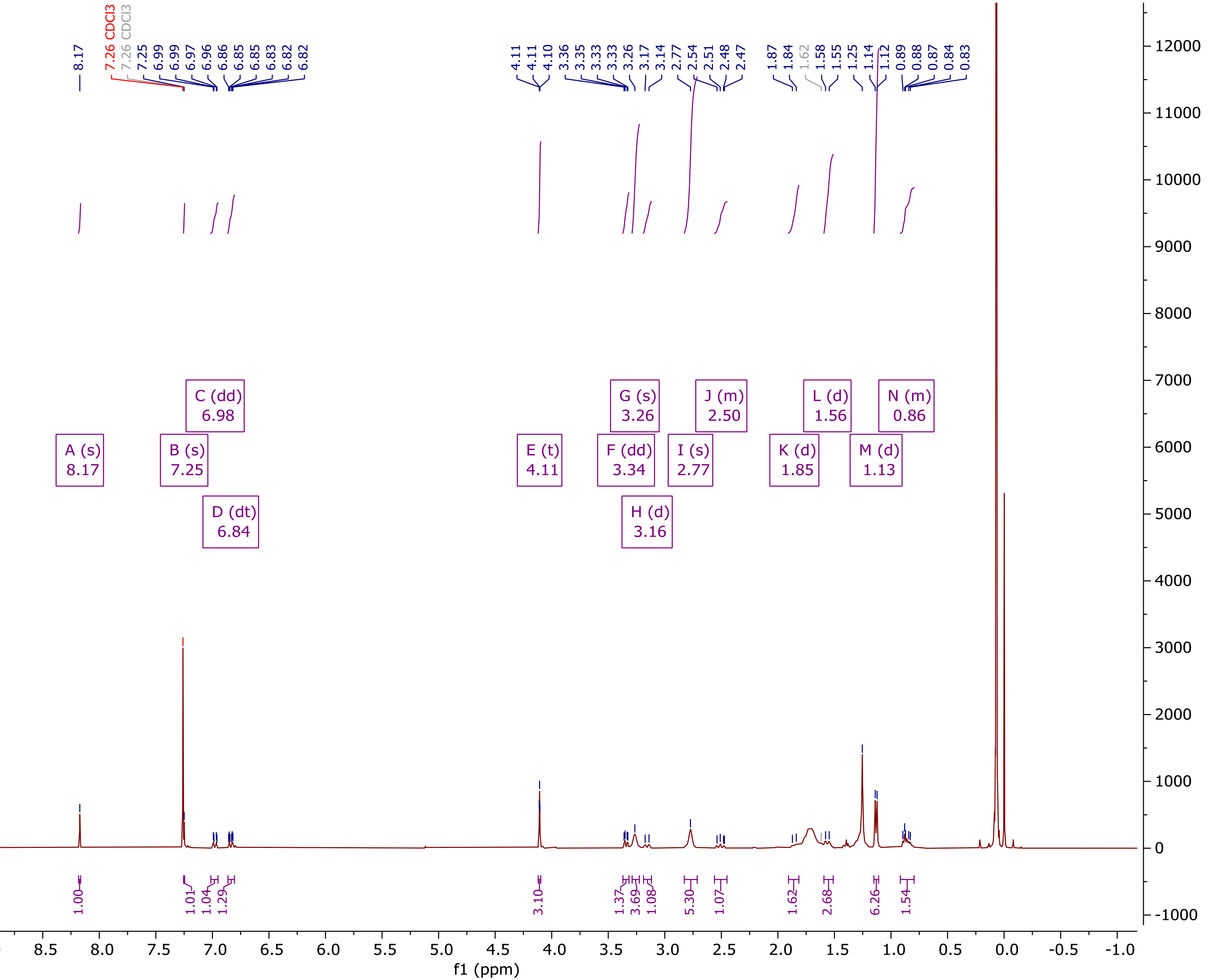

400 MHz, $CDCl_3$

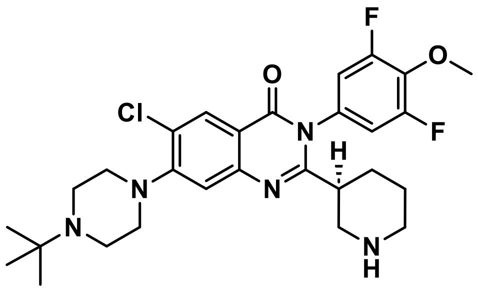

(*S*)-1j

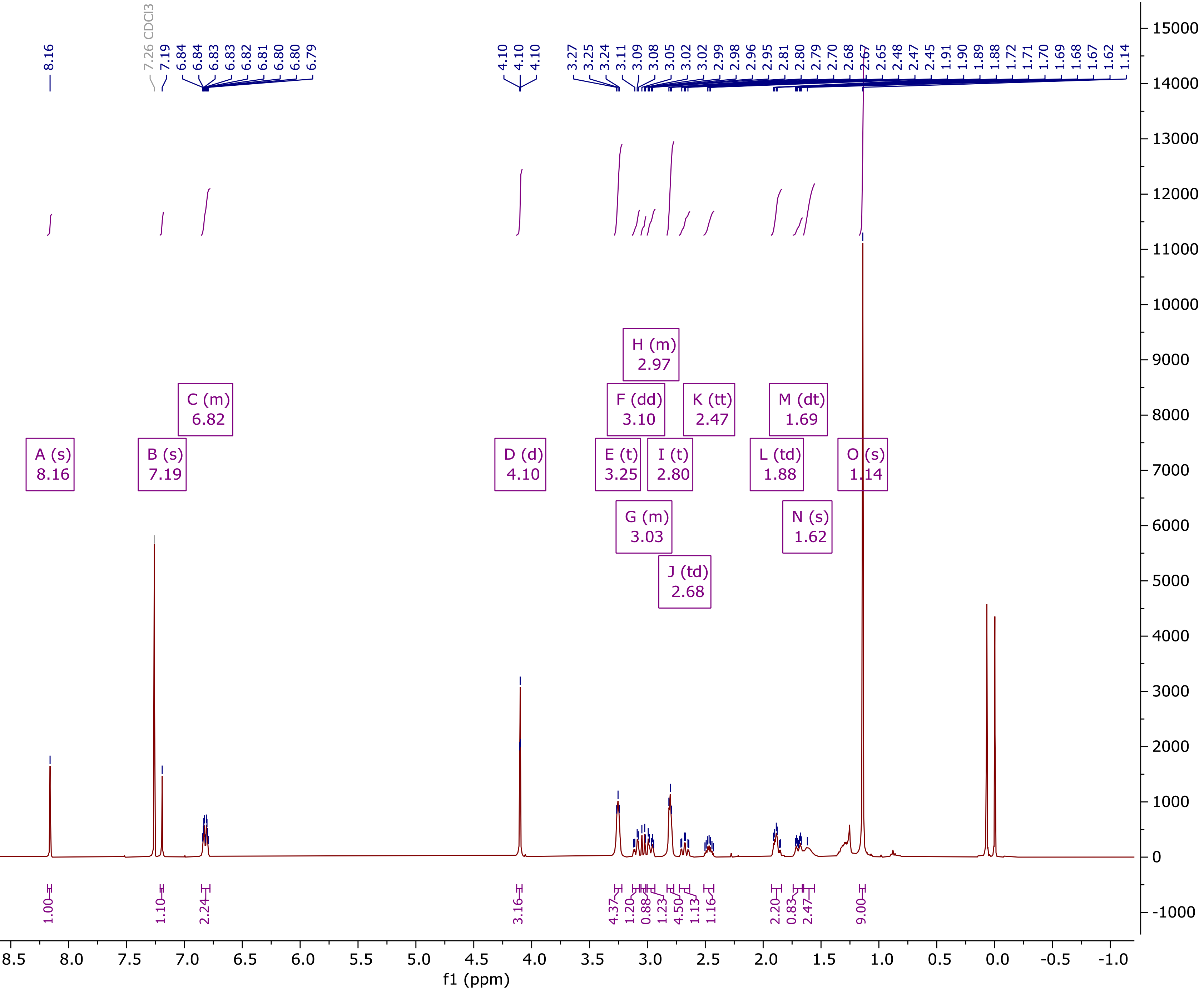

101 MHz, $CDCl_3$

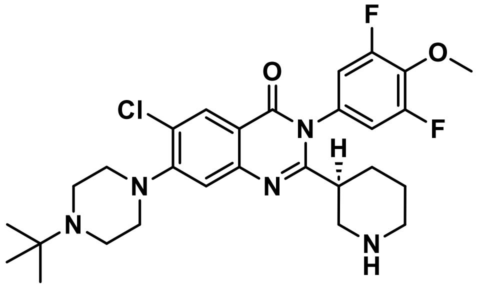

(*S*)-1j

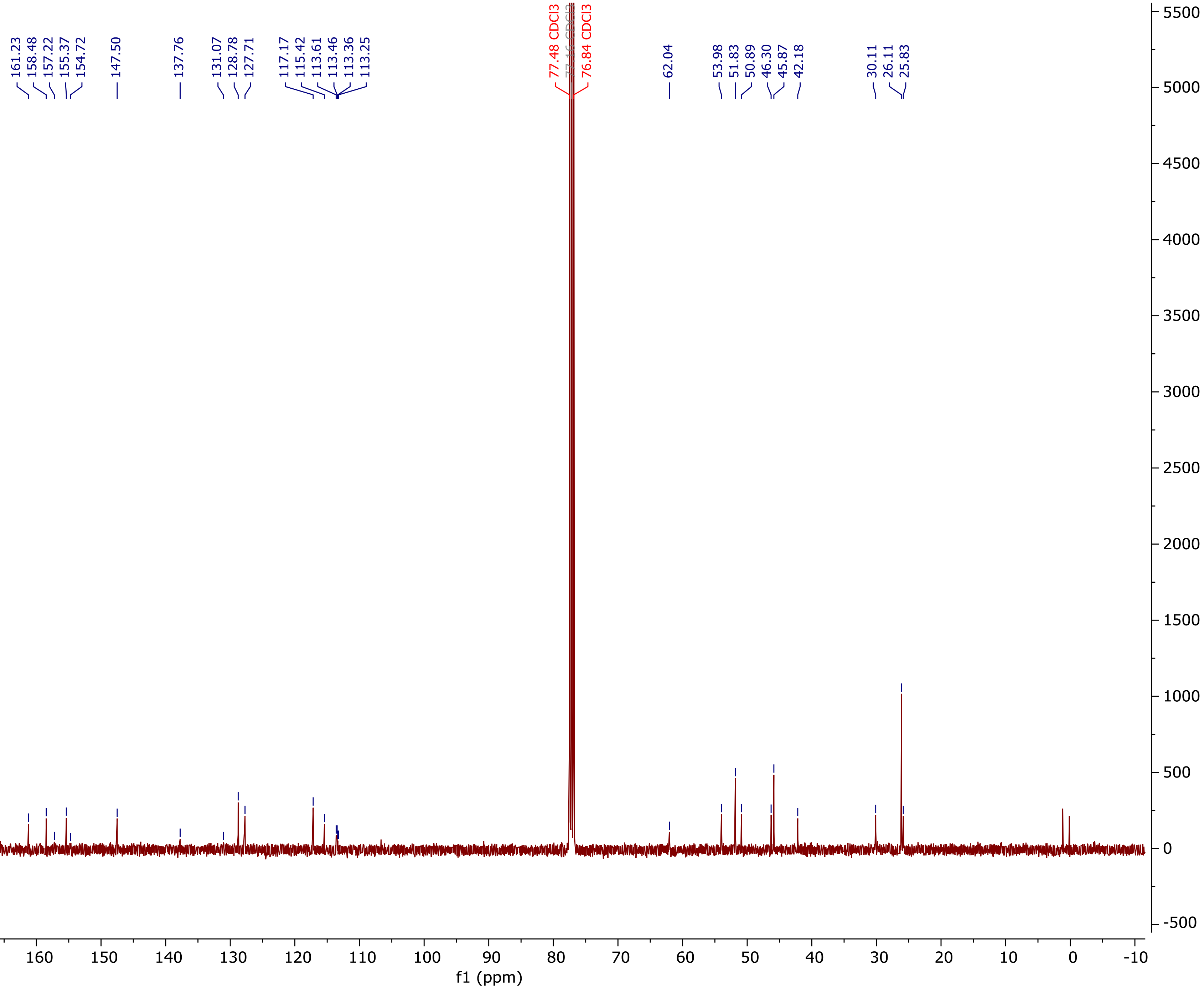

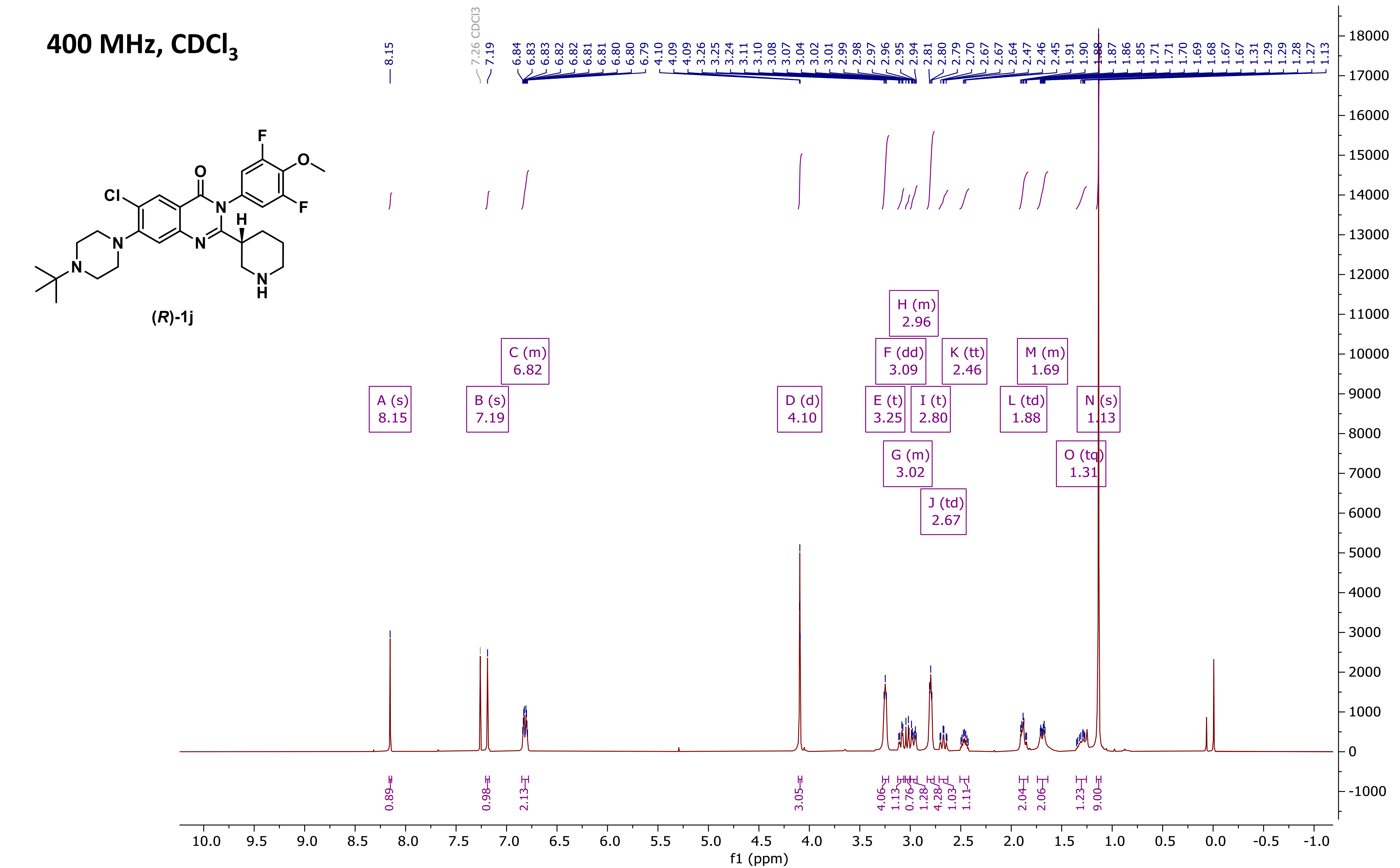
400 MHz, CDCl3
(R)-1j
A (s) 8.15
B (s) 7.19
C (m) 6.82
D (d) 4.10
E (t) 3.25
F (dd) 3.09
G (m) 3.02
H (m) 2.96
I (t) 2.80
J (td) 2.67
K (tt) 2.46
L (td) 1.88
M (m) 1.69
N (s) 1.13
O (tq) 1.31
f1 (ppm)

101 MHz, $CDCl_3$

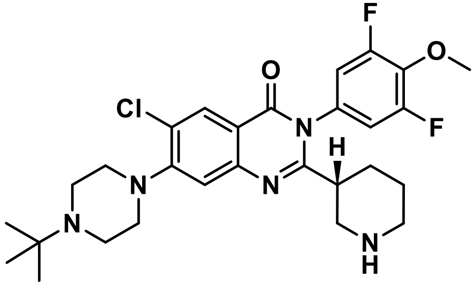

(*R*)-1j

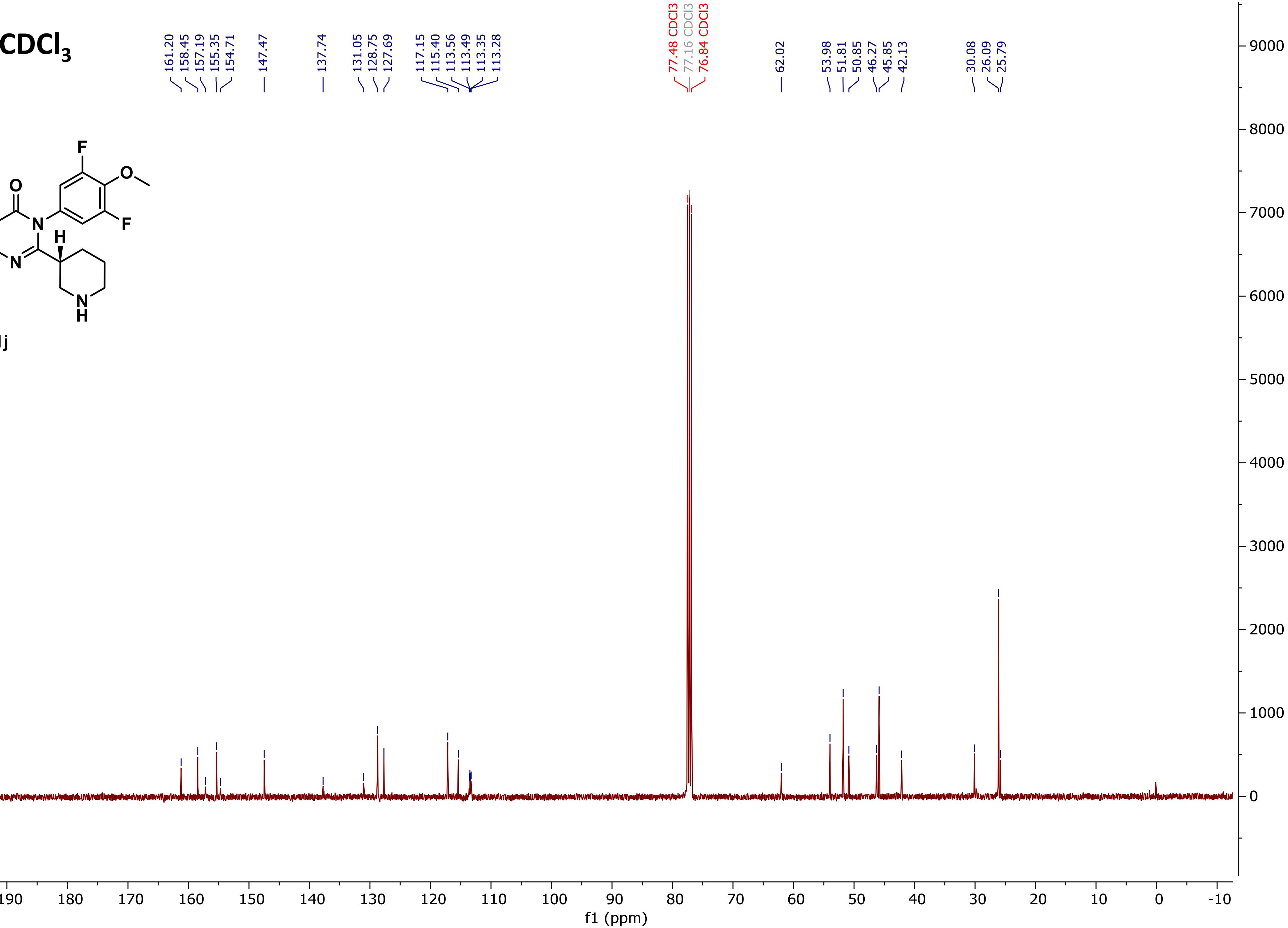

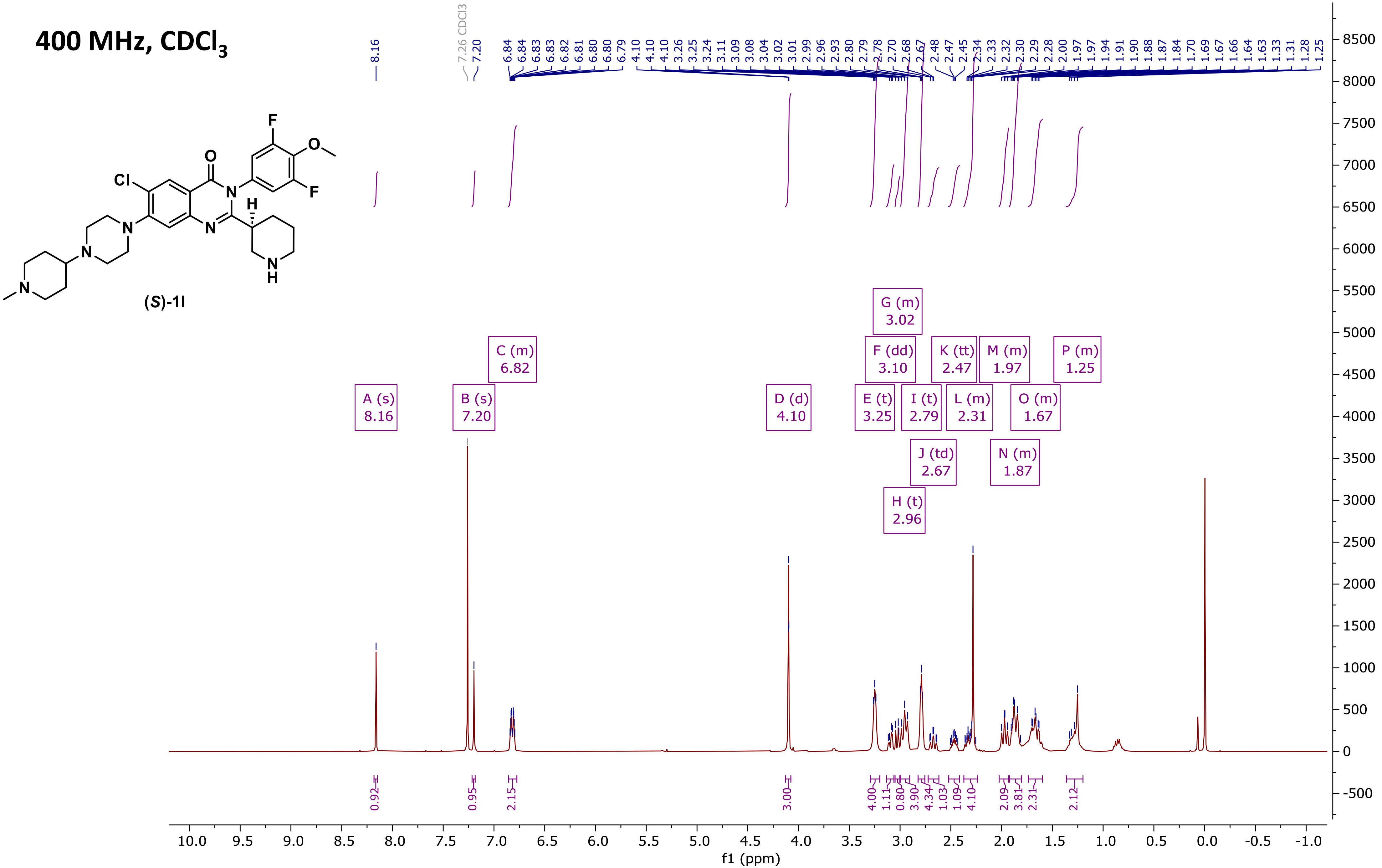

400 MHz, CDCl3
(S)-1l
A (s) 8.16
B (s) 7.20
C (m) 6.82
D (d) 4.10
E (t) 3.25
F (dd) 3.10
G (m) 3.02
H (t) 2.96
I (t) 2.79
J (td) 2.67
K (tt) 2.47
L (m) 2.31
M (m) 1.97
N (m) 1.87
O (m) 1.67
P (m) 1.25
f1 (ppm)

101 MHz, $CDCl_3$

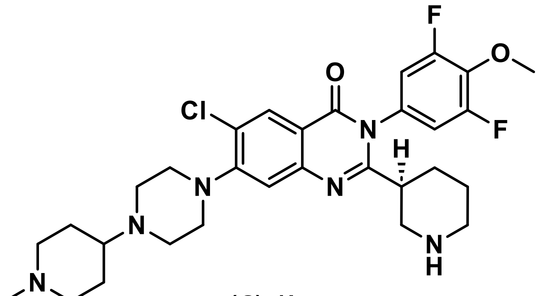

(*S*)-1l

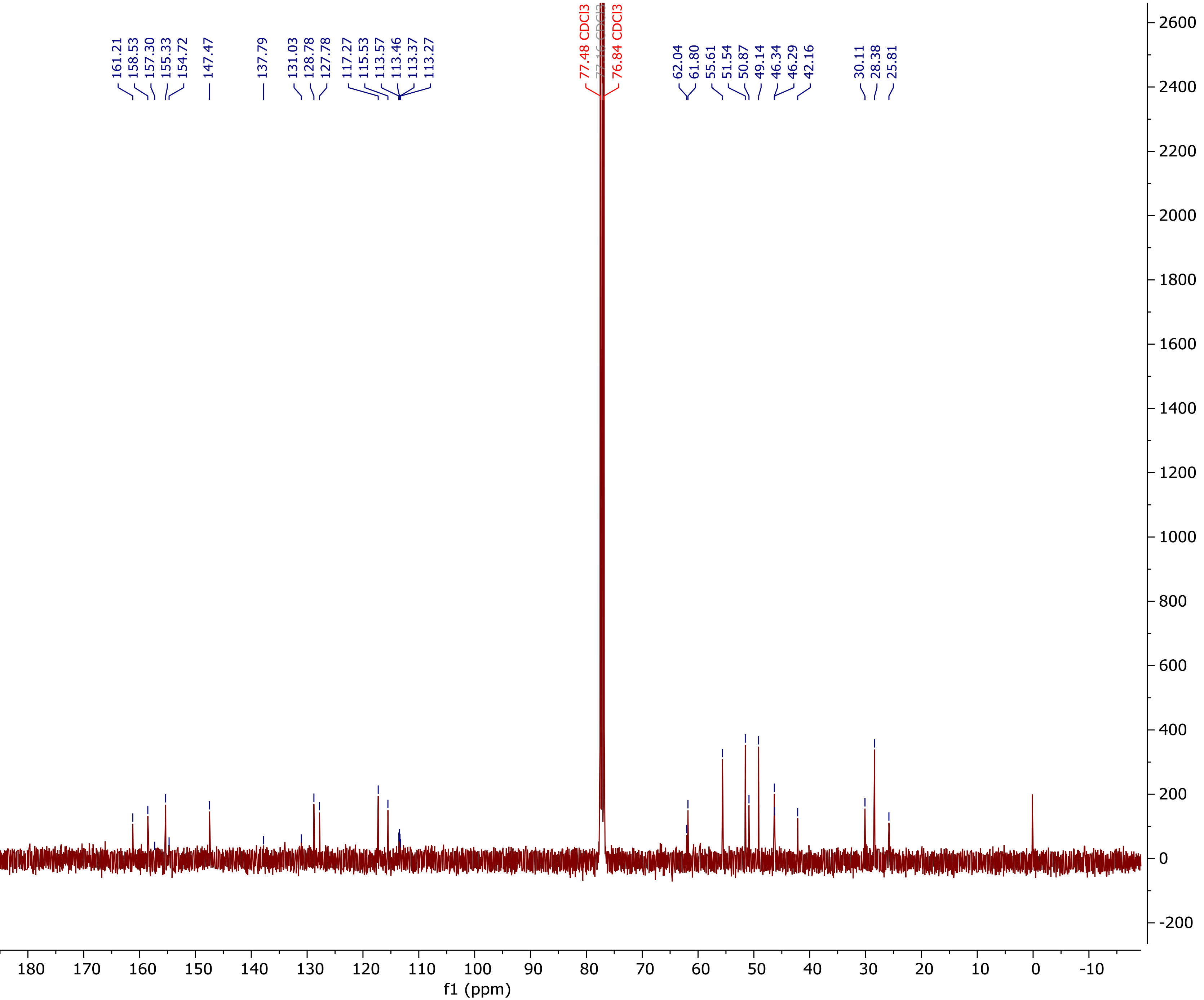

400 MHz, $CDCl_3$

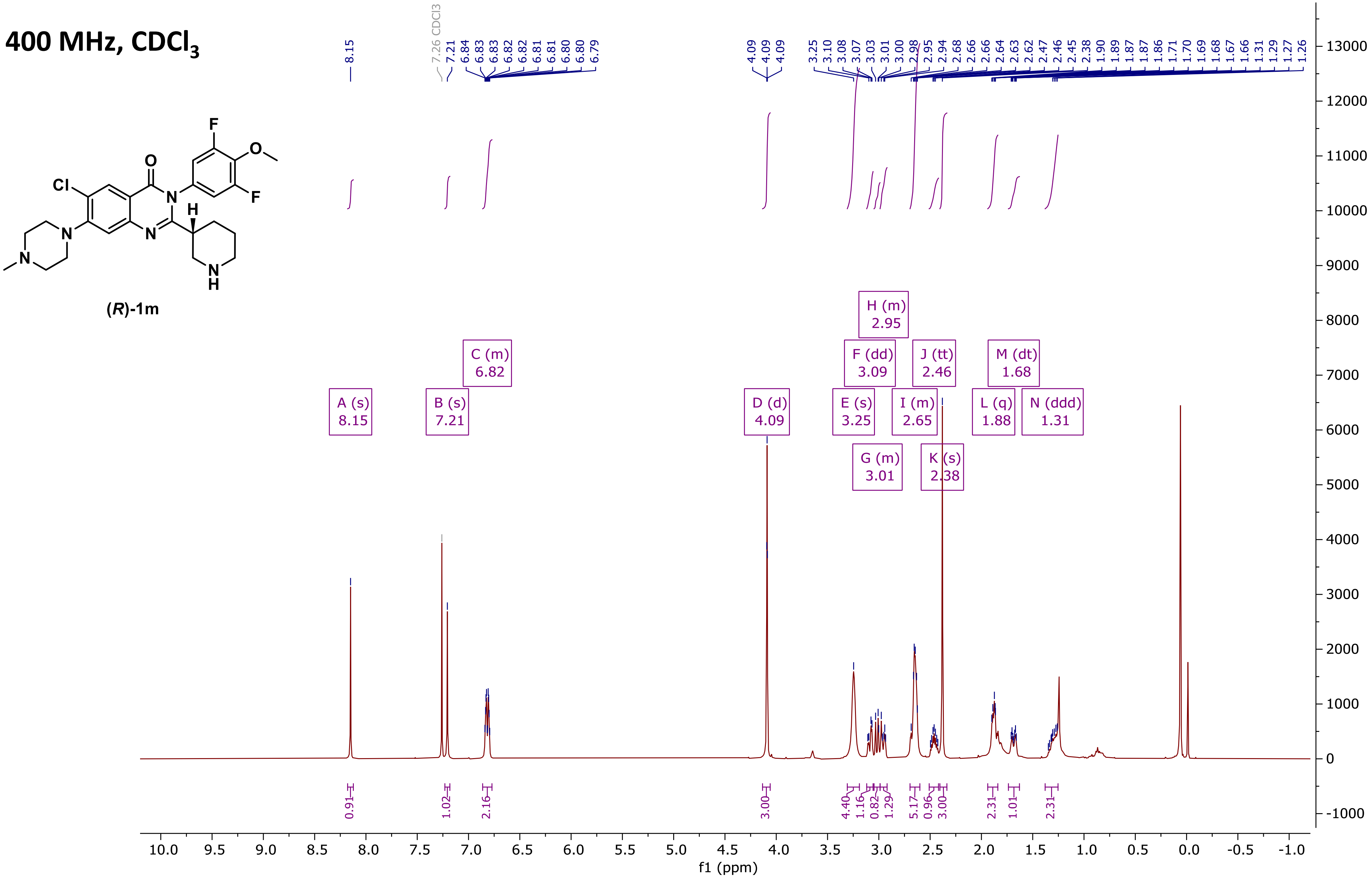

(*R*)-1m

101 MHz, $CDCl_3$

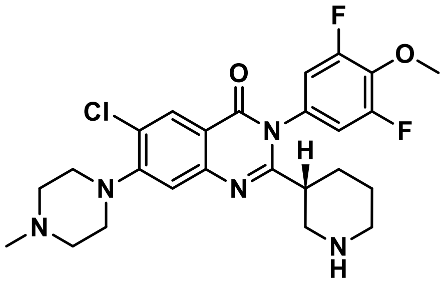

(*R*)-1m

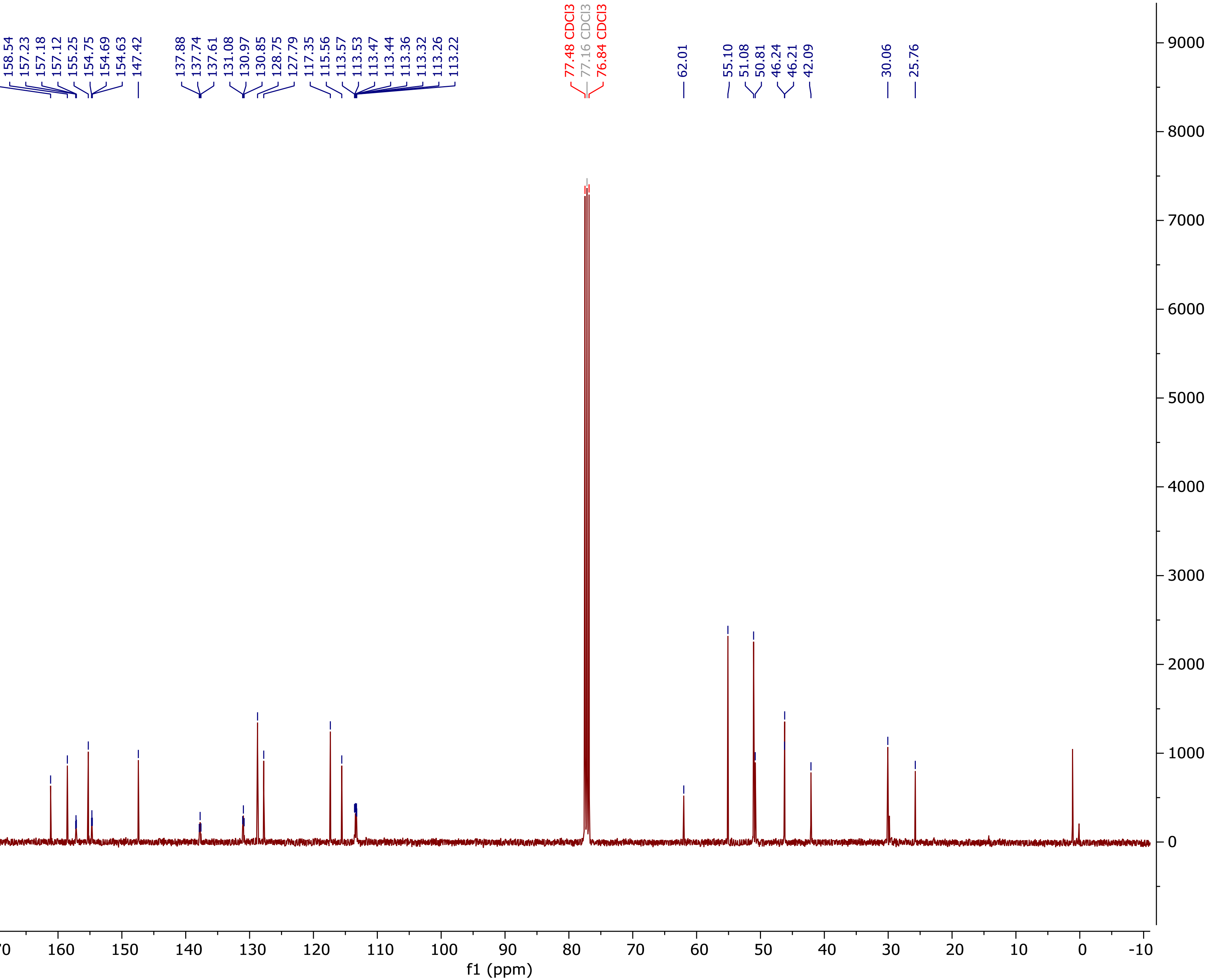

400 MHz, $CDCl_3$

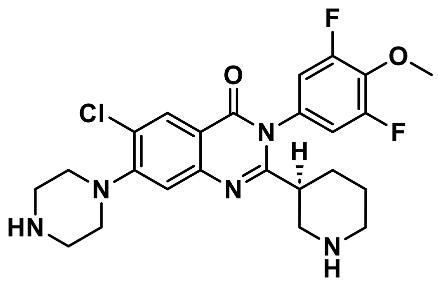

(*S*)-1n

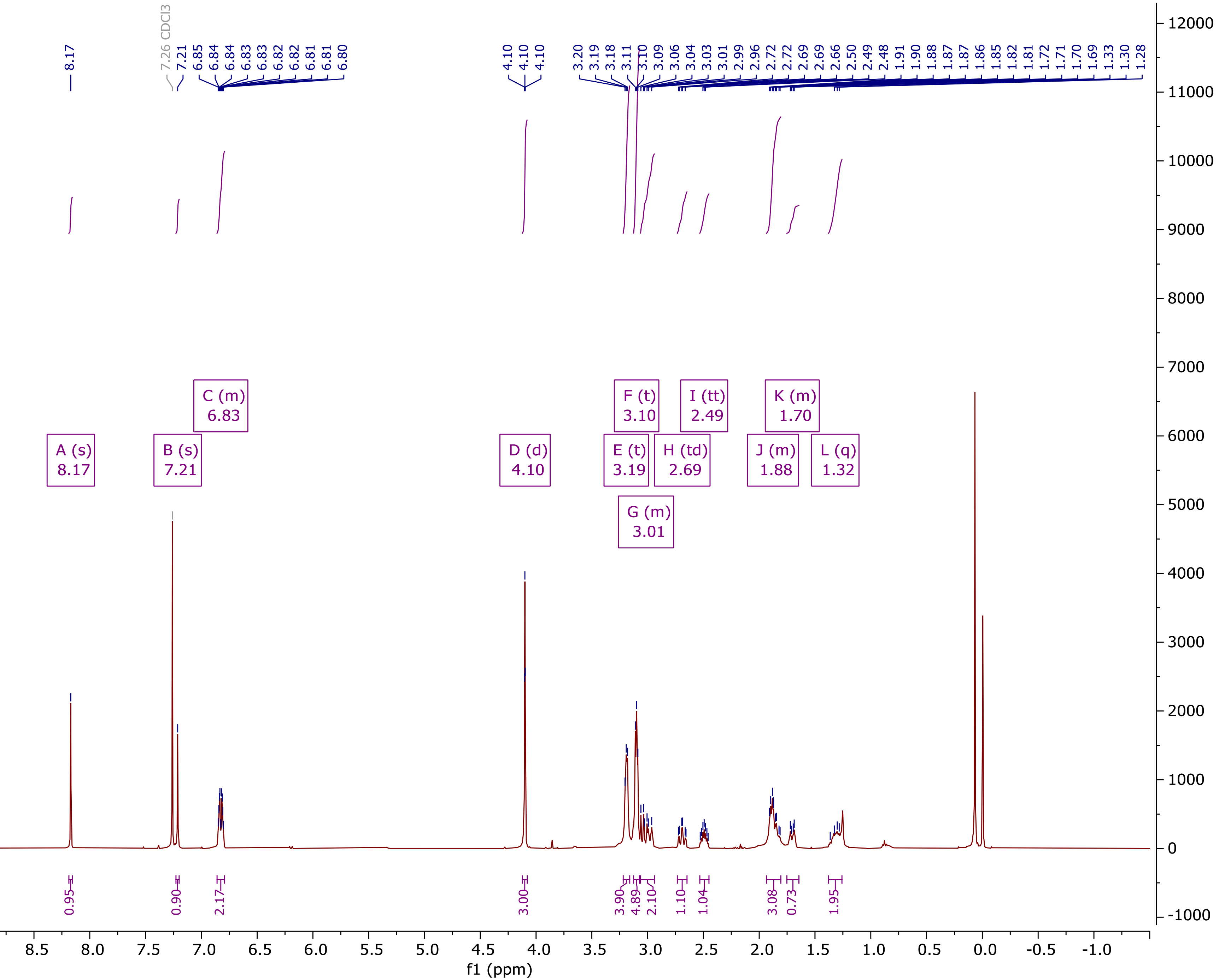

101 MHz, $CDCl_3$

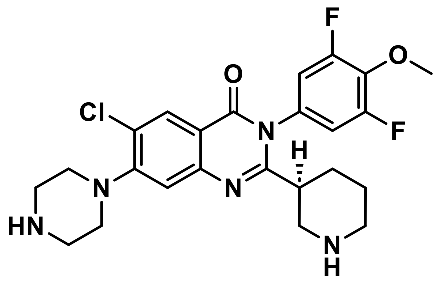

(*S*)-1n

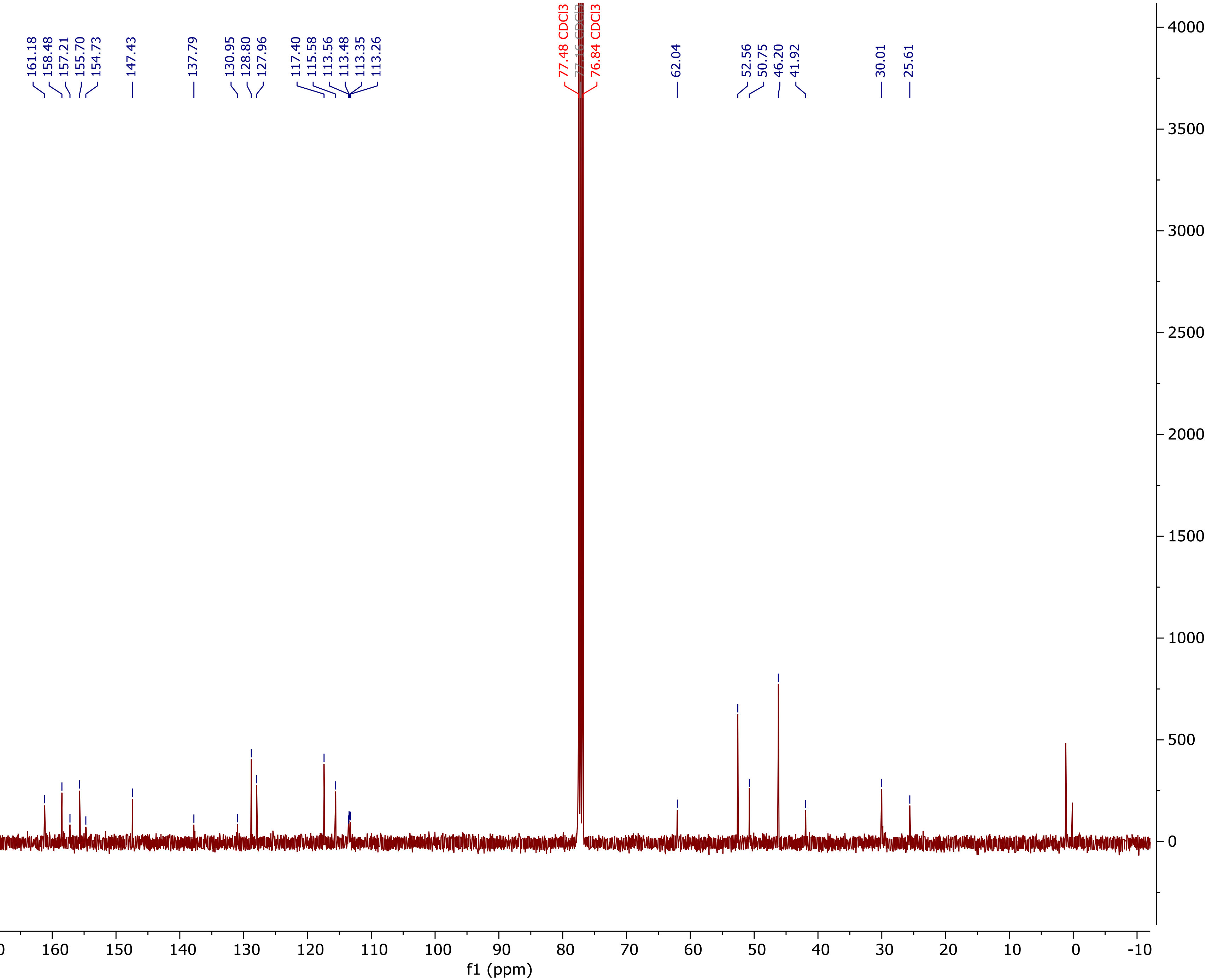

400 MHz, $CDCl_3$

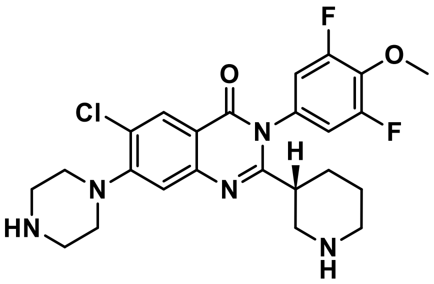

(*R*)-1n

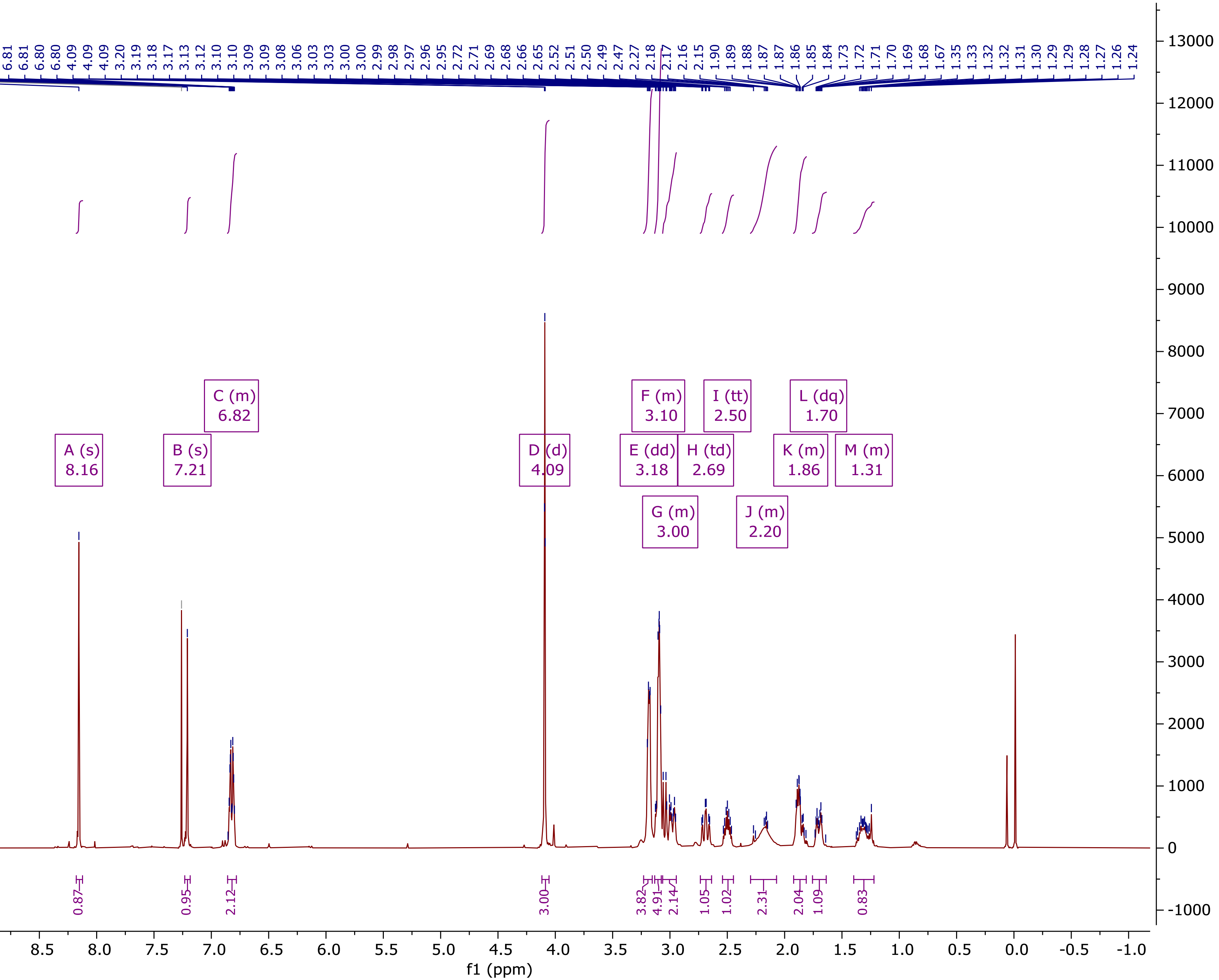

101 MHz, $CDCl_3$

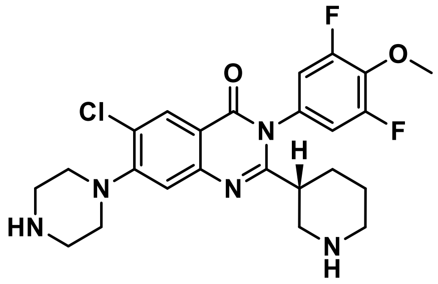

(*R*)-1n

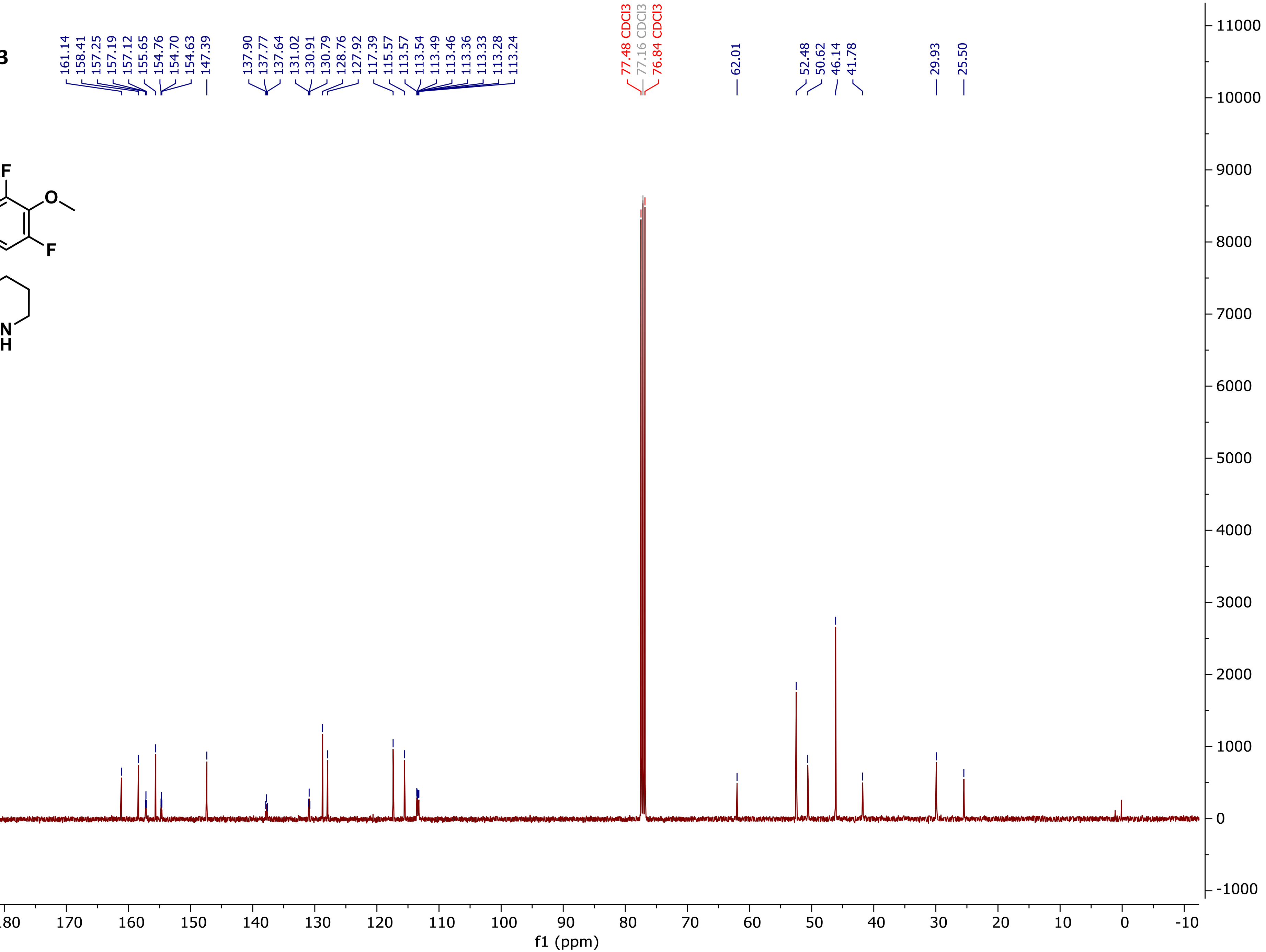

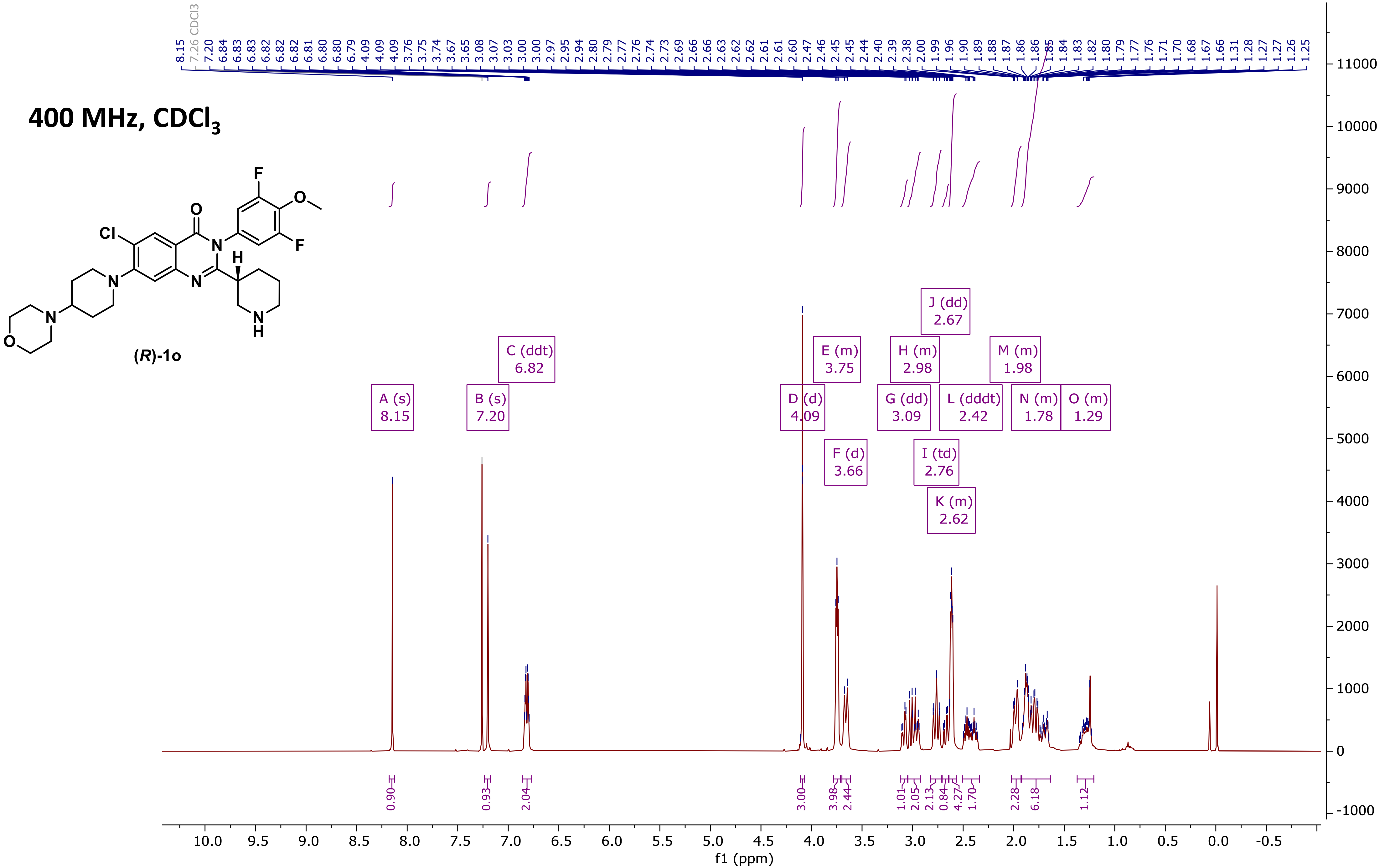

400 MHz, CDCl3
(R)-1o
A (s) 8.15
B (s) 7.20
C (ddt) 6.82
D (d) 4.09
E (m) 3.75
F (d) 3.66
G (dd) 3.09
H (m) 2.98
I (td) 2.76
J (dd) 2.67
K (m) 2.62
L (dddt) 2.42
M (m) 1.98
N (m) 1.78
O (m) 1.29
f1 (ppm)

101 MHz, CDCl$_3$

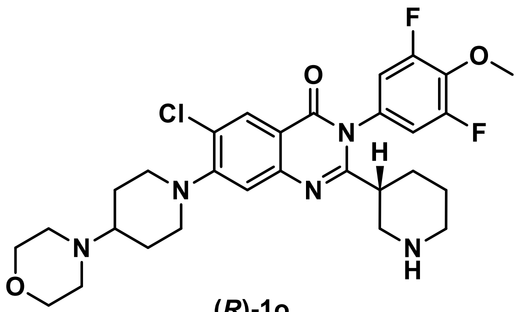

(*R*)-1o

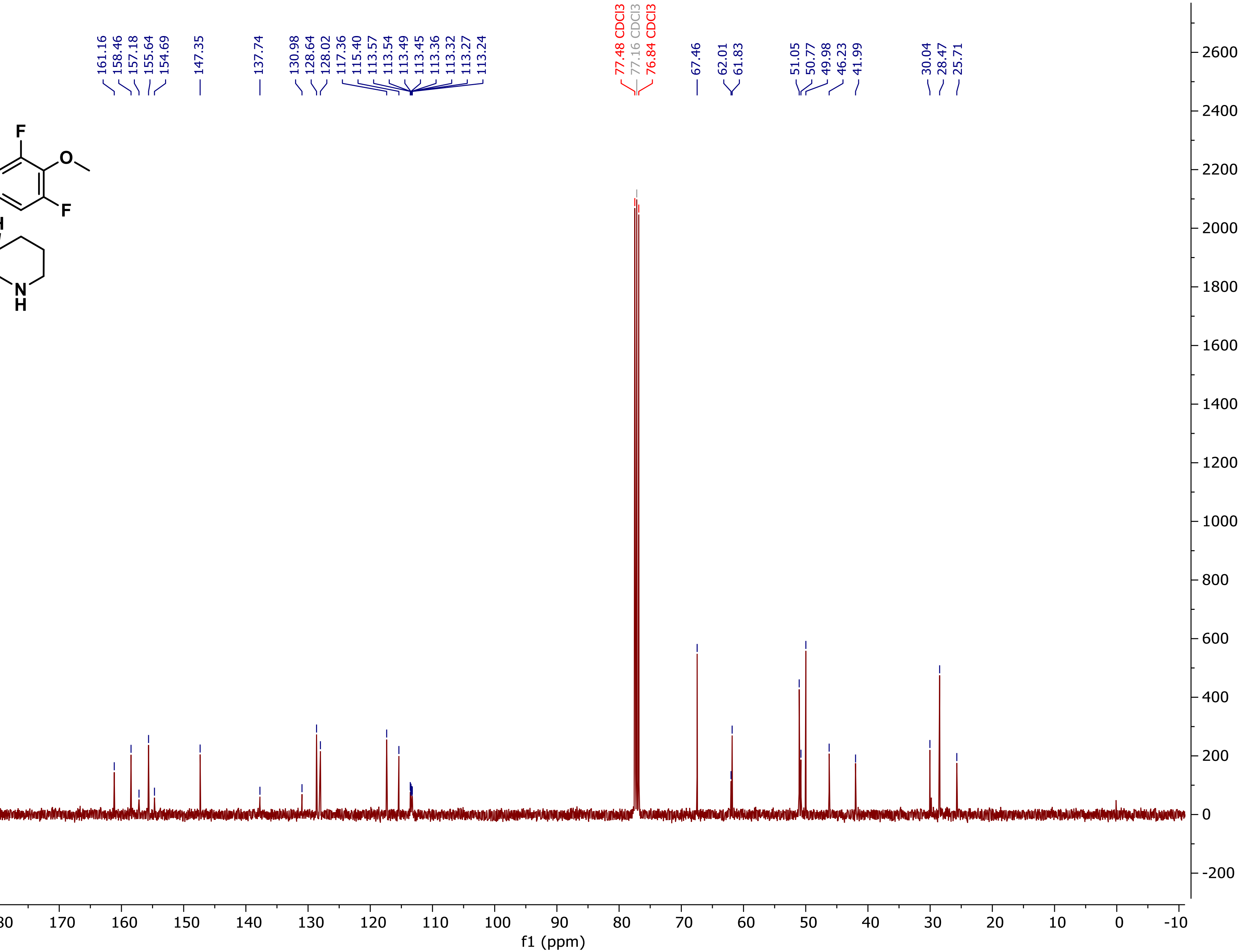

400 MHz, $CDCl_3$

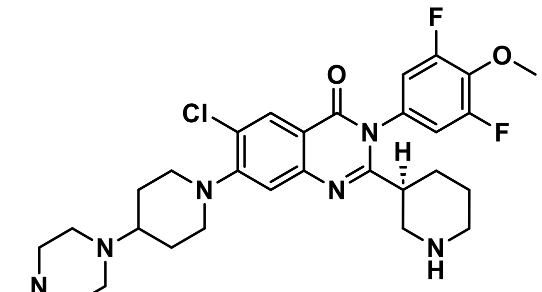

(*S*)-1p

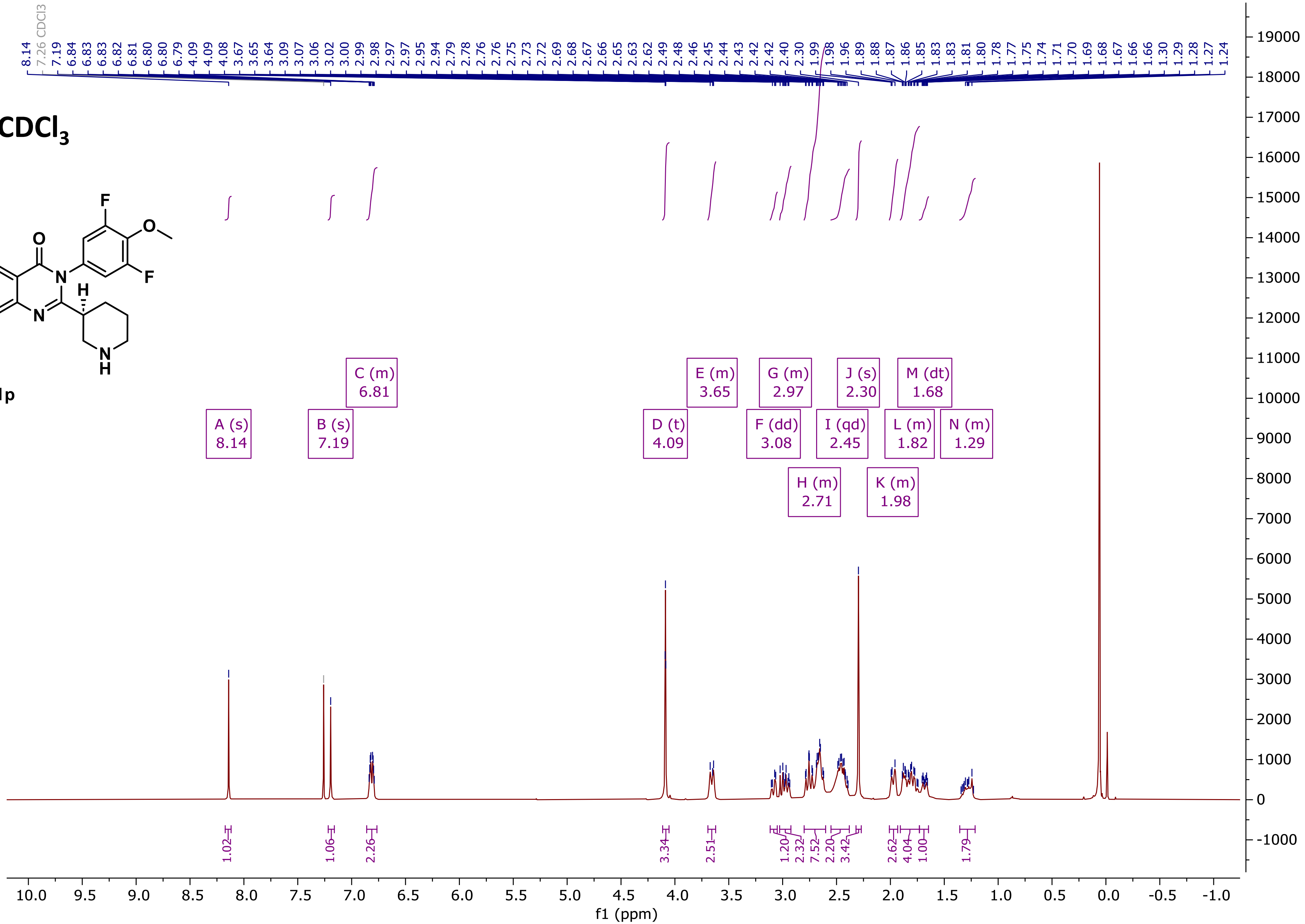

101 MHz, CDCl$_3$

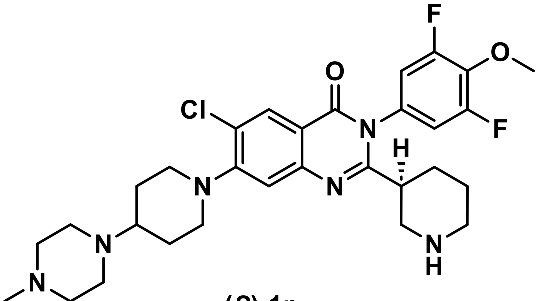

(*S*)-1p

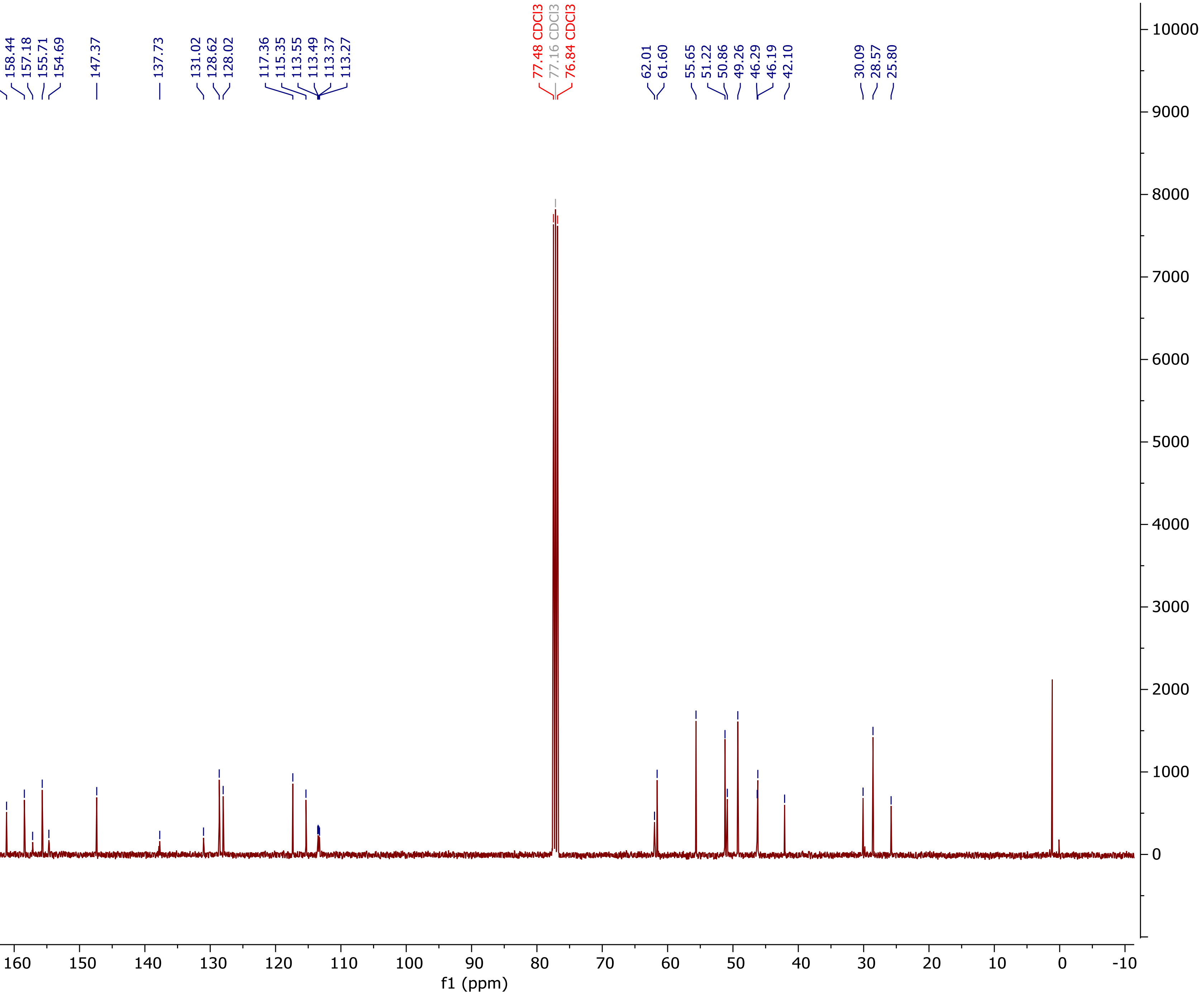

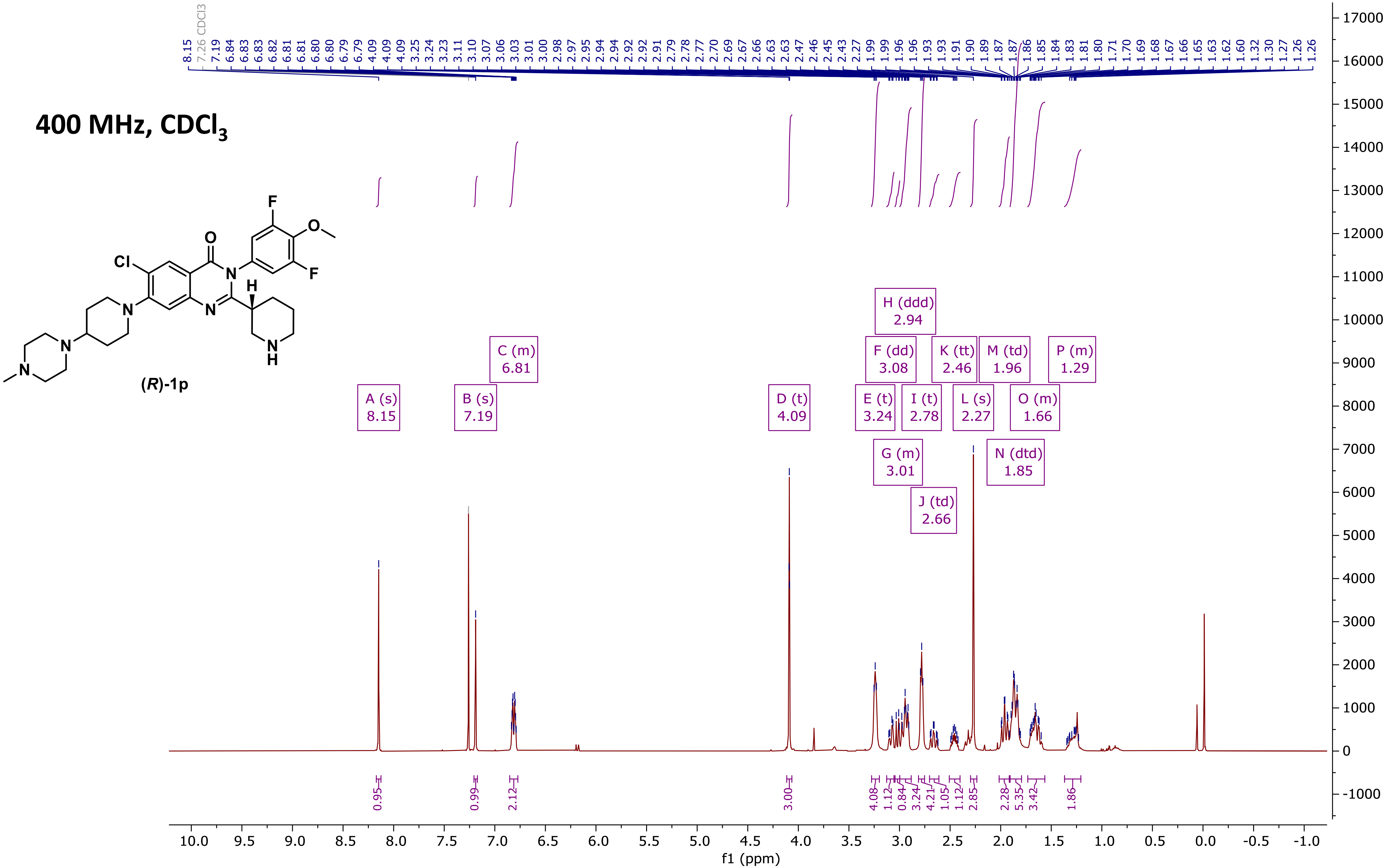
400 MHz, CDCl3
(R)-1p
A (s) 8.15
B (s) 7.19
C (m) 6.81
D (t) 4.09
E (t) 3.24
F (dd) 3.08
G (m) 3.01
H (ddd) 2.94
I (t) 2.78
J (td) 2.66
K (tt) 2.46
L (s) 2.27
M (td) 1.96
N (dtd) 1.85
O (m) 1.66
P (m) 1.29
f1 (ppm)

101 MHz, $CDCl_3$

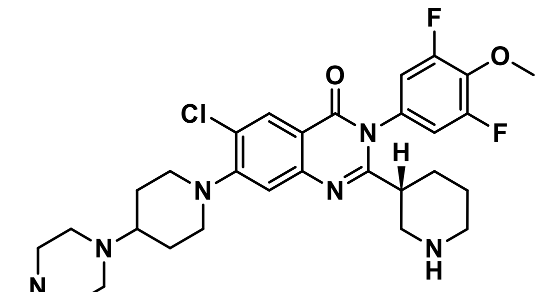

(*R*)-1p

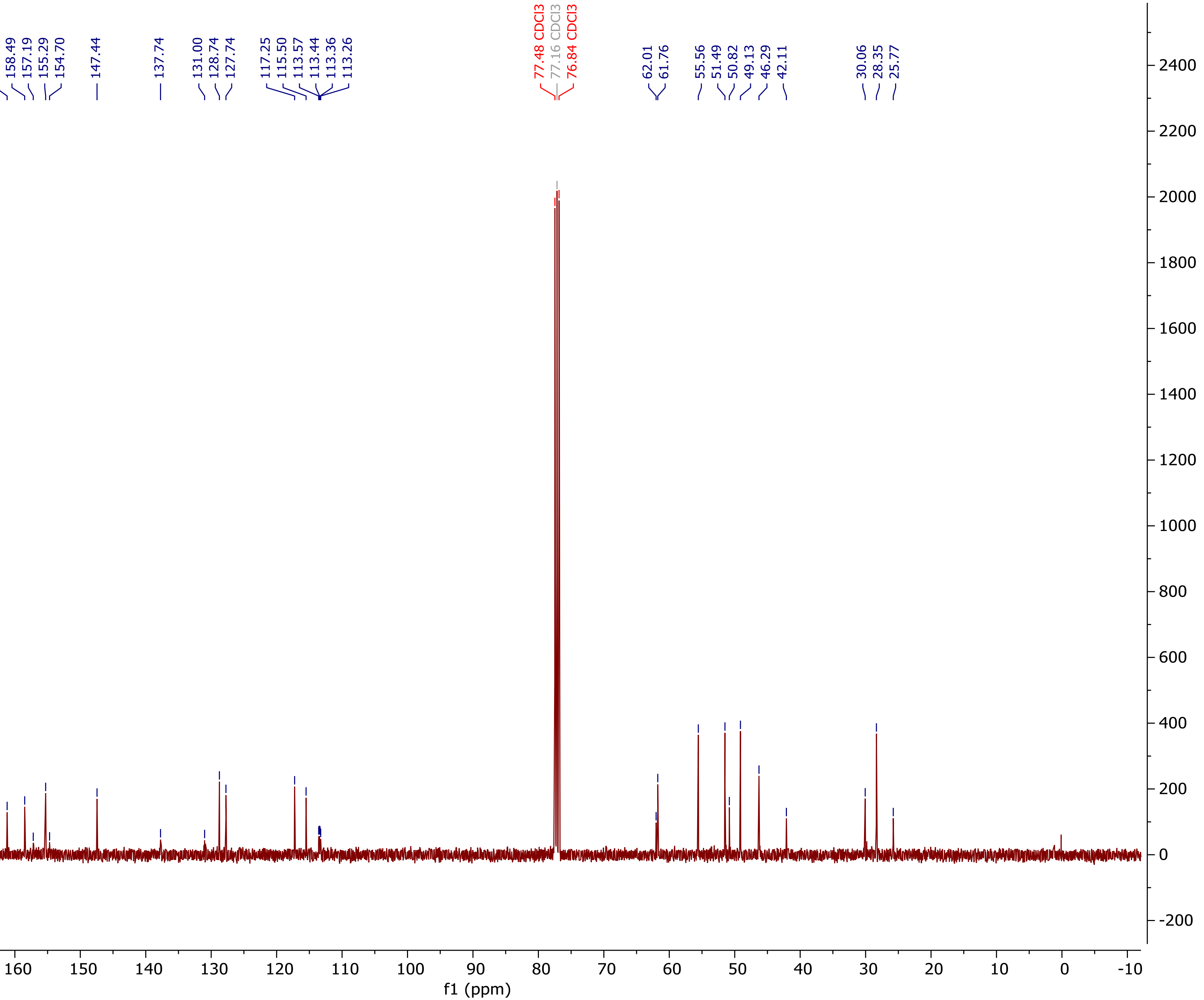

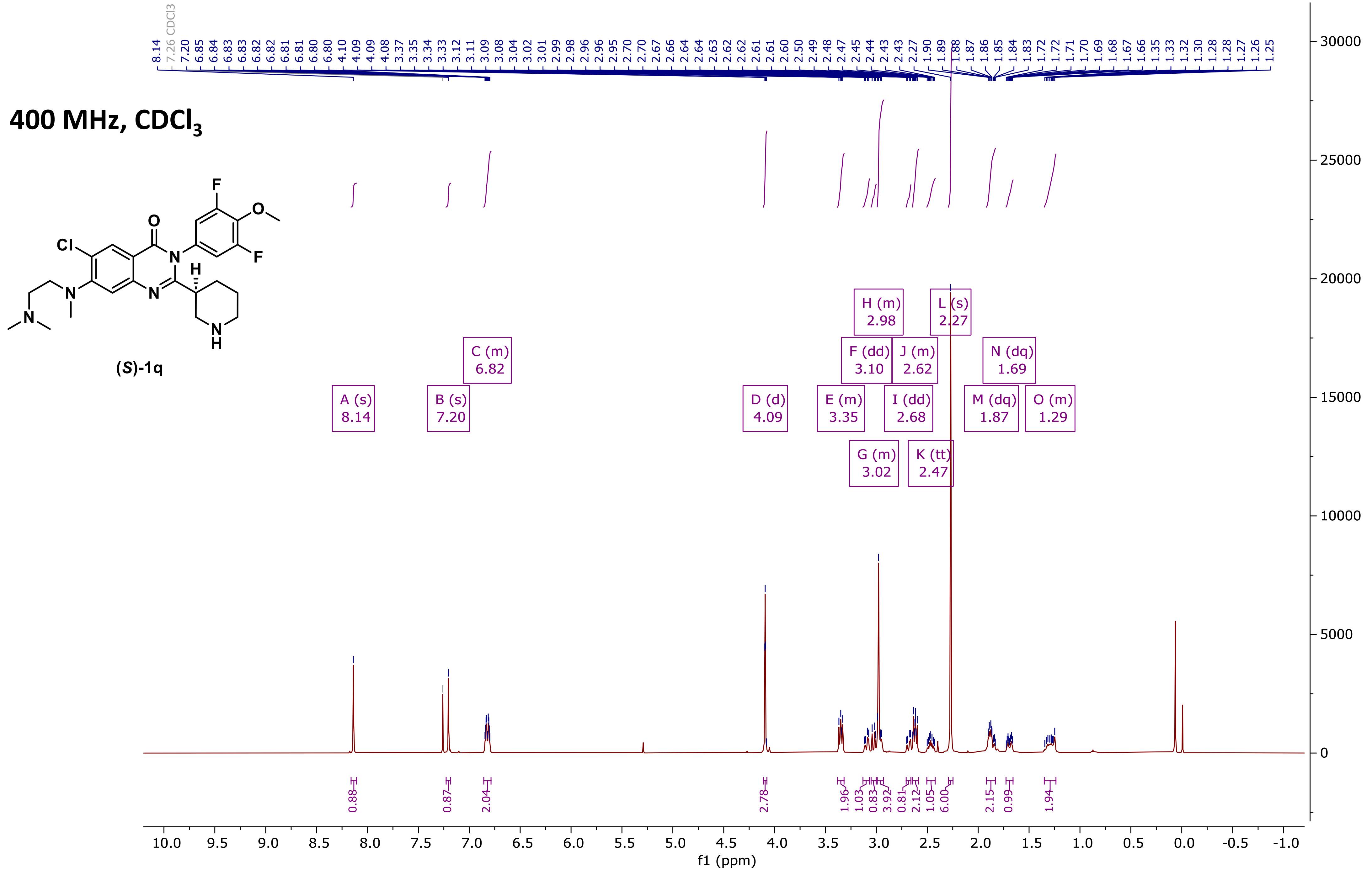
400 MHz, CDCl3
(S)-1q
A (s) 8.14
B (s) 7.20
C (m) 6.82
D (d) 4.09
E (m) 3.35
F (dd) 3.10
G (m) 3.02
H (m) 2.98
I (dd) 2.68
J (m) 2.62
K (tt) 2.47
L (s) 2.27
M (dq) 1.87
N (dq) 1.69
O (m) 1.29
f1 (ppm)

101 MHz, $CDCl_3$

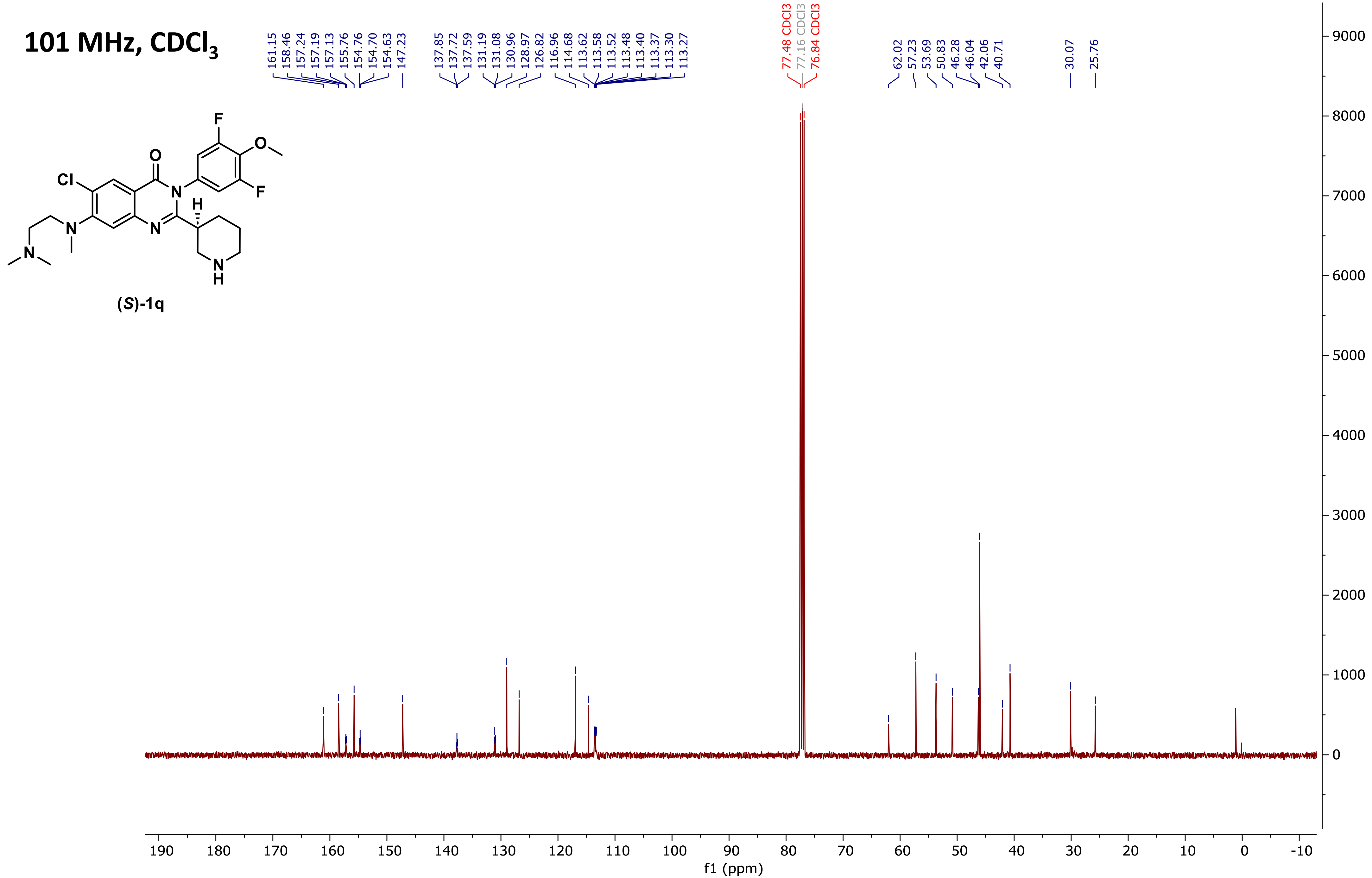

(*S*)-1q

400 MHz, $CDCl_3$

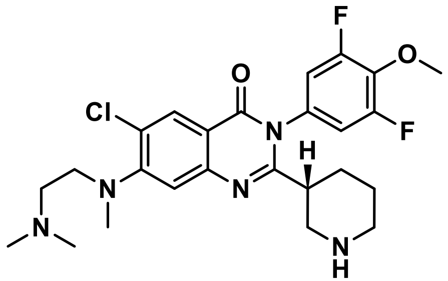

(*R*)-1q

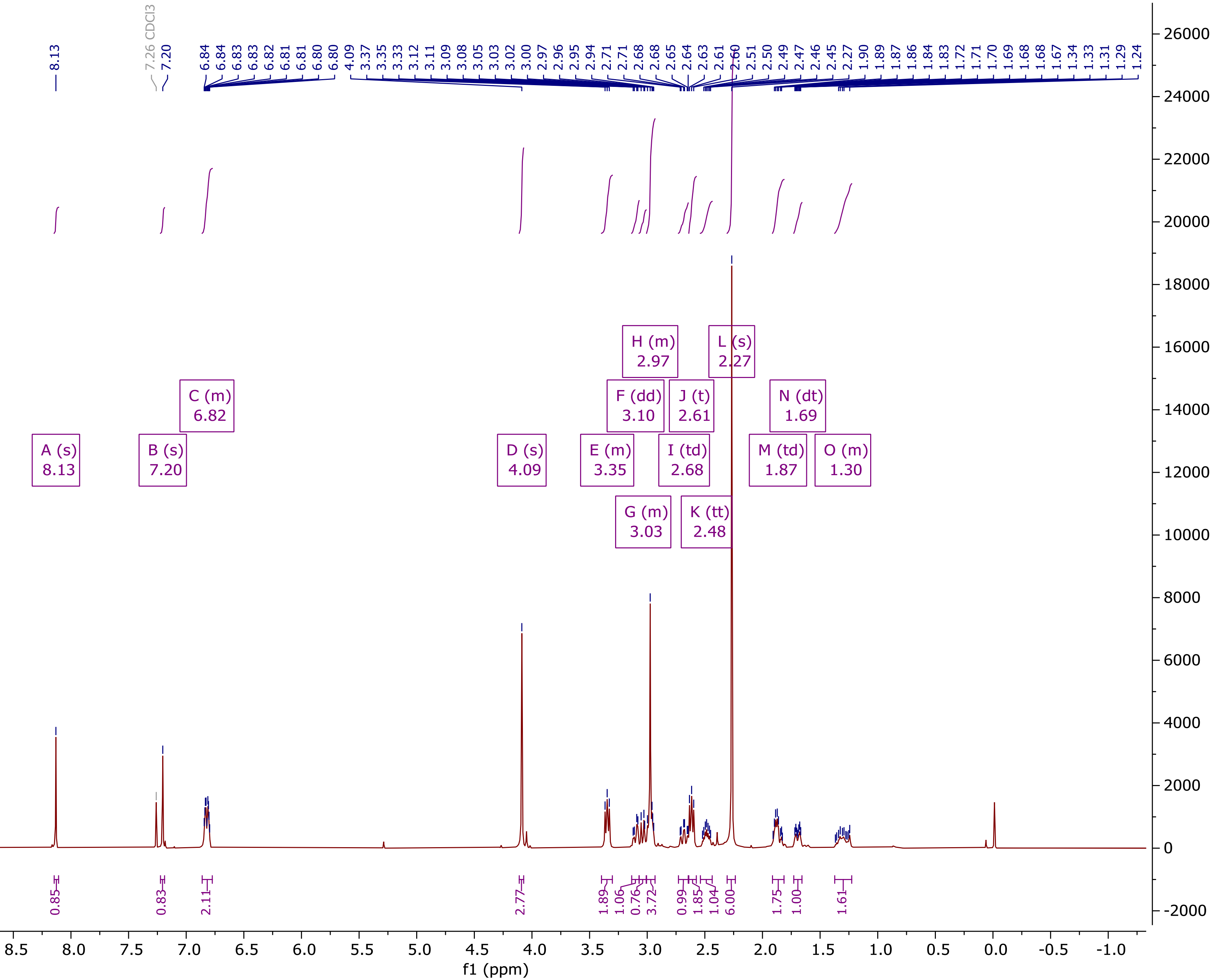

101 MHz, $CDCl_3$

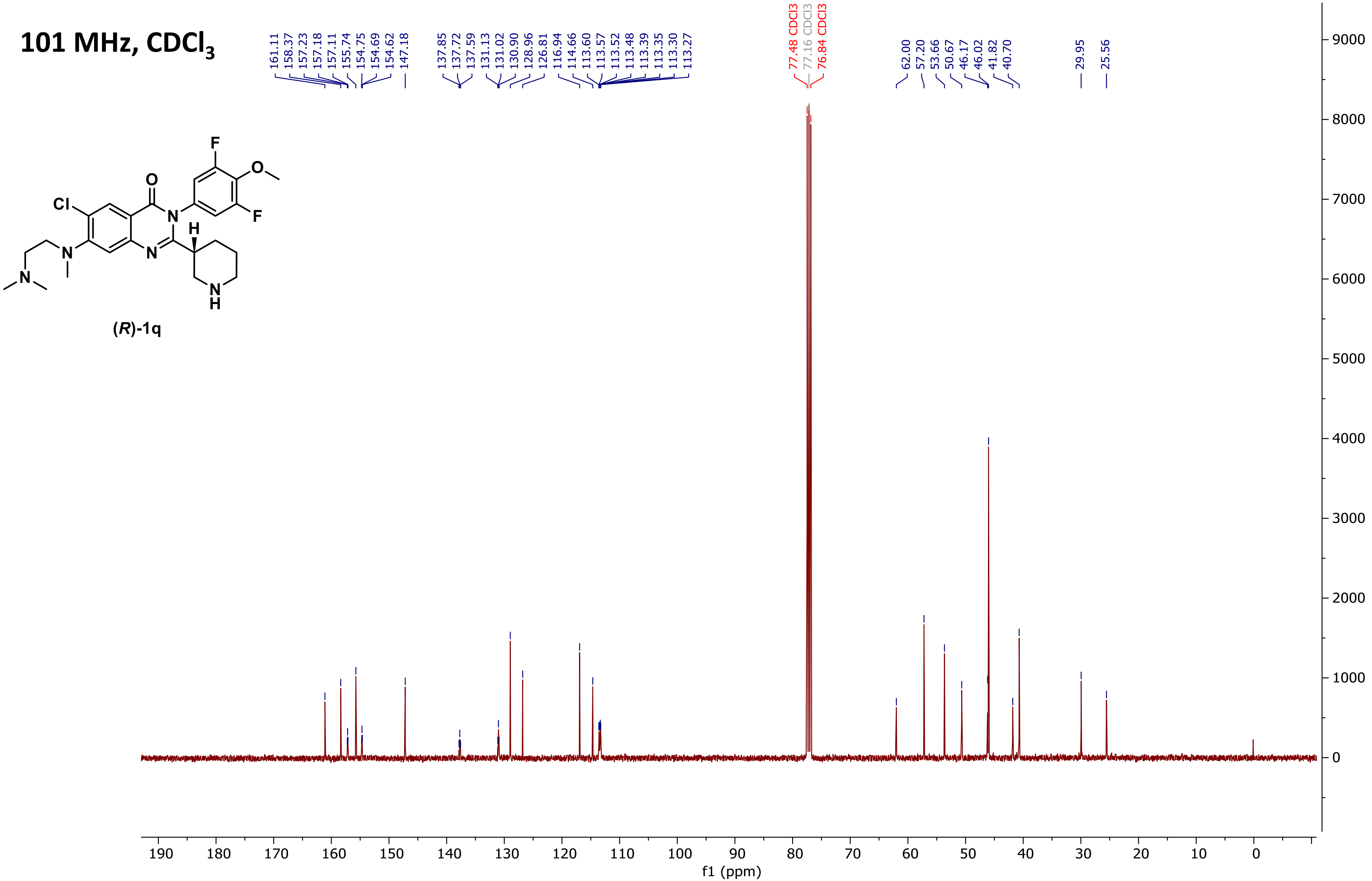

(*R*)-1q

400 MHz, CDCl$_3$

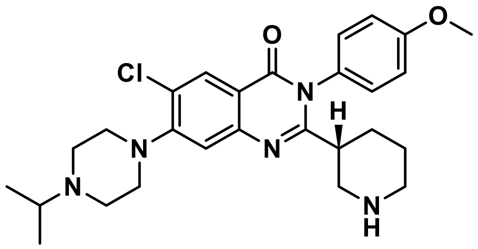

(*R*)-1t

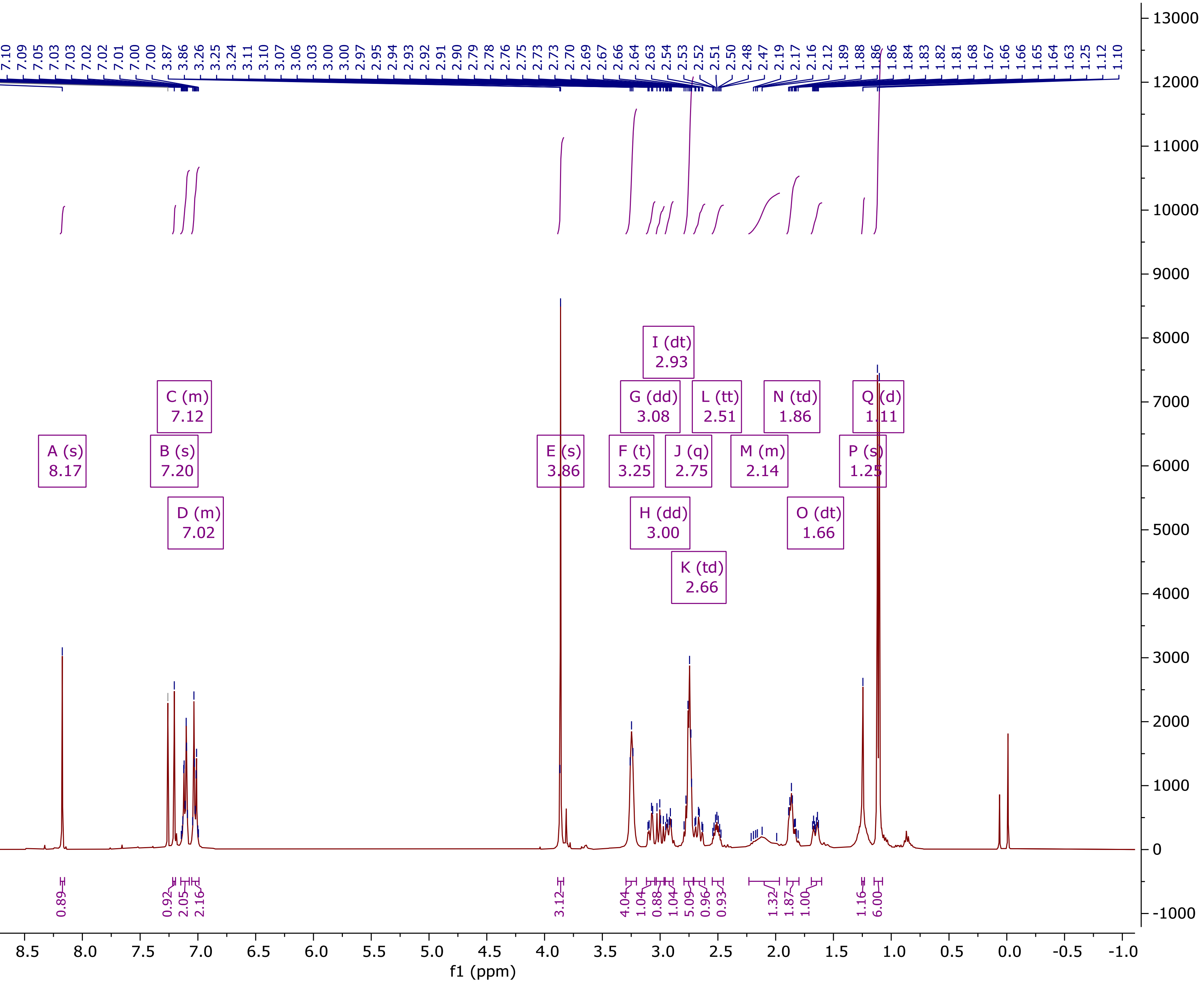

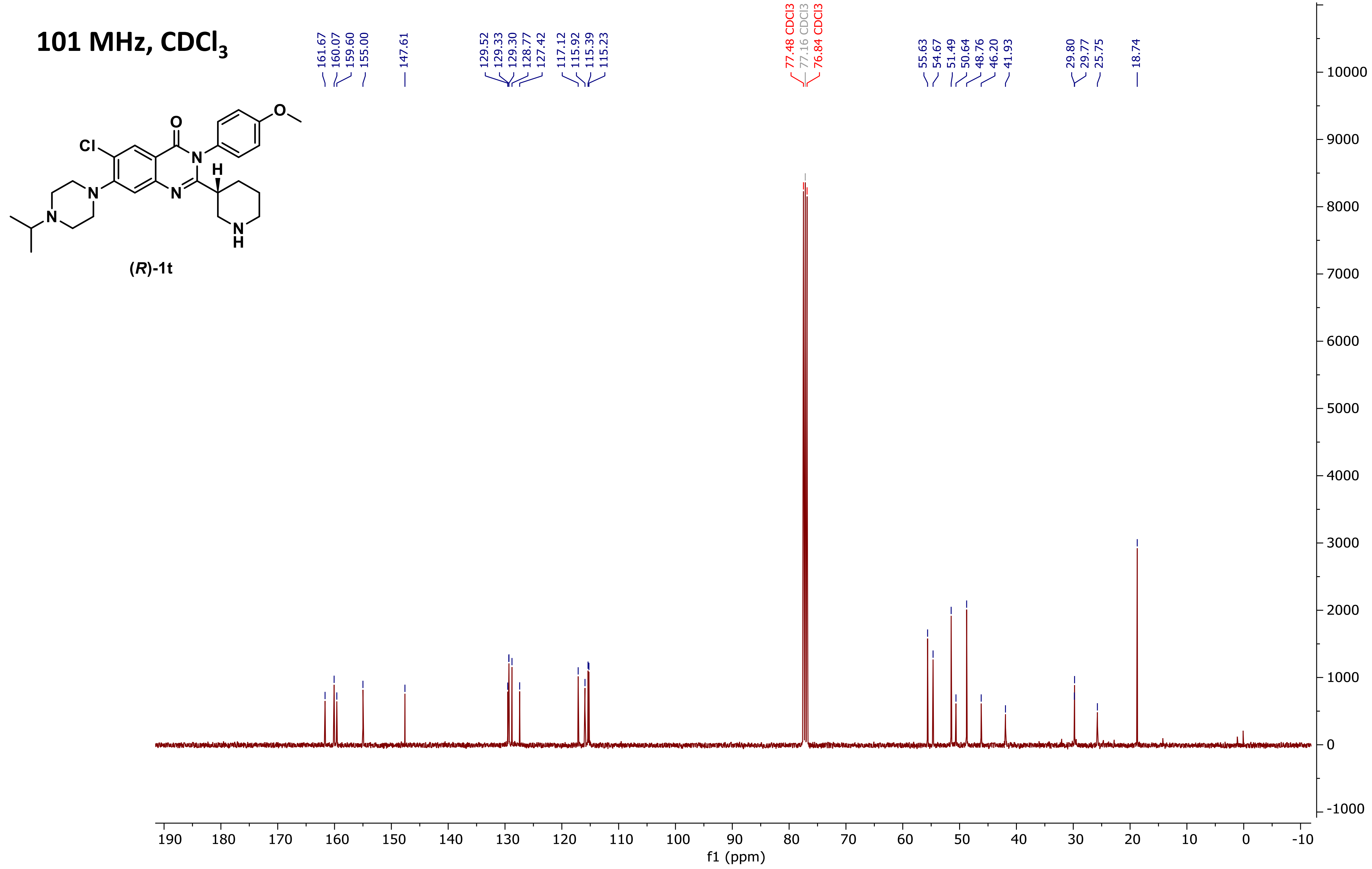
101 MHz, CDCl3
Cl
O
N
O
N
N
H
N
N
H
(R)-1t
161.67
160.07
159.60
155.00
147.61
129.52
129.33
129.30
128.77
127.42
117.12
115.92
115.39
115.23
77.48 CDCl3
77.16 CDCl3
76.84 CDCl3
55.63
54.67
51.49
50.64
48.76
46.20
41.93
29.80
29.77
25.75
18.74
f1 (ppm)

400 MHz, $CDCl_3$

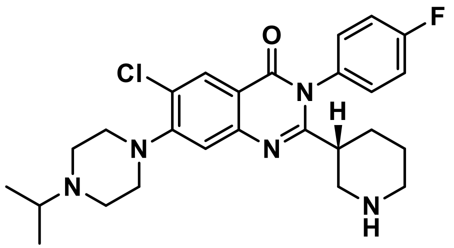

(*R*)-1u

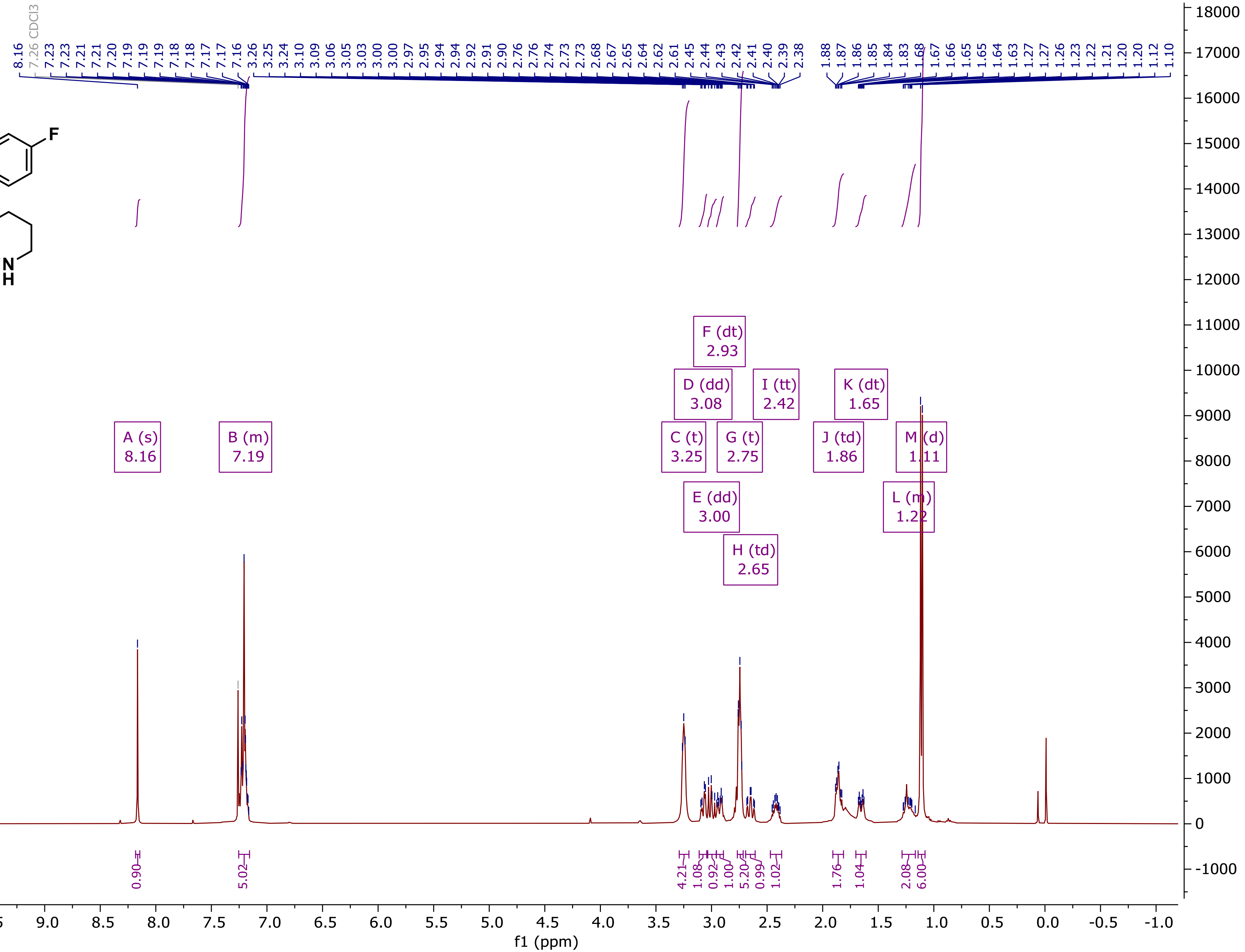

101 MHz, $CDCl_3$

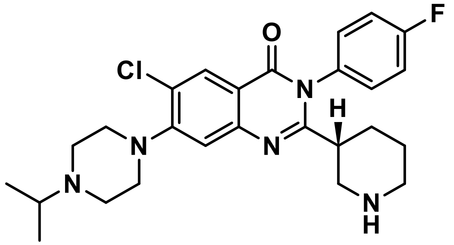

(*R*)-1u

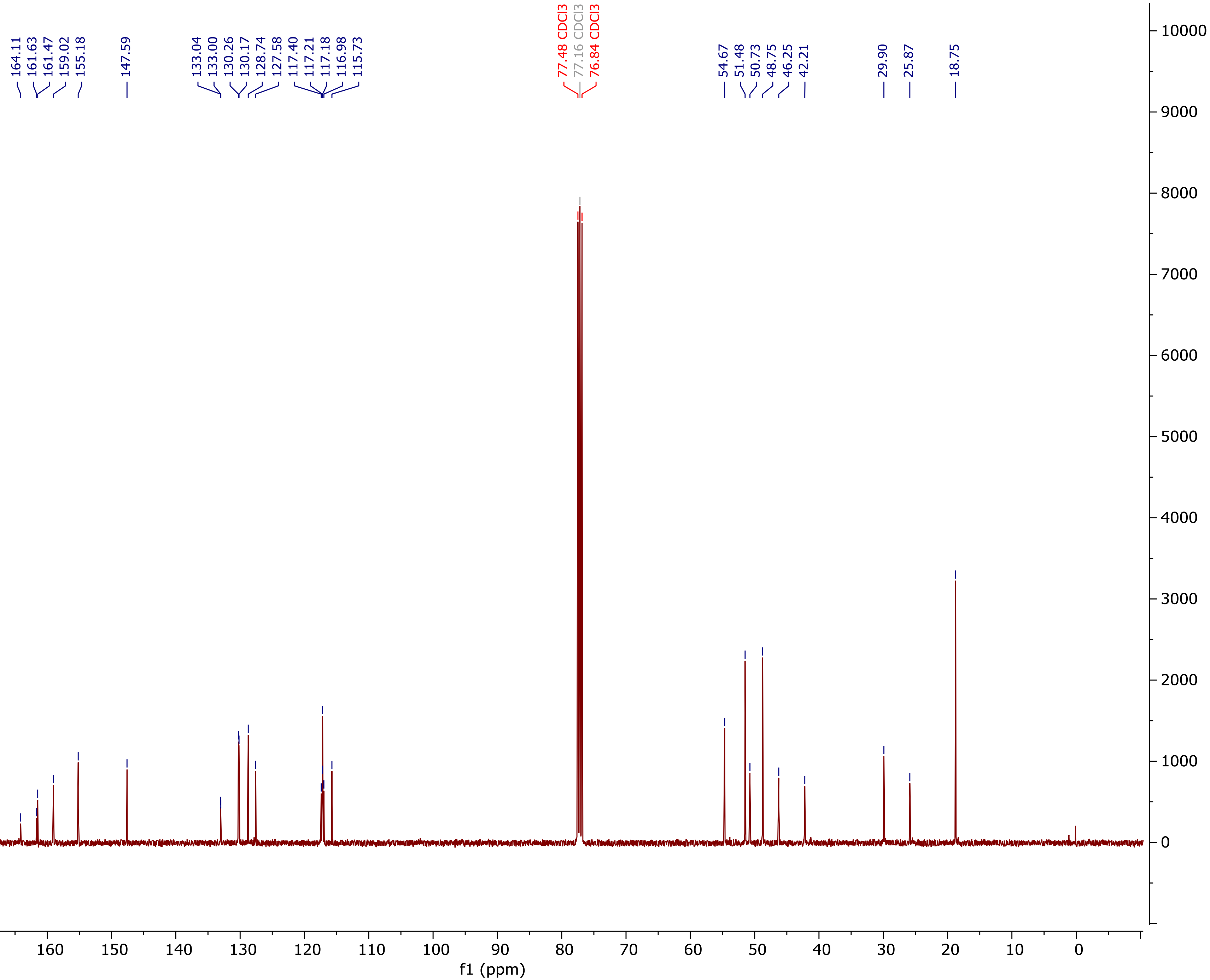

400 MHz, $CDCl_3$

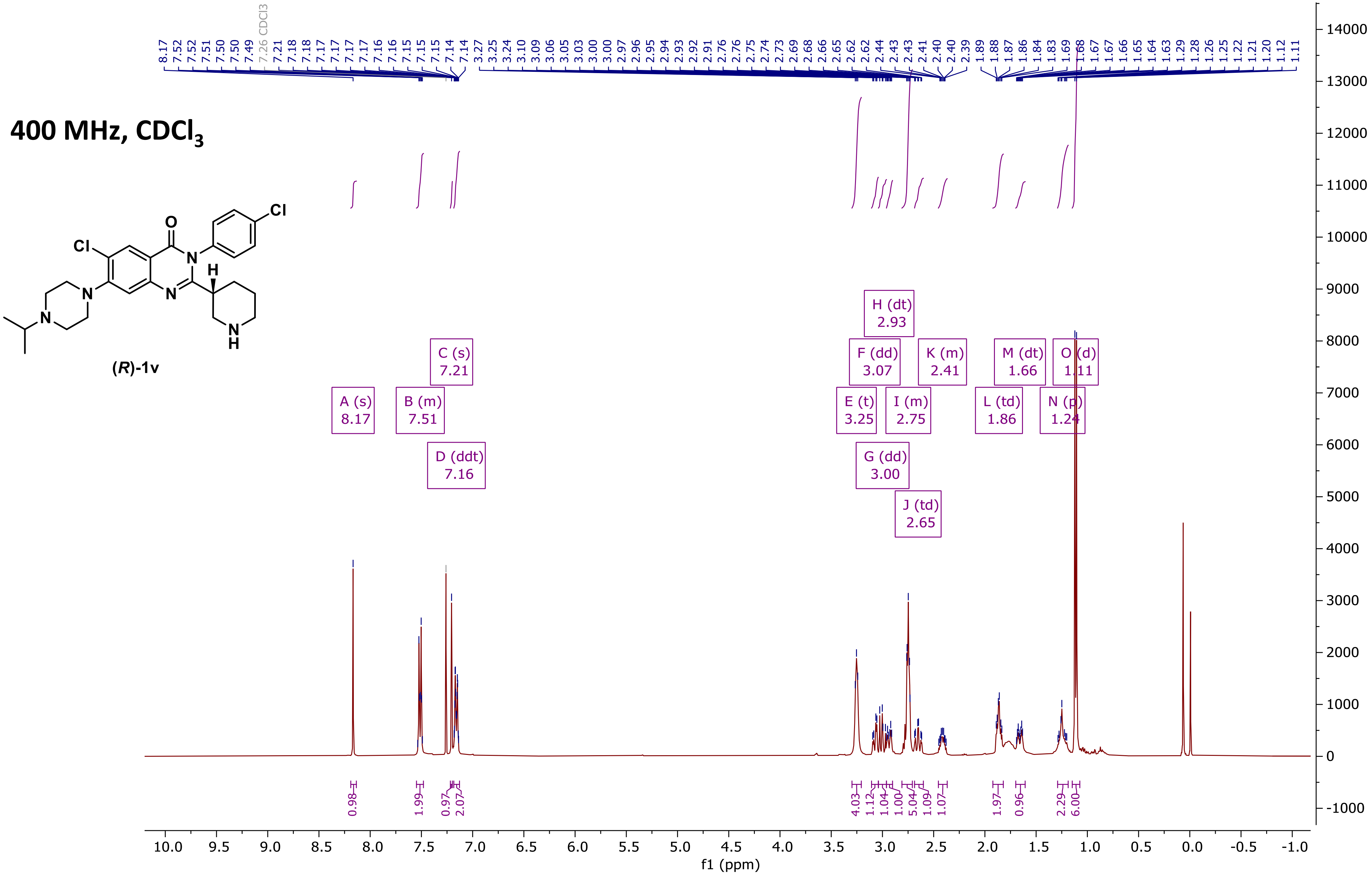

101 MHz, $CDCl_3$

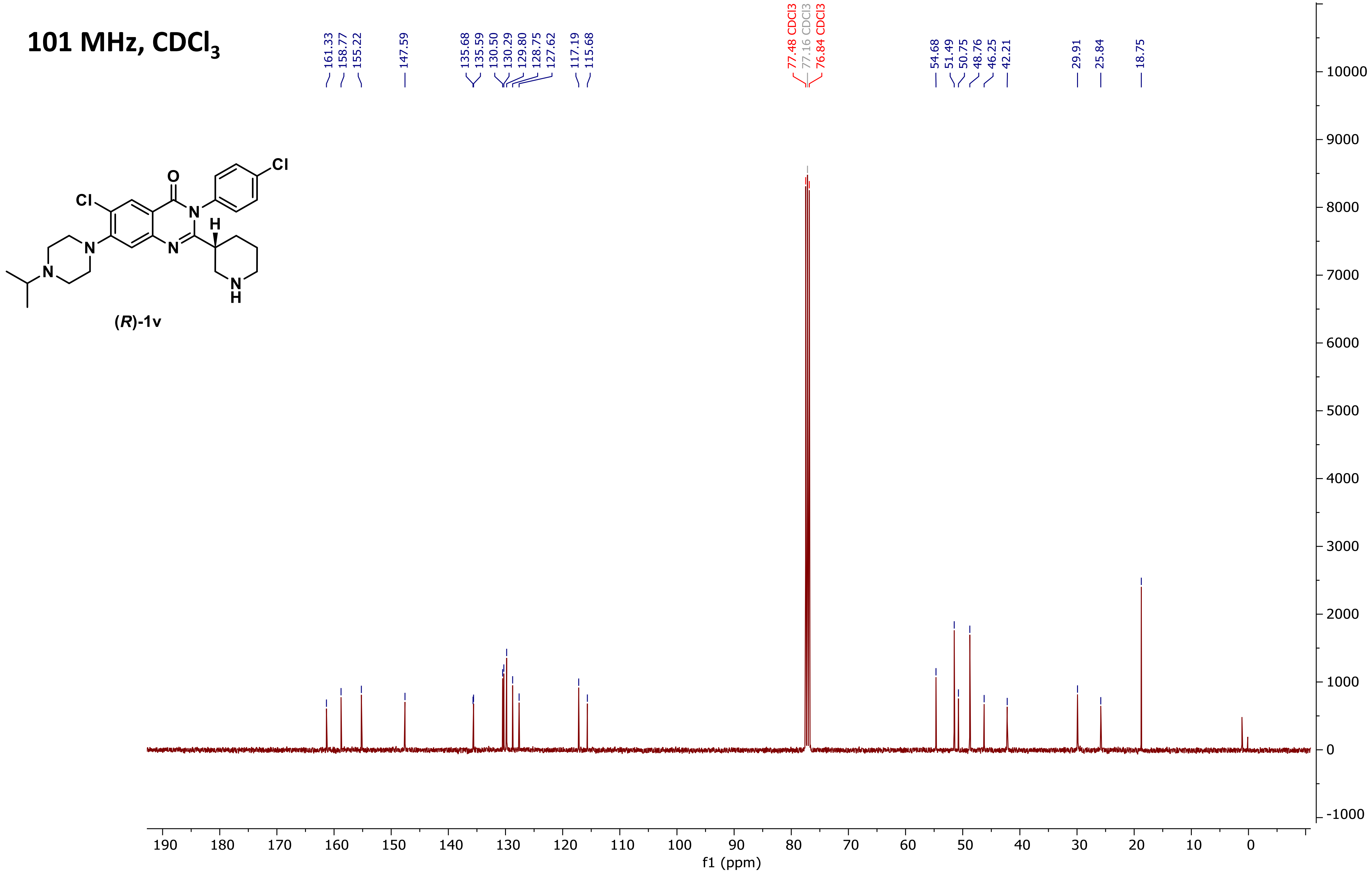

(*R*)-1v

400 MHz, $CDCl_3$

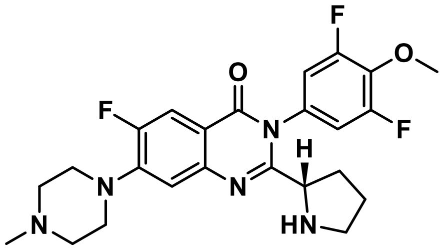

(*S*)-1a

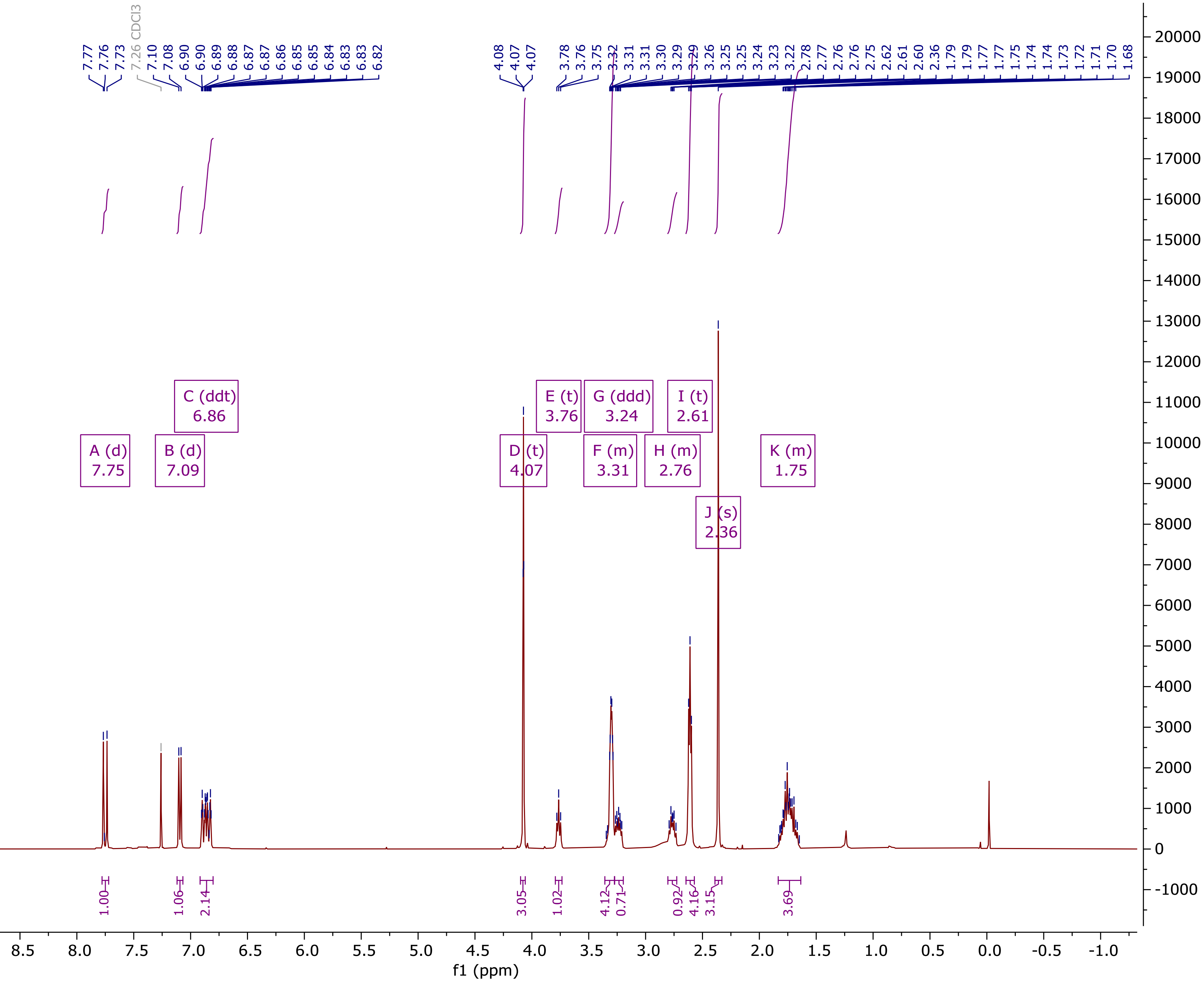

101 MHz, $CDCl_3$

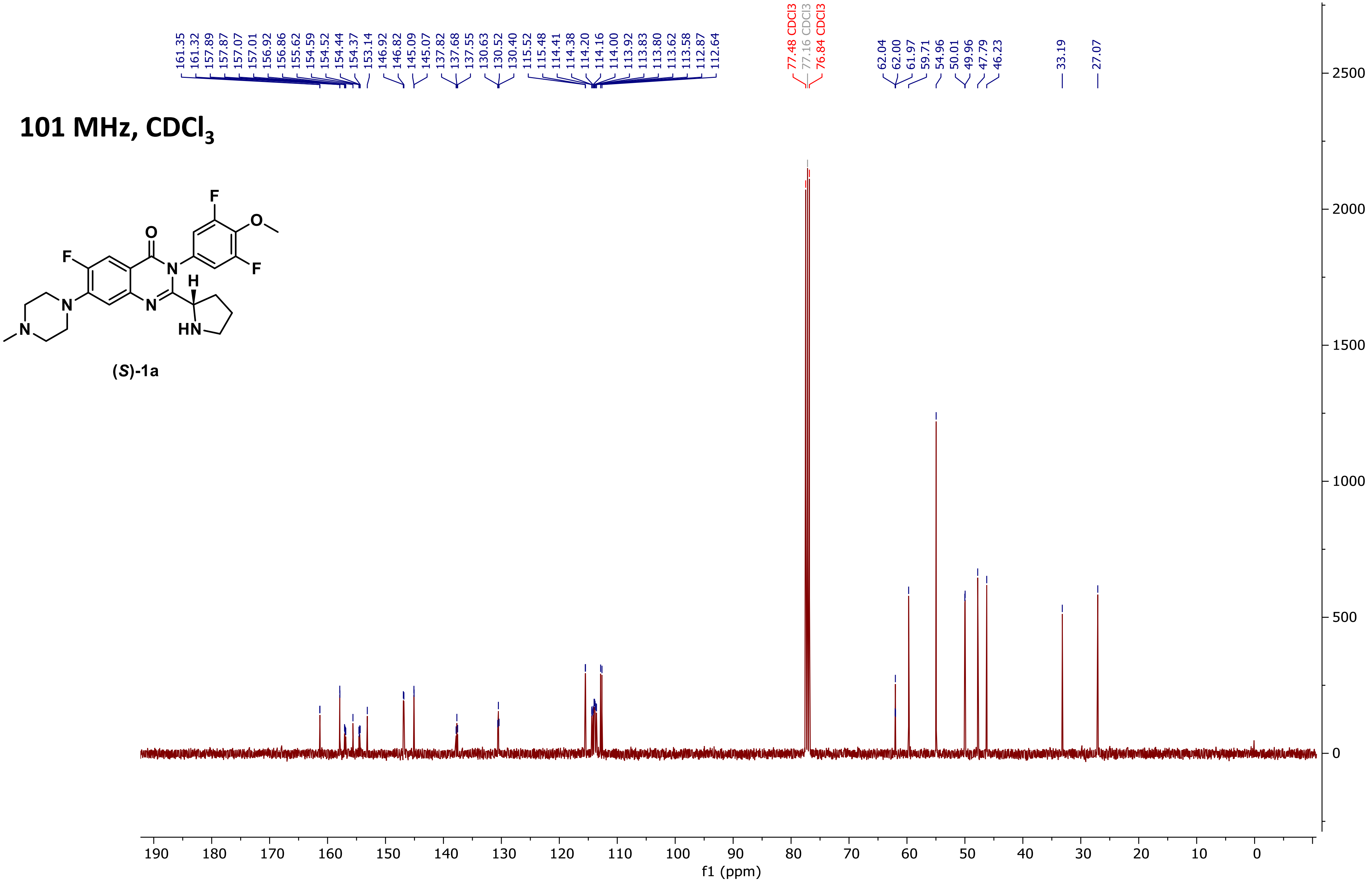

(*S*)-1a

400 MHz, $CDCl_3$

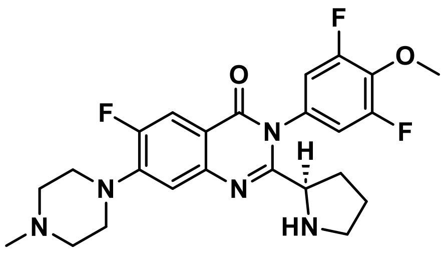

(*R*)-1a

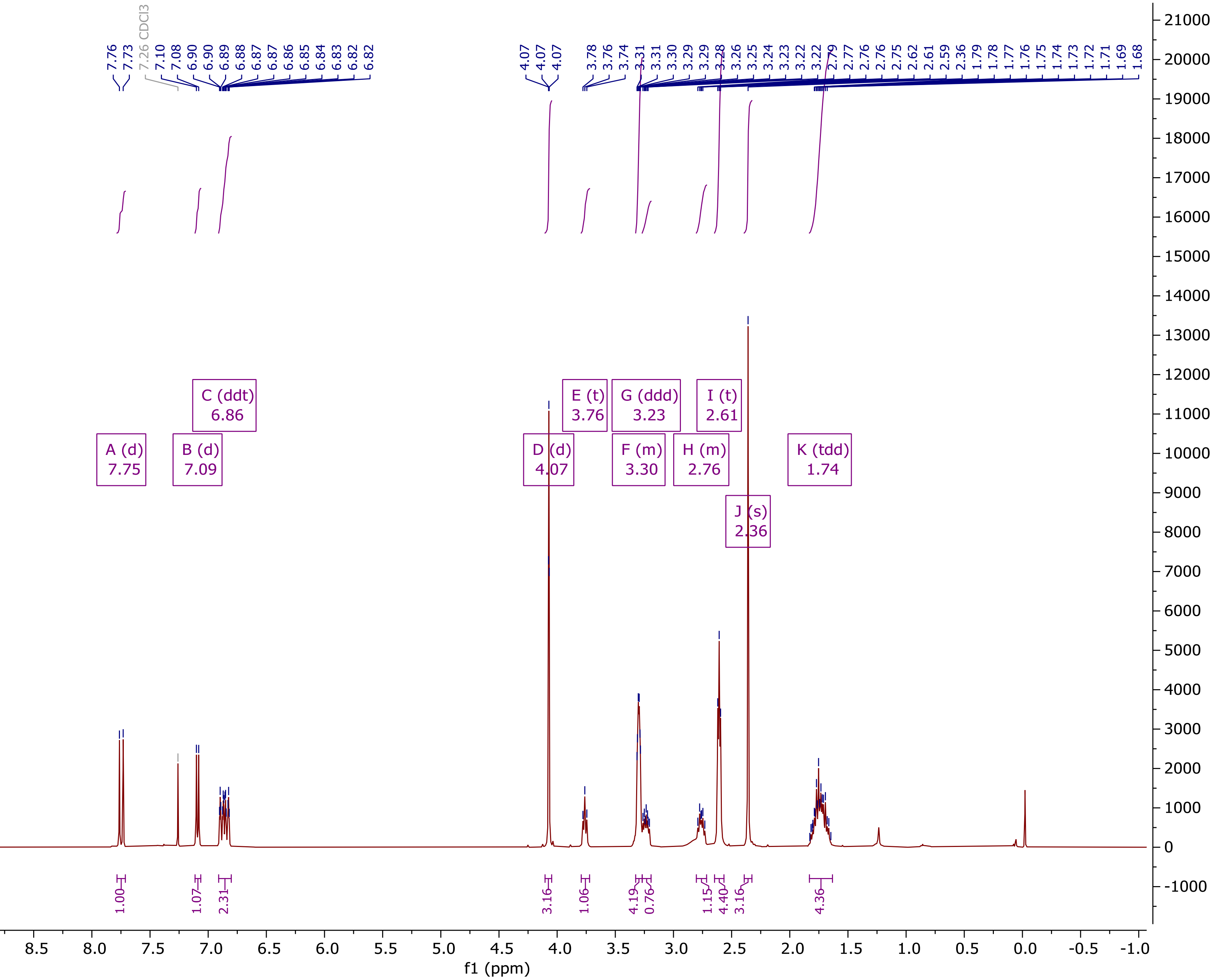

101 MHz, $CDCl_3$

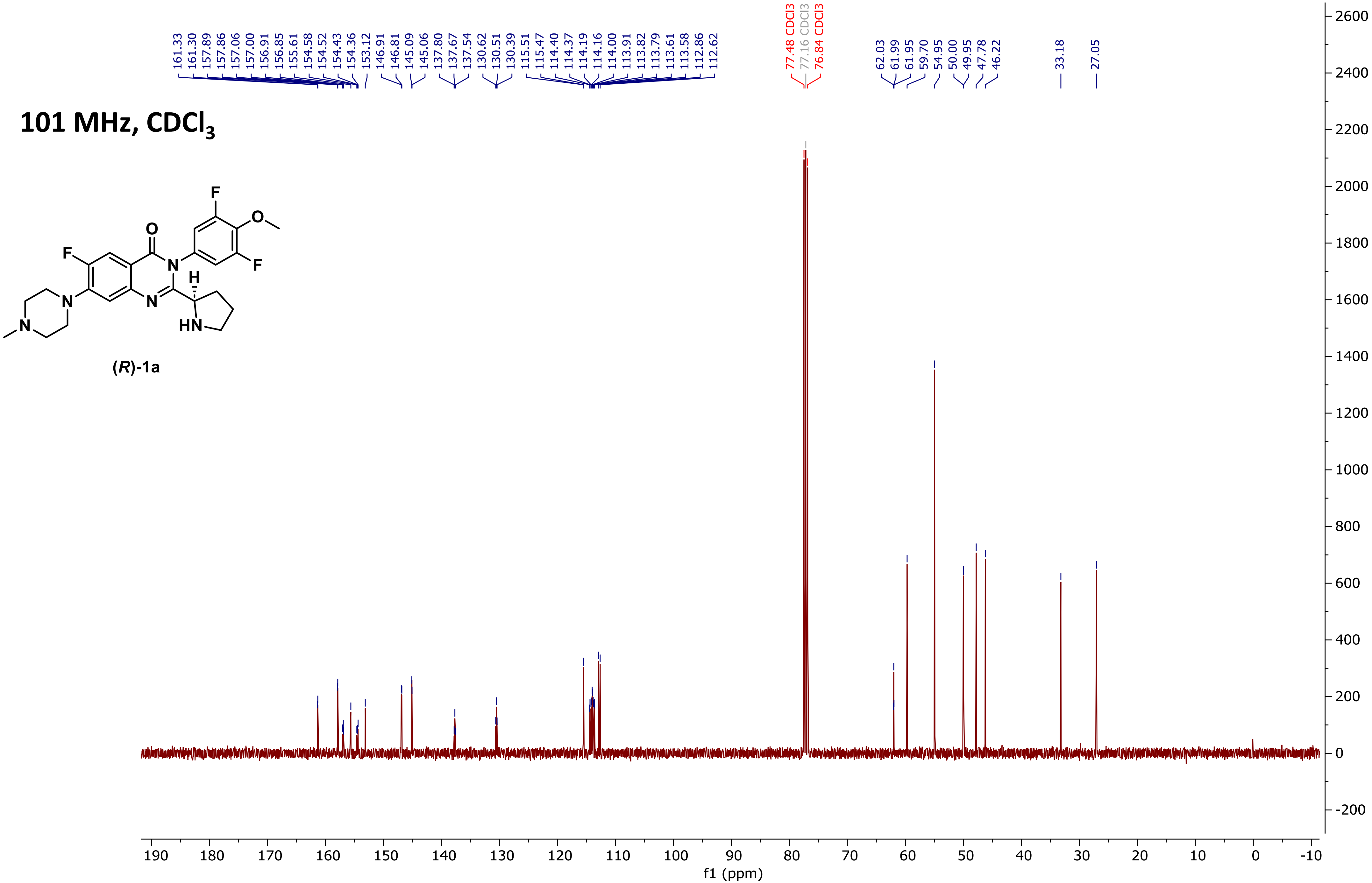

(*R*)-1a

400 MHz, CDCl$_3$

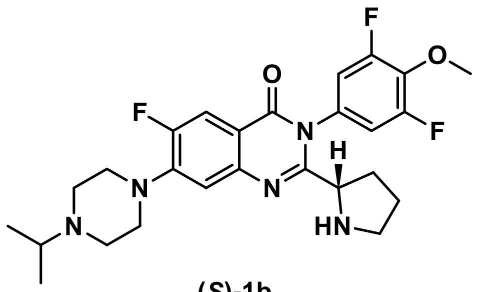

(*S*)-1b

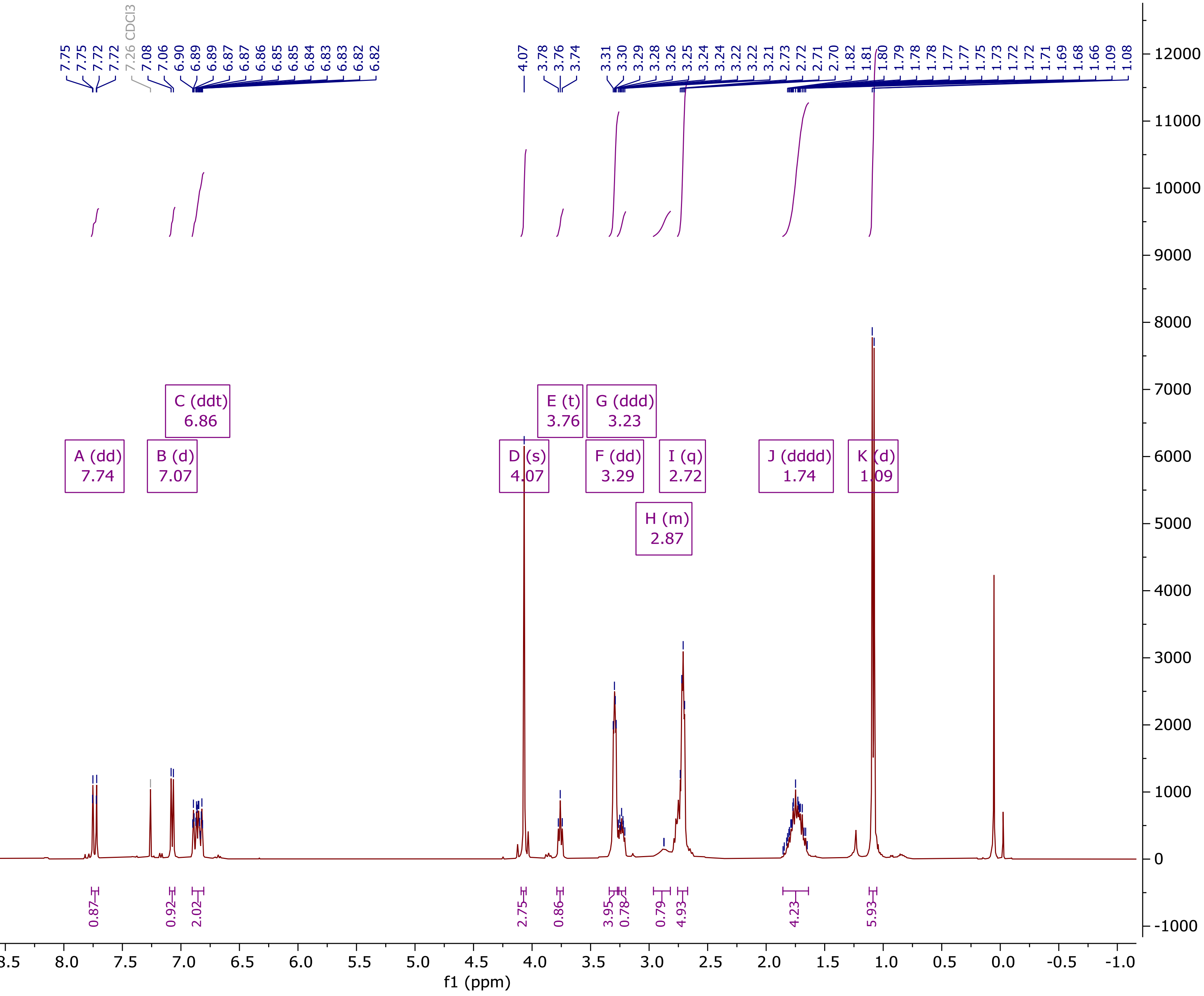

101 MHz, $CDCl_3$

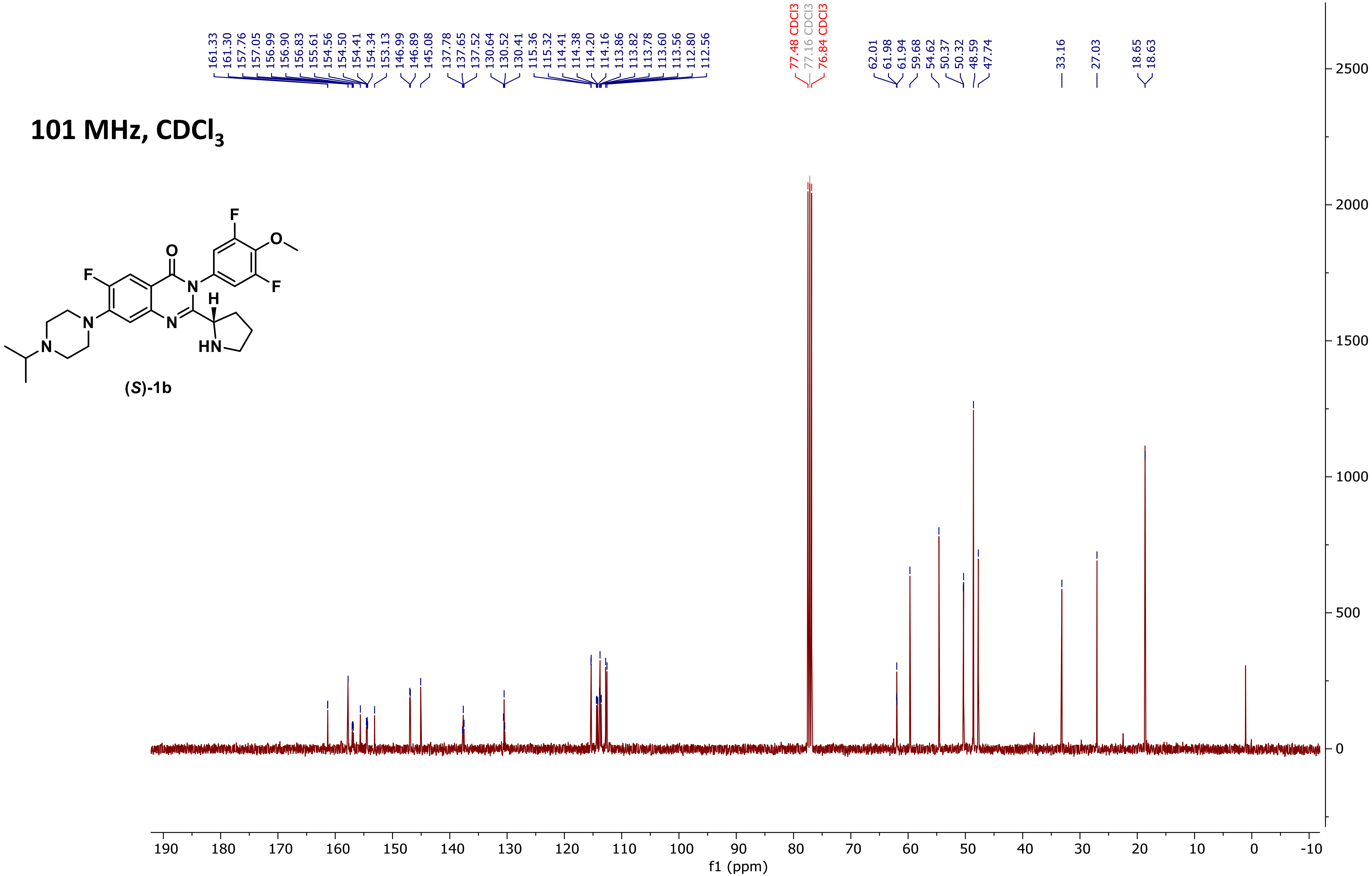

400 MHz, $CDCl_3$

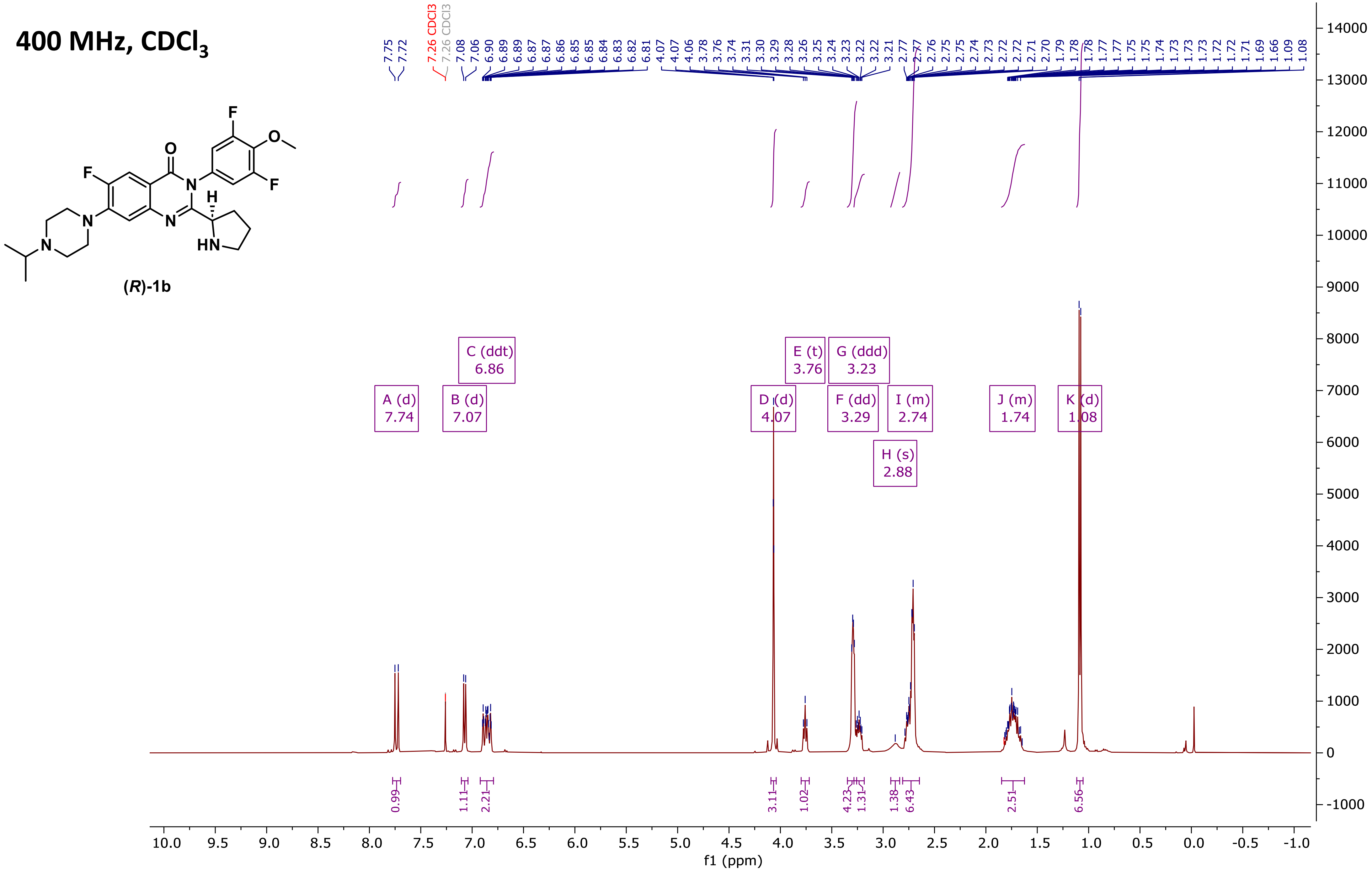

101 MHz, $CDCl_3$

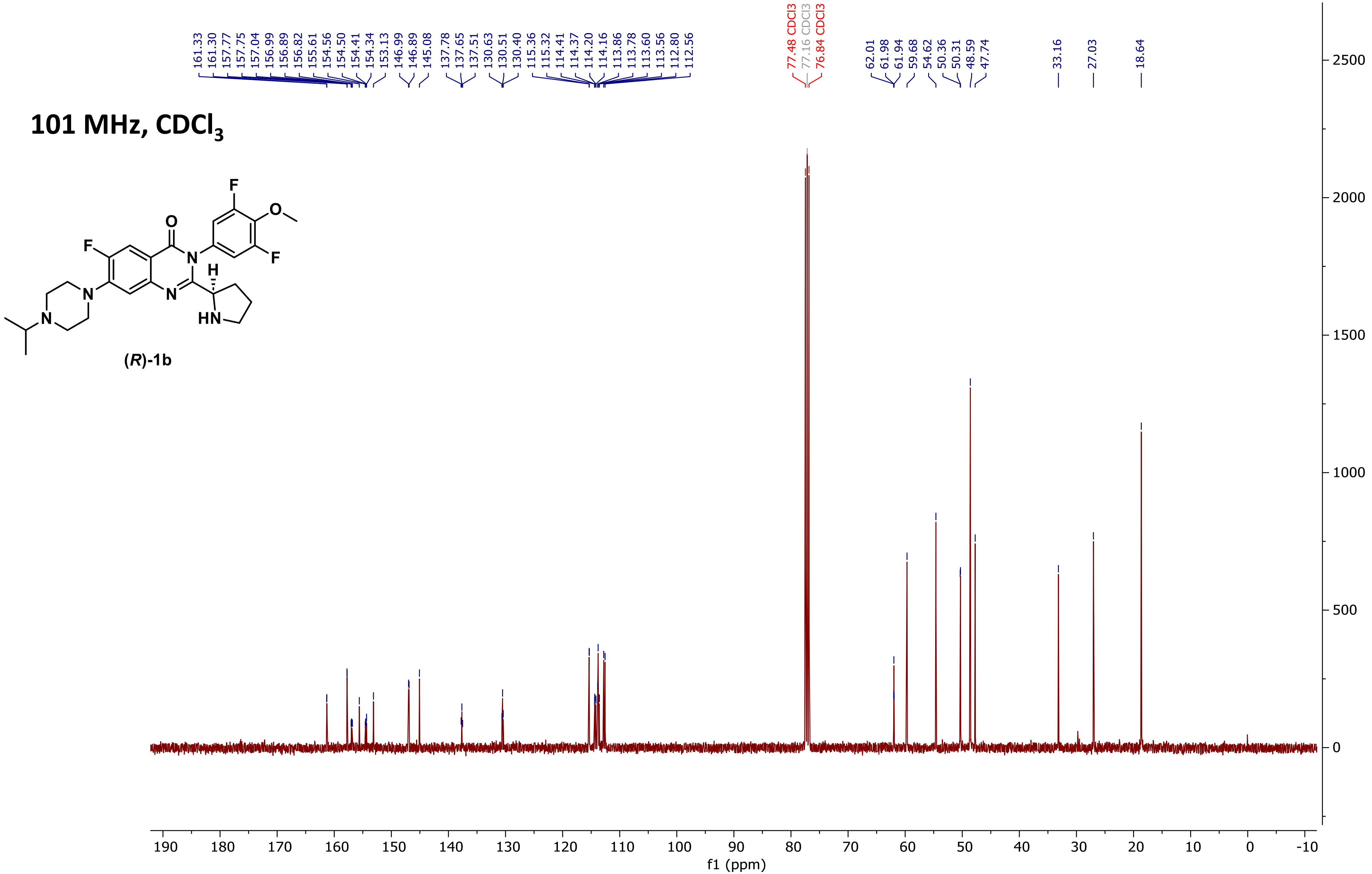

400 MHz, $CDCl_3$

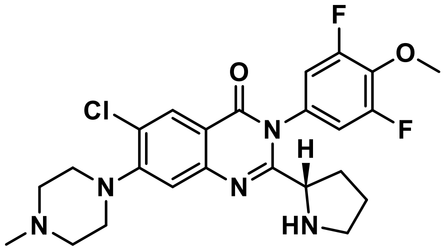

(*S*-1c

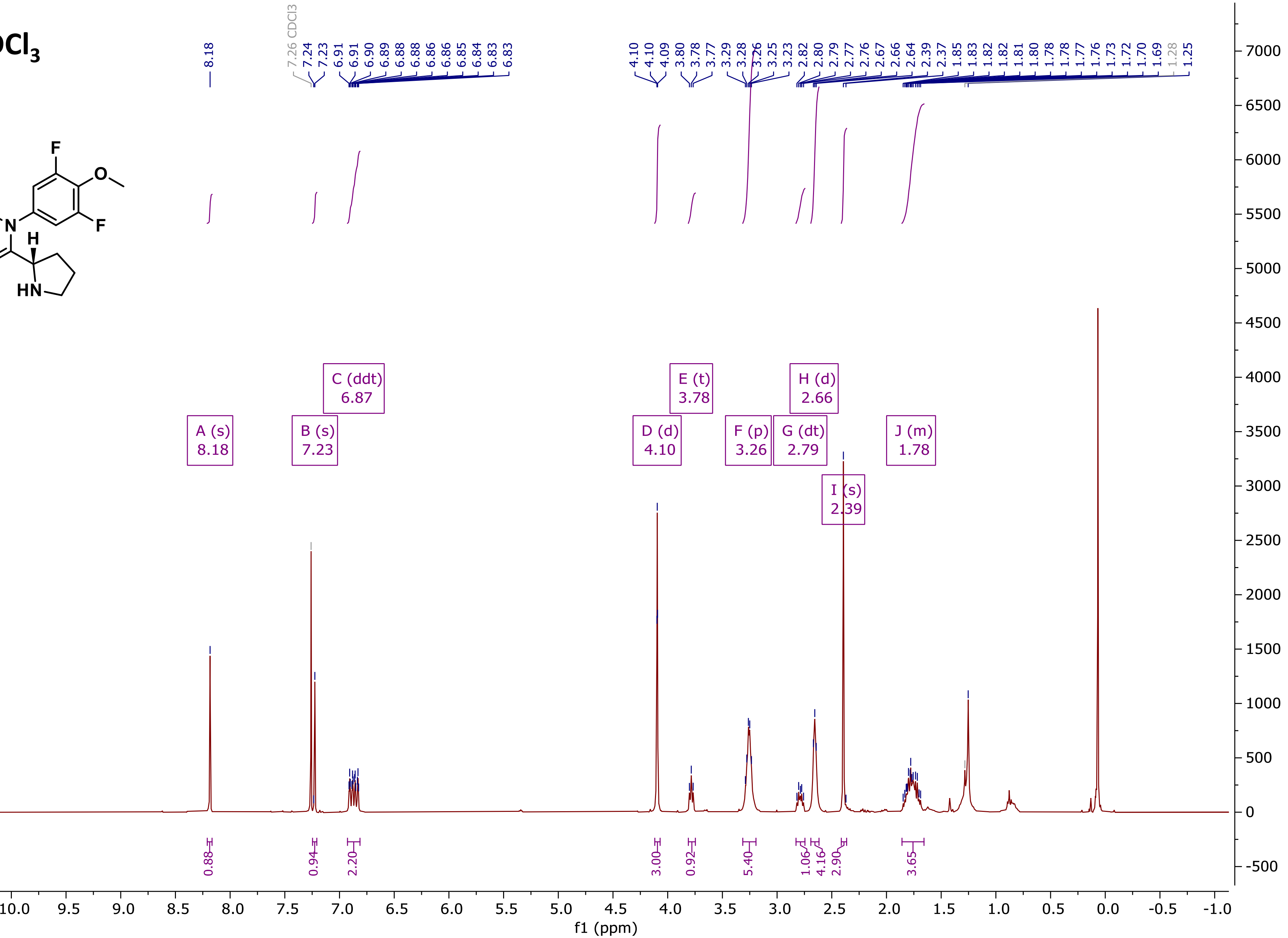

101 MHz, $CDCl_3$

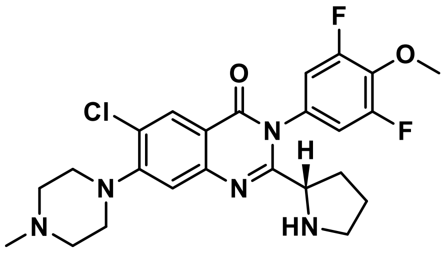

(*S*)-1c

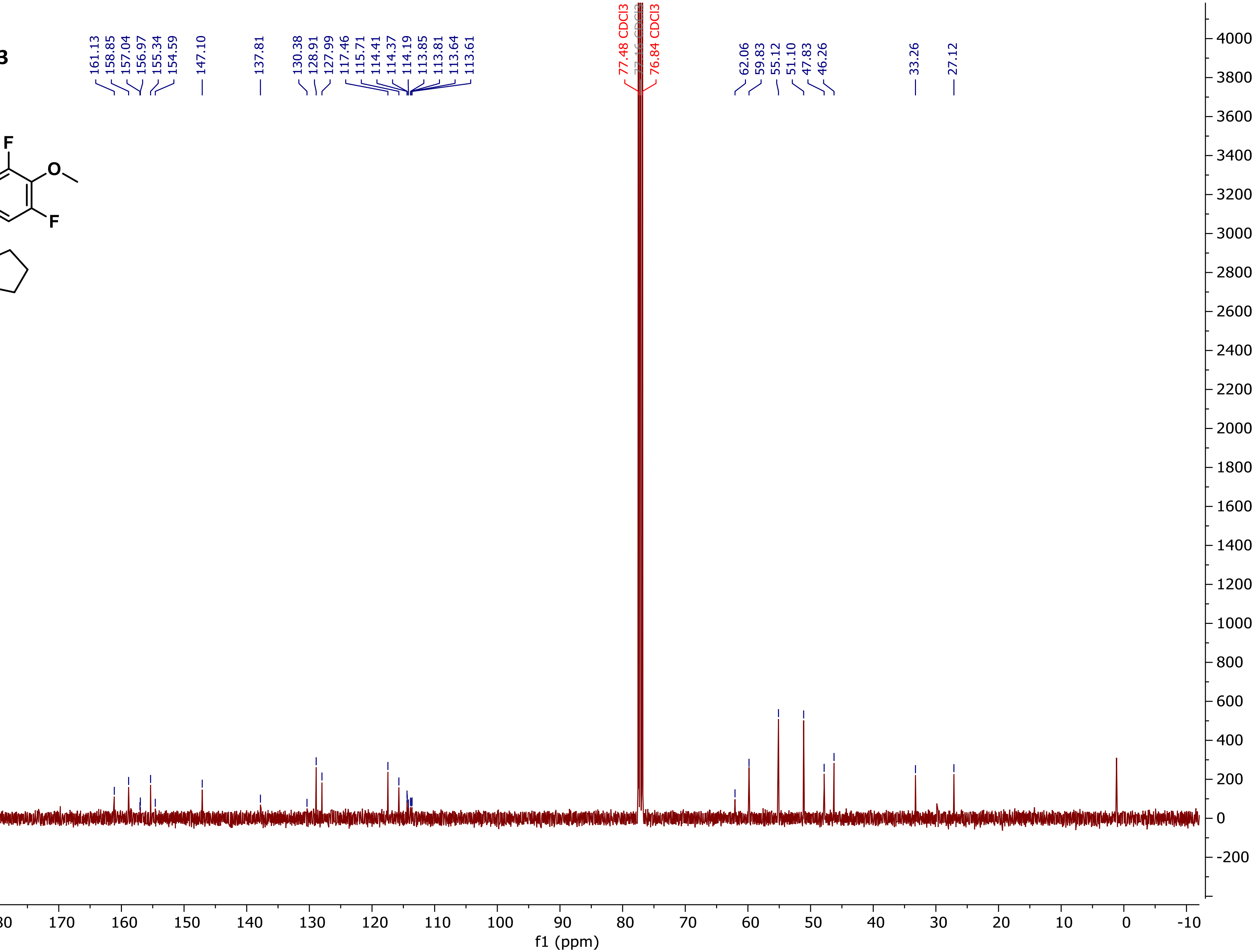

400 MHz, $CDCl_3$

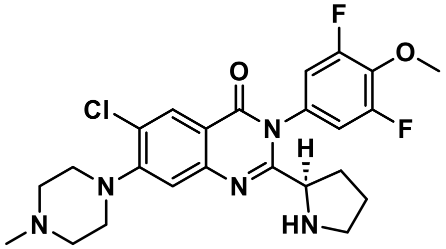

(*R*)-1c

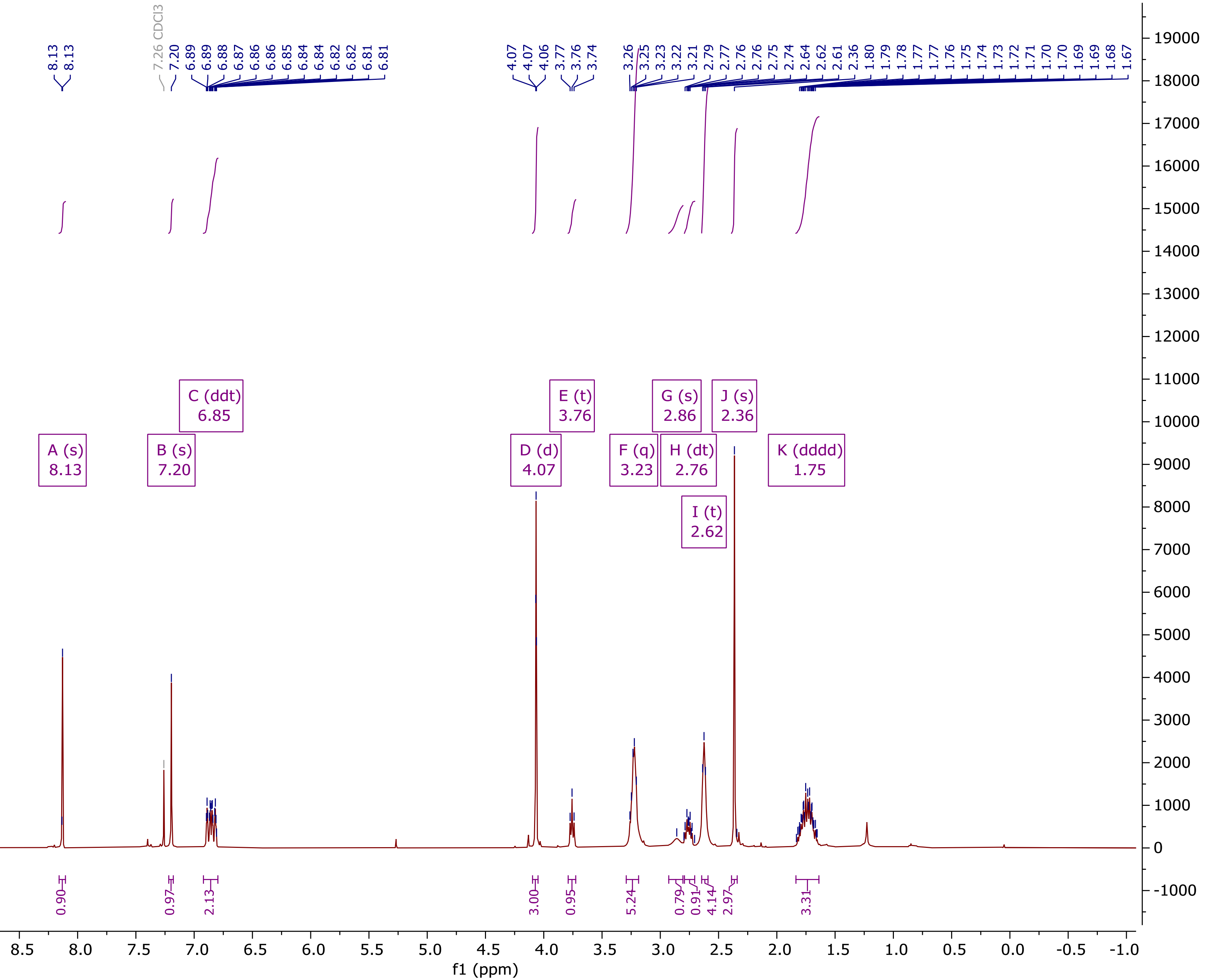

101 MHz, $CDCl_3$

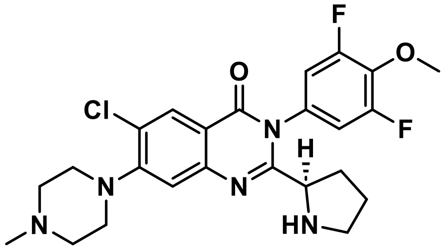

(*R*)-1c

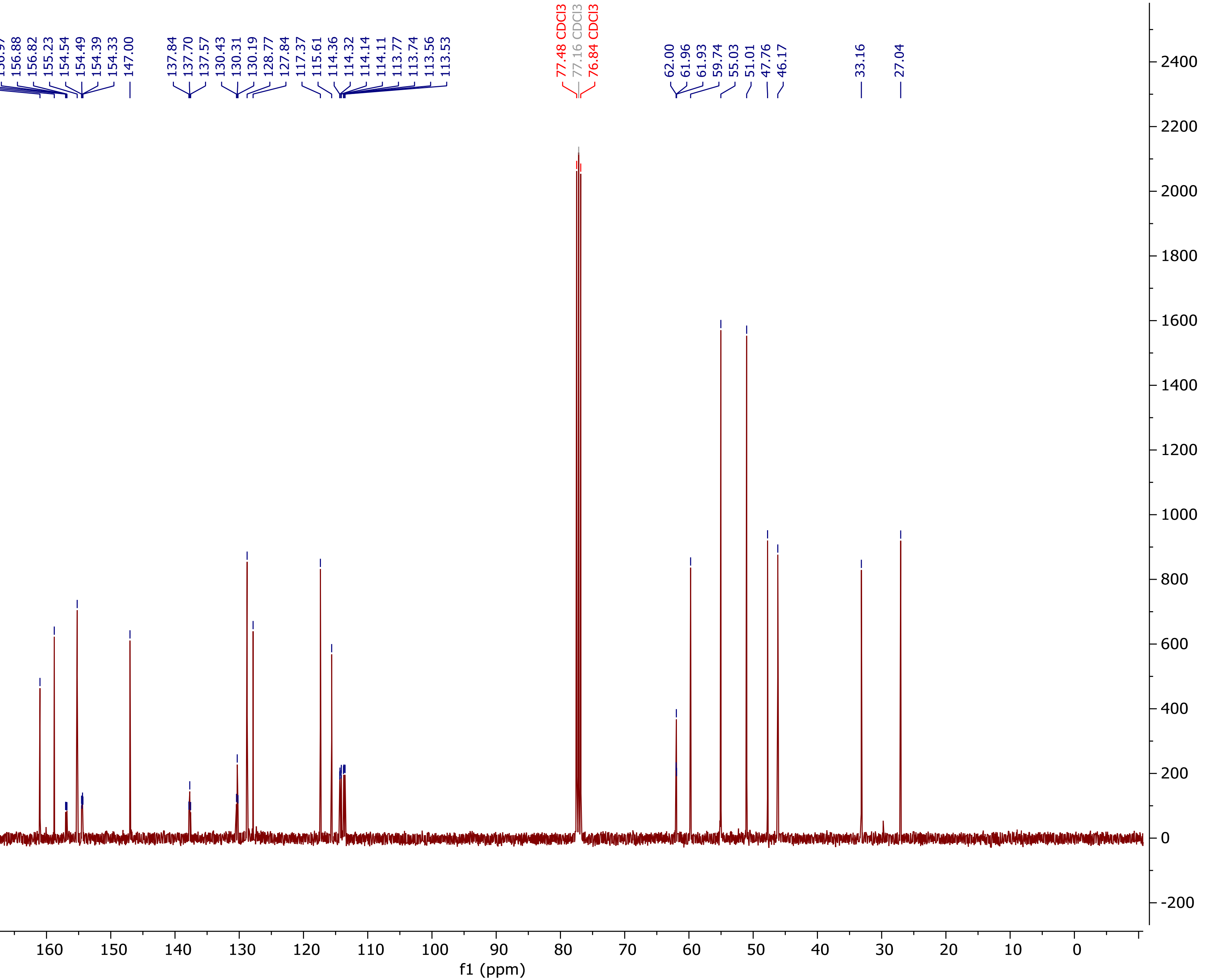

400 MHz, $CDCl_3$

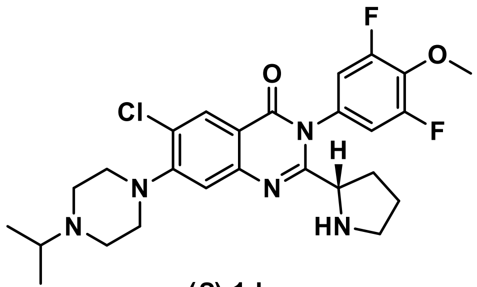

(*S*)-1d

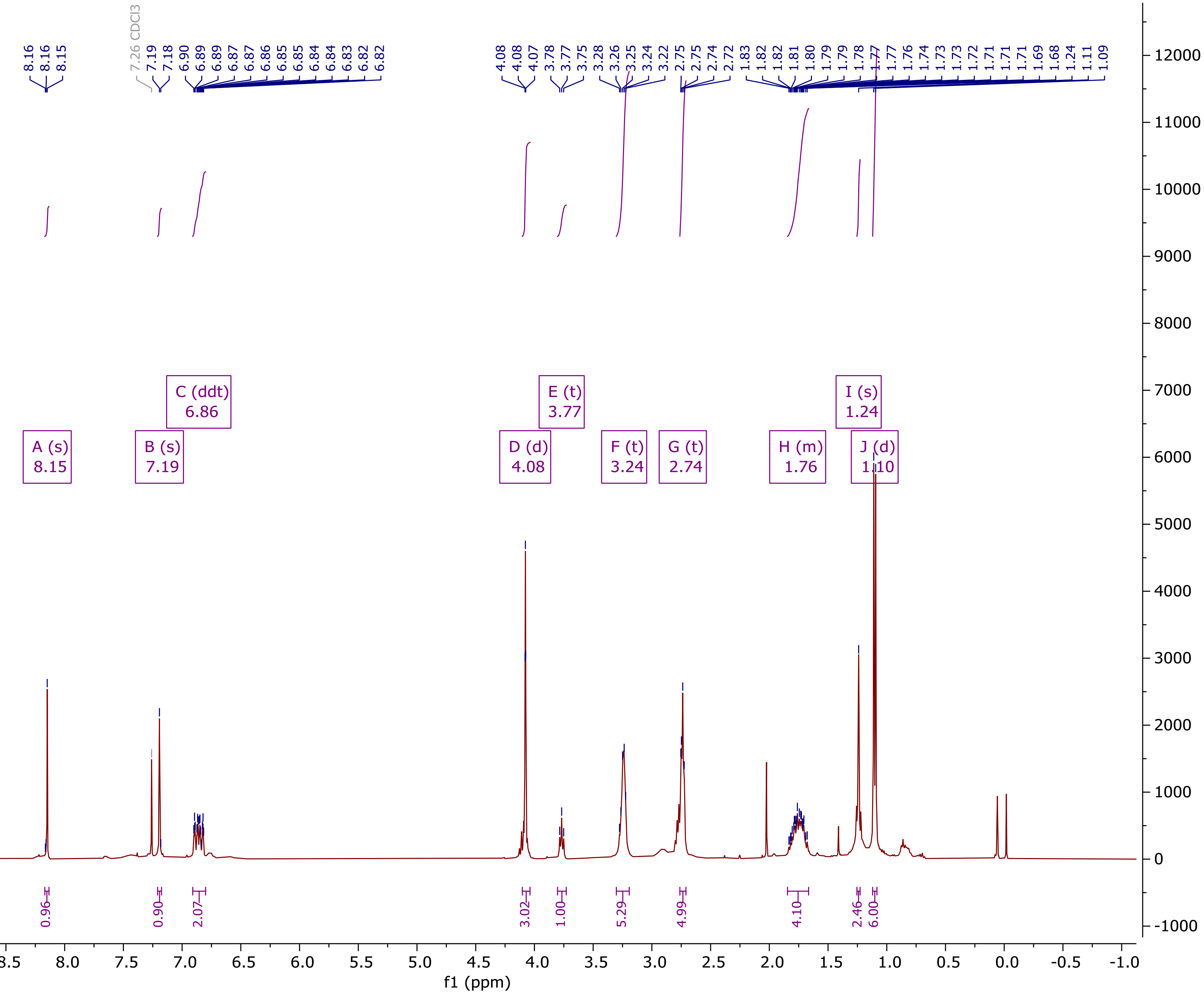

101 MHz, $CDCl_3$

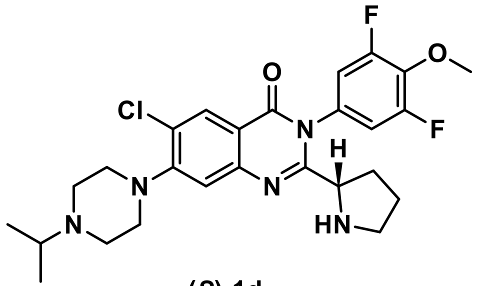

(*S*)-1d

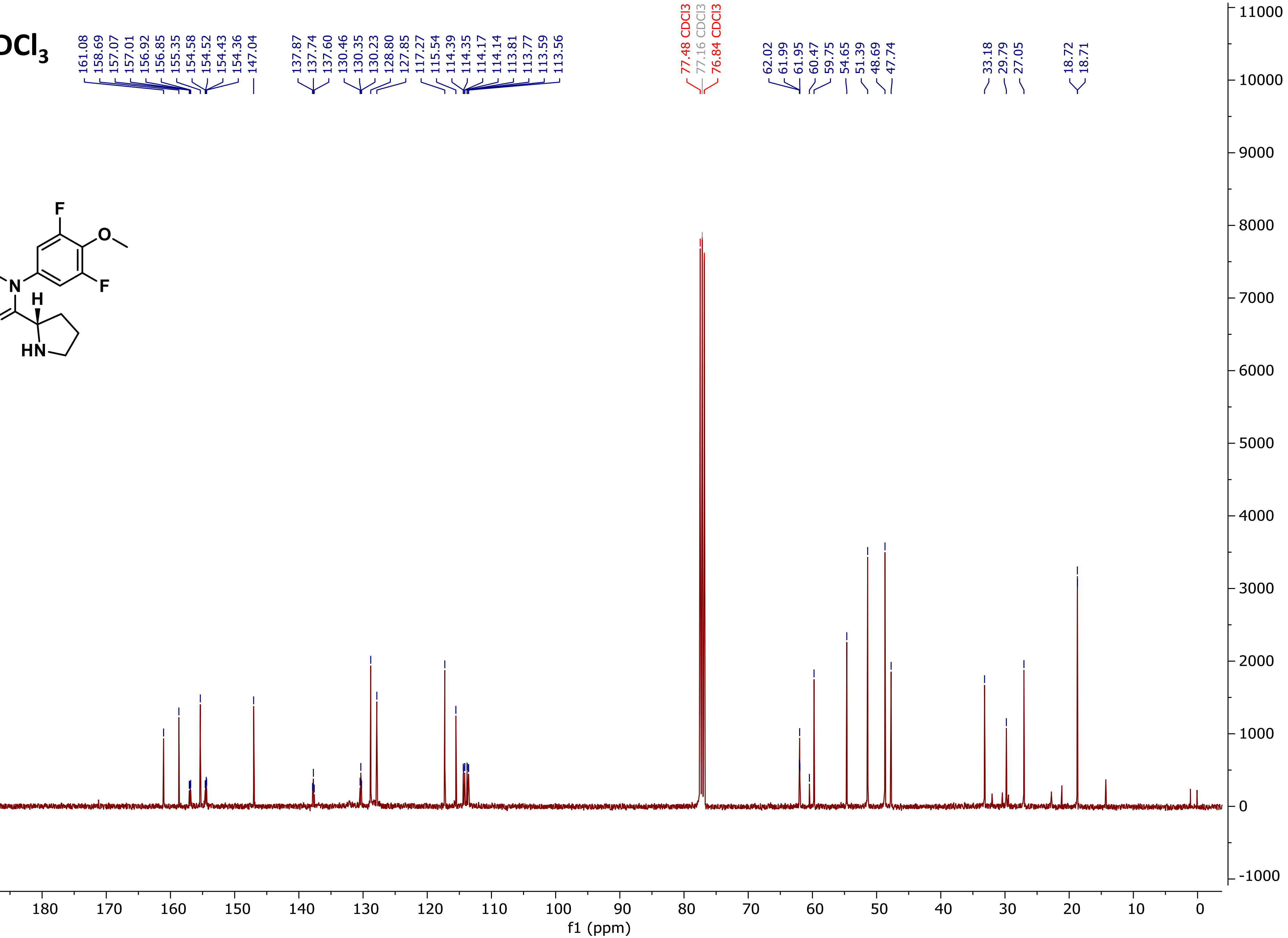

400 MHz, CDCl$_3$

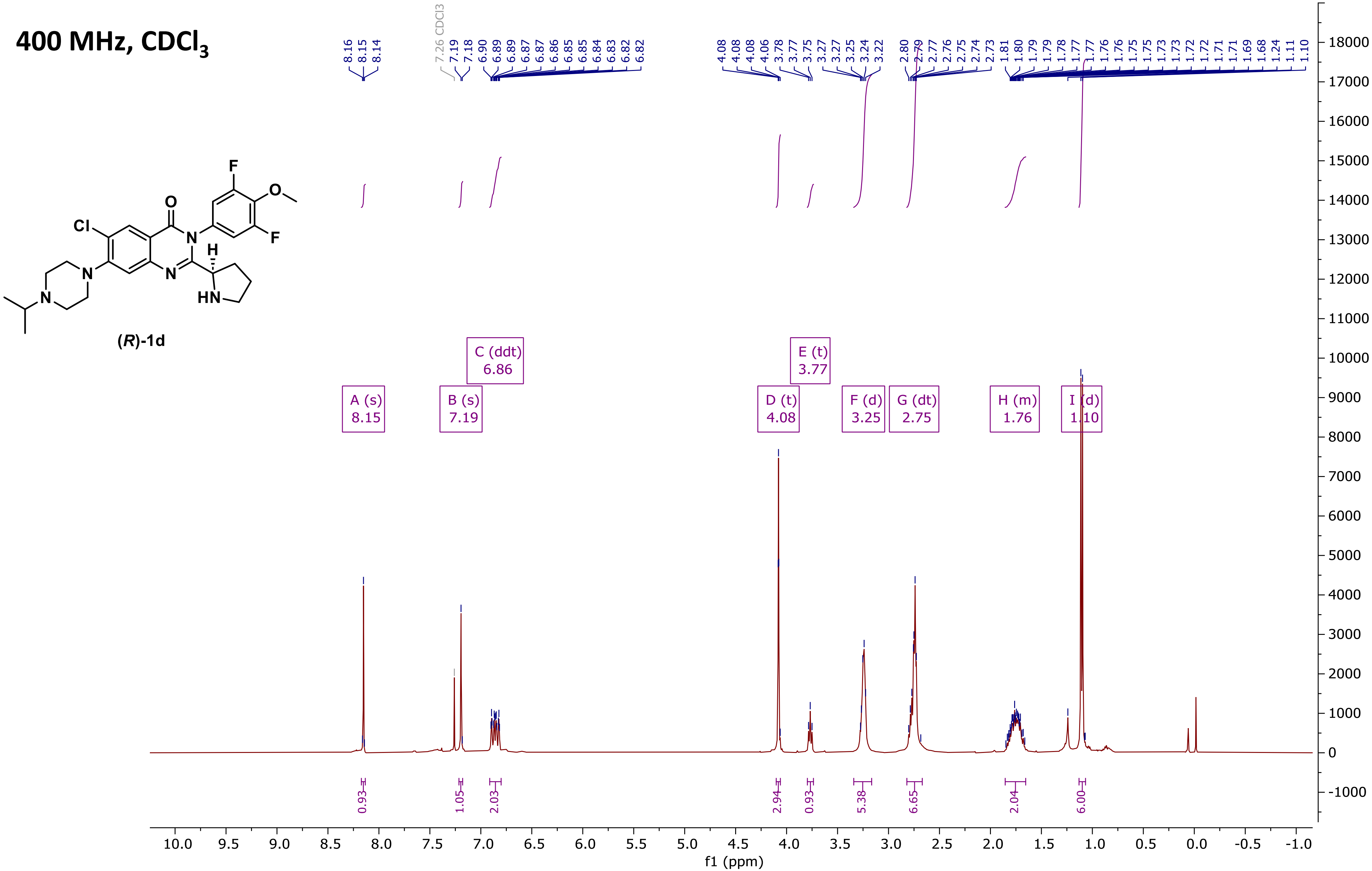

(*R*)-1d

101 MHz, $CDCl_3$

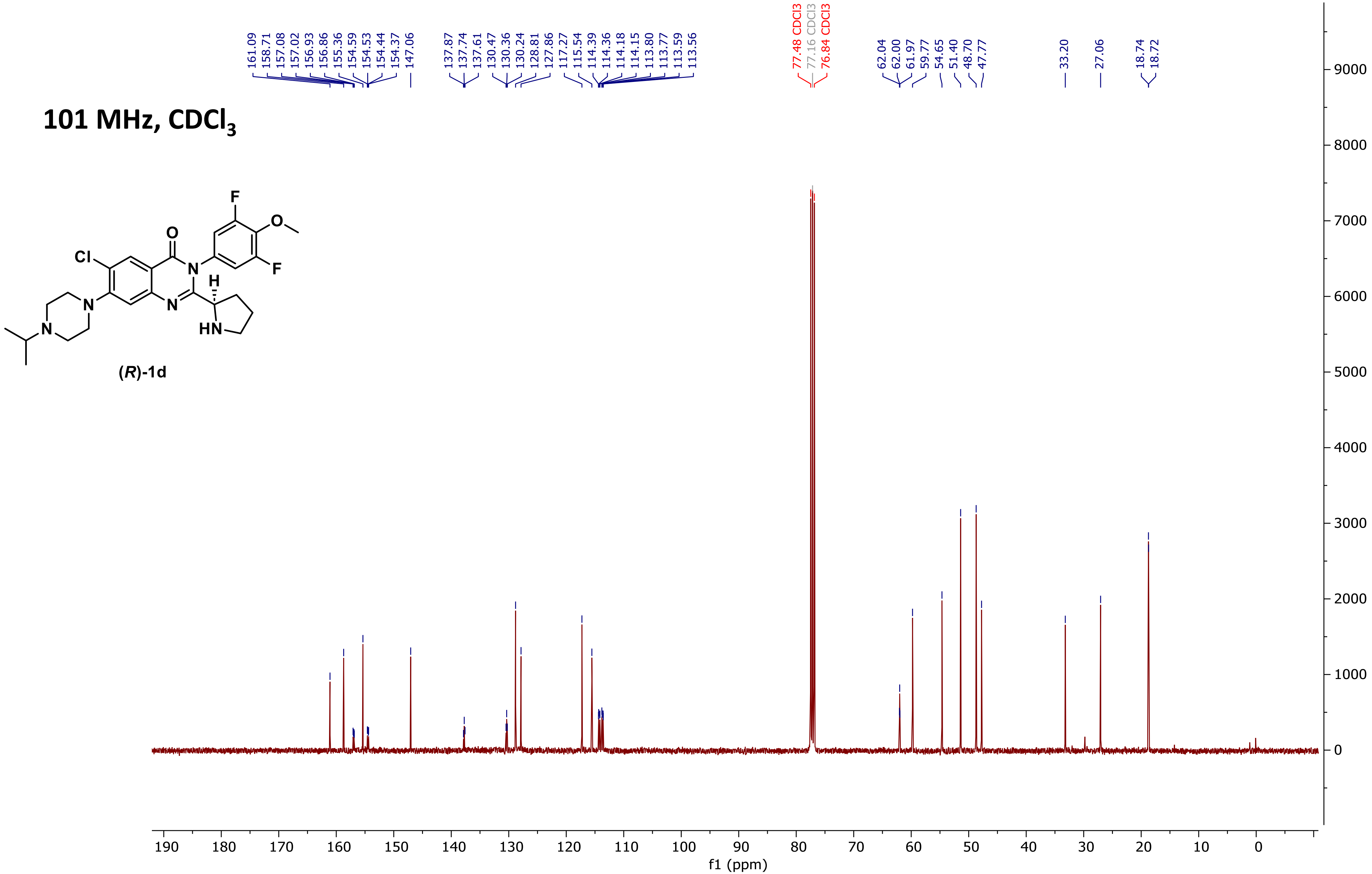

(*R*)-1d

400 MHz, $CDCl_3$

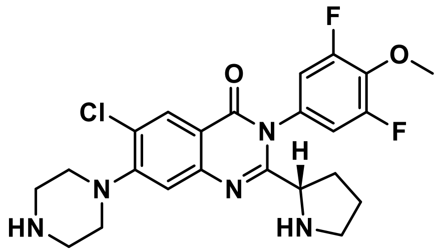

(*S*)-1e

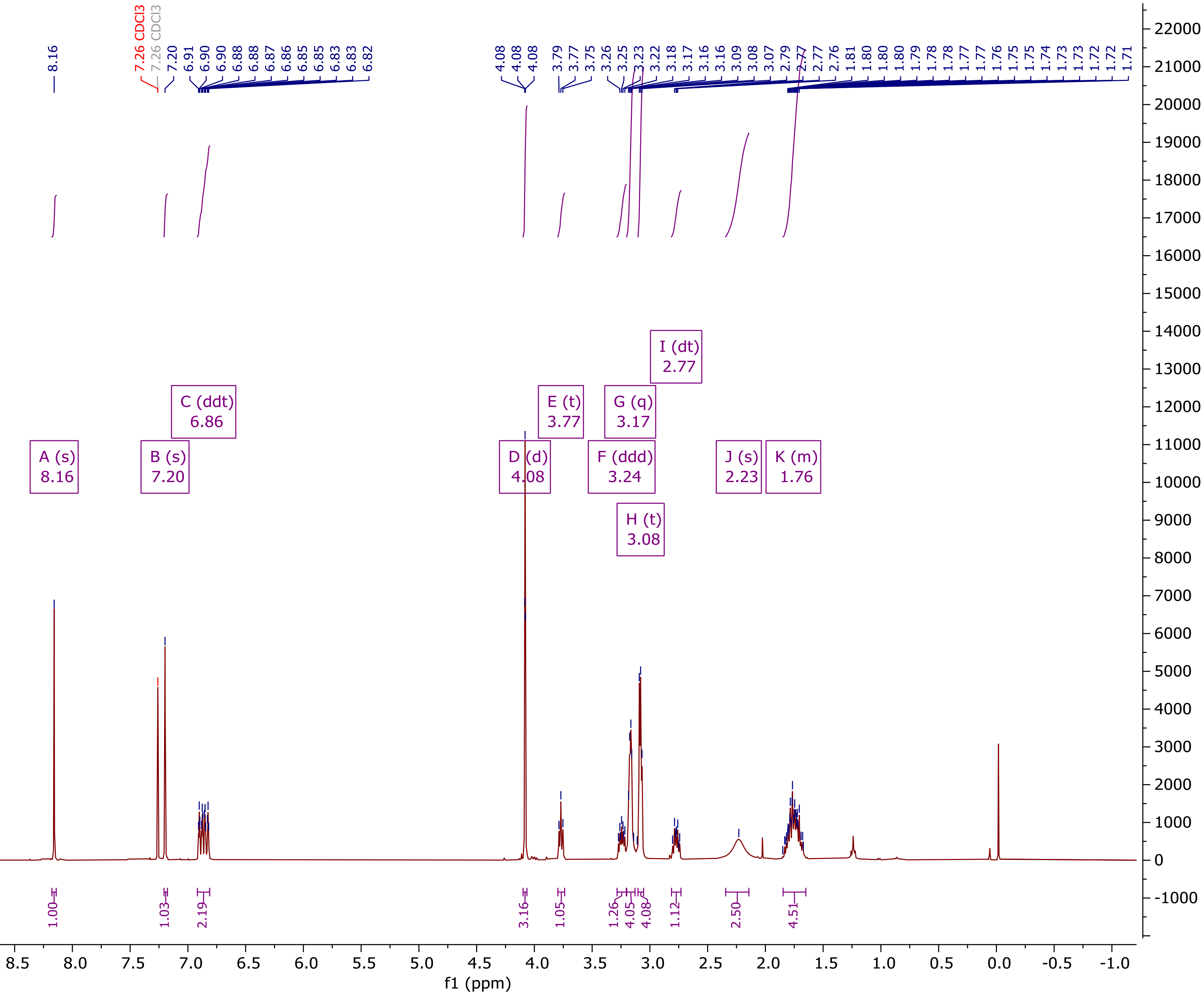

101 MHz, $CDCl_3$

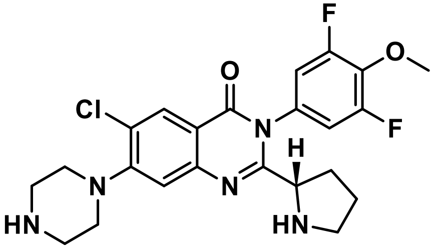

(*S*)-1e

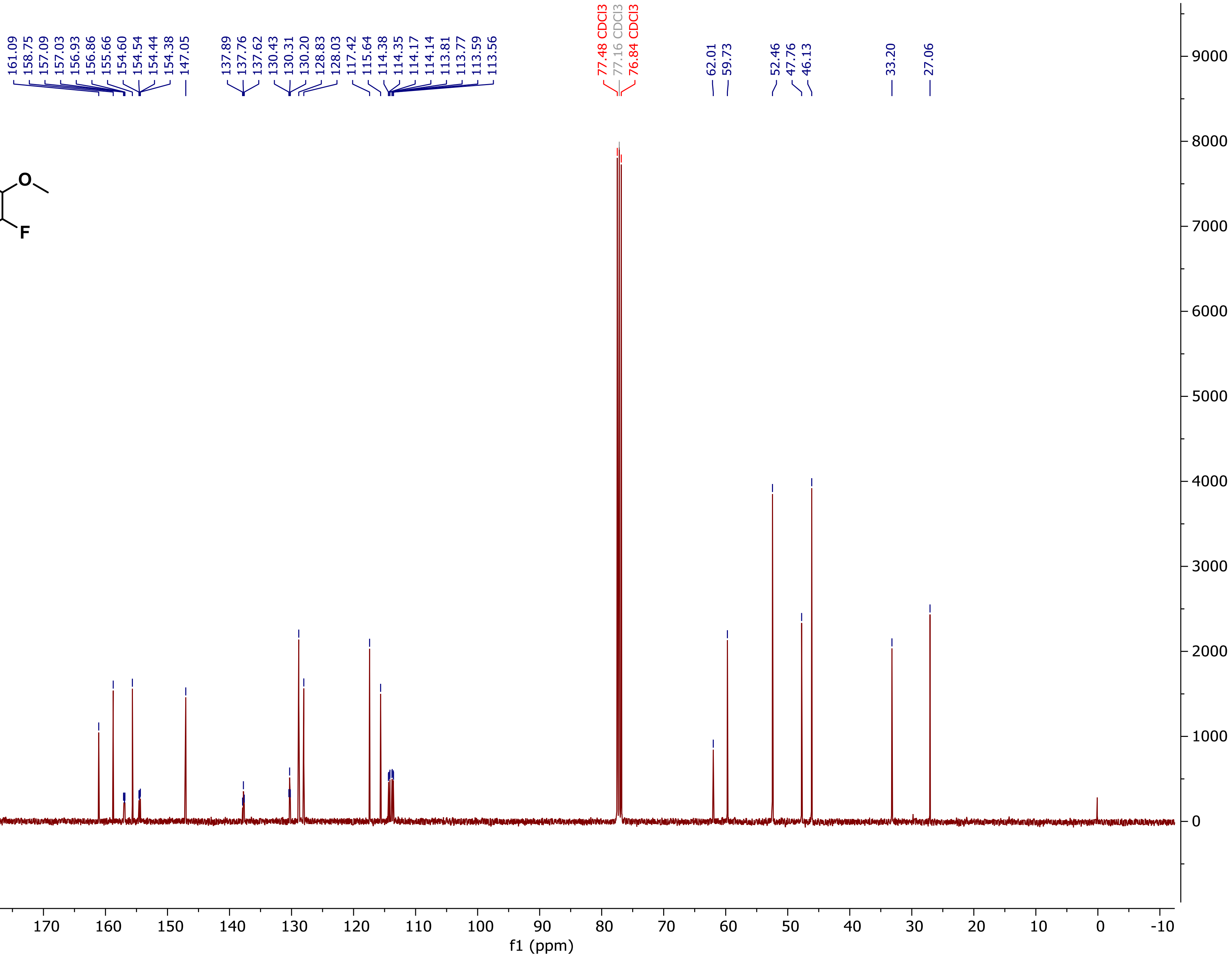

400 MHz, $CDCl_3$

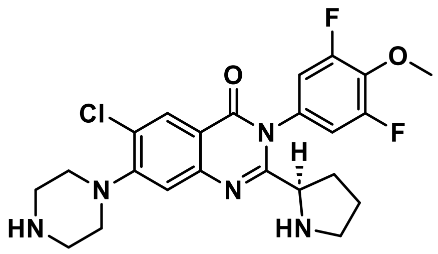

(*R*)-1e

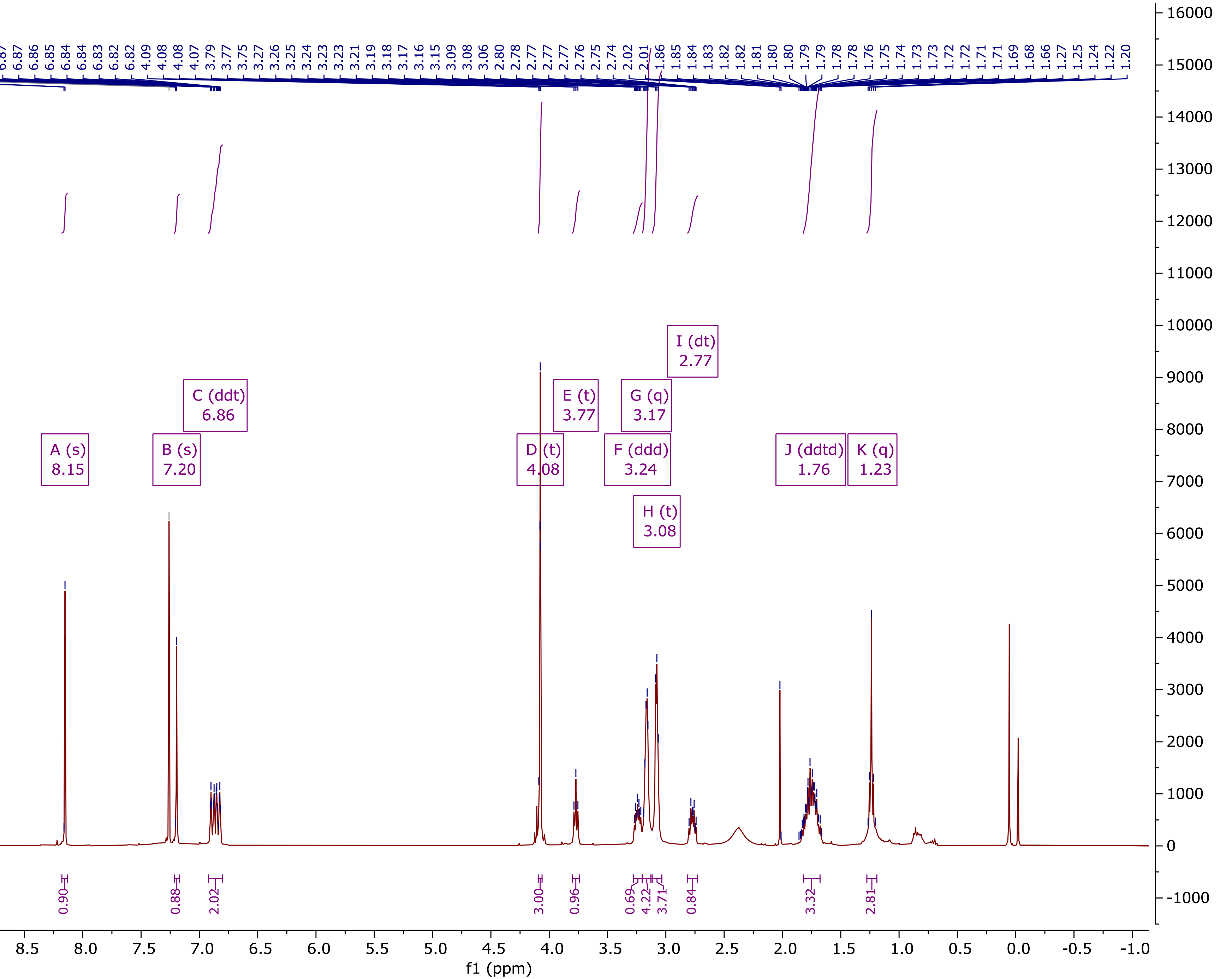

101 MHz, $CDCl_3$

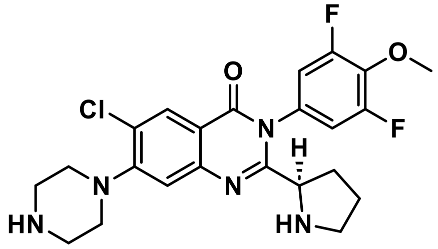

(*R*)-1e

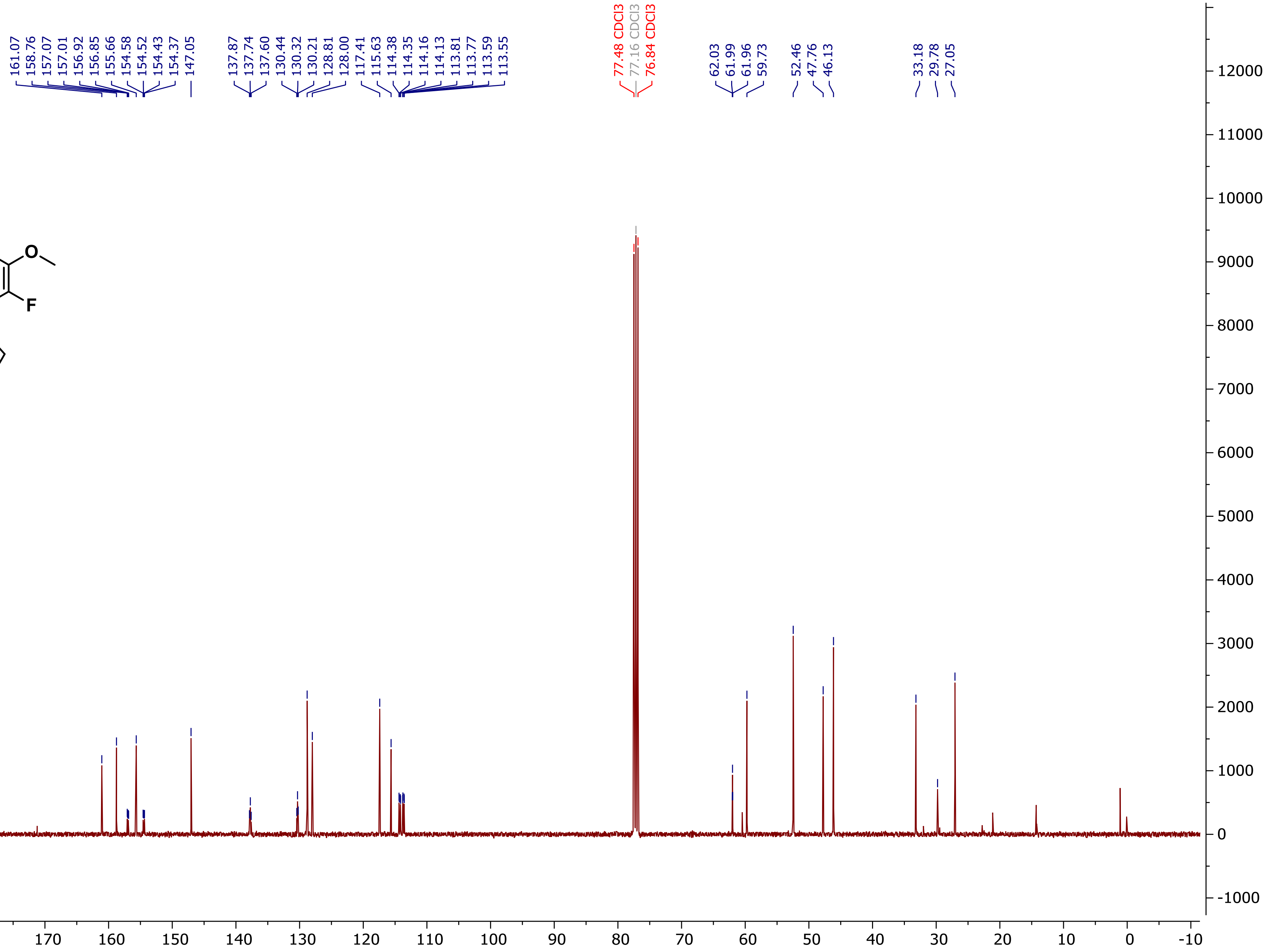

400 MHz, $CDCl_3$

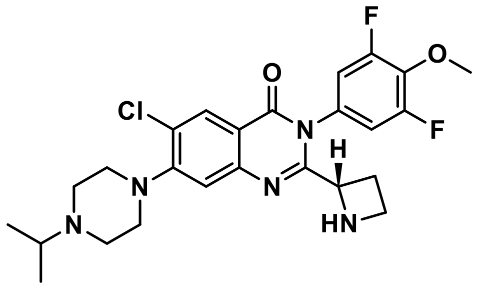

(*S*)-1f

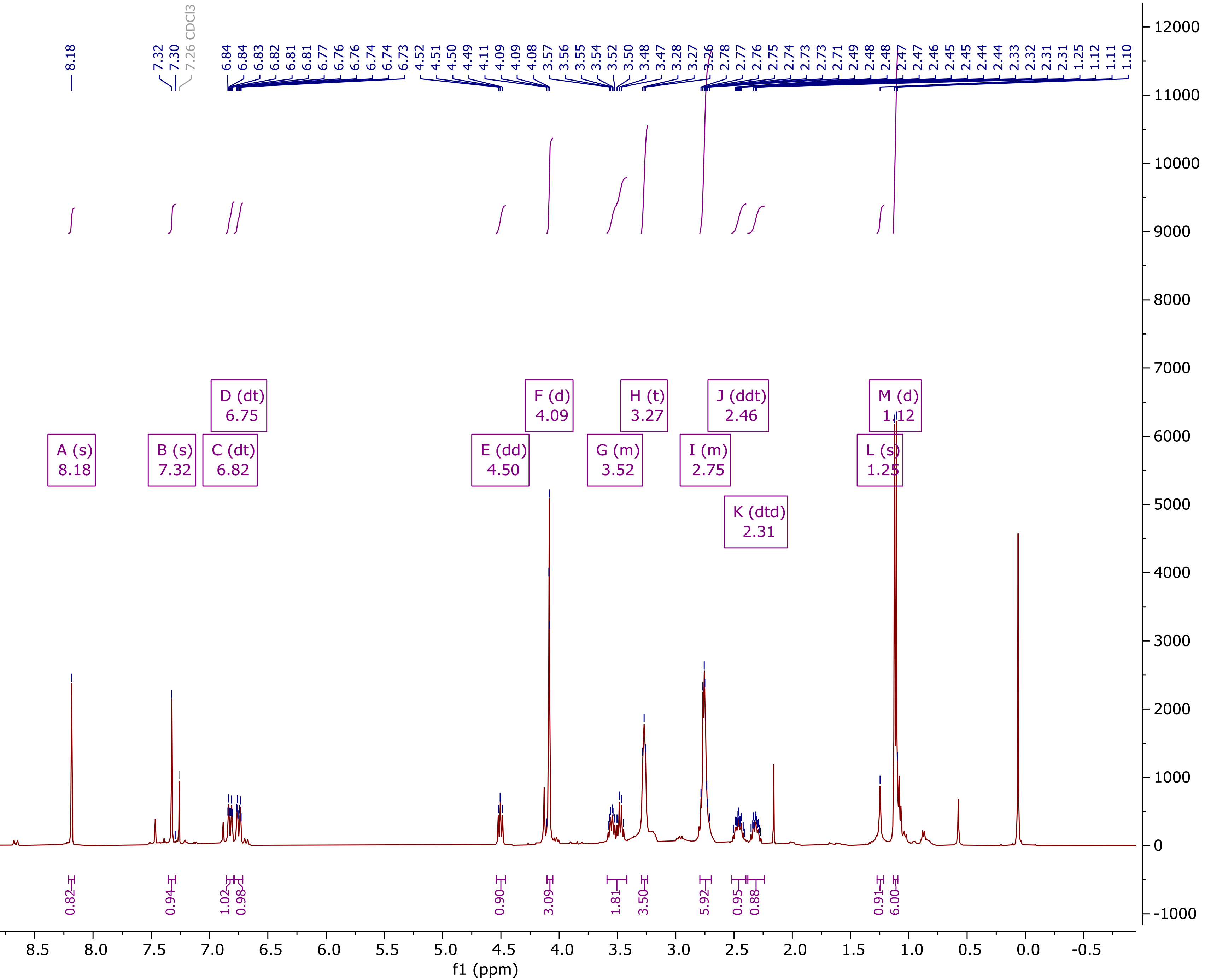

101 MHz, $CDCl_3$

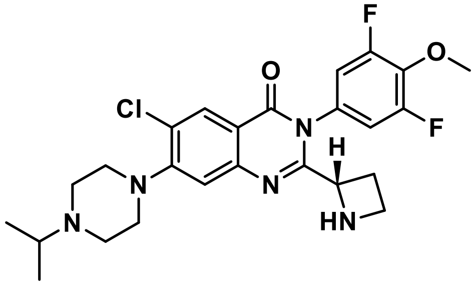

(*S*)-1f

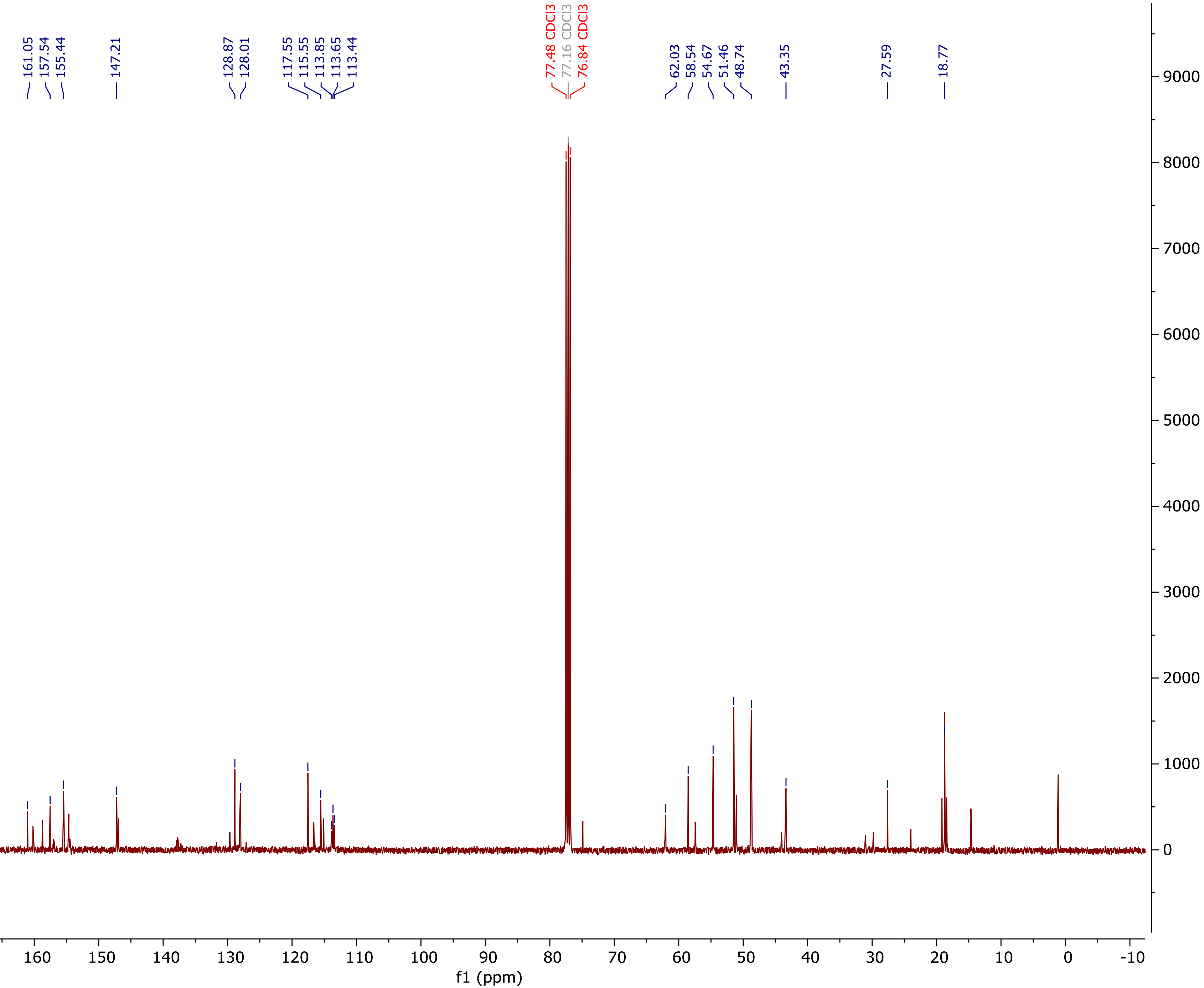

400 MHz, $CDCl_3$

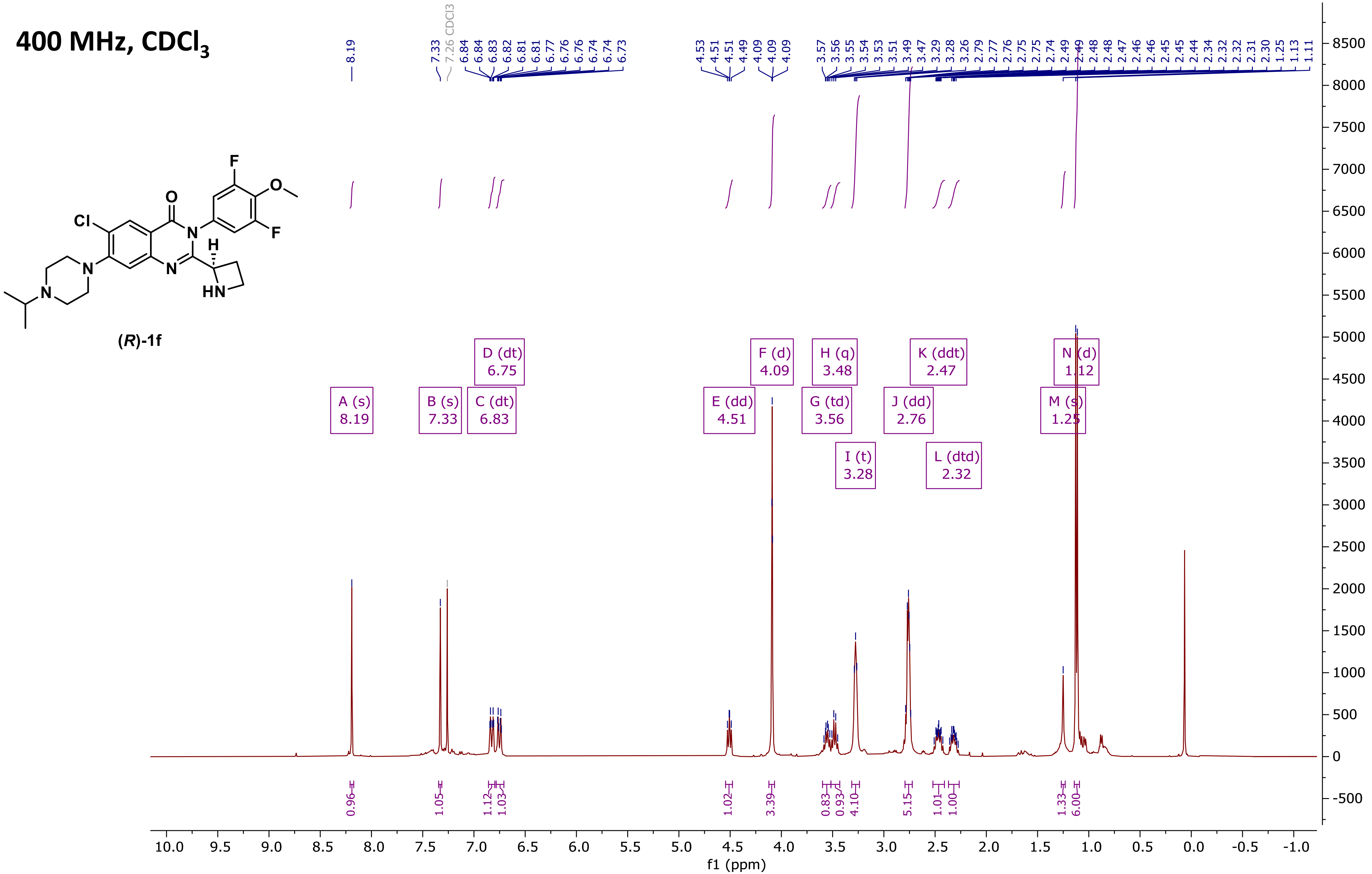

101 MHz, $CDCl_3$

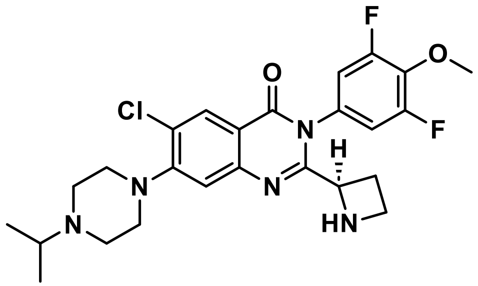

(*R*)-1f

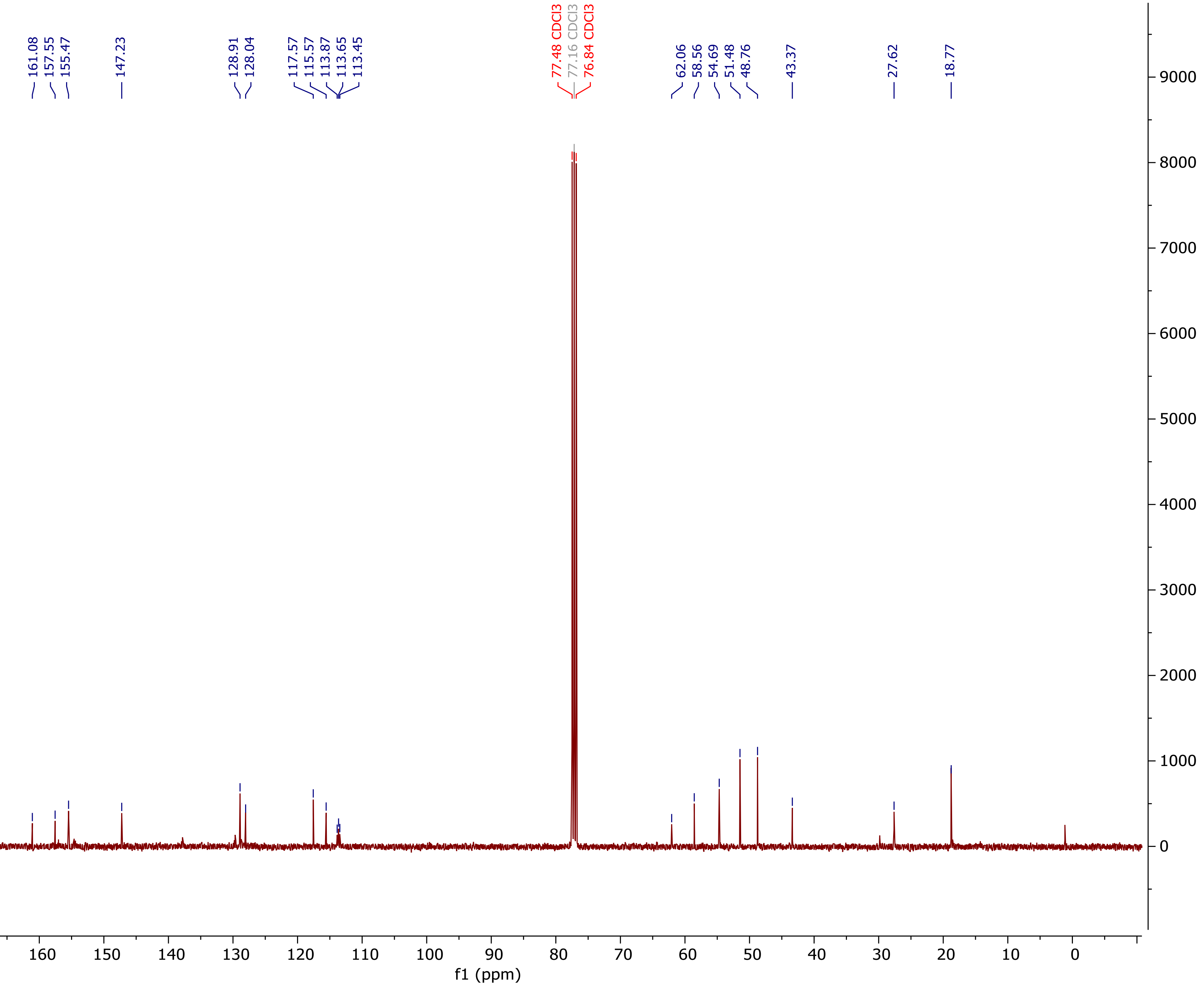

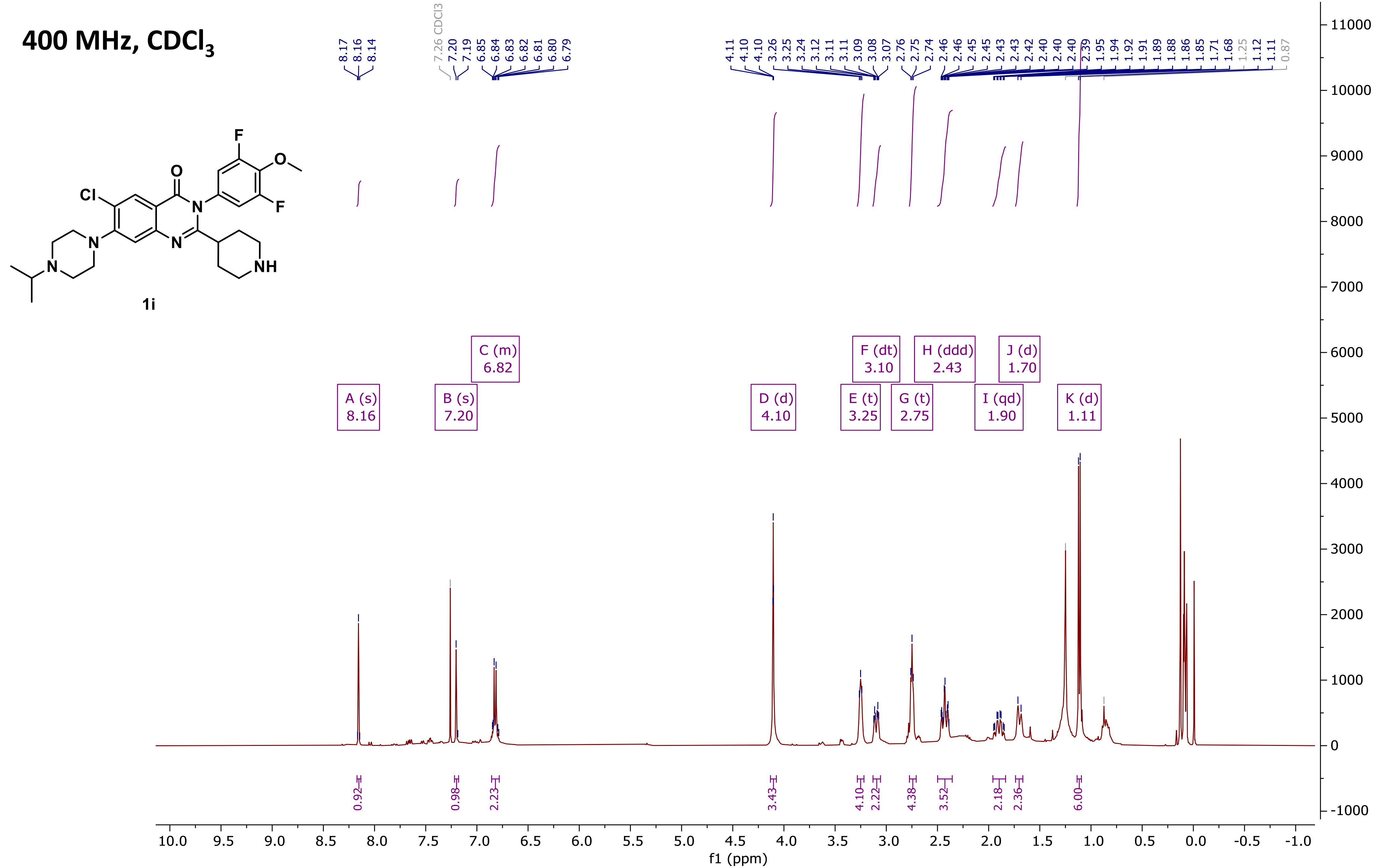
400 MHz, CDCl3
1i
A (s) 8.16
B (s) 7.20
C (m) 6.82
D (d) 4.10
E (t) 3.25
F (dt) 3.10
G (t) 2.75
H (ddd) 2.43
I (qd) 1.90
J (d) 1.70
K (d) 1.11
f1 (ppm)

101 MHz, $CDCl_3$

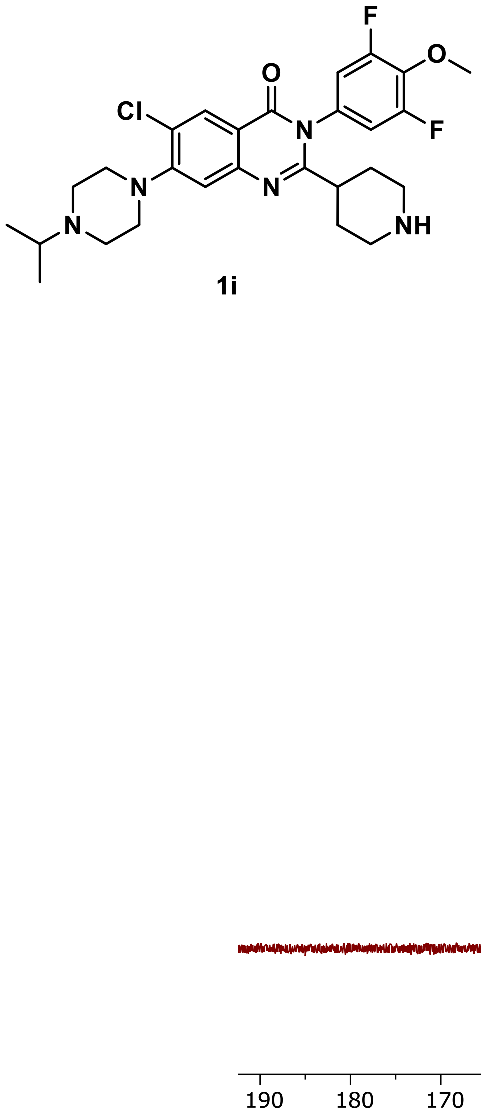

1i

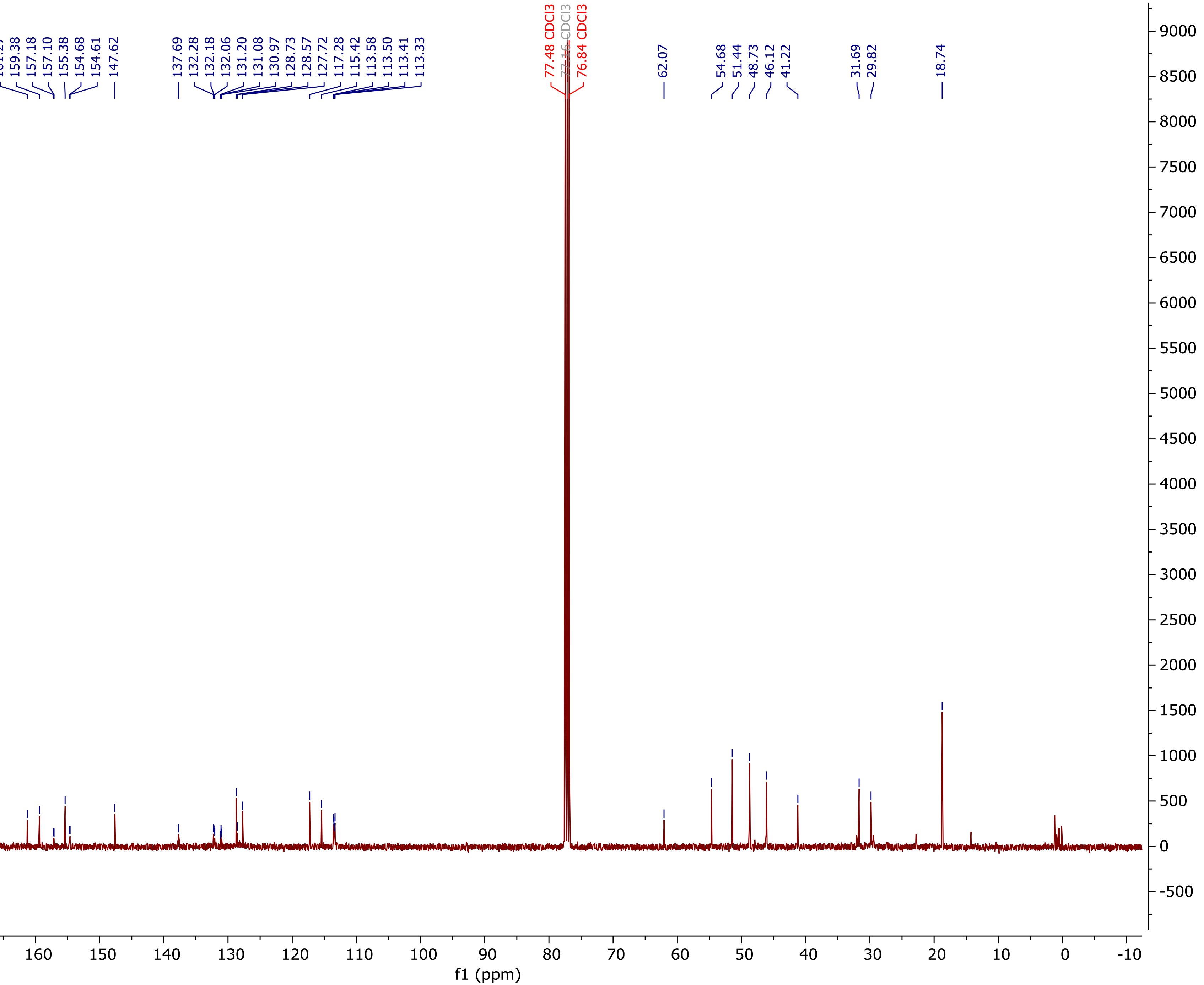

400 MHz, $CDCl_3$

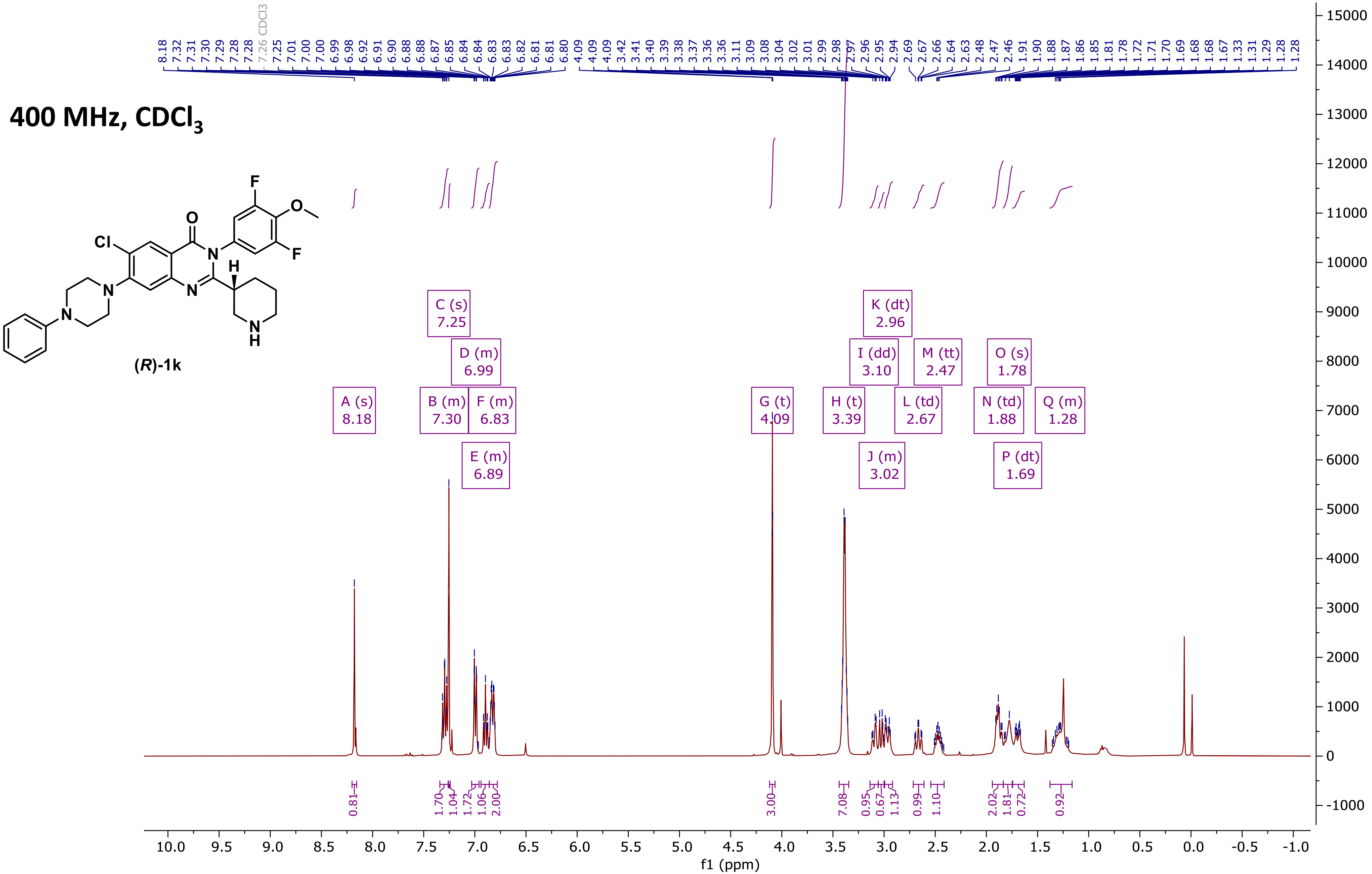

(*R*)-1k

101 MHz, $CDCl_3$

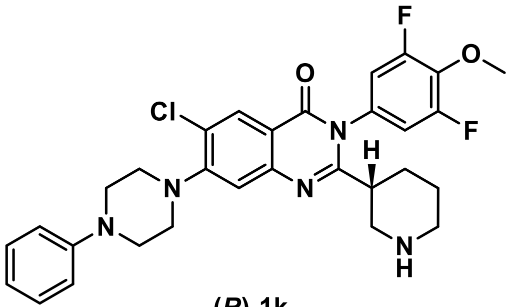

(*R*)-1k

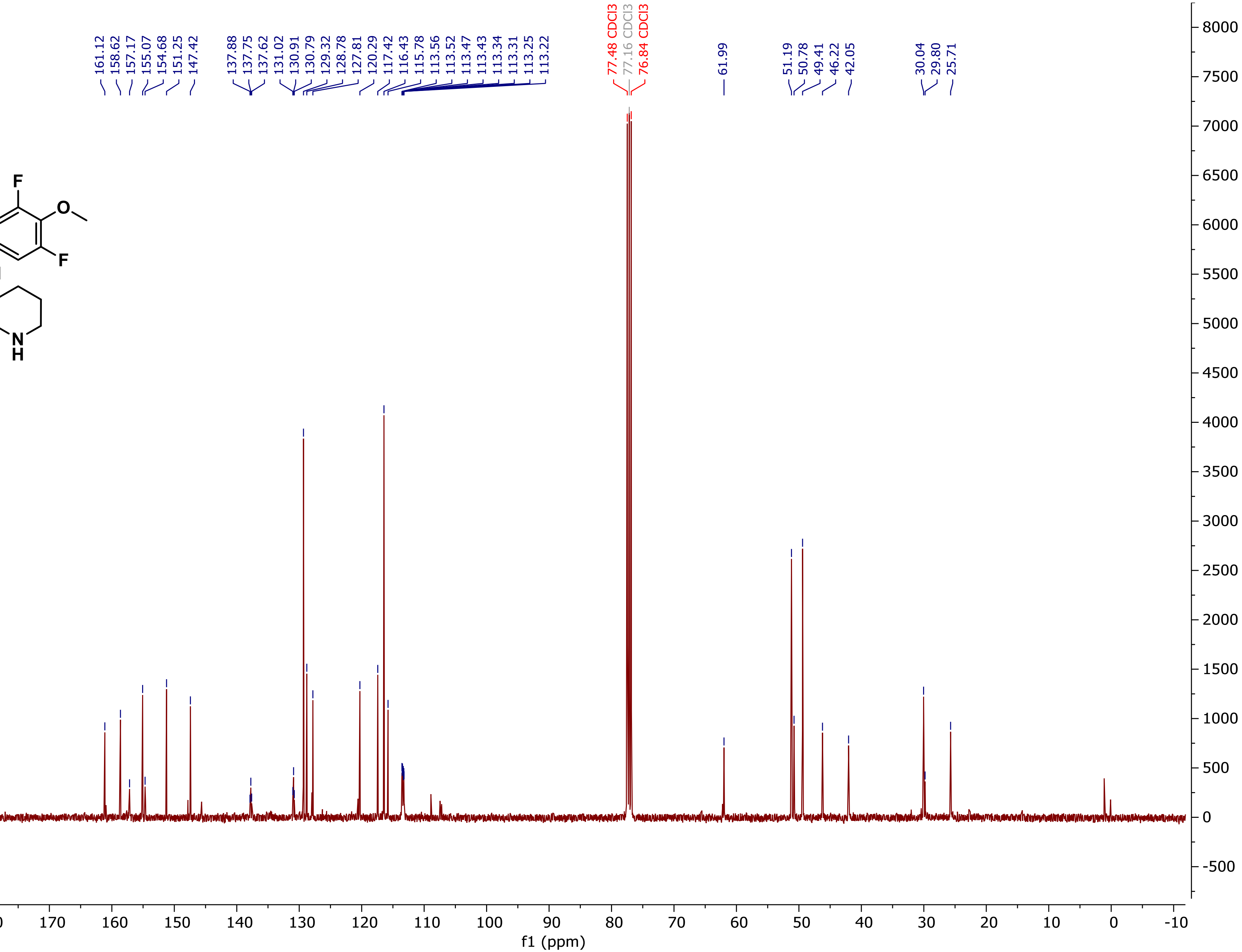

400 MHz, $CDCl_3$

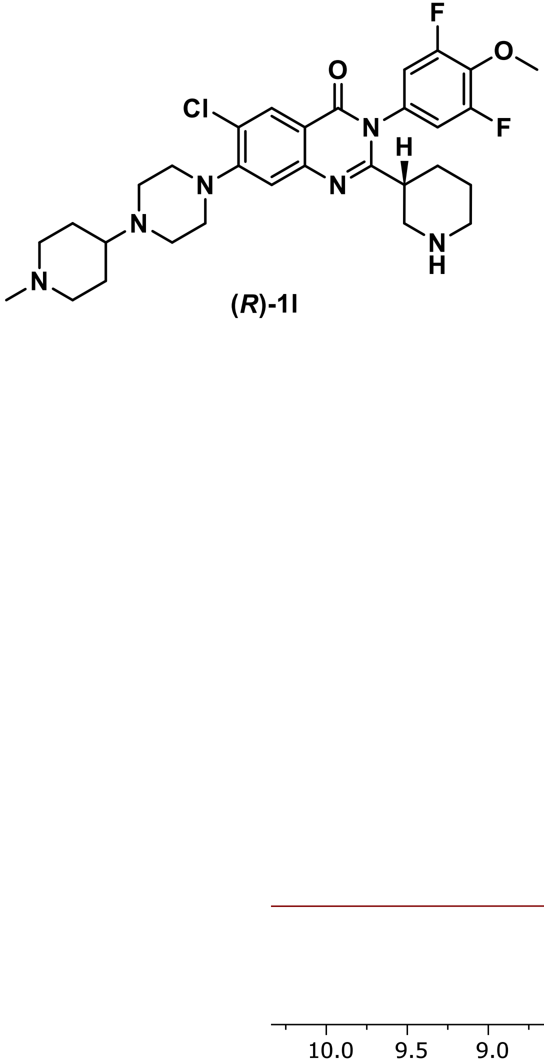

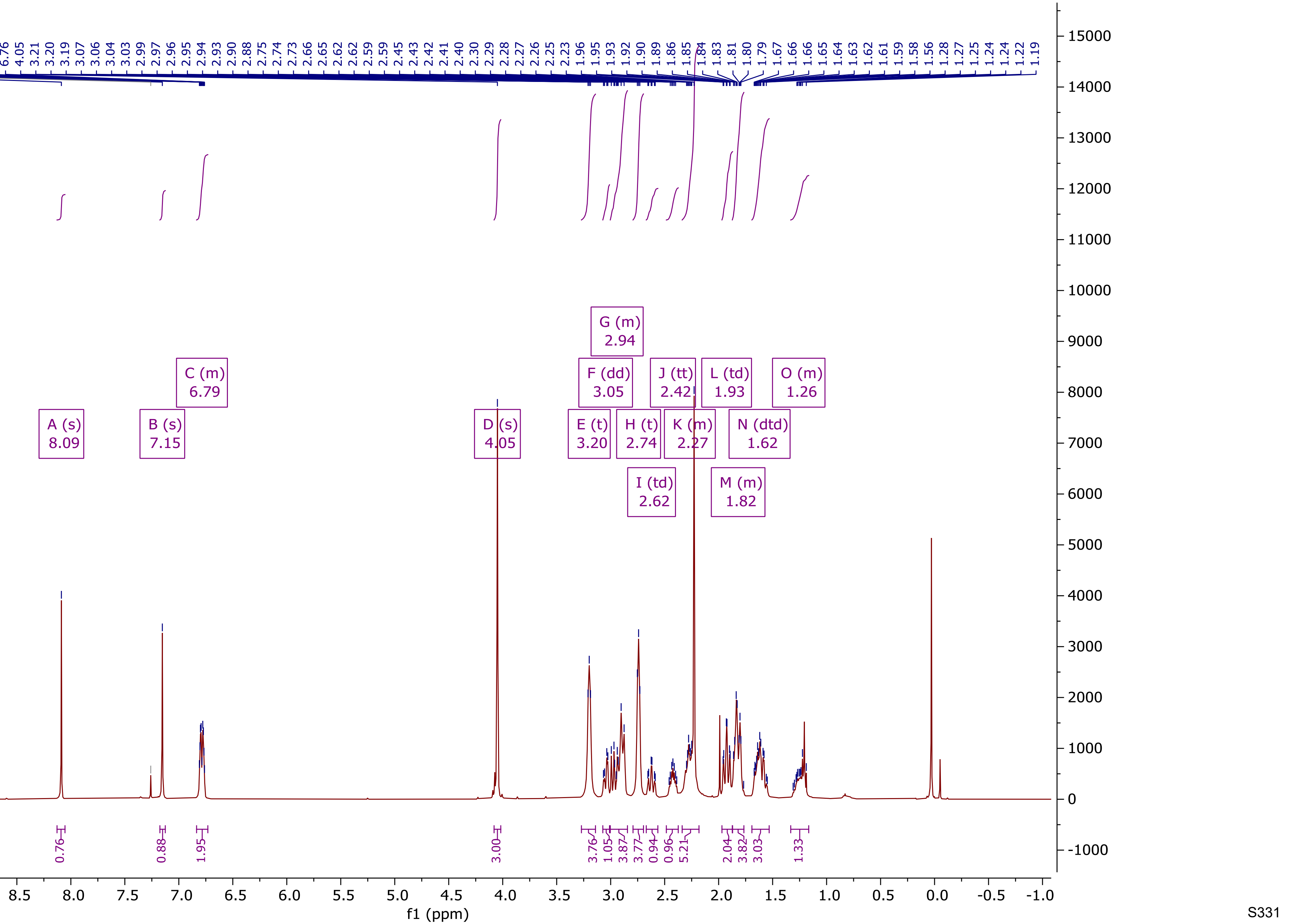

101 MHz, $CDCl_3$

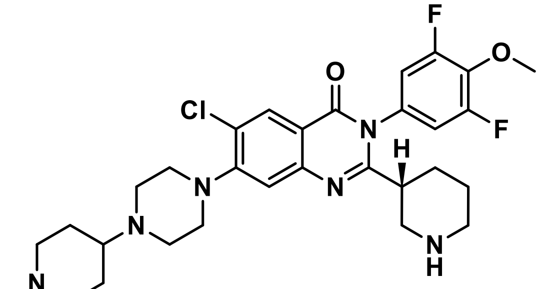

(*R*)-1l

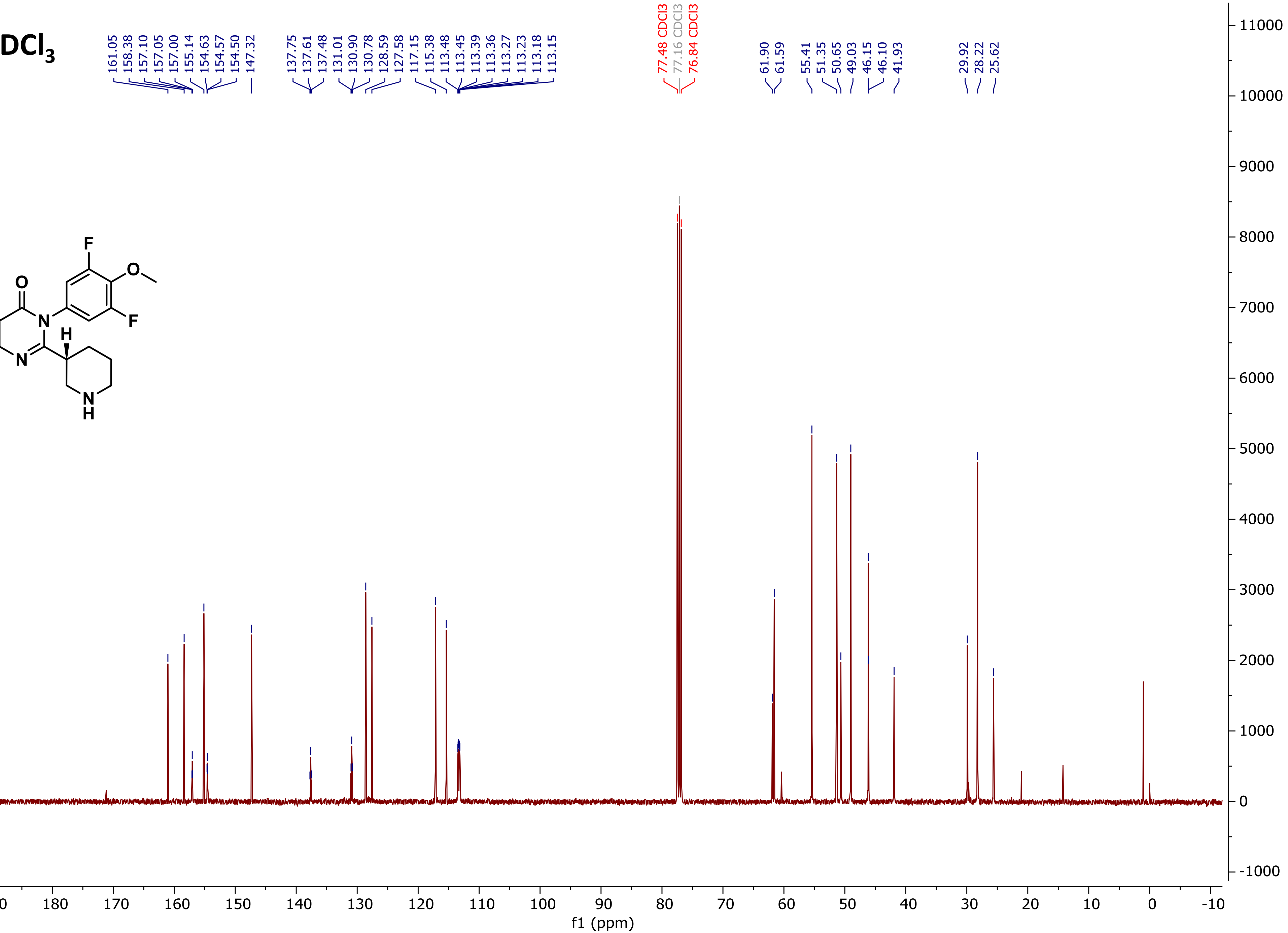

400 MHz, $CDCl_3$

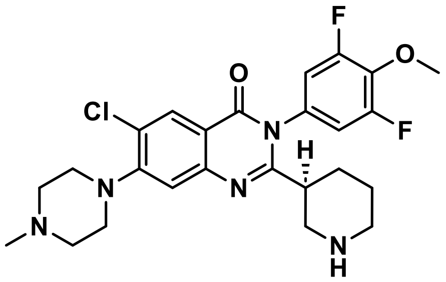

(*S*)-1m

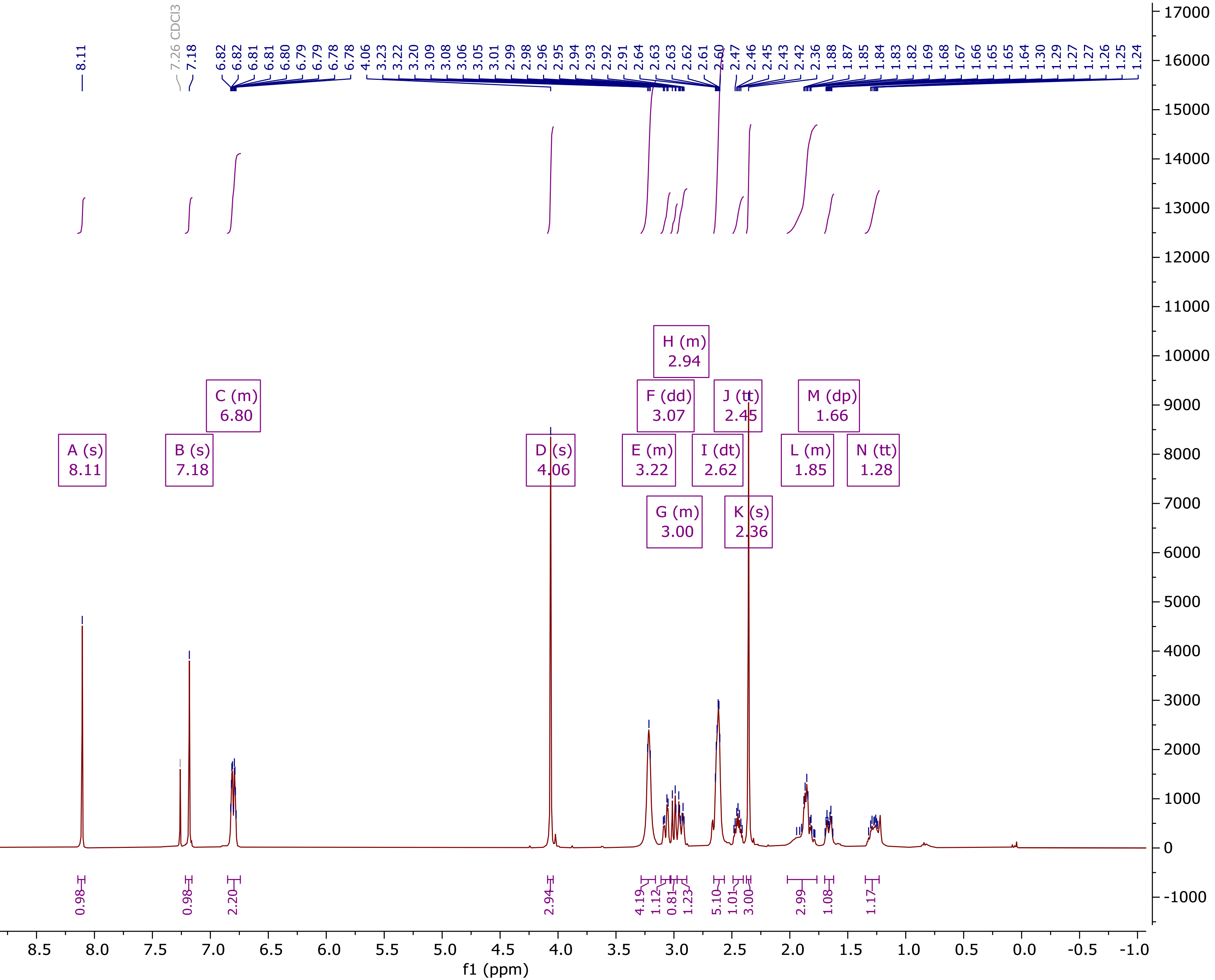

101 MHz, $CDCl_3$

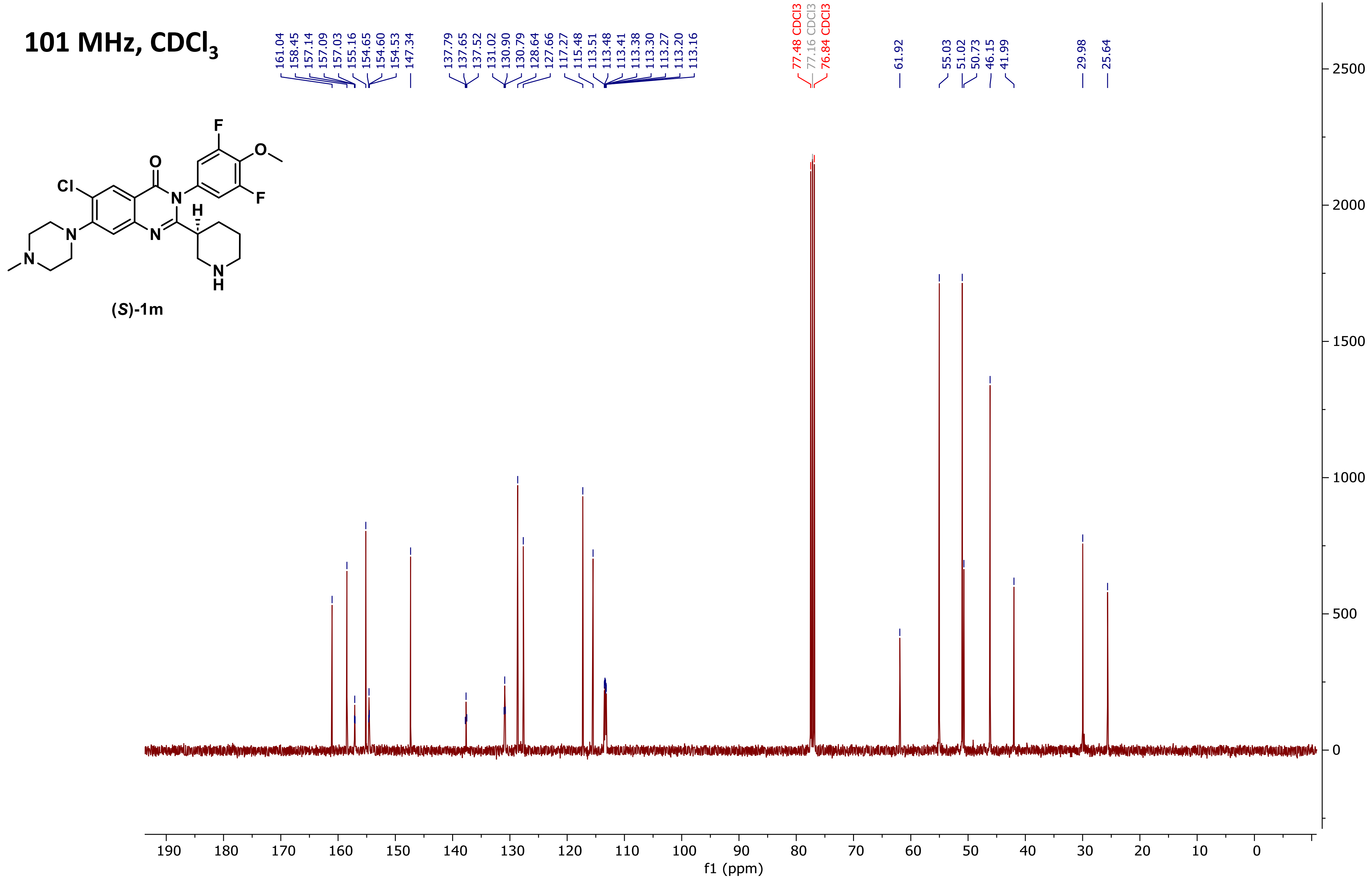

(*S*)-1m

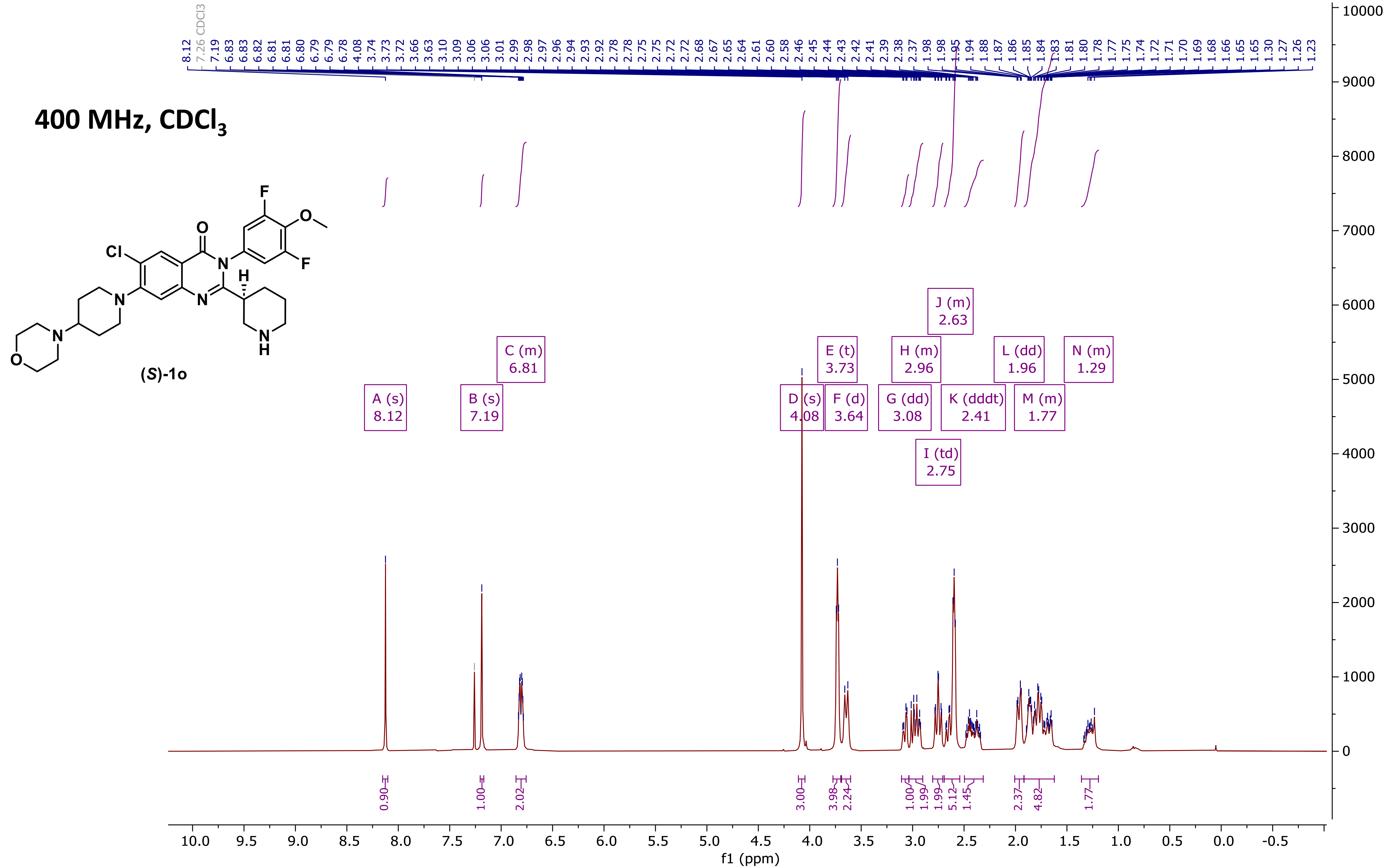

400 MHz, CDCl3
(S)-1o
A (s) 8.12
B (s) 7.19
C (m) 6.81
D (s) 4.08
E (t) 3.73
F (d) 3.64
G (dd) 3.08
H (m) 2.96
I (td) 2.75
J (m) 2.63
K (dddt) 2.41
L (dd) 1.96
M (m) 1.77
N (m) 1.29
f1 (ppm)

101 MHz, $CDCl_3$

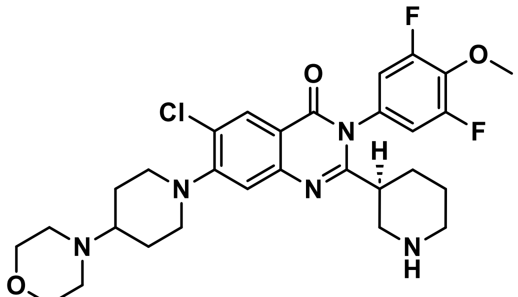

(*S*)-1o

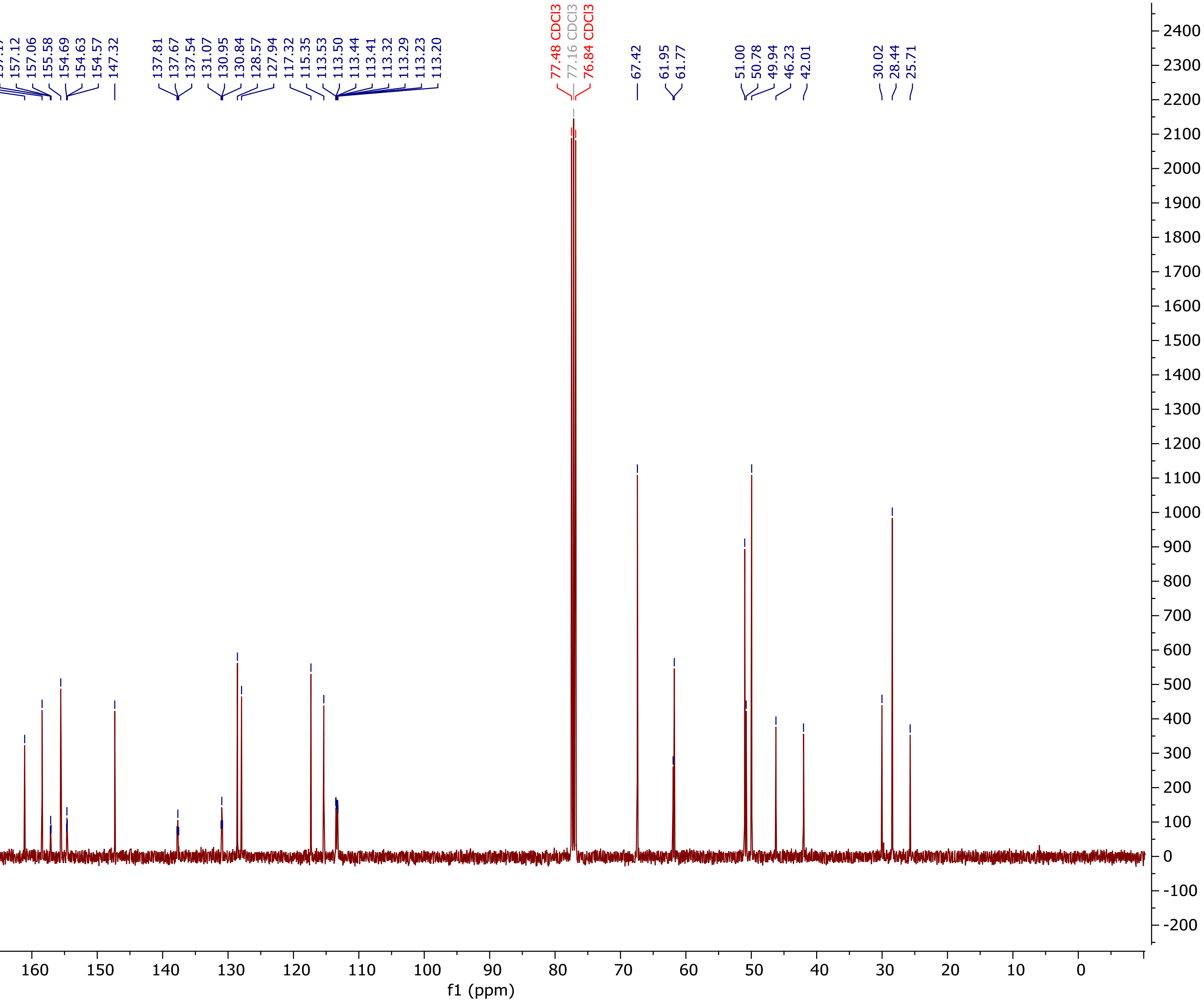

400 MHz, $CDCl_3$

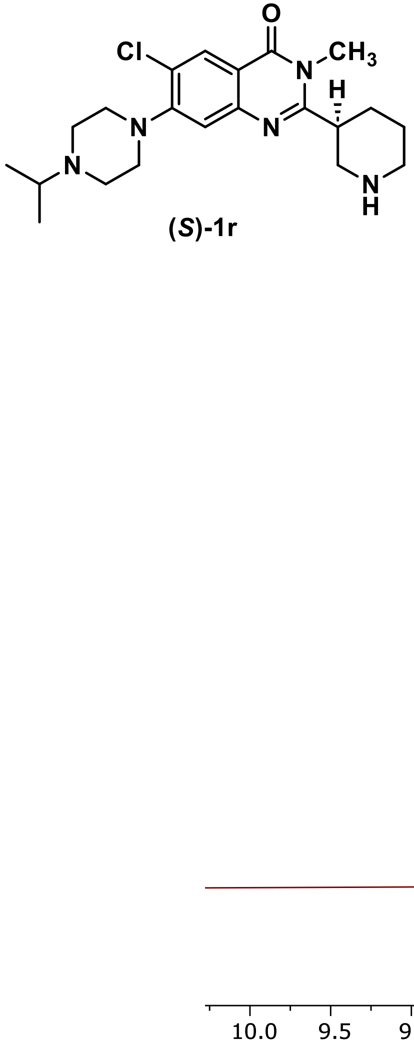

(*S*)-1r

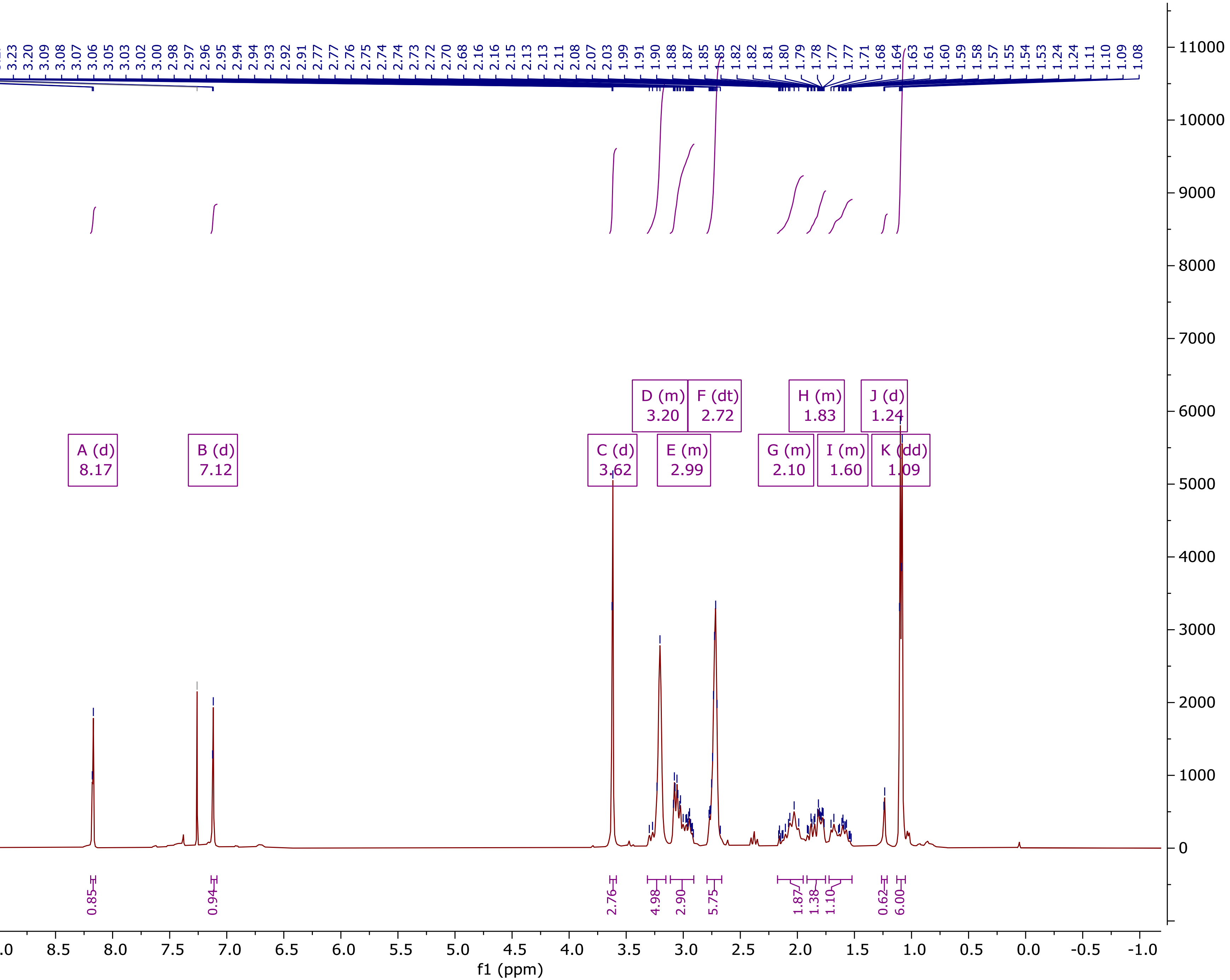

101 MHz, $CDCl_3$

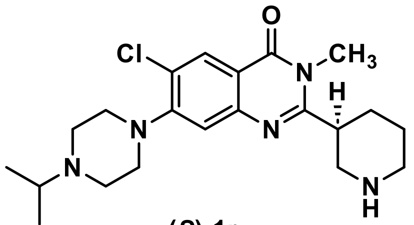

(*S*)-1r

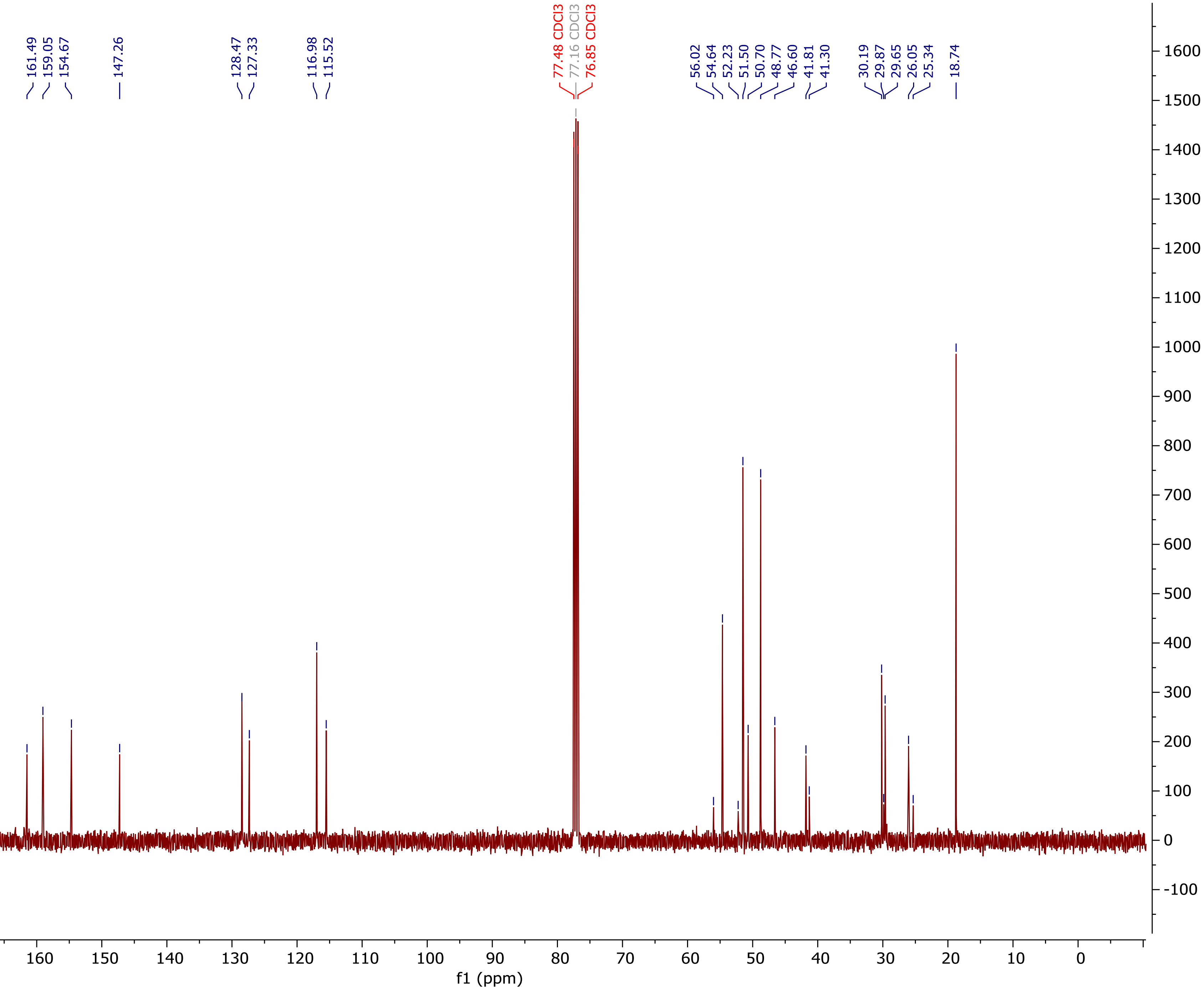

400 MHz, $CDCl_3$

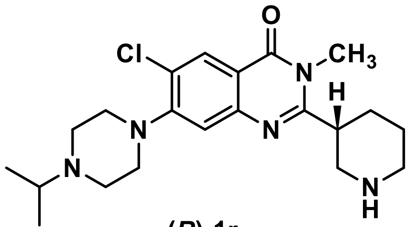

(*R*)-1r

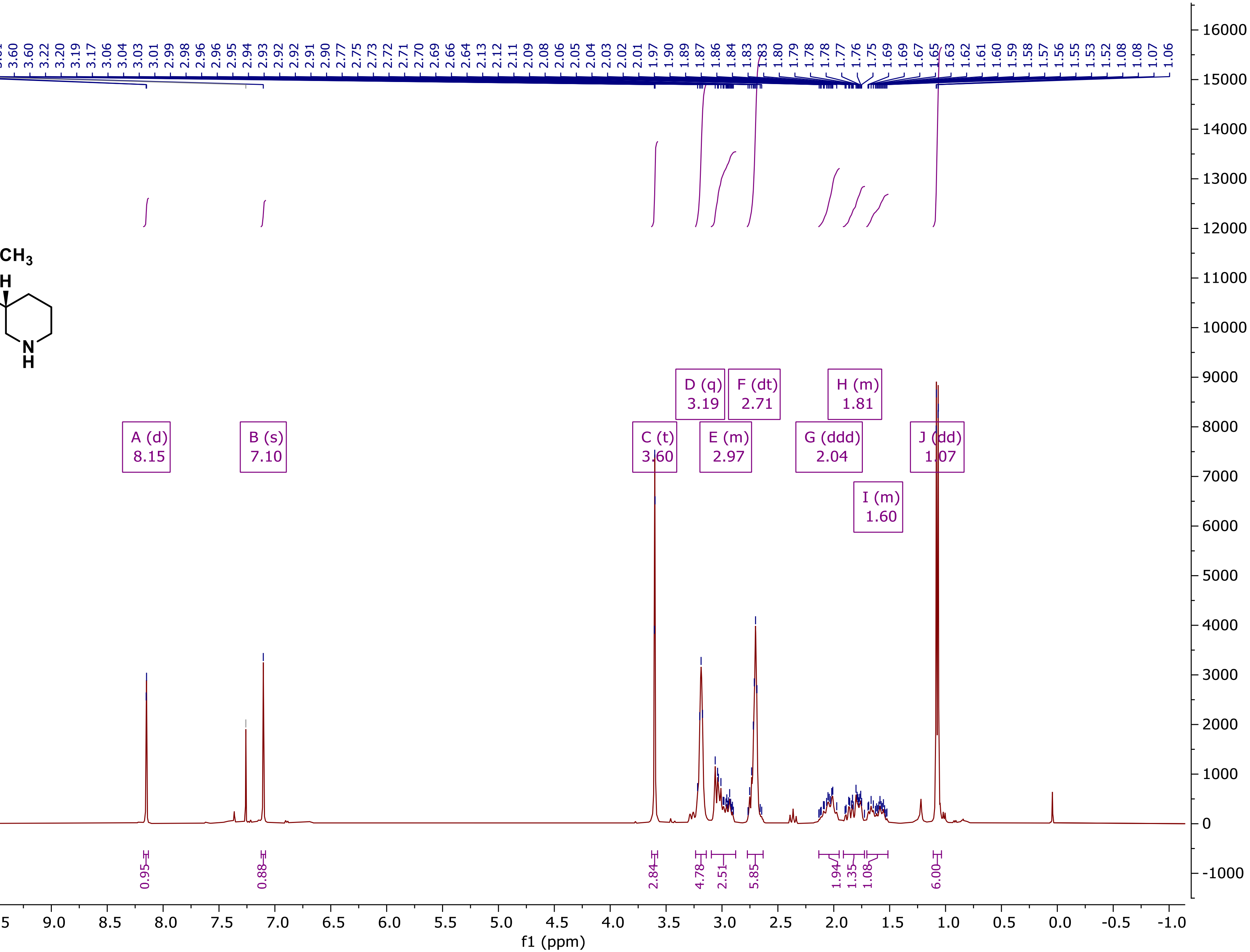

101 MHz, $CDCl_3$

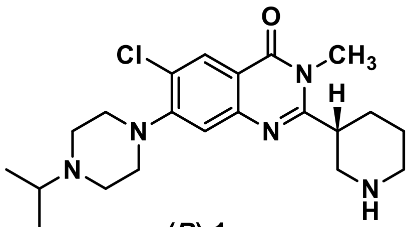

(*R*)-1r

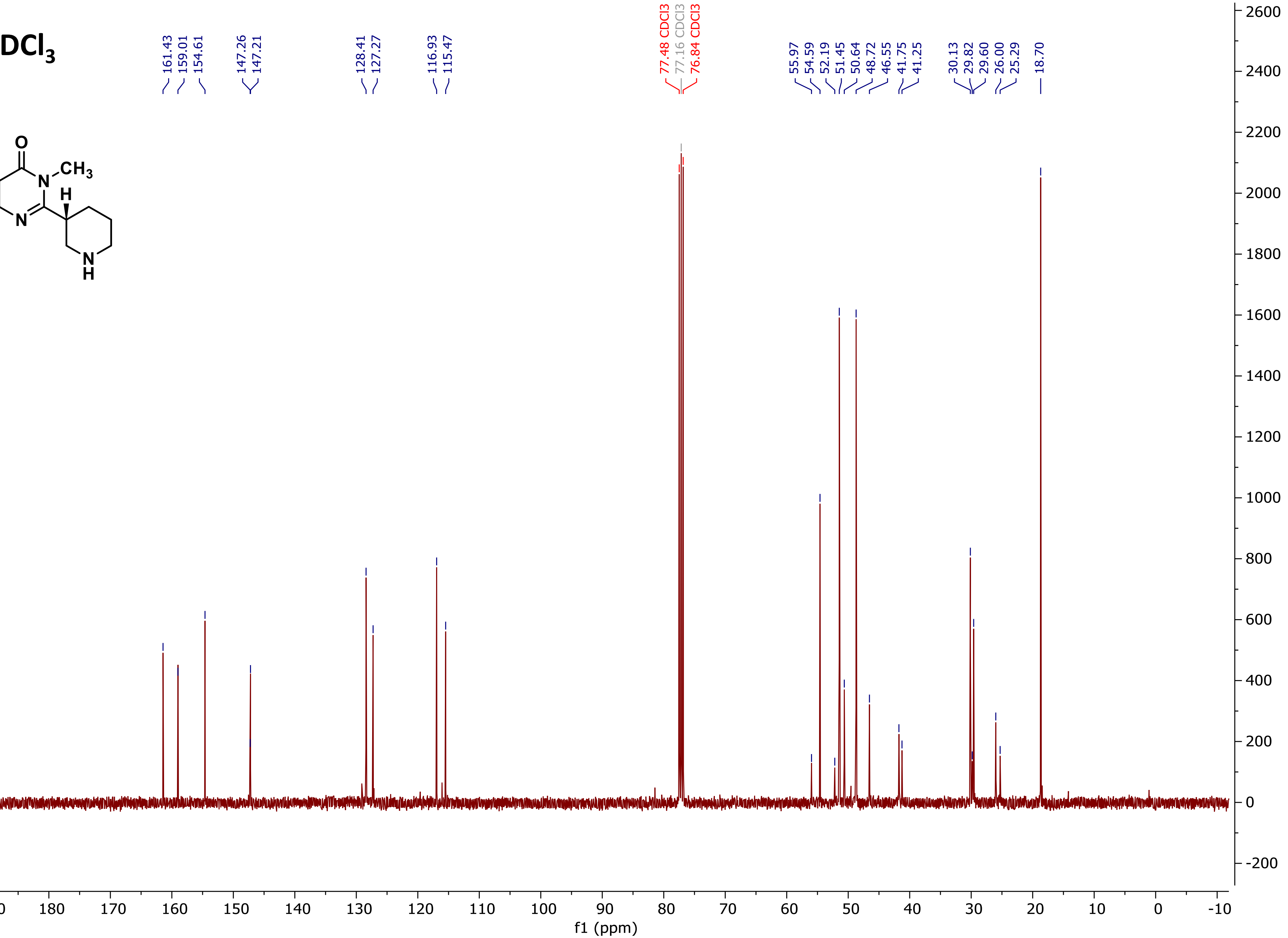

400 MHz, $CDCl_3$

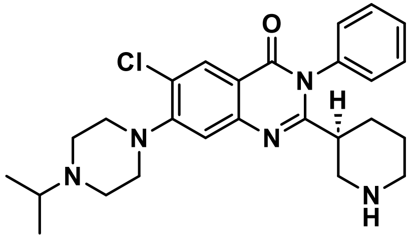

(*S*)-1s

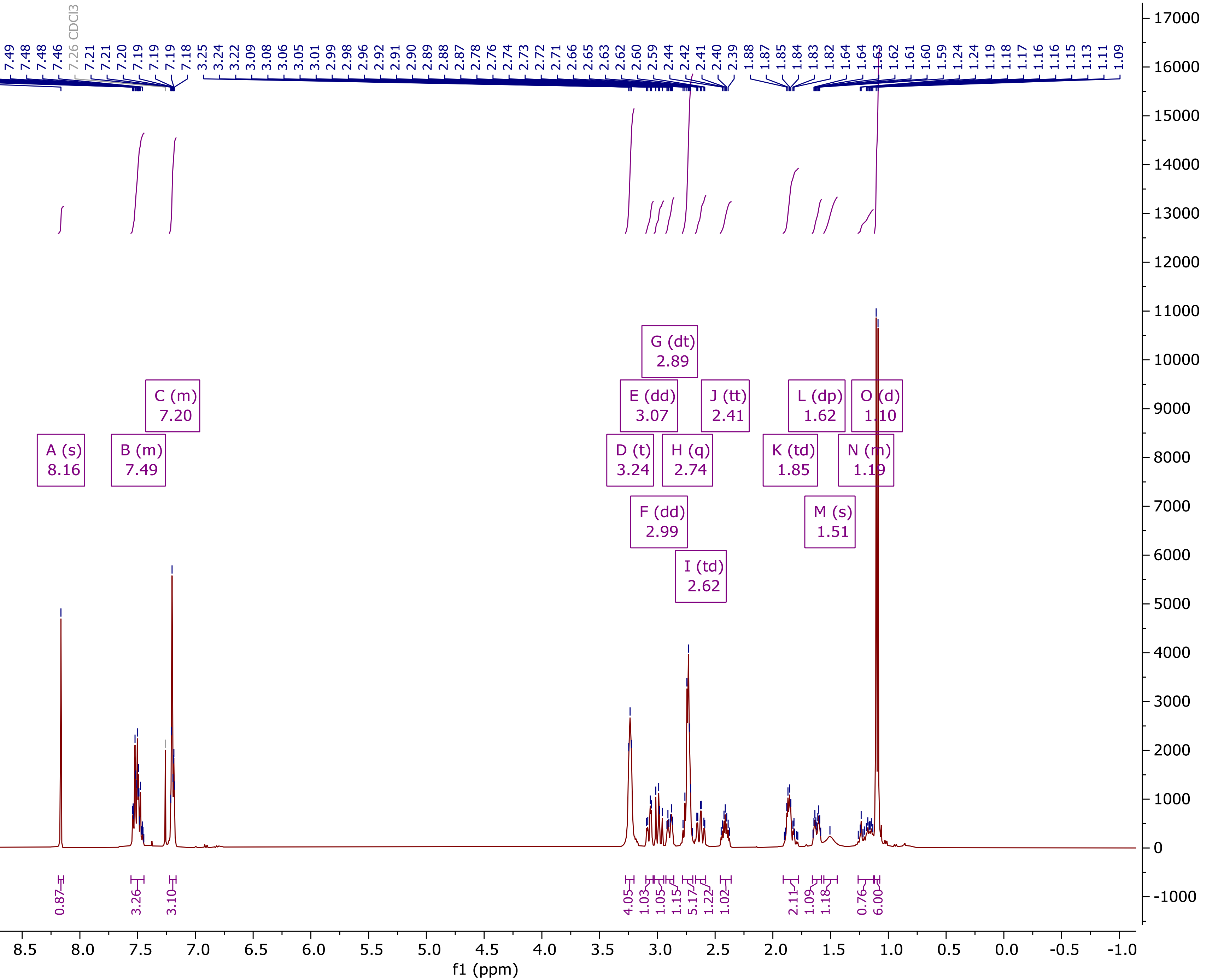

101 MHz, $CDCl_3$

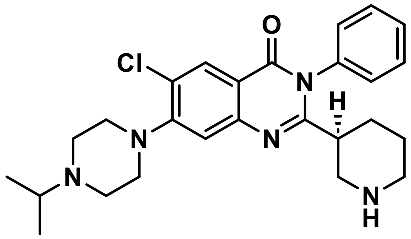

(*S*)-1s

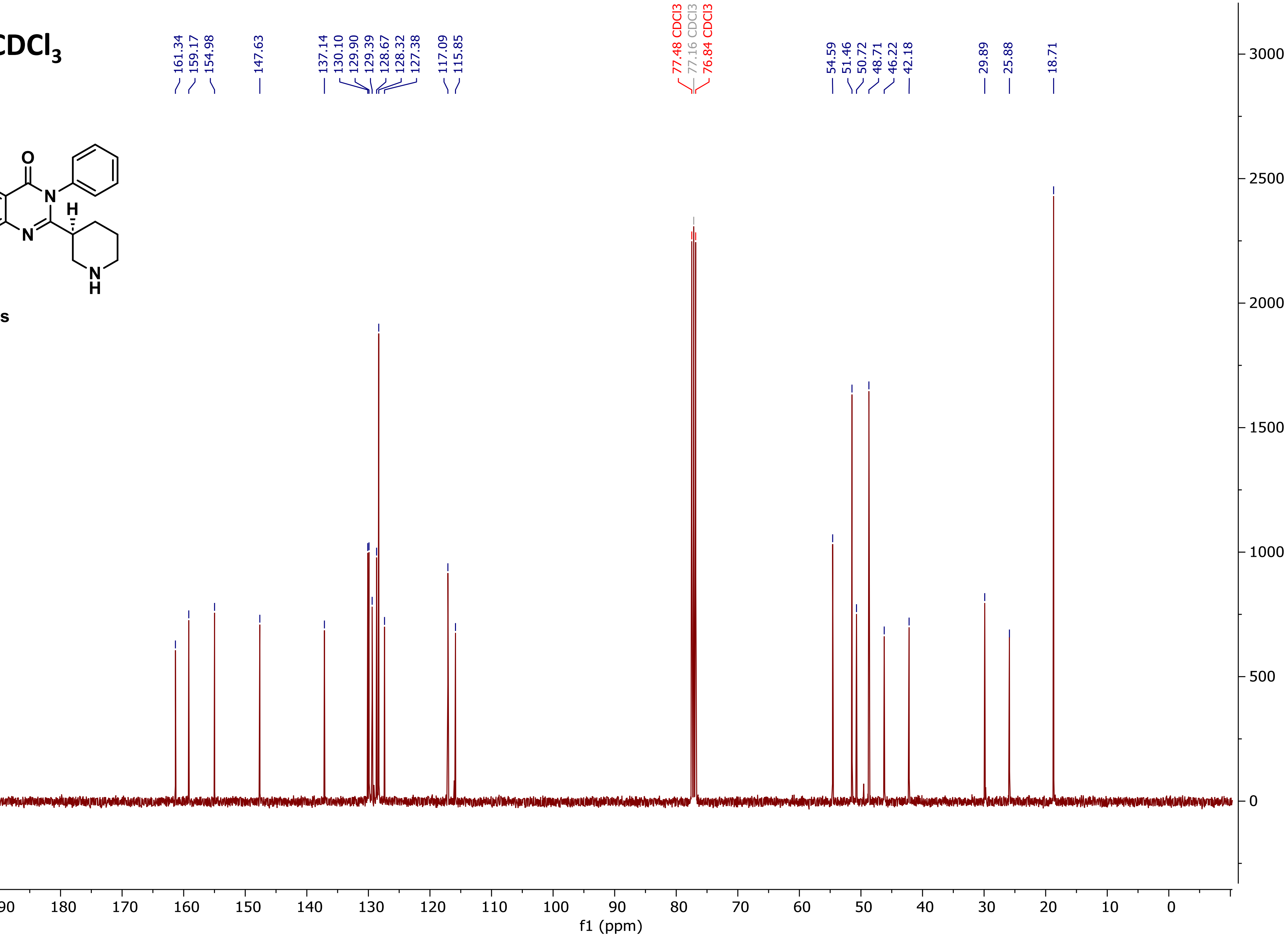

400 MHz, $CDCl_3$

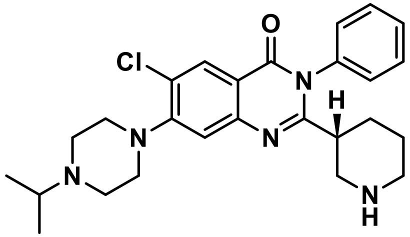

(*R*)-1s

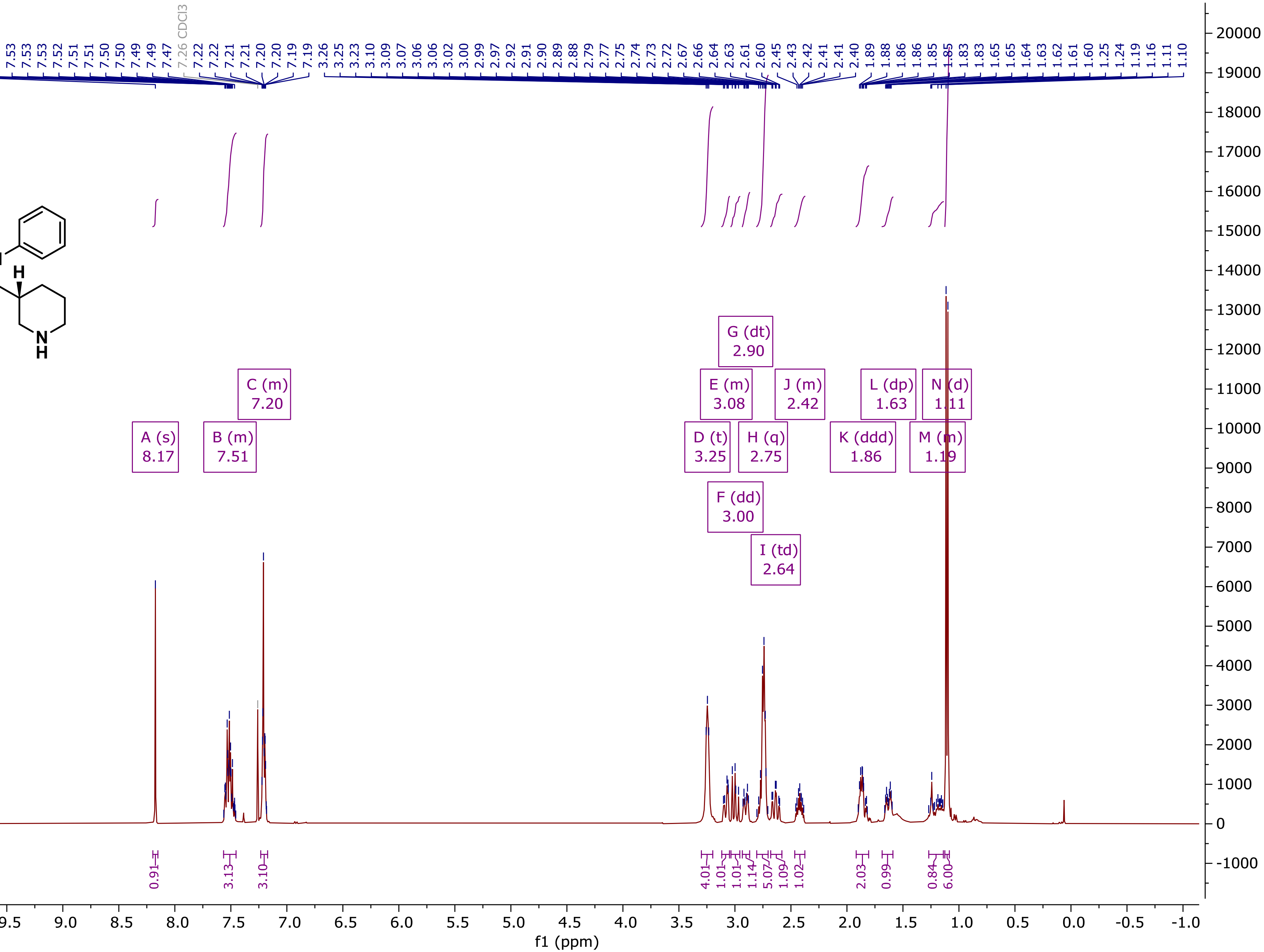

101 MHz, $CDCl_3$

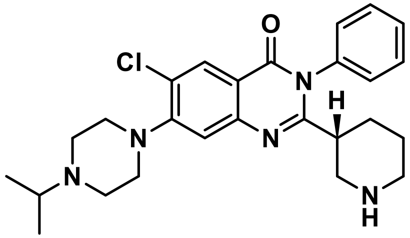

(*R*)-1s

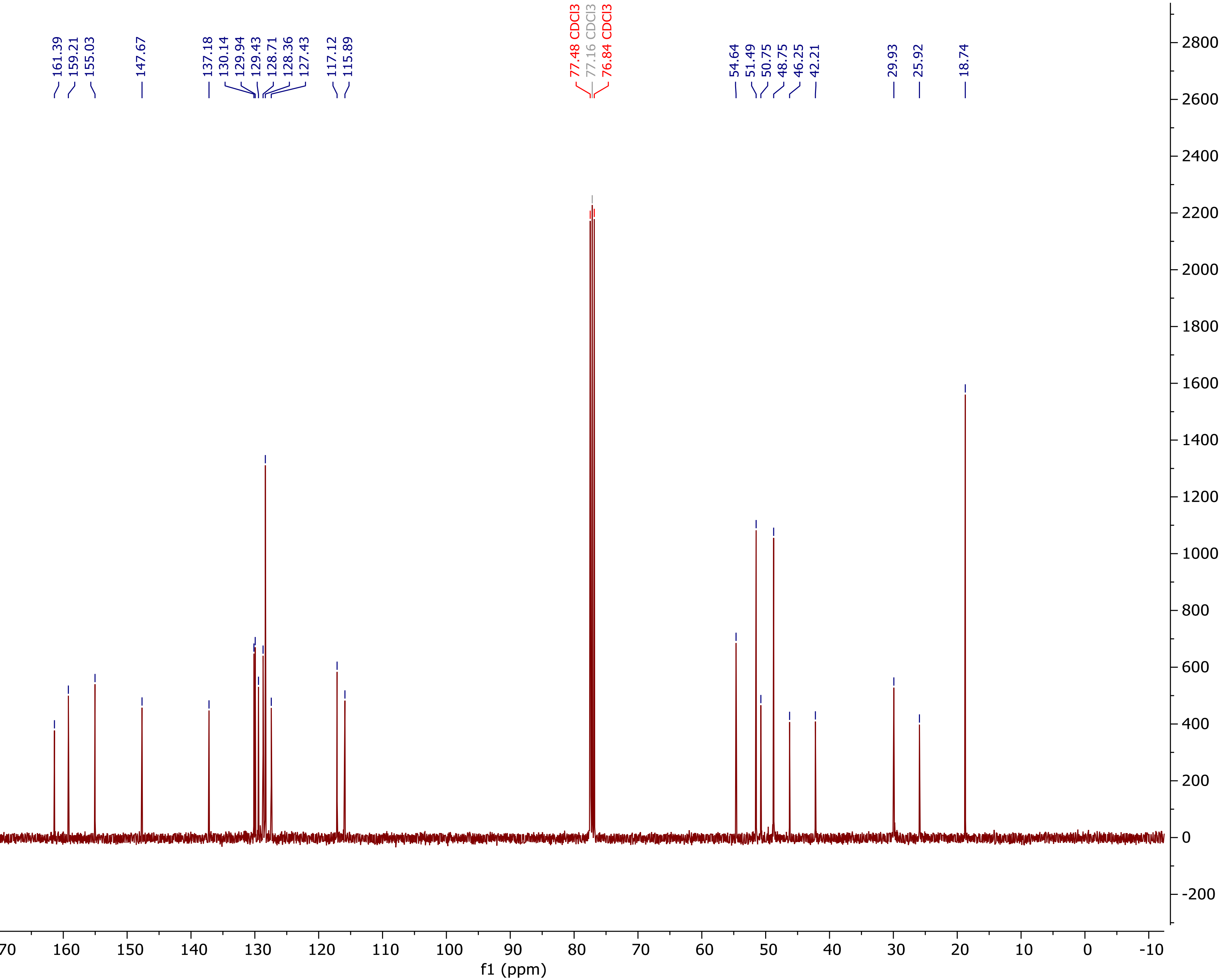

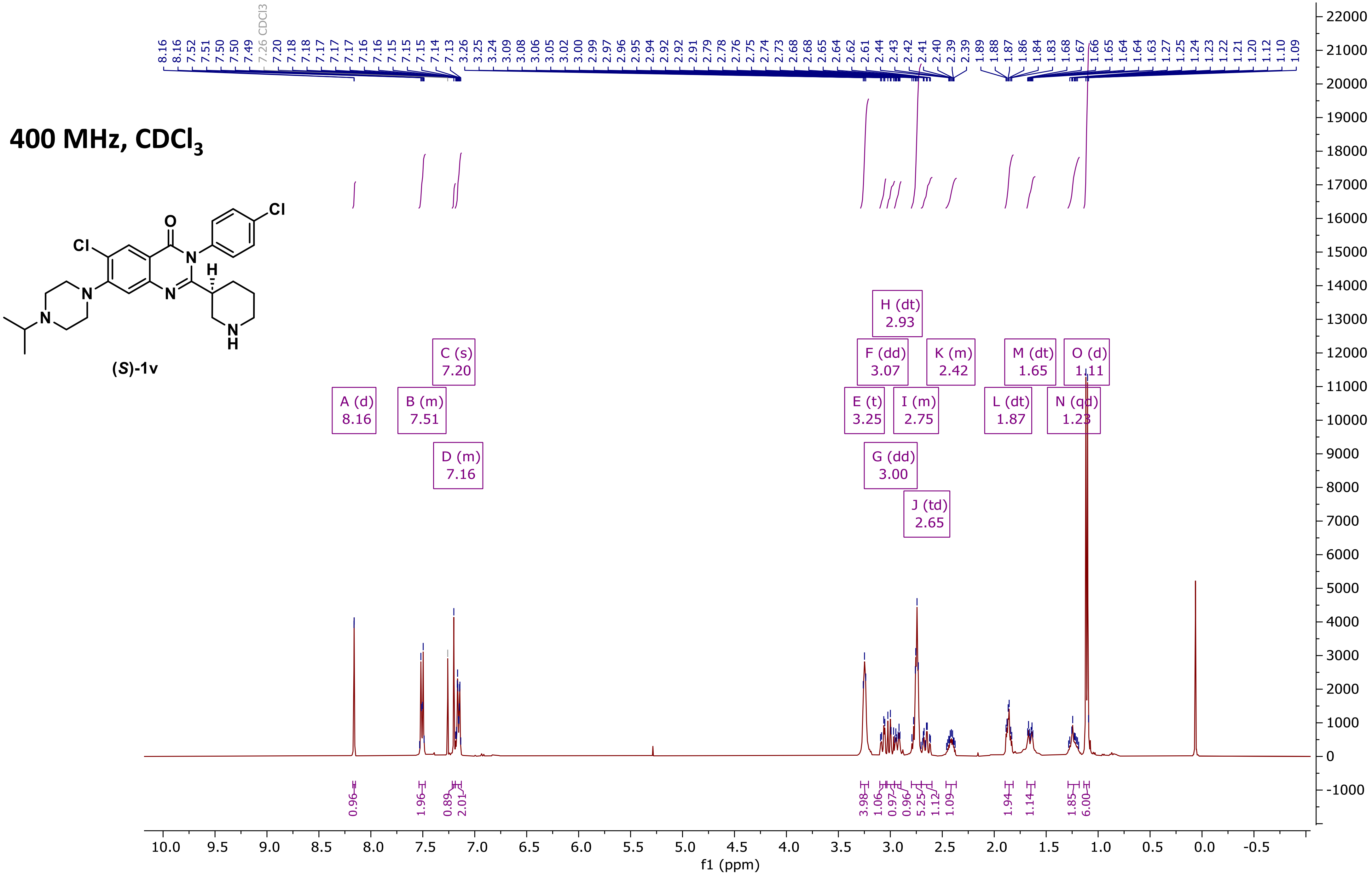

400 MHz, CDCl3
(S)-1v
A (d) 8.16
B (m) 7.51
C (s) 7.20
D (m) 7.16
E (t) 3.25
F (dd) 3.07
G (dd) 3.00
H (dt) 2.93
I (m) 2.75
J (td) 2.65
K (m) 2.42
L (dt) 1.87
M (dt) 1.65
N (qd) 1.23
O (d) 1.11
f1 (ppm)

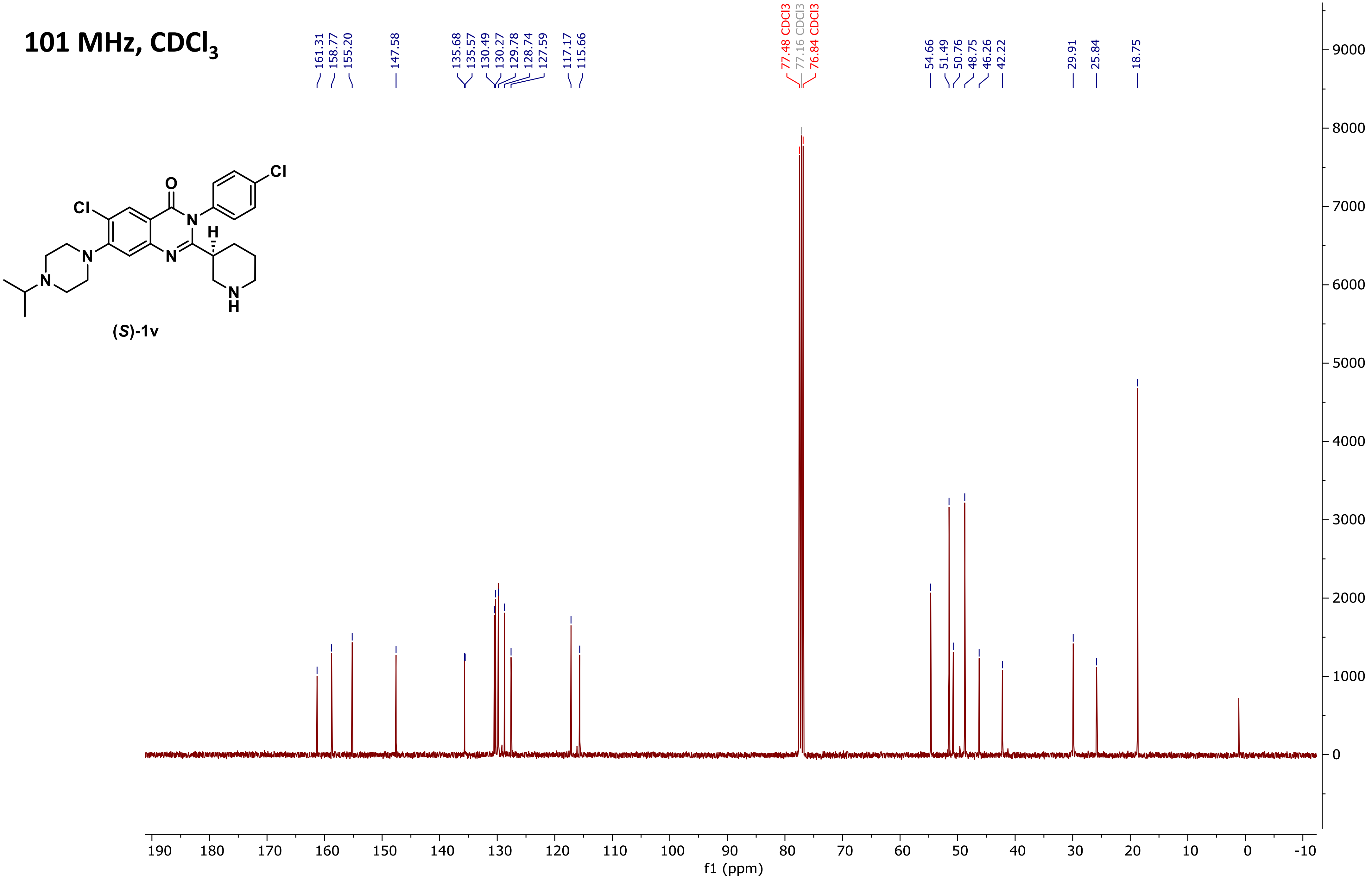
101 MHz, CDCl3
161.31
158.77
155.20
147.58
135.68
135.57
130.49
130.27
129.78
128.74
127.59
117.17
115.66
77.48 CDCl3
77.16 CDCl3
76.84 CDCl3
54.66
51.49
50.76
48.75
46.26
42.22
29.91
25.84
18.75
(S)-1v
f1 (ppm)

400 MHz, $CDCl_3$

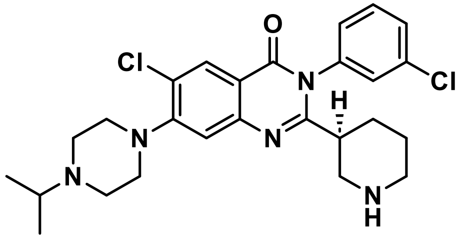

(*S*)-1w

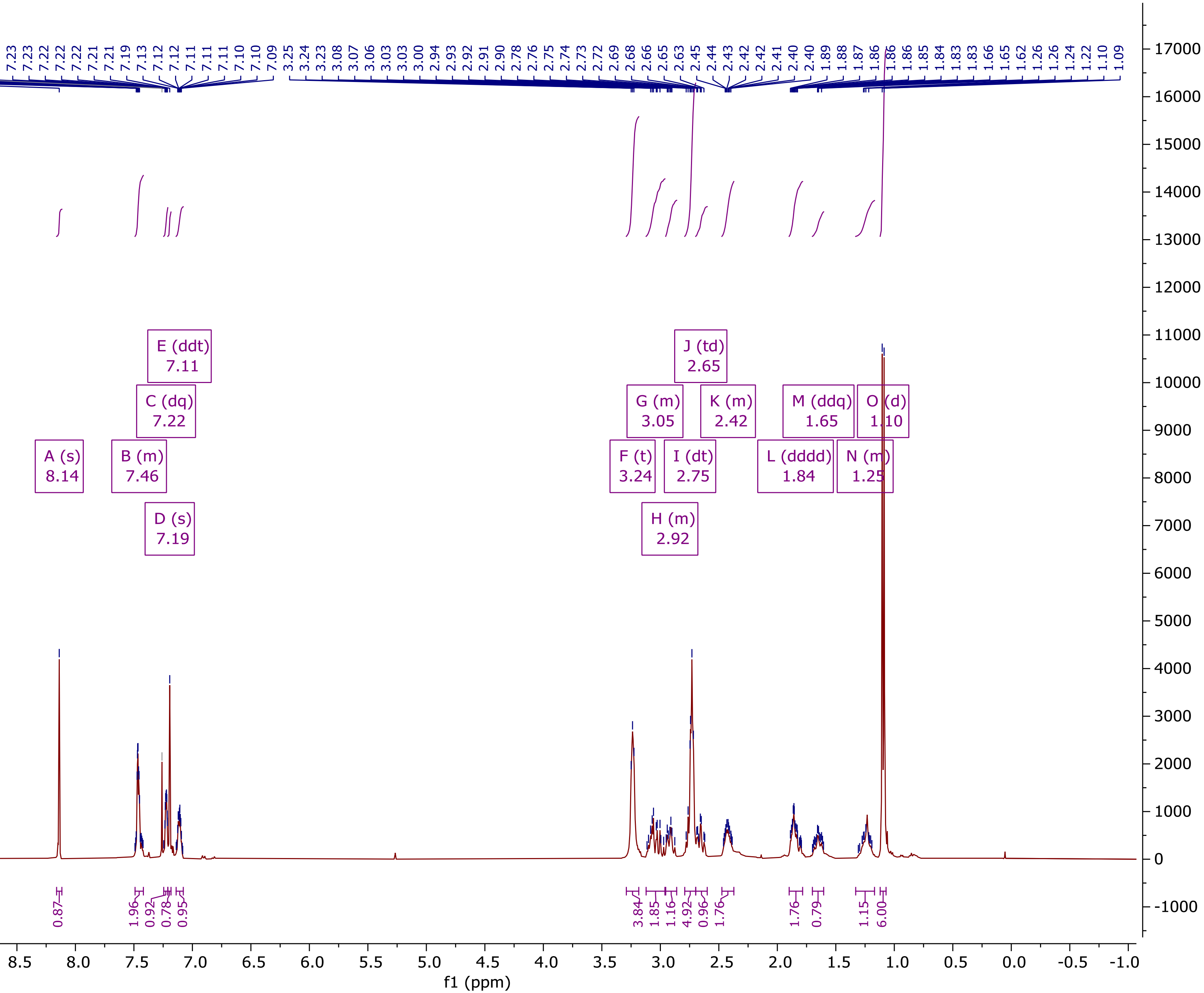

101 MHz, $CDCl_3$

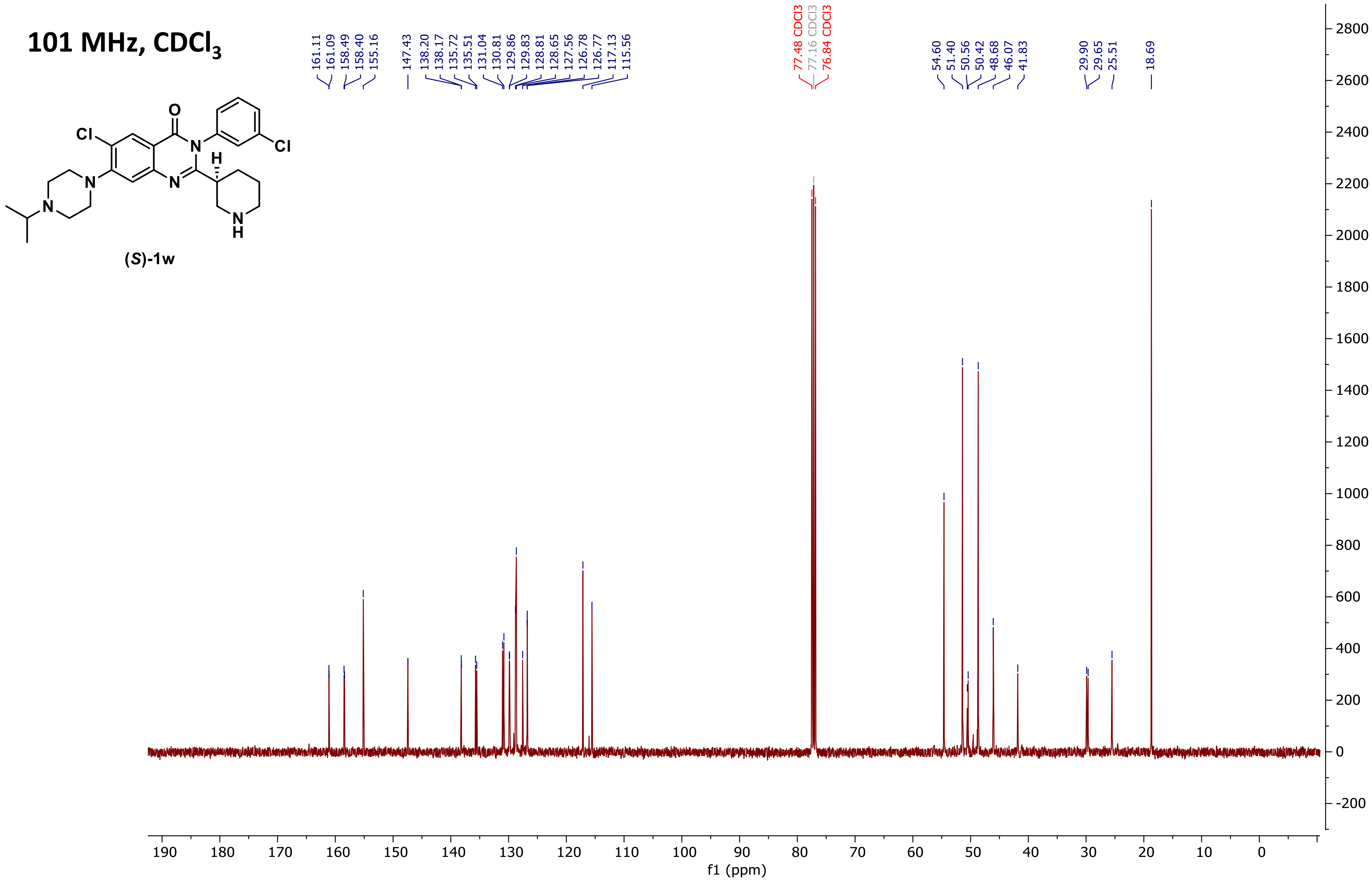

(*S*)-1w

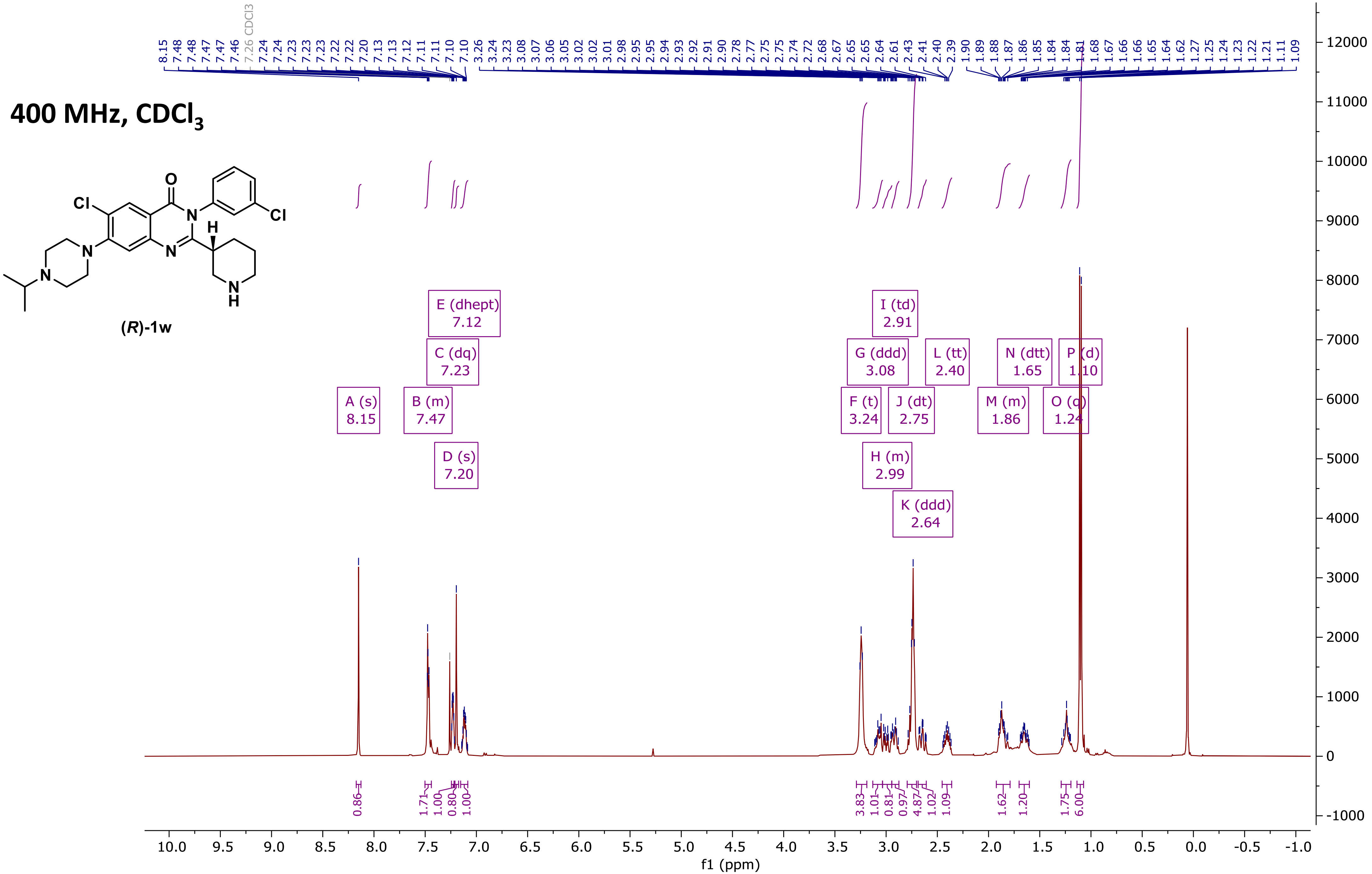
400 MHz, CDCl3
(R)-1w
8.15 7.48 7.48 7.47 7.47 7.46 7.26 CDCl3 7.24 7.24 7.23 7.23 7.23 7.22 7.22 7.20 7.13 7.13 7.12 7.11 7.11 7.10 7.10 3.26 3.24 3.23 3.08 3.07 3.06 3.05 3.02 3.02 3.01 2.98 2.95 2.95 2.94 2.93 2.92 2.91 2.90 2.78 2.77 2.75 2.75 2.74 2.72 2.68 2.67 2.65 2.65 2.64 2.61 2.43 2.41 2.40 2.39 1.90 1.89 1.88 1.87 1.86 1.85 1.84 1.84 1.81 1.68 1.67 1.66 1.66 1.65 1.64 1.62 1.27 1.25 1.24 1.23 1.22 1.21 1.11 1.09
A (s) 8.15
B (m) 7.47
C (dq) 7.23
D (s) 7.20
E (dhept) 7.12
F (t) 3.24
G (ddd) 3.08
H (m) 2.99
I (td) 2.91
J (dt) 2.75
K (ddd) 2.64
L (tt) 2.40
M (m) 1.86
N (dtt) 1.65
O (q) 1.24
P (d) 1.10
0.86 1.71 1.00 0.80 1.00 3.83 1.01 0.81 0.97 4.87 1.02 1.09 1.62 1.20 1.75 6.00
f1 (ppm)

101 MHz, $CDCl_3$

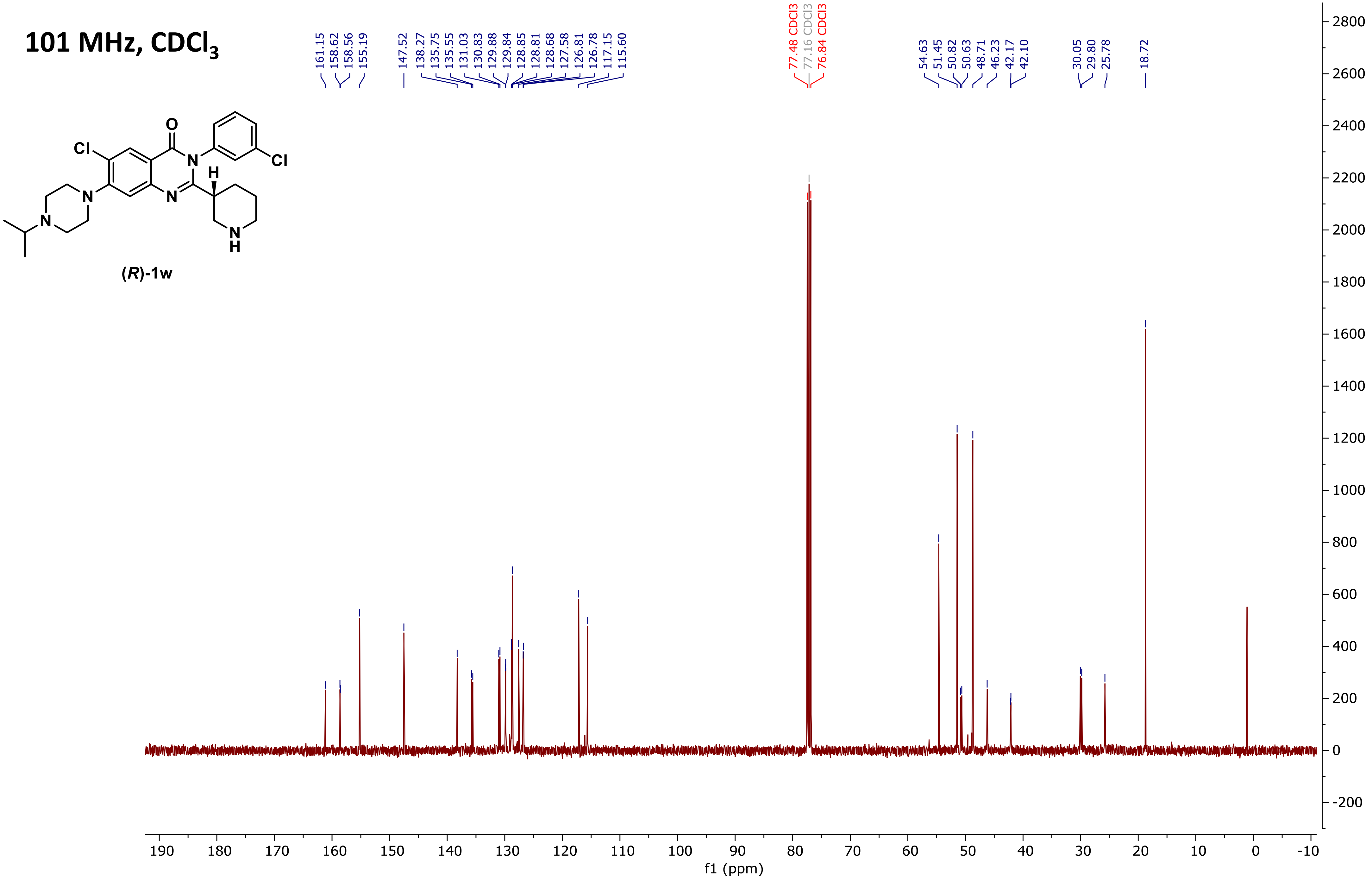

400 MHz, $CDCl_3$

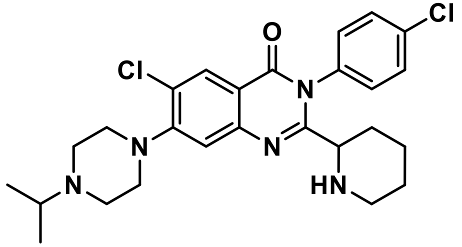

***rac*-1x**

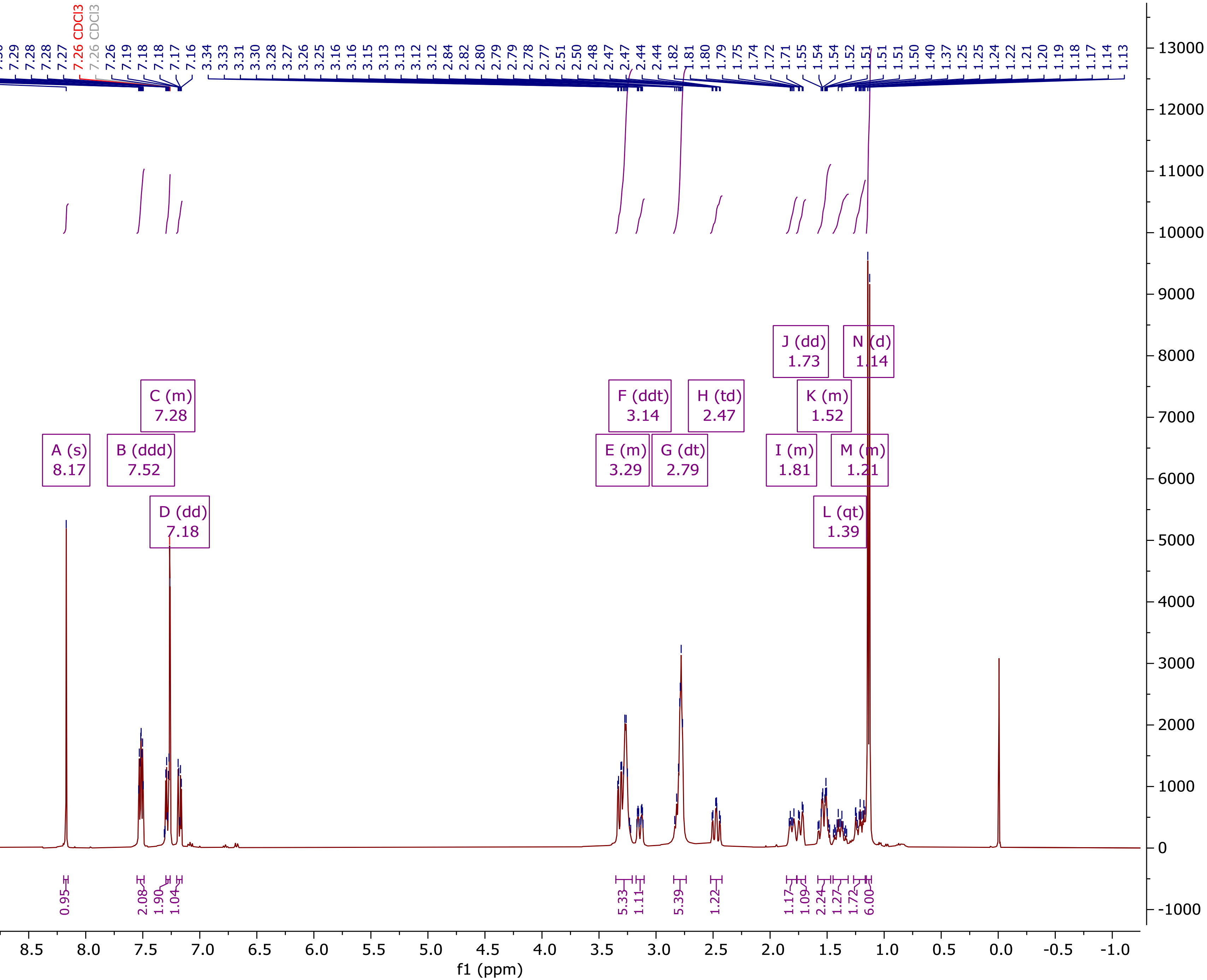

101 MHz, CDCl$_3$

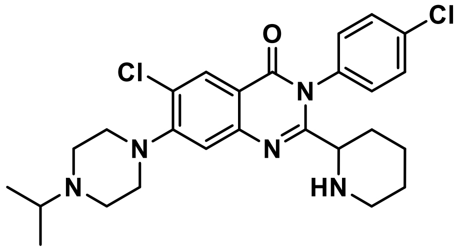

***rac*-1x**

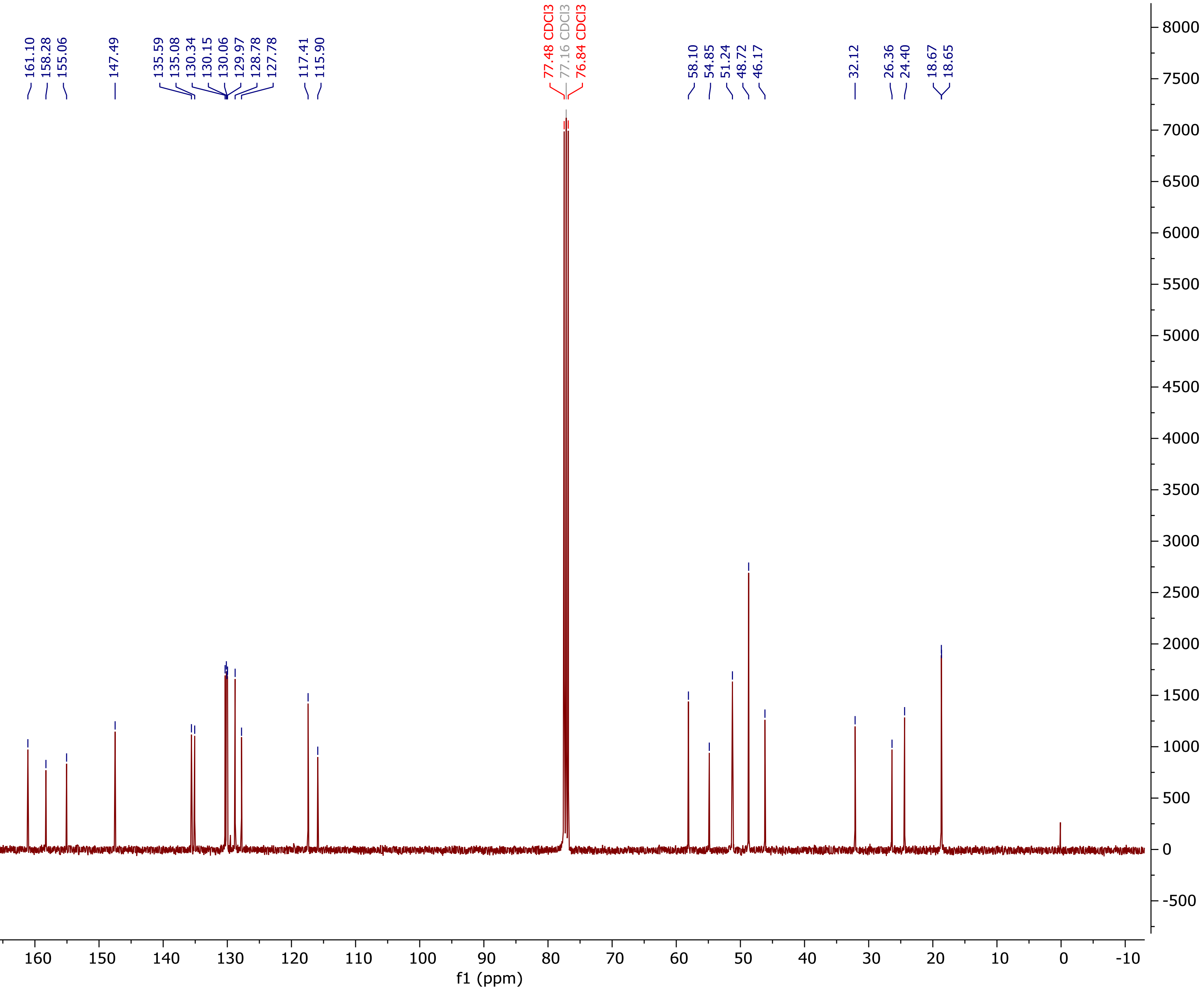

400 MHz, $CDCl_3$

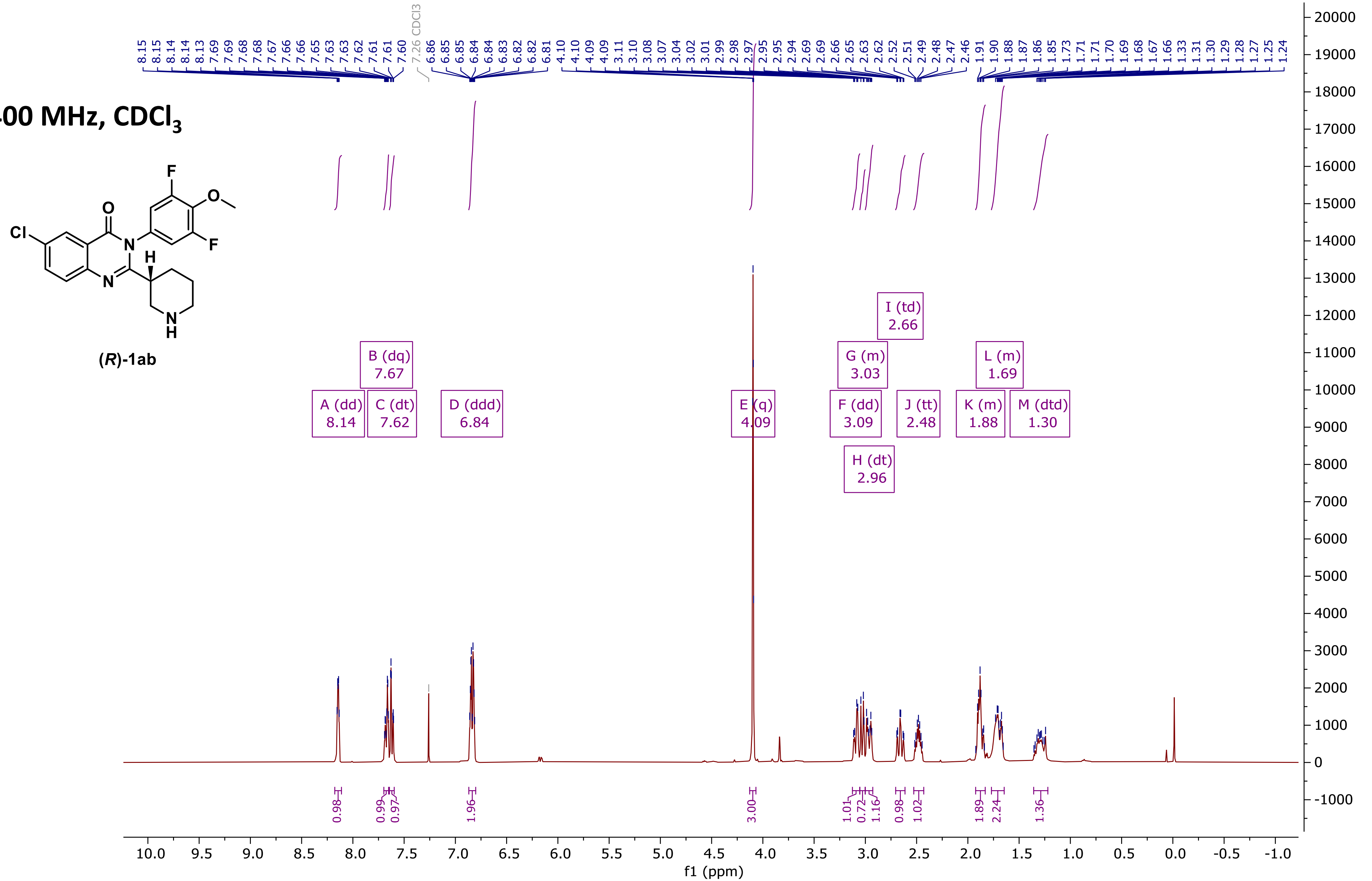

(*R*)-1ab

101 MHz, $CDCl_3$

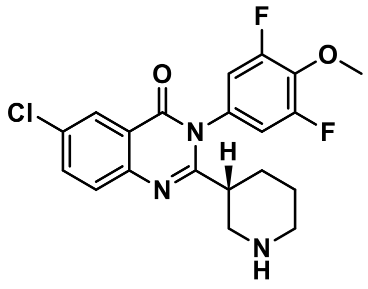

(*R*)-1ab

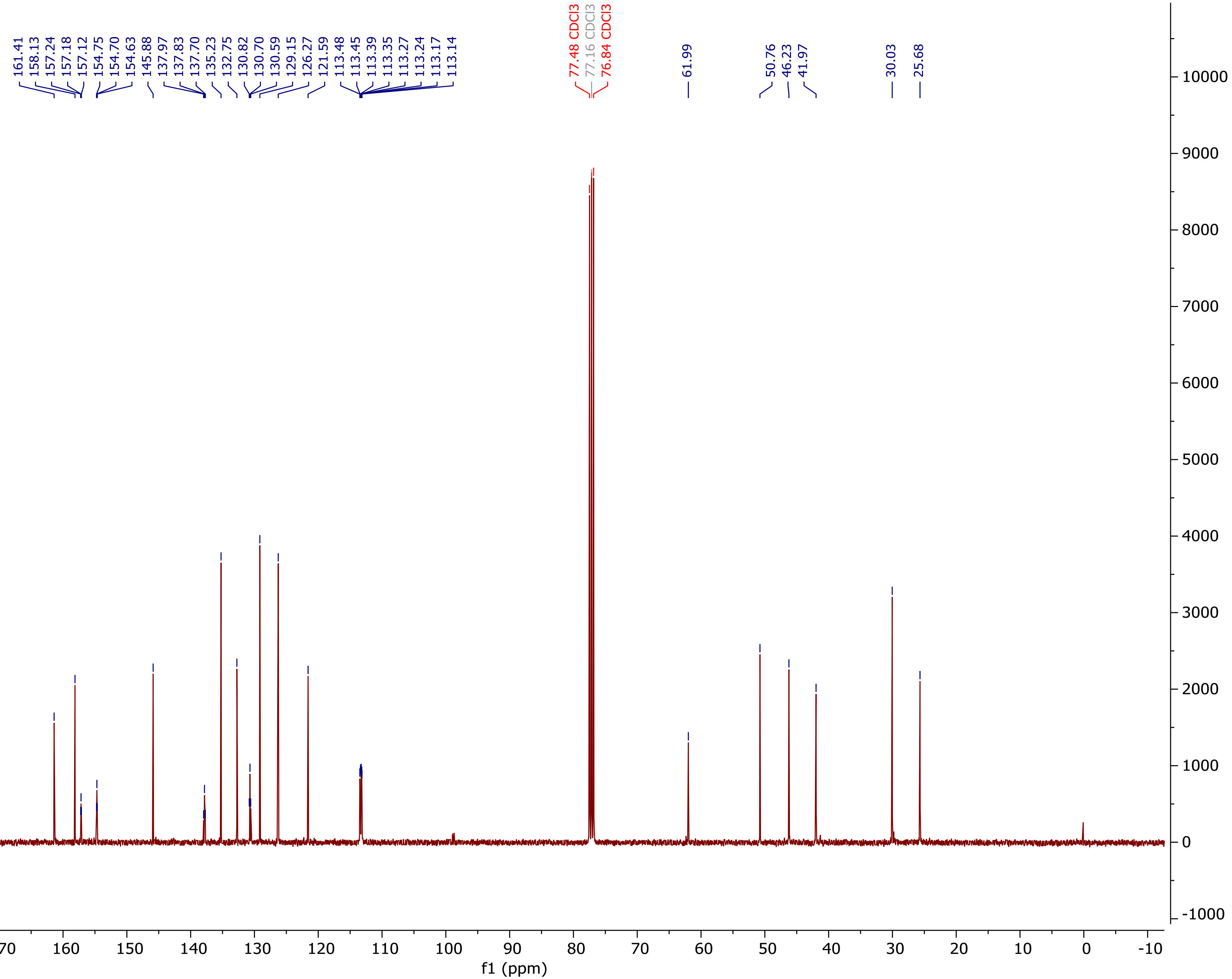

400 MHz, $CDCl_3$

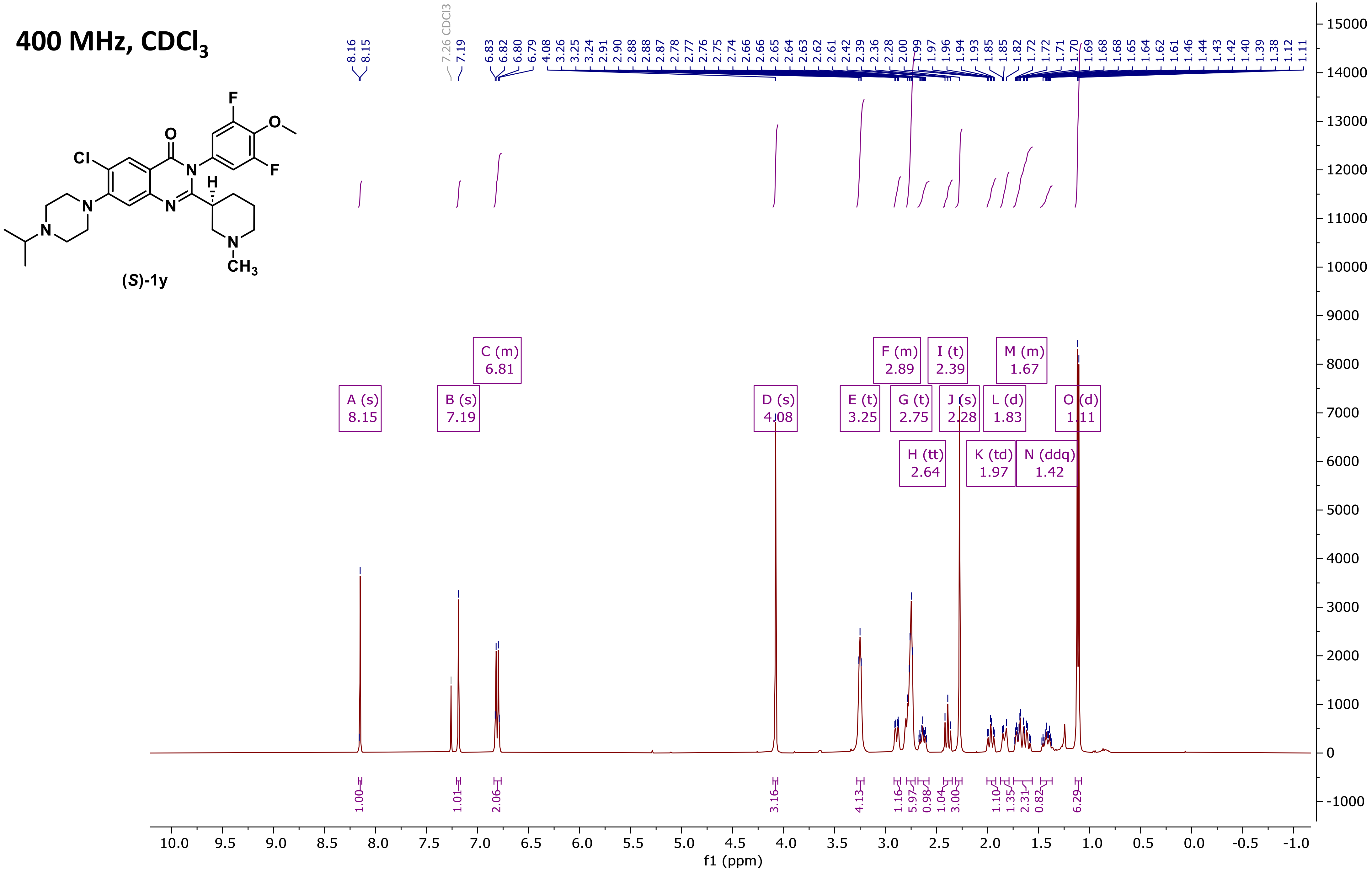

101 MHz, $CDCl_3$

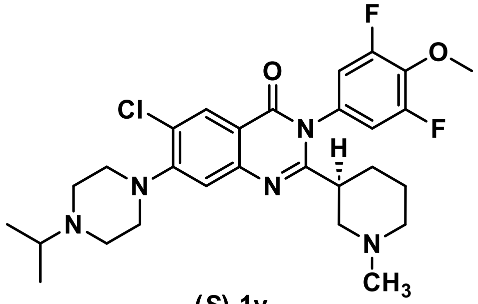

(*S*)-1y

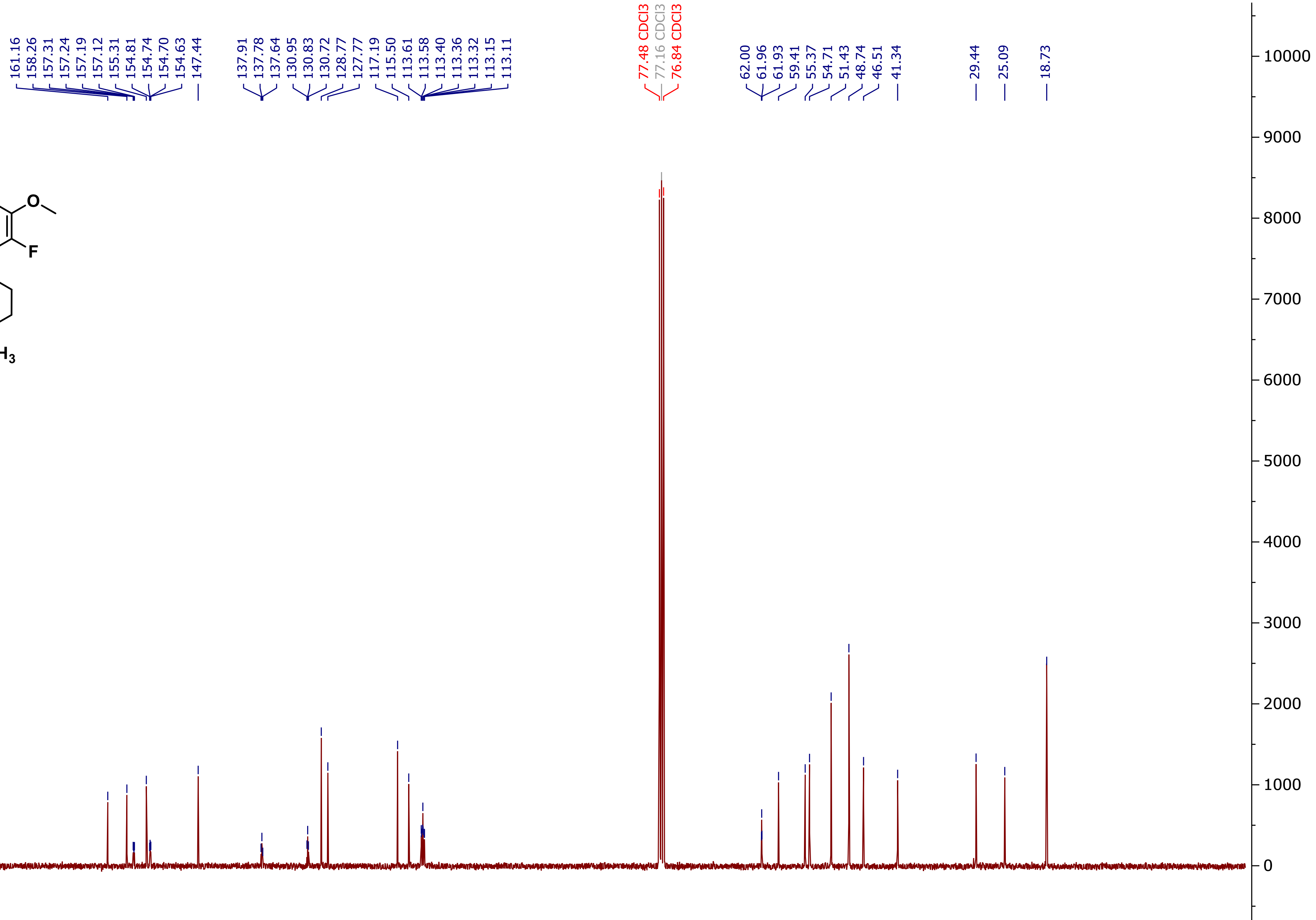

400 MHz, $CDCl_3$

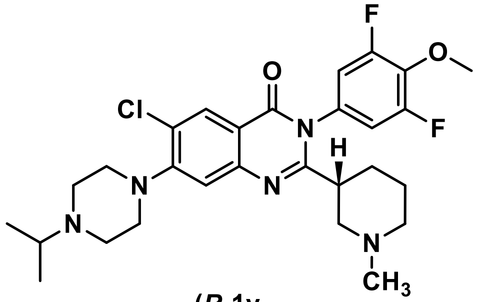

(*R*-1y

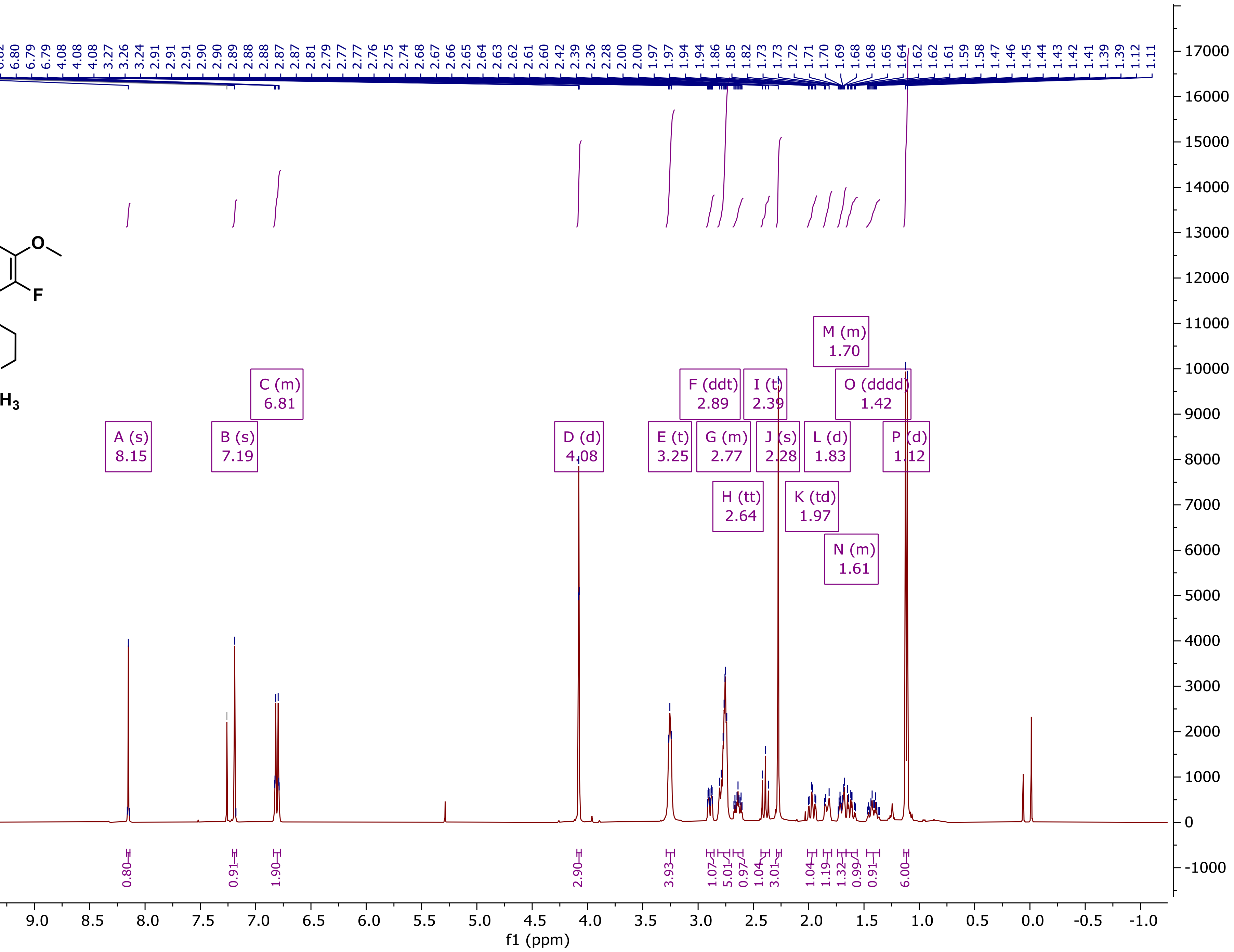

101 MHz, $CDCl_3$

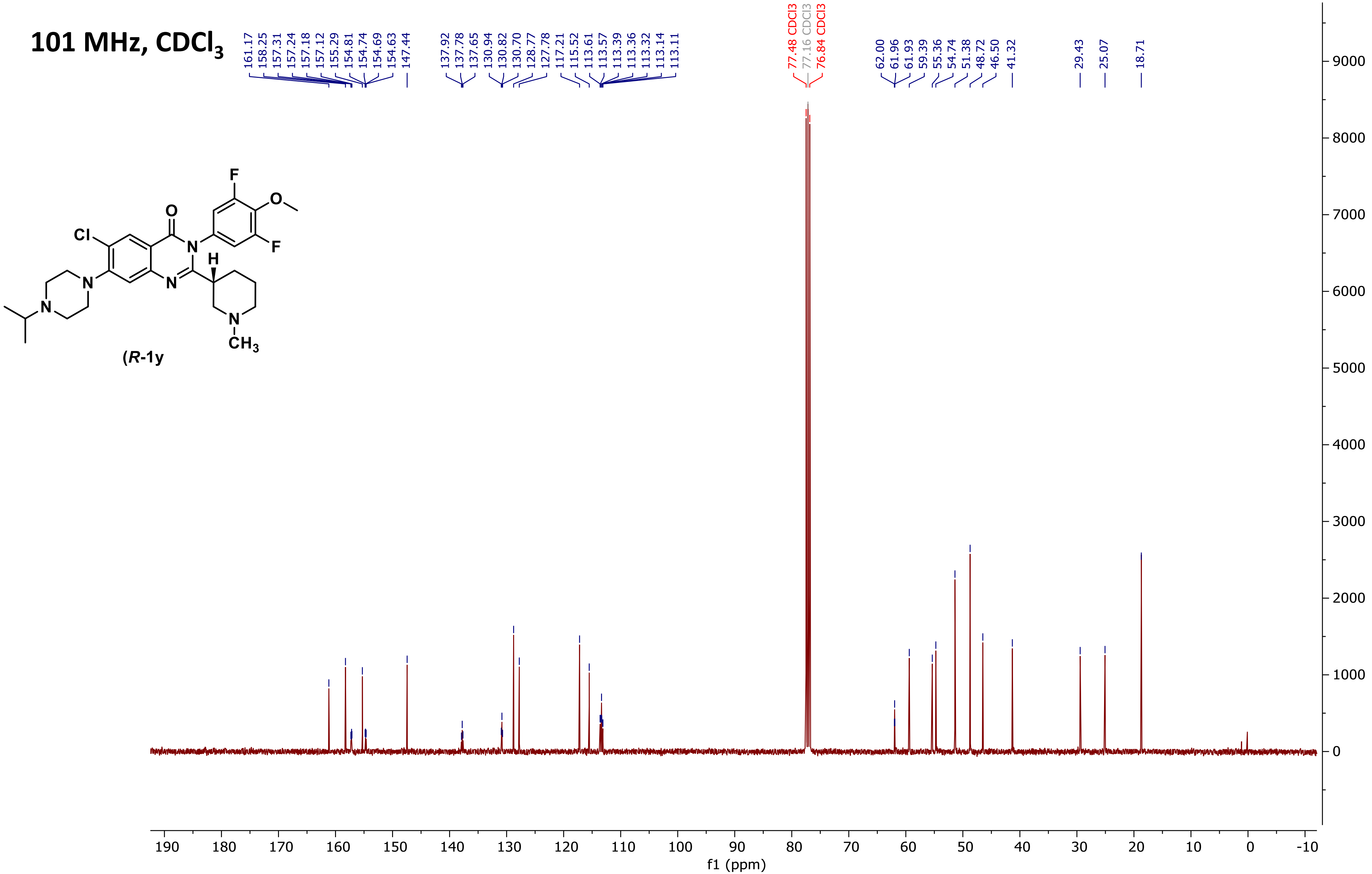

(*R*-1y

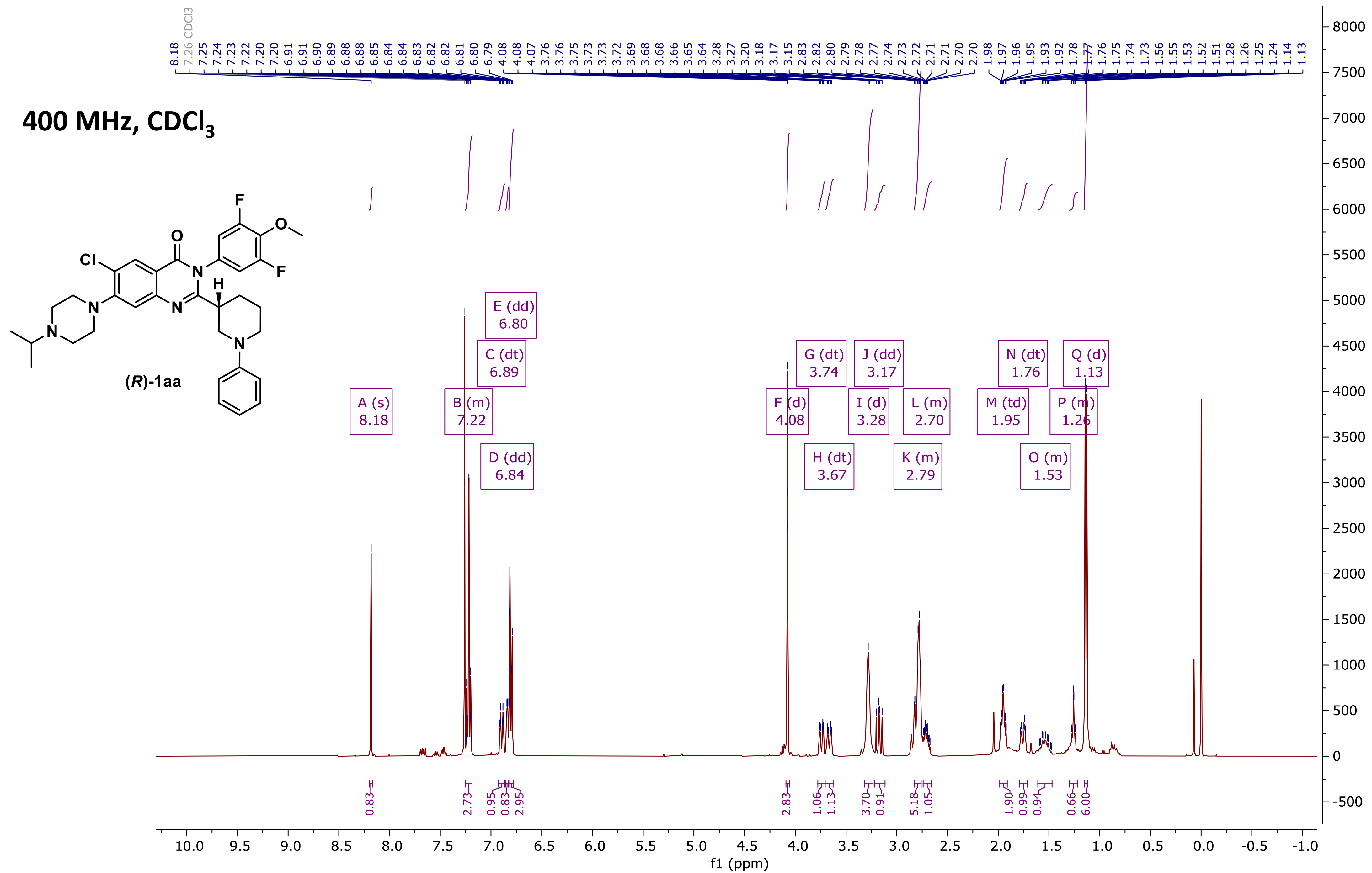
400 MHz, CDCl3
(R)-1aa
A (s) 8.18
B (m) 7.22
C (dt) 6.89
D (dd) 6.84
E (dd) 6.80
F (d) 4.08
G (dt) 3.74
H (dt) 3.67
I (d) 3.28
J (dd) 3.17
K (m) 2.79
L (m) 2.70
M (td) 1.95
N (dt) 1.76
O (m) 1.53
P (m) 1.26
Q (d) 1.13
f1 (ppm)

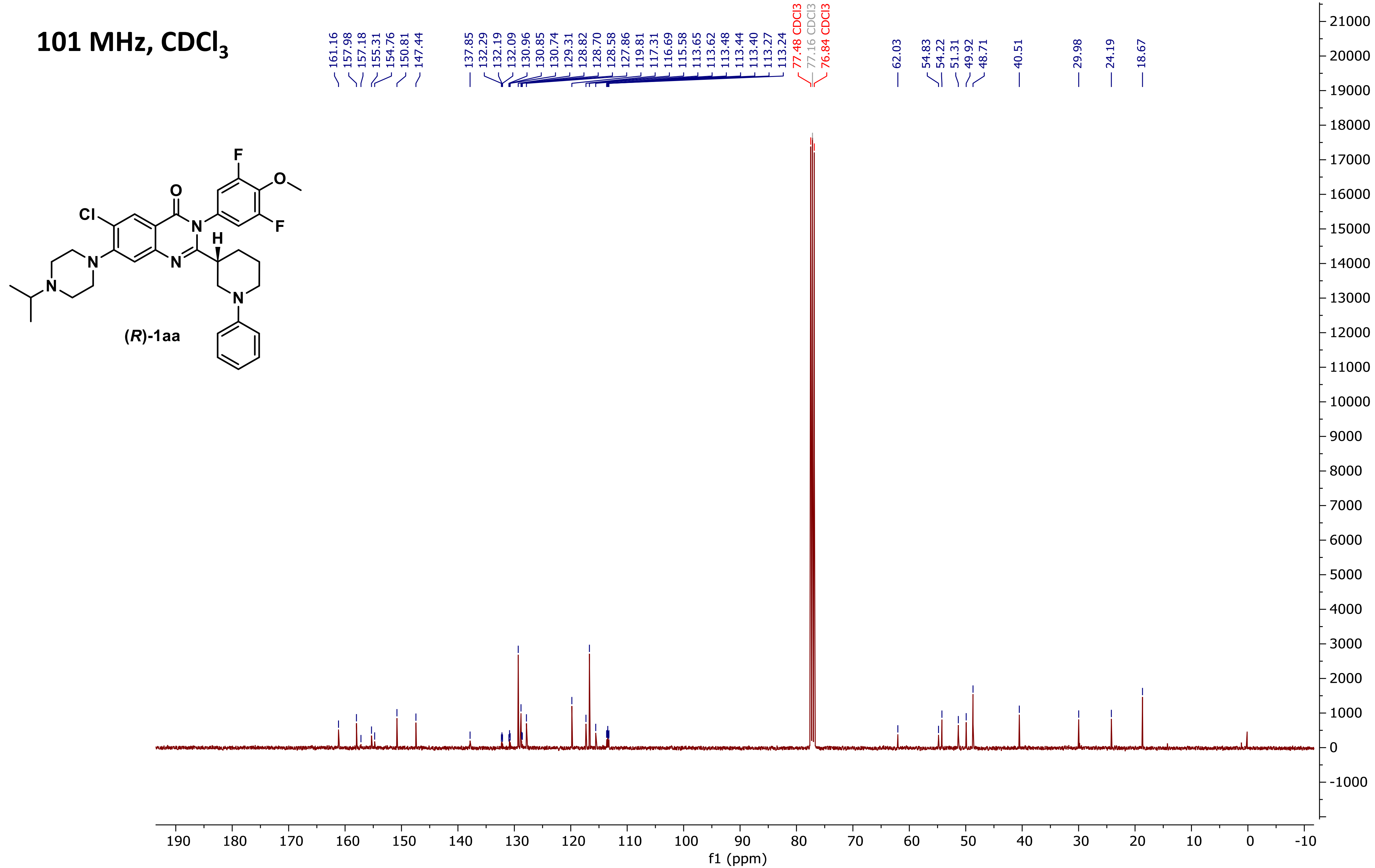
101 MHz, CDCl3
161.16
157.98
157.18
155.31
154.76
150.81
147.44
137.85
132.29
132.19
132.09
130.96
130.85
130.74
129.31
128.82
128.70
128.58
127.86
119.81
117.31
116.69
115.58
113.65
113.62
113.48
113.44
113.40
113.27
113.24
77.48 CDCl3
77.16 CDCl3
76.84 CDCl3
62.03
54.83
54.22
51.31
49.92
48.71
40.51
29.98
24.19
18.67
(R)-1aa
f1 (ppm)

400 MHz, DMSO-D_6

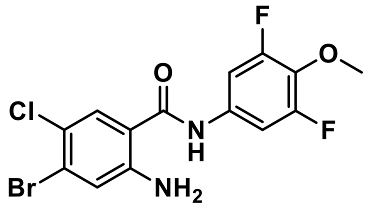

5b

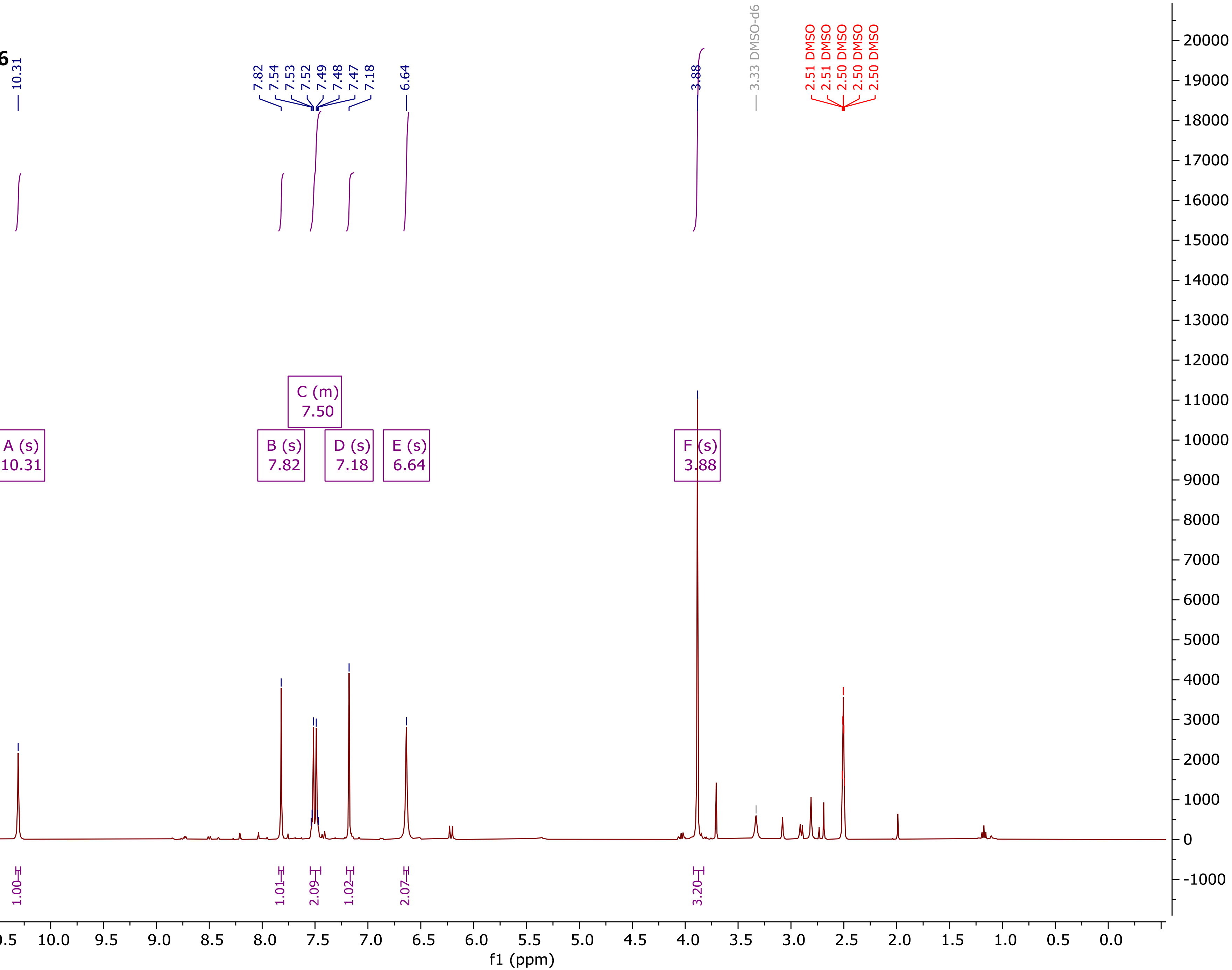

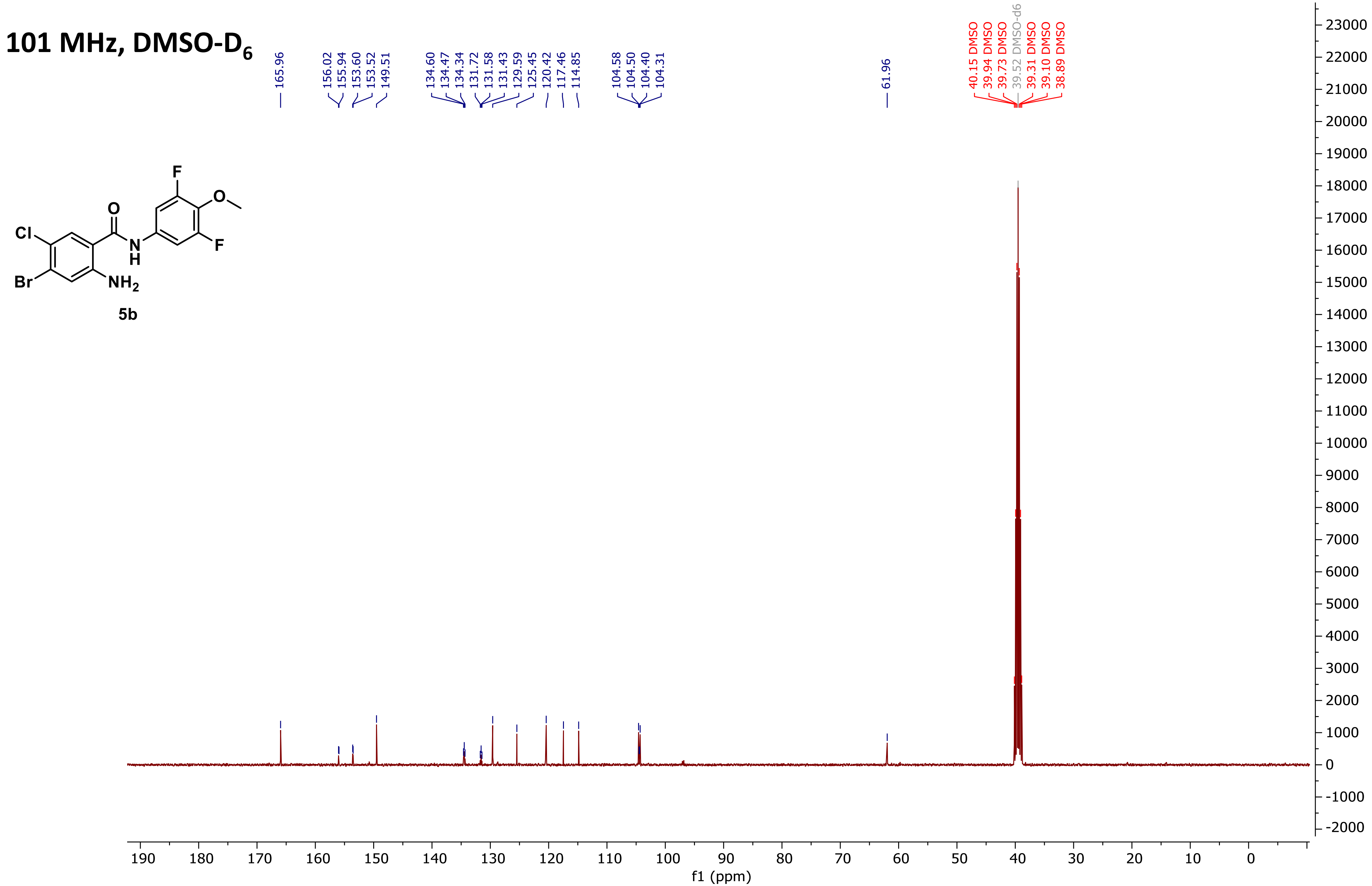

101 MHz, DMSO-D6
165.96
156.02
155.94
153.60
153.52
149.51
134.60
134.47
134.34
131.72
131.58
131.43
129.59
125.45
120.42
117.46
114.85
104.58
104.50
104.40
104.31
61.96
40.15 DMSO
39.94 DMSO
39.73 DMSO
39.52 DMSO-d6
39.31 DMSO
39.10 DMSO
38.89 DMSO
5b
f1 (ppm)

400 MHz, DMSO-D_6

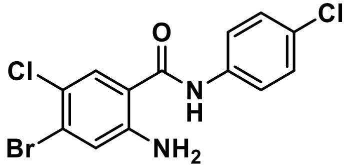

5c

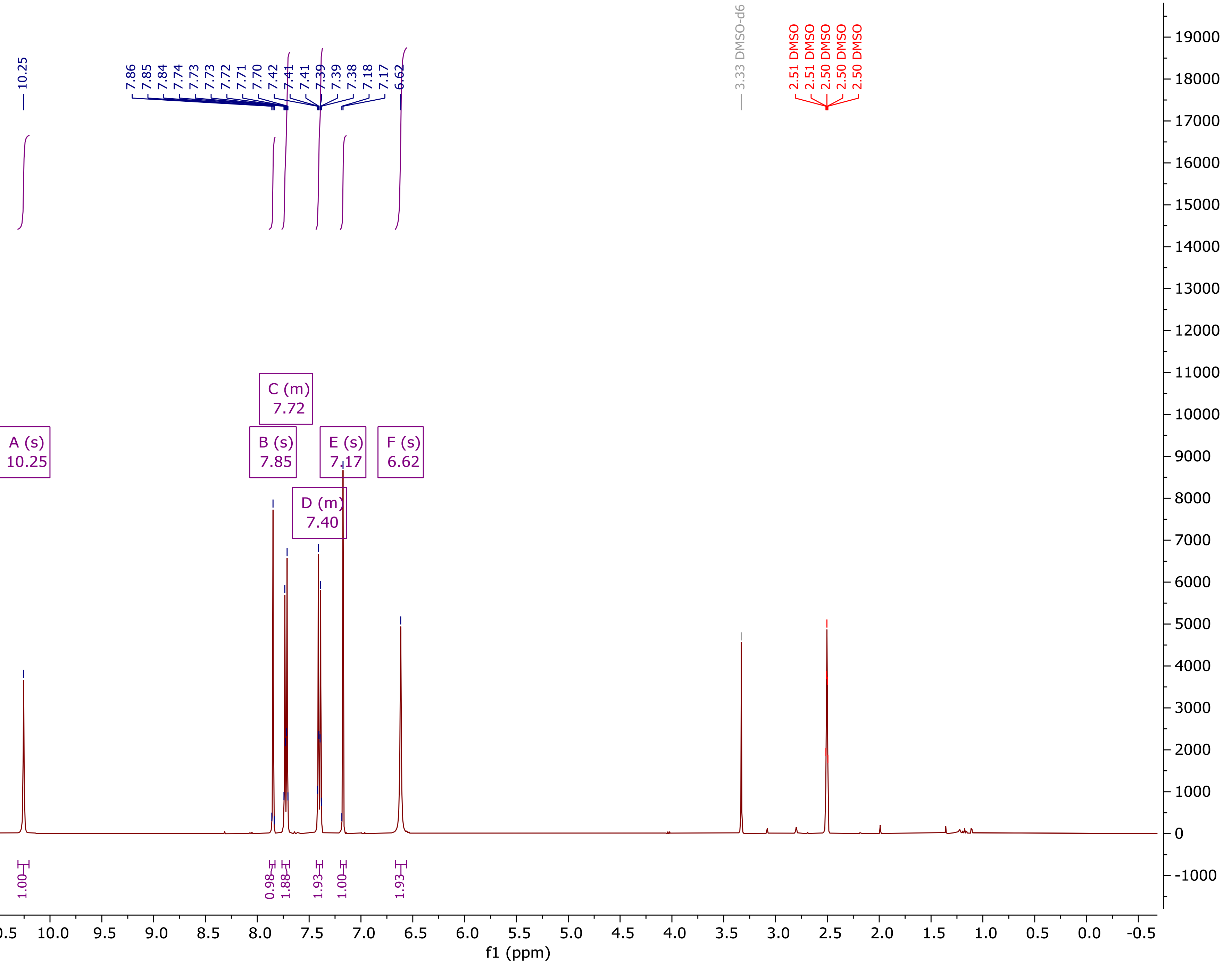

101 MHz, DMSO-D$_6$

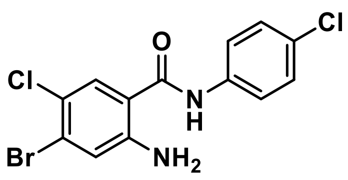

5c

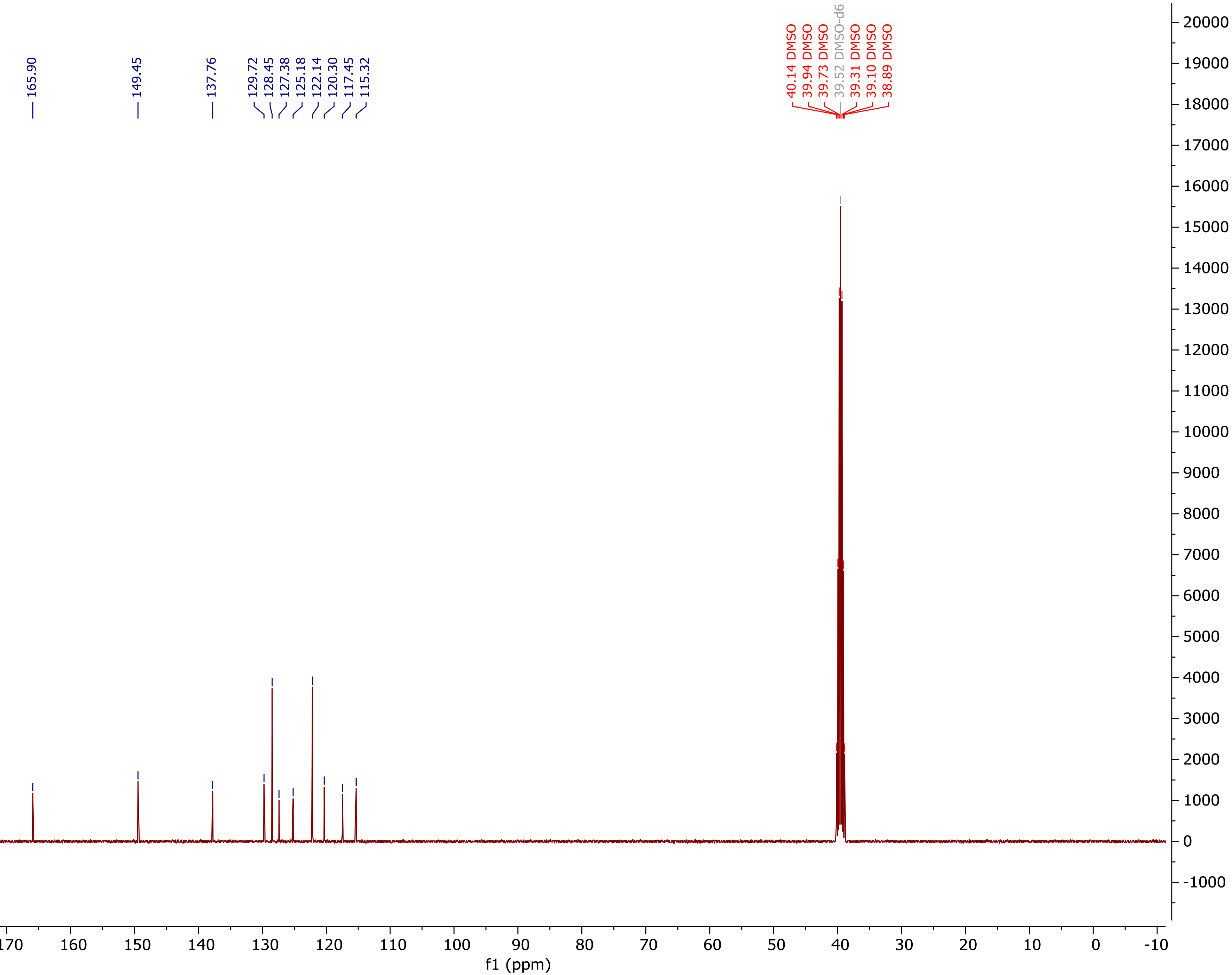

400 MHz, CDCl$_3$

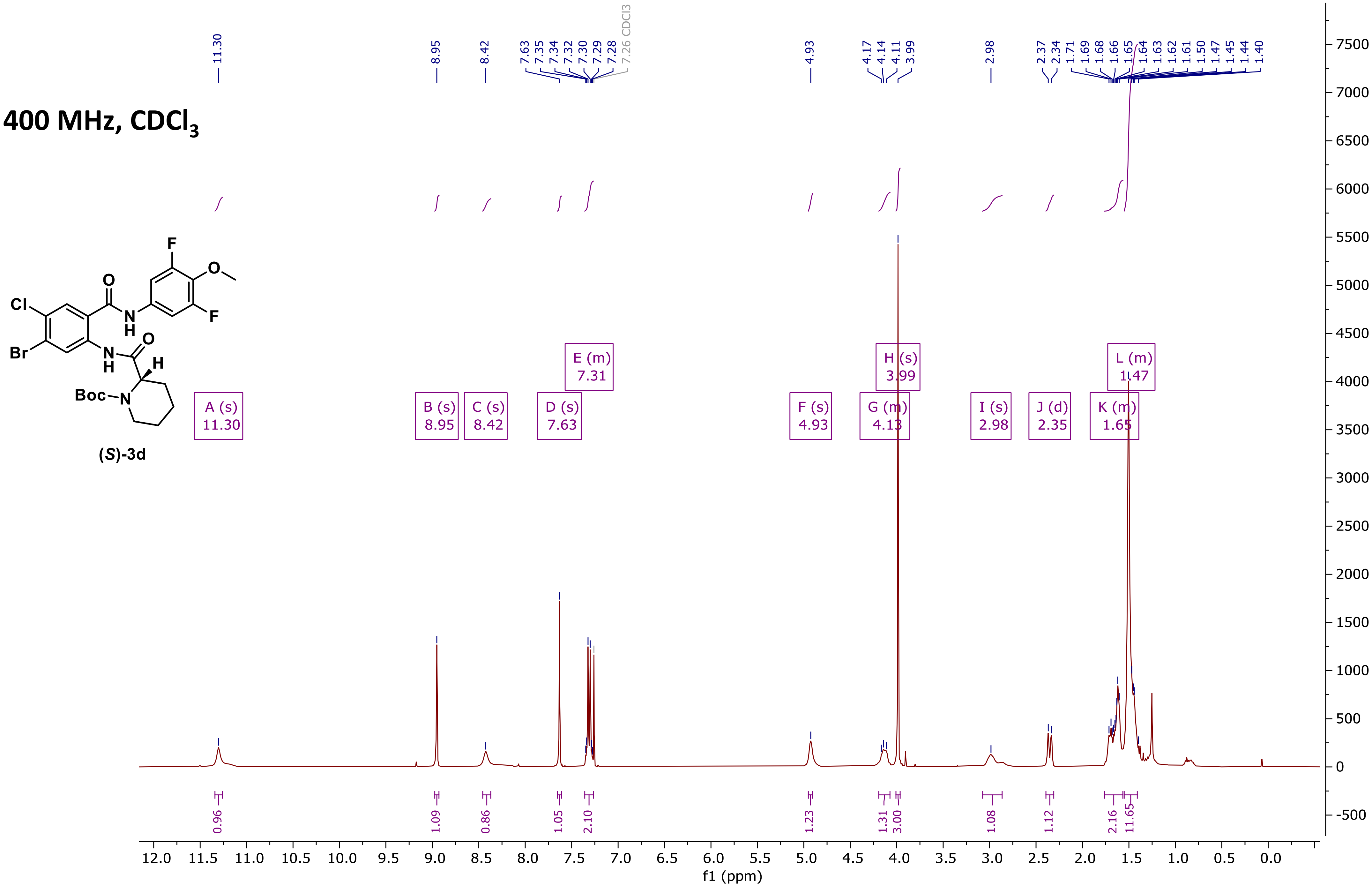

101 MHz, $CDCl_3$

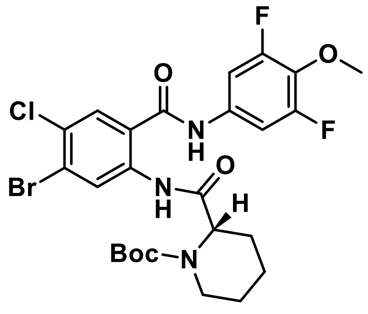

(*S*)-3d

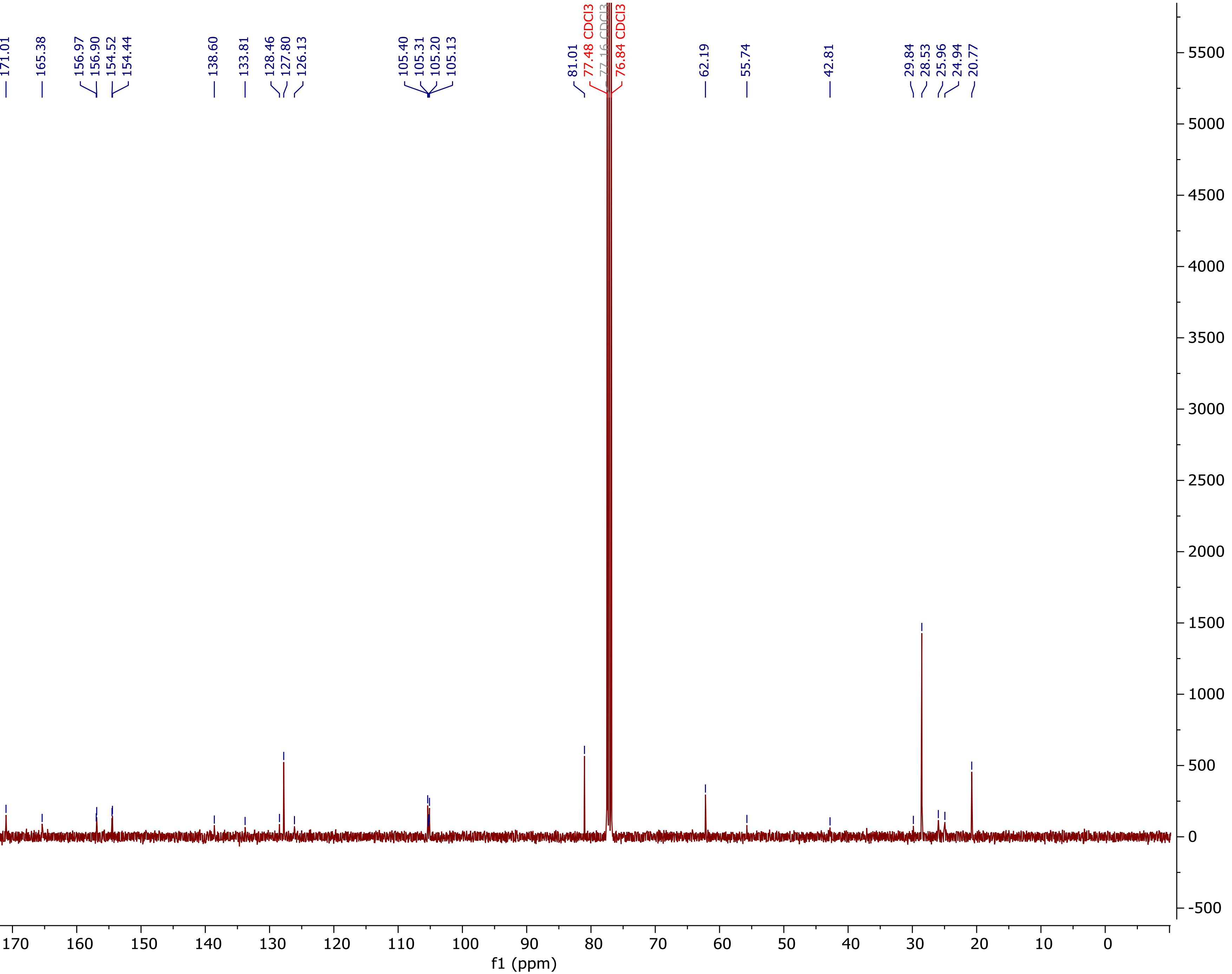

400 MHz, $CDCl_3$

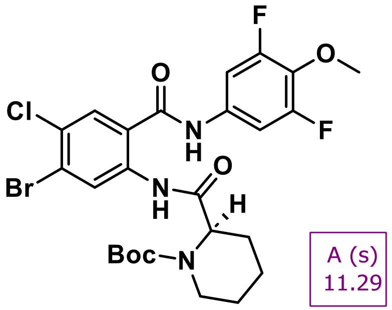

(*R*)-3d

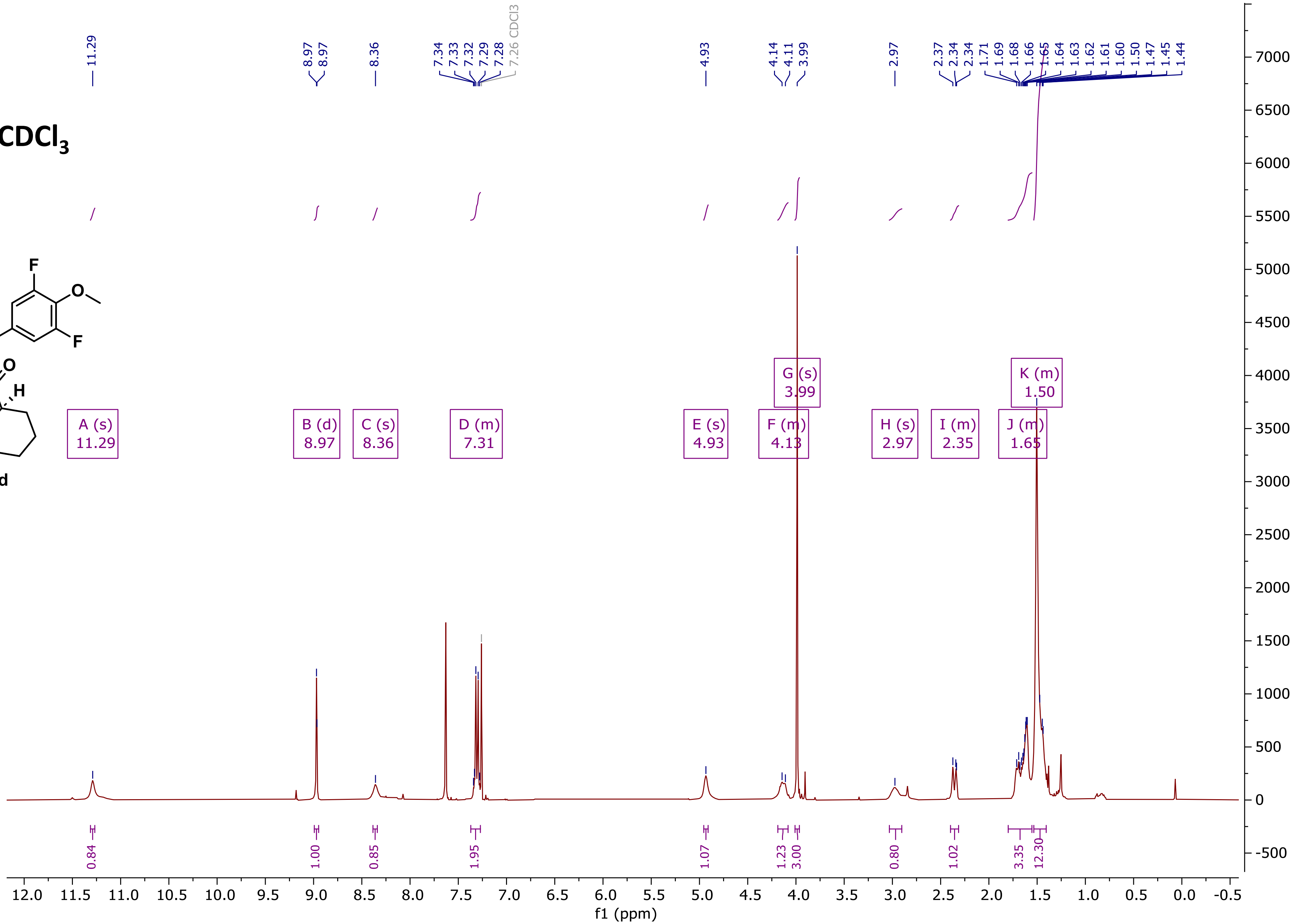

101 MHz, $CDCl_3$

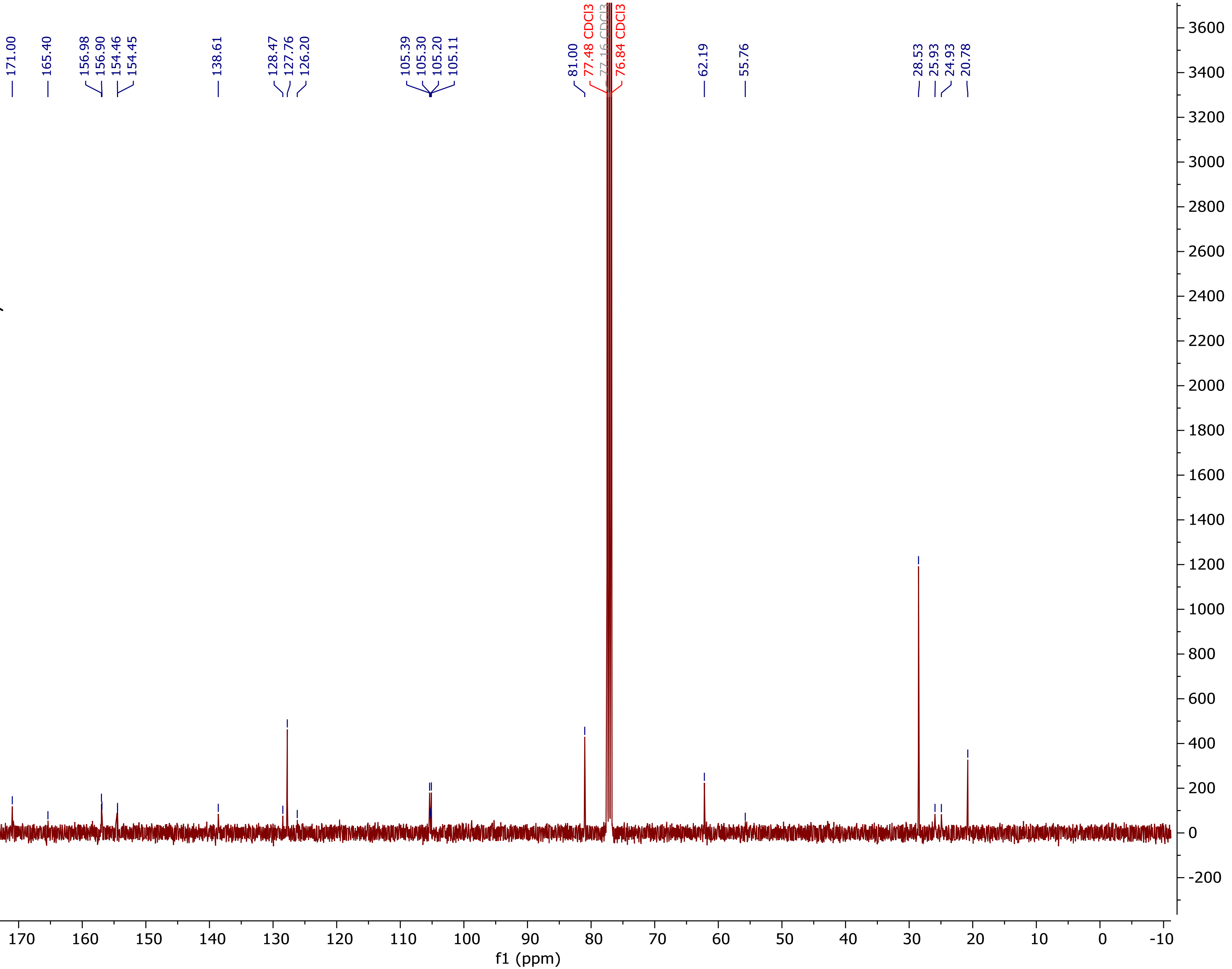

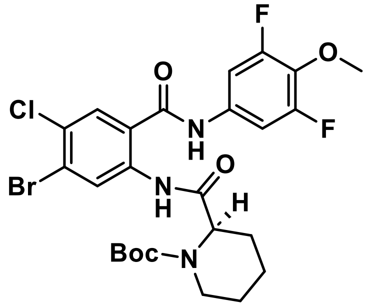

(*R*)-3d

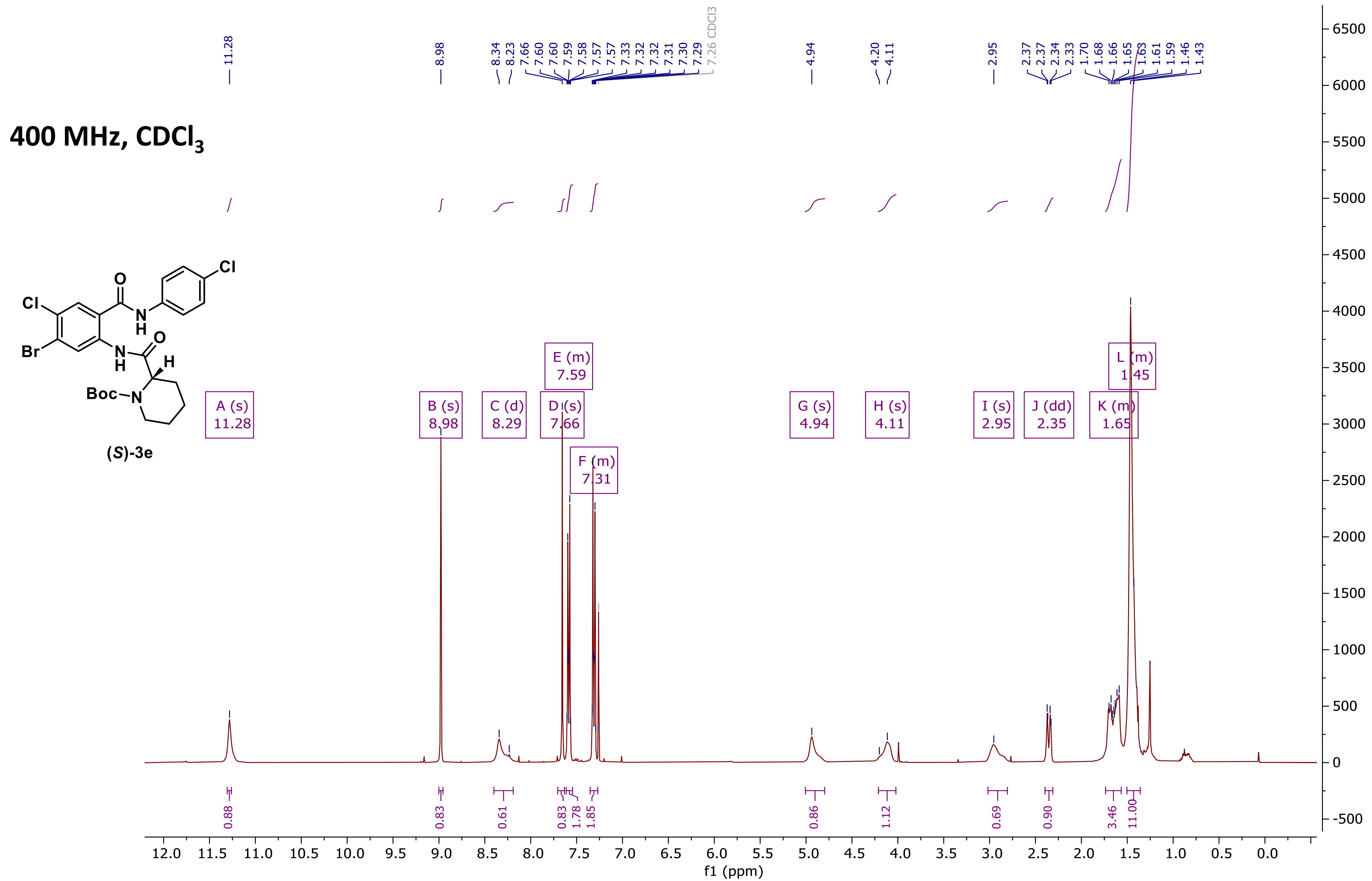

400 MHz, CDCl3
(S)-3e
A (s) 11.28
B (s) 8.98
C (d) 8.29
D (s) 7.66
E (m) 7.59
F (m) 7.31
G (s) 4.94
H (s) 4.11
I (s) 2.95
J (dd) 2.35
K (m) 1.65
L (m) 1.45
f1 (ppm)

101 MHz, $CDCl_3$

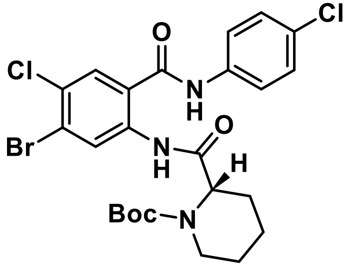

(*S*)-3e

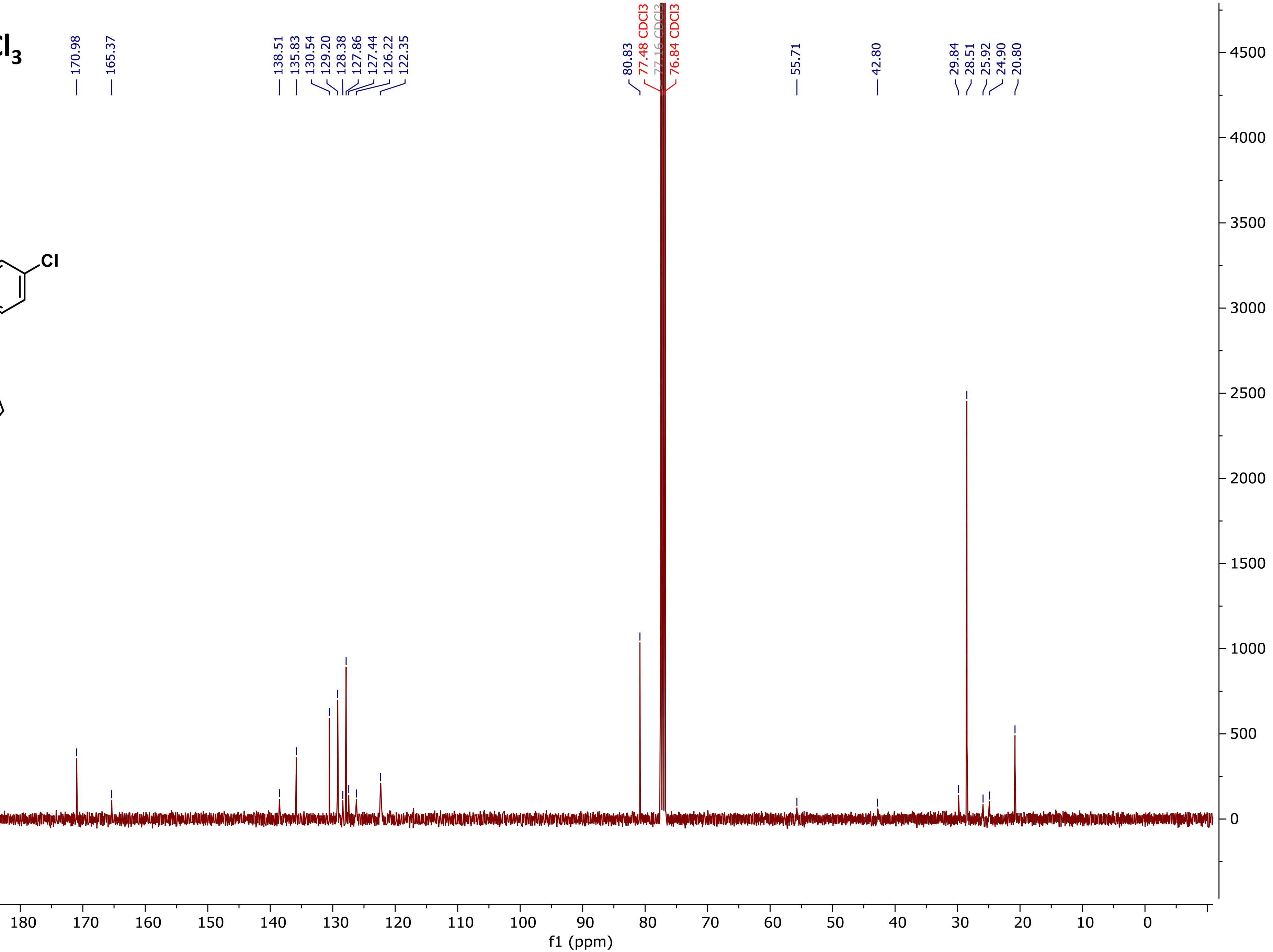

400 MHz, $CDCl_3$

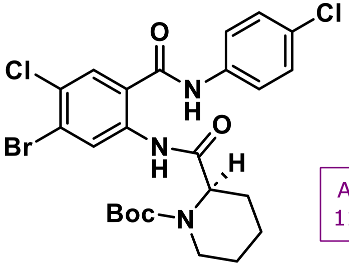

(*R*)-3e

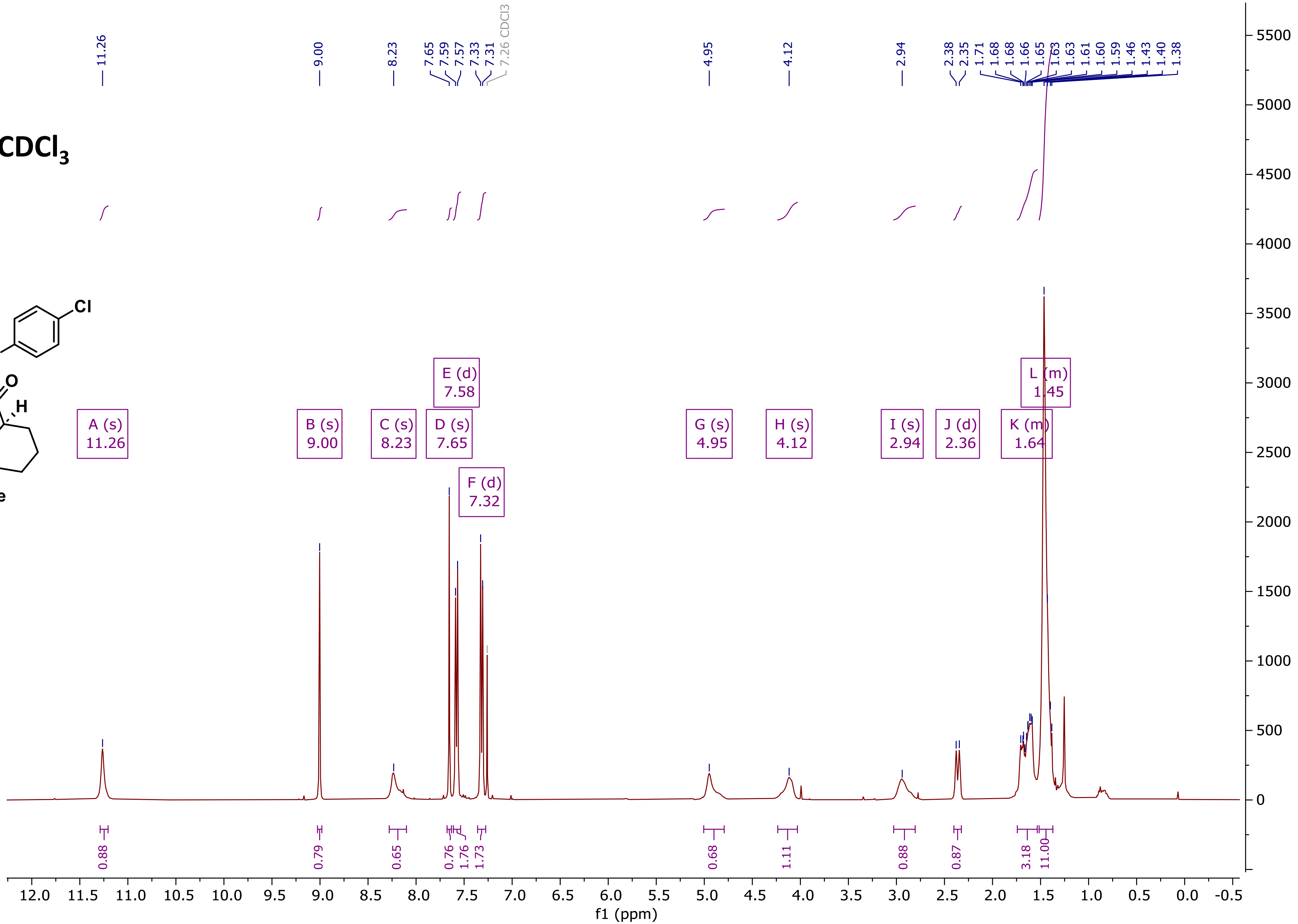

101 MHz, $CDCl_3$

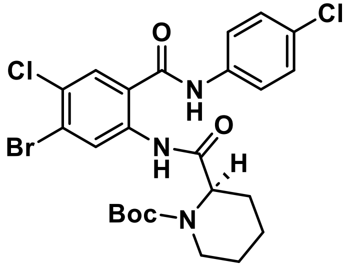

(*R*)-3e

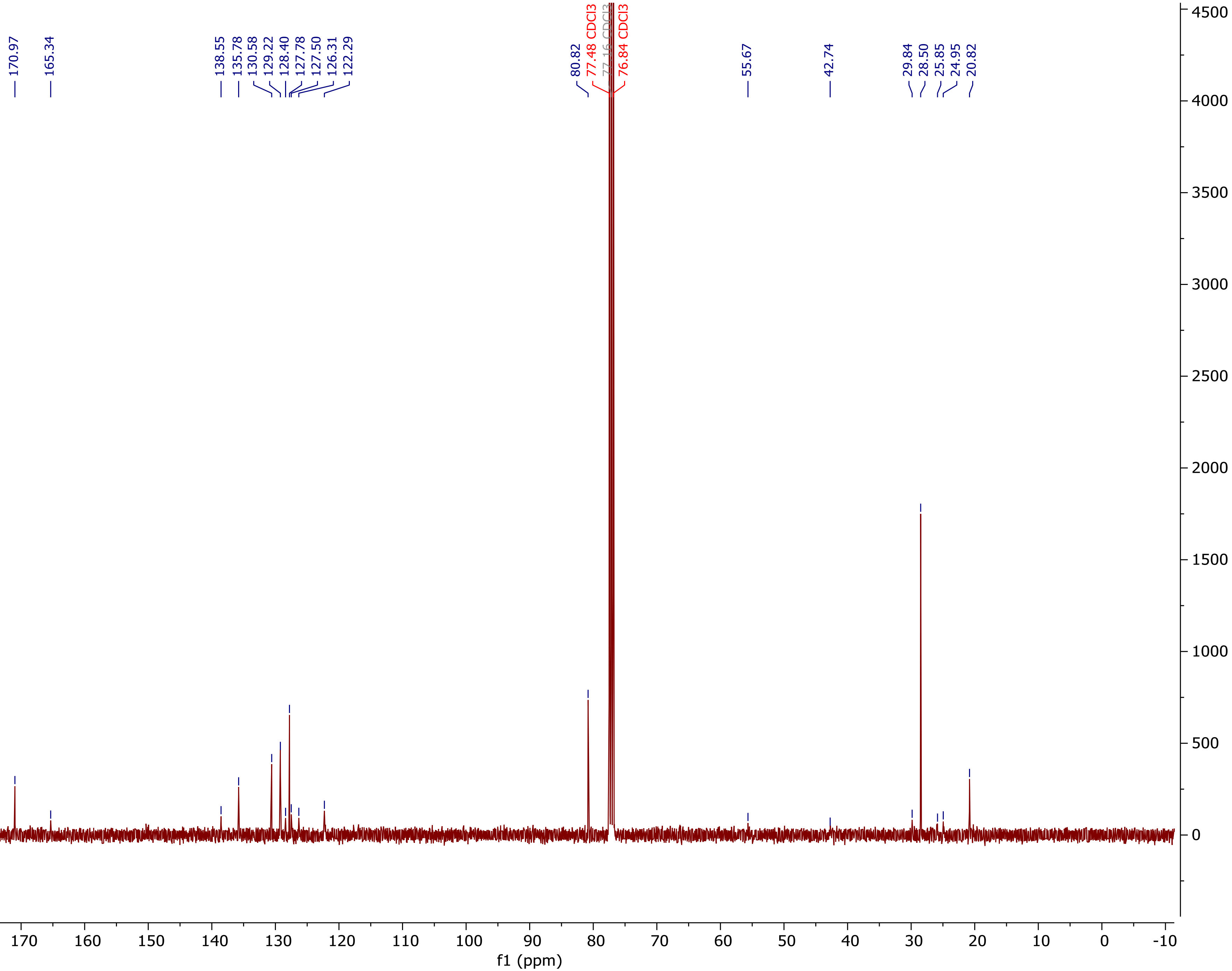

400 MHz, $CDCl_3$

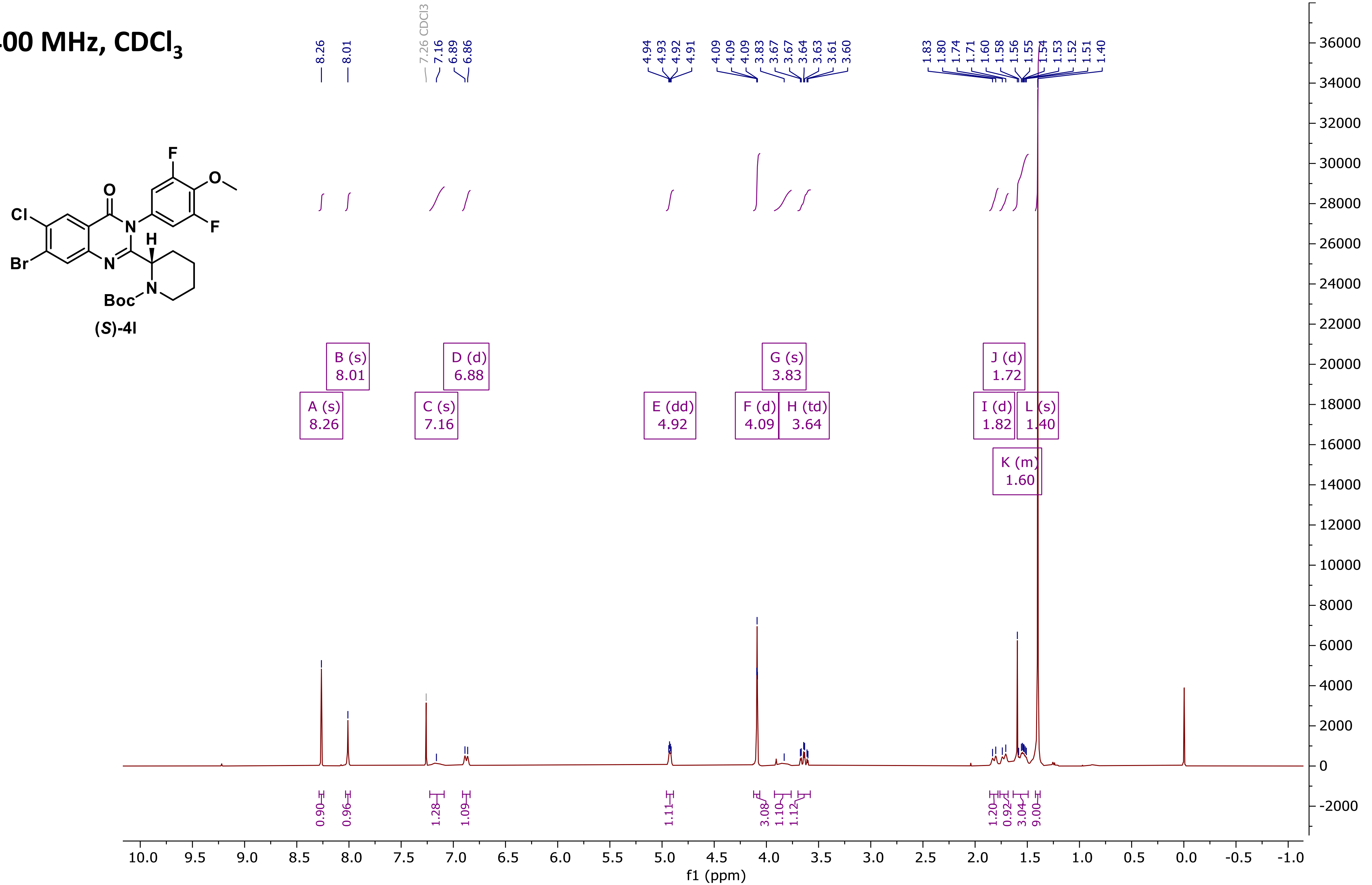

101 MHz, $CDCl_3$

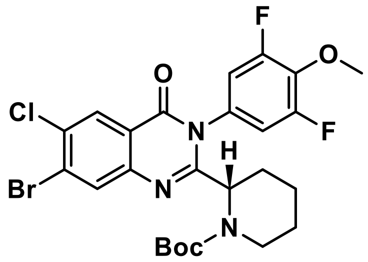

(*S*)-4l

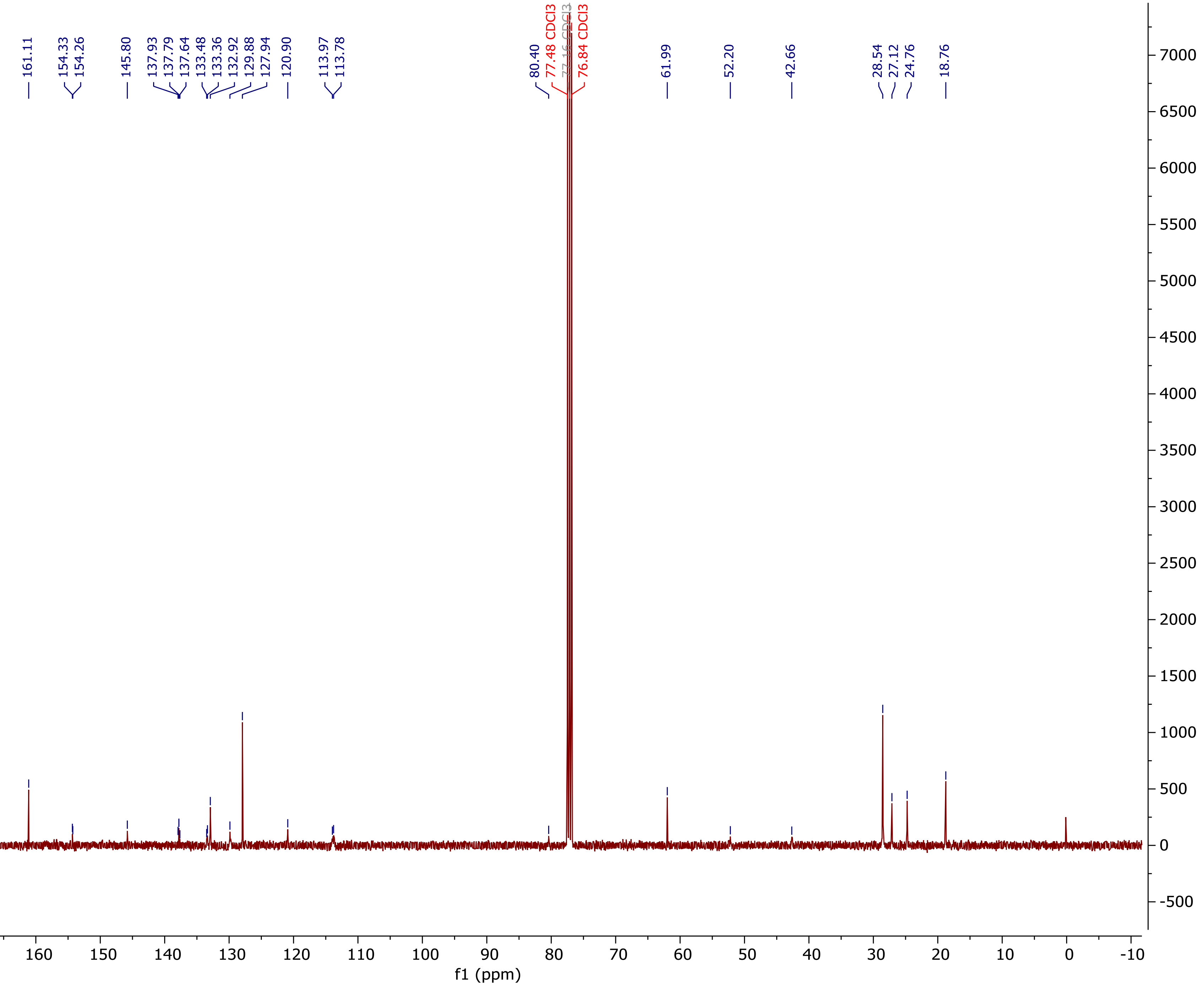

400 MHz, $CDCl_3$

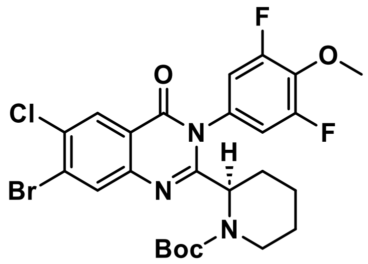

(*R*)-4l

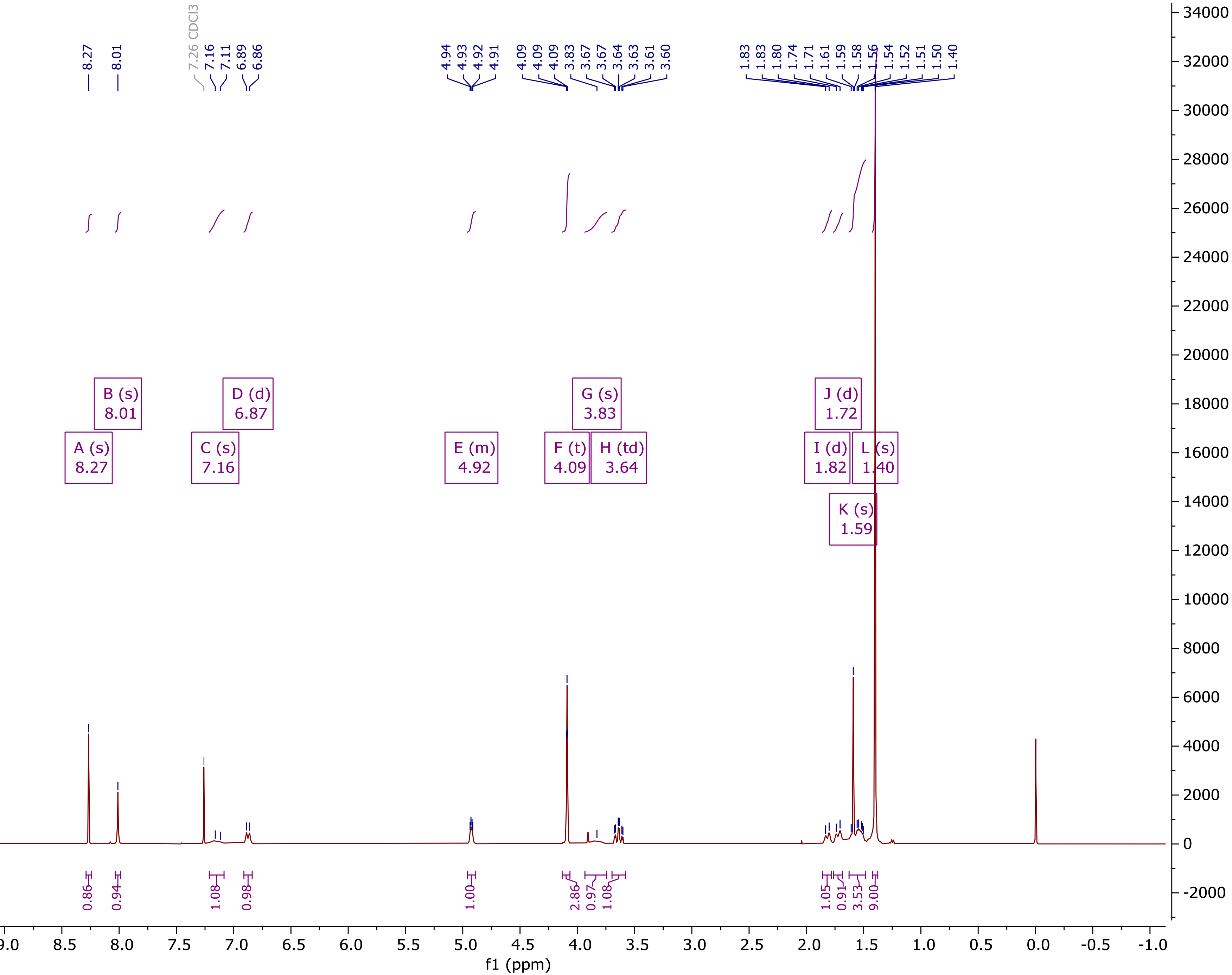

101 MHz, $CDCl_3$

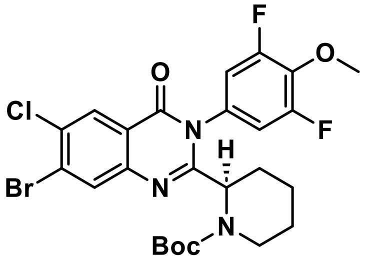

(*R*)-4l

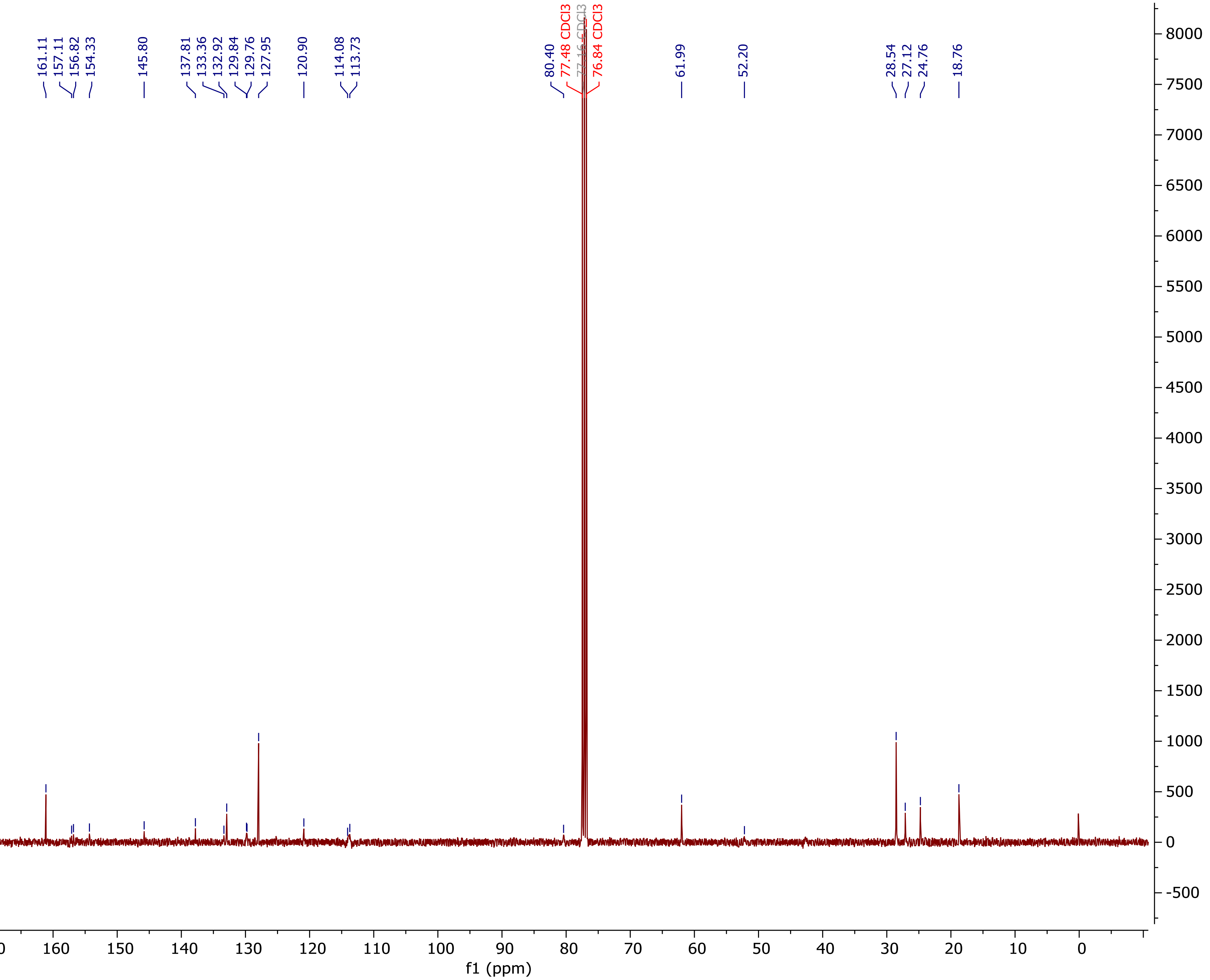

400 MHz, $CDCl_3$

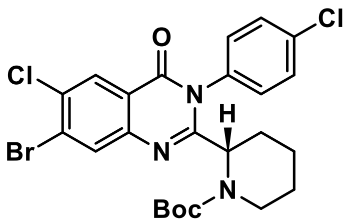

(*S*)-4m

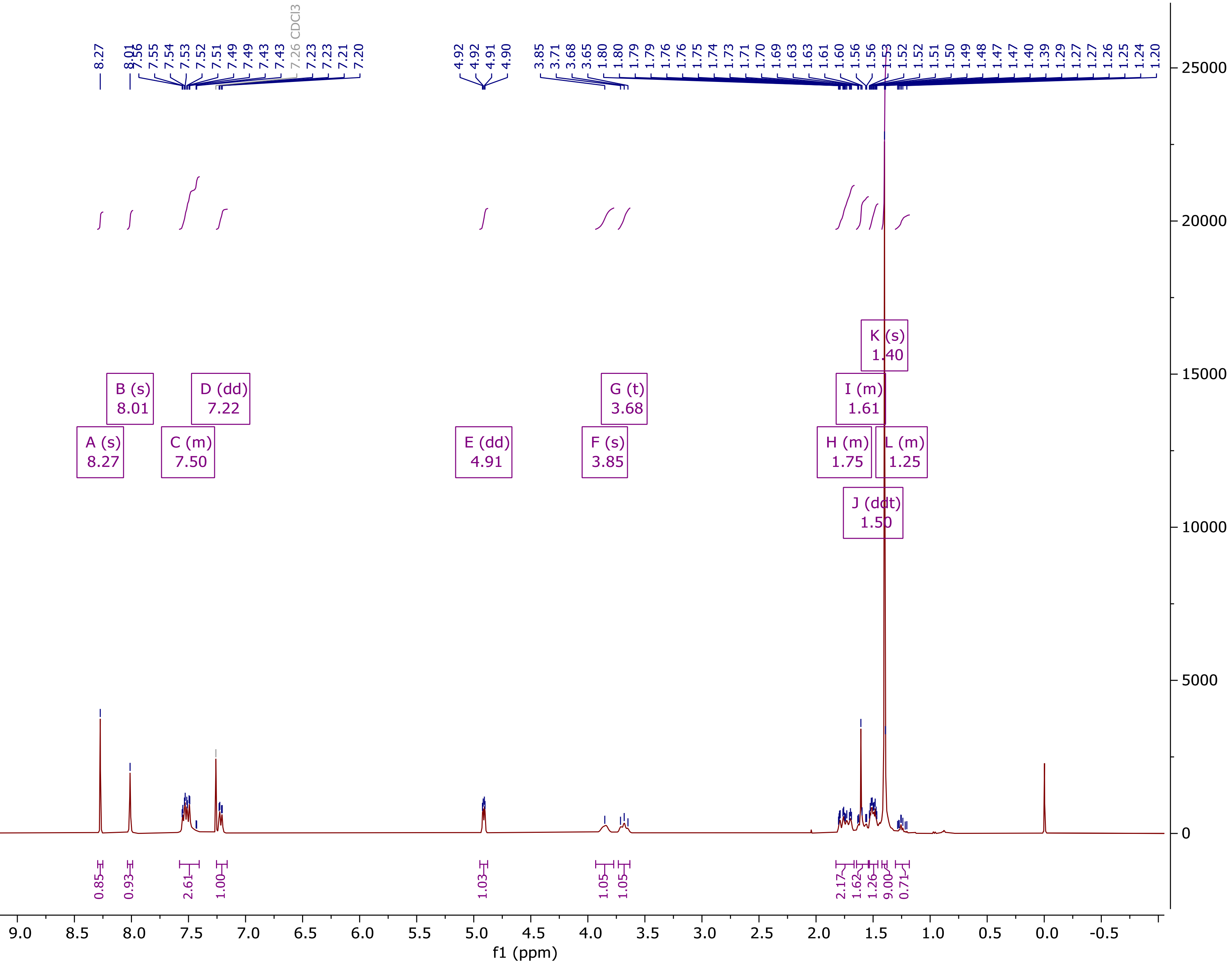

101 MHz, $CDCl_3$

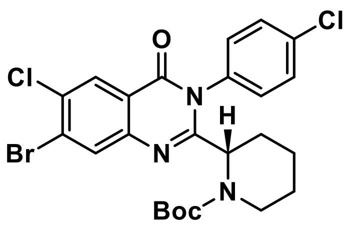

(*S*)-4m

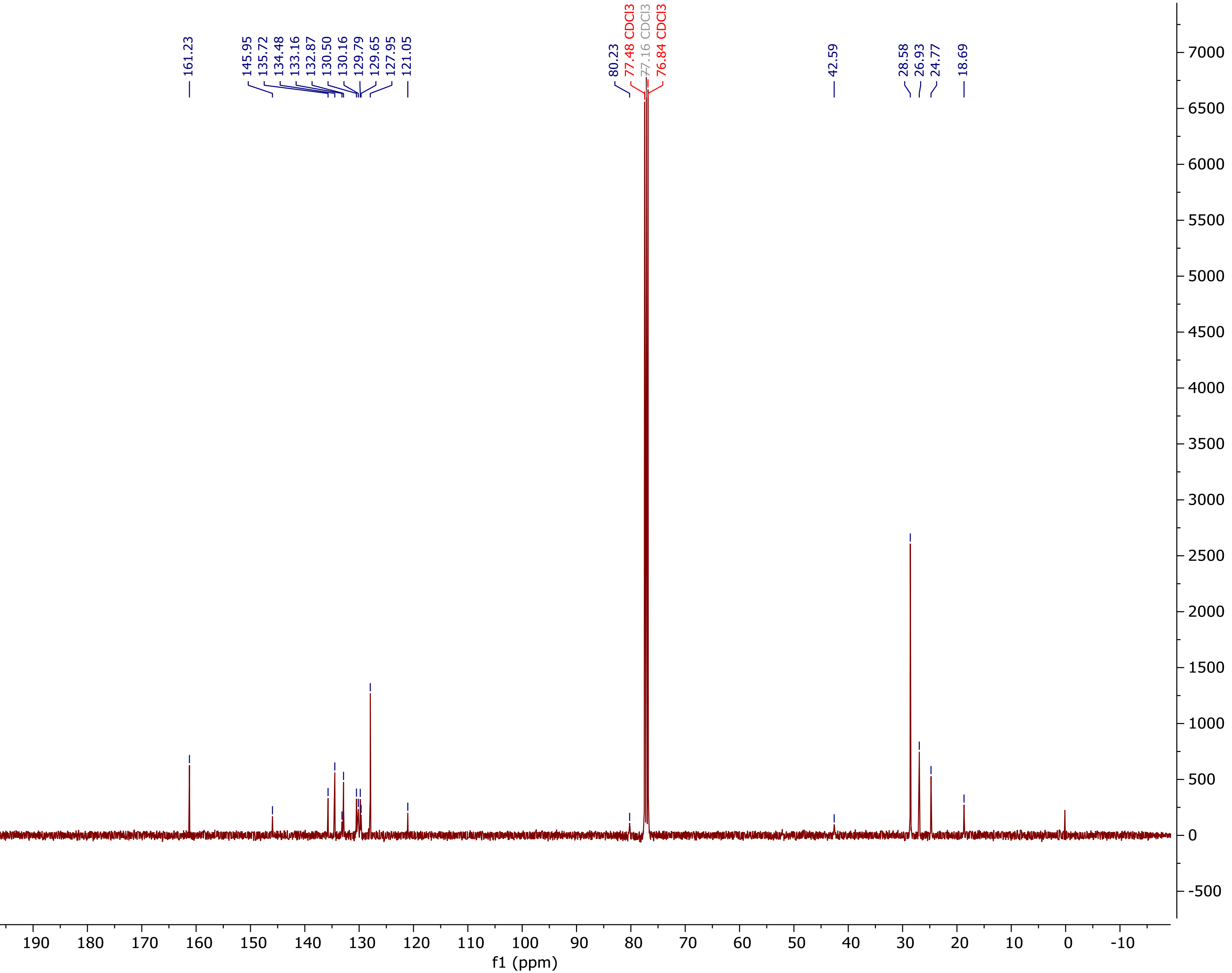

400 MHz, CDCl3

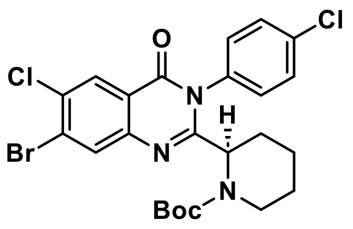

(*R*)-4l

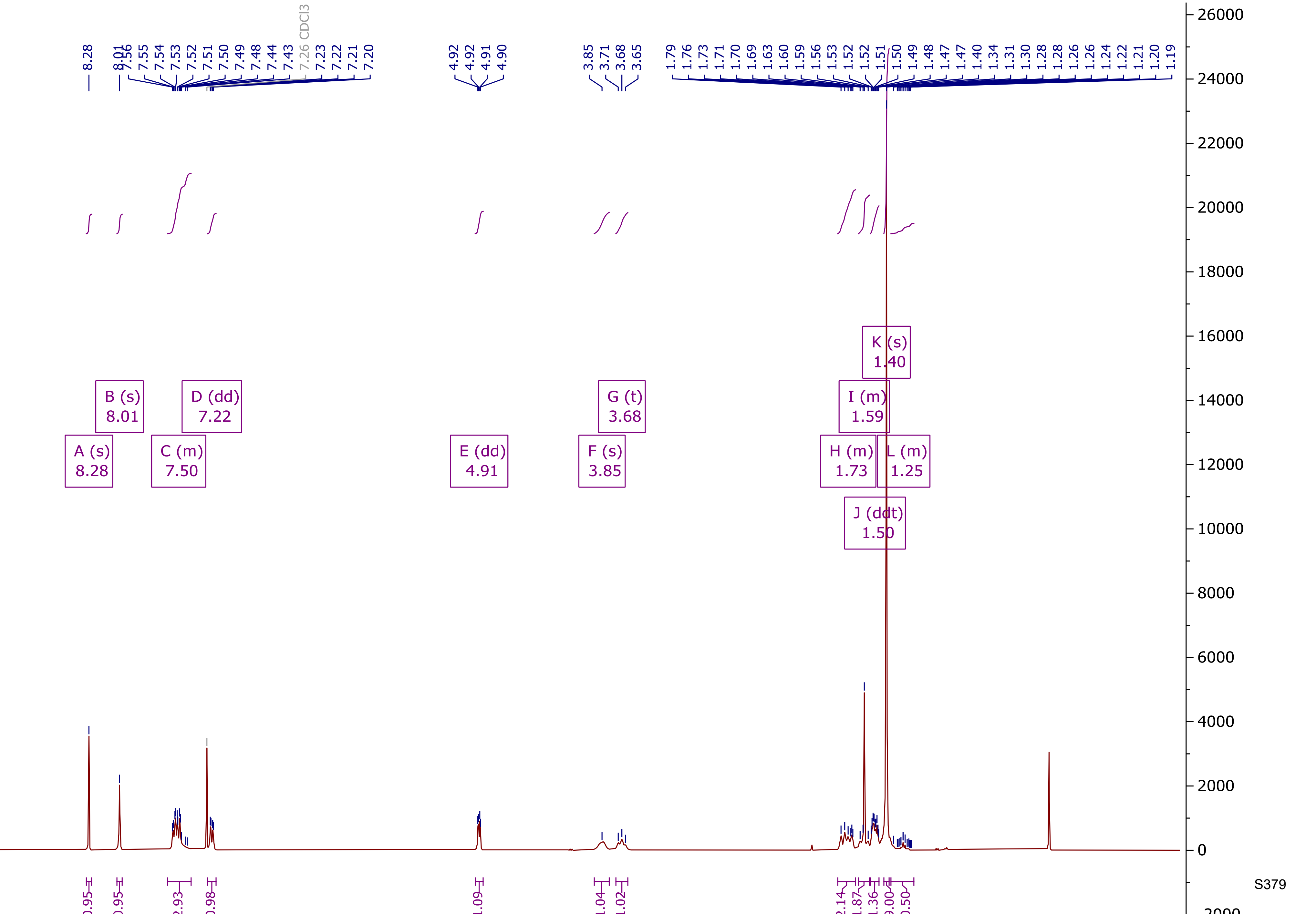

101 MHz, $CDCl_3$

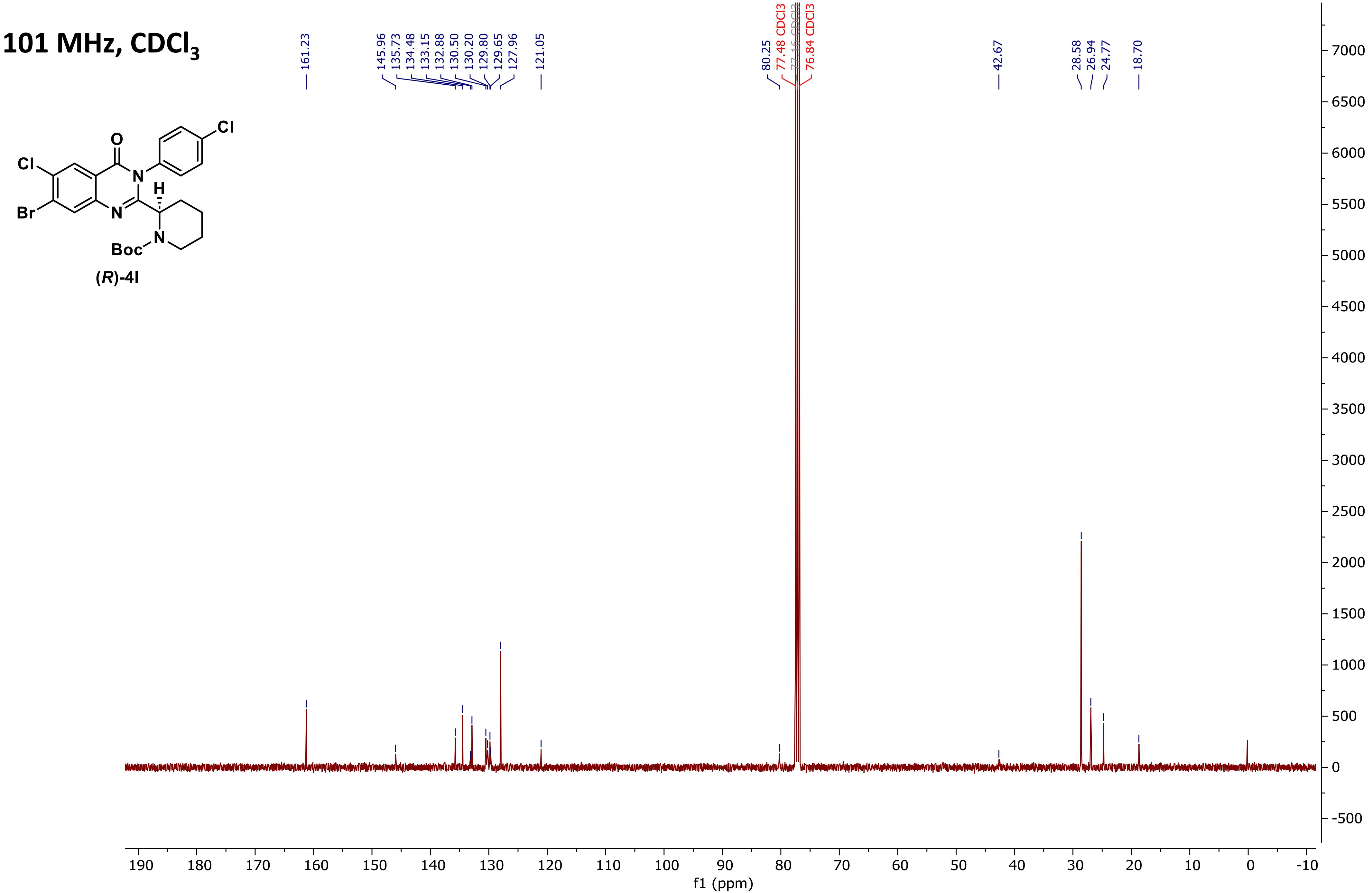

400 MHz, $CDCl_3$

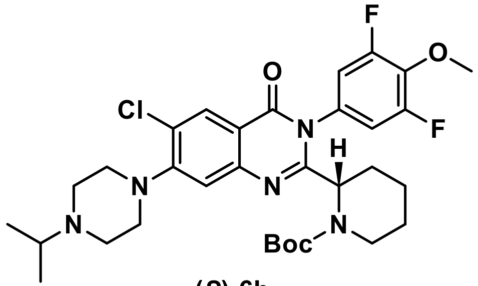

(*S*)-6h

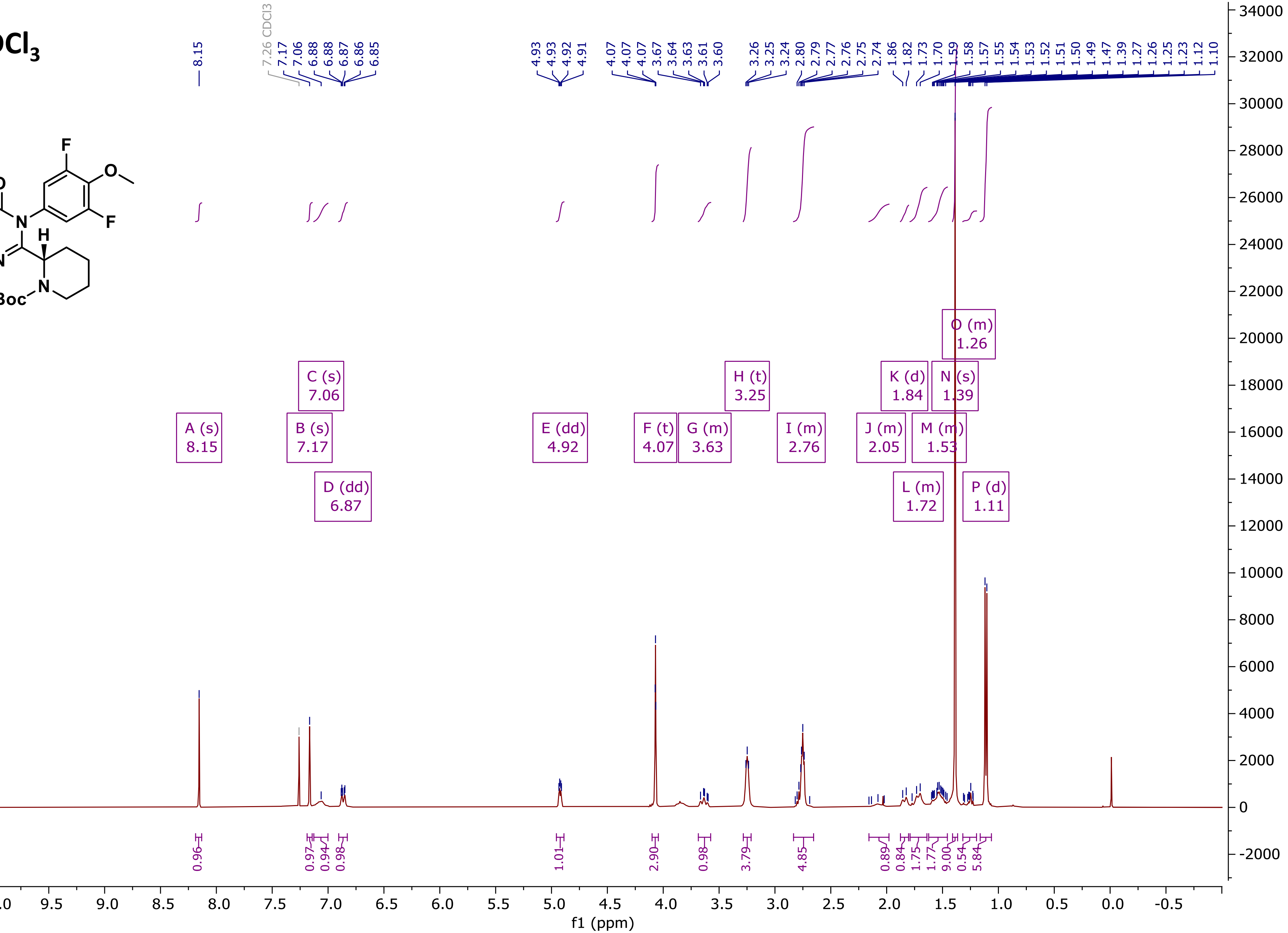

101 MHz, $CDCl_3$

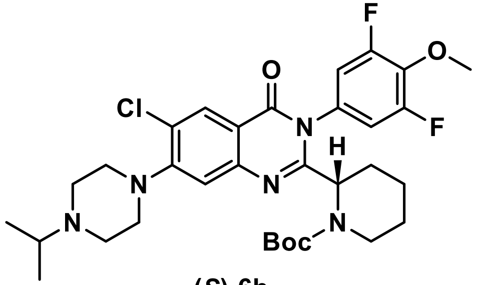

(*S*)-6h

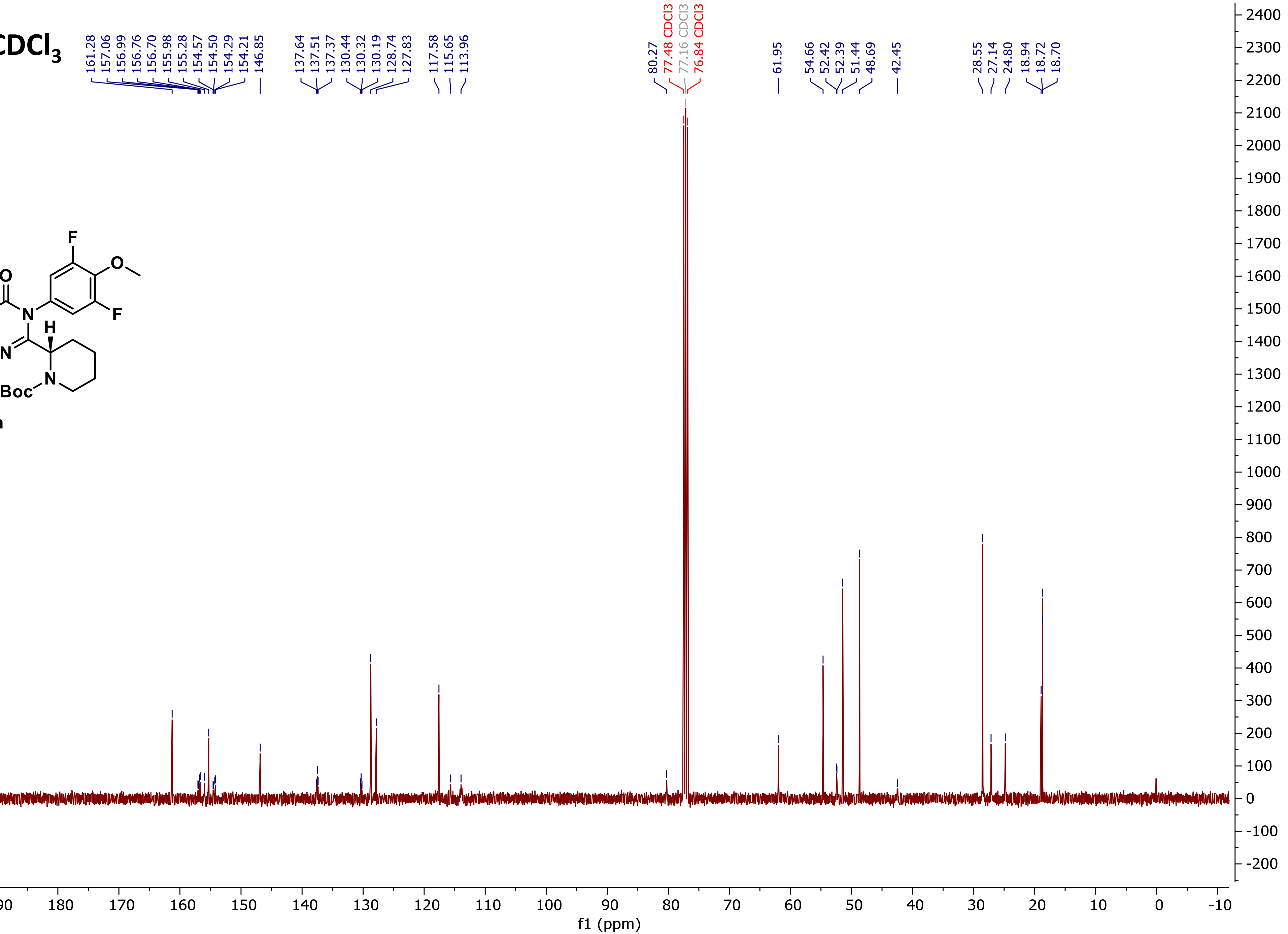

400 MHz, $CDCl_3$

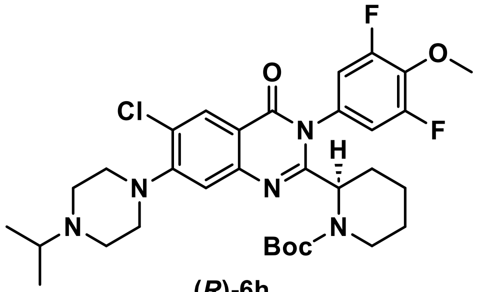

(*R*)-6h

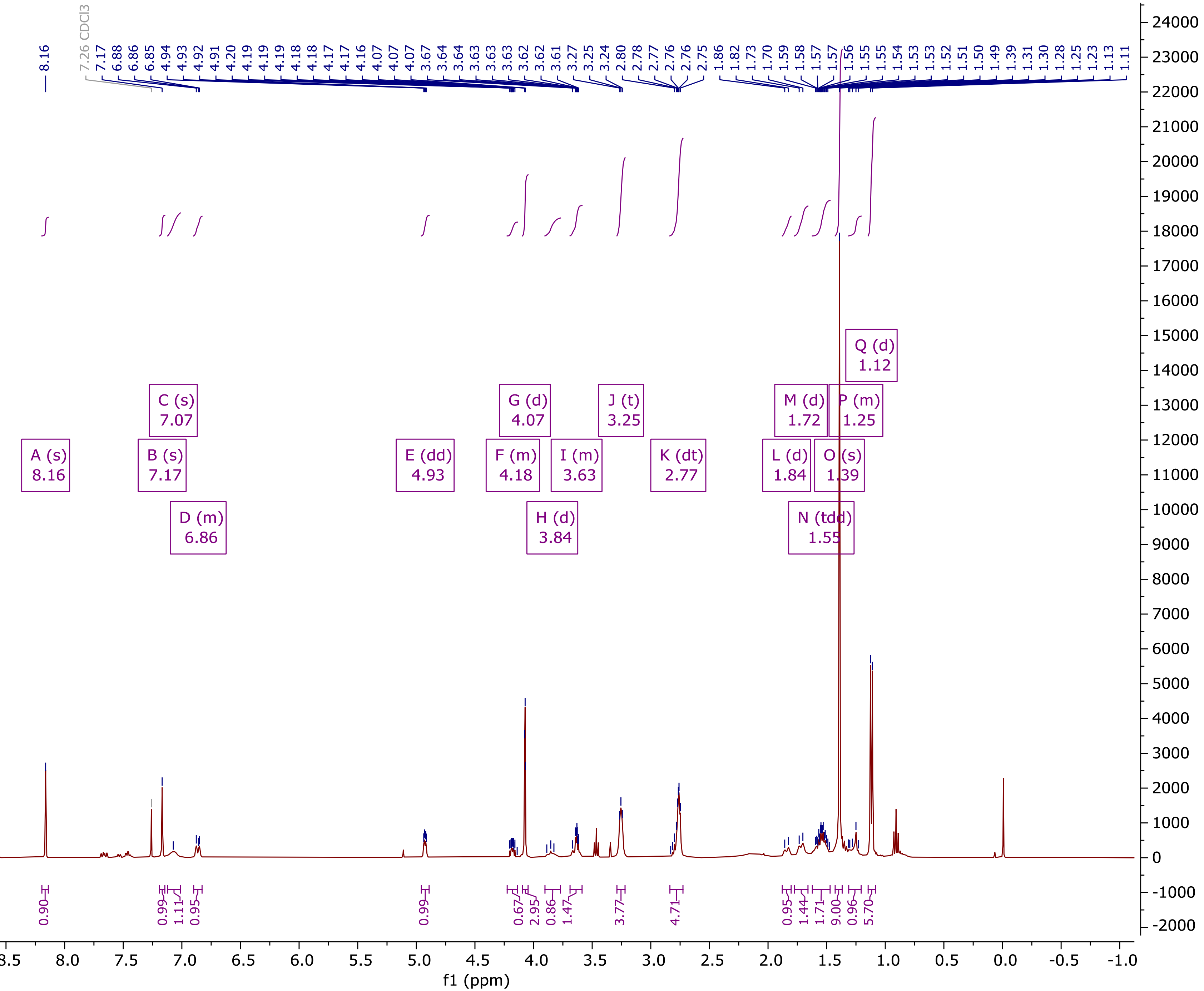

101 MHz, $CDCl_3$

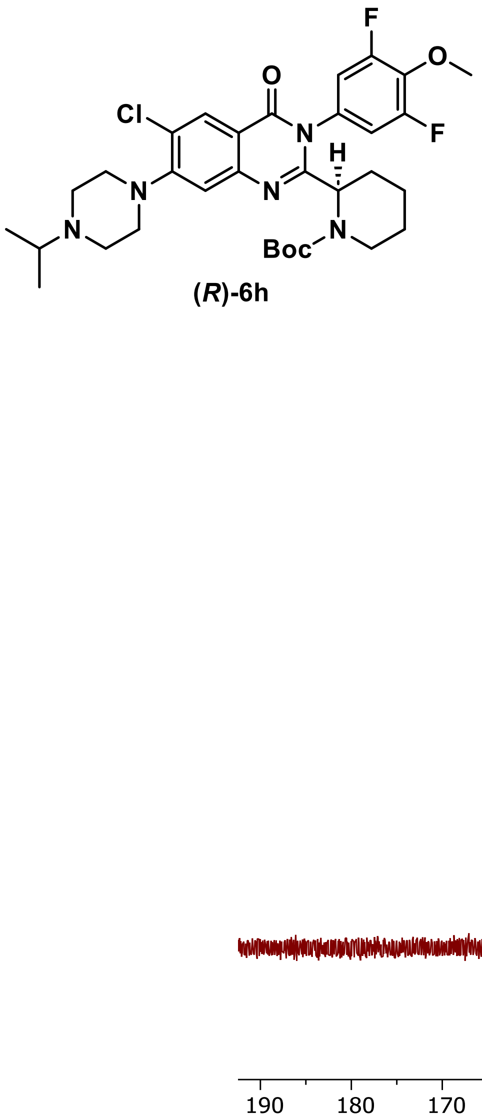

(*R*)-6h

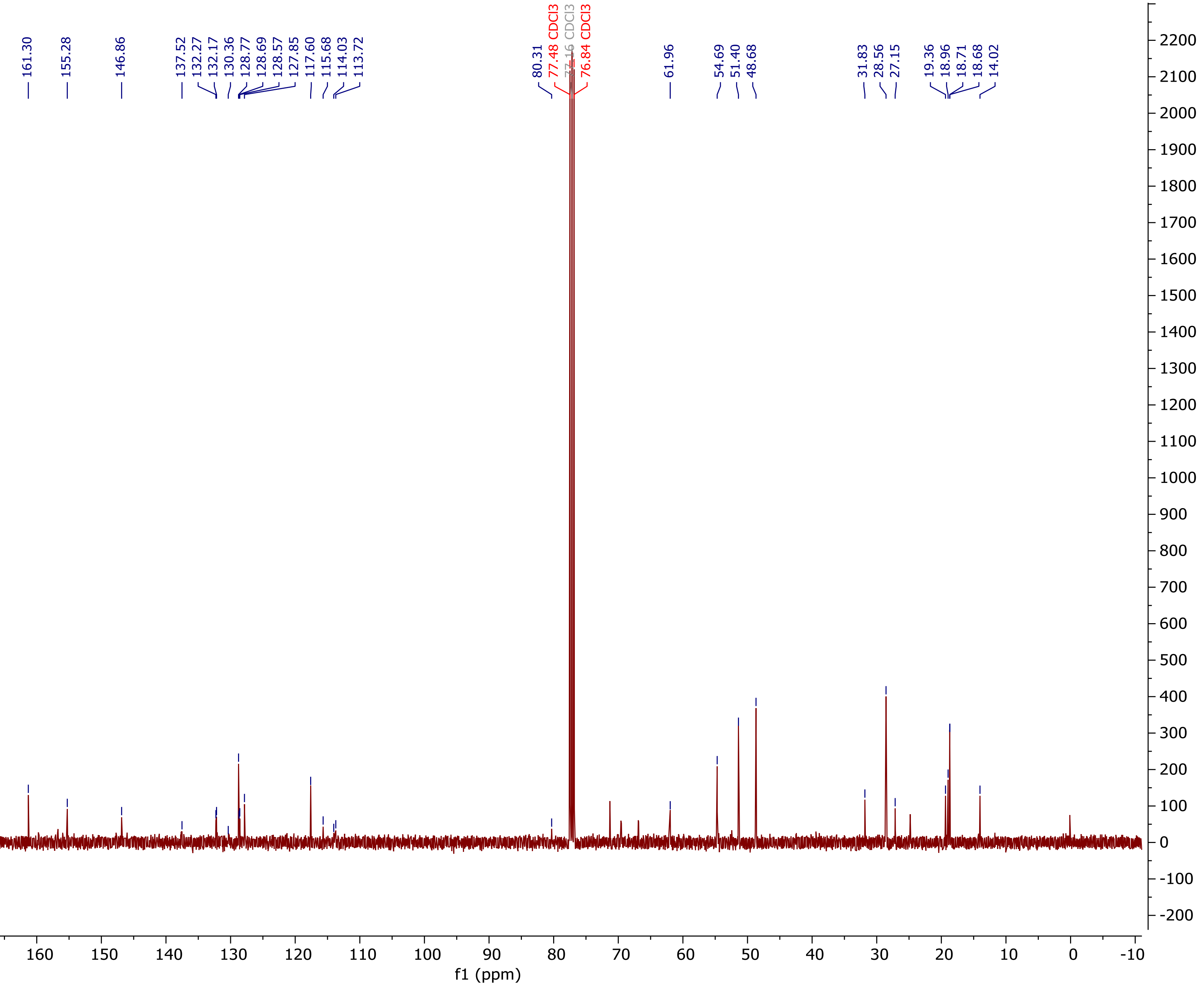

400 MHz, $CDCl_3$

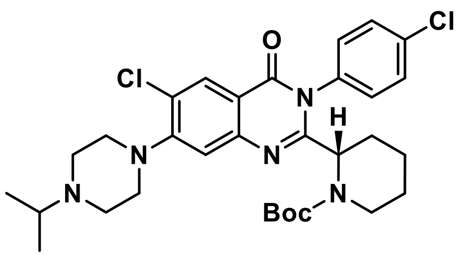

(*S*)-6x

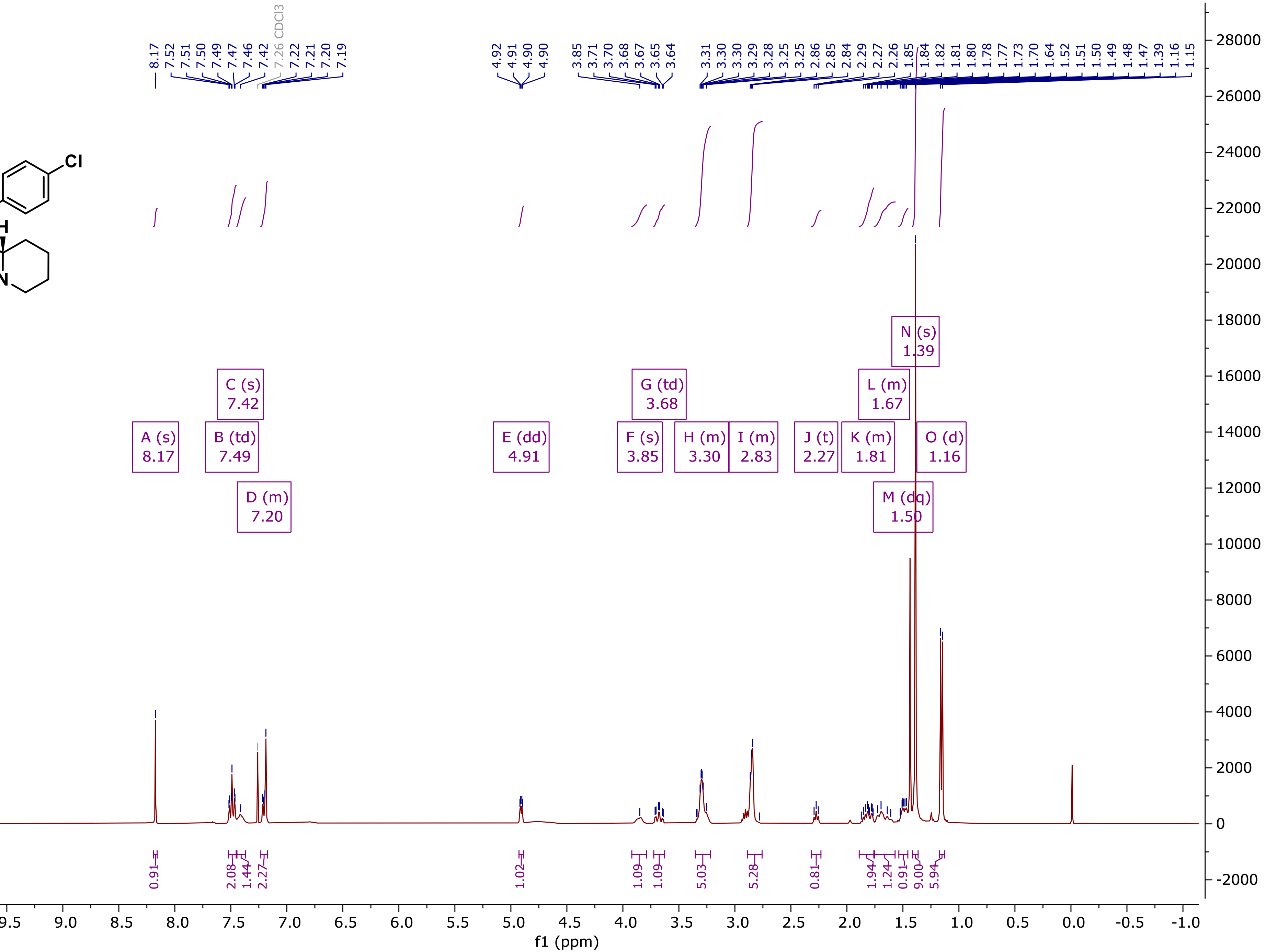

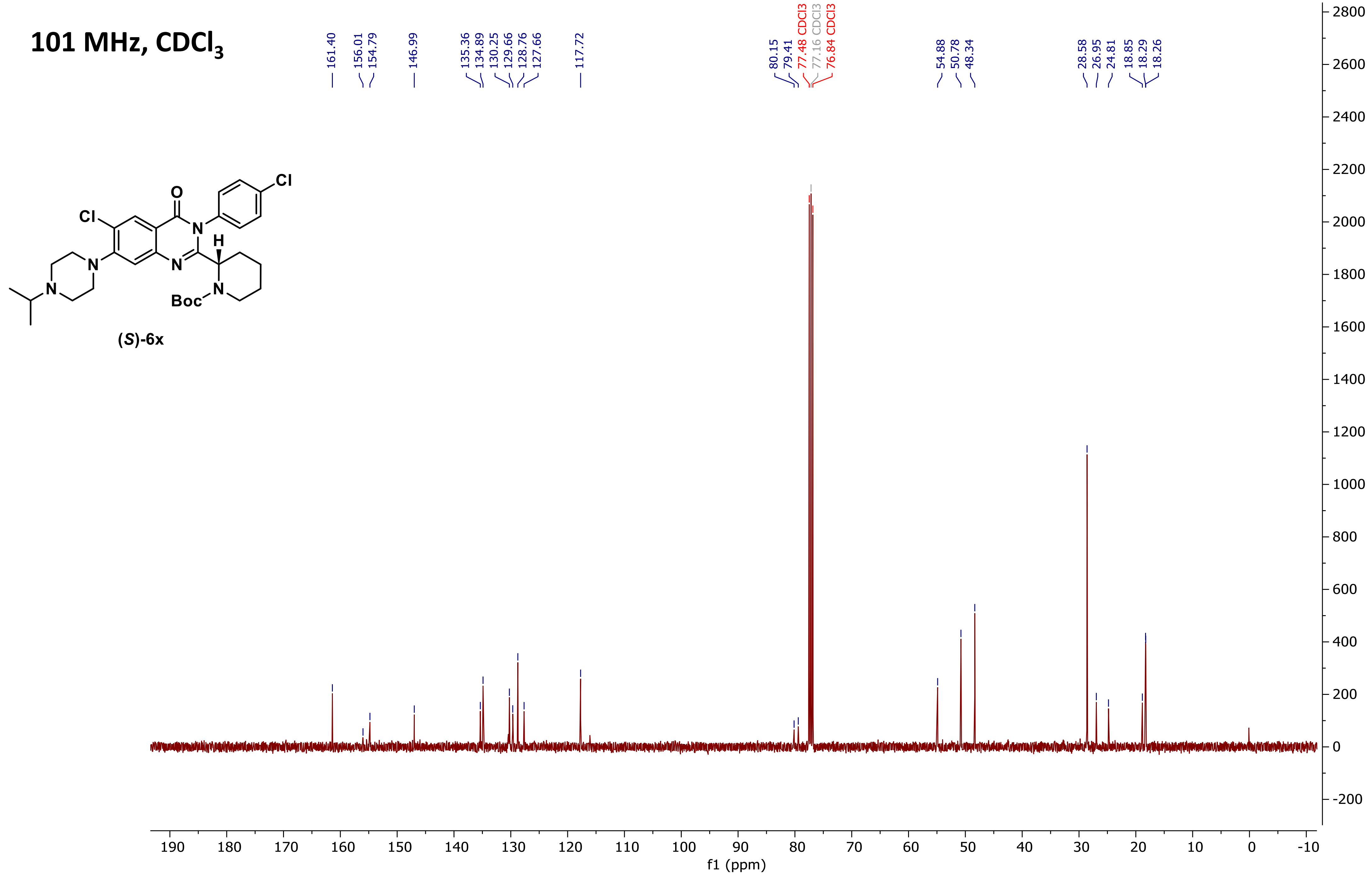

101 MHz, CDCl3
161.40
156.01
154.79
146.99
135.36
134.89
130.25
129.66
128.76
127.66
117.72
80.15
79.41
77.48 CDCl3
77.16 CDCl3
76.84 CDCl3
54.88
50.78
48.34
28.58
26.95
24.81
18.85
18.29
18.26
O
Cl
N
H
N
N
N
Boc
N
Cl
(S)-6x
f1 (ppm)

400 MHz, $CDCl_3$

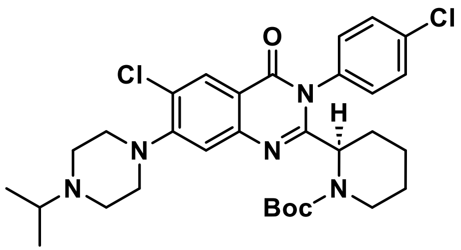

(*R*)-6x

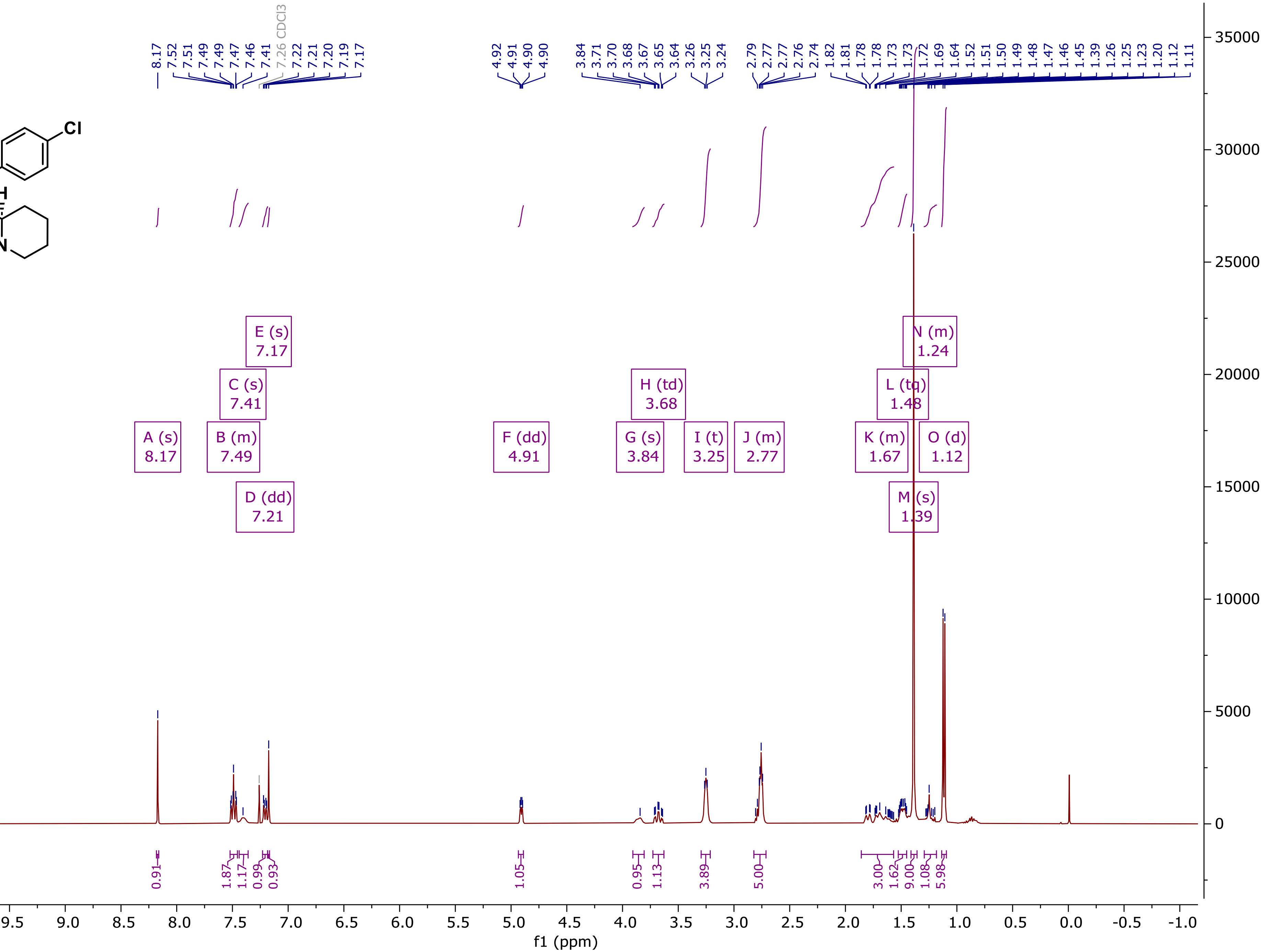

101 MHz, $CDCl_3$

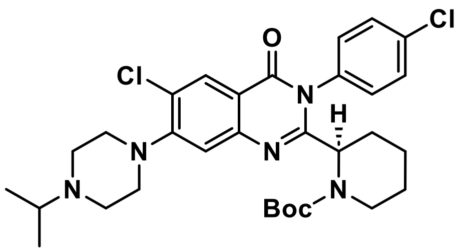

(*R*)-6x

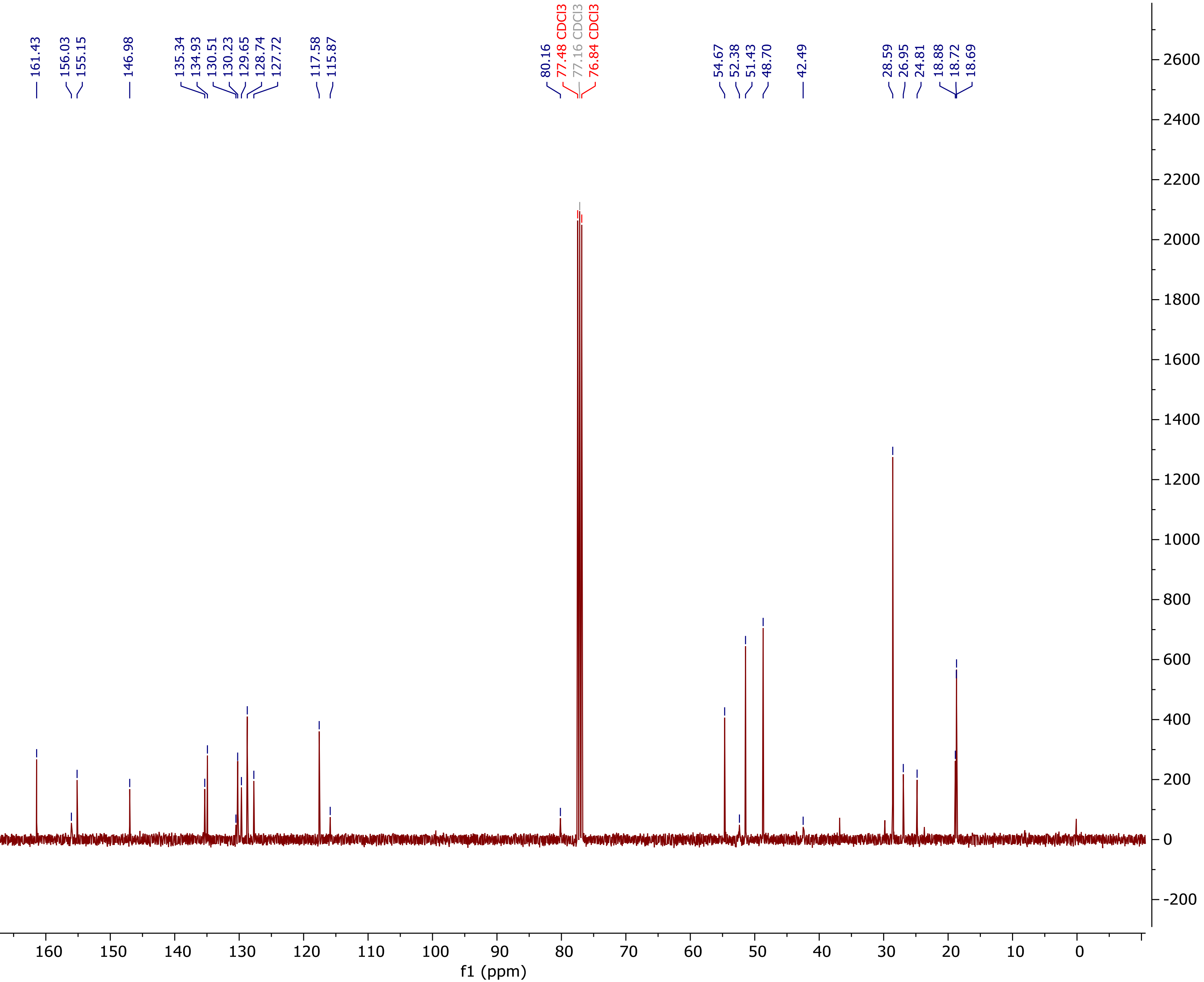

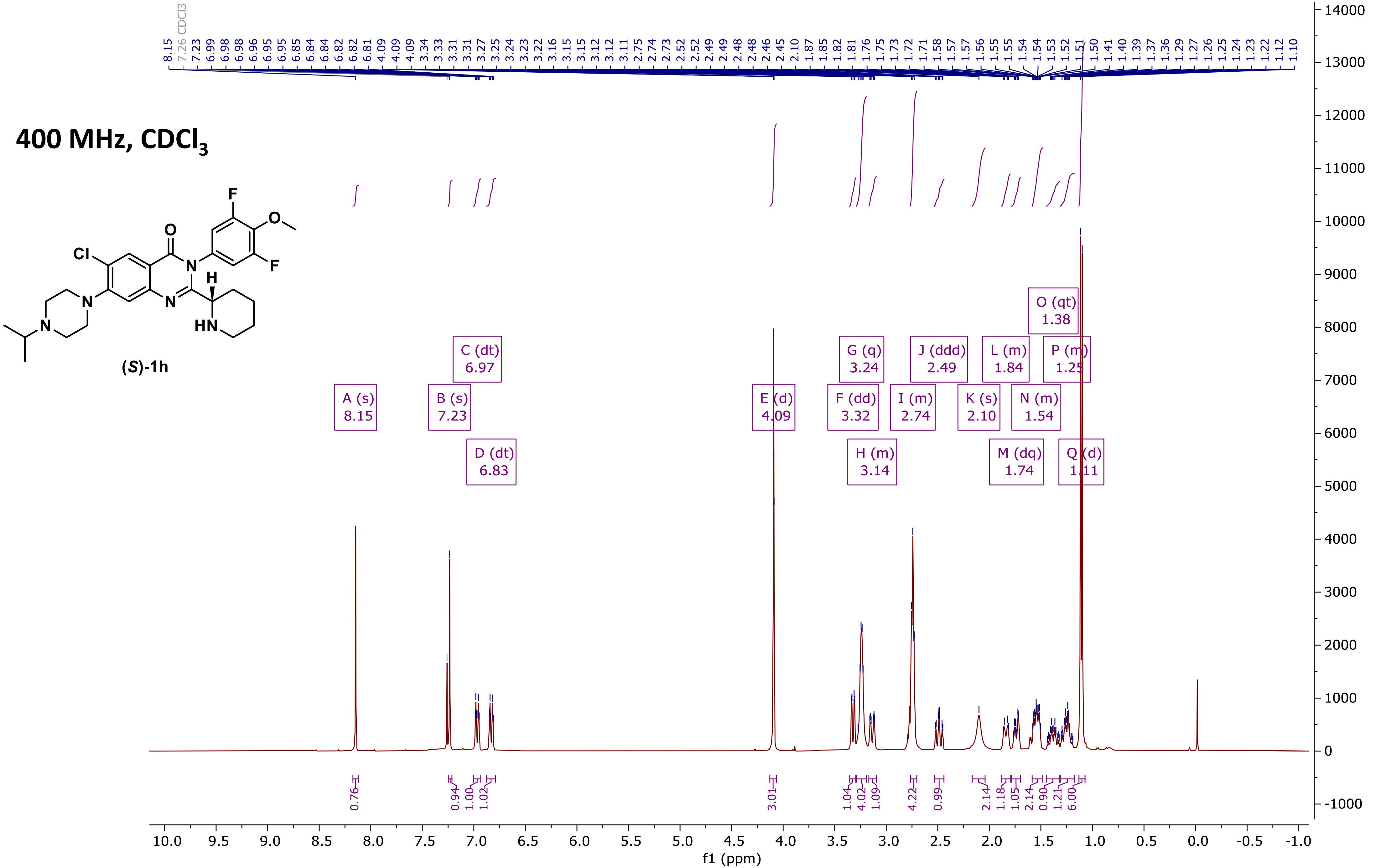
400 MHz, $CDCl_3$
(S)-1h
8.15 7.26 CDCl3 7.23 6.99 6.98 6.98 6.96 6.95 6.95 6.85 6.84 6.84 6.82 6.82 6.81 4.09 4.09 4.09 3.34 3.33 3.31 3.31 3.27 3.25 3.24 3.23 3.22 3.16 3.15 3.15 3.12 3.11 2.75 2.74 2.73 2.52 2.52 2.49 2.49 2.48 2.48 2.46 2.45 2.10 1.87 1.85 1.82 1.81 1.76 1.75 1.73 1.72 1.71 1.58 1.57 1.57 1.56 1.55 1.55 1.54 1.54 1.53 1.52 1.51 1.50 1.41 1.40 1.39 1.37 1.36 1.29 1.27 1.26 1.25 1.24 1.23 1.22 1.12 1.10
A (s) 8.15
B (s) 7.23
C (dt) 6.97
D (dt) 6.83
E (d) 4.09
F (dd) 3.32
G (q) 3.24
H (m) 3.14
I (m) 2.74
J (ddd) 2.49
K (s) 2.10
L (m) 1.84
M (dq) 1.74
N (m) 1.54
O (qt) 1.38
P (m) 1.25
Q (d) 1.11
0.76 0.94 1.00 1.02 3.01 1.04 4.02 1.09 4.22 0.99 2.14 1.18 1.05 2.14 0.90 1.21 6.00
f1 (ppm)

101 MHz, $CDCl_3$

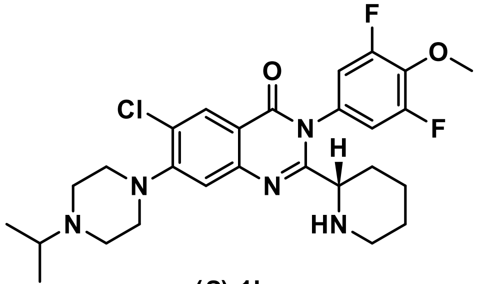

(*S*)-1h

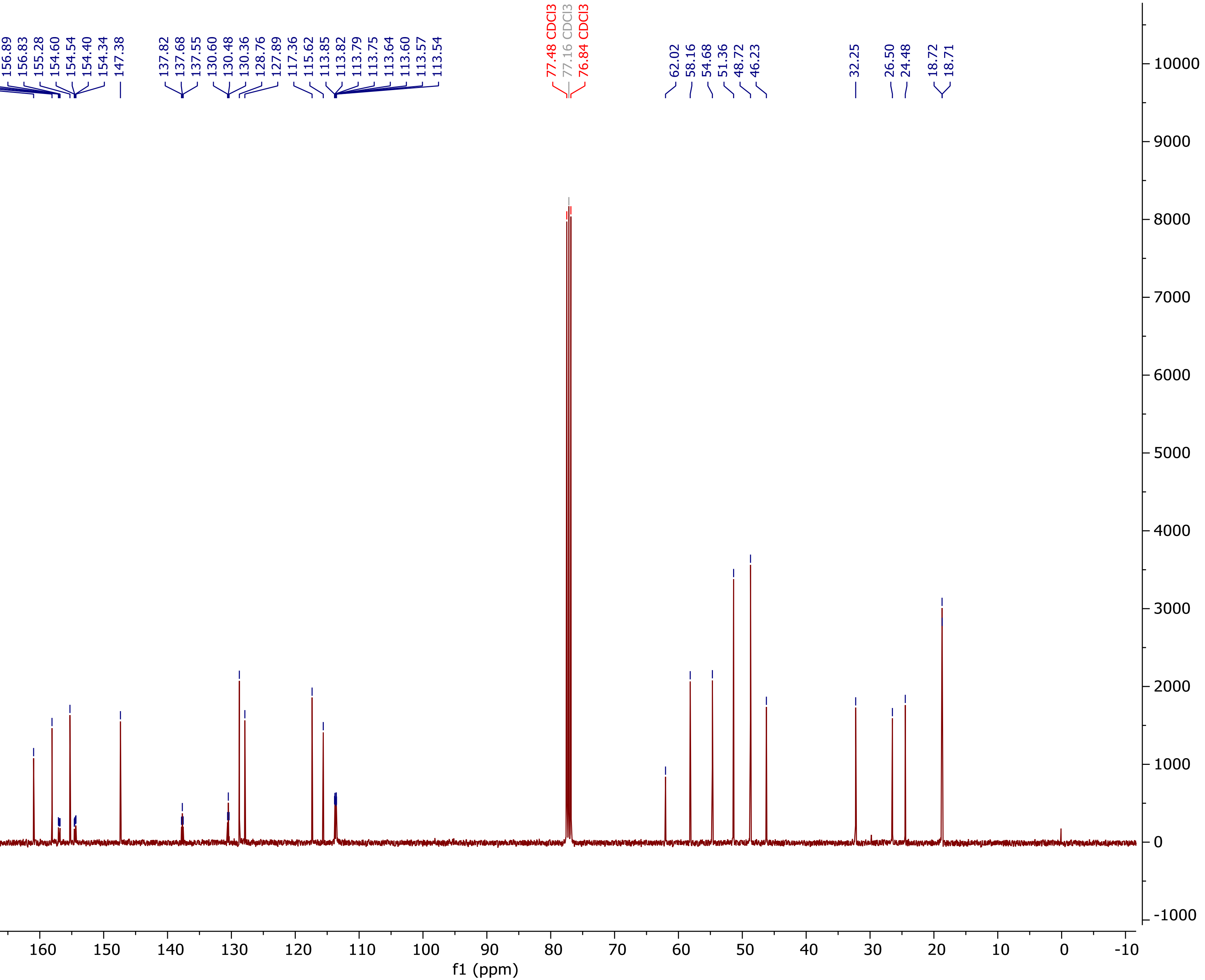

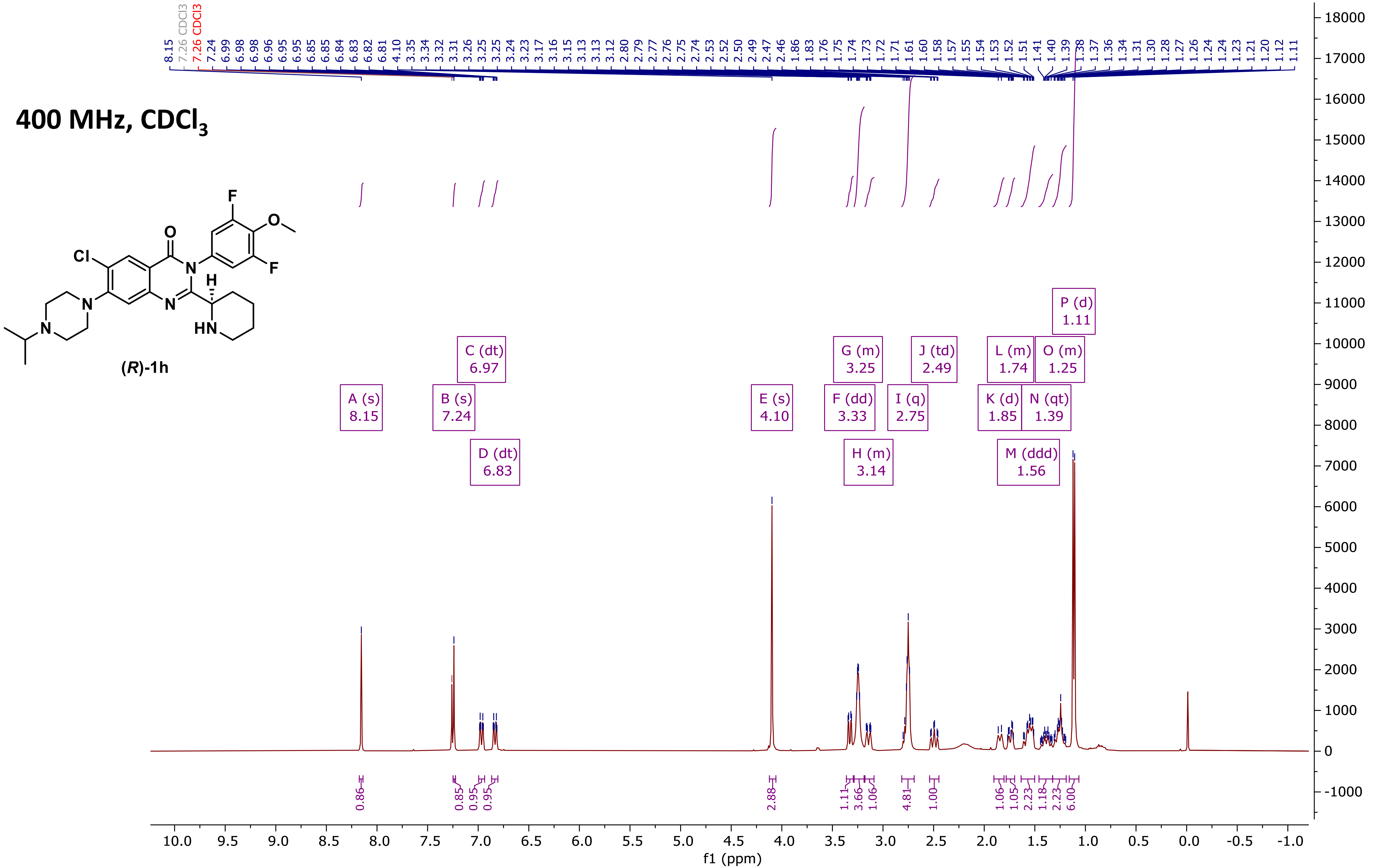
400 MHz, $CDCl_3$
(R)-1h
8.15 7.26 CDCl3 7.26 CDCl3 7.24 6.99 6.98 6.98 6.96 6.95 6.95 6.85 6.85 6.84 6.83 6.82 6.81 4.10 3.35 3.34 3.32 3.31 3.26 3.25 3.25 3.24 3.23 3.17 3.16 3.15 3.13 3.13 3.12 2.80 2.79 2.77 2.76 2.75 2.74 2.53 2.52 2.50 2.49 2.47 2.46 1.86 1.83 1.76 1.75 1.74 1.73 1.72 1.71 1.61 1.60 1.58 1.57 1.55 1.54 1.53 1.52 1.51 1.41 1.40 1.39 1.38 1.37 1.36 1.34 1.31 1.30 1.28 1.27 1.26 1.24 1.24 1.23 1.21 1.20 1.12 1.11
A (s) 8.15
B (s) 7.24
C (dt) 6.97
D (dt) 6.83
E (s) 4.10
F (dd) 3.33
G (m) 3.25
H (m) 3.14
I (q) 2.75
J (td) 2.49
K (d) 1.85
L (m) 1.74
M (ddd) 1.56
N (qt) 1.39
O (m) 1.25
P (d) 1.11
0.86 0.85 0.95 0.95 2.88 1.11 3.66 1.06 4.81 1.00 1.06 1.05 2.23 1.18 2.23 6.00
f1 (ppm)

376 MHz, CDCl$_3$

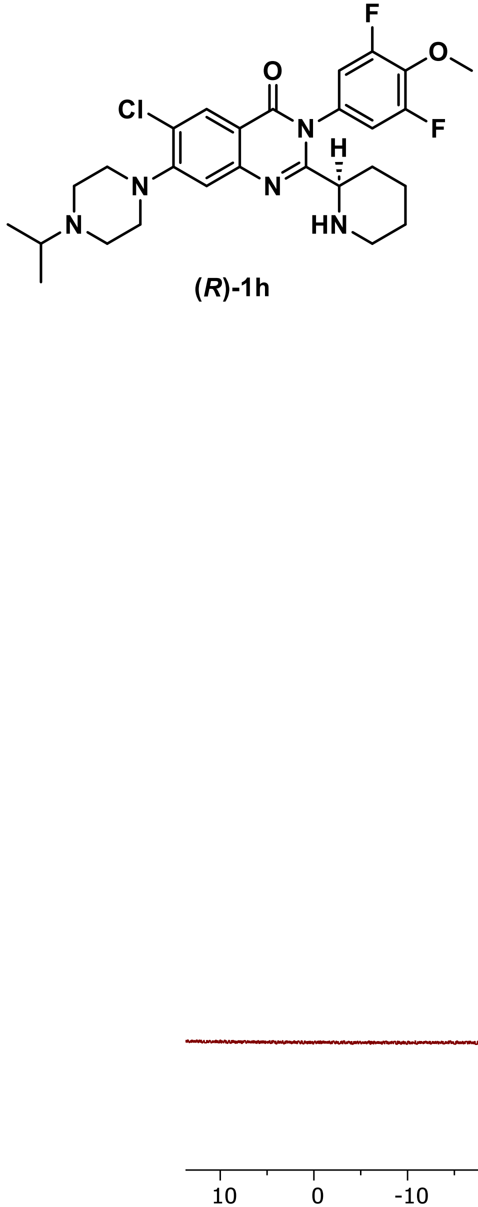

(*R*)-1h

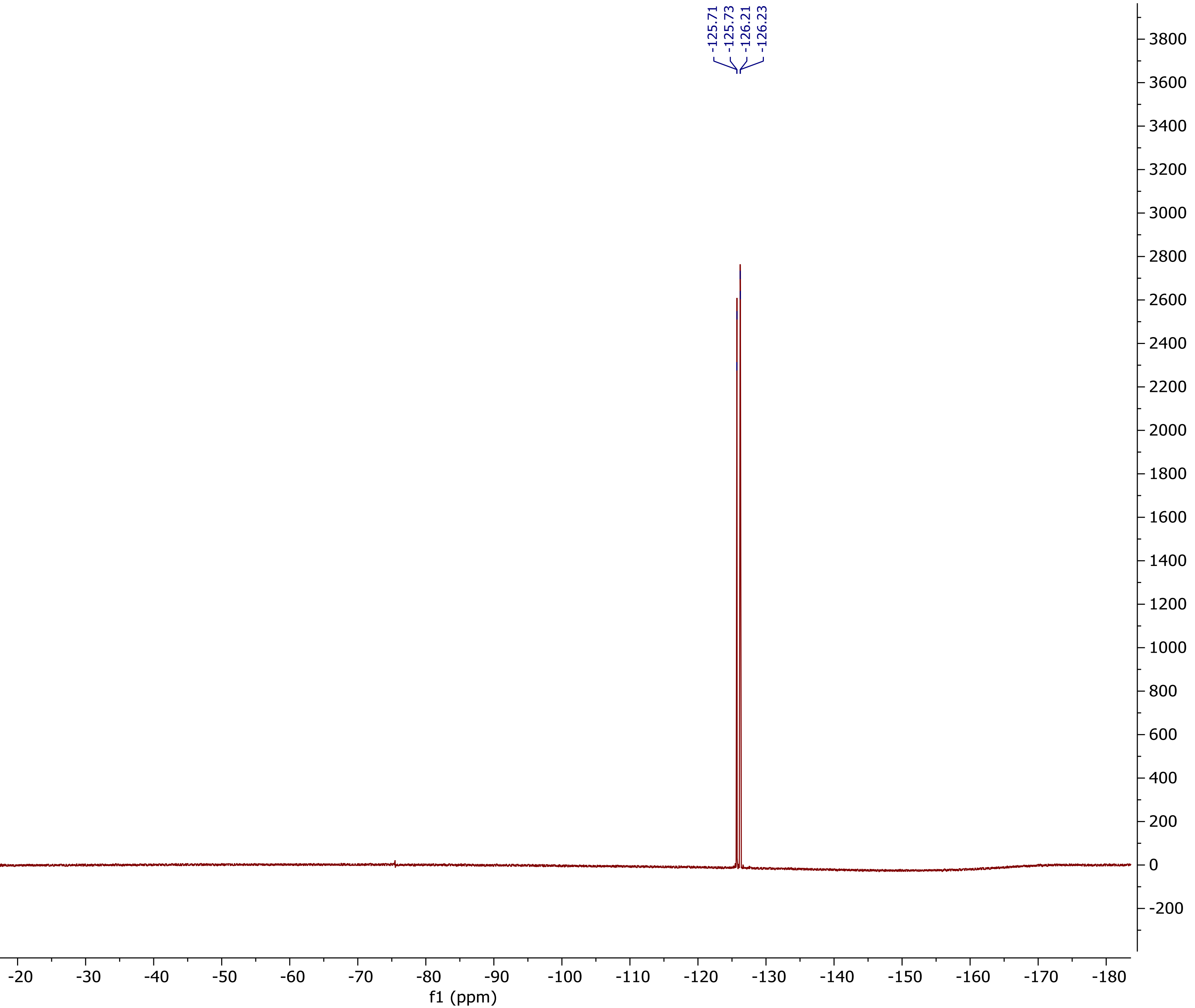

101 MHz, $CDCl_3$

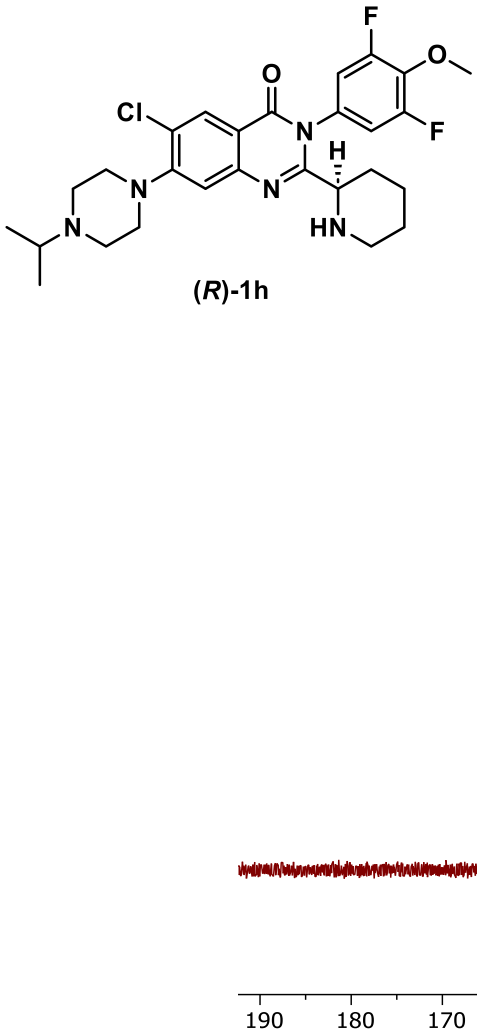

(*R*)-1h

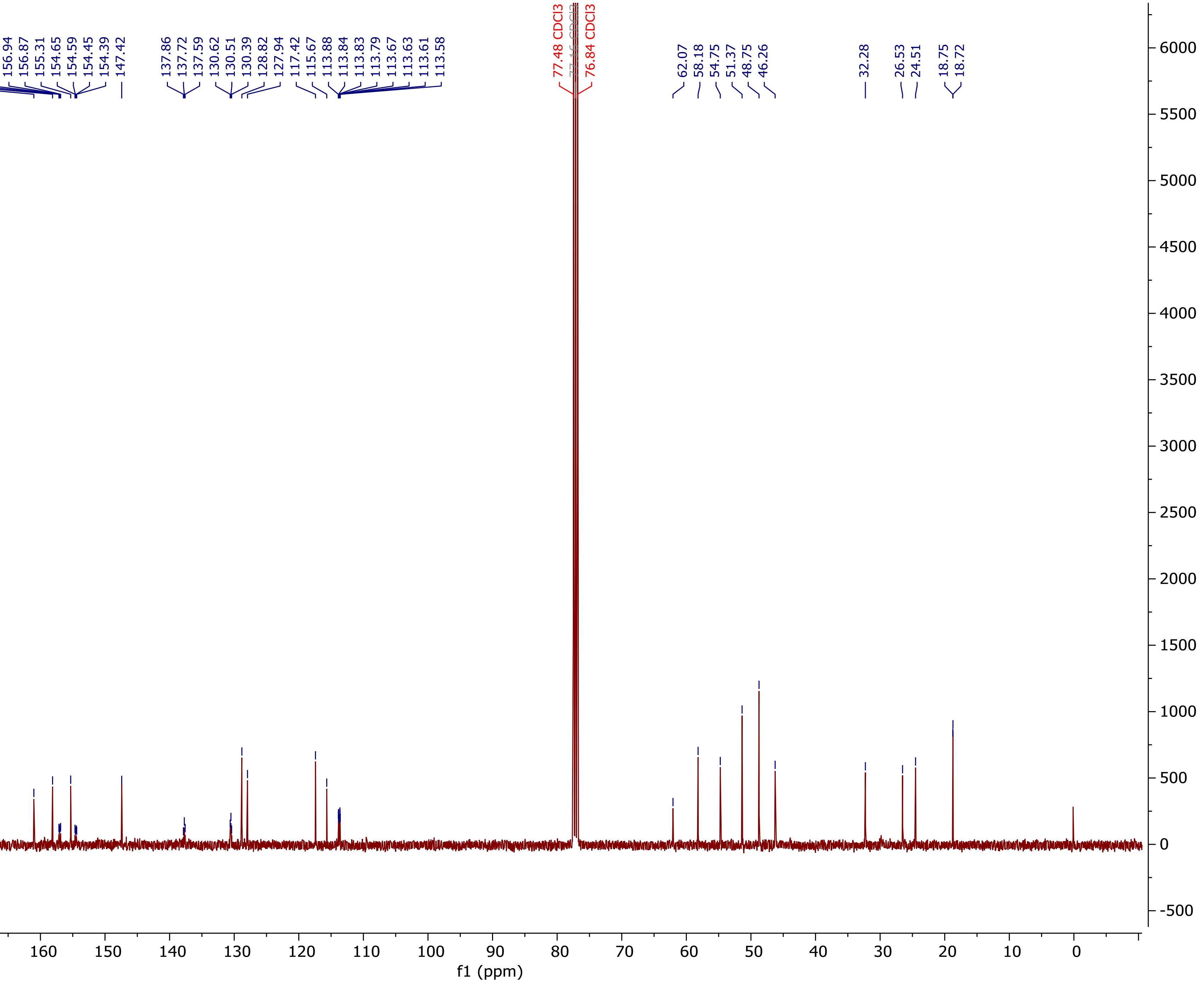

400 MHz, $CDCl_3$

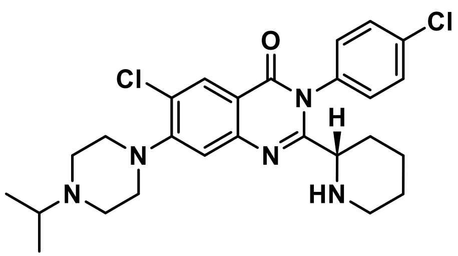

(*S*)-1x

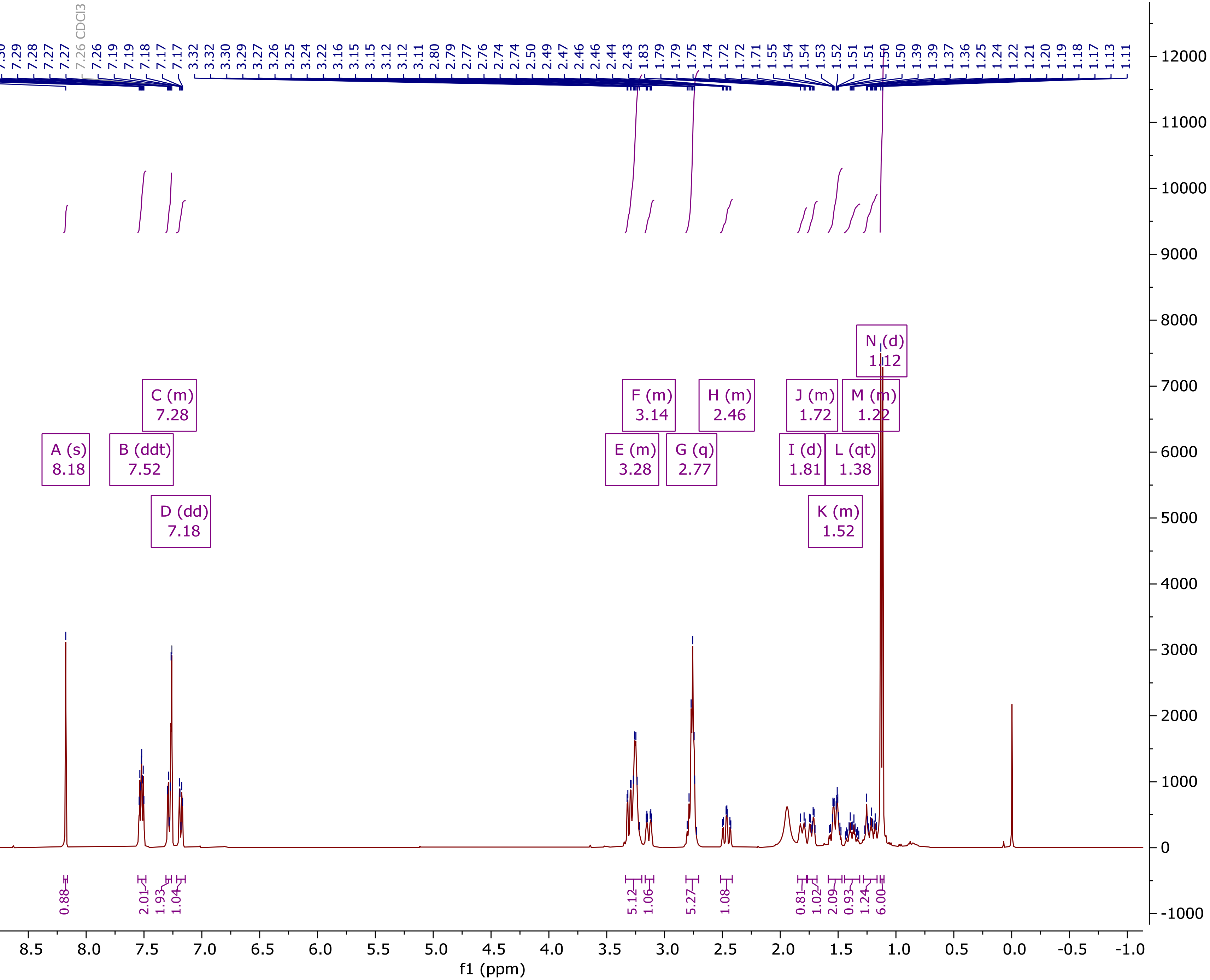

101 MHz, $CDCl_3$

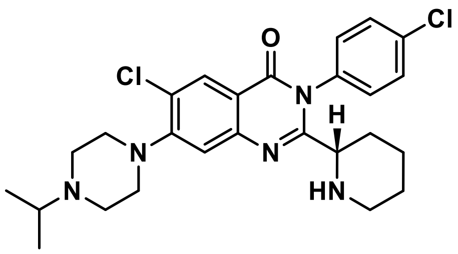

(*S*)-1x

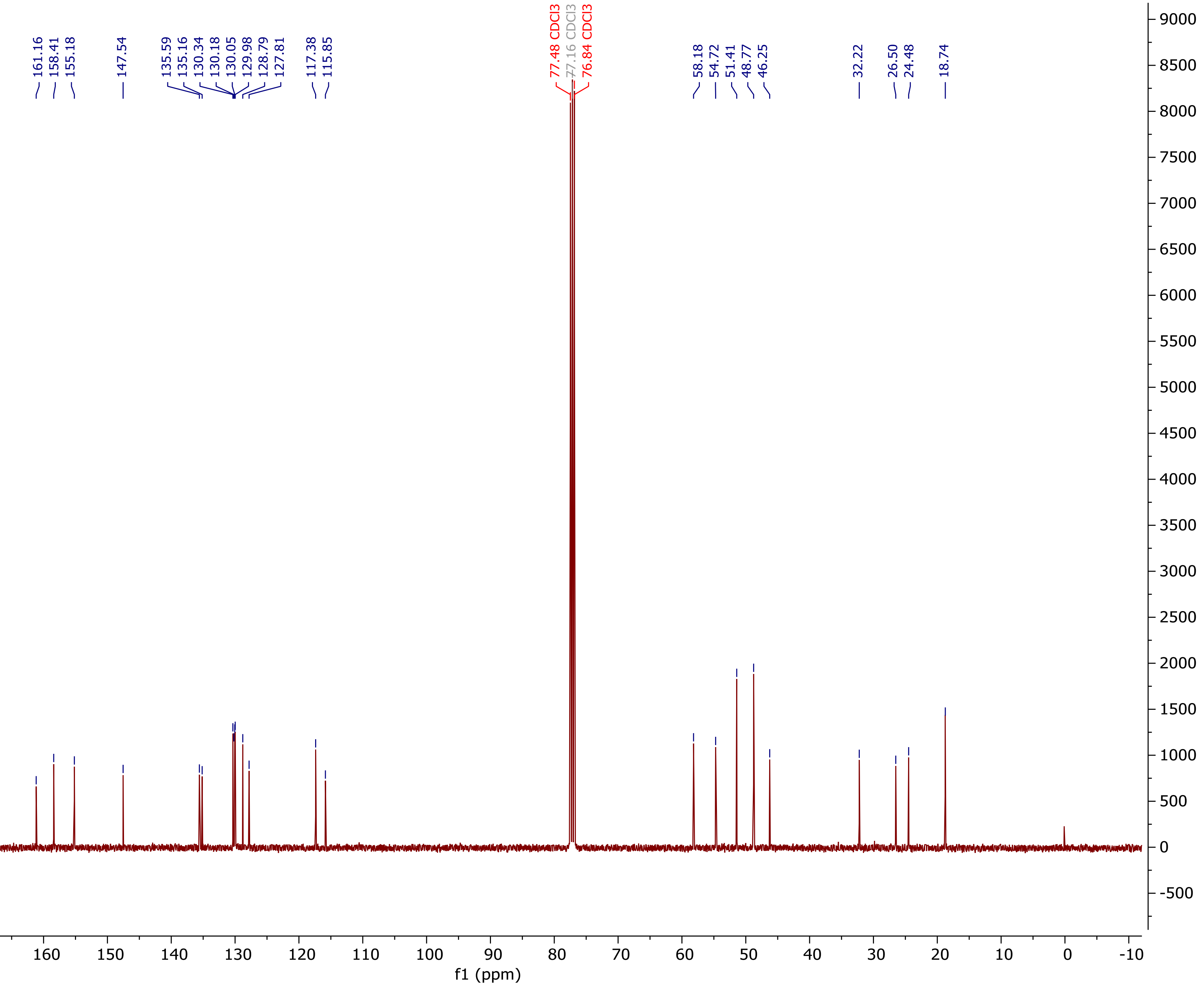

400 MHz, $CDCl_3$

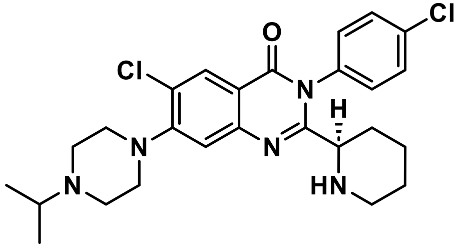

(*R*)-1x

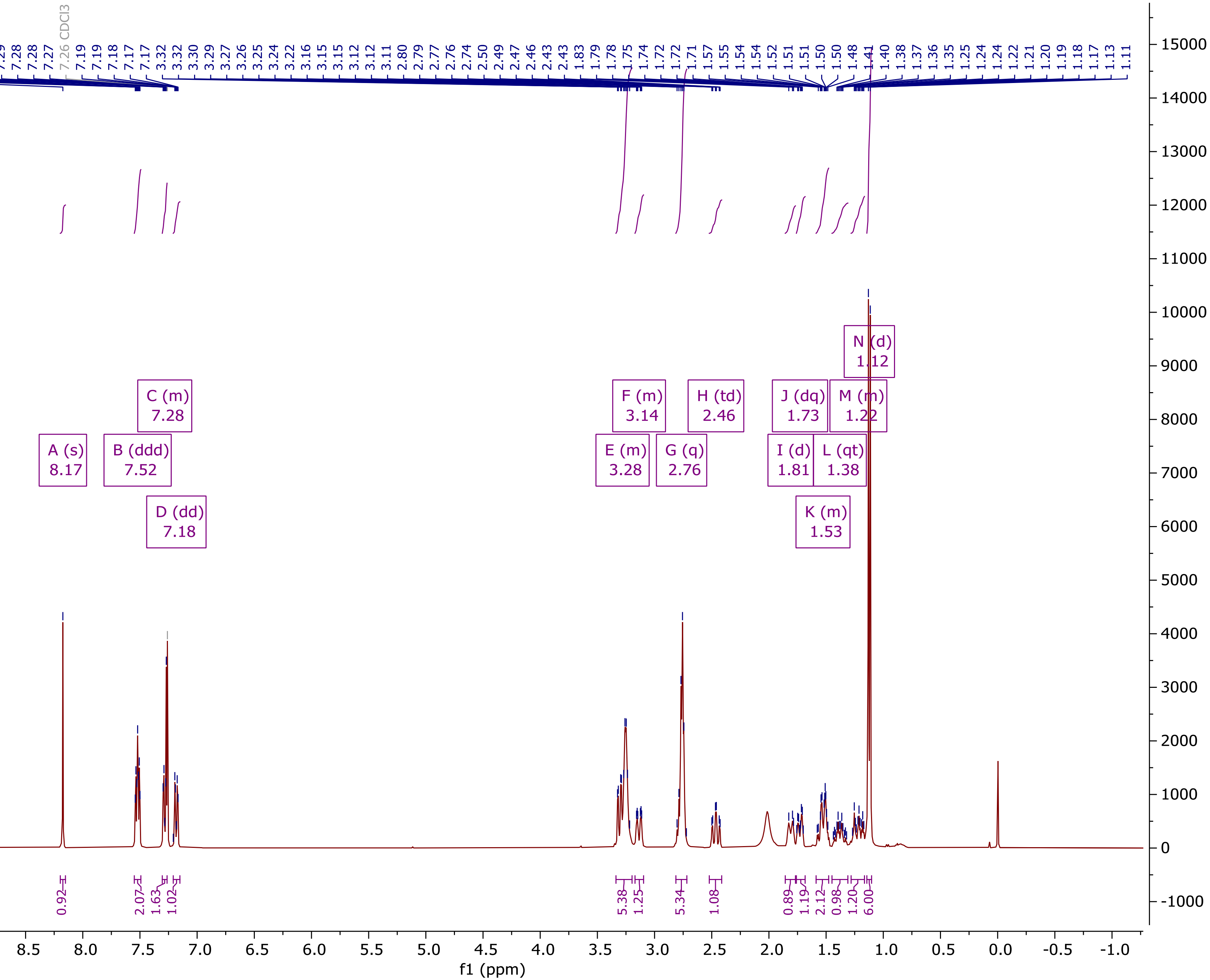

101 MHz, $CDCl_3$

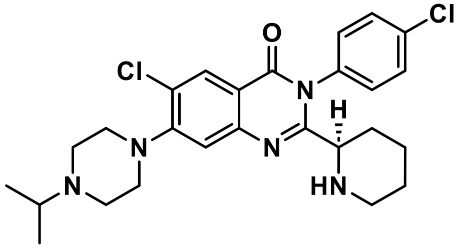

(*R*)-1x

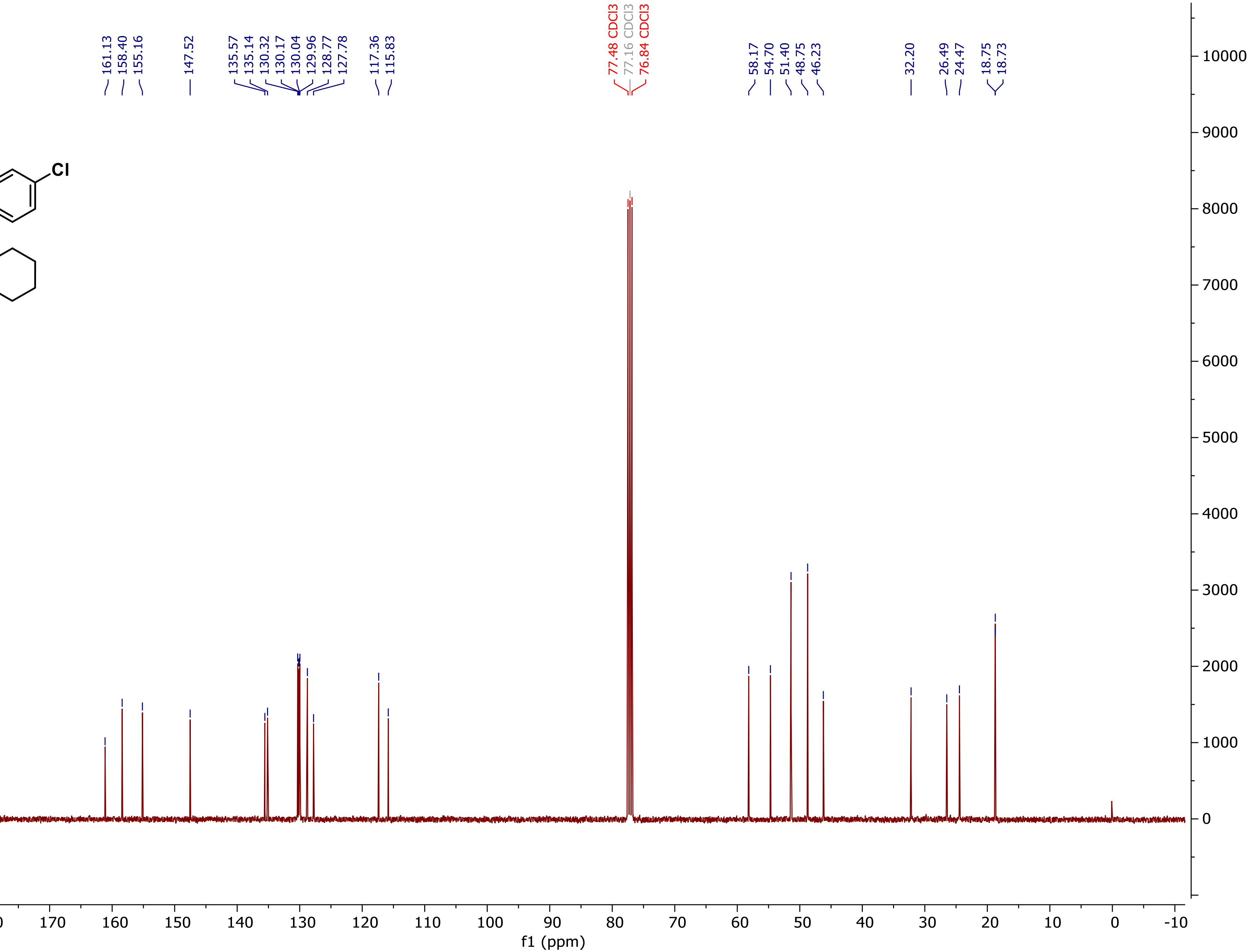

400 MHz, $CDCl_3$

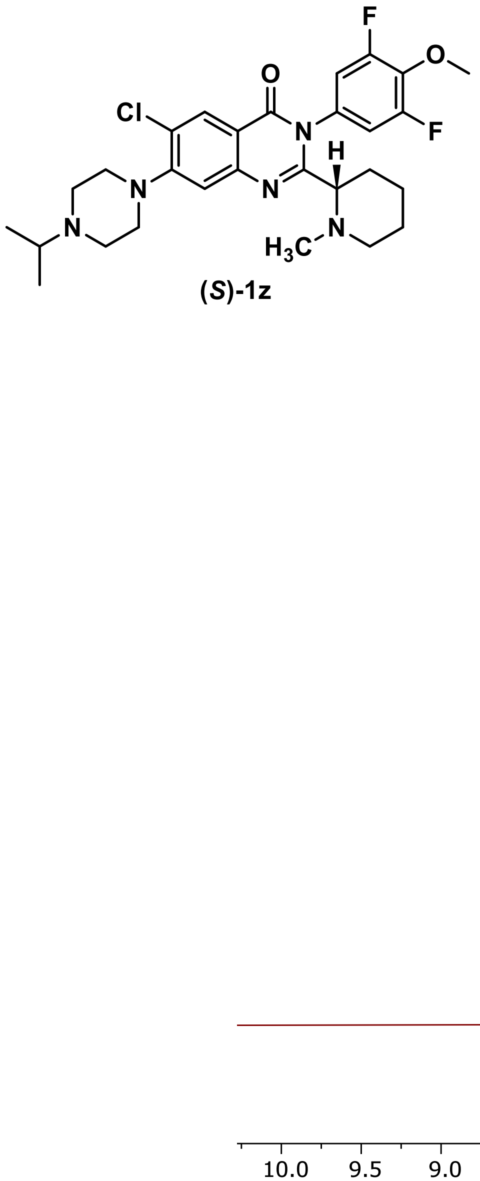

(*S*)-1z

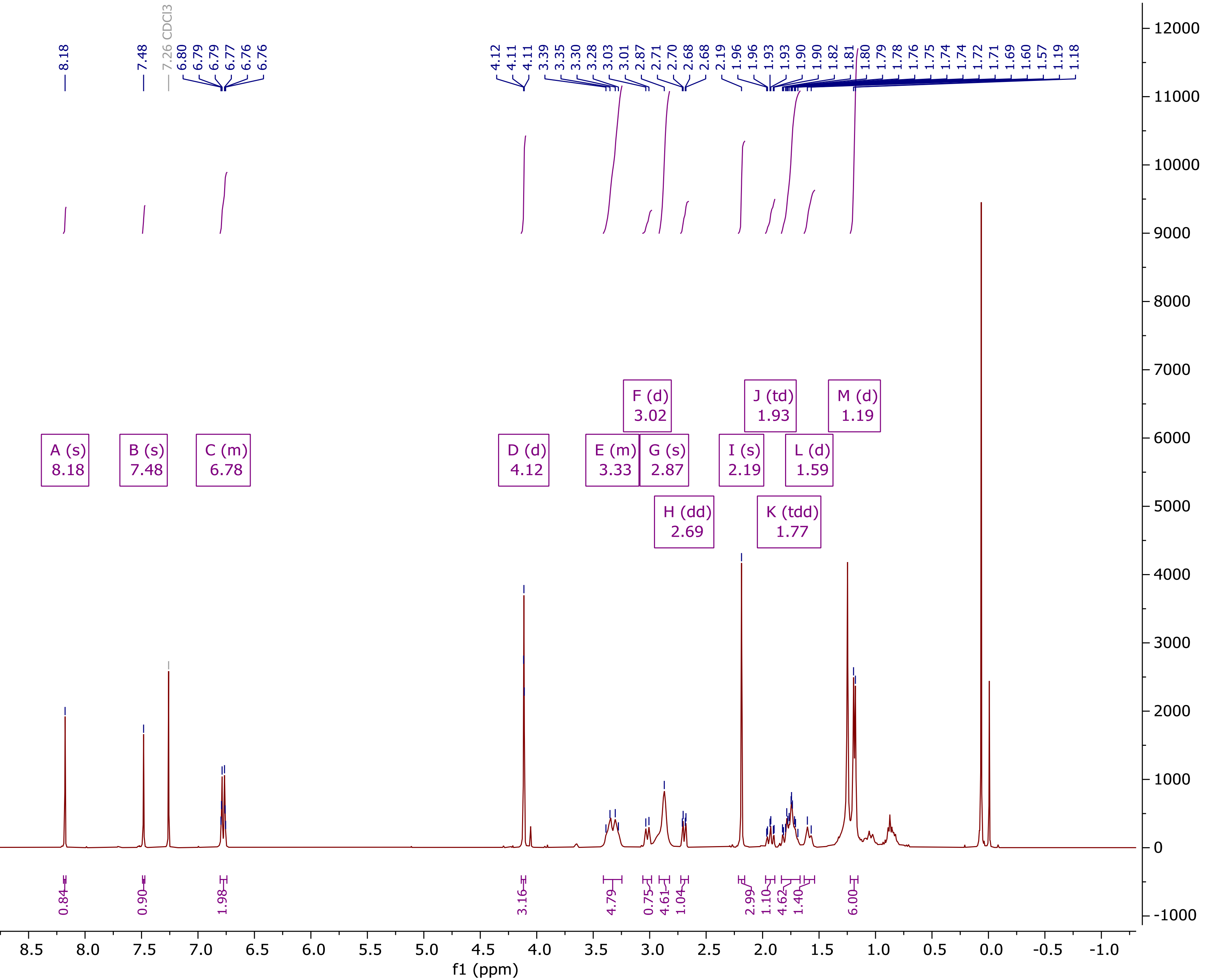

\

400 MHz, $CDCl_3$

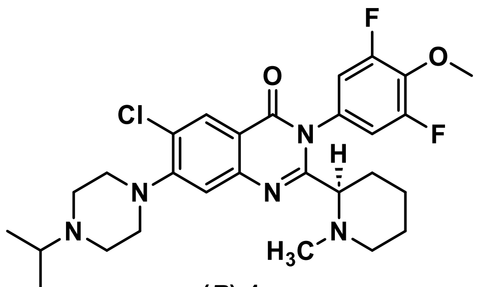

(*R*)-1z

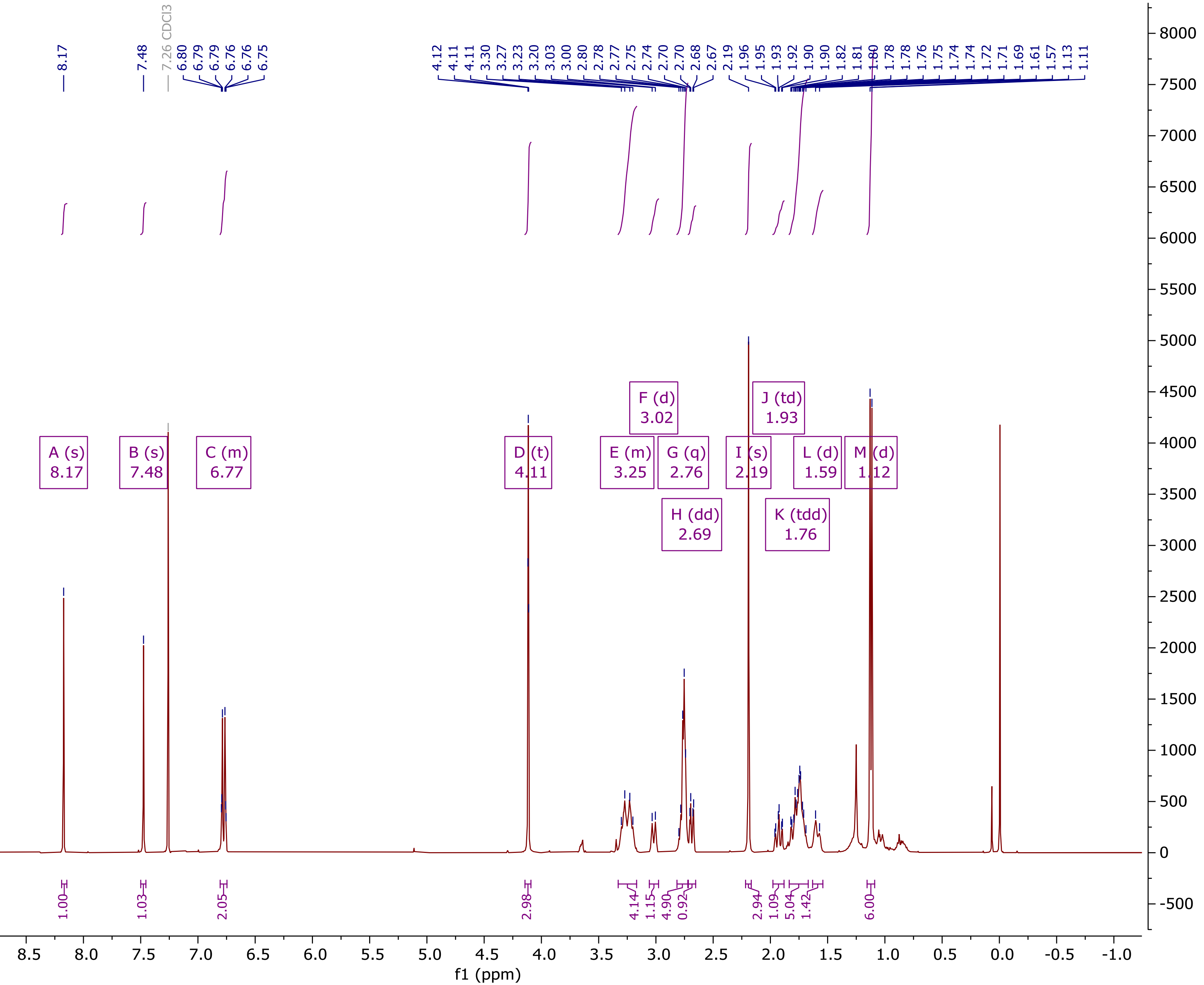

Supplement: Supplementary file 1 [file ml5c00515_si_001.pdf]
